# A cardiac fibroblast-enriched micropeptide regulates inflammation in ischemia/reperfusion injury

**DOI:** 10.1172/jci.insight.187848

**Published:** 2025-03-20

**Authors:** Youchen Yan, Tingting Zhang, Xin He, Tailai Du, Gang Dai, Xingfeng Xu, Zhuohui Chen, Jialing Wu, Huimin Zhou, Yazhi Peng, Yan Li, Chen Liu, Xinxue Liao, Yugang Dong, Jing-song Ou, Zhan-Peng Huang

**Affiliations:** 1 Department of Cardiology, Center for Translational Medicine, Institute of Precision Medicine, The First Affiliated Hospital, Sun Yat-sen University, Guangzhou, China.; 2 Chinese Medicine Guangdong Laboratory, Guangdong Hengqin, China.; 3 NHC Key Laboratory of Assisted Circulation and Vascular Diseases, Sun Yat-sen University, Guangzhou, China.; 4 Division of Cardiac Surgery, National-Guangdong Joint Engineering Laboratory for Diagnosis and Treatment of Vascular Diseases, The First Affiliated Hospital, Sun Yat-sen University, Guangzhou, China.; 5 Key Laboratory of Assisted Circulation and Vascular Diseases, Chinese Academy of Medical Sciences, Guangzhou, China.

**Keywords:** Cardiology, Cell biology, Cardiovascular disease

## Abstract

Inflammation is a critical pathological process in myocardial infarction. Although immunosuppressive therapies can mitigate inflammatory responses and improve outcomes in myocardial infarction, they also increase the risk of infections. Identifying novel regulators of local cardiac inflammation could provide safer therapeutic targets for myocardial ischemia/reperfusion injury. In this study, we identified a previously uncharacterized micropeptide, which we named Inflammation Associated MicroPeptide (IAMP). IAMP is predominantly expressed in cardiac fibroblasts, and its expression is closely associated with cardiac inflammation. Downregulation of IAMP promotes, whereas its overexpression prevents, the transformation of cardiac fibroblasts into a more inflammatory phenotype under stressed/stimulated conditions, as evidenced by changes in the expression and secretion of proinflammatory cytokines. Consequently, loss of IAMP function leads to uncontrolled inflammation and worsens cardiac injury following ischemia/reperfusion surgery. Mechanistically, IAMP promotes the degradation of HIF-1α by interacting with its stabilizing partner HSP90 and, thus, suppresses the transcription of proinflammatory genes downstream of HIF-1α. This study underscores the significance of fibroblast-mediated inflammation in cardiac ischemia/reperfusion injury and highlights the therapeutic potential of targeting micropeptides for myocardial infarction.

## Abstract 2

A micropeptide IAMP modulates cardiac inflammation through promoting the degradation of HIF-1α by interacting with its stabilizing partner HSP90 in cardiac fibroblasts

## Graphical abstract

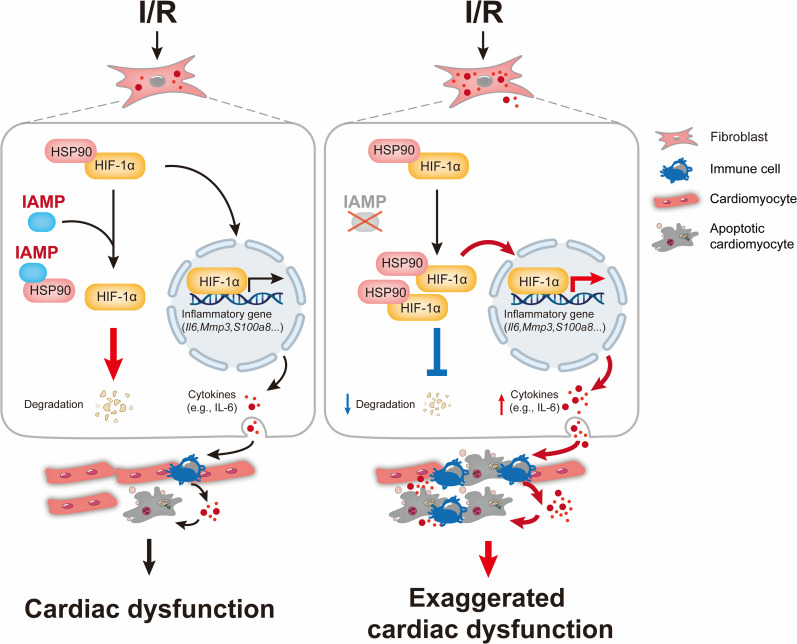

## Introduction

Myocardial infarction (MI) stands as one of the leading causes of cardiovascular mortality, claiming over 2.4 million lives annually in the United States and more than 4 million lives in Europe and northern Asia (1). Despite the establishment of modern medical standards for managing MI, its long-term prognosis remains poor (2), primarily due to the irreversible nature of ischemic damage to the heart. Therefore, the identification of novel therapeutic targets is imperative.

Inflammation is a pivotal pathological process orchestrating ischemic/reperfusion injury following MI (3). This notion finds validation in the Canakinumab Anti-inflammatory Thrombosis Outcomes Study trial, where IL-1β inhibition demonstrated improved prognosis in patients with previous MI (4). Conventionally, the inflammatory response is attributed to immune cells such as neutrophils, macrophages, T cells, and B cells. Studies have shown that partially suppressing the function of these cells confers cardioprotection after MI (5–11). However, beyond the regional inflammatory response in the heart, these immune cells serve as defenders against pathogens. Thus, global immunosuppression inherently escalates the risk of infections. Indeed, IL-1β inhibition was associated with increased fatal infections despite its cardiovascular benefits (4). Similarly, the Colchicine Cardiovascular Outcomes Trial revealed that inflammation inhibition via low-dose colchicine improved the prognosis of recent patients with MI but elevated the risk of pneumonia (12). This conundrum poses limitations on the further advancement of antiinflammatory therapy for MI.

Recent investigations have illuminated the role of fibroblasts as key participants in local inflammation. A subset of these fibroblasts actively modulates the inflammatory response in various diseases, including MI (13, 14). In certain contexts, fibroblasts may even exert a more significant influence on inflammation than immune cells. For instance, cardiac fibroblasts (CFs) preferentially secrete IL-6, a pivotal proinflammatory cytokine, in post-MI hearts (15). Thus, these proinflammatory fibroblasts represent a promising therapeutic target capable of suppressing cardiac inflammation while preserving the immune cell’s defensive functions.

Micropeptides, defined as polypeptides shorter than 100 amino acids, have historically been overlooked due to their abbreviated length. However, recent advancements in translatomic analysis techniques have unveiled plenty of micropeptides in mammalian cells (16). These studies have demonstrated the involvement of micropeptides in diverse cellular processes and their regulatory roles in multiple diseases (17–19). Despite these discoveries, the functional repertoire of this molecule class remains largely unexplored. In a prior investigation, we identified a micropeptide, hereafter referred to as Inflammation Associated MicroPeptide (IAMP), associated with cardiovascular disease (20). Our findings indicate an enrichment of IAMP in CFs, hinting at its potential regulatory role within this cellular population. In this study, we unveil that IAMP suppresses the proinflammatory effects of CFs by inhibiting the HIF-1α signaling pathway, thus presenting a promising therapeutic avenue for locally attenuating inflammation in cardiac ischemic/reperfusion injury.

## Results

### IAMP is an evolutionarily conserved micropeptide enriched in CFs.

**Figure 1:**
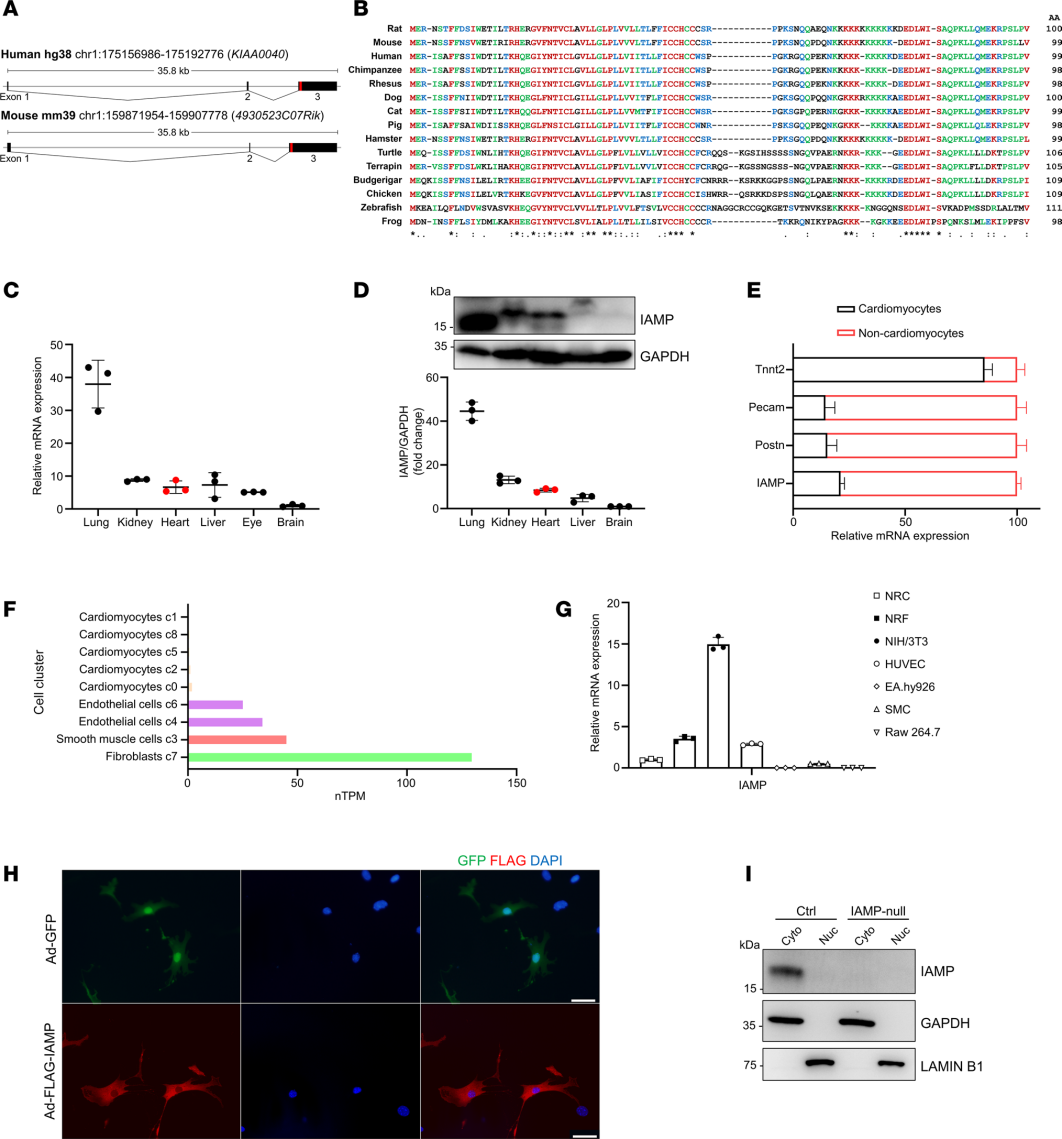
Micropeptide IAMP is highly expressed in cardiac fibroblasts. (**A**) Genomic locus of IAMP in human (*KIAA0040*) and mouse (*4930523C07Rik*). The ORF encoding IAMP is highlighted in red. (**B**) Multiple alignment of amino acid sequences of IAMP proteins across vertebrates. (**C**) qPCR analysis of IAMP mRNA expression in different tissues of adult mice. *n* = 3 for each group. (**D**) Western blotting of IAMP protein and quantification in different tissues of adult mice. *n* = 3 for each group. (**E**) qPCR analysis of IAMP mRNA expression in isolated adult cardiomyocytes and noncardiomyocytes. *n* = 4 for each group. (**F**) Analysis of IAMP expression in different cell clusters in the human heart using single-cell RNA-Seq data (https://www.proteinatlas.org/). (**G**) qPCR analysis of IAMP mRNA expression in different cell types. NRC, neonatal rat ventricular cardiomyocytes; NRF, neonatal rat ventricular fibroblasts; NIH-3T3, fibroblast cell line; HUVEC, human umbilical endothelial cells; EA.hy926, endothelial cell line; SMC, human smooth muscle cell; RAW 264.7, macrophage cell line. *n* = 3 for each group. (**H**) Immunofluorescence of cardiac fibroblasts infected by indicated adenoviruses. Scale bar: 50 μm. (**I**) Western blotting of IAMP protein in nuclear and cytoplasmic fractions in control and IAMP-null NIH-3T3 cells.

In a previous study, we identified multiple micropeptides associated with cardiac stress, most of which remain functionally uncharacterized (20). Many of these micropeptides lack evolutionary conservation. However, 1 micropeptide, comprising 99 amino acids, drew our attention due to its high conservation across species. We named this micropeptide IAMP based on its regulatory function uncovered in subsequent analyses. In humans, IAMP is encoded by the *KIAA0040* gene, while in mice, it is encoded by *4930523C07Rik*. Both genes are located on chromosome 1 of the human and mouse genomes (Figure 1A). The small open reading frame (sORF) encoding IAMP is situated within the third exon of both *KIAA0040* and *4930523C07Rik* (Figure 1A). Comparative analysis of DNA sequences from 100 vertebrates revealed high conservation of the IAMP sORF, as predicted by both the phyloP and phastCons algorithms (Supplemental Figure 1A; supplemental material available online with this article; https://doi.org/10.1172/jci.insight.187848DS1). Additionally, multiple sequence alignment of amino acid sequences across 15 selected vertebrates confirmed this conservation (Figure 1B). Notably, IAMP is predicted to possess 3 α-helices, as inferred by Alphafold (Supplemental Figure 1B). Analysis of transcriptomic data across multiple organs in both humans and mice showed moderate expression of IAMP in the heart (Supplemental Figure 1, C and D), a finding further validated through quantitative PCR (qPCR) analysis (Figure 1C) and Western blot analysis (Figure 1D).

Given that IAMP was identified as a micropeptide associated with cardiac stress, we further investigated its expression profile across different cell types within the heart. Initially, we isolated cardiomyocytes from noncardiomyocytes using Langendorff procedure and observed predominant expression of IAMP in noncardiomyocytes (Figure 1E). Subsequent analysis of a single-cell RNA-Seq dataset from the human heart corroborated this observation, revealing CFs as the cell type with the highest expression level of IAMP among various noncardiomyocytes (Figure 1F). To validate these findings, RNA was collected from various heart-related cell types, including neonatal rat cardiomyocytes (NRC), neonatal rat CFs (NRF), human umbilical vein endothelial cells (HUVEC), human aortic smooth muscle cells (SMC), NIH-3T3 fibroblasts, EA.hy926 endothelial cells, and RAW 264.7 macrophages. Consistent with previous analyses, NIH-3T3 and NRF exhibited the highest expression levels of IAMP among these cell types (Figure 1G).

Immunofluorescence staining indicated that ectopically expressed IAMP was primarily localized in the cytoplasm (Figure 1H). To detect endogenous IAMP, we generated a multiclonal IAMP antibody. Western blot analysis revealed successful detection of IAMP in WT NIH-3T3 cells using this antibody, while no signal was observed in IAMP-null NIH-3T3 cells, confirming the efficiency and specificity of the antibody (Figure 1I). Furthermore, cellular nucleus and cytoplasm fractionation followed by Western blotting confirmed the cytoplasmic localization of endogenous IAMP (Figure 1I). Together, our results demonstrate that IAMP, a micropeptide enriched in CFs, is evolutionarily conserved and predominantly expressed in the cytosol.

### IAMP suppresses inflammatory response in CFs.

**Figure 2:**
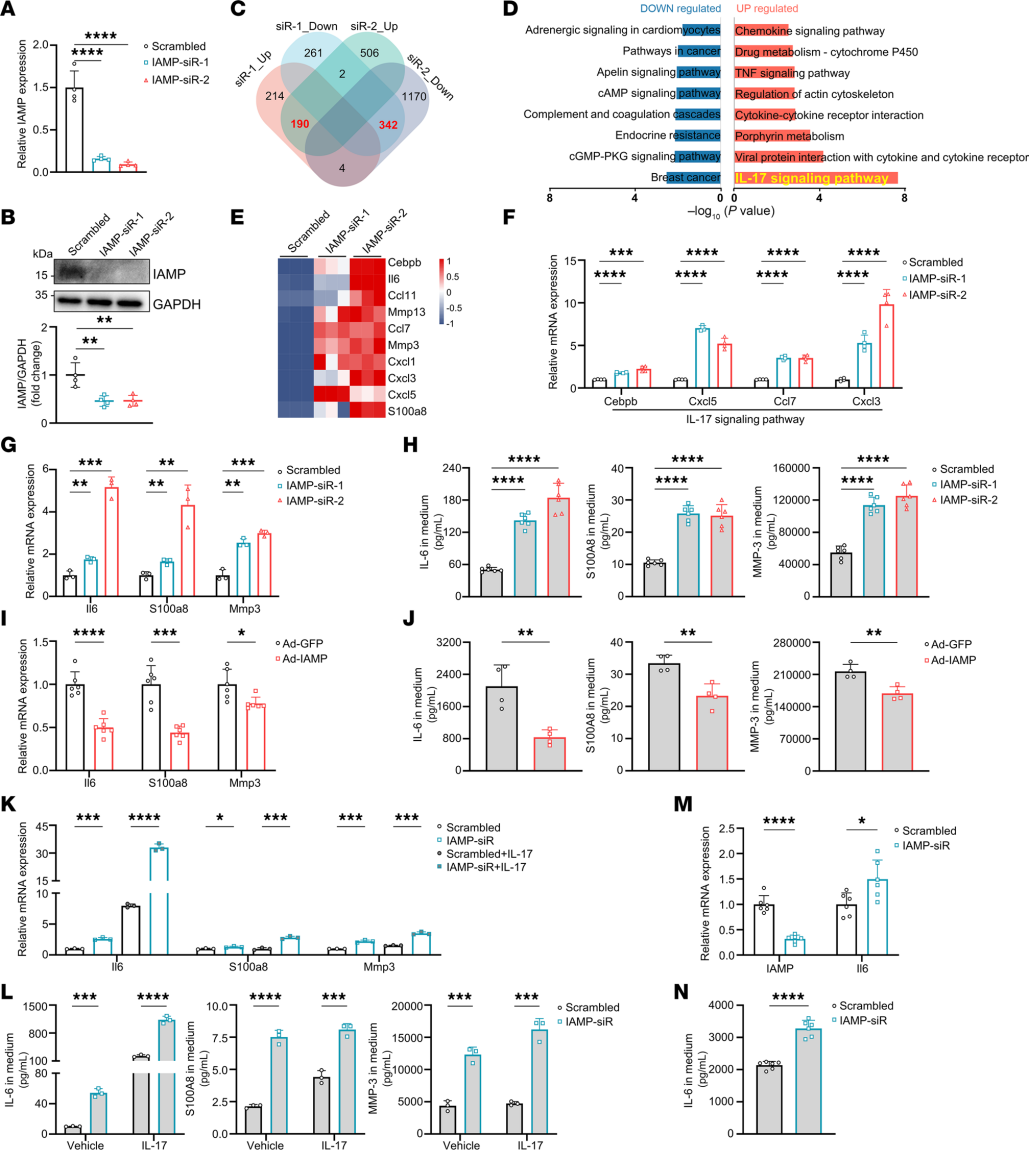
IAMP represses inflammatory response in cardiac fibroblasts. (**A** and **B**) qPCR and Western blot analysis of IAMP expression in IAMP-KD (knockdown) mouse cardiac fibroblasts. *n* = 4 for each group. (**C**) Venn diagram showing the number of differentially expressed genes in cardiac fibroblasts upon IAMP knockdown. (**D**) KEGG pathway enrichment analysis of dysregulated genes. (**E**) Heatmap showing relative expression of upregulated genes in IL-17 signaling pathway. (**F**) qPCR analysis of expression of genes in IL-17 signaling pathway in IAMP-KD mouse cardiac fibroblasts. *n* = 4 for each group. (**G**) qPCR analysis of mRNA levels of *Il6*, *S100a8*, and *Mmp3* in IAMP-knockdown (IAMP-KD) mouse cardiac fibroblasts. *n* = 3 for each group. (**H**) ELISA detecting protein levels of IL-6, S100A8, and MMP3 in culture medium of IAMP-KD mouse cardiac fibroblasts. *n* = 6 for each group. (**I**) qPCR analysis of mRNA levels of *Il6*, *S100a8*, and *Mmp3* in IAMP-overexpressing (IAMP-OE) mouse cardiac fibroblasts. *n* = 6 for each group. (**J**) ELISA detecting protein levels of IL-6, S100A8, and MMP3 in culture medium of IAMP-OE mouse cardiac fibroblasts. *n* = 4 for each group. (**K**) qPCR analysis of mRNA levels of *Il6*, *S100a8*, and *Mmp3* in IAMP-KD mouse cardiac fibroblasts with or without IL-17 stimulation. *n* = 3 for each group. (**L**) ELISA detecting protein levels of IL-6, S100A8, and MMP3 in culture medium of IAMP-KD mouse cardiac fibroblasts with or without IL-17 stimulation. *n* = 3 for each group. (**M**) qPCR analysis of mRNA levels of *IL6* in IAMP-KD human cardiac fibroblasts. *n* = 6 for each group. (**N**) ELISA detecting protein levels of IL-6 in culture medium of IAMP-KD human cardiac fibroblasts. *n* = 6 for each group. *P < 0.05; **P < 0.01; ***P < 0.001; ****P < 0.0001, by 2-tailed Student’s *t* test (**I**, **J**, **M**, and **N**) or ANOVA (1-way for **A**, **B**, **F**–**H** and 2-way for K and L) with Tukey’s correction (**A**, **B**, **F**–**H**, **K**, and **L**).

Subsequently, our focus shifted to elucidating the biological function of IAMP in CFs. We employed 2 independent siRNAs to knock down IAMP in primary mouse CFs isolated from adult mice (Figure 2, A and B). Total RNAs from both control and IAMP knockdown fibroblasts underwent RNA-Seq, revealing substantial alterations in the transcriptome upon IAMP knockdown (Supplemental Figure 2A and Supplemental Table 1). Cross-analysis of the transcriptomic changes induced by the 2 siRNAs unveiled 190 commonly upregulated genes and 342 commonly downregulated genes (Figure 2C; siRNA1: 408 genes upregulated, 605 genes downregulated; siRNA2: 698 genes upregulated, 1,516 genes downregulated). KEGG enrichment analysis highlighted the upregulation of inflammation-related pathways, such as the IL-17 and TNF signaling pathways and downregulation of pathways like cGMP/PKG signaling and cAMP signaling pathways upon IAMP knockdown (Figure 2D). Notably, the enrichment of the IL-17 signaling pathway, encompassing *Il6*, *S100a8*, *Mmp3*, and multiple genes for C-X-C motif chemokine ligands, was particularly marked (Figure 2, D and E). We validated these transcriptomic changes by assessing the expression levels of representative genes in these pathways using qPCR. (Figure 2F and Supplemental Figure 2B). It is worth noting that knockdown of IAMP did not substantially alter pathways related to fibroblast activation. Further investigation of IAMP on myofibroblast differentiation showed that knockdown of IAMP had a minimal effect on the differentiation of CFs under the stimulation of TGF-β (Supplemental Figure 3 and Supplemental Table 2). Together, these results suggest the involvement of IAMP primarily in inflammation rather than myofibroblast differentiation.

Previous studies have unveiled the emerging role of fibroblasts in triggering tissue inflammation and inflammation within the tumor microenvironment (13, 21–23). Given that loss of IAMP activates proinflammatory gene expression, we hypothesized that IAMP could regulate the inflammatory response in cardiac diseases mediated by CFs. Proinflammatory cytokines such as IL-6, S100A8, and MMP3 are known to be secreted by fibroblasts (23–26). Knockdown of IAMP in primary CFs led to substantial increases in the mRNA levels of *Il6*, *S100a8*, and *Mmp3* with independent siRNAs (Figure 2G). Consistently, the levels of secreted cytokines were elevated in the culture media of IAMP-knockdown fibroblasts as determined by ELISA (Figure 2H). Similarly, the expression and secretion of proinflammatory cytokines were activated in IAMP-null NIH-3T3 cells generated through CRISPR-Cas9 genome editing (Supplemental Figure 4, A–D). Conversely, overexpression of IAMP with adenovirus in primary mouse CFs suppressed the expression and secretion of IL-6, S100A8, and MMP3 (Figure 2, I and J). We then explored whether this regulation occurred in an inflammatory environment. Treatment of primary mouse CFs with proinflammatory cytokines such as IL-17, LPS, and TNF-α increased the expression of *Il6*, *S100a8*, and *Mmp3*, while further knockdown of IAMP intensified these inflammatory responses (Figure 2, K and L, and Supplemental Figure 4, E and F). To ascertain whether the function of IAMP is conserved in humans, we knocked down IAMP expression in primary human CFs. Consistently, loss of IAMP in primary human CFs activated the expression and secretion of the proinflammatory cytokine IL-6 (Figure 2, M and N). Collectively, these data suggest that IAMP functions as an antiinflammatory factor in CFs.

### IAMP expression correlates with myocardial inflammation in cardiac stress.

**Figure 3:**
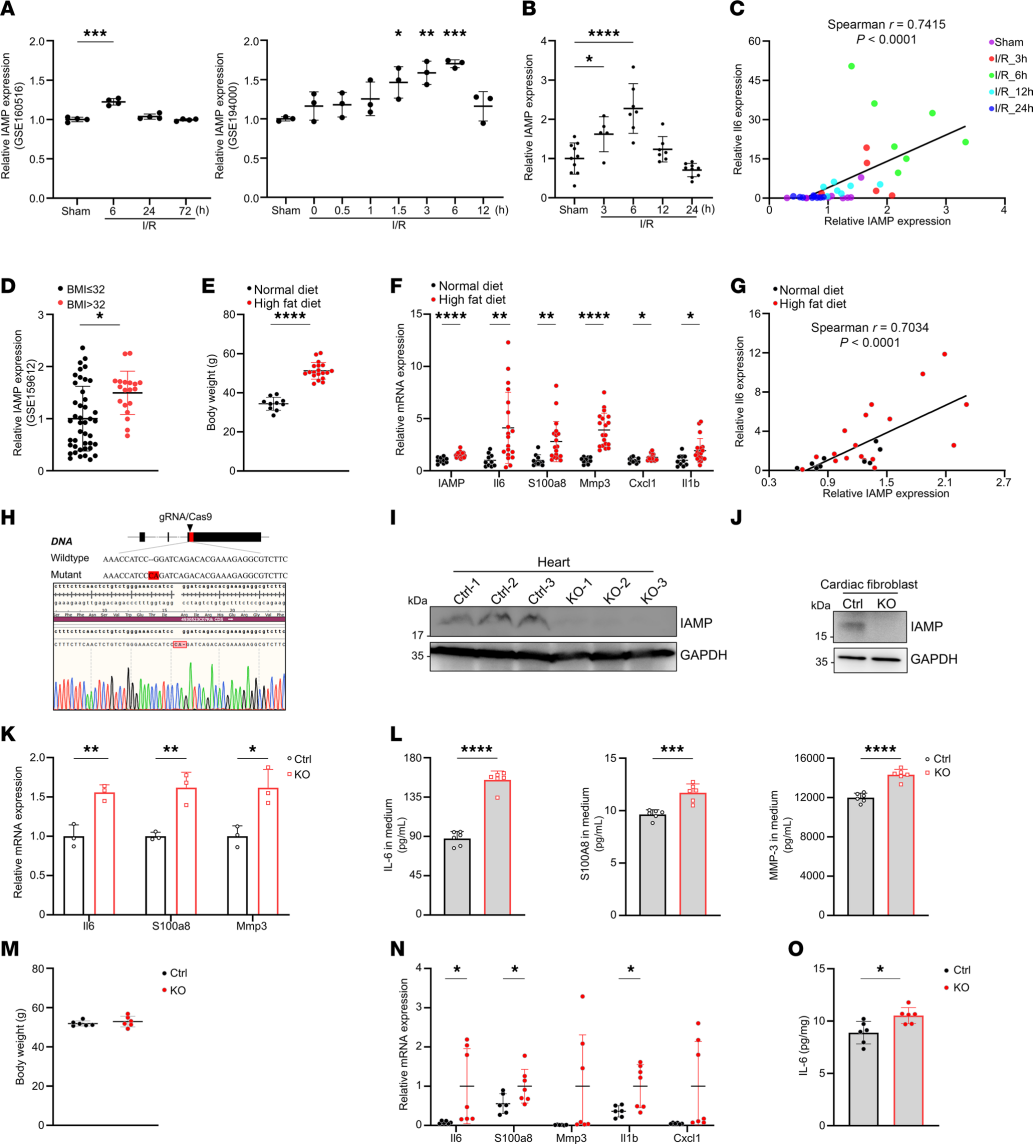
IAMP expression is correlated with myocardial inflammation in heart. (**A**) Relative expression of *IAMP* in hearts after IR injury from public RNA-Seq datasets. *n* = 3–4 for each group. (**B**) qPCR analysis of mRNA level of *IAMP* in mouse hearts after IR injury. *n* = 5–10 for each group. (**C**) Correlation between *IAMP* and *Il6* mRNA levels in hearts mentioned in **B**. (**D**) Relative expression of *IAMP* in patients with different BMI. BMI ≤ 32 (*n* = 43); BMI > 32 (*n* = 19). (**E** and **F**) Body weights and gene expression in hearts of 8-month-old mice fed with normal (*n* = 10) or high-fat (*n* = 19) diets for 5 months. (**G**) Correlation between *IAMP* and *Il6* expression in hearts mentioned in **F**. (**H**) Strategy of generating IAMP-KO mice by CRISPR/Cas9 editing and DNA sequence confirmation. (**I**) Western blotting of IAMP protein in hearts of control (Ctrl) and KO adult mice. (**J**) Western blotting of IAMP protein in cardiac fibroblasts isolated from Ctrl and KO adult mice. (**K**) qPCR analysis of mRNA levels of *Il6*, *S100a8*, and *Mmp3* mRNA levels in cardiac fibroblasts isolated from Ctrl and KO adult mouse. *n* = 3 for each group. (**L**) ELISA detecting protein levels of IL-6, S100A8, and MMP3 in culture medium of cardiac fibroblasts isolated from Ctrl and KO adult mouse. *n* = 6 for each group. (**M**–**O**) Body weights, gene expression in hearts, and IL-6 protein levels in hearts of Ctrl and IAMP-KO mice fed with high-fat diets for 5 months. *n* = 6–7 for each group. *P < 0.05; **P < 0.01; ***P < 0.001; ****P < 0.0001, by 2-tailed Student’s *t* test (**D**–**F** and **K–O**), ANOVA with Tukey’s correction (**A** and **B**), or Pearson correlation (**C** and **G**).

The inflammatory response plays a pivotal role in the pathophysiology of various cardiac diseases. To elucidate the relationship between IAMP and myocardial inflammation in cardiac stress, we initially examined the correlation of IAMP expression with cardiac ischemia/reperfusion (I/R) injury. Transcript expression of proinflammatory cytokine genes were detected predominantly in the noncardiomyocyte portion of the heart and dramatically increased in the early stage of I/R injury (Supplemental Figure 5). Analysis of 2 sets of RNA-Seq data reveal a transient increase in IAMP expression during I/R injury, peaking rapidly at 6 hours of reperfusion and returning to basal levels by 24 hours of reperfusion (Figure 3A). Additionally, the expression of IAMP exhibited a positive correlation with the expression levels of proinflammatory cytokine genes such as *Il6*, *Il1b*, *S100a8*, and *Cxcl1* (Supplemental Figure 6A), findings corroborated by qPCR (Figure 3, B and C, and Supplemental Figure 6B). Inflammation has been implicated as a crucial factor in cardiomyopathy associated with obesity. As anticipated, IAMP expression was substantially higher in the hearts of patients with obesity (Figure 3D). Furthermore, a positive correlation was observed between the expression levels of *IAMP*, *IL6*, and *S100A8* in cardiac transcriptomes from individuals with varying BMI (Supplemental Figure 6C). To validate these findings, we subjected mice to a high-fat diet (HFD) for 5 months and evaluated the expression of IAMP and other proinflammatory factors in the heart. In the HFD group, the expression levels of *IAMP*, *Il6*, *S100a8*, *Mmp3*, *Cxcl1*, and *Il1b* were all upregulated (Figure 3, E and F). Moreover, IAMP exhibited a positive correlation with the gene expression of these proinflammatory factors (Figure 3G and Supplemental Figure 6D).

To further investigate the function of IAMP, we generated an IAMP-KO mouse line using a CRISPR-Cas9 strategy to induce a frameshift mutation (Figure 3H and Supplemental Figure 7A). Western blot analysis confirmed the loss of the IAMP micropeptide in cardiac protein extracts from IAMP-KO mice (Figure 3I) without affecting its transcript levels (Supplemental Figure 7B). The KO of IAMP did not result in any discernible cardiac phenotype (Supplemental Figure 7C and Supplemental Table 3). Subsequently, we performed RNA-Seq using hearts from 8-week-old IAMP-KO and control mice under basal conditions. Transcriptome analysis revealed only a minimal number of differentially expressed genes (DEGs) (Supplemental Figure 7D), none of which were substantially enriched in any KEGG pathway. These results suggest that IAMP is dispensable for normal cardiac morphology and function. However, when we isolated and cultured primary CFs from IAMP-KO adult mice and their control littermates (Figure 3J), we observed increased expression and secretion of IL-6, S100A8, and MMP3 in IAMP-KO CFs (Figure 3, K and L), consistent with our previous findings. In addition, loss of IAMP promoted inflammation reaction in the myocardium of HFD-fed mice (Figure 3, M–O). Together, these data further underscore the potential regulatory role of IAMP in myocardial inflammation during cardiac stress.

### Loss of IAMP exacerbates I/R injury in the heart.

**Figure 4:**
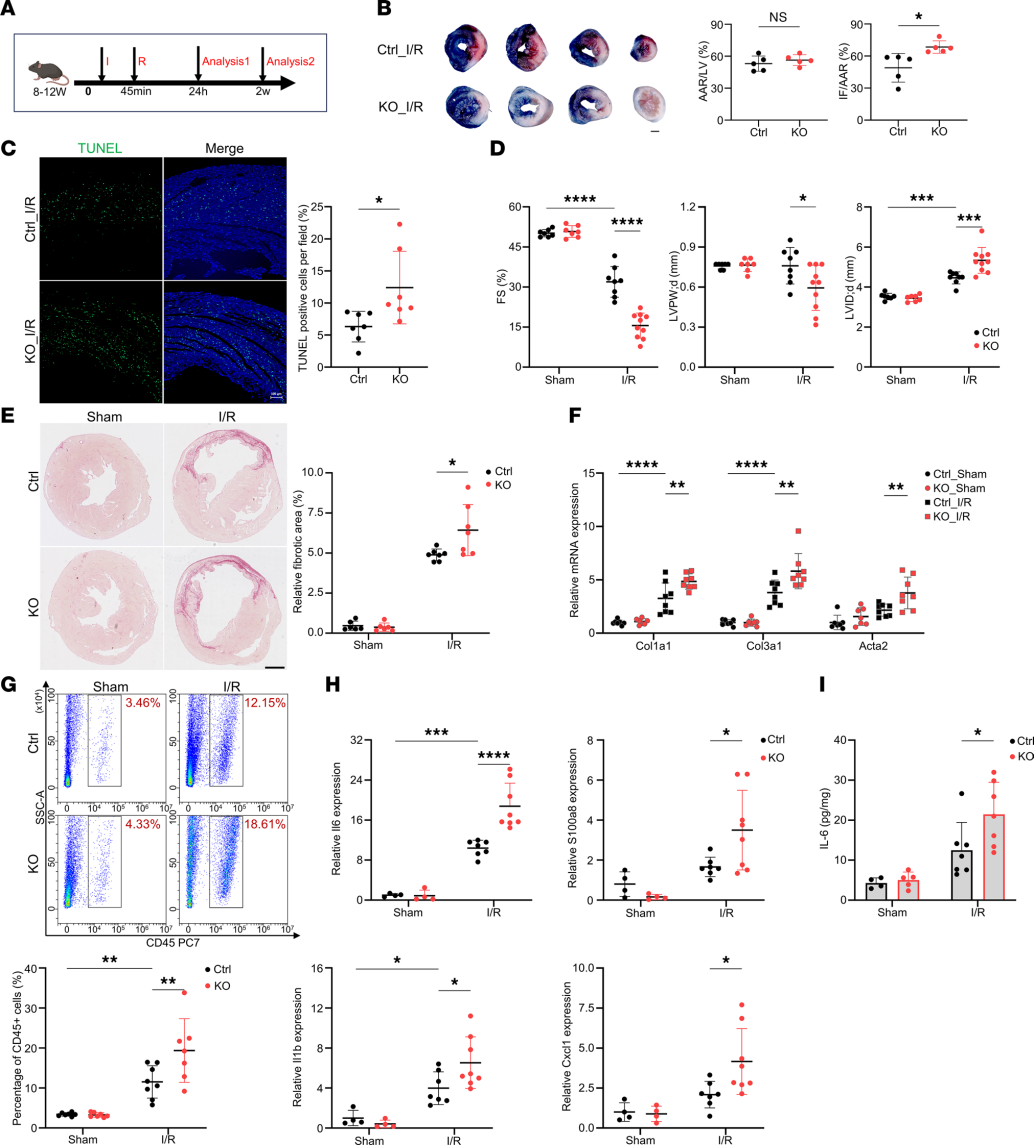
Loss of IAMP exacerbates ischemia/reperfusion injury in the heart. (**A**) Schedule for the in vivo study. (**B**) Evans blue and triphenyltetrazolium chloride (TTC) double staining and quantification of area at risk (AAR) and infarct size (IF) in hearts from Ctrl and IAMP-KO mice 24 hours after I/R surgery. *n* = 5 for each group. Scale bar: 1 mm. (**C**) TUNEL staining and quantification of apoptotic cells in cross sections of hearts from Ctrl and IAMP-KO mice 24 hours after I/R or surgery. *n* = 7 for each group. Scale bar: 100 μm. (**D**) Fractional shortening (FS), left ventricular posterior wall thickness at end-diastole (LVPW;d), and left ventricular internal dimension at end-diastole (LVID;d) accessed by echocardiography 2 weeks after I/R or sham surgery. *n* = 7–10 for each group. (**E**) Sirius red staining and quantifications of fibrotic area in cross sections of hearts from Ctrl and IAMP-KO mice 2 weeks after I/R or sham surgery. Scale bar: 1 mm. (**F**) qPCR analysis of mRNA levels of fibrosis marker genes. *n* = 7–8 for each group. (**G**) Flow cytometry and quantification of percentages of CD45^+^ cells in the ischemic area of hearts from Ctrl and IAMP-KO mice 24 hours after I/R or sham surgery. *n* = 7–8 for each group. (**H** and **I**) mRNA levels of *Il6*, *Il1b*, *Cxcl1*, and *S100a8* (**H**) and protein levels of IL-6 (**I**) in the ischemic area of hearts from Ctrl and IAMP-KO mice 24 hours after I/R or sham surgery. *n* = 4–8 for each group. *P < 0.05; **P < 0.01; ***P < 0.001; ****P < 0.0001, by 2-tailed Student’s *t* test (**B** and **C**) or ANOVA with Tukey’s correction (**D**–**I**).

To investigate the regulatory role of IAMP in myocardial inflammation, we subjected IAMP-KO mice and their control littermates to I/R injury (Figure 4A). While loss of IAMP did not alter the ischemic area, it substantially increased the infarct area 24 hours after surgery (Figure 4B). Apoptosis, detected by TUNEL staining, was also heightened in the hearts of IAMP-KO mice (Figure 4C and Supplemental Figure 8A). Two weeks after surgery, I/R injury resulted in a decrease in fractional shortening, a decrease in left ventricular (LV) posterior wall thickness, and an increase in internal diameter (Figure 4D and Supplemental Table 4). Importantly, loss of IAMP further exacerbated cardiac dysfunction and remodeling (Figure 4D). Furthermore, loss of IAMP led to enhanced cardiac fibrosis, as evidenced by an increase in the fibrotic area (Figure 4E), as well as elevated expression of fibrosis markers *Col1a1*, *Col3a1*, and *Acta2* (Figure 4F). Moreover, the expression of remodeling markers *Nppb*, *Nppa*, and *Acta1* was also increased (Supplemental Figure 8B).

Given the previous findings suggesting an antiinflammatory role of IAMP, we further evaluated the inflammatory response in IAMP-KO hearts. Noncardiomyocyte cells isolated from the ischemic myocardium were subjected to flow cytometry analysis. While I/R injury increased the percentages of CD45^+^ immune cells to approximately 10%, IAMP-KO further elevated this percentage to about 20% (Figure 4G). Additionally, mRNA levels of inflammation markers *Il6*, *Il1b*, *Cxcl1*, and *S100a8* were all increased in the ischemic myocardium of IAMP-KO hearts (Figure 4H). Consistently, ELISA confirmed a higher protein level of proinflammatory cytokine IL-6 in ischemic IAMP-KO myocardium (Figure 4I). Taken together, these results indicate that loss of IAMP promotes inflammation in the ischemic myocardium and, therefore, further exacerbates I/R injury by inducing more severe cardiac remodeling.

### IAMP regulates proinflammatory gene expression through downregulating HIF-1α.

**Figure 5:**
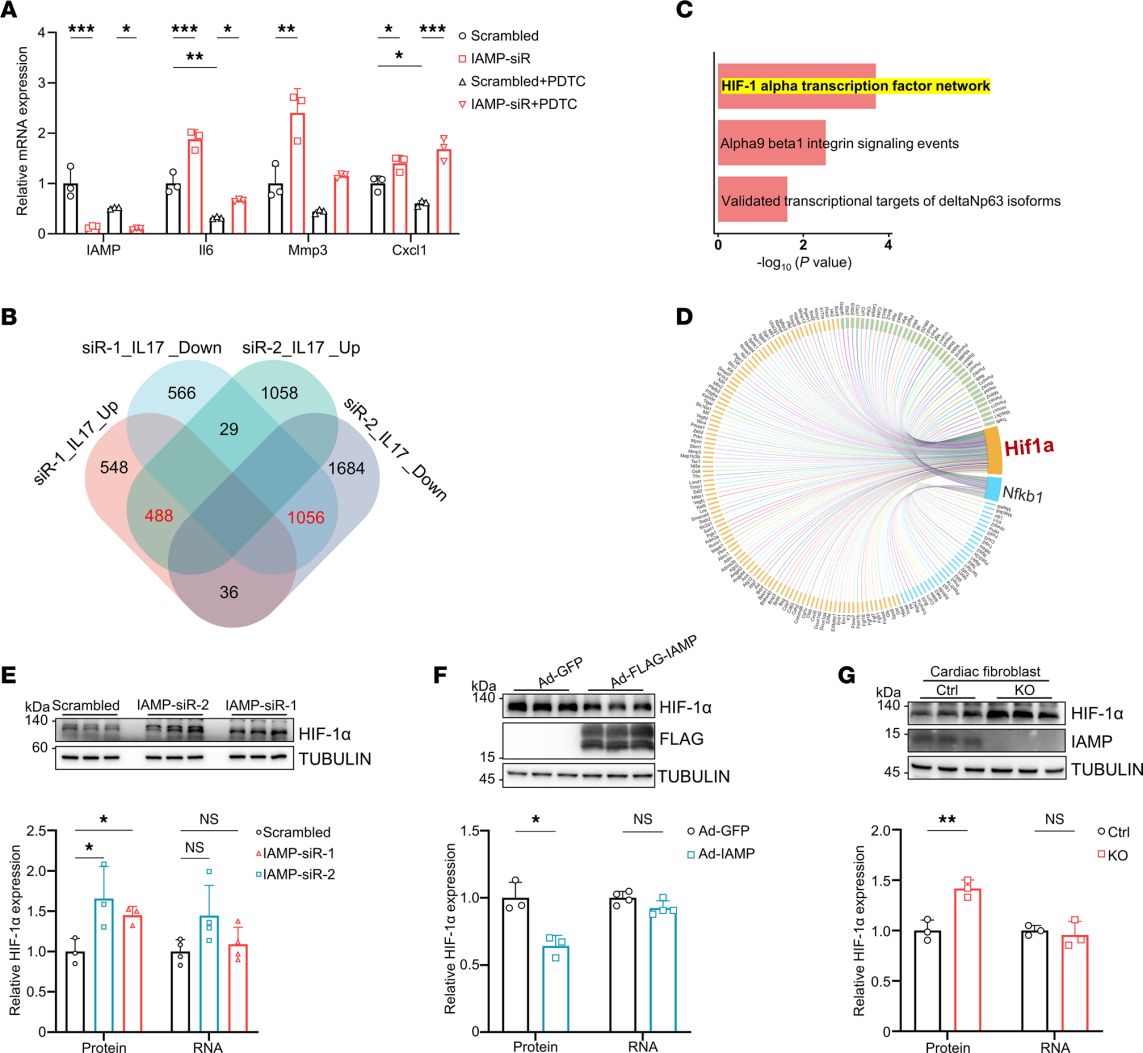
IAMP regulates HIF-1α protein expression. (**A**) qPCR analysis of mRNA levels of *IAMP*, *Il6*, *Mmp3*, and *Cxcl1* in control and IAMP-KD cardiac fibroblasts with or without PDTC (10 �M) treatment. *n* = 3 for each group. (**B**) Venn diagram showing the numbers of differentially expressed gene in IL-17–stimulated IAMP-KD cardiac fibroblasts. (**C**) Pathway enrichment analysis of the upregulated genes using the NCI pathway interaction database. (**D**) STRING interaction network of HIF-1α and NF-κB with proteins of upregulated genes. The yellow and blue boxes represent HIF-1α– and NF-κB–interacting proteins, respectively. (**E**) Western blotting and quantification of HIF-1α protein (*n* = 3) and qPCR analysis of *Hif1a* mRNA (*n* = 4) in the IAMP-KD cardiac fibroblasts. (**F**) Western blotting and quantification of HIF-1α protein (*n* = 3) and qPCR analysis of *Hif1a* mRNA (*n* = 4) in the IAMP-OE cardiac fibroblasts. (**G**) Western blotting and quantification of HIF-1α protein (*n* = 3) and qPCR analysis of *Hif1a* mRNA (*n* = 3) in the cardiac fibroblasts isolated from IAMP-KO and Ctrl mice. *P < 0.05; **P < 0.01; ***P < 0.001, by 2-tailed Student’s *t* test (**F** and **G**) or ANOVA with Tukey’s correction (**A** and **E**).

We sought to elucidate the mechanism by which IAMP regulates the inflammatory response in CFs. NF-κB signaling is known to be pivotal in inflammation, with the phosphorylation of IKBA and p65 representing key steps in the activation of this proinflammatory pathway (27, 28). Surprisingly, under baseline conditions, both phospho-IKBA and phospho-p65 levels failed to be induced by IAMP knockdown (Supplemental Figure 9A). Moreover, in CFs stimulated by IL-17, TNF-α, or LPS, IAMP knockdown did not lead to increased NF-κB signaling–related gene expression nor increased phosphorylation of IKBA or p65 (Supplemental Figure 9, B and C). Furthermore, NF-κB inhibitor Pyrrolidinedithiocarbamate ammonium (PDTC) failed to abolish the upregulation of proinflammatory cytokine genes induced by IAMP knockdown (Figure 5A). Therefore, these data suggest that NF-κB signaling is unlikely to mediate the antiinflammatory role of IAMP.

To explore potential signaling pathways mediating the expression changes of proinflammatory cytokine genes, pathway enrichment analyses were conducted to identify dysregulated signaling pathways in RNA-Seq data from IAMP-knockdown CFs treated with IL-17 (Figure 5B and Supplemental Table 5). The HIF-1α signaling pathway was enriched in both the NCI-Nature Pathway Interaction Database and the Molecular Signatures Database (MSigDB) among the upregulated genes in IAMP-knockdown CFs under IL-17 treatment (Figure 5C and Supplemental Figure 9D). Subsequently, we subjected the upregulated genes to protein-protein interaction (PPI) analysis and identified proteins of 146 genes that might interact with HIF-1α, whereas only proteins of 70 genes were predicted to interact with NF-κB (Figure 5D). This analysis suggests that the HIF-1α–related network is more likely to be involved in the antiinflammatory effect of IAMP. Indeed, knockdown of IAMP in CFs resulted in an increase in HIF-1α protein levels but not its transcript expression (Figure 5E). Conversely, adenovirus-mediated overexpression of IAMP in CFs led to a reduction in HIF-1α protein levels but not its transcript expression (Figure 5F). Furthermore, protein levels of HIF-1α were substantially elevated in IAMP-KO CFs with unchanged transcript levels (Figure 5G).

**Figure 6:**
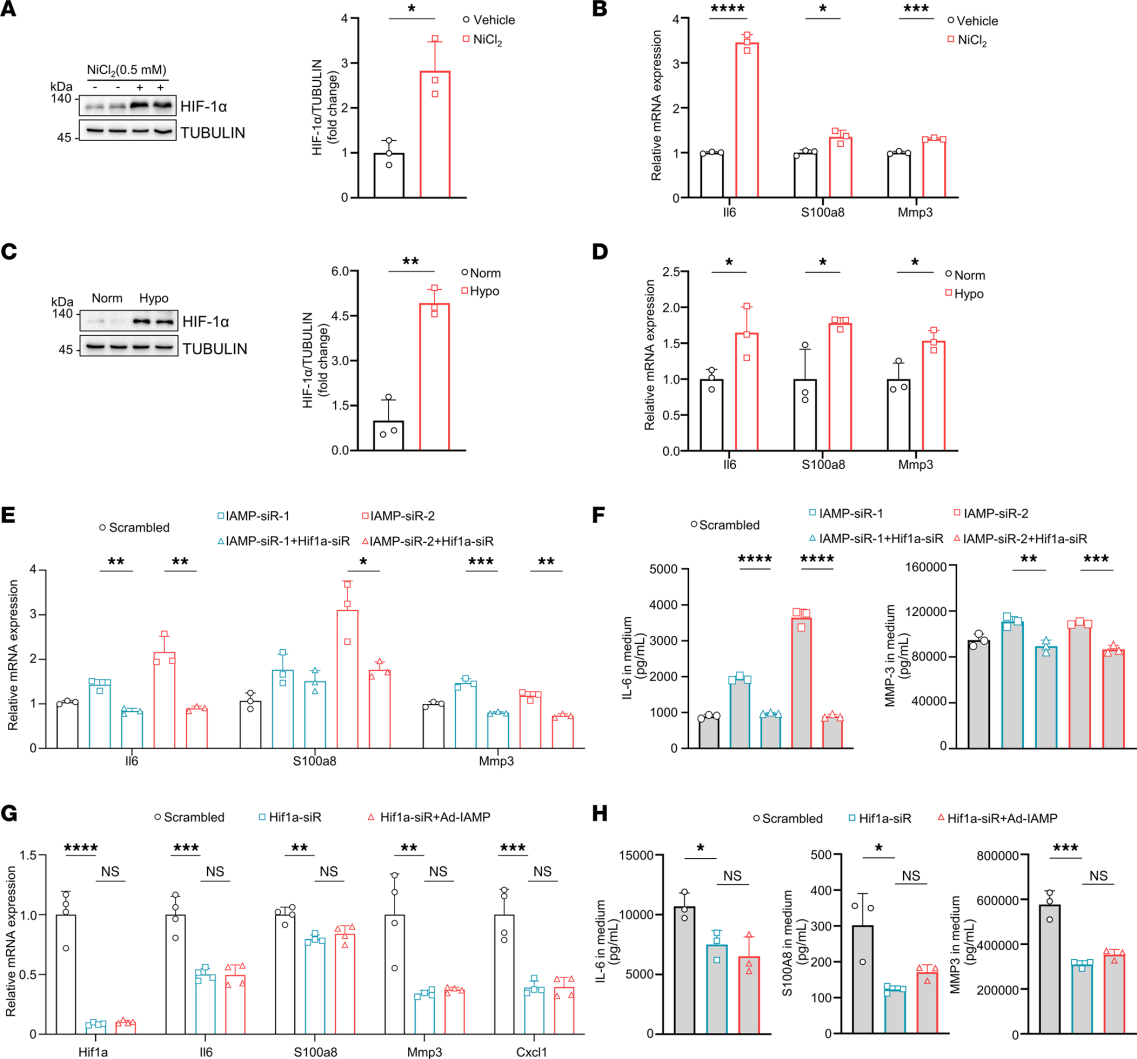
IAMP regulates proinflammatory gene expression through HIF-1α. (**A**) Western blotting and quantification of HIF-1α protein in cardiac fibroblasts with or without NiCl_2_ treatment. *n* = 3 for each group. (**B**) qPCR analysis of mRNA levels of *Il6*, *S100a8*, and *Mmp3* in cardiac fibroblasts with or without NiCl_2_ treatment. *n* = 3 for each group. (**C**) Western blotting and quantification of HIF-1α protein in cardiac fibroblasts with or without hypoxia treatment. *n* = 3 for each group. (**D**) qPCR analysis of mRNA levels of *Il6*, *S100a8*, and *Mmp3* in cardiac fibroblasts with or without hypoxia treatment. *n* = 3 for each group. (**E**) qPCR analysis of mRNA levels of *Il6*, *S100a8*, and *Mmp3* in cardiac fibroblasts transfected with indicated siRNAs. *n* = 3 for each group. (**F**) ELISA detecting protein levels of IL-6 and MMP3 in culture medium of cardiac fibroblasts transfected with indicated siRNAs. *n* = 3 for each group. (**G**) qPCR analysis of mRNA levels of *Hif1a*, *Il6*, *S100a8*, *Mmp3*, and *Cxcl1* in cardiac fibroblasts treated with indicated siRNA and adenoviruses. *n* = 4 for each group. (**H**) ELISA detecting protein levels of IL-6, S100A8, and MMP-3 in culture medium of cardiac fibroblasts treated with indicated siRNA and adenoviruses. *n* = 3 for each group. *P < 0.05; **P < 0.01; ***P < 0.001; ****P < 0.0001, by 2-tailed Student’s *t* test (**A**–**D**) or ANOVA with Tukey’s correction (**E**–**H**).

HIF-1α has been implicated in promoting inflammation (29, 30), yet its involvement in the inflammatory response within CFs remains uncertain. Therefore, we induced intracellular accumulation of HIF-1α through NiCl_2_ treatment and hypoxia and assessed alterations in the inflammatory response. As expected, both approaches increased protein levels of HIF-1α in CFs (Figure 6, A–D). Consequently, expression of inflammatory markers, such as *Il6*, *S100a8*, and *Mmp3*, were substantially elevated under both NiCl_2_ treatment and hypoxia (Figure 6, A–D). To further determine whether the antiinflammatory effects of IAMP were dependent on HIF-1α, we examined the effect of IAMP knockdown on the expression and secretion of proinflammatory factors. Consistent with previous findings, knockdown of IAMP heightened the expression and secretion of proinflammatory factors (Figure 6, E and F), while knockdown of HIF-1α abolished this effect (Figure 6, E and F, and Supplemental Figure 10A). Furthermore, the regulation of proinflammatory gene expression by IAMP was abolished once the expression of HIF-1α was forcedly modulated in CFs (Figure 6, G and H, and Supplemental Figure 10B). Collectively, these results indicate that HIF-1α mediates the regulatory role of IAMP in inflammation in CFs.

### IAMP destabilizes HIF-1α proteins through interacting with its stabilizing partner HSP90.

**Figure 7:**
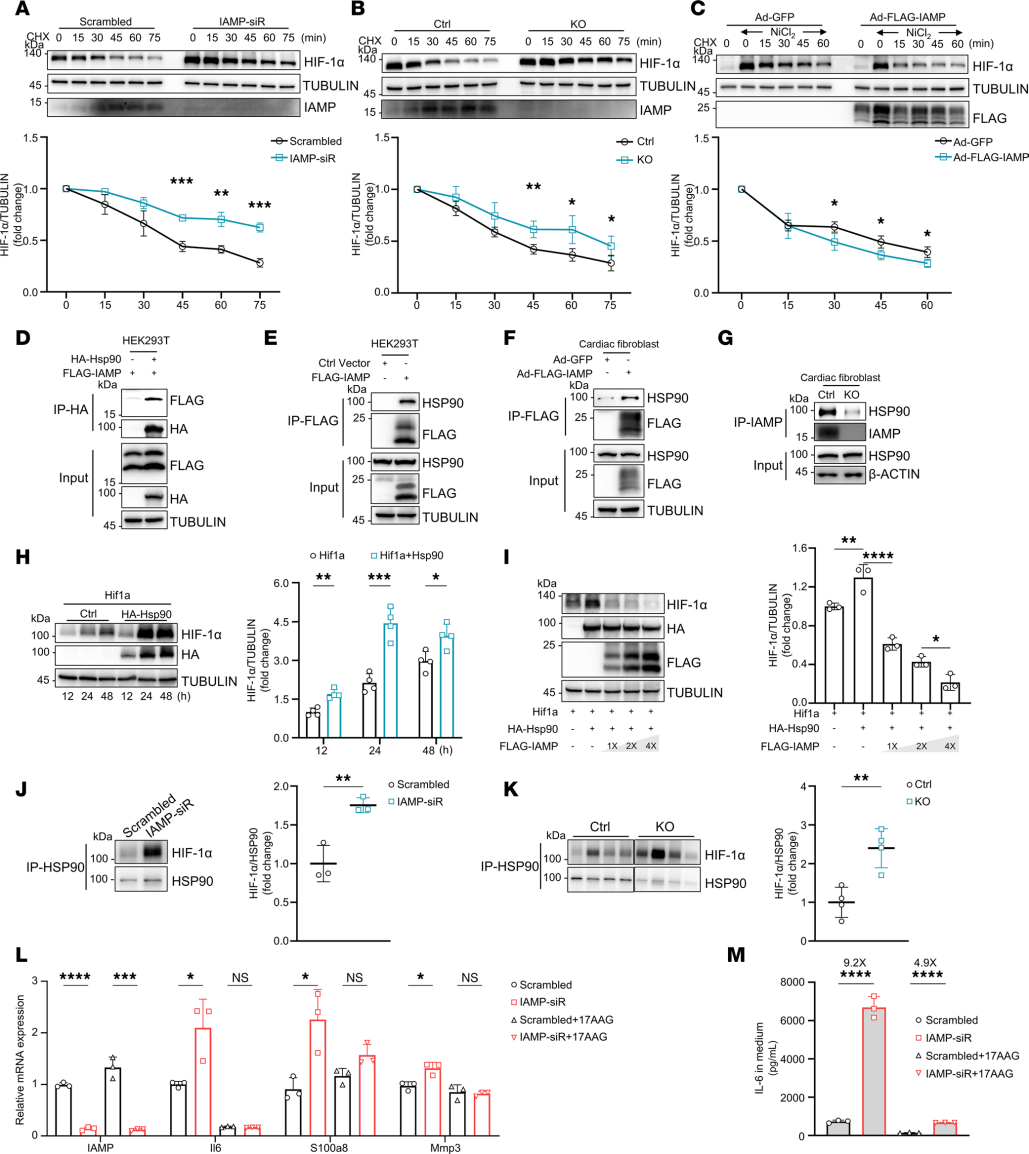
IAMP destabilizes HIF-1α proteins through interacting with HSP90. (**A**–**C**) HIF-1α stability in cardiac fibroblasts transfected with IAMP siRNAs (**A**), isolated from IAMP-KO and Ctrl mice (**B**), or infected by Ad-FLAG-IAMP (**C**) were exposed to NiCl_2_ (50 mM) for 24 hours. Cells were cultured with cycloheximide (CHX) (50 μg/mL) with indicated time before harvest. Western blotting and quantification of HIF-1α were shown. *n* = 3–4 for each group. (**D** and **E**) Coimmunoprecipitation and Western blotting showing the interaction of exogenous HA-HSP90 and FLAG-IAMP (**D**) and endogenous HSP90 and exogenous FLAG-IAMP in HEK293 cells (**E**). (**F**) Coimmunoprecipitation and Western blotting showing the interaction of endogenous HSP90 and exogenous FLAG-IAMP in cardiac fibroblasts. (**G**) Coimmunoprecipitation and Western blotting showing the interaction of endogenous HSP90 and IAMP in cardiac fibroblasts isolated from IAMP-KO or Ctrl mice. (**H**) Western blotting and quantification of HIF-1α protein in HEK293 transfected with indicated plasmids for 12, 24, and 48 hours. *n* = 4 for each group. (**I**) Western blotting and quantification of HIF-1α protein in HEK293 transfected with indicated plasmids. *n* = 3 for each group. (**J** and **K**) Pull-down experiments with anti-HSP90 antibodies followed by Western blotting detecting the interaction of HSP90 and HIF-1α in control and IAMP-KD cardiac fibroblasts (*n* = 3 for each group) (**J**), and in myocardium from Ctrl and IAMP-KO mice 6 hours after I/R surgery (*n* = 4 for each group) (**K**). (**L** and **M**) Gene expression of *Il6*, *S100a8*, and *Mmp3* in cardiac fibroblasts (**L**) and IL-6 protein (**M**) in the culture medium of cardiac fibroblasts transfected with IAMP or control siRNAs with or without treatment with 17-AAG. *n* = 3 for each group. *P < 0.05; **P < 0.01; ***P < 0.001; ****P < 0.0001, by 2-tailed Student’s *t* test (**A**–**C**, **H**, **J**, and **K**) or ANOVA with Tukey’s correction (**I**, **L**, and **M**).

Our results suggest that IAMP regulates the expression of HIF-1α in a transcription-independent manner. Since protein stability is one of the main factors for HIF-1α protein expression (31), we subsequently examined the effect of IAMP on the protein stability of HIF-1α through a cycloheximide (CHX) chase assay. CFs were treated with NiCl_2_ to enhance the expression of HIF-1α, followed by the addition of CHX to halt protein translation. Serial protein samples were collected every 15 minutes to assess the degradation rate. Loss of IAMP through either siRNA knockdown or genetic KO substantially attenuated the degradation of HIF-1α (Figure 7, A and B). Conversely, overexpression of IAMP accelerated the rate of HIF-1α degradation (Figure 7C). Collectively, these findings indicate that IAMP promotes the degradation of HIF-1α.

We then asked how IAMP regulated the stability of HIF-1α protein. Given that IAMP promotes the degradation of HIF-1α, we hypothesize that IAMP inhibits the binding of HIF-1α to its partner for protein stabilization. HSP90 is a well-established protein partner known to prevent HIF-1α degradation (32, 33). To test our hypothesis, we first explored the potential interaction between IAMP and HSP90. The interaction between IAMP and HSP90 was predicted by PDBePISA (Supplemental Figure 10C). Indeed, FLAG-tagged IAMP interacted with both ectopic-expressed HA-tagged and endogenous HSP90 in HEK293 cells in a coimmunoprecipitation (Co-IP) assay (Figure 7, D and E). In CFs, overexpression of FLAG-tagged IAMP also led to the coprecipitation of HSP90 with anti-FLAG antibodies (Figure 7F). To reinforce this discovery, we performed endogenous immunoprecipitation in CFs from IAMP-KO and control mice using an IAMP antibody. HSP90 was found to be enriched in the immunoprecipitation of control fibroblasts compared with IAMP-KO fibroblasts (Figure 7G), thus confirming the interaction between IAMP and HSP90. Next, we investigated whether IAMP regulates the protein stability of HIF-1α through its interaction with HSP90. Consistent with previous reports (32, 33), overexpression of HSP90 increased the ectopic protein expression of HIF-1α in CFs (Figure 7, H and I). However, co-overexpression of IAMP counteracted this effect in a dose-dependent manner (Figure 7I). Moreover, loss of IAMP promoted the PPI between HIF-1α and HSP90 both in CFs, and myocardium underwent I/R (Figure 7, J and K). Notably, treatment with the HSP90 inhibitor 17-AAG attenuated the proinflammatory effect of IAMP knockdown in CFs, as evidenced by the mRNA levels of *Il6*, *S100a8*, and *Mmp3* and the secretion of cytokine IL-6 (Figure 7, L and M). In summary, these findings indicate that IAMP promotes the degradation of HIF-1α by interacting with its stabilizing partner HSP90, thereby suppressing the HIF-1α–mediated inflammatory response in CFs.

## Discussion

**Figure 8:**
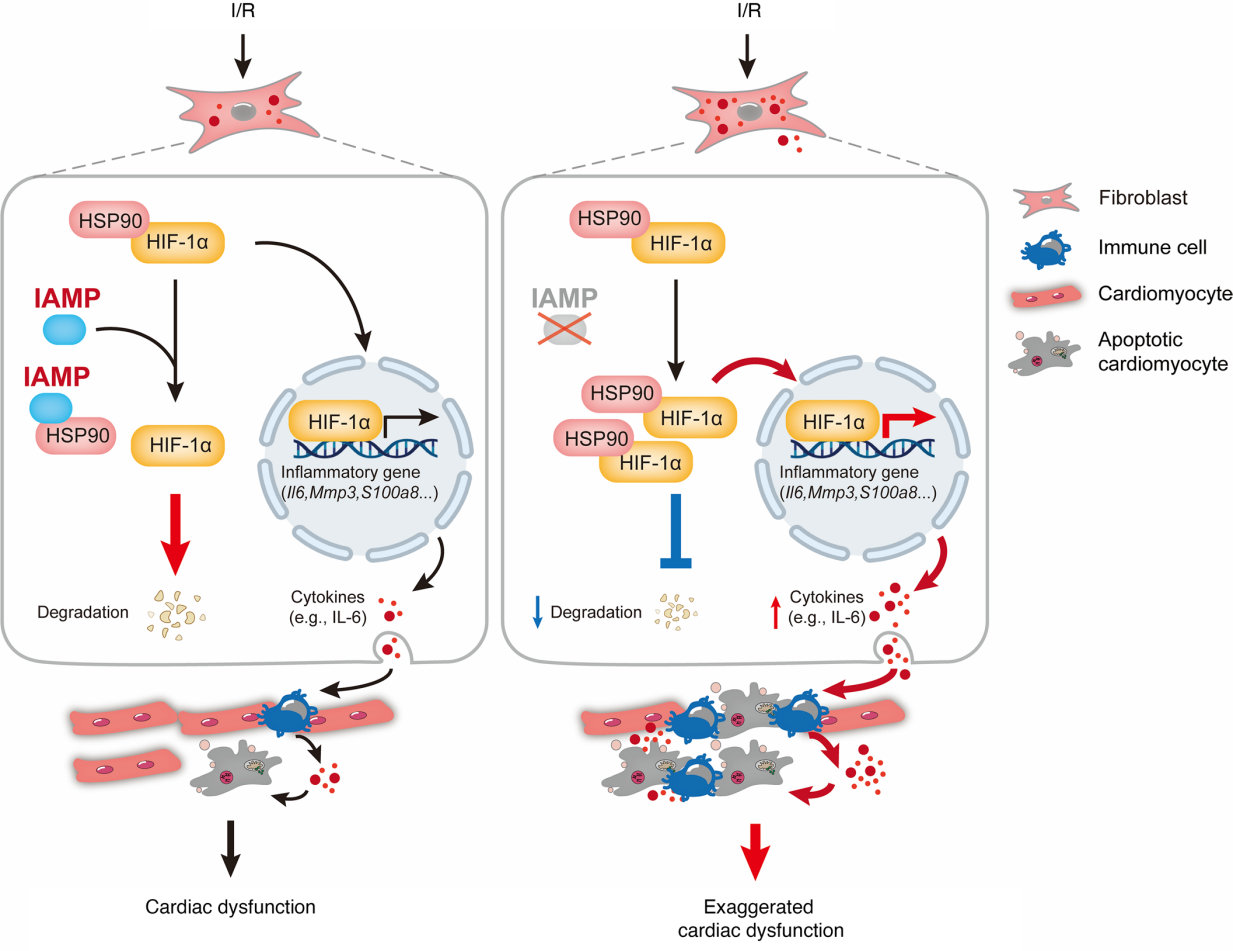
A proposed working model for the regulation of inflammation in cardiac fibroblasts and cardiac ischemia/reperfusion injury by IAMP

Here, we report the function of a previously uncharacterized micropeptide, IAMP, in myocardial I/R injury and inflammation mediated by CFs. We found that IAMP suppresses the inflammatory response of CFs. Consequently, the loss of IAMP induces uncontrolled myocardial inflammation during I/R injury. We further demonstrated that HIF-1α is the major downstream effector of IAMP in this condition. By interacting with its stabilizing factor HSP90, IAMP suppresses the stabilization of HIF-1α under ischemic stress and its function as a transcription factor for proinflammatory genes. Genetic deletion of IAMP increases the stability of HIF-1α, promotes the transcription of proinflammatory genes, and thereby exacerbates myocardial I/R injury (Figure 8). These findings support the biological significance of micropeptides in cardiac diseases.

Given that the likelihood an ORF encodes a protein increases with its length, ORFs shorter than 300 nucleotides were previously annotated as noncoding. However, recent advancements in proteomic and translatomic techniques prove otherwise (34). A portion of long noncoding RNAs (lncRNAs) have been found to encode proteins shorter than 100 amino acids, known as micropeptides. Multiple micropeptides have been identified as important regulators in the heart. For example, a micropeptide named DWarf Open Reading Frame (DWORF) in cardiomyocytes interacts with SR-Ca^2+^-ATPase (SERCA), enhancing its activity and improving cardiac contractility in dilated cardiomyopathy (35). The micropeptide humanin, encoded by mitochondrial DNA, is involved in reactive oxygen species (ROS) accumulation and cardiomyocyte hypertrophy induced by endonuclease G deficiency (36). The micropeptide regulator of β-oxidation (MOXI) enhances fatty acid β-oxidation in cardiomyocytes (37). Despite these advances, much remains to be explored, as it is estimated that around one-fourth of lncRNAs in the heart encode micropeptides (16). Furthermore, most studies on cardiac micropeptides focus on cardiomyocytes, and the role of micropeptides in CFs remains poorly understood. IAMP is detectable at relatively low levels in neonatal cardiomyocytes (Figure 1G, compared with neonatal fibroblasts), which is consistent with our previous data regarding the identification of IAMP in libraries form NRC (20), and its expression is substantially enriched in CFs during adulthood. In the present study, we discovered that the fibroblast-enriched micropeptide IAMP suppresses the inflammatory response of CFs and regulates myocardial I/R injury. These findings shed light on the pathological role of micropeptides in CFs. However, our current study cannot exclude the possibility that IAMP may also play a regulatory role in other cell types during I/R injury. A CF-specific IAMP-KO mouse model should be employed to overcome this limitation and further elucidate the in vivo function of IAMP in the future.

Inflammation is a crucial pathophysiological process in myocardial I/R injury. While the inflammatory response contributes to cardiac repair after injury, an excessive early burst of inflammation following MI is detrimental in terms of ventricular remodeling (38). Some small-scale trials testing the efficacy of suppressing early inflammation through IL blockade have shown promising results (39, 40), but these treatments invariably increase the risk of infection (4). IAMP might serve as a potential therapeutic target with the following advantages: (a) IAMP appears to be a specific regulator of the early burst of inflammation due to its transient increase early in I/R, and (b) IAMP may have minimal effect on systemic defense as it suppresses the inflammatory response mediated by fibroblasts rather than classical immune cells.

The proinflammatory role of fibroblasts has been studied in various pathological processes, including cardiac diseases (41). Some researchers propose that fibroblasts differentiate into heterogeneous subpopulations with diverse functions in diseases, one of which has a proinflammatory phenotype (13). However, the regulatory network driving the differentiation of specialized fibroblasts is not fully understood. The recent discovery of micropeptides encoded by lncRNAs adds another layer of complexity to this regulatory network. Our study identified IAMP, which is highly expressed in CFs. Loss of IAMP induces the transformation of CFs into a more proinflammatory phenotype and exacerbates cardiac ischemic injury. The findings of this study further support the concept that, despite their small molecular weight, micropeptides play an important role in pathological processes.

Under hypoxic conditions, HIF-1α is stabilized at least partially by HSP90 and accumulates in the nucleus, where it acts as a transcription factor (42). HIF-1α binds to hypoxia-response elements and promotes the transcription of a subset of genes, including antioxidants and proinflammatory factors (42). HIF-1α has heterogeneous effects on MI. While cardiomyocyte-specific HIF-1α overexpression preserves cardiac function 4 weeks after MI (43), HIF-1α downregulation in cardiomyocytes also appears to have a short-term cardioprotective effect after MI, noted by reduced apoptosis and cardiac rupture (44). The dual effect of HIF-1α in antioxidation and inflammation could account for these conflicting results. In cancer, hypoxia induces an inflammatory fibroblast phenotype in a HIF-1α–dependent manner (45). More recently, HIF-1α has been shown to suppress the proliferation of CFs following myocardial infraction (46). Consistent with these findings, our data indicate that knockdown of IAMP, which increases HIF-1α protein levels, leads to a decrease in the proliferation of CFs during differentiation induced by TGF-β. Given that IAMP is transiently upregulated during the early stages of I/R injury, a critical time window for the inflammatory response, and that loss of IAMP primarily affects the expression of proinflammatory genes in CFs (under both IL-17 and TGF-β treatments), we focused on the regulatory role of HIF-1α in proinflammatory gene expression in this study. Here, we found that HIF-1α is a critical mediator of the antiinflammatory effect of IAMP in CFs. These data provide more evidence on the diverse functions of HIF-1α in ischemic cardiomyopathy. Future studies should investigate how HIF-1α’s roles in regulating proinflammatory gene expression and fibroblast proliferation are coordinated during cardiac fibrosis formation, especially in different cardiac injury models, such as I/R versus MI.

## Methods

### Sex as a biological variable.

Our study examined male and female animals, and similar findings are reported for both sexes.

### Animals.

Anesthesia of mice was performed with a nose cone delivering 2%–4% isoflurane in oxygen via small animal ventilator. The setting of ventilator was 110–120 breaths per minute with a tidal volume of 0.1 mL under constant monitoring of the inspiratory pressure. At the end of the experiments, mice were killed with the i.p. injection of an overdose of sodium pentobarbital (200 mg/kg).

### Generation of IAMP-KO mice.

IAMP-KO mice were generated by Biocytogen. In brief, mRNA of Cas9 and 1 sgRNA (guide sequence: 5′-TCTTTCGTGTCTGATCCGGATGG-3′) were injected into single-cell fertilized eggs of C57BL/6 mice. The injected fertilized eggs were transplanted into surrogate female mice. F0 generation mice were obtained after 21 days of pregnancy. Genotyping was performed using DNA extracted from tail tips by Sanger sequencing. F0 mice with a positive genotype were mated with WT mice to produce the F1 generation. IAMP-HET mice were crossed to generate IAMP-KO mice. IAMP-HET and WT littermates were used as controls for IAMP-KO mice. The primers for genotyping were: Common_forward: 5′ GCGCGTCTAACACTGTGTTCTGTGACACCG-3′; WT_reverse: 5′-GATACGCTGCTTTAATGCCTTTAAAGACGCCTCTTTCGTGTCTGATCC-3′; and KO_reverse: 5′-CGGCCAAGACGCCTCTTTCGTGTCTGATCT-3′.

### Myocardial IR.

Eight-week-old mice were anesthetized with 2%–4% isoflurane and placed supine on a heated operating table for I/R surgery. A small left parasternal incision was made between intercostal spaces VI and V. After retracting the chest wall, mice were rotated into the right lateral position to expose the left ventricle. The left anterior descending (LAD) artery was visualized, and an 8–0 suture was passed around this coronary artery just under the tip of the left auricle. Polyethylene tubing was placed under the suture on top of the vessel to occlude the coronary artery. Body temperature was maintained at 37°C using a heating pad. After 45 minutes of ischemia, the slipknot was released, and the heart was reperfused for 24 hours based on the assays. Mice were maintained at thermoneutrality on the day of the procedure. Sham-operated control mice underwent the same surgical procedure, except the suture placed under the left coronary artery was not tied.

### Measurement of myocardial infarct size.

Combined Evans blue and triphenyltetrazolium chloride (TTC) staining was performed to determine the early cardioprotective effects of IAMP. Mice were anesthetized and ventilated. After 45 minutes of ischemia and 24 hours of reperfusion, the suture thread around the LAD artery was retied, and Evans blue dye (2% in PBS) was injected i.v. into the mice. After 3 minutes, the heart was quickly excised and incubated for 10 minutes at –80°C. The heart was cut into 4 slices (~2 mm thick each) and incubated with 1% TTC for 30 minutes at 37°C in the dark. After washing 3 times, the tissue was fixed in 4% paraformaldehyde. The noninfarcted myocardium was stained deep blue with Evans blue, the viable myocardium was stained red with TTC, and the necrotic myocardium appeared white after TTC staining. The area at risk (AAR) and the necrotic area were determined digitally using ImageJ (NIH).

### Measurement of cardiac function by echocardiography.

Echocardiographic measurements were performed on mice using a Visual Sonics Vevo 2100 Imaging System (Visual Sonics) with a 40 MHz MicroScan transducer (model MS-550D). Mice were anesthetized with isoflurane (2.5% for induction) and fastened on the platform. Echocardiography was performed on conscious mice when their heartbeat returned to ~600 beats per minute. Heart rate and LV dimensions, including diastolic and systolic wall thicknesses and LV end-diastolic and end-systolic chamber dimensions, were measured from 2-dimensional (2D) short axis under M-mode tracings at the level of the papillary muscle. LV mass and functional parameters such as fractional shortening and ejection fraction were calculated using the primary measurements and accompanying software. Data from at least 5 animals were collected for each group.

### Establishment of HFD-induced obesity in mice.

Mice around 8 weeks old were randomly divided into 2 groups: a standard diet (SD) group and a HFD group. The SD group was fed with normal chow, and the HFD group was fed with a HFD (D12492, Research Diets) with a fat energy supply ratio of 60%. Mice were raised in an SPF environment with constant temperature and humidity, a 12-hour light-dark cycle, free access to water, and 3–5 animals per cage. The general condition of the animals was observed daily, and body weight was measured weekly. After 20 weeks of feeding, mice were sacrificed for sample collection, and relevant indicators were tested.

### H&E staining.

Mice were euthanized, and the hearts were harvested. The hearts were fixed in 4% paraformaldehyde overnight, embedded in paraffin, and sectioned into 4 μm sections using a microtome. The sections were deparaffinized with xylene and rehydrated. The sections were immersed in hematoxylin solution for 10 seconds and eosin solution for 30 seconds, with washes performed between each step. After washing with tap water, the tissues were dehydrated and mounted. Imaging of sections was performed with Kfbio/KF-PRO-020.

### TUNEL assay.

A TUNEL kit (ServiceBio, G1502-50T) assay was used to identify apoptotic cells. The sections were deparaffinized with xylene and rehydrated. The sections were permeabilized with 50 μL of Proteinase K for 20 minutes at room temperature. The sections were then incubated for 2 hours at 37°C in the dark with staining solution containing deoxynucleotidyl transferase. After washing with PBS, the sections were mounted with DAPI mounting solution (Vector, H-1500).

### Sirius red collagen staining.

Mouse heart tissues were dissected out, rinsed with PBS, and fixed in 4% paraformaldehyde (pH 8.0) overnight. After dehydration through a series of ethanol baths, samples were embedded in paraffin wax according to standard laboratory procedures. For Sirius red collagen staining, sections were fixed with prewarmed Bouin’s solution at 55°C for 1 hour before being washed in running water. After rinsing in tap water, sections were stained in 0.1% Sirius red solution for 60 minutes. After staining, sections were dehydrated and cleared with xylene. The images were examined with a light microscope and quantified with ImageJ software. Data from at least 5 animals were collected for each group.

### qPCR and Western blot analysis.

Total RNAs were isolated using Trizol Reagent (Invitrogen) from cells and tissue samples. For qPCR, 2.0 μg RNA samples were reverse transcribed to cDNA using random hexamers and MMLV reverse transcriptase (Invitrogen) in a 20 μL reaction system. In each analysis, 0.1 μL of the cDNA pool was used for qPCR. Target gene expression levels were normalized against the expression of glyceraldehyde 3-phosphate dehydrogenase (*Gapdh*) or *18s* rRNA. Primers for qPCR are listed in the Supplemental Table 6. For Western blot analyses, tissue homogenate was cleared by 10,000*g* centrifugation for 10 minutes. Samples were subsequently analyzed by SDS/PAGE and transferred to PVDF membranes, which were incubated with 5% nonfat dry milk in TBST containing anti-IAMP (GLBiochem; customized antibodies as described previously) (20), anti–HIF-1α (CST, 36169S), anti-HSP90 (Proteintech, 13171-1-AP), anti–LAMIN B1 (Proteintech, 12987-1-AP), anti-FLAG (Sigma-Aldrich, F1804), anti–NF-κB P65 (Cell Signaling Technology [CST]; 8242S), anti–phospho–NF-κB p65 (CST, 3033S), anti-IκBα (CST, 4814S), anti–phospho-IκBα (CST, 2859S), anti-HA (CST, 3724S), anti-GAPDH (Proteintech, 60004-1-Ig), or anti–β-TUBULIN (Sigma-Aldrich, T0198) antibodies overnight at 4°C. Membranes were then washed 3 times with TBST buffer before adding the HRP goat anti–rabbit IgG (Proteintech, SA00001-2) secondary antibody. Specific protein bands were visualized through chemiluminescence detection.

### Generation of IAMP-null NIH-3T3 fibroblasts.

IAMP-null NIH-3T3 fibroblasts were generated using CRISPR-Cas9 genome editing. NIH-3T3 cells (ATCC, CRL-1658) were transfected with the PX459 vector containing the targeting gRNA to create a frameshift mutation in sORF for 1 day, followed by another 3-day selection with puromycin (3.2 mg/mL). The mixed cell population was then cultured in full medium for protein extraction or indicated experiments. Guide sequences for gRNAs used in this study include gRNA1, 5′-TCTTTCGTGTCTGATCCGGA-3′, and gRNA2, 5′-ACTCTGTCTGGGAAACCATC-3′.

### Isolation of cardiomyocytes and CFs from adult mice.

Eight-week-old mice were euthanized and administered heparin sodium via i.p. injection. The chest cavity was opened to expose the heart and aorta. The heart was then removed and placed in a calcium-free solution at 4°C. After the removal of the vena cava, pulmonary artery, and pulmonary vein, the aorta was tied to a 20 G needle to maintain coronary artery patency, followed by perfusion with 0.5 mL of calcium-free solution to remove residual blood. Additional perfusion and enzymatic digestion were facilitated using a Langendorff apparatus. Once the heart was softened, ventricular tissues were dissected and pipetted into a single-cell suspension using a Pasteur pipette. The suspension was filtered through a 100 μm mesh. Cardiomyocytes were separated from noncardiomyocytes and dead cells via gravity sedimentation. The remaining suspension containing noncardiomyocytes was centrifuged at 300*g* for 10 minutes to collect noncardiomyocytes, primarily CFs, which were then passaged for subsequent CF-related experiments.

### Immunofluorescence.

CFs were fixed with 4% paraformaldehyde for 15 minutes and permeabilized with PBS supplemented with 0.1% Triton X-100 for 10 minutes at room temperature. Cells were then blocked with 3% BSA in PBS for 1 hour, followed by incubation with anti-FLAG antibody (1:200; Sigma-Aldrich, F1804) in blocking buffer overnight at 4°C. After 3 washes with PBS, cells were incubated with Alexa Fluor 594–conjugated anti-mouse secondary antibodies (1:1,000; Thermo Fisher Scientific, A-11032) and the nuclear stain DAPI (0.1 mg/mL; MilliporeSigma, D9542) in PBS for 1 hour. Images were captured using a confocal microscope (Zeiss, LSM880).

### RNA-Seq and analysis.

Total RNA was extracted from CFs and heart tissues using Trizol Reagent (Takara). mRNA was enriched using oligo (dT)-attached magnetic beads, followed by fragmentation and cDNA synthesis. Steps included end repair, A-tailing, and adaptor ligation, followed by PCR amplification. To generate clean reads from RNA-Seq data, adapter sequences, low-quality, and unpaired reads were removed using Cutadapt (version 3.7). Quality assessment was performed using FastQC (version 0.11.9), and clean reads were aligned to the mouse reference genome (Mus_musculus.GRCm38) using HISAT2 (version 2.2.1). Gene expression levels were quantified using StringTie (version 2.1.4). Differential gene expression analysis was performed using DESeq2 (version 1.24.0) in RStudio (version 4.3.3). DEGs in IAMP-knockdown CFs were identified based on criteria of |log_2_ fold change| ≥ 1 and adjusted *P* ≤ 0.05, while DEGs in IAMP-knockdown CFs under IL-17 treatment were identified with criteria of |log_2_ fold change| ≥ 0.585 and adjusted *P* ≤ 0.05.

### ELISA.

For cultured cells, the medium was collected and centrifuged at 1,000*g* for 10 minutes to remove particles and polymers. IL-6, S100A8, and MMP3 concentrations in the medium of CFs were measured using ELISA kits (Elabscience and 4Abio) according to the manufacturer’s instructions. For myocardial tissues, an appropriate amount of mouse heart tissue was washed in precooled PBS to remove blood, and PBS containing the protease inhibitor PMSF was added. The tissue block was homogenized and centrifuged at 5,000*g* for 5 minutes at 4°C. The supernatant was collected for subsequent detection. IL-6 concentrations in the supernatant from heart tissue were measured using ELISA kits (R&D) following the manufacturer’s instructions. OD readings at 450 nm were obtained using an Omega reader.

### Coimmunoprecipitation assays.

HEK293 cells (ATCC, CRL-1573) with ectopic protein expression and CFs were harvested in lysis buffer composed of PBS containing 0.5% Triton X-100, 1 mM EDTA, 1 mM PMSF, and complete protease inhibitors (Roche); or cardiac tissues were lysed with the above lysis buffer. After debris removal by centrifugation at 10,000*g* for 10 minutes, proteins were incubated with anti-FLAG (Sigma-Aldrich, F1804), anti-HA (CST, 3724S), anti-HSP90 (Proteintech, 13171-1-AP) or anti-IAMP (GLBiochem, customized) antibodies, followed by precipitation with protein A/G beads. Precipitated proteins were analyzed by Western blotting with the indicated antibodies.

### Cell preparation for flow cytometry.

Mice were fully anesthetized, and the heart was quickly excised and perfused with ice-cold PBS to exclude blood cells. The left ventricles from sham-operated mice or ischemic areas of I/R hearts were excised, weighed, minced, and enzymatically digested in PBS buffer containing collagenase type II, DNase I, and elastase (Miltenyi Biotec) for 30 minutes at 37°C with gentle shaking. After complete digestion, RBCs were removed by adding RBC lysis solution. Cell debris was removed using Debris Removal Solution (Miltenyi Biotec) through centrifugation at 3000*g* for 10 minutes. Noncardiomyocytes were collected from the bottom. The collected cells were washed, centrifuged at 1000*g* for 10 minutes, and suspended in PBS. Cells were stained with PE-Cyanine7–conjugated CD45 antibody (clone 30-F11, TONBO) and measured on a CytoFLEX (Beckman). CytExpert software was used to analyze the percentage of CD45^+^ cells in the whole-cell population.

### Cytoplasmic and nuclear separation.

NIH-3T3 fibroblasts were harvested with trypsin-EDTA and centrifuged at 500*g* for 5 minutes. The supernatant was carefully removed, leaving the cell pellet as dry as possible. Ice-cold CER I (Thermo Fisher Scientific, 78833) was added to the cell pellet, and the tube was vortexed vigorously for 15 seconds to fully suspend the cell pellet. The tube was incubated on ice for 10 minutes. Ice-cold CER II (Thermo Fisher Scientific, 78833) was added, and the tube was vortexed for 5 seconds, incubated on ice for 1 minute, and vortexed again for 5 seconds. The tube was centrifuged at maximum speed (~16,000*g*) for 5 minutes. The supernatant (cytoplasmic extract) was transferred to a clean prechilled tube and placed on ice until use or storage. The insoluble fraction containing nuclei was suspended in ice-cold NER (Thermo Fisher Scientific, 78833), vortexed for 15 seconds, incubated on ice with intermittent vortexing for a total of 40 minutes, and centrifuged at 16000*g* for 10 minutes. The supernatant (nuclear extract) was transferred to a clean prechilled tube.

### Culture of human fibroblasts.

Human CFs were purchased from iCell (HUM-iCell-c003) and cultured in T-25 cell culture flasks. Upon reaching 85% confluence, the culture medium was replaced with 3 mL of sterile 1× PBS to ensure full coverage by horizontal placement of the flask. After PBS removal, 1 mL of digestive fluid was evenly distributed over the bottom surface and incubated for 1–2 minutes at 37°C. Cell detachment was confirmed under a microscope. The suspension was centrifuged at 300*g* for 5 minutes, the supernatant was discarded, and the cell pellet was resuspended in 2 mL of complete culture medium and evenly distributed into 2 new culture flasks. Incubation was carried out horizontally at 37°C with 5% CO_2_ for static culture.

### Computational prediction of PPI.

The docking complexes of IAMP/HSP90 obtained from GRAMM-X (https://gramm.compbio.ku.edu/) were uploaded to PDBePISA server (https://www.ebi.ac.uk/pdbe/pisa/) for analysis of the PPIs. Identification of hydrogen bonds, interacting interfaces, salt bridges, Gibbs free energy of binding (DGint, kcal/mol) in protein complexes were carried out. Mapping of the docking complexes was performed using PYMOL software (https://www.pymol.org).

### Statistics.

Mean ± SD was presented for each measurement unless otherwise stated. Normality of data was evaluated by the Shapiro-Wilk test if applicable. For comparisons between 2 groups, a 2-tailed Student’s *t* test was performed if the variable followed a normal distribution; otherwise, a Mann-Whitney *U* test was performed. For comparisons among multiple groups, 1-way or 2-way ANOVA (if there were 2 factor levels) was performed. For pairwise comparisons, post hoc tests were performed with Tukey’s correction. Values of *P* < 0.05 were considered statistically significant.

### Study approval.

All animal experiments were approved by Independent Ethics Committee for Clinical Research and Animal Trials of the First Affiliated Hospital of Sun Yat-sen University (protocol no. [2023]003). All procedures conformed to the 1964 Declaration of Helsinki and its later amendments or comparable ethics standards and were approved by the Ethics Committee of the First Affiliated Hospital of Sun Yat-sen University, Guangzhou, China.

### Data availability.

Supporting data values associated with the graphs in the main manuscript and the supplemental material are provided in the Supporting Data Values file. Values for each figure are presented in separate tabs. The raw sequencing data generated in this study are available at the NCBI GEO (GSE26613). (https://www.ncbi.nlm.nih.gov/geo/query/acc.cgi?acc=GSE266133)

## Author contributions

YY, TZ, and XH performed most of the experiments. GD and XX performed the IR on mice. TD and ZC performed bioinformatic analyses. JW, HZ, and YP built viral vectors and designed siRNAs/gRNAs used in this study. YL isolated and maintained fibroblasts used in this study. CL, XL, YD, and JSO performed quality control of all data and revised the manuscript. ZPH supervised the study and wrote the manuscript.

## Supplementary Material

Supplemental data

Unedited blot and gel images

Supporting data values

## Version 1 03/20/2025

In-Press Preview

## Version 2 05/08/2025

Electronic publication
