# Supplementary material for: A cardiac fibroblast-enriched micropeptide regulates inflammation in ischemia/reperfusion injury
Source: JCI Insight. 2025 Mar 20;10(9):e187848. doi: 10.1172/jci.insight.187848 (PMC12128956; doi:10.1172/jci.insight.187848)
Supplement: Supplemental data [file jciinsight-10-187848-s237.pdf]

A cardiac fibroblast-enriched micropeptide regulates inflammation in ischemia/reperfusion injury

Youchen Yan, Tingting Zhang, Xin He, Tailai Du, Gang Dai, Xingfeng Xu, Zhuohui Chen, Jialing Wu, Huimin Zhou, Yazhi Peng, Yan Li, Chen Liu, Xinxue Liao, Yugang Dong, Jingsong Ou, Zhan-Peng Huang

Supplemental Figure legends

Supplemental Figure 1. (A) Conservation analysis of the ORF sequence of IAMP in vertebrates using phastCons and phyloP algorithm (https://genome.ucsc.edu/). **(B)** The 3D structure of human IAMP protein predicted by AlphaFold. **(C)** IAMP expression profile in different human tissues generated by the GTEx Portal (Genotype-Tissue Expression Portal). **(D)** IAMP expression profile in different mouse tissue (BioProject: PRJNA66167)

Supplemental Figure 2. (A) Volcano plot showing transcriptomic change in cardiac fibroblasts with IAMP knocked-down vs. control. **(B)** qRT-PCR confirmation of selected differentially expressed genes in transcriptome analysis. n=4 for each group. *: $p < 0.05$; **: $p < 0.01$; ***: $p < 0.001$; ****: $p < 0.0001$, by ANOVA with Tukey's correction.

Supplemental Figure 3. (A) qRT-PCR analysis of mRNA levels of Col1a1, Ctgf and Postn in control and IAMP-KD cardiac fibroblasts with or without TGFβ (10ng/ml) treatment. n=3 for each group. **(B)** Western blotting of ACTA2 protein in control and IAMP-KD cardiac fibroblasts with or without TGFβ (10ng/ml) treatment. **(C)** Volcano plot showing transcriptomic change in IAMP-KD cardiac fibroblasts treated with TGFβ. **(D)** KEGG pathway enrichment analysis of up-regulated (red) and down-regulated (blue) genes in IAMP-KD cardiac fibroblasts treated with TGFβ.

Supplemental Figure 4. (A) Results of Sanger sequencing showing the genomic change in IAMP gene locus in NIH-3T3 cells after CRISPR/Cas9 editing. **(B)** Western blotting of IAMP protein in IAMP-null and control NIH-3T3 cells. **(C)** qRT-PCR analysis of mRNA levels of Il6, S100a8, and Mmp3 in IAMP-null and control NIH-3T3 cells. n=3 for each group. **(D)** ELISA detecting protein

levels of IL-6, S100A8, and MMP3 in the culture medium of IAMP-null and control NIH-3T3 cells. n=3 for each group. **(E)** qRT-PCR of mRNA levels of Il6, S100a8, and Mmp3 in cardiac fibroblasts infected with indicated siRNAs with or without LPS (20ng/ml) treatment. n=3 for each group. **(F)** qRT-PCR of mRNA levels of Il6, S100a8, and Mmp3 in cardiac fibroblasts infected with indicated siRNAs with or without TNFα (10ng/ml) treatment. n=3 for each group. *: $p < 0.05$; **: $p < 0.01$; ***: $p < 0.001$; ****: $p < 0.0001$, by ANOVA with Tukey's correction.

Supplemental Figure 5. qRT-PCR analysis of mRNA levels of Il6, S100a8, and Il1b in cardiomyocytes and non-cardiomyocytes from control (Sham) and I/R hearts by Langendorff approach. n=4 for each group. *: $p < 0.05$; **: $p < 0.01$; ***: $p < 0.001$, by ANOVA with Tukey's correction.

Supplemental Figure 6. (A) Correlation between Il6, S100a8, Il1b and Cxcl1, and IAMP mRNA levels in the GSE160516 RNA-seq dataset (n=16). **(B)** Correlation between Cxcl1 and S100a8, and IAMP mRNA levels in hearts at different time points after I/R or sham surgery (n=38). **(C)** Correlation between Il6 and S100a8, and IAMP mRNA levels in the GSE159612 RNA-seq dataset (n=53). **(D)** Correlation between Mmp3, Il1b, S100a8 and Cxcl1, and IAMP mRNA levels in hearts from mice fed with normal or high-fat diet (n=29). Statistical analysis was performed using Pearson correlation.

Supplemental Figure 7. (A) Western blotting of IAMP protein in the lung from control (Ctrl) and IAMP-KO adult mice. **(B)** qRT-qPCR analysis of IAMP mRNA levels in the heart of Ctrl and IAMP-KO adult mice. n=5 for each group. **(C)** Fractional shortening (FS), left ventricular posterior wall thickness at end-diastole (LVPW;d) and left ventricular internal dimension at end-systole (LVID;d) accessed by echocardiography in 10-week-old and 7-month-old Ctrl and IAMP-KO mice. n=3-5 for each group. **(D)** PCA analysis of the cardiac transcriptome from Ctrl and IAMP-KO adult mice. n=4 for each group. Statistical significance indicated in this figure was determined using a two-tailed unpaired t-test to assess differences between two groups. ns: not significant.

Supplemental Figure 8. (A) TUNEL assay and quantification of apoptotic cells in cross-sections of hearts from Ctrl and IAMP-KO mice 24 hours after sham surgery. n=5-6 for each group. **(B)**

qRT-PCR analysis of mRNA levels of hypertrophic marker genes in hearts from Ctrl or IAMP-KO mice 2 weeks after I/R or sham surgery. n=7-8 for each group. *: $p < 0.05$; **: $p < 0.01$; ***: $p < 0.001$; ****: $p < 0.0001$, ns: not significant, by ANOVA with Tukey's correction.

Supplemental Figure 9. **(A)** Western blotting of total and phosphorylated IκBa (p-IκBa) and p65 (p-p65) in cardiac fibroblasts transfected with indicated siRNAs at different time points. **(B)** Western blotting of total and phosphorylated IκBa (p-IκBa) and p65 (p-p65) in cardiac fibroblasts transfected with indicated siRNAs followed by IL-17, TNFα or LPS treatment. **(C)** qRT-PCR analysis of mRNA levels of NF-κB signaling-related genes in control and IAMP-KD cardiac fibroblasts treated with IL-17. n=3 for each group. **(D)** Pathway enrichment analysis of up-regulated genes in the Molecular Signatures Database (MSigDB). *: $p < 0.05$, ns: not significant, by ANOVA with Tukey's correction.

Supplemental Figure 10. **(A)** Western blotting and quantification of HIF-1α protein in cardiac fibroblasts transfected with indicated siRNAs. n=3 for each group. **(B)** qRT-PCR analysis of mRNA levels of Il6, S100a8, Mmp3, and Cxcl3 in cardiac fibroblasts transfected with indicated adenoviruses. n=3 for each group. **(C)** PyMOL representation showing polar interactions between the IAMP and HSP90 proteins. IAMP and HSP90 are colored in purple and blue, respectively. *: $p < 0.05$; **: $p < 0.01$; ***: $p < 0.001$; ns: not significant, by ANOVA with Tukey's correction.

Supplemental Figure 1

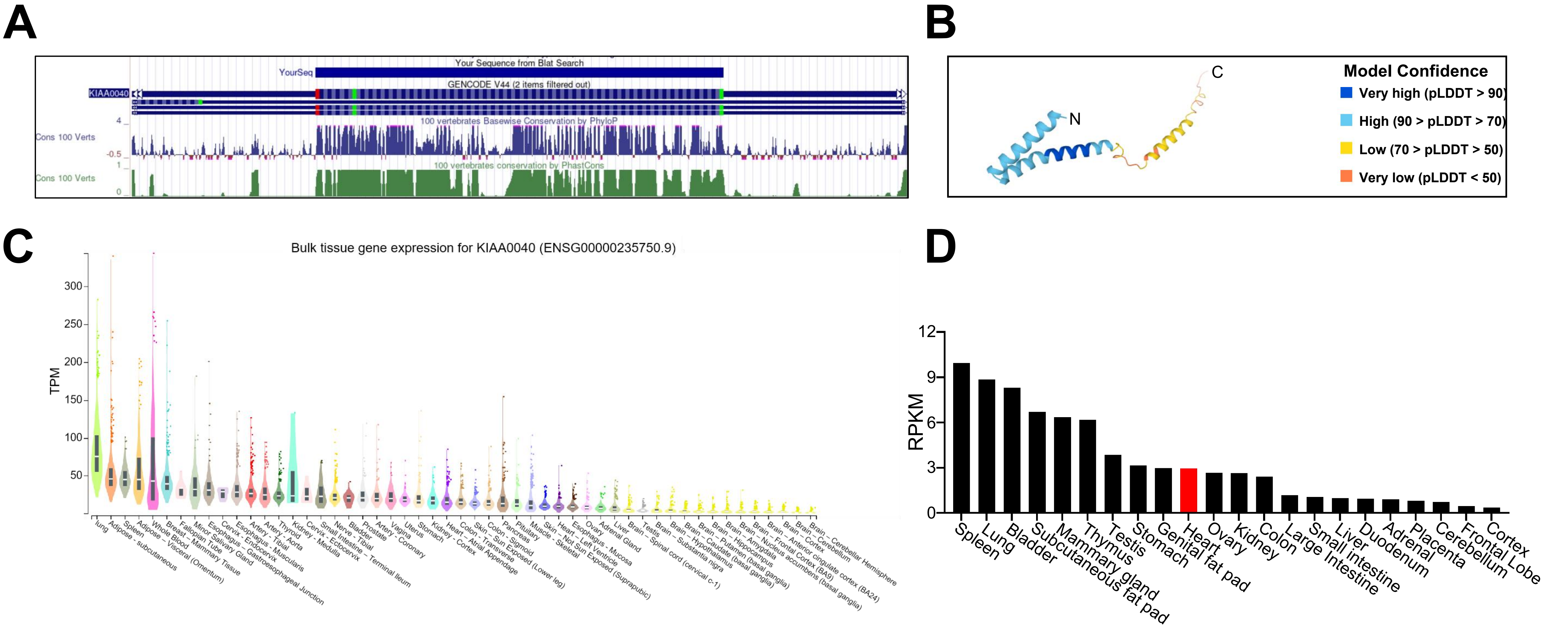

Supplemental Figure 2

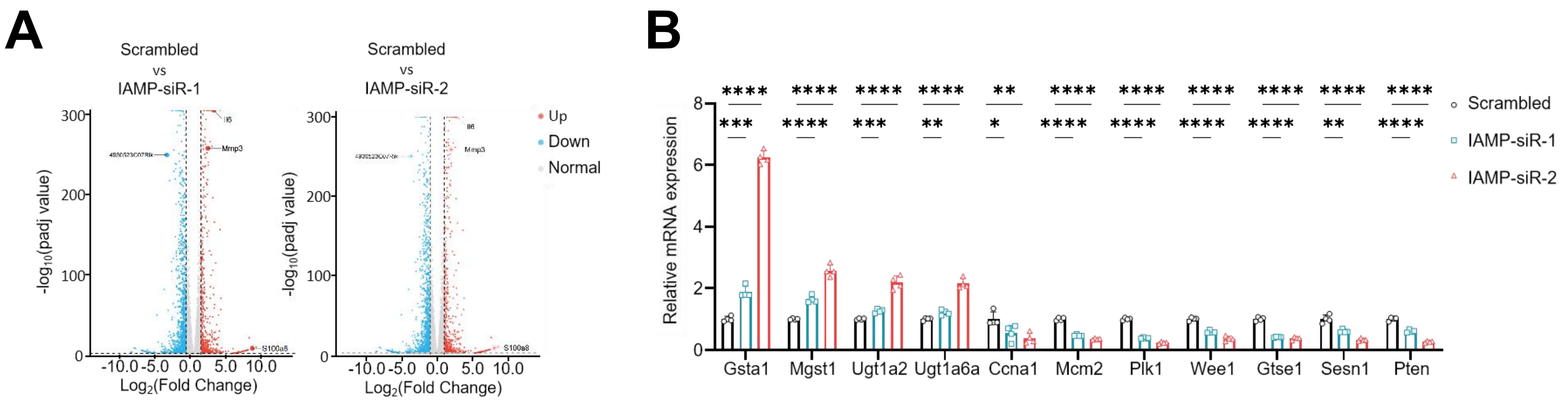

Supplemental Figure 3

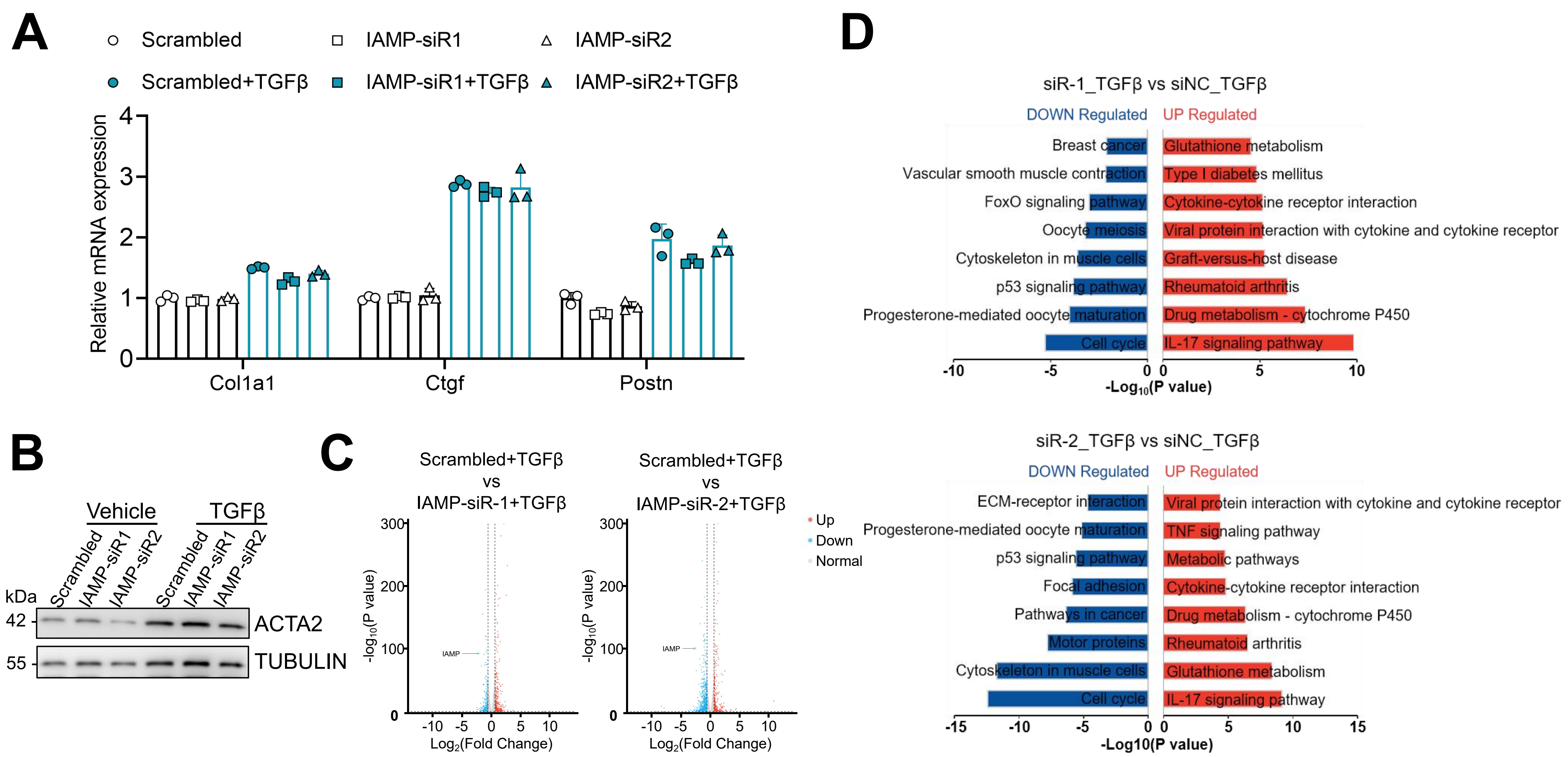

Supplemental Figure 4

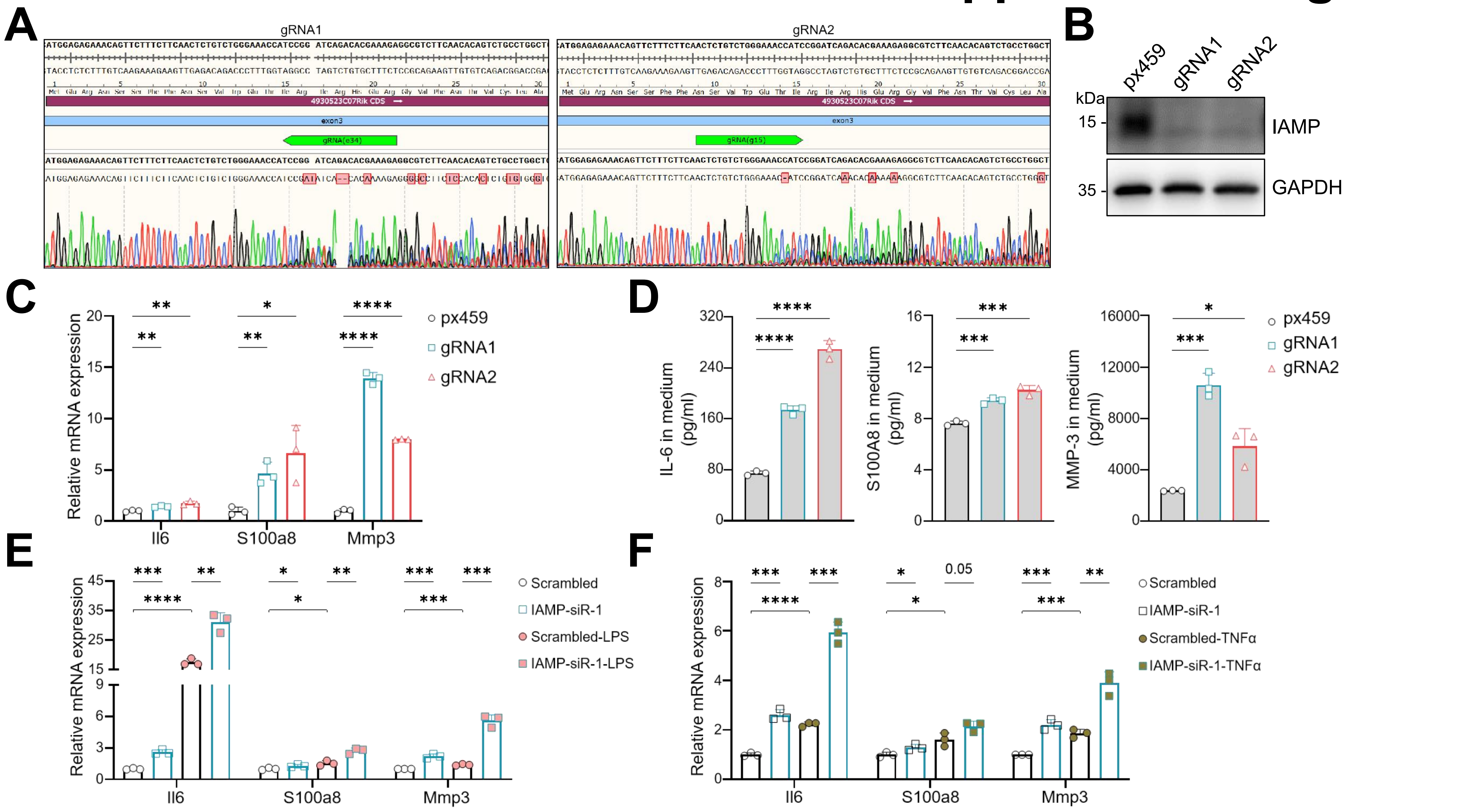

Supplemental Figure 5

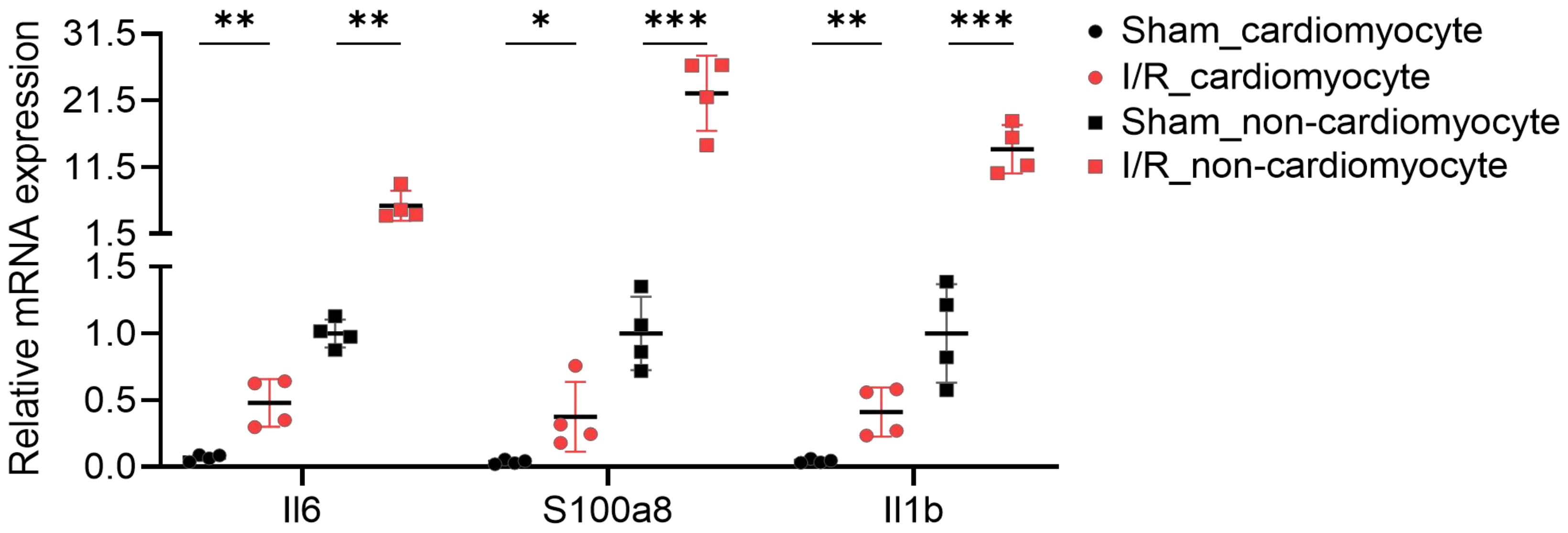

Supplemental Figure 6

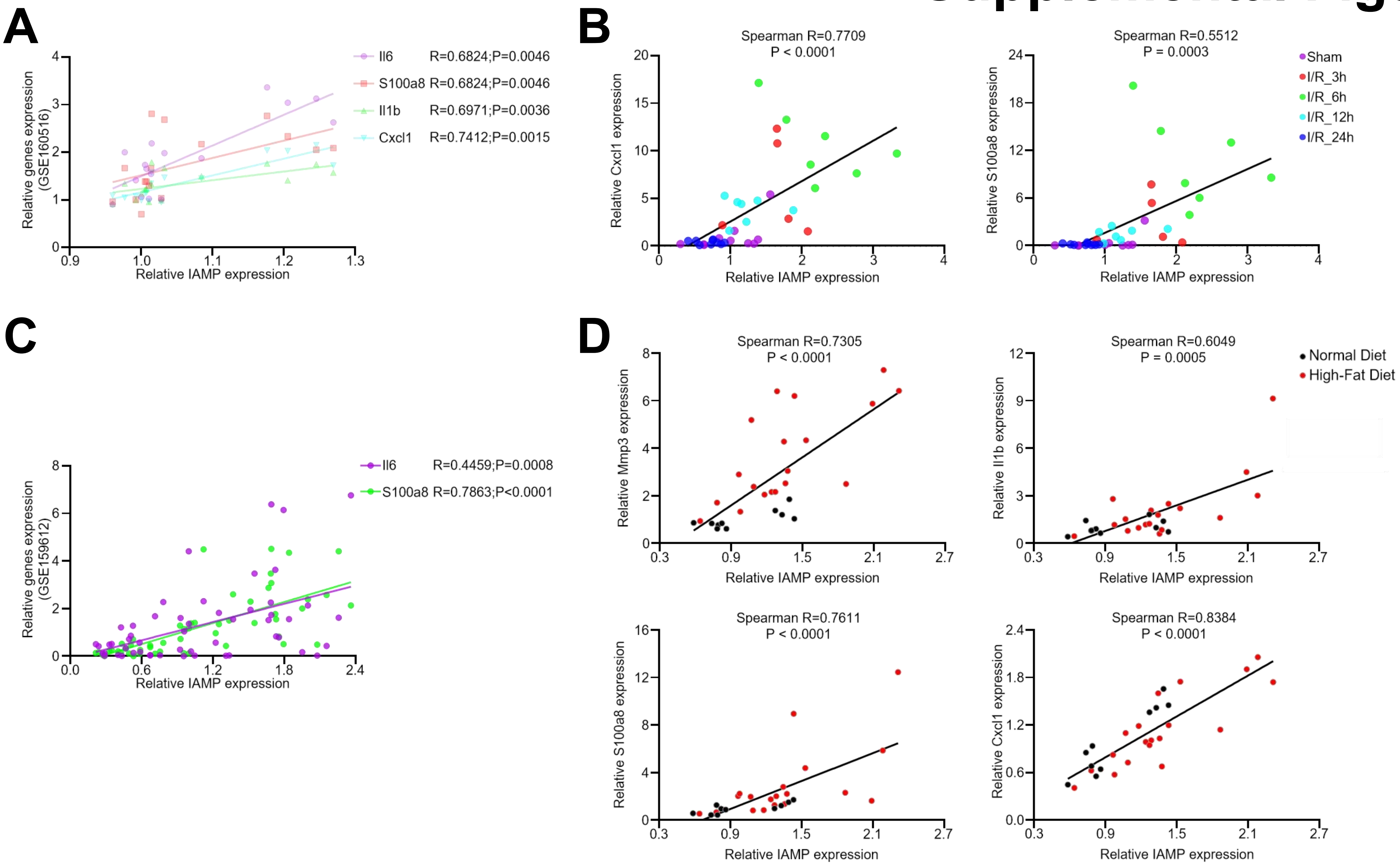

Supplemental Figure 7

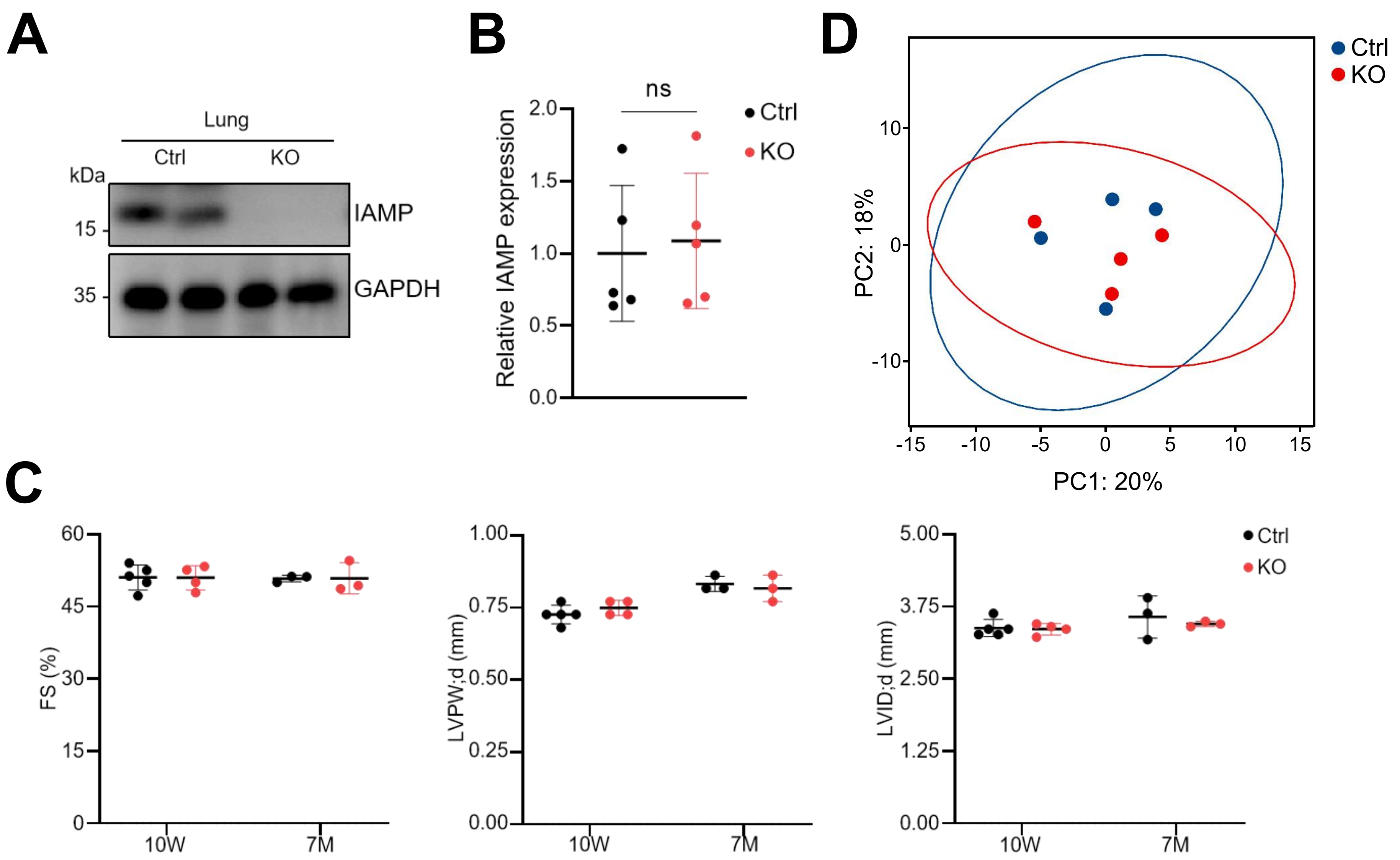

Supplemental Figure 8

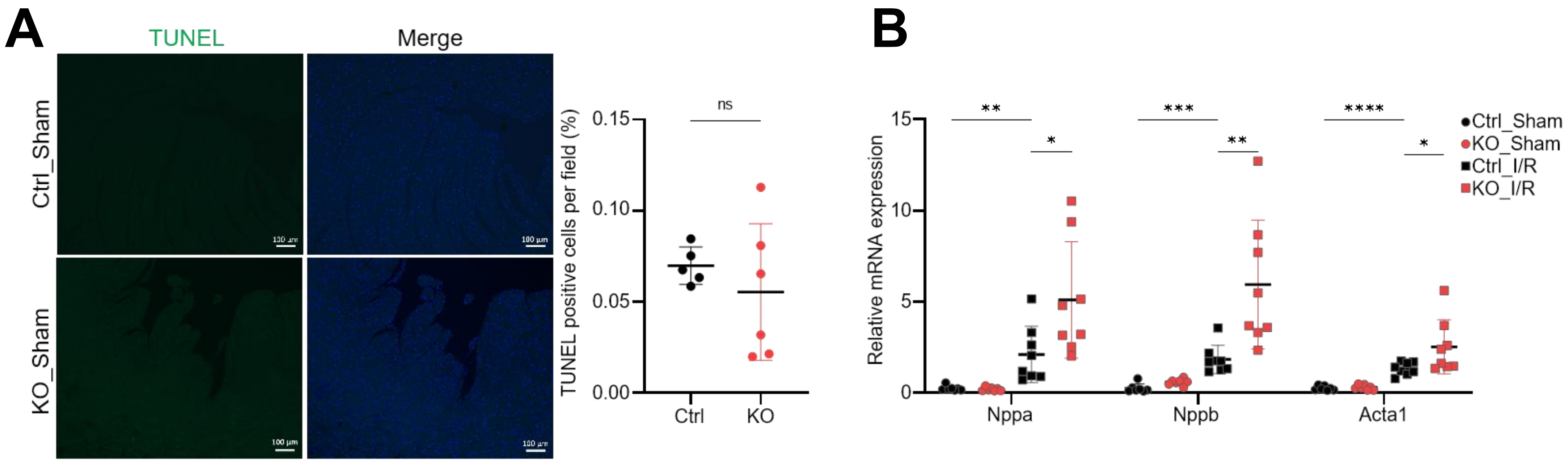

Supplemental Figure 9

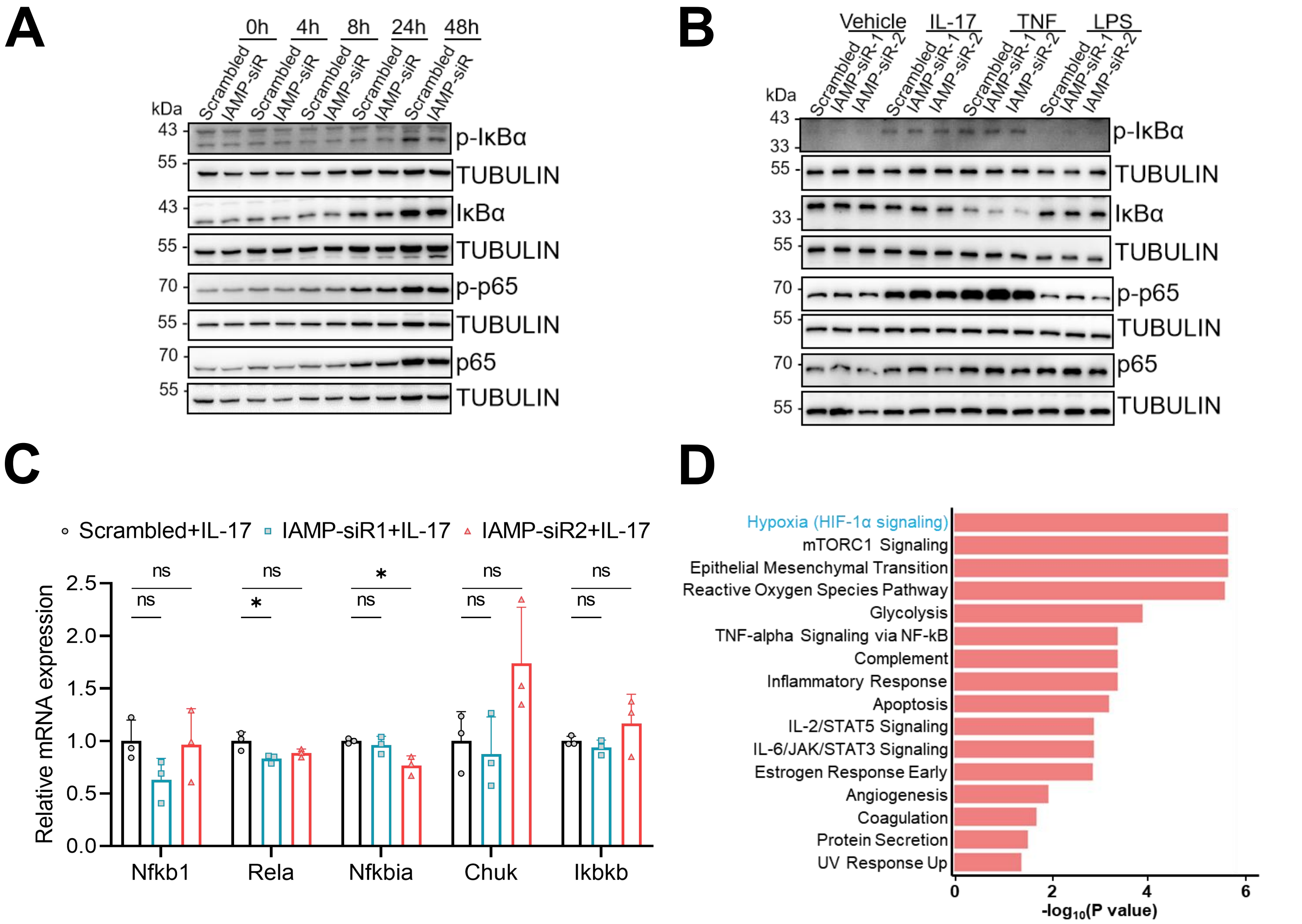

Supplemental Figure 10

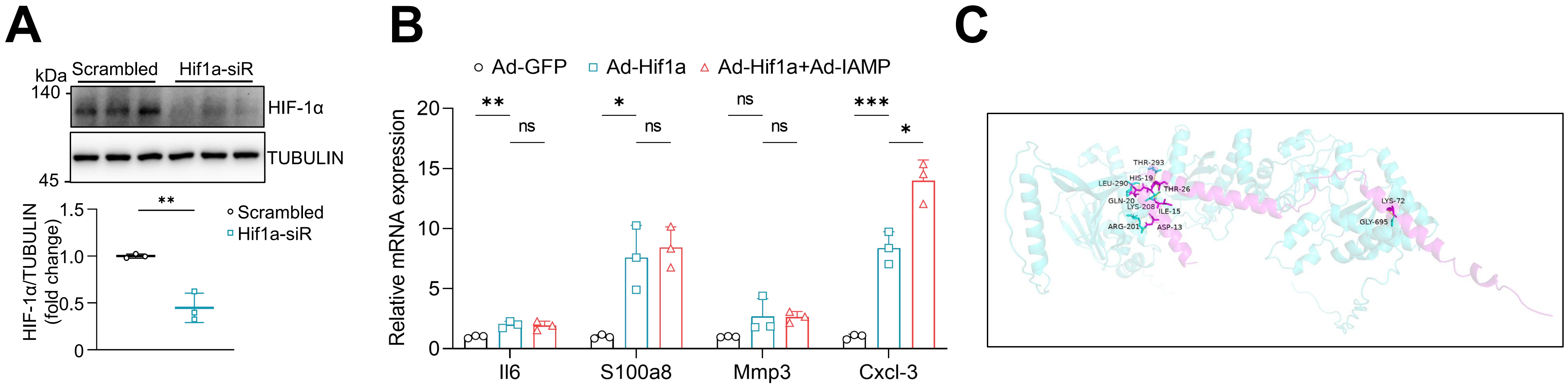

Supplemental Table 1. Differentially expressed genes in IAMP knockdown cardiac fibroblasts

siR-1 vs siNC

ensembl_gene_id	gene_name	log2FoldChange	padj
ENSMUSG00000049692	Tmem239	7.248460704	2.03E-05
ENSMUSG00000083500	Gm15470	7.072658668	3.02E-05
ENSMUSG00000118153	Gm50288	6.772394687	0.000197412
ENSMUSG00000020673	Tpo	6.742134518	0.000221887
ENSMUSG00000045942	BC049762	6.718534692	0.000354547
ENSMUSG00000038883	Prl3a1	6.693500427	0.000346786
ENSMUSG00000100432	Gm7539	6.688477979	0.000293277
ENSMUSG00000074403	H3c13	6.578471296	0.000405999
ENSMUSG00000115855	Gm34643	6.55129552	0.000702261
ENSMUSG00000020676	Ccl11	6.488745018	0.000875662
ENSMUSG00000116210	Spn-ps	6.454461349	0.001013688
ENSMUSG00000109039	Gm33570	6.424653363	0.000954563
ENSMUSG00000092353	Gm20539	6.424017774	0.000886657
ENSMUSG00000075224	Lrrc55	6.423811695	0.001138585
ENSMUSG00000026523	Wdr64	6.356839903	0.001243819
ENSMUSG00000097310	A930038B10Rik	6.288540446	0.010818052
ENSMUSG00000108476	Gm44974	6.287843141	0.002099858
ENSMUSG00000005864	Cnga2	6.251214078	0.002530182
ENSMUSG00000073184	Gm49957	6.215981043	0.011694919
ENSMUSG00000056054	S100a8	6.210344391	0.01099823
ENSMUSG00000107932	Gm44432	6.208777906	0.002723836
ENSMUSG00000107455	Gm43868	6.135641097	0.00360205
ENSMUSG00000084139	Gm12322	6.131565122	0.003568129
ENSMUSG00000110899	Gm48840	6.001745782	0.020797562
ENSMUSG00000058470	Gm8369	5.96463216	0.006107063
ENSMUSG00000111761	9230112J17Rik	5.922722169	0.024885672
ENSMUSG00000066652	Lefty2	5.922342975	0.024610424
ENSMUSG00000022044	Stmn4	5.920009756	0.008062957
ENSMUSG00000085171	D830026I12Rik	5.875243377	0.027235508
ENSMUSG00000025038	Efhc2	5.870702596	0.008479178
ENSMUSG00000085664	Atxn7l1os2	5.86876479	0.008788238
ENSMUSG00000085803	Gm12056	5.866542745	0.027956023
ENSMUSG00000052276	Ostn	5.826394147	0.030922346
ENSMUSG00000070806	Zmynd12	5.821099888	0.01028778
ENSMUSG00000102670	Gm37436	5.820055656	0.011024565
ENSMUSG00000097085	Gm26634	5.819445323	0.03120177
ENSMUSG00000097513	Gm26696	5.817677433	0.011873004
ENSMUSG00000036634	Mag	5.815173567	0.033687236
ENSMUSG00000055561	Spink5	5.773008903	0.012832345
ENSMUSG00000103380	Gm37756	5.77089209	0.011865097
ENSMUSG00000062073	Gm10109	5.768355314	0.012891062
ENSMUSG00000055471	Alk	5.766945293	0.035506525
ENSMUSG00000087624	9230111E07Rik	5.723130044	0.039311995
ENSMUSG00000097892	Gm26801	5.719453501	0.042471399
ENSMUSG00000092309	4930518C09Rik	5.71624158	0.016120674
ENSMUSG00000019359	Gdpd2	5.715597528	0.015416428
ENSMUSG00000112744	Gm48880	5.71503402	0.01595173
ENSMUSG00000056290	Ms4a4b	5.658303527	0.046705886
ENSMUSG00000113362	Gm40557	5.607548378	0.020733356
ENSMUSG00000114378	Gm49355	5.6057356	0.020816424
ENSMUSG00000081871	Gm11488	5.544012793	0.02937978
ENSMUSG00000116768	Gm18269	5.489436981	0.031702919
ENSMUSG00000022755	Adgrg7	5.486608574	0.029739529
ENSMUSG00000115131	Gm49268	5.352145452	0.046038258
ENSMUSG00000116347	Gm49416	4.763311144	0.000647187
ENSMUSG00000109941	Exosc6	4.598603188	0.014464593
ENSMUSG00000021700	Rab3c	4.497129773	0.001133966
ENSMUSG00000069223	4930451E10Rik	4.411726529	0.006128451
ENSMUSG00000103442	Pcdha1	4.223958523	0.04569241
ENSMUSG00000096051	Vmn1r40	4.010245163	0.003863189
ENSMUSG00000028327	Stra6l	3.99524955	0.006072614
ENSMUSG00000090171	Ugt1a2	3.991366007	0.019943241
ENSMUSG00000027925	Sprr2j-ps	3.972667702	0.001718954
ENSMUSG00000071679	Rtl4	3.957141742	0.000489485
ENSMUSG00000064246	Chil1	3.947123536	0.003798769
ENSMUSG00000100303	2600014E21Rik	3.846303427	1.05E-07
ENSMUSG00000103266	Gm36979	3.78447435	0.005897197
ENSMUSG00000028128	F3	3.771813875	0
ENSMUSG00000044708	Kcnj10	3.637482443	0.009023915
ENSMUSG00000096169	Olfr1564	3.633151172	0.011791445
ENSMUSG00000021983	Atp8a2	3.295763489	0.001397585
ENSMUSG00000029371	Cxcl5	3.264750598	0
ENSMUSG00000006490	Prl8a9	3.261102315	6.41E-27
ENSMUSG00000049719	Prss46	3.257071346	0.001352202

siR-2 vs siNC

ensembl_gene_id	gene_name	log2FoldChange	padj
ENSMUSG00000020676	Ccl11	8.591224011	8.32E-10
ENSMUSG00000056054	S100a8	8.046386985	3.36E-08
ENSMUSG00000090171	Ugt1a2	7.627191543	9.09E-21
ENSMUSG00000030483	Cyp2b10	7.474893685	1.94E-06
ENSMUSG00000115727	4930432J09Rik	7.413108079	3.50E-06
ENSMUSG00000075224	Lrrc55	7.323101515	1.08E-05
ENSMUSG00000116210	Spn-ps	7.261099001	8.44E-06
ENSMUSG00000076617	Ighm	7.147433667	1.46E-05
ENSMUSG00000109369	Vmn2r-ps88	7.130652338	1.74E-05
ENSMUSG00000021640	Naip1	7.126668719	2.35E-05
ENSMUSG00000087545	Gm13966	6.983827877	4.37E-05
ENSMUSG00000090527	Aadacl2fm2	6.893820092	6.74E-05
ENSMUSG00000021943	Gdf10	6.827860076	0.000115286
ENSMUSG00000109629	Gm34099	6.776922323	0.00014691
ENSMUSG00000055561	Spink5	6.749963687	0.000180075
ENSMUSG00000091215	Pate1	6.701674682	0.000180479
ENSMUSG00000086962	Gm12248	6.675418608	0.000208842
ENSMUSG00000070806	Zmynd12	6.645820865	0.000241422
ENSMUSG00000116180	Gm49492	6.62348086	0.000350926
ENSMUSG00000118153	Gm50288	6.445540569	0.000900804
ENSMUSG00000109039	Gm33570	6.438370472	0.000974593
ENSMUSG00000114378	Gm49355	6.382510586	0.001071921
ENSMUSG00000027869	Hsd3b6	6.376310322	0.001193751
ENSMUSG00000091864	Gm17102	6.374047	0.001482649
ENSMUSG00000050201	Otop2	6.373685296	0.001181967
ENSMUSG00000115575	Gm49024	6.305609227	0.006660325
ENSMUSG00000087360	Gm15104	6.275215304	0.001457545
ENSMUSG00000109127	Gm31135	6.274449672	0.001491483
ENSMUSG00000037469	Acp7	6.274395776	0.002566826
ENSMUSG00000039720	Got1l1	6.239065897	0.00203654
ENSMUSG00000047222	Rnase2a	6.206645888	0.002094786
ENSMUSG00000022886	Prl2a1	6.201968213	0.002019811
ENSMUSG00000048776	Pthlh	6.167315105	0.002600114
ENSMUSG00000026011	Ctla4	6.144457987	0.000208391
ENSMUSG00000066652	Lefty2	6.134746385	0.011013705
ENSMUSG00000118038	Gm9895	6.126628776	0.002876598
ENSMUSG00000112058	Gm47593	6.093168117	0.012530022
ENSMUSG00000052131	Akr1b7	6.092416947	0.003538268
ENSMUSG00000035131	Brinp3	6.089949481	0.012350232
ENSMUSG00000021587	Pcsk1	6.086078101	0.012891168
ENSMUSG00000045394	Epcam	6.053323337	0.01371344
ENSMUSG00000054910	4931415C17Rik	6.008320002	0.004365605
ENSMUSG00000103270	Gm37915	6.006972343	0.00429485
ENSMUSG00000092309	4930518C09Rik	6.005656743	0.004556691
ENSMUSG00000086709	Gm16263	6.004515788	0.00441923
ENSMUSG00000107455	Gm43868	6.002731517	0.004800953
ENSMUSG00000033255	Gm5134	5.96897684	0.01811526
ENSMUSG00000056290	Ms4a4b	5.966089292	0.017818652
ENSMUSG00000038883	Prl3a1	5.964921881	0.005255178
ENSMUSG00000068234	Vmn1r44	5.927256319	0.021829663
ENSMUSG00000085664	Atxn7l1os2	5.924486451	0.007386022
ENSMUSG00000085171	D830026I12Rik	5.918558056	0.006413787
ENSMUSG00000041608	Entpd3	5.916400785	0.006224695
ENSMUSG00000026241	Nppc	5.916396612	0.020421688
ENSMUSG00000102822	1700113B19Rik	5.914989746	0.007170947
ENSMUSG00000074403	H3c13	5.913404704	0.020561355
ENSMUSG00000047502	Mroh7	5.869350631	0.023530578
ENSMUSG00000078500	Zfp986	5.827131316	0.009668783
ENSMUSG00000117350	Gm49975	5.823049531	0.008835024
ENSMUSG00000097924	A730020E08Rik	5.822286844	0.009229987
ENSMUSG00000086256	Gm12052	5.780791606	0.028626344
ENSMUSG00000115611	Gm4606	5.778591635	0.010021563
ENSMUSG00000078872	Gm14401	5.775203753	0.028975718
ENSMUSG00000099512	Gm28703	5.771520729	0.012342947
ENSMUSG00000115355	4930445E18Rik	5.771118955	0.029238637
ENSMUSG00000021255	Esrrb	5.730051752	0.032106573
ENSMUSG00000058470	Gm8369	5.727329204	0.012114501
ENSMUSG00000105049	Gm42486	5.726720804	0.034802231
ENSMUSG00000020436	Gabrg2	5.725719504	0.032408823
ENSMUSG00000096001	2610528A11Rik	5.724066944	0.011996228
ENSMUSG00000052276	Ostn	5.723786192	0.01218218
ENSMUSG00000107203	Gm7542	5.718757292	0.03307336
ENSMUSG00000113632	Gm47446	5.670183165	0.038045253
ENSMUSG00000083500	Gm15470	5.625433323	0.040968932

ENSMUSG00000027408	Cpxm1	3.16240739	0	ENSMUSG00000086727	4931428L18Rik	5.619823073	0.01659017
ENSMUSG00000031932	Gpr83	3.155415141	1.76E-06	ENSMUSG00000109572	Cfap99	5.615714383	0.01824247
ENSMUSG00000117764	Gm50143	3.126633246	0.007592632	ENSMUSG00000090406	Gm17058	5.614124874	0.042971647
ENSMUSG00000020374	Rasgef1c	3.036375509	2.42E-21	ENSMUSG00000118147	Gm41555	5.611782604	0.042362724
ENSMUSG00000031289	Il13ra2	2.987005059	4.06E-11	ENSMUSG00000045942	BC049762	5.611513462	0.048894262
ENSMUSG00000006567	Atp7b	2.81299665	0.009815561	ENSMUSG00000024215	Spdef	5.610303173	0.045720269
ENSMUSG00000091230	H1f11-ps	2.78705278	0.042646825	ENSMUSG00000042244	Pglyrp3	5.568952251	0.048065254
ENSMUSG00000026166	Ccl20	2.775758718	2.90E-14	ENSMUSG00000104828	Gm42898	5.563619843	0.049387735
ENSMUSG00000108122	Gm44116	2.758514003	0.000817894	ENSMUSG00000112673	Gm7413	5.562760043	0.01969118
ENSMUSG00000027347	Rasgrp1	2.753138125	0.010143676	ENSMUSG00000114790	4921509O07Rik	5.562149182	0.019624075
ENSMUSG00000037994	Slc9b2	2.748657916	1.62E-14	ENSMUSG00000049692	Tmem239	5.559703312	0.048337922
ENSMUSG00000056771	Gm10010	2.740599704	0.046040945	ENSMUSG00000040726	Hesx1	5.556233679	0.047541193
ENSMUSG00000009185	Ccl8	2.738737446	1.19E-18	ENSMUSG00000092536	Gm20501	5.50682274	0.024278618
ENSMUSG00000023914	Mep1a	2.700409365	0.031293453	ENSMUSG00000083774	Gm7180	5.447682359	0.034503375
ENSMUSG00000095139	Pou3f2	2.693683546	0.000971895	ENSMUSG00000107008	Gm2762	5.445673094	0.029305299
ENSMUSG00000107411	Gm19040	2.670721789	0.012780978	ENSMUSG00000048834	Vstm2a	5.417553894	0.011525353
ENSMUSG00000029379	Cxcl3	2.655063157	8.37E-19	ENSMUSG00000050635	Sprr2f	5.378788432	1.05E-26
ENSMUSG00000003949	Hlf	2.617409881	5.56E-20	ENSMUSG00000097370	4930467K11Rik	5.375919123	0.035430502
ENSMUSG00000005413	Hmox1	2.613880183	0	ENSMUSG00000112095	A130077B15Rik	5.311010295	0.039559745
ENSMUSG00000049100	Pcdh10	2.578116775	2.97E-07	ENSMUSG00000090175	Ugt1a9	4.776828285	0.000110774
ENSMUSG00000075707	Dio3	2.576622382	0.001652671	ENSMUSG00000109941	Exosc6	4.635990707	0.010551077
ENSMUSG00000107667	C530044C16Rik	2.563114883	0.028523664	ENSMUSG00000028327	Stra6l	4.385732908	0.000120054
ENSMUSG00000015134	Aldh1a3	2.547688735	0	ENSMUSG00000036218	Pdzrn4	4.043878674	0.000217542
ENSMUSG00000021070	Bdkrb2	2.536313728	3.85E-05	ENSMUSG00000049537	Tecrl	4.022243785	0.000861749
ENSMUSG00000079105	C7	2.524168523	1.33E-08	ENSMUSG00000007653	Gabrb2	3.935154442	0.00306522
ENSMUSG00000050830	Vwc2	2.509150593	0.037570919	ENSMUSG00000087070	Gm12505	3.860159365	0.014087221
ENSMUSG00000117604	Gm33228	2.50230996	1.01E-40	ENSMUSG00000022018	Rgcc	3.827856341	1.33E-08
ENSMUSG00000068794	Col28a1	2.492048377	6.22E-244	ENSMUSG00000103266	Gm36979	3.827554542	0.004125528
ENSMUSG00000047497	Adamts12	2.485550943	0	ENSMUSG00000027925	Sprr2j-ps	3.796053816	0.003011238
ENSMUSG00000050587	Lrrc4c	2.472839294	4.28E-22	ENSMUSG00000087597	Gm15345	3.781250317	0.019349436
ENSMUSG00000052271	Bhlha15	2.47178324	7.82E-09	ENSMUSG00000056300	Zfp981	3.761129597	0.008000834
ENSMUSG00000026185	Igfbp5	2.440382935	3.29E-36	ENSMUSG00000009185	Ccl8	3.757665402	2.70E-62
ENSMUSG00000041620	Mmp1b	2.423451793	1.41E-14	ENSMUSG00000029379	Cxcl3	3.72464814	6.96E-53
ENSMUSG00000097111	Peak1os	2.375531769	3.53E-05	ENSMUSG00000057726	Serpinb9g	3.71989927	0.030419377
ENSMUSG00000109771	Gm35315	2.365829859	7.58E-10	ENSMUSG00000028766	Alpl	3.654722189	0
ENSMUSG00000032878	Ccdc85a	2.325381213	1.37E-12	ENSMUSG00000055030	Sprr2e	3.647556443	0.000197326
ENSMUSG00000034755	Pcdh11x	2.320093455	5.45E-19	ENSMUSG00000062345	Serpinb2	3.630060188	3.07E-154
ENSMUSG00000050069	Grem2	2.317556492	4.66E-11	ENSMUSG00000090230	Gm16315	3.625682067	0.007642968
ENSMUSG00000035373	Ccl7	2.300687473	0	ENSMUSG00000096051	Vmn1r40	3.615287017	0.024775911
ENSMUSG00000085576	Dpy19l2	2.291241999	0.04265818	ENSMUSG00000051008	4930412M03Rik	3.603982457	0.012573393
ENSMUSG00000012428	Steap4	2.270504748	5.53E-13	ENSMUSG00000110397	Gm45540	3.60392064	0.01146288
ENSMUSG00000052496	Pkdrej	2.253753431	0.045355452	ENSMUSG00000111709	Gm3776	3.531876623	4.39E-28
ENSMUSG00000085180	AI838599	2.209305787	0.017415983	ENSMUSG00000010142	Tnfrsf13b	3.51838978	0.012235307
ENSMUSG00000054215	Sprr2k	2.190439462	0.00191535	ENSMUSG00000050092	Sprr2b	3.4930289	2.24E-05
ENSMUSG00000043085	Tmem82	2.174098756	1.07E-13	ENSMUSG00000114736	Gm47336	3.478040744	0.028918266
ENSMUSG00000082248	Gm13161	2.122018004	0.036354656	ENSMUSG00000021348	Prl7d1	3.421062853	0.004731483
ENSMUSG00000093388	Gm22655	2.119032431	0.002036487	ENSMUSG00000068587	Mgam	3.418775522	1.53E-50
ENSMUSG00000085501	Gm11772	2.118267231	0.013866629	ENSMUSG00000044176	Spink10	3.38983435	4.13E-19
ENSMUSG00000034981	Parm1	2.115674394	1.66E-245	ENSMUSG00000028976	Slc2a5	3.379417798	0.027889124
ENSMUSG00000005800	Mmp8	2.101657936	1.09E-15	ENSMUSG00000105102	Gm35507	3.316626845	0.032512134
ENSMUSG00000073514	Dok6	2.099659506	2.96E-07	ENSMUSG00000046203	Sprr2g	3.288310172	2.46E-12
ENSMUSG00000021732	Fgf10	2.098598345	1.96E-35	ENSMUSG00000102549	Gm38137	3.244926616	0.000274134
ENSMUSG00000034987	Hrh2	2.092515876	0.0327312	ENSMUSG00000100303	2600014E21Rik	3.228591821	0.00032316
ENSMUSG00000025885	Myo5b	2.076895851	4.05E-05	ENSMUSG00000100843	Gm8307	3.222829094	0.000768286
ENSMUSG00000099803	Gm28863	2.057726025	0.030946103	ENSMUSG00000030187	Klra2	3.215287489	7.15E-12
ENSMUSG00000107549	Gm43961	2.037433405	0.040561916	ENSMUSG00000097411	B430218F22Rik	3.209543873	0.04704561
ENSMUSG00000118100	Gm50340	2.026865839	0.000492757	ENSMUSG00000039099	Wdr93	3.206135502	3.16E-05
ENSMUSG00000036381	P2ry14	2.020050659	8.42E-07	ENSMUSG00000070465	Gm9696	3.190204434	0.039559745
ENSMUSG00000031549	Ido2	2.015465091	0.007229348	ENSMUSG00000051361	6030498E09Rik	3.157274029	4.02E-06
ENSMUSG00000030187	Klra2	1.987900215	0.003289322	ENSMUSG00000079105	C7	3.136325664	1.61E-17
ENSMUSG00000048138	Dmrt2	1.9546788	0.005895012	ENSMUSG00000031289	Il13ra2	3.134485182	5.72E-13
ENSMUSG00000001865	Cpa3	1.944094491	0.03384164	ENSMUSG00000050808	Muc15	3.13117377	6.43E-35
ENSMUSG00000028031	Dkk2	1.880055986	1.11E-19	ENSMUSG00000115759	Gm18787	3.096363027	4.30E-08
ENSMUSG00000044903	Psg22	1.865163518	0.000919186	ENSMUSG00000089943	Ugt1a5	3.094344472	0.016924073
ENSMUSG00000063887	Nlgn1	1.853366294	0.009881691	ENSMUSG00000097186	Gm26595	3.071010341	0.006882693
ENSMUSG00000086447	Gm13522	1.845106319	0.02081258	ENSMUSG00000087659	Gm12606	3.047904317	0.000124509
ENSMUSG00000000730	Dnmt3l	1.839981907	1.48E-16	ENSMUSG00000085781	Gm15640	3.01414717	8.84E-98
ENSMUSG00000031936	Hephl1	1.838433332	7.40E-13	ENSMUSG00000020374	Rasgef1c	2.989734395	2.83E-17
ENSMUSG00000036330	Slc18a1	1.833189595	1.17E-08	ENSMUSG00000079138	Gm8818	2.97418813	7.76E-25
ENSMUSG00000044522	A730020M07Rik	1.820386952	5.86E-05	ENSMUSG00000033854	Kcnk10	2.945879853	2.11E-05
ENSMUSG00000116633	Gm18519	1.819680282	0.024183267	ENSMUSG00000058252	Tcp11x2	2.914862269	4.29E-06
ENSMUSG00000083567	Gm11451	1.808229309	0.043340435	ENSMUSG00000029321	Slc10a6	2.884124026	8.57E-17
ENSMUSG00000043613	Mmp3	1.791774274	0	ENSMUSG00000074183	Gsta1	2.883373785	1.38E-54
ENSMUSG00000021573	Tppp	1.787498028	0.002355262	ENSMUSG00000050069	Grem2	2.847572865	7.36E-19
ENSMUSG00000078496	Zfp982	1.785416924	1.17E-28	ENSMUSG00000044349	Snhg11	2.834199714	2.29E-148
ENSMUSG00000048644	Ctxn1	1.781918692	8.57E-09	ENSMUSG00000042845	Wfdc12	2.803337827	0.016534982
ENSMUSG00000036197	Gxylt1	1.776396512	0	ENSMUSG00000000182	Fgf23	2.802832312	1.39E-23

ENSMUSG00000025503	Taldo1	1.77088653	0
ENSMUSG00000080862	Gm14523	1.759520753	0.001403145
ENSMUSG00000046593	Tmem215	1.754787752	5.84E-21
ENSMUSG00000047330	Kcne4	1.748849874	7.60E-218
ENSMUSG00000000934	Top1mt	1.716617122	1.24E-40
ENSMUSG00000026442	Nfasc	1.704994144	5.59E-08
ENSMUSG00000022206	Npr3	1.703505109	8.90E-230
ENSMUSG00000104861	3110039M20Rik	1.670887827	0.002946018
ENSMUSG00000028417	Tal2	1.64658593	0.046613451
ENSMUSG00000041481	Serpina3g	1.646346989	5.99E-63
ENSMUSG00000021758	Ddx4	1.645482827	0.000863388
ENSMUSG00000019326	Aoc3	1.641917234	7.31E-26
ENSMUSG00000103195	Gm37644	1.606931829	0.048678771
ENSMUSG00000114942	Gm49361	1.605977482	0.020441151
ENSMUSG00000038496	Slc19a3	1.605061836	3.44E-06
ENSMUSG00000066755	Tnfsf18	1.603188755	2.82E-98
ENSMUSG00000049721	Gal3st1	1.597396921	1.37E-08
ENSMUSG00000019647	Sema6a	1.594035839	9.22E-30
ENSMUSG00000049112	Oxtr	1.593056106	0.004542155
ENSMUSG00000022383	Ppara	1.5786611	6.37E-05
ENSMUSG00000035258	Abi3bp	1.577791656	1.48E-16
ENSMUSG00000026822	Lcn2	1.577618658	0.004045713
ENSMUSG00000039701	Usp53	1.576982698	6.94E-117
ENSMUSG00000042659	Arrdc4	1.574355089	1.71E-102
ENSMUSG00000097207	6030443J06Rik	1.572415165	5.75E-05
ENSMUSG00000056427	Slit3	1.567876932	1.98E-38
ENSMUSG00000022321	Cdh10	1.566341646	0.001591577
ENSMUSG00000085058	8030453O22Rik	1.562249494	1.47E-08
ENSMUSG00000020431	Adcy1	1.561474031	6.87E-24
ENSMUSG00000037035	Inhbb	1.549981975	2.80E-282
ENSMUSG00000086320	Gm12840	1.548774126	3.18E-11
ENSMUSG00000044349	Snhg11	1.54722106	1.26E-27
ENSMUSG00000012889	Podnl1	1.54637489	0.034488325
ENSMUSG00000014813	Stc1	1.545737845	1.11E-10
ENSMUSG00000045215	Asxl3	1.544826193	0.039423847
ENSMUSG00000042436	Mfap4	1.544654822	4.03E-33
ENSMUSG00000110588	Gm45774	1.538763292	1.54E-05
ENSMUSG00000103677	Pcdhga4	1.537213129	0.004687171
ENSMUSG00000057522	Spop	1.53080469	7.15E-301
ENSMUSG00000097532	Gm4349	1.527568349	0.031373189
ENSMUSG00000021091	Serpina3n	1.525361814	1.40E-64
ENSMUSG00000111709	Gm3776	1.520325241	0.021712578
ENSMUSG00000054545	Ugt1a6a	1.510817846	1.24E-06
ENSMUSG00000050578	Mmp13	1.505234989	5.76E-12
ENSMUSG00000002489	Tiam1	1.502108176	5.05E-134
ENSMUSG00000058498	Rnf207	1.496546451	0.044676209
ENSMUSG00000031340	Gabre	1.493704719	3.63E-17
ENSMUSG00000039865	Slc44a3	1.492619781	0.000259516
ENSMUSG00000074183	Gsta1	1.492574273	1.03E-08
ENSMUSG00000107478	Gm45234	1.48917071	0.013710658
ENSMUSG00000105194	Gm6755	1.485041111	0.00959292
ENSMUSG00000024186	Rgs11	1.483547735	0.007400568
ENSMUSG00000045294	Insig1	1.47924571	1.63E-191
ENSMUSG00000048916	Gm5540	1.477695384	0.015474508
ENSMUSG00000090145	Ugt1a6b	1.475342578	0.000246124
ENSMUSG00000040606	Kazn	1.473588832	2.86E-79
ENSMUSG00000110676	Gm45897	1.46318968	0.011975931
ENSMUSG00000070304	Scn2b	1.460714963	0.011293194
ENSMUSG00000025105	Bnc1	1.4602949	1.90E-07
ENSMUSG00000042784	Muc1	1.457816265	0.000337572
ENSMUSG00000044788	Fads6	1.457240292	1.48E-32
ENSMUSG00000093424	6330562C20Rik	1.45515137	0.018121042
ENSMUSG00000089744	Gm16146	1.450454095	0.025203923
ENSMUSG00000019966	Kitl	1.44932496	8.89E-180
ENSMUSG00000000340	Dbt	1.447493714	1.49E-68
ENSMUSG00000090564	A430057M04Rik	1.445903231	3.22E-19
ENSMUSG00000040896	Kcnd3	1.444680088	1.09E-05
ENSMUSG00000109864	Eid3	1.444277603	2.55E-06
ENSMUSG00000115240	Gm5144	1.440792336	0.034913398
ENSMUSG00000118097	Gm5237	1.439387302	0.040112297
ENSMUSG00000027832	Ptx3	1.42782761	1.79E-283
ENSMUSG00000032332	Col12a1	1.413712427	8.97E-37
ENSMUSG00000029348	Asphd2	1.412462395	3.04E-07
ENSMUSG00000105981	2810428J06Rik	1.410001606	0.020967725
ENSMUSG00000031853	Map3k21	1.408938704	1.25E-05
ENSMUSG00000042616	Oscp1	1.407535331	7.76E-07
ENSMUSG00000029380	Cxcl1	1.404971758	2.84E-11
ENSMUSG00000117604	Gm33228	2.802150993	4.41E-53
ENSMUSG00000021983	Atp8a2	2.768386897	0.019422267
ENSMUSG00000025746	Il6	2.762648935	0
ENSMUSG00000028128	F3	2.756120256	0
ENSMUSG00000051022	Hs3st1	2.750691074	2.35E-66
ENSMUSG00000107134	Gm42528	2.718451468	9.73E-08
ENSMUSG00000090145	Ugt1a6b	2.711994568	1.35E-13
ENSMUSG00000033508	Asprv1	2.703591562	0.021620801
ENSMUSG00000043263	Ifi209	2.697326693	0.001709211
ENSMUSG00000031613	Hpgd	2.694361616	1.44E-71
ENSMUSG00000086124	A530076I17Rik	2.683041651	0.01274856
ENSMUSG00000068794	Col28a1	2.675555301	3.92E-295
ENSMUSG00000078868	Gm14412	2.658494857	3.86E-06
ENSMUSG00000116755	6430553K19Rik	2.639159643	0.031205227
ENSMUSG00000026185	Igfbp5	2.636324803	8.02E-99
ENSMUSG00000006014	Prg4	2.634771871	3.55E-198
ENSMUSG00000115414	Gm49257	2.632394406	0.001644585
ENSMUSG00000079012	Serpina3m	2.627027296	1.82E-16
ENSMUSG00000027500	Stmn2	2.603170459	0.023217661
ENSMUSG00000029371	Cxcl5	2.602690775	0
ENSMUSG00000086320	Gm12840	2.597794068	1.10E-41
ENSMUSG00000112547	Gm47096	2.591146933	0.000541324
ENSMUSG00000048138	Dmrt2	2.586256899	1.12E-05
ENSMUSG00000045392	Olfr1033	2.571116608	1.45E-25
ENSMUSG00000113548	Gm34047	2.544633853	0.000205504
ENSMUSG00000027408	Cpxm1	2.528112743	1.37E-269
ENSMUSG00000003949	Hlf	2.504707704	2.08E-19
ENSMUSG00000049676	Catsperg1	2.501572441	7.36E-15
ENSMUSG00000021070	Bdkrb2	2.49537312	0.000111412
ENSMUSG00000019966	Kitl	2.488202876	0
ENSMUSG00000050587	Lrrc4c	2.482686719	2.49E-20
ENSMUSG00000074179	Gm10639	2.48170921	0.034223152
ENSMUSG00000109864	Eid3	2.469192781	5.21E-25
ENSMUSG00000097282	5031415H12Rik	2.46755101	0.026120009
ENSMUSG00000025503	Taldo1	2.451326626	0
ENSMUSG00000101625	Gm29371	2.447881176	2.94E-07
ENSMUSG00000034981	Parm1	2.445545826	0
ENSMUSG00000057933	Gsta2	2.442341187	1.98E-26
ENSMUSG00000001865	Cpa3	2.4418313	0.001924964
ENSMUSG00000032348	Gsta4	2.406412755	6.01E-110
ENSMUSG00000104765	Gm43058	2.389101696	0.02277678
ENSMUSG00000027375	Mal	2.372459935	7.47E-85
ENSMUSG00000029882	2210010C04Rik	2.372060412	0.036097031
ENSMUSG00000115801	AC160336.1	2.366273474	0.002107938
ENSMUSG00000020363	Gfpt2	2.362485878	0
ENSMUSG00000036964	Trim17	2.340487652	0.000257101
ENSMUSG00000025889	Snca	2.314646427	0.000744352
ENSMUSG00000044626	Liph	2.304904696	1.25E-05
ENSMUSG00000099803	Gm28863	2.304068104	0.006362125
ENSMUSG00000054598	9130230L23Rik	2.302952257	0.031334904
ENSMUSG00000097254	C430042M11Rik	2.301626776	1.28E-07
ENSMUSG00000030162	Olr1	2.294104665	3.01E-301
ENSMUSG00000073514	Dok6	2.293483618	7.97E-11
ENSMUSG00000049100	Pcdh10	2.289409422	3.32E-05
ENSMUSG00000002565	Scin	2.277112626	2.69E-76
ENSMUSG00000046598	Bdh1	2.275380391	1.59E-24
ENSMUSG00000103195	Gm37644	2.27152206	0.000315075
ENSMUSG00000053175	Bcl3	2.264230278	2.62E-67
ENSMUSG00000035373	Ccl7	2.252860174	5.84E-269
ENSMUSG00000046093	Hpcal4	2.251513245	0.022323264
ENSMUSG00000106447	Gm42957	2.24294367	0.044439493
ENSMUSG00000111818	Gm17749	2.239345545	0.003699792
ENSMUSG00000036437	Npy1r	2.225521314	0.000385857
ENSMUSG00000021732	Fgf10	2.219683935	1.57E-45
ENSMUSG00000044017	Adgrd1	2.208568078	3.22E-35
ENSMUSG00000007682	Dio2	2.180477422	5.61E-24
ENSMUSG00000034755	Pcdh11x	2.179145542	1.56E-15
ENSMUSG00000087439	Gm15788	2.161709138	0.010202069
ENSMUSG00000026888	Grb14	2.158550091	5.72E-121
ENSMUSG00000024008	Cpne5	2.142167543	0.001849072
ENSMUSG00000028354	Fmn2	2.140093291	2.91E-06
ENSMUSG00000028294	Cfap206	2.138957222	0.000580845
ENSMUSG00000005800	Mmp8	2.112872909	5.28E-16
ENSMUSG00000056073	Grik2	2.111074081	1.45E-18
ENSMUSG00000026399	Cd55	2.095060322	0
ENSMUSG00000115099	1700087I21Rik	2.087242333	6.15E-05
ENSMUSG00000019832	Rab32	2.062306825	0

ENSMUSG00000037463	Fbxo27	1.400714707	0.049075707
ENSMUSG00000048905	Bnip5	1.398628595	0.00068314
ENSMUSG00000020123	Avpr1a	1.397452224	1.68E-81
ENSMUSG00000024479	Mal2	1.396172188	0.010825084
ENSMUSG00000002565	Scin	1.392932986	1.36E-20
ENSMUSG00000022367	Has2	1.386871982	1.16E-28
ENSMUSG00000109251	E230032D23Rik	1.383471918	0.004465495
ENSMUSG00000028370	Pappa	1.381871771	2.56E-75
ENSMUSG00000092200	Tnxa	1.379794949	0.000121254
ENSMUSG00000038555	Reep2	1.376493164	8.44E-06
ENSMUSG00000037185	Krt80	1.37418212	1.50E-29
ENSMUSG00000085781	Gm15640	1.37231179	1.61E-08
ENSMUSG00000097124	A530020G20Rik	1.370501714	0.003885765
ENSMUSG00000041958	Pigs	1.369391453	9.56E-180
ENSMUSG00000024164	C3	1.367326722	5.18E-09
ENSMUSG00000027220	Syt13	1.366090828	4.45E-07
ENSMUSG00000052605	Isoc2b	1.361150482	2.06E-06
ENSMUSG00000025892	Gria4	1.360451275	1.73E-49
ENSMUSG00000029321	Slc10a6	1.354586233	0.009943366
ENSMUSG00000034258	Flvcr2	1.350406704	8.96E-06
ENSMUSG00000067855	Speer3	1.350036432	0.002215659
ENSMUSG00000021904	Sema3g	1.346121821	0.024328323
ENSMUSG00000028420	Tmem38b	1.345371794	5.48E-86
ENSMUSG00000050989	Selenon	1.333931882	8.09E-234
ENSMUSG00000048376	F2r	1.333353318	0
ENSMUSG00000067356	B430203G13Rik	1.331439939	0.020158553
ENSMUSG00000041945	Mfsd9	1.328162553	5.05E-19
ENSMUSG00000025746	Il6	1.326798873	4.88E-111
ENSMUSG00000021478	Drd1	1.323333424	0.001624052
ENSMUSG00000051022	Hs3st1	1.323277328	9.17E-09
ENSMUSG00000020427	Igfbp3	1.317300104	7.70E-152
ENSMUSG00000084289	Gm6977	1.31533447	0.010305455
ENSMUSG00000030340	Scnn1a	1.309693184	0.001404899
ENSMUSG00000037104	Socs5	1.308733152	0
ENSMUSG00000047810	Ccdc88b	1.295993763	1.05E-07
ENSMUSG00000052026	Slc6a7	1.295255948	0.006655866
ENSMUSG00000050545	Fam228b	1.294921147	0.036531039
ENSMUSG00000042581	Thsd7b	1.294864127	9.06E-09
ENSMUSG00000006345	Ggt1	1.283360918	0.002000295
ENSMUSG00000115785	Gm6740	1.279753688	1.29E-06
ENSMUSG00000073433	Arhgdig	1.278893212	0.002002403
ENSMUSG00000068587	Mgam	1.278791381	9.38E-05
ENSMUSG00000046589	Lrrc8e	1.274037689	1.98E-07
ENSMUSG00000043811	Rtn4r	1.269642192	6.11E-19
ENSMUSG00000028583	Pdpn	1.264351641	1.02E-298
ENSMUSG00000078838	Gm17382	1.263502063	0.029372516
ENSMUSG00000030623	Prss23os	1.262655756	6.93E-07
ENSMUSG00000026888	Grb14	1.260809202	1.47E-33
ENSMUSG00000108228	6430584L05Rik	1.258611213	2.41E-06
ENSMUSG00000003617	Cp	1.258539753	9.64E-256
ENSMUSG00000067365	Tmem128	1.257650312	1.91E-113
ENSMUSG00000072653	Zfp783	1.254117172	0.001298278
ENSMUSG00000032420	Nt5e	1.251693476	7.57E-40
ENSMUSG00000075000	Nrbf2	1.249309535	2.35E-44
ENSMUSG00000034993	Vat1	1.241790406	0
ENSMUSG00000037169	Mycn	1.234067696	3.20E-64
ENSMUSG00000037428	Vgf	1.229714578	3.88E-06
ENSMUSG00000023826	Prkn	1.22559994	0.043846581
ENSMUSG00000097675	1700101I11Rik	1.22313169	0.047279913
ENSMUSG00000034936	Arl4d	1.22174646	4.99E-06
ENSMUSG00000025933	Tmem14a	1.221567858	1.15E-07
ENSMUSG00000011256	Adam19	1.221332893	2.94E-284
ENSMUSG00000094786	Gm14403	1.220942543	0.000725864
ENSMUSG00000106426	Gm36211	1.21480533	0.00578375
ENSMUSG00000016552	Foxred2	1.213323656	8.40E-07
ENSMUSG00000046959	Slc26a1	1.20696692	0.00166518
ENSMUSG00000027208	Fgf7	1.20607282	3.20E-57
ENSMUSG00000050808	Muc15	1.205536215	0.002800399
ENSMUSG00000020473	Aebp1	1.202929228	0
ENSMUSG00000071650	Ganab	1.199732356	1.16E-280
ENSMUSG00000091784	Gm17022	1.199725096	0.039130363
ENSMUSG00000045392	Olfr1033	1.196091263	0.000273732
ENSMUSG00000105211	Gm47302	1.19062473	0.022820779
ENSMUSG00000039005	Tlr4	1.189960791	4.56E-109
ENSMUSG00000056501	Cebpb	1.189318316	2.99E-87
ENSMUSG00000118383	Gm50321	1.187685217	2.82E-19
ENSMUSG00000030407	Qpctl	1.184366688	1.02E-44
ENSMUSG00000070687	Htr1d	2.051544765	0.003090351
ENSMUSG00000032420	Nt5e	2.050692684	2.03E-136
ENSMUSG00000030077	Chl1	2.042614272	0.048752601
ENSMUSG00000021091	Serpina3n	2.041340325	0
ENSMUSG00000104801	Gm43834	2.040926045	0.047787513
ENSMUSG00000095054	Gm13146	2.040125826	0.015211499
ENSMUSG00000026442	Nfasc	2.026433043	1.19E-13
ENSMUSG00000026401	Cd55b	2.016350938	5.95E-11
ENSMUSG00000073761	4933427I04Rik	2.015312119	0.018916835
ENSMUSG00000029378	Areg	2.004365497	1.27E-05
ENSMUSG00000050395	Tnfsf15	1.99128558	2.66E-237
ENSMUSG00000032878	Ccdc85a	1.989749014	1.19E-08
ENSMUSG00000057000	Nxf3	1.986284745	8.38E-11
ENSMUSG00000027832	Ptx3	1.983760954	0
ENSMUSG00000109517	Gm44763	1.980599751	1.53E-05
ENSMUSG00000045294	Insig1	1.980380765	4.31E-232
ENSMUSG00000096052	9930004E17Rik	1.980144608	0.00108967
ENSMUSG00000024331	Dsc2	1.977126704	7.69E-06
ENSMUSG00000096233	Gm13238	1.975650034	9.94E-05
ENSMUSG00000056296	Synpr	1.961006507	2.33E-08
ENSMUSG00000027360	Hdc	1.96003375	3.86E-08
ENSMUSG00000114452	A530001N23Rik	1.95681854	0.002982038
ENSMUSG00000097166	9330179D12Rik	1.956524767	0.000589913
ENSMUSG00000022861	Dgkg	1.952446496	3.53E-06
ENSMUSG00000043613	Mmp3	1.951162258	2.95E-102
ENSMUSG00000019880	Rspo3	1.946735811	2.01E-08
ENSMUSG00000054545	Ugt1a6a	1.935886557	3.44E-13
ENSMUSG00000022122	Ednrb	1.920187349	1.30E-39
ENSMUSG00000021758	Ddx4	1.91210962	1.72E-05
ENSMUSG00000062342	Serpinb9e	1.910488537	0.002796872
ENSMUSG00000056270	Prr9	1.905790231	0.032946439
ENSMUSG00000026114	Cnga3	1.899615588	0.001693536
ENSMUSG00000040569	Slc26a7	1.895038311	0.001658879
ENSMUSG00000028362	Tnfsf8	1.889674402	6.99E-06
ENSMUSG00000061039	Olfr920	1.887873697	0.002183268
ENSMUSG00000035513	Ntng2	1.887002413	5.48E-23
ENSMUSG00000034810	Scn7a	1.883171437	2.57E-218
ENSMUSG00000026971	Itgb6	1.882980149	4.96E-16
ENSMUSG00000042659	Arrdc4	1.877348184	7.67E-135
ENSMUSG00000086040	Wipf3	1.877252457	3.55E-10
ENSMUSG00000094786	Gm14403	1.873157217	4.38E-12
ENSMUSG00000018500	Adora2b	1.858265339	1.89E-168
ENSMUSG00000016349	Eef1a2	1.85739548	8.73E-35
ENSMUSG00000000730	Dnmt3l	1.848361957	7.23E-16
ENSMUSG00000033453	Adamts15	1.843926353	0.000207766
ENSMUSG00000113985	9130015A21Rik	1.831202369	1.17E-13
ENSMUSG00000044734	Serpinb1a	1.828074516	6.86E-176
ENSMUSG00000025321	Itgb8	1.824803726	2.33E-159
ENSMUSG00000037996	Slc24a2	1.823099773	0.011890424
ENSMUSG00000028974	Dffa	1.822232862	3.25E-129
ENSMUSG00000025037	Maoa	1.818227537	0
ENSMUSG00000071713	Csf2rb	1.817577559	3.25E-07
ENSMUSG00000021214	Akr1c18	1.813818391	8.75E-05
ENSMUSG00000010797	Wnt2	1.80531665	1.21E-06
ENSMUSG00000044788	Fads6	1.80385308	9.11E-58
ENSMUSG00000042717	Ppp1r3a	1.798200961	1.15E-09
ENSMUSG00000002459	Rgs20	1.796971308	0.020705349
ENSMUSG00000092274	Neat1	1.791100126	1.49E-277
ENSMUSG00000014813	Stc1	1.786278343	4.92E-15
ENSMUSG00000050721	Plekho2	1.785579414	0
ENSMUSG00000090628	Gm17083	1.768601949	0.026010738
ENSMUSG00000089940	Gm4117	1.762905173	3.15E-06
ENSMUSG00000106457	Gm42585	1.754239094	0.016845133
ENSMUSG00000025784	Clec3b	1.753341701	0.000535957
ENSMUSG00000028341	Nr4a3	1.750723383	1.70E-70
ENSMUSG00000043085	Tmem82	1.743435295	2.78E-06
ENSMUSG00000115338	Pnp	1.741776905	2.00E-222
ENSMUSG00000035258	Abi3bp	1.737766956	1.97E-25
ENSMUSG00000038259	Gdf5	1.737511353	0.011220399
ENSMUSG00000050447	Lypd6	1.731471909	0.035593893
ENSMUSG00000048905	Bnip5	1.725990985	9.18E-06
ENSMUSG00000038071	Npy6r	1.722644731	4.74E-07
ENSMUSG00000022479	Vdr	1.709560676	3.12E-24
ENSMUSG00000061654	Spry3	1.701178883	2.06E-09
ENSMUSG00000105651	1700017M07Rik	1.689054193	0.0033526
ENSMUSG00000045362	Tnfrsf26	1.685680608	1.45E-300
ENSMUSG00000086358	Gm13270	1.680146868	0.007641468

ENSMUSG00000045362	Tnfrsf26	1.181396258	9.84E-175	ENSMUSG00000106205	C230096K16Rik	1.674275256	0.00029079
ENSMUSG00000091514	Gm17484	1.178209527	0.006156678	ENSMUSG00000020806	Rhbdf2	1.670639923	4.64E-165
ENSMUSG00000033327	Tnxb	1.178127537	6.22E-69	ENSMUSG00000100213	Gm28151	1.668050815	4.00E-05
ENSMUSG00000031762	Mt2	1.177717639	9.87E-85	ENSMUSG00000110588	Gm45774	1.666328215	1.09E-06
ENSMUSG00000108461	AV356131	1.177232267	0.005317327	ENSMUSG00000050578	Mmp13	1.665907969	6.62E-18
ENSMUSG00000028780	Sema3c	1.176288722	5.59E-74	ENSMUSG00000104667	Gm4961	1.661496429	1.71E-14
ENSMUSG00000022514	Il1rap	1.175074142	3.79E-264	ENSMUSG00000055301	Adh7	1.655836441	2.17E-13
ENSMUSG00000034810	Scn7a	1.174574616	5.40E-84	ENSMUSG00000047822	Angptl8	1.655017127	0.027189358
ENSMUSG00000050860	Phospho1	1.172753987	6.70E-09	ENSMUSG00000085058	8030453O22Rik	1.65469363	2.63E-10
ENSMUSG00000022800	Fyttd1	1.172699077	2.10E-132	ENSMUSG00000079014	Serpina3i	1.651919346	9.36E-16
ENSMUSG00000015536	Mocs2	1.167995879	6.35E-46	ENSMUSG00000047330	Kcne4	1.649480367	7.32E-172
ENSMUSG00000087684	1200007C13Rik	1.165971858	0.001579406	ENSMUSG00000037463	Fbxo27	1.648289673	0.006039515
ENSMUSG00000099041	Gm28035	1.16167088	0.01150341	ENSMUSG00000031762	Mt2	1.644995883	8.28E-227
ENSMUSG00000021575	Ahrr	1.160947826	7.85E-11	ENSMUSG00000097305	Gm17276	1.644205522	0.0139729
ENSMUSG00000055480	Zfp458	1.157324581	2.44E-09	ENSMUSG00000097378	B230208H11Rik	1.644140166	0.045684683
ENSMUSG00000040663	Clcf1	1.154952369	2.39E-55	ENSMUSG00000038415	Foxq1	1.642881457	9.09E-05
ENSMUSG00000108892	Gm9449	1.154807693	0.041787454	ENSMUSG00000015829	Tnr	1.637821539	0.042634374
ENSMUSG00000054280	Prr14l	1.153494816	6.40E-51	ENSMUSG00000106874	Gm20186	1.637523674	1.78E-40
ENSMUSG00000050721	Plekho2	1.145289871	1.62E-205	ENSMUSG00000041695	Kcnj2	1.637134295	4.29E-05
ENSMUSG00000004885	Crabp2	1.143502083	0.001207746	ENSMUSG00000112963	Gm6093	1.631569054	3.57E-06
ENSMUSG00000092274	Neat1	1.137445745	1.50E-108	ENSMUSG00000031340	Gabre	1.630444264	2.36E-20
ENSMUSG00000032625	Thsd7a	1.136202968	2.91E-96	ENSMUSG00000026692	Fmo4	1.626462469	0.023131849
ENSMUSG00000089940	Gm4117	1.135380734	0.021244931	ENSMUSG00000003665	Has1	1.626296271	1.28E-14
ENSMUSG00000115338	Pnp	1.133611449	3.36E-14	ENSMUSG00000078866	Zfp970	1.616474629	7.61E-40
ENSMUSG00000090793	Gm6650	1.132199337	0.034786384	ENSMUSG00000024479	Mal2	1.610652147	0.001264923
ENSMUSG00000006014	Prg4	1.131912591	7.14E-24	ENSMUSG00000056501	Cebpb	1.608298815	5.64E-181
ENSMUSG00000041842	Fhdc1	1.129060111	2.24E-145	ENSMUSG00000015533	Itga2	1.607606425	8.26E-42
ENSMUSG00000028124	Gclm	1.125721632	6.70E-125	ENSMUSG00000115785	Gm6740	1.606300041	1.83E-11
ENSMUSG00000007564	Ppp2r1a	1.12459635	4.13E-159	ENSMUSG00000110676	Gm45897	1.605925969	0.003873977
ENSMUSG00000028766	Alpl	1.122985588	4.17E-12	ENSMUSG00000008540	Mgst1	1.605409368	2.06E-235
ENSMUSG00000073478	D730003I15Rik	1.119354746	0.009710681	ENSMUSG00000097675	1700101I11Rik	1.602860654	0.000459351
ENSMUSG00000024360	Etf1	1.118220393	2.72E-281	ENSMUSG00000024381	Bin1	1.599645993	1.44E-251
ENSMUSG00000019880	Rspo3	1.116541478	0.023192915	ENSMUSG00000039982	Dtx4	1.59481429	6.83E-191
ENSMUSG00000074071	Fam169b	1.115511952	0.001594389	ENSMUSG00000023826	Prkn	1.589289766	0.001518608
ENSMUSG00000002997	Prkar2b	1.114658473	5.38E-247	ENSMUSG00000098009	Gm5597	1.580115715	0.000182364
ENSMUSG00000046169	Adamts6	1.113716035	3.79E-39	ENSMUSG00000000934	Top1mt	1.579351864	8.87E-27
ENSMUSG00000038508	Gdf15	1.113173254	7.27E-20	ENSMUSG00000037033	Clca3b	1.574667178	0.006331599
ENSMUSG00000064105	Cnnm2	1.112110071	6.91E-13	ENSMUSG00000046589	Lrrc8e	1.573537286	1.45E-11
ENSMUSG00000041912	Tdrkh	1.111118043	1.40E-21	ENSMUSG00000024897	Apba1	1.562420498	1.99E-54
ENSMUSG00000107810	Gm18609	1.109803085	0.008856127	ENSMUSG00000070343	Gm10288	1.561343439	0.013969808
ENSMUSG00000052392	Acot4	1.109447123	0.003922593	ENSMUSG00000080862	Gm14523	1.56047668	0.009264708
ENSMUSG00000112963	Gm6093	1.108766385	0.003306153	ENSMUSG00000002289	Angptl4	1.544983486	1.89E-248
ENSMUSG00000060961	Slc4a4	1.10546929	1.58E-38	ENSMUSG00000058624	Gda	1.544677602	1.43E-239
ENSMUSG00000002289	Angptl4	1.105004665	1.36E-101	ENSMUSG00000038058	Nod1	1.544071454	6.01E-257
ENSMUSG00000070323	Mmp27	1.10326201	1.73E-08	ENSMUSG00000025194	Abcc2	1.538213957	9.02E-06
ENSMUSG00000053117	E330013P04Rik	1.101834125	0.004531649	ENSMUSG00000028583	Pdpn	1.534346187	0
ENSMUSG00000072294	Klf12	1.099887984	1.36E-13	ENSMUSG00000048706	Lurap1l	1.532725974	1.34E-29
ENSMUSG00000038276	Asic3	1.099252033	0.008469344	ENSMUSG00000079055	Slc8a3	1.53265443	0.006802139
ENSMUSG00000024529	Lox	1.096829565	2.91E-257	ENSMUSG00000042581	Thsd7b	1.532255743	1.51E-12
ENSMUSG00000041605	Inava	1.095503924	0.000120695	ENSMUSG00000109251	E230032D23Rik	1.531167956	0.000667497
ENSMUSG00000039982	Dtx4	1.095314863	5.18E-90	ENSMUSG00000105194	Gm6755	1.529085444	0.006678035
ENSMUSG00000029778	Adcyap1r1	1.08982107	8.89E-181	ENSMUSG00000106339	Gm43489	1.529012614	1.97E-06
ENSMUSG00000049128	Ivl	1.086797726	3.27E-18	ENSMUSG00000021687	Scamp1	1.526507889	2.40E-211
ENSMUSG00000075254	Heg1	1.085080918	1.29E-129	ENSMUSG00000052605	Isoc2b	1.518644701	2.07E-08
ENSMUSG00000048706	Lurap1l	1.084843064	3.48E-14	ENSMUSG00000075254	Heg1	1.515788965	7.03E-271
ENSMUSG00000021190	Lgmn	1.084735815	3.44E-173	ENSMUSG00000086914	Gm16124	1.508635658	0.009738458
ENSMUSG00000037362	Ccn3	1.083882583	4.19E-53	ENSMUSG00000032845	Alpk2	1.504022844	7.24E-05
ENSMUSG00000001910	Nacc1	1.083678391	1.33E-115	ENSMUSG00000089698	Gm2541	1.502013958	4.76E-05
ENSMUSG00000067916	Zfp991	1.079021644	1.46E-53	ENSMUSG00000028373	Astn2	1.501580659	0.000441201
ENSMUSG00000028974	Dffa	1.074409861	1.12E-30	ENSMUSG00000017400	Stac2	1.496374084	0.002968137
ENSMUSG00000054640	Slc8a1	1.073498848	4.13E-54	ENSMUSG00000066755	Tnfsf18	1.49034002	1.83E-80
ENSMUSG00000033361	Prrg3	1.072666091	3.68E-55	ENSMUSG00000052125	F730043M19Rik	1.48432481	2.15E-57
ENSMUSG00000029844	Hoxa1	1.072269995	0.000447915	ENSMUSG00000041481	Serpina3g	1.482947871	7.90E-56
ENSMUSG00000035000	Dpp4	1.069593998	2.86E-12	ENSMUSG00000052180	Serpinb6c	1.482432888	1.47E-09
ENSMUSG00000017929	B4galt5	1.068987293	1.92E-165	ENSMUSG00000037622	Wdtc1	1.479729977	9.14E-82
ENSMUSG00000007594	Hapln4	1.063511207	0.000472245	ENSMUSG00000048647	Exd1	1.475939888	1.98E-06
ENSMUSG00000048826	Dact2	1.060642764	0.001267206	ENSMUSG00000075000	Nrbf2	1.474010895	1.74E-69
ENSMUSG00000028654	Mycl	1.05921929	0.019599136	ENSMUSG00000080888	Gm14387	1.473276023	0.016748637
ENSMUSG00000063245	Zfp993	1.055283659	8.94E-15	ENSMUSG00000024360	Etf1	1.472645398	0
ENSMUSG00000113425	Gm48653	1.05434445	0.001146993	ENSMUSG00000030854	Ptpn5	1.472257917	0.038560478
ENSMUSG00000026701	Prdx6	1.052258897	3.71E-170	ENSMUSG00000104861	3110039M20Rik	1.468652168	0.004254618
ENSMUSG00000022376	Adcy8	1.050876794	3.58E-12	ENSMUSG00000040183	Ankrd6	1.467897141	7.60E-18
ENSMUSG00000036242	Armh4	1.047682532	2.02E-08	ENSMUSG00000063458	Lrmda	1.465006949	8.34E-09
ENSMUSG00000024661	Fth1	1.04462558	2.42E-290	ENSMUSG00000045625	Pigz	1.46418057	0.046694287
ENSMUSG00000030022	Adamts9	1.044518876	5.36E-48	ENSMUSG00000048142	Nat8l	1.462452217	8.21E-05
ENSMUSG00000041482	Piezo2	1.043955286	6.18E-37	ENSMUSG00000020121	Srgap1	1.46066263	2.78E-80
ENSMUSG00000055435	Maf	1.0411931	4.53E-13	ENSMUSG00000078439	Smim24	1.460595995	0.002759254

ENSMUSG00000008540	Mgst1	1.036731758	1.04E-98	ENSMUSG00000033538	Casp4	1.455637862	1.87E-72
ENSMUSG00000079259	Trim71	1.035284332	0.041152404	ENSMUSG00000019787	Trdn	1.455370586	0.00823317
ENSMUSG00000103313	Gm38357	1.035050558	0.010563301	ENSMUSG00000030433	Sbk2	1.455280537	0.026525216
ENSMUSG00000000182	Fgf23	1.034729154	0.048325325	ENSMUSG00000006262	Mob1b	1.454204767	1.63E-163
ENSMUSG00000006241	Ccdc159	1.032077189	0.007153533	ENSMUSG00000001622	Csn3	1.450475364	0.016387076
ENSMUSG00000074634	Tmem267	1.030497742	5.85E-14	ENSMUSG00000026043	Col3a1	1.446323393	0
ENSMUSG00000024806	Mlana	1.028568808	3.03E-06	ENSMUSG00000026360	Rgs2	1.44463772	1.62E-30
ENSMUSG00000079138	Gm8818	1.025939321	0.044263256	ENSMUSG00000028670	Lypla2	1.440700445	1.95E-76
ENSMUSG00000048264	Dip2c	1.025265446	6.09E-57	ENSMUSG00000024053	Emilin2	1.44061236	3.89E-105
ENSMUSG00000106825	2510016D11Rik	1.025073193	0.000292522	ENSMUSG00000067219	Nipal1	1.439650805	1.99E-82
ENSMUSG00000110605	Gm32856	1.023253663	0.000188713	ENSMUSG00000002489	Tiam1	1.436303476	3.16E-151
ENSMUSG00000003534	Ddr1	1.021706015	3.05E-268	ENSMUSG00000021098	4930447C04Rik	1.436166328	0.000839123
ENSMUSG00000048731	Ggnbp1	1.020573776	0.045122145	ENSMUSG00000029380	Cxcl1	1.431861782	3.27E-14
ENSMUSG00000031608	Galnt7	1.019098993	4.89E-62	ENSMUSG00000060402	Chst8	1.431195911	0.002432662
ENSMUSG00000036334	Igsf10	1.0187665	7.55E-21	ENSMUSG00000032802	Srxn1	1.43104245	0
ENSMUSG00000022621	Rabl2	1.017366371	3.87E-11	ENSMUSG00000056413	Adap1	1.427698065	0.000136475
ENSMUSG00000073988	Ttpa	1.016982735	0.017139761	ENSMUSG00000083355	Gm11581	1.426343757	0.001217855
ENSMUSG00000041439	Mfsd6	1.016674973	1.36E-34	ENSMUSG00000033377	Palmd	1.425861698	0.000225743
ENSMUSG00000079215	Zfp664	1.015484385	2.01E-120	ENSMUSG00000081378	Rps13-ps4	1.424900068	0.047118836
ENSMUSG00000060733	Ipmk	1.010059886	8.29E-45	ENSMUSG00000024066	Xdh	1.424851671	6.93E-209
ENSMUSG00000006462	A530013C23Rik	1.008358019	0.026624219	ENSMUSG00000025885	Myo5b	1.41970796	0.035527671
ENSMUSG00000106339	Gm43489	1.006801858	0.012345932	ENSMUSG00000022621	Rabl2	1.418994538	2.73E-20
ENSMUSG00000016024	Lbp	1.003155306	1.03E-14	ENSMUSG00000037362	Ccn3	1.417806853	5.49E-74
ENSMUSG00000022449	Adamts20	1.003154439	0.013885481	ENSMUSG00000031400	G6pdx	1.416680886	0
ENSMUSG00000029072	Tas1r3	1.00284107	0.010381292	ENSMUSG00000031377	Bmx	1.416189494	1.25E-33
ENSMUSG00000021831	Ero1l	1.0003331	1.81E-76	ENSMUSG00000032776	Mctp2	1.414792179	2.89E-11
ENSMUSG00000021485	Mxd3	-1.000089658	1.62E-10	ENSMUSG00000071637	Cebpd	1.413523629	3.99E-177
ENSMUSG00000022235	Cmbl	-1.000137383	1.43E-10	ENSMUSG00000020334	Slc22a4	1.41318315	1.54E-06
ENSMUSG00000029861	Fam131b	-1.000265004	9.38E-06	ENSMUSG00000017390	Aldoc	1.400976461	2.47E-09
ENSMUSG00000028445	Enho	-1.001046813	0.000288102	ENSMUSG00000044026	Slc35g1	1.395327335	7.97E-35
ENSMUSG00000118423	Lrrc70	-1.00118252	0.005100565	ENSMUSG00000021996	Esd	1.391008401	0
ENSMUSG00000037206	Islr	-1.002341489	5.99E-37	ENSMUSG00000049422	Chchd10	1.386523223	6.72E-06
ENSMUSG00000020340	Cyfip2	-1.003368288	0.020637838	ENSMUSG00000038555	Reep2	1.386399623	3.53E-06
ENSMUSG00000078762	Haus5	-1.003702551	3.79E-08	ENSMUSG00000025950	Idh1	1.38382618	0
ENSMUSG00000045827	Serpinb9	-1.004251281	5.97E-76	ENSMUSG00000078566	Bnip3	1.380173533	1.41E-76
ENSMUSG00000087060	Eldr	-1.005901541	0.008062957	ENSMUSG00000034762	Glis1	1.379730488	0.001882794
ENSMUSG00000040350	Trim7	-1.006068918	6.42E-12	ENSMUSG00000030160	Tmem52b	1.379355484	0.034168689
ENSMUSG00000002257	Def6	-1.008476267	5.05E-16	ENSMUSG00000056749	Nfil3	1.373723425	5.31E-74
ENSMUSG00000020155	Kcnmb1	-1.01099615	0.004195273	ENSMUSG00000108228	6430584L05Rik	1.373123003	1.83E-07
ENSMUSG00000003134	Tbc1d8	-1.011375133	2.76E-05	ENSMUSG00000028420	Tmem38b	1.37211282	1.92E-84
ENSMUSG00000038984	Tspyl5	-1.01330348	9.14E-06	ENSMUSG00000031438	Rnf128	1.371379287	8.14E-96
ENSMUSG00000005107	Slc2a9	-1.013554353	2.32E-11	ENSMUSG00000039616	Mocos	1.369687844	6.00E-52
ENSMUSG00000039716	Dock3	-1.014183171	1.65E-05	ENSMUSG00000007564	Ppp2r1a	1.368967978	5.27E-209
ENSMUSG00000020303	Stc2	-1.016749599	8.39E-30	ENSMUSG00000043342	Hoxd9	1.362859015	0.019702925
ENSMUSG00000013033	Adgrl1	-1.018254992	4.50E-62	ENSMUSG00000069727	Zfp975	1.361222973	4.89E-19
ENSMUSG00000001270	Ckb	-1.018409079	1.81E-136	ENSMUSG00000074794	Arrdc3	1.356474617	2.55E-111
ENSMUSG00000032122	Slc37a2	-1.019362363	6.00E-14	ENSMUSG00000073565	Prr16	1.355822428	8.76E-05
ENSMUSG00000024190	Dusp1	-1.023304862	2.77E-75	ENSMUSG00000025888	Casp1	1.355703687	0.003960251
ENSMUSG00000053007	Creb5	-1.025293218	9.29E-16	ENSMUSG00000035863	Palm	1.353608935	4.28E-183
ENSMUSG00000036023	Parp2	-1.027267464	2.00E-25	ENSMUSG00000033213	AA467197	1.349724805	5.34E-10
ENSMUSG00000032009	Sesn3	-1.027270524	7.99E-52	ENSMUSG00000030553	Pgpep1l	1.347522111	3.11E-08
ENSMUSG00000017314	Mpp2	-1.027550716	1.46E-23	ENSMUSG00000030203	Dusp16	1.34689286	6.25E-103
ENSMUSG00000030616	Sytl2	-1.027673873	7.89E-30	ENSMUSG00000042616	Oscp1	1.345941536	6.82E-07
ENSMUSG00000019990	Pde7b	-1.027902171	7.86E-20	ENSMUSG00000040606	Kazn	1.341169819	1.79E-59
ENSMUSG00000042978	Sbk1	-1.028517526	3.78E-12	ENSMUSG00000006490	Prl8a9	1.339180571	0.006491362
ENSMUSG00000021990	Spata13	-1.034497069	6.14E-23	ENSMUSG00000022790	Igsf11	1.338382422	1.33E-19
ENSMUSG00000005410	Mcm5	-1.034540449	1.61E-71	ENSMUSG00000050989	Selenon	1.3377952	4.78E-227
ENSMUSG00000027349	Fam98b	-1.035215759	1.04E-57	ENSMUSG00000042607	Asb4	1.337060841	2.72E-14
ENSMUSG00000037649	H2-DMa	-1.036031698	4.28E-07	ENSMUSG00000050697	Prkaa1	1.334083861	3.63E-192
ENSMUSG00000079499	6530402F18Rik	-1.036103738	5.79E-17	ENSMUSG00000032179	Bmp5	1.334045844	0.011163652
ENSMUSG00000031066	Usp11	-1.037681446	9.45E-06	ENSMUSG00000057522	Spop	1.333549405	2.87E-212
ENSMUSG00000038816	Ctnnal1	-1.038197084	2.47E-43	ENSMUSG00000105211	Gm47302	1.333065535	0.005171649
ENSMUSG00000054408	Spcs3	-1.038797993	2.71E-304	ENSMUSG00000006273	Atp6v1b2	1.326331386	6.29E-232
ENSMUSG00000020150	Gamt	-1.039241796	2.49E-12	ENSMUSG00000078838	Gm17382	1.325833561	0.016697019
ENSMUSG00000042444	Mindy2	-1.040070947	3.54E-76	ENSMUSG00000048644	Ctxn1	1.325683352	0.00011789
ENSMUSG00000074968	Ano3	-1.041706943	2.14E-43	ENSMUSG00000005803	Sqor	1.32525766	4.33E-91
ENSMUSG00000099032	Tcf24	-1.042674069	0.000676193	ENSMUSG00000039701	Usp53	1.324355791	3.40E-66
ENSMUSG00000030541	Idh2	-1.042919723	7.25E-55	ENSMUSG00000047347	Tdg-ps	1.321631053	0.039026219
ENSMUSG00000051920	Rspo2	-1.043686706	0.03894788	ENSMUSG00000086695	Gm15247	1.319613495	0.042955518
ENSMUSG00000038007	Acer2	-1.045023483	2.93E-29	ENSMUSG00000037104	Socs5	1.318553196	6.42E-264
ENSMUSG00000041907	Gpr45	-1.045909559	0.028288009	ENSMUSG00000015536	Mocs2	1.315223635	1.98E-57
ENSMUSG00000108350	Gm44950	-1.047945942	1.48E-05	ENSMUSG00000039956	Mrap	1.312809784	1.49E-13
ENSMUSG00000020785	Camkk1	-1.053064059	4.35E-14	ENSMUSG00000054280	Prr14l	1.310520296	3.54E-75
ENSMUSG00000032174	Icam5	-1.054135754	0.037129062	ENSMUSG00000062168	Ppef1	1.307480023	8.64E-10
ENSMUSG00000061887	Ssbp3	-1.055365986	4.31E-49	ENSMUSG00000027797	Dclk1	1.306739801	1.13E-260
ENSMUSG00000079553	Kifc1	-1.056042178	5.07E-44	ENSMUSG00000029072	Tas1r3	1.303476917	9.58E-05
ENSMUSG00000074355	Gm10676	-1.057128403	0.006497131	ENSMUSG00000047022	Mipol1	1.299340118	9.23E-13

ENSMUSG00000025789	St8sia2	-1.058253606	0.000436473	ENSMUSG00000073147	5031425E22Rik	1.298791722	3.78E-91
ENSMUSG00000027919	Lce1g	-1.059677825	0.003931918	ENSMUSG00000024042	Sik1	1.293460924	2.55E-47
ENSMUSG00000021379	Id4	-1.060127425	5.02E-13	ENSMUSG00000006517	Mvd	1.293367452	3.96E-53
ENSMUSG00000020357	Flt4	-1.064068182	4.69E-58	ENSMUSG00000071226	Cecr2	1.293251572	0.022741733
ENSMUSG00000090272	Mndal	-1.06442454	0.004885841	ENSMUSG00000097276	4930525G20Rik	1.292640519	0.033888728
ENSMUSG00000032221	Mns1	-1.064864667	6.86E-17	ENSMUSG00000044337	Ackr3	1.291933883	2.83E-13
ENSMUSG00000037010	Apln	-1.065457497	8.72E-46	ENSMUSG00000059588	Calcrl	1.291229431	7.87E-69
ENSMUSG00000050671	Ism2	-1.065801172	0.025179876	ENSMUSG00000072769	Gm10419	1.29037008	0.028506649
ENSMUSG00000001604	Tcea3	-1.066005477	3.24E-13	ENSMUSG00000041439	Mfsd6	1.288103314	1.03E-60
ENSMUSG00000021391	Cenpp	-1.067910797	4.60E-06	ENSMUSG00000051435	Fhad1	1.287983099	0.020577848
ENSMUSG00000023206	Il15ra	-1.067937679	1.85E-06	ENSMUSG00000022223	Sdr39u1	1.2875832	4.50E-11
ENSMUSG00000097430	Gm10544	-1.069399642	0.017321154	ENSMUSG00000026473	Glul	1.283876478	1.36E-217
ENSMUSG00000052143	Gm9869	-1.071280694	0.000263474	ENSMUSG00000040855	Reps2	1.281749079	7.33E-13
ENSMUSG00000038011	Dnah10	-1.073054009	0.025319522	ENSMUSG00000118502	AL645861.1	1.280362426	0.008245515
ENSMUSG00000059824	Dbp	-1.074365314	3.36E-09	ENSMUSG00000082361	Btc	1.279365682	1.26E-12
ENSMUSG00000025240	Sacm1l	-1.077153278	7.44E-116	ENSMUSG00000041135	Ripk2	1.278114968	3.42E-188
ENSMUSG00000028873	Cdca8	-1.079092914	9.56E-74	ENSMUSG00000004885	Crabp2	1.275135923	0.000639096
ENSMUSG00000021811	Dnajc9	-1.079896957	1.58E-59	ENSMUSG00000023046	Igfbp6	1.27499388	7.69E-24
ENSMUSG00000074738	Fndc10	-1.081900748	1.63E-06	ENSMUSG00000028751	Pla2g2e	1.27410886	3.23E-08
ENSMUSG00000105940	Gm42635	-1.0821068	0.008587962	ENSMUSG00000039629	Strip2	1.27145023	1.19E-11
ENSMUSG00000026414	Tnnt2	-1.082820879	3.85E-78	ENSMUSG00000015134	Aldh1a3	1.267676974	3.48E-51
ENSMUSG00000011263	Exoc3l2	-1.083266352	0.022282702	ENSMUSG00000027792	Bche	1.266217017	4.11E-09
ENSMUSG00000110185	Igip	-1.083306013	1.77E-25	ENSMUSG00000035506	Slc12a8	1.265053628	3.22E-06
ENSMUSG00000029096	Htra3	-1.083974619	1.50E-21	ENSMUSG00000047731	Wbp1l	1.264234941	6.43E-246
ENSMUSG00000079018	Ly6c1	-1.084353361	1.41E-05	ENSMUSG00000019139	Isyna1	1.263345647	2.06E-49
ENSMUSG00000027199	Gatm	-1.086018451	5.04E-06	ENSMUSG00000053113	Socs3	1.256771093	2.60E-48
ENSMUSG00000062309	Rpp25	-1.087123311	2.55E-06	ENSMUSG00000050370	Ch25h	1.255865941	4.33E-133
ENSMUSG00000020475	Pgam2	-1.090875407	5.91E-05	ENSMUSG00000047187	Rab2a	1.253907165	9.73E-278
ENSMUSG00000055013	Agap1	-1.090963566	1.31E-32	ENSMUSG00000001300	Efnb2	1.253426311	2.23E-75
ENSMUSG00000009394	Syn2	-1.091128006	0.002080329	ENSMUSG00000047604	Frat2	1.252537079	1.70E-05
ENSMUSG00000027932	Slc27a3	-1.092758114	6.55E-14	ENSMUSG00000056427	Slit3	1.252331447	1.20E-18
ENSMUSG00000029536	Gatc	-1.093113404	3.81E-26	ENSMUSG00000055430	Nap1l5	1.251318126	1.63E-08
ENSMUSG00000070469	Adamtsl3	-1.093662049	8.12E-175	ENSMUSG00000028064	Sema4a	1.249699221	0.010598914
ENSMUSG00000113529	Gm47484	-1.093671549	0.012878565	ENSMUSG00000036381	P2ry14	1.248939206	0.012481197
ENSMUSG00000051098	Mblac2	-1.094195325	2.81E-06	ENSMUSG00000049511	Htr1b	1.24807556	0.000946979
ENSMUSG00000021697	Depdc1b	-1.095408604	5.27E-08	ENSMUSG00000020277	Pfkl	1.247878604	1.01E-143
ENSMUSG00000048799	Cep120	-1.09573937	8.23E-88	ENSMUSG00000021213	Akr1c13	1.247132504	2.53E-22
ENSMUSG00000032374	Plod2	-1.096404188	2.68E-292	ENSMUSG00000022999	Lmbr1l	1.24491081	5.22E-41
ENSMUSG00000042115	Klhdc8a	-1.098214555	3.22E-39	ENSMUSG00000095649	Gm8979	1.24305772	1.63E-15
ENSMUSG00000048186	Bend7	-1.099030024	0.023355808	ENSMUSG00000053117	E330013P04Rik	1.242588299	0.000643821
ENSMUSG00000097684	Gm26645	-1.099584794	0.012067354	ENSMUSG00000039958	Etfbkmt	1.24185593	3.95E-15
ENSMUSG00000023034	Nr4a1	-1.102441607	9.36E-35	ENSMUSG00000031266	Gla	1.23982441	3.55E-198
ENSMUSG00000038351	Sgsm2	-1.102669724	7.95E-24	ENSMUSG00000037994	Slc9b2	1.239329117	0.0174554
ENSMUSG00000018865	Sult4a1	-1.106622575	0.000426581	ENSMUSG00000026525	Opn3	1.230005483	1.16E-07
ENSMUSG00000040260	Daam2	-1.109128619	2.25E-23	ENSMUSG00000039865	Slc44a3	1.229732956	0.004408375
ENSMUSG00000029442	Wdr66	-1.110264365	8.97E-07	ENSMUSG00000029348	Asphd2	1.229580113	2.26E-05
ENSMUSG00000020381	Mrnip	-1.11144878	0.041780754	ENSMUSG00000023991	Foxp4	1.228797233	4.06E-80
ENSMUSG00000071862	Lrrtm2	-1.11230361	6.40E-07	ENSMUSG00000012889	Podnl1	1.228417799	0.040028598
ENSMUSG00000030554	Synm	-1.112658512	2.91E-09	ENSMUSG00000059182	Skap2	1.223411791	6.85E-72
ENSMUSG00000028005	Gucy1b1	-1.11268377	1.08E-228	ENSMUSG00000053025	Sv2b	1.223159071	0.04404341
ENSMUSG00000087370	Tmem170b	-1.113554876	2.58E-25	ENSMUSG00000017929	B4galt5	1.22148923	1.38E-170
ENSMUSG00000067818	Myl9	-1.113560037	2.73E-77	ENSMUSG00000036192	Rorb	1.219397617	0.008471688
ENSMUSG00000026955	Sapcd2	-1.114452602	3.15E-12	ENSMUSG00000068417	Pnp2	1.218474644	1.42E-35
ENSMUSG00000040918	Slc19a2	-1.118668631	4.54E-146	ENSMUSG00000025911	Adhfe1	1.216597333	7.87E-18
ENSMUSG00000049191	Rtl5	-1.119355647	1.81E-43	ENSMUSG00000015869	Prpsap1	1.215663691	1.26E-116
ENSMUSG00000032218	Ccnb2	-1.119647102	1.44E-105	ENSMUSG00000056671	Prelid2	1.214629109	3.22E-06
ENSMUSG00000040424	Hipk4	-1.121306848	0.009982697	ENSMUSG00000072949	Acot1	1.213341732	1.54E-12
ENSMUSG00000031538	Plat	-1.121933288	2.03E-174	ENSMUSG00000105565	Gm43566	1.212917754	0.000571148
ENSMUSG00000024486	Hbegf	-1.123721869	1.19E-107	ENSMUSG00000096965	3300005D01Rik	1.211843647	2.70E-89
ENSMUSG00000044201	Cdc25c	-1.124201936	2.75E-18	ENSMUSG00000044471	Lncpint	1.209616528	0.001088062
ENSMUSG00000039450	Dcxr	-1.124555264	5.18E-36	ENSMUSG00000046169	Adamts6	1.209158787	2.88E-45
ENSMUSG00000027107	Chrna1	-1.125277653	9.19E-10	ENSMUSG00000069170	Adgrv1	1.208607257	0.03995026
ENSMUSG00000086245	Gm16170	-1.127103414	0.009416204	ENSMUSG00000096606	Tpbgl	1.206760181	5.83E-06
ENSMUSG00000027171	Prrg4	-1.127713122	1.08E-13	ENSMUSG00000105867	Gm42517	1.206527583	0.006110815
ENSMUSG00000030772	Dkk3	-1.13002625	8.49E-47	ENSMUSG00000047446	Arl4a	1.205966583	4.53E-21
ENSMUSG00000032841	Prr5l	-1.13088063	1.95E-36	ENSMUSG00000117628	Gm50012	1.205921119	1.31E-13
ENSMUSG00000062151	Unc13c	-1.131756617	6.56E-08	ENSMUSG00000033361	Prrg3	1.204939123	1.35E-68
ENSMUSG00000045333	Zfp423	-1.132729551	7.49E-05	ENSMUSG00000095545	Zfp969	1.203077796	0.00928432
ENSMUSG00000071359	Tbpl1	-1.132818259	7.44E-47	ENSMUSG00000079101	Esd-ps	1.201581233	0.047815166
ENSMUSG00000078349	AW011738	-1.134565922	0.000147457	ENSMUSG00000027678	Ncoa3	1.201571045	3.76E-66
ENSMUSG00000003585	Sec14l2	-1.135177226	9.97E-10	ENSMUSG00000026202	Tuba4a	1.200081095	1.39E-119
ENSMUSG00000040723	Rcsd1	-1.137395727	3.33E-89	ENSMUSG00000029499	Pxmp2	1.199998607	1.10E-05
ENSMUSG00000001053	N4bp3	-1.139302766	0.000108596	ENSMUSG00000053199	Arhgap20	1.198185117	0.034971938
ENSMUSG00000066842	Hmcn1	-1.139588208	4.20E-05	ENSMUSG00000086513	9130208D14Rik	1.197058482	8.02E-11
ENSMUSG00000044469	Tnfaip8l1	-1.141260186	5.19E-05	ENSMUSG00000036769	Wdr44	1.195710173	5.09E-87
ENSMUSG00000105176	Gm43668	-1.143173916	0.001353812	ENSMUSG00000004837	Grap	1.194083956	7.44E-16
ENSMUSG00000025076	Casp7	-1.144251883	5.67E-31	ENSMUSG00000050549	Fam241a	1.192107386	1.31E-14

ENSMUSG00000027612	Mmp24	-1.145002479	0.039659983
ENSMUSG00000042213	Zfand4	-1.145172132	0.000206891
ENSMUSG00000037940	Inpp4b	-1.14819214	0.002136873
ENSMUSG00000032010	Usp2	-1.150349213	2.87E-25
ENSMUSG00000022464	Slc38a4	-1.15073892	3.68E-20
ENSMUSG00000037685	Atp8a1	-1.153066547	9.32E-07
ENSMUSG00000116461	Gm44502	-1.153445981	0.022499688
ENSMUSG00000036306	Lzts1	-1.154153708	0.030593032
ENSMUSG00000103649	Gm37768	-1.155435814	0.000209522
ENSMUSG00000049493	Pls1	-1.157579078	2.98E-05
ENSMUSG00000026447	Pik3c2b	-1.159268795	0.004139912
ENSMUSG00000027715	Ccna2	-1.16149627	2.11E-133
ENSMUSG00000028525	Pde4b	-1.16562214	7.53E-66
ENSMUSG00000014164	Klhl3	-1.166113114	0.000881156
ENSMUSG00000025154	Arhgap19	-1.169035978	5.04E-17
ENSMUSG00000079017	Ifi27l2a	-1.170636445	9.82E-27
ENSMUSG00000020330	Hmmr	-1.171148284	1.80E-116
ENSMUSG00000033730	Egr3	-1.172543659	1.95E-07
ENSMUSG00000097806	Gm6556	-1.173219229	1.86E-05
ENSMUSG00000024338	Psmb8	-1.173701972	3.49E-10
ENSMUSG00000090698	Apold1	-1.173988445	4.12E-07
ENSMUSG00000026582	Sele	-1.17563989	3.52E-33
ENSMUSG00000036223	Ska1	-1.176651208	7.60E-13
ENSMUSG00000043165	Lor	-1.177278044	2.87E-08
ENSMUSG00000040253	Gbp7	-1.178789371	0.001961727
ENSMUSG00000023927	Satb1	-1.187366661	5.21E-09
ENSMUSG00000070425	Xntrpc	-1.189690012	0.026197538
ENSMUSG00000000253	Gmpr	-1.192181252	1.40E-12
ENSMUSG00000021388	Aspn	-1.194186653	1.83E-24
ENSMUSG00000026548	Slamf9	-1.194331798	2.88E-06
ENSMUSG00000023032	Slc4a8	-1.194687065	0.0016813
ENSMUSG00000027221	Chst1	-1.194966273	4.17E-15
ENSMUSG00000039556	Ppp1r3f	-1.195038649	5.58E-05
ENSMUSG00000028568	Btf3l4	-1.197621582	6.66E-106
ENSMUSG00000040430	Pitpnc1	-1.198023072	9.52E-13
ENSMUSG00000040447	Spns2	-1.202336881	2.09E-12
ENSMUSG00000118394	Gm50475	-1.202951819	0.010190182
ENSMUSG00000037166	Ppp1r14a	-1.204676523	1.03E-10
ENSMUSG00000007613	Tgfbr1	-1.204753353	1.79E-140
ENSMUSG00000038418	Egr1	-1.206511059	2.84E-111
ENSMUSG00000037253	Mex3c	-1.206979566	2.08E-160
ENSMUSG00000025950	Idh1	-1.209263237	1.76E-166
ENSMUSG00000079671	2610203C22Rik	-1.20991055	1.68E-05
ENSMUSG00000022220	Adcy4	-1.210602969	1.72E-43
ENSMUSG00000041329	Atp1b2	-1.211098681	0.001912556
ENSMUSG00000024640	Psat1	-1.211106147	2.99E-94
ENSMUSG00000079317	Trappc2	-1.211557744	1.61E-45
ENSMUSG00000033355	Rtp4	-1.211771949	0.010354045
ENSMUSG00000021208	Ifi27l2b	-1.214377325	2.44E-05
ENSMUSG00000055805	Fmnl1	-1.21840527	1.58E-07
ENSMUSG00000013089	Etv5	-1.219485123	1.36E-83
ENSMUSG00000036040	Adamtsl2	-1.221295088	8.68E-13
ENSMUSG00000032224	Fam81a	-1.222824763	0.017457879
ENSMUSG00000027333	Smox	-1.223885736	6.30E-27
ENSMUSG00000017692	Rhbdl3	-1.226721903	0.003692587
ENSMUSG00000027200	Sema6d	-1.226843788	4.56E-111
ENSMUSG00000048603	Gm9828	-1.227157049	0.040909239
ENSMUSG00000032289	Thsd4	-1.231416967	0.039989605
ENSMUSG00000103726	Gm30074	-1.232325985	0.007815942
ENSMUSG00000020182	Ddc	-1.233566359	0.000313596
ENSMUSG00000097789	Gm2115	-1.234754584	4.64E-10
ENSMUSG00000006344	Ggt5	-1.235769541	2.05E-11
ENSMUSG00000021319	Sfrp4	-1.236081052	3.30E-14
ENSMUSG00000109783	Gm45338	-1.236364672	0.000835
ENSMUSG00000029352	Crybb3	-1.240800993	0.015070647
ENSMUSG00000000782	Tcf7	-1.242068591	0.001351711
ENSMUSG00000048126	Col6a3	-1.244187373	5.36E-71
ENSMUSG00000024043	Arhgap28	-1.24661901	9.14E-33
ENSMUSG00000018927	Ccl6	-1.24888137	7.32E-11
ENSMUSG00000017446	C1qtnf1	-1.253609678	3.40E-34
ENSMUSG00000004891	Nes	-1.256130061	1.90E-220
ENSMUSG00000052516	Robo2	-1.256974898	2.11E-14
ENSMUSG00000025582	Nptx1	-1.259817079	0.030561511
ENSMUSG00000031780	Ccl17	-1.26182376	1.10E-06
ENSMUSG00000078922	Tgtp1	-1.26377826	3.84E-05
ENSMUSG00000054013	Tmem179	-1.268651491	0.016946064
ENSMUSG00000018983	E2f2	-1.270986094	2.42E-07

ENSMUSG00000022323	Rida	1.189293548	5.15E-31
ENSMUSG00000072653	Zfp783	1.189245558	0.001795247
ENSMUSG00000086212	Mkln1os	1.187139554	0.044778164
ENSMUSG00000087249	Gm16062	1.186270232	5.95E-06
ENSMUSG00000110344	Gm45716	1.185364078	4.37E-06
ENSMUSG00000039031	Arhgap18	1.185154404	6.67E-120
ENSMUSG00000021271	Zfp839	1.181256923	1.24E-17
ENSMUSG00000079657	Rab26	1.180282739	0.028007989
ENSMUSG00000048636	A730049H05Rik	1.177521158	1.12E-05
ENSMUSG00000068874	Selenbp1	1.176418869	7.92E-33
ENSMUSG00000028402	Mpdz	1.173008261	1.47E-105
ENSMUSG00000108461	AV356131	1.171295583	0.005670253
ENSMUSG00000031379	Pir	1.170732607	1.70E-50
ENSMUSG00000032501	Trib1	1.170704471	2.36E-36
ENSMUSG00000022456	Septin3	1.17026367	1.87E-11
ENSMUSG00000001910	Nacc1	1.167456857	4.71E-118
ENSMUSG00000078942	Naip6	1.166985505	0.003607697
ENSMUSG00000052430	Bmpr1b	1.166525325	4.36E-15
ENSMUSG00000059146	Ntrk3	1.16567956	1.12E-05
ENSMUSG00000028637	Ccdc30	1.162707419	0.00479127
ENSMUSG00000045414	Dipk2a	1.162427611	2.29E-125
ENSMUSG00000039089	L3mbtl3	1.162399012	3.60E-90
ENSMUSG00000033066	Gas7	1.162398646	7.31E-90
ENSMUSG00000008734	Gprc5b	1.161817848	1.40E-170
ENSMUSG00000062380	Tubb3	1.160651658	2.85E-119
ENSMUSG00000041112	Elmo1	1.160578513	2.43E-42
ENSMUSG00000024235	Map3k8	1.15999409	2.25E-24
ENSMUSG00000041235	Chd7	1.157035288	5.75E-30
ENSMUSG00000061118	Dnajc30	1.156630661	9.33E-22
ENSMUSG00000037185	Krt80	1.153615175	6.28E-19
ENSMUSG00000032217	Rnf111	1.153347487	9.51E-131
ENSMUSG00000049999	Ppp1r3d	1.15211668	2.24E-05
ENSMUSG00000025373	Rnf41	1.151078295	8.69E-104
ENSMUSG00000100779	Gm29292	1.149082639	0.008526608
ENSMUSG00000024014	Pim1	1.147352157	7.23E-52
ENSMUSG00000064247	Plcxd1	1.147263517	0.005563558
ENSMUSG00000021508	Cxcl14	1.143594288	4.26E-16
ENSMUSG00000032246	Calml4	1.142274729	0.021728311
ENSMUSG00000032489	Kif9	1.141866692	9.91E-05
ENSMUSG00000044229	Nxpe4	1.138996917	1.43E-35
ENSMUSG00000024621	Csf1r	1.136543393	3.52E-41
ENSMUSG00000031963	Bmper	1.131519441	7.38E-73
ENSMUSG00000039633	Lonrf1	1.128880504	5.08E-11
ENSMUSG00000078606	Gm4070	1.128534314	3.16E-12
ENSMUSG00000026670	Uap1	1.127134337	4.04E-210
ENSMUSG00000104476	Gm38211	1.122101034	0.002243204
ENSMUSG00000108825	Gm45838	1.121890028	7.51E-06
ENSMUSG00000024867	Pip5k1b	1.12151694	0.000897944
ENSMUSG00000038217	Tlcd2	1.121057851	1.11E-40
ENSMUSG00000020407	Upp1	1.120318061	0.000244688
ENSMUSG00000045868	Gvin1	1.119537243	6.86E-13
ENSMUSG00000022894	Adamts5	1.117811892	2.03E-118
ENSMUSG00000028803	Nipal3	1.117555065	2.42E-11
ENSMUSG00000020227	Irak3	1.117215672	9.50E-23
ENSMUSG00000044854	1700056E22Rik	1.117085791	0.007729732
ENSMUSG00000005057	Sh2b2	1.116663662	3.72E-09
ENSMUSG00000036144	Meox2	1.11541356	5.74E-10
ENSMUSG00000031765	Mt1	1.108097421	8.16E-151
ENSMUSG00000086392	Mccc1os	1.107579234	0.016046744
ENSMUSG00000106288	Gm43593	1.106566997	0.029745218
ENSMUSG00000021567	Nkd2	1.105265472	9.87E-114
ENSMUSG00000001998	Ap4e1	1.104735752	1.67E-33
ENSMUSG00000106825	2510016D11Rik	1.102113882	3.86E-05
ENSMUSG00000048240	Gng7	1.101295966	6.10E-05
ENSMUSG00000021054	Sgpp1	1.100839803	3.24E-149
ENSMUSG00000091514	Gm17484	1.09703067	0.022862985
ENSMUSG00000025478	Dpysl4	1.094563534	0.00028387
ENSMUSG00000021575	Ahrr	1.093734735	4.48E-07
ENSMUSG00000112825	Gm9118	1.09345384	0.004208519
ENSMUSG00000029209	Gnpda2	1.093127671	7.67E-70
ENSMUSG00000004040	Stat3	1.091217199	1.30E-268
ENSMUSG00000030259	Rassf8	1.089679835	3.21E-100
ENSMUSG00000018199	Ro60	1.088818916	1.38E-63
ENSMUSG00000101037	Gm28424	1.088618102	0.009652729
ENSMUSG00000045917	Tmem268	1.087293359	4.20E-16
ENSMUSG00000027287	Snap23	1.085965157	7.95E-143
ENSMUSG00000057123	Gja5	1.084499946	0.000101194

ENSMUSG00000036022	Fam122b	-1.278229299	7.63E-24	ENSMUSG00000059439	Bcas3	1.084110908	7.80E-49
ENSMUSG00000036553	Sh3tc1	-1.278704272	6.03E-10	ENSMUSG00000013539	Tango2	1.083611377	5.68E-38
ENSMUSG00000004933	Matk	-1.280576778	0.025319522	ENSMUSG00000029136	Rbks	1.082923115	0.000314119
ENSMUSG00000094845	Tmem95	-1.281175583	0.018900965	ENSMUSG00000031558	Slit2	1.080625981	8.28E-108
ENSMUSG00000055027	Smyd1	-1.282748664	0.000157147	ENSMUSG00000029577	Ube3b	1.078329265	1.34E-94
ENSMUSG00000053693	Mast1	-1.285307277	0.032062665	ENSMUSG00000053475	Tnfaip6	1.078292408	4.25E-46
ENSMUSG00000028943	Espn	-1.289321058	1.39E-06	ENSMUSG00000021069	Pygl	1.078088607	1.72E-17
ENSMUSG00000037628	Cdkn3	-1.291171095	5.17E-36	ENSMUSG00000058809	Hspd1-ps3	1.077261423	2.29E-06
ENSMUSG00000096727	Psmb9	-1.291867705	0.00103706	ENSMUSG00000034220	Gpc1	1.073876597	5.72E-60
ENSMUSG00000042155	Klhl23	-1.293474741	1.84E-15	ENSMUSG00000027208	Fgf7	1.073543194	2.22E-41
ENSMUSG00000021822	Plau	-1.29597369	3.42E-210	ENSMUSG00000011256	Adam19	1.071917799	3.44E-123
ENSMUSG00000018012	Rac3	-1.297136349	9.33E-06	ENSMUSG00000038725	Pkhd1l1	1.070354445	8.24E-25
ENSMUSG00000004880	Lbr	-1.297218898	1.15E-81	ENSMUSG00000028278	Rragd	1.069782444	3.34E-63
ENSMUSG00000068606	Gm4841	-1.300975189	0.038824034	ENSMUSG00000036330	Slc18a1	1.068920317	0.007899134
ENSMUSG00000041642	Kif21b	-1.303599359	8.79E-16	ENSMUSG00000078898	Zfp968	1.065775303	0.000217463
ENSMUSG00000031137	Fgf13	-1.309840866	1.48E-10	ENSMUSG00000028124	Gclm	1.06539499	8.44E-102
ENSMUSG00000050640	Tmem150c	-1.310909096	0.034866545	ENSMUSG00000044122	Proca1	1.063302721	0.002809589
ENSMUSG00000114133	Gm20075	-1.311125543	2.36E-13	ENSMUSG00000024593	Megf10	1.06215039	1.35E-33
ENSMUSG00000052302	Tbc1d30	-1.313145262	0.023343524	ENSMUSG00000038342	Mlxip	1.060107458	5.43E-100
ENSMUSG00000042109	Csdc2	-1.313249263	4.30E-17	ENSMUSG00000034993	Vat1	1.057937113	7.18E-269
ENSMUSG00000063388	BC023105	-1.315384808	0.033545916	ENSMUSG00000036782	Klhl13	1.057439864	4.09E-125
ENSMUSG00000031292	Cdkl5	-1.31571292	3.31E-12	ENSMUSG00000031729	Ist1	1.05631068	8.46E-150
ENSMUSG00000004633	Chn2	-1.319768263	2.97E-15	ENSMUSG00000026289	Atg16l1	1.055666007	9.16E-73
ENSMUSG00000050666	Vstm4	-1.324569917	0.008633965	ENSMUSG00000038028	Tigar	1.055432905	1.33E-21
ENSMUSG00000025612	Bach1	-1.32631347	7.78E-100	ENSMUSG00000023473	Celsr3	1.053894732	0.001364456
ENSMUSG00000087006	Gm13889	-1.326918703	0.00011359	ENSMUSG00000035509	Fbxl21	1.053052208	0.000253303
ENSMUSG00000026873	Phf19	-1.327023986	1.14E-26	ENSMUSG00000030560	Ctsc	1.052838651	4.79E-27
ENSMUSG00000042834	Nrep	-1.33302015	9.15E-187	ENSMUSG00000005413	Hmox1	1.052680953	3.41E-222
ENSMUSG00000025507	Pidd1	-1.334670188	6.49E-49	ENSMUSG00000091243	Vgll3	1.051795332	7.43E-239
ENSMUSG00000028884	Rpa2	-1.342122447	1.44E-46	ENSMUSG00000030946	Lhpp	1.04999254	5.34E-10
ENSMUSG00000022296	Baalc	-1.343059101	2.94E-07	ENSMUSG00000030861	Acadsb	1.04959932	7.55E-173
ENSMUSG00000037235	Mxd4	-1.347072738	2.28E-236	ENSMUSG00000032350	Gclc	1.04844688	8.91E-127
ENSMUSG00000029309	Sparcl1	-1.359841881	6.00E-45	ENSMUSG00000019528	Gyg	1.047566012	1.26E-165
ENSMUSG00000113909	Gm36377	-1.360808746	0.001154999	ENSMUSG00000038020	Rapgefl1	1.046955195	0.000225219
ENSMUSG00000028782	Adgrb2	-1.362564871	9.18E-06	ENSMUSG00000034674	Tdg	1.046809425	5.14E-107
ENSMUSG00000034738	Nostrin	-1.363670471	6.19E-27	ENSMUSG00000034300	Fam53c	1.046048765	4.57E-66
ENSMUSG00000054206	Gzmm	-1.370178741	0.008481553	ENSMUSG00000025934	Gsta3	1.045904414	5.67E-66
ENSMUSG00000040605	Bace2	-1.372690858	9.64E-50	ENSMUSG00000102573	Gm7265	1.045324789	6.91E-05
ENSMUSG00000085658	Gm15704	-1.375295639	0.029399661	ENSMUSG00000035671	Zswim4	1.045008951	1.87E-65
ENSMUSG00000094483	Purb	-1.376207544	1.40E-180	ENSMUSG00000022146	Osmr	1.044726607	8.22E-99
ENSMUSG00000055210	Foxd2	-1.378031053	0.002764954	ENSMUSG00000074647	Fam83c	1.044290164	2.24E-05
ENSMUSG00000033207	Mamdc2	-1.378061666	8.96E-43	ENSMUSG00000037638	Zbtb42	1.042942833	1.97E-09
ENSMUSG00000020623	Map2k6	-1.382880223	3.35E-07	ENSMUSG00000060147	Serpinb6a	1.04200146	5.84E-178
ENSMUSG00000021055	Esr2	-1.384521642	0.02607596	ENSMUSG00000104876	Trdc	1.041325314	0.036802874
ENSMUSG00000086825	Gm15675	-1.388920487	7.17E-13	ENSMUSG00000021360	Gcnt2	1.041091134	3.06E-49
ENSMUSG00000034522	Zfp395	-1.392830695	1.45E-12	ENSMUSG00000031936	Hephl1	1.039633636	0.00083885
ENSMUSG00000113918	Gm6566	-1.392867533	0.009471233	ENSMUSG00000035107	Dcbld2	1.037109576	5.85E-158
ENSMUSG00000042359	Osbpl6	-1.405288684	1.40E-15	ENSMUSG00000041958	Pigs	1.03617327	1.43E-68
ENSMUSG00000058656	Samd12	-1.408430792	1.05E-05	ENSMUSG00000030022	Adamts9	1.036165522	7.22E-63
ENSMUSG00000040138	Ndp	-1.412780341	0.038492057	ENSMUSG00000029641	Rasl11a	1.035272886	3.56E-38
ENSMUSG00000052572	Dlg2	-1.414693859	1.51E-18	ENSMUSG00000114737	Gm8483	1.034873198	0.028338899
ENSMUSG00000072720	Myo18b	-1.419097156	3.53E-14	ENSMUSG00000045664	Cdc42ep2	1.031554689	1.77E-57
ENSMUSG00000031886	Ces2e	-1.421228377	2.57E-23	ENSMUSG00000053411	Cbx7	1.030707879	7.11E-06
ENSMUSG00000017417	Plxdc1	-1.42330373	0.000936609	ENSMUSG00000087213	2810408I11Rik	1.028030619	1.52E-07
ENSMUSG00000099583	H3c4	-1.426015415	1.18E-05	ENSMUSG00000049580	Tsku	1.027647448	2.88E-118
ENSMUSG00000031776	Arl2bp	-1.427316964	7.38E-241	ENSMUSG00000036136	Fam110c	1.027097883	6.05E-16
ENSMUSG00000038677	Scube3	-1.427925754	0.046104361	ENSMUSG00000111063	Zkscan7	1.025999239	0.028896643
ENSMUSG00000035493	Tgfbi	-1.429853448	2.00E-66	ENSMUSG00000044952	Kctd21	1.025945698	2.14E-15
ENSMUSG00000004748	Mtfp1	-1.430612112	0.007774714	ENSMUSG00000021950	Anxa8	1.025320682	1.79E-16
ENSMUSG00000079363	Gbp4	-1.431192368	0.006041389	ENSMUSG00000015305	Sash1	1.02521519	8.75E-89
ENSMUSG00000059901	Adamts14	-1.431310355	6.23E-51	ENSMUSG00000039607	Rbms3	1.025075547	1.21E-84
ENSMUSG00000052415	Tchh	-1.432064403	5.85E-12	ENSMUSG00000087684	1200007C13Rik	1.025008164	0.006980659
ENSMUSG00000046997	Spsb4	-1.437880562	1.81E-06	ENSMUSG00000032265	Tent5a	1.024759485	3.06E-43
ENSMUSG00000079429	Mroh2a	-1.438620166	3.50E-10	ENSMUSG00000039428	Tmem135	1.023620426	1.92E-65
ENSMUSG00000039814	Xkr5	-1.440563098	2.55E-06	ENSMUSG00000102691	Gm37780	1.022227451	0.00649592
ENSMUSG00000040037	Negr1	-1.441110348	1.06E-06	ENSMUSG00000028793	Rnf19b	1.020997905	1.21E-101
ENSMUSG00000028399	Ptprd	-1.442256439	4.95E-103	ENSMUSG00000092569	Gm20544	1.018226043	2.60E-11
ENSMUSG00000031712	Il15	-1.444674688	1.54E-12	ENSMUSG00000002897	Il17ra	1.018164541	8.32E-78
ENSMUSG00000039217	Il18	-1.449296023	0.014918506	ENSMUSG00000020101	Vsir	1.016741116	1.40E-08
ENSMUSG00000089812	Gm15867	-1.450400099	0.035939144	ENSMUSG00000033149	Phldb2	1.014448274	1.17E-162
ENSMUSG00000049866	Arl4c	-1.451754279	5.35E-32	ENSMUSG00000038518	Jarid2	1.013462886	1.28E-23
ENSMUSG00000024598	Fbn2	-1.456981418	1.44E-08	ENSMUSG00000046230	Vps13a	1.012231245	3.23E-26
ENSMUSG00000041515	Irf8	-1.460193864	5.17E-16	ENSMUSG00000089756	Zfp966	1.011623356	0.003542881
ENSMUSG00000044461	Shisa2	-1.464902866	0.000807477	ENSMUSG00000029153	Ociad2	1.011140072	2.50E-15
ENSMUSG00000046314	Stxbp6	-1.467366444	7.01E-09	ENSMUSG00000027668	Mfn1	1.010063432	1.46E-85
ENSMUSG00000094626	Tmem121b	-1.470208685	0.004901571	ENSMUSG00000031709	Tbc1d9	1.009701268	3.28E-40
ENSMUSG00000032034	Kcnj5	-1.475983563	1.03E-06	ENSMUSG00000024533	Spire1	1.009041271	7.68E-41

ENSMUSG00000053414	Hunk	-1.476009185	0.004823847	ENSMUSG00000039652	Cpeb3	1.008402576	2.69E-14
ENSMUSG00000004655	Aqp1	-1.481017258	8.62E-135	ENSMUSG00000007908	Hmgcll1	1.0083826	0.033780304
ENSMUSG00000027574	Nkain4	-1.481260776	1.46E-36	ENSMUSG00000048376	F2r	1.004938236	9.36E-192
ENSMUSG00000057657	Rps18-ps3	-1.489559328	0.029739529	ENSMUSG00000029482	Aacs	1.001566738	6.75E-82
ENSMUSG00000056665	Them6	-1.497575298	9.97E-13	ENSMUSG00000053615	Gm9913	1.001415498	4.24E-06
ENSMUSG00000085042	Abhd11os	-1.498240484	0.014470871	ENSMUSG00000048264	Dip2c	1.000720684	1.14E-45
ENSMUSG00000051627	H1f4	-1.500001855	2.11E-05	ENSMUSG00000041423	Paqr6	1.000577606	0.023530578
ENSMUSG00000045658	Pid1	-1.504594541	7.92E-46	ENSMUSG00000020262	Adarb1	1.000326394	2.78E-53
ENSMUSG00000037709	Fam13a	-1.504703788	0.002576079	ENSMUSG00000052584	Serp2	-1.000196668	0.046460303
ENSMUSG00000037846	Rtkn2	-1.504744411	8.65E-07	ENSMUSG00000026096	Osgepl1	-1.000476891	4.03E-11
ENSMUSG00000031553	Adam3	-1.505073389	0.014690126	ENSMUSG00000060743	H3f3a	-1.001499021	5.02E-118
ENSMUSG00000041596	Nlrp5-ps	-1.506194484	0.027943387	ENSMUSG00000036912	Piwil4	-1.001535104	0.000940354
ENSMUSG00000036912	Piwil4	-1.508502022	1.89E-06	ENSMUSG00000007655	Cav1	-1.001788523	1.65E-89
ENSMUSG00000032744	Heyl	-1.511711691	1.25E-30	ENSMUSG00000039377	Hlx	-1.001863996	1.25E-08
ENSMUSG00000073295	Nudt11	-1.515049722	1.60E-07	ENSMUSG00000042099	Kank3	-1.002473946	3.36E-05
ENSMUSG00000022309	Angpt1	-1.515546183	3.85E-09	ENSMUSG00000031906	Smpd3	-1.003103381	3.67E-28
ENSMUSG00000037126	Psd	-1.517643381	3.62E-14	ENSMUSG00000035051	Dhx57	-1.003940273	7.91E-44
ENSMUSG00000020251	Glt8d2	-1.520263969	1.32E-68	ENSMUSG00000028121	Bcar3	-1.005310672	0.000970902
ENSMUSG00000000244	Tspan32	-1.520608276	0.00042714	ENSMUSG00000028414	Fktn	-1.006712933	1.26E-42
ENSMUSG00000056055	Sag	-1.520856894	0.046305469	ENSMUSG00000029765	Plxna4	-1.00688826	1.48E-22
ENSMUSG00000043496	Tril	-1.521290608	8.31E-23	ENSMUSG00000038264	Sema7a	-1.007065005	4.66E-35
ENSMUSG00000032135	Mcam	-1.523764595	5.44E-55	ENSMUSG00000038319	Kcnh2	-1.007068438	2.65E-10
ENSMUSG00000072437	Nanos1	-1.5250046	6.93E-45	ENSMUSG00000027932	Slc27a3	-1.007070899	1.18E-11
ENSMUSG00000033849	B3galt2	-1.528205702	0.000690234	ENSMUSG00000087006	Gm13889	-1.007217738	0.003461538
ENSMUSG00000116597	Gm536	-1.52941683	0.001726869	ENSMUSG00000105402	Gm3716	-1.007264189	0.030269115
ENSMUSG00000046585	Cfap58	-1.530875915	5.61E-05	ENSMUSG00000019726	Lyst	-1.007363611	8.32E-35
ENSMUSG00000041205	Map6d1	-1.531267976	0.041773784	ENSMUSG00000068245	Phf11d	-1.008949243	3.36E-21
ENSMUSG00000031790	Mmp15	-1.533479083	1.19E-06	ENSMUSG00000026434	Nucks1	-1.009434692	4.39E-128
ENSMUSG00000039853	Trim14	-1.534430016	0.002786725	ENSMUSG00000030035	Wbp1	-1.009440861	3.00E-26
ENSMUSG00000032750	Gab3	-1.535775109	0.003418338	ENSMUSG00000066894	Vsig10	-1.009548122	1.25E-15
ENSMUSG00000032698	Lmo2	-1.549434925	9.50E-59	ENSMUSG00000074925	Ptar1	-1.010620492	1.16E-79
ENSMUSG00000086524	Pabpc1l2a-ps	-1.549814972	0.018471335	ENSMUSG00000031748	Gnao1	-1.011208769	7.39E-15
ENSMUSG00000027329	Spef1	-1.550318237	2.83E-33	ENSMUSG00000046111	Cep295	-1.013313433	1.86E-27
ENSMUSG00000052942	Glis3	-1.557177327	7.18E-47	ENSMUSG00000049823	Zbtb12	-1.014415955	1.98E-06
ENSMUSG00000022032	Scara5	-1.559182405	5.23E-07	ENSMUSG00000097048	1600020E01Rik	-1.014446527	3.15E-06
ENSMUSG00000062588	Gm6104	-1.561898952	0.041613945	ENSMUSG00000085604	Dhx58os	-1.014673202	0.000156693
ENSMUSG00000021340	Gpld1	-1.562046526	0.000833744	ENSMUSG00000031529	Tnks	-1.014675411	9.03E-98
ENSMUSG00000047842	Diras2	-1.568242829	7.31E-13	ENSMUSG00000035024	Ncapd3	-1.015969078	8.05E-39
ENSMUSG00000084353	Gm15452	-1.569689138	0.005697791	ENSMUSG00000022673	Mcm4	-1.016034842	1.19E-110
ENSMUSG00000000049	Apoh	-1.576294324	0.010190182	ENSMUSG00000036086	Zranb3	-1.017951087	3.42E-06
ENSMUSG00000049357	Brd8dc	-1.587422979	0.000191365	ENSMUSG00000021536	Adcy2	-1.018178496	6.85E-06
ENSMUSG00000108218	Olfr1372-ps1	-1.590929844	6.48E-10	ENSMUSG00000049657	Zbtb5	-1.018419919	5.53E-15
ENSMUSG00000030793	Pycard	-1.595202668	2.09E-18	ENSMUSG00000030008	Pradc1	-1.019342214	1.48E-05
ENSMUSG00000053263	Gm12592	-1.596539472	0.010674615	ENSMUSG00000090210	Itga10	-1.019766253	9.02E-07
ENSMUSG00000035095	Fam167a	-1.597053627	7.23E-05	ENSMUSG00000020990	Cdkl1	-1.019826175	0.007364632
ENSMUSG00000028713	Cyp4b1	-1.600283144	0.024432687	ENSMUSG00000026785	Pkn3	-1.020491874	5.24E-08
ENSMUSG00000078234	Klhdc7a	-1.604422279	6.97E-08	ENSMUSG00000026429	Ube2t	-1.020737937	1.13E-17
ENSMUSG00000051726	Kcnf1	-1.605262145	0.001191157	ENSMUSG00000038152	5033430I15Rik	-1.020822243	0.003852337
ENSMUSG00000045725	Prr15	-1.606436193	0.000586436	ENSMUSG00000054074	Skida1	-1.021171155	1.39E-08
ENSMUSG00000026308	Klhl30	-1.606488713	1.44E-32	ENSMUSG00000001507	Itga3	-1.021881633	1.29E-41
ENSMUSG00000038146	Notch3	-1.610602777	4.26E-121	ENSMUSG00000025925	Terf1	-1.022584845	9.54E-25
ENSMUSG00000038540	Tmc3	-1.630009819	0.001091713	ENSMUSG00000031626	Sorbs2	-1.023249089	2.98E-84
ENSMUSG00000078921	Tgtp2	-1.631176242	0.000383142	ENSMUSG00000062075	Lmnb2	-1.023303224	1.98E-31
ENSMUSG00000015733	Capza2	-1.633772038	0	ENSMUSG00000045930	Clec14a	-1.023412682	0.006171002
ENSMUSG00000118138	Gm50322	-1.634812943	8.10E-09	ENSMUSG00000076437	Selenoh	-1.02395083	7.13E-27
ENSMUSG00000004791	Pgf	-1.641630347	1.52E-31	ENSMUSG00000045751	Mms22l	-1.02440281	3.90E-20
ENSMUSG00000030214	Plbd1	-1.642464785	0.022693958	ENSMUSG00000055745	Rtl6	-1.02488024	5.53E-07
ENSMUSG00000032595	Cdhr4	-1.643137928	0.042605919	ENSMUSG00000025867	Cplx2	-1.025735011	1.65E-42
ENSMUSG00000022831	Hcls1	-1.644494376	2.73E-24	ENSMUSG00000036943	Rab8b	-1.026239147	7.59E-63
ENSMUSG00000006235	Epor	-1.64981804	0.000769282	ENSMUSG00000036206	Sh3bp4	-1.026895812	1.02E-33
ENSMUSG00000025610	Map3k7cl	-1.650355454	6.79E-15	ENSMUSG00000043631	Ecm2	-1.02726254	1.45E-11
ENSMUSG00000034282	Evpl	-1.652381045	2.04E-06	ENSMUSG00000029687	Ezh2	-1.027364018	5.19E-66
ENSMUSG00000057346	Apol9a	-1.658255536	5.05E-10	ENSMUSG00000070034	Sp110	-1.028423012	1.98E-08
ENSMUSG00000108049	Gm44168	-1.658941815	2.70E-05	ENSMUSG00000028024	Enpep	-1.029248023	1.31E-09
ENSMUSG00000111171	Gm47815	-1.664039103	0.022876991	ENSMUSG00000064141	Zfp69	-1.030646713	0.008330238
ENSMUSG00000052485	Tmem171	-1.667852504	1.48E-07	ENSMUSG00000016995	Matn4	-1.032144828	9.20E-05
ENSMUSG00000024440	Pcdh12	-1.672398226	2.24E-13	ENSMUSG00000022945	Chaf1b	-1.032881212	5.99E-19
ENSMUSG00000037962	Rflna	-1.674702994	0.000402132	ENSMUSG00000067276	Capn6	-1.033691362	2.47E-39
ENSMUSG00000050014	Apol10b	-1.683090414	0.004379096	ENSMUSG00000022831	Hcls1	-1.034510532	5.55E-13
ENSMUSG00000037379	Spon2	-1.688137172	5.51E-107	ENSMUSG00000071722	Spin4	-1.036431371	1.36E-10
ENSMUSG00000006930	Hap1	-1.690125297	3.90E-07	ENSMUSG00000090272	Mndal	-1.03685804	1.51E-11
ENSMUSG00000044313	Mab21l3	-1.693109999	6.13E-10	ENSMUSG00000021978	Extl3	-1.037327893	1.19E-123
ENSMUSG00000104342	Gm36401	-1.705279835	7.75E-06	ENSMUSG00000016496	Cd274	-1.038193449	0.000633126
ENSMUSG00000062257	Opcml	-1.715226303	0.000308611	ENSMUSG00000035403	Crb2	-1.03905647	0.001869314
ENSMUSG00000049241	Hcar1	-1.718186308	0.00576895	ENSMUSG00000006800	Sulf2	-1.03977599	2.04E-143
ENSMUSG00000087651	1500009L16Rik	-1.719929636	3.02E-35	ENSMUSG00000074916	Chst14	-1.039961094	2.21E-42
ENSMUSG00000068246	Apol9b	-1.722518708	7.42E-09	ENSMUSG00000002274	Metrn	-1.040601008	1.04E-30

ENSMUSG00000086763	Plxna4os1	-1.725048563	0.015148548	ENSMUSG00000102516	Gm38340	-1.040784016	0.010370107
ENSMUSG00000026051	Ecrg4	-1.736651783	7.57E-86	ENSMUSG00000035842	Ddx11	-1.04241435	6.67E-11
ENSMUSG00000047638	Nr1h4	-1.740228835	2.48E-32	ENSMUSG00000040612	Ildr2	-1.042626328	2.37E-69
ENSMUSG00000085389	1700003M07Rik	-1.740783105	0.000555892	ENSMUSG00000074863	Platr25	-1.043268099	2.28E-12
ENSMUSG00000073600	Prob1	-1.756165066	0.00155452	ENSMUSG00000022761	Lztr1	-1.043423765	5.37E-107
ENSMUSG00000057137	Tmem140	-1.763370881	7.36E-35	ENSMUSG00000031997	Trpc6	-1.044336217	1.19E-11
ENSMUSG00000054435	Gimap4	-1.768105042	0.002466131	ENSMUSG00000078853	Igtp	-1.044580803	4.90E-12
ENSMUSG00000051537	Gm5124	-1.770961216	3.47E-05	ENSMUSG00000026274	Pask	-1.044752033	4.88E-16
ENSMUSG00000041757	Plekha6	-1.775689086	3.66E-76	ENSMUSG00000032340	Neo1	-1.044975969	6.70E-124
ENSMUSG00000021200	Asb2	-1.776498274	0.007032451	ENSMUSG00000039599	Fam149b	-1.045363282	7.07E-26
ENSMUSG00000111656	Gm47232	-1.783181044	0.030485009	ENSMUSG00000070867	Trabd2b	-1.046725953	8.24E-25
ENSMUSG00000033544	Angptl1	-1.786201254	2.65E-05	ENSMUSG00000090919	Pabpc4l	-1.048423901	0.00079728
ENSMUSG00000031698	Mylk3	-1.787905508	3.47E-05	ENSMUSG00000034258	Flvcr2	-1.048697923	0.016551303
ENSMUSG00000081111	Gm5913	-1.793370565	0.000977067	ENSMUSG00000051375	Pcdh1	-1.048815287	4.00E-30
ENSMUSG00000045287	Rtn4rl1	-1.819137972	2.55E-15	ENSMUSG00000055884	Fancm	-1.049042522	3.80E-26
ENSMUSG00000024168	Tmem204	-1.822221465	2.62E-66	ENSMUSG00000021265	Slc25a29	-1.049594232	0.001063007
ENSMUSG00000044927	H1f10	-1.824988558	6.70E-36	ENSMUSG00000004633	Chn2	-1.050062967	4.03E-12
ENSMUSG00000057606	Colq	-1.840375853	1.57E-05	ENSMUSG00000032011	Thy1	-1.050616416	3.60E-209
ENSMUSG00000033578	Tmem35a	-1.851422336	3.97E-18	ENSMUSG00000045903	Npas4	-1.050682318	0.000396596
ENSMUSG00000013523	Bcas1	-1.85703834	0.018139372	ENSMUSG00000097231	Gm26852	-1.050848479	0.038149175
ENSMUSG00000070348	Ccnd1	-1.85732849	0	ENSMUSG00000022385	Gtse1	-1.052489116	2.47E-95
ENSMUSG00000102553	D930036K23Rik	-1.857513609	0.007886755	ENSMUSG00000071537	Klrg2	-1.053513782	1.94E-06
ENSMUSG00000044365	Cxxc4	-1.867732451	0.004315488	ENSMUSG00000040620	Dhx33	-1.054210808	5.58E-61
ENSMUSG00000026564	Dusp27	-1.868058997	2.13E-32	ENSMUSG00000046491	C1qtnf2	-1.054348393	1.50E-27
ENSMUSG00000024525	Impa2	-1.8685108	4.28E-57	ENSMUSG00000075588	Hoxb2	-1.054768534	6.67E-05
ENSMUSG00000073293	Nudt10	-1.871146123	6.97E-05	ENSMUSG00000003070	Efna2	-1.056071431	0.003800252
ENSMUSG00000040432	Ltb4r2	-1.874463534	0.016043285	ENSMUSG00000070407	Hs3st3b1	-1.056442713	1.53E-06
ENSMUSG00000027223	Mapk8ip1	-1.877572617	7.39E-91	ENSMUSG00000044807	Zfp354c	-1.056761255	2.97E-19
ENSMUSG00000001029	Icam2	-1.881627539	7.97E-13	ENSMUSG00000004151	Etv1	-1.057506075	7.30E-34
ENSMUSG00000084842	Pabpc1l2b-ps	-1.889984897	0.002055141	ENSMUSG00000031951	Tmem231	-1.057689011	9.12E-12
ENSMUSG00000018566	Slc2a4	-1.890923775	0.00118783	ENSMUSG00000040447	Spns2	-1.059135212	3.06E-10
ENSMUSG00000036655	Colec11	-1.899150203	6.26E-15	ENSMUSG00000076431	Sox4	-1.059924857	2.53E-81
ENSMUSG00000109452	Gm44530	-1.903561897	0.016685448	ENSMUSG00000079017	Ifi27l2a	-1.060084891	0.009443959
ENSMUSG00000044041	Krt13	-1.90535499	0.011579087	ENSMUSG00000104713	Gbp6	-1.061162703	8.56E-07
ENSMUSG00000068522	Aard	-1.911218986	0.005605847	ENSMUSG00000042210	Abhd14a	-1.061427824	6.91E-17
ENSMUSG00000021798	Ldb3	-1.922331576	1.40E-24	ENSMUSG00000044881	Coa4	-1.061649105	1.06E-06
ENSMUSG00000039899	Fgl2	-1.923384419	1.37E-90	ENSMUSG00000045672	Col27a1	-1.061821424	7.72E-09
ENSMUSG00000028211	Trp53inp1	-1.930057232	0	ENSMUSG00000051359	Ncald	-1.061925394	4.65E-30
ENSMUSG00000040856	Dlk1	-1.933253287	9.11E-05	ENSMUSG00000044864	Ankrd50	-1.063128685	1.21E-174
ENSMUSG00000021118	Plek2	-1.944945554	0.014424761	ENSMUSG00000015217	Hmgb3	-1.063353061	9.17E-17
ENSMUSG00000051359	Ncald	-1.977944125	6.41E-70	ENSMUSG00000056025	Clca3a1	-1.063479494	2.76E-280
ENSMUSG00000074673	Ttll9	-1.987354189	0.01444414	ENSMUSG00000035845	Alg12	-1.063716387	3.82E-11
ENSMUSG00000015405	Ace2	-1.992769174	0.010016584	ENSMUSG00000025265	Fgd1	-1.0649155	4.34E-45
ENSMUSG00000072966	Gprasp2	-2.001381183	0.002186638	ENSMUSG00000018012	Rac3	-1.065329514	6.36E-05
ENSMUSG00000048402	Gli2	-2.009792088	1.07E-05	ENSMUSG00000022092	Ppp3cc	-1.066947694	2.56E-27
ENSMUSG00000037977	6430571L13Rik	-2.024719484	0.017038968	ENSMUSG00000025001	Hells	-1.067142127	1.09E-51
ENSMUSG00000029120	Ppp2r2c	-2.054073546	0.004379879	ENSMUSG00000000093	Tbx2	-1.067528189	1.03E-12
ENSMUSG00000057880	Abat	-2.062395375	3.24E-131	ENSMUSG00000097820	E530011L22Rik	-1.069574487	0.01344835
ENSMUSG00000109225	4930513N20Rik	-2.064905404	0.002942751	ENSMUSG00000037126	Psd	-1.070231971	1.45E-06
ENSMUSG00000038128	Camk4	-2.06565849	0.001193917	ENSMUSG00000048960	Prex2	-1.070358352	1.06E-67
ENSMUSG00000036295	Lrrn3	-2.066826206	6.80E-14	ENSMUSG00000060044	Tmem26	-1.071246853	0.020167014
ENSMUSG00000021703	Serinc5	-2.073070598	2.84E-201	ENSMUSG00000048218	Amigo2	-1.071731977	3.05E-06
ENSMUSG00000106062	Gm43820	-2.085249986	8.61E-07	ENSMUSG00000026988	Wdsub1	-1.073307711	1.84E-24
ENSMUSG00000104965	Gm43437	-2.100351036	7.09E-05	ENSMUSG00000031840	Rab3a	-1.073850559	2.18E-13
ENSMUSG00000019301	Hsd17b1	-2.101166973	0.022884659	ENSMUSG00000038244	Mical2	-1.073944075	1.89E-213
ENSMUSG00000032648	Pygm	-2.106173895	2.26E-20	ENSMUSG00000062563	Cys1	-1.07442809	7.18E-06
ENSMUSG00000117098	Gm49909	-2.111007606	0.023916194	ENSMUSG00000031877	Ces2g	-1.074684195	6.22E-48
ENSMUSG00000030470	Csrp3	-2.116673221	0.013852994	ENSMUSG00000039943	Plcb4	-1.074903753	2.56E-82
ENSMUSG00000042078	Svop	-2.122184976	1.60E-07	ENSMUSG00000033578	Tmem35a	-1.075006633	5.33E-08
ENSMUSG00000025475	Adgra1	-2.138978636	4.98E-06	ENSMUSG00000064128	Cenpj	-1.075058409	5.45E-22
ENSMUSG00000058589	Anks1b	-2.139621589	2.05E-24	ENSMUSG00000070802	Pnmal2	-1.075591205	3.54E-05
ENSMUSG00000034818	Celf5	-2.165770089	2.98E-15	ENSMUSG00000044702	Palb2	-1.075930653	2.06E-08
ENSMUSG00000029838	Ptn	-2.175986223	2.49E-205	ENSMUSG00000070469	Adamtsl3	-1.076167411	9.22E-214
ENSMUSG00000048485	Zbtb8b	-2.199302273	2.79E-10	ENSMUSG00000038894	Irs2	-1.076350065	3.00E-24
ENSMUSG00000078956	Gm14221	-2.217225782	4.34E-07	ENSMUSG00000028018	Gstcd	-1.076416036	4.87E-30
ENSMUSG00000085006	BC021767	-2.225622204	2.35E-06	ENSMUSG00000001986	Gria3	-1.076846752	2.32E-75
ENSMUSG00000029797	Sspo	-2.229991585	0.0011531	ENSMUSG00000030772	Dkk3	-1.077712336	7.53E-78
ENSMUSG00000020798	Spns3	-2.234876801	0.003831579	ENSMUSG00000031292	Cdkl5	-1.07802797	2.24E-08
ENSMUSG00000109134	Gm45076	-2.253132167	0.016165989	ENSMUSG00000061607	Mdc1	-1.07960251	8.52E-64
ENSMUSG00000109056	A630009H07Rik	-2.260885779	4.91E-05	ENSMUSG00000028464	Tpm2	-1.080066576	3.30E-70
ENSMUSG00000030465	Psd3	-2.262116294	4.21E-195	ENSMUSG00000030107	Usp18	-1.080244317	0.002943815
ENSMUSG00000031778	Cx3cl1	-2.268095341	5.85E-242	ENSMUSG00000027811	4930579G24Rik	-1.081343555	2.38E-19
ENSMUSG00000047146	Tet1	-2.275992099	5.27E-14	ENSMUSG00000031861	Lpar2	-1.084919978	2.72E-16
ENSMUSG00000032769	Trpa1	-2.276222885	2.24E-05	ENSMUSG00000028078	Dclk2	-1.085512274	4.94E-14
ENSMUSG00000025701	Alox5	-2.349449545	3.74E-08	ENSMUSG00000069808	Fam57a	-1.085927587	1.06E-57
ENSMUSG00000051457	Spn	-2.359445012	5.97E-18	ENSMUSG00000027313	Chac1	-1.08819949	5.41E-14
ENSMUSG00000048939	Atp13a5	-2.393740836	7.14E-05	ENSMUSG00000037353	Letmd1	-1.088308878	1.14E-37

ENSMUSG00000028555	Ttc39a	-2.423842982	1.21E-12	ENSMUSG00000027896	Slc16a4	-1.088801186	1.26E-06
ENSMUSG00000106139	Gm30648	-2.451011442	4.55E-06	ENSMUSG00000043987	Cep164	-1.088934725	6.49E-47
ENSMUSG00000021448	Shc3	-2.458050024	1.74E-07	ENSMUSG00000003062	Stard3nl	-1.089935382	1.81E-56
ENSMUSG00000070644	Etnk2	-2.462802444	5.50E-06	ENSMUSG00000000686	Abhd15	-1.09045061	1.54E-05
ENSMUSG00000026126	Ptpn18	-2.47299987	3.98E-05	ENSMUSG00000029283	Cdc7	-1.091057329	6.64E-19
ENSMUSG00000035189	Ano4	-2.474692536	2.12E-14	ENSMUSG00000051048	P4ha3	-1.092045063	2.20E-33
ENSMUSG00000100798	Gm19589	-2.508606341	0.010329677	ENSMUSG00000043795	Prr33	-1.092474912	4.35E-06
ENSMUSG00000034792	Gna15	-2.545084215	0.023680415	ENSMUSG00000008136	Fhl2	-1.092998593	1.48E-139
ENSMUSG00000010122	Slc47a1	-2.564213498	0.004542155	ENSMUSG00000097960	A330074K22Rik	-1.093315806	0.026070218
ENSMUSG00000082196	Gm14231	-2.568704895	0.025624385	ENSMUSG00000036975	Tmem177	-1.094633806	1.01E-10
ENSMUSG00000026205	Slc23a3	-2.586912954	0.034602637	ENSMUSG00000051832	E230016K23Rik	-1.09499452	0.000422761
ENSMUSG00000074570	Cass4	-2.597940984	0.040195964	ENSMUSG00000026831	1700007K13Rik	-1.095243309	9.42E-06
ENSMUSG00000038224	Serpinf2	-2.60378485	0.018467316	ENSMUSG00000031381	Piga	-1.095732967	1.55E-20
ENSMUSG00000029685	Asb15	-2.620345742	1.40E-05	ENSMUSG00000118394	Gm50475	-1.095760986	0.00785193
ENSMUSG00000026831	1700007K13Rik	-2.641136391	5.27E-23	ENSMUSG00000046818	Ddit4l	-1.096074442	3.25E-28
ENSMUSG00000042846	Lrrtm3	-2.655180904	0.032229801	ENSMUSG00000026281	Dtymk	-1.096911531	1.48E-40
ENSMUSG00000020787	P2rx1	-2.658596552	4.53E-06	ENSMUSG00000034544	Rsrc1	-1.097364126	3.63E-69
ENSMUSG00000035383	Pmch	-2.680131282	0.033715677	ENSMUSG00000058729	Lin9	-1.097839505	4.54E-23
ENSMUSG00000049593	Lce1h	-2.686444174	0.033942261	ENSMUSG00000027639	Samhd1	-1.097886937	1.25E-82
ENSMUSG00000035283	Adrb1	-2.699865614	9.35E-07	ENSMUSG00000109455	Gm44710	-1.099853117	0.045812678
ENSMUSG00000114409	Gm9042	-2.755530082	0.046294426	ENSMUSG00000032744	Heyl	-1.100275347	1.58E-18
ENSMUSG00000036596	Cpz	-2.757218283	2.55E-08	ENSMUSG00000025969	Nrp2	-1.100390929	2.90E-97
ENSMUSG00000062393	Dgkk	-2.788720183	0.004300532	ENSMUSG00000025323	Sp4	-1.100773992	0.000144208
ENSMUSG00000039304	Tnfsf10	-2.799340606	3.13E-07	ENSMUSG00000051331	Cacna1c	-1.100833928	3.68E-17
ENSMUSG00000057948	Unc13d	-2.815878292	0.000573764	ENSMUSG00000025912	Mybl1	-1.100910172	9.71E-33
ENSMUSG00000059645	Gm7361	-2.819050328	0.00479302	ENSMUSG00000006930	Hap1	-1.102537109	0.00076573
ENSMUSG00000084797	Gm14321	-2.834713257	0.04093851	ENSMUSG00000117545	Gm30794	-1.103518008	0.005180629
ENSMUSG00000029102	Hgfac	-2.853686873	0.007688328	ENSMUSG00000021952	Xpo4	-1.103859587	6.22E-42
ENSMUSG00000058396	Gpr182	-2.857553141	6.02E-64	ENSMUSG00000026147	Col9a1	-1.105032634	0.028885908
ENSMUSG00000040310	Alx4	-2.8941307	1.33E-10	ENSMUSG00000001493	Meox1	-1.106831281	3.20E-27
ENSMUSG00000099250	Rn7s2	-2.901742485	0.000279895	ENSMUSG00000045201	Lrrc3b	-1.108627018	4.72E-09
ENSMUSG00000046480	Scn4b	-2.939854365	0.001209979	ENSMUSG00000026413	Pkp1	-1.108811608	3.28E-54
ENSMUSG00000090394	4930523C07Rik	-2.97563584	2.61E-130	ENSMUSG00000033355	Rtp4	-1.109010381	0.005738586
ENSMUSG00000046908	Ltb4r1	-3.020833386	0.003538052	ENSMUSG00000029436	Mmp17	-1.109282857	2.92E-05
ENSMUSG00000002324	Rec8	-3.040900086	0.001786615	ENSMUSG00000032322	Pstpip1	-1.109367718	3.00E-06
ENSMUSG00000099021	Rn7s1	-3.198349601	0.000160468	ENSMUSG00000018339	Gpx3	-1.109368853	3.19E-79
ENSMUSG00000046999	1110032F04Rik	-3.229969293	3.81E-26	ENSMUSG00000079109	Pms2	-1.109845232	8.28E-18
ENSMUSG00000045534	Kcna5	-3.240637484	3.51E-17	ENSMUSG00000032085	Tagln	-1.109973436	6.40E-111
ENSMUSG00000066975	Cryba4	-3.279723755	0.038419087	ENSMUSG00000028884	Rpa2	-1.110706282	2.57E-31
ENSMUSG00000026715	Serpinc1	-3.28411369	0.012198416	ENSMUSG00000021906	Oxnad1	-1.11158574	3.37E-16
ENSMUSG00000078157	Fem1al	-3.299959556	0.029839041	ENSMUSG00000037894	H2az1	-1.111966496	5.54E-139
ENSMUSG00000035407	Kank4	-3.32665581	4.36E-06	ENSMUSG00000037235	Mxd4	-1.112004231	1.45E-209
ENSMUSG00000053310	Nrgn	-3.332692105	0.000129369	ENSMUSG00000002997	Prkar2b	-1.112456757	3.28E-163
ENSMUSG00000030310	Slc6a1	-3.345826689	0.000688605	ENSMUSG00000028933	Xrcc2	-1.112623896	5.70E-14
ENSMUSG00000019935	Slc17a8	-3.349889083	0.029240888	ENSMUSG00000027381	Bcl2l11	-1.112725491	2.68E-10
ENSMUSG00000100635	Gm29157	-3.352234178	0.000674312	ENSMUSG00000022766	Serpind1	-1.112800421	7.22E-10
ENSMUSG00000085723	Gm15915	-3.358568688	0.02727821	ENSMUSG00000071064	Zfp827	-1.113026168	1.58E-15
ENSMUSG00000068859	Sp9	-3.393829876	5.15E-05	ENSMUSG00000013878	Rnf170	-1.113408871	6.19E-52
ENSMUSG00000079355	Ackr4	-3.395352569	7.30E-145	ENSMUSG00000054967	Zfp647	-1.113890749	0.001301771
ENSMUSG00000032401	Lctl	-3.42063569	0.018613761	ENSMUSG00000040669	Phc1	-1.114406965	3.31E-35
ENSMUSG00000071551	Akr1c19	-3.467225839	0.008473868	ENSMUSG00000021388	Aspn	-1.115945551	1.90E-20
ENSMUSG00000038583	Pln	-3.494391271	0.021044587	ENSMUSG00000042302	Ehbp1	-1.116229811	9.08E-42
ENSMUSG00000025422	Agap2	-3.544038847	0.014223902	ENSMUSG00000025150	Cbr2	-1.117735323	0.002222195
ENSMUSG00000043164	Tmem212	-3.567785617	0.026753923	ENSMUSG00000063919	Srrm4	-1.117761205	0.001945031
ENSMUSG00000079019	Insl3	-3.593216939	0.047971677	ENSMUSG00000038736	Nudcd1	-1.117835034	1.97E-41
ENSMUSG00000086003	B230206L02Rik	-3.593868444	0.02255378	ENSMUSG00000032815	Fanca	-1.118178352	1.23E-30
ENSMUSG00000027559	Car3	-3.613030399	0.001731236	ENSMUSG00000025207	Sema4g	-1.12069621	0.02182405
ENSMUSG00000024155	Meiob	-3.620513217	0.00109335	ENSMUSG00000039114	Nrn1	-1.121334965	4.47E-27
ENSMUSG00000022015	Tnfsf11	-3.627230887	0.013156092	ENSMUSG00000079429	Mroh2a	-1.121401691	4.46E-07
ENSMUSG00000109764	Klkb1	-3.637031683	0.011553957	ENSMUSG00000034317	Trim59	-1.124283568	1.19E-95
ENSMUSG00000010529	Gm266	-3.774301157	0.04740897	ENSMUSG00000012017	Scarf2	-1.12596131	1.07E-100
ENSMUSG00000032131	Abcg4	-3.819224258	0.003042592	ENSMUSG00000060510	Zfp266	-1.126362938	7.07E-49
ENSMUSG00000050675	Gp1ba	-3.825127772	4.32E-17	ENSMUSG00000079654	Prrt4	-1.126930206	1.18E-06
ENSMUSG00000111293	Gm34006	-4.018507625	0.000755435	ENSMUSG00000056116	H2-T22	-1.12725937	3.03E-53
ENSMUSG00000109031	Gm45201	-4.213893837	0.03600708	ENSMUSG00000043051	Disc1	-1.127859489	0.008136759
ENSMUSG00000102009	4933400F21Rik	-4.339082589	0.005416599	ENSMUSG00000021130	Galnt16	-1.128096286	0.000737639
ENSMUSG00000039419	Cntnap2	-4.400667134	7.04E-06	ENSMUSG00000051037	Zfp455	-1.12849851	0.009920779
ENSMUSG00000027875	Hmgcs2	-4.406432512	0.01161004	ENSMUSG00000025156	Gps1	-1.128520899	1.07E-94
ENSMUSG00000060371	Caln1	-4.520984763	3.68E-05	ENSMUSG00000022469	Rapgef3	-1.129751591	1.42E-67
ENSMUSG00000084081	Gm12057	-5.008446511	0.020018731	ENSMUSG00000021262	Evl	-1.130353176	1.95E-40
ENSMUSG00000117799	Gm50295	-5.188347444	0.009924985	ENSMUSG00000046985	Tapt1	-1.132489629	8.85E-64
ENSMUSG00000038236	Hoxa7	-5.269545797	0.044862059	ENSMUSG00000041245	Wnk3	-1.133231904	2.40E-05
ENSMUSG00000083223	Gm15016	-5.273285439	0.043304032	ENSMUSG00000070047	Fat1	-1.133616438	7.97E-29
ENSMUSG00000102897	Gm37853	-5.273814104	0.045525043	ENSMUSG00000032410	Xrn1	-1.134023965	5.40E-39
ENSMUSG00000118015	Gm41708	-5.279348559	0.042994575	ENSMUSG00000114019	Gm47155	-1.134513484	0.013805391
ENSMUSG00000051596	Otop1	-5.281260408	0.043773616	ENSMUSG00000090698	Apold1	-1.134677686	5.22E-07
ENSMUSG00000082630	Gm14834	-5.281260408	0.043773616	ENSMUSG00000033763	Mtss2	-1.13516527	2.12E-30

ENSMUSG00000088504	Gm26490	-5.285410861	0.046513713	ENSMUSG00000031506	Ptpn7	-1.135683374	0.002894749
ENSMUSG00000102894	Gm37851	-5.325469576	0.041787454	ENSMUSG00000057706	Mex3b	-1.135883763	4.42E-16
ENSMUSG00000109523	Gdf1	-5.334987295	0.01706994	ENSMUSG00000044224	Dnajc21	-1.136997202	4.36E-46
ENSMUSG00000036913	Trim67	-5.336662552	0.037634984	ENSMUSG00000089862	Umad1	-1.137648837	6.50E-26
ENSMUSG00000087198	Tmem274	-5.340737196	0.03680851	ENSMUSG00000026640	Plxna2	-1.138552155	8.76E-88
ENSMUSG00000091272	Gm17641	-5.341144027	0.00053416	ENSMUSG00000054150	Syne3	-1.139317612	1.41E-25
ENSMUSG00000083921	Gm15750	-5.34348569	0.037769595	ENSMUSG00000032224	Fam81a	-1.139457626	0.019700421
ENSMUSG00000003477	Inmt	-5.383913049	0.039509116	ENSMUSG00000031924	Cyb5b	-1.140512446	3.58E-152
ENSMUSG00000086411	Gm14236	-5.393050974	0.037052779	ENSMUSG00000021390	Ogn	-1.140559782	3.84E-108
ENSMUSG00000093749	4921523L03Rik	-5.395707844	0.032457285	ENSMUSG00000024210	Ip6k3	-1.141070207	0.026948857
ENSMUSG00000085016	Gm11335	-5.402257458	0.03199232	ENSMUSG00000047749	Zc3hav1l	-1.141134583	7.31E-22
ENSMUSG00000029158	Yipf7	-5.403138676	0.032621916	ENSMUSG00000036109	Mbnl3	-1.141534407	9.78E-21
ENSMUSG00000103596	Gm37354	-5.406492032	0.037625536	ENSMUSG00000097471	5830432E09Rik	-1.141743785	0.004852379
ENSMUSG00000071342	Lsmem1	-5.45957552	0.0275705	ENSMUSG00000020340	Cyfip2	-1.141817228	0.011502265
ENSMUSG00000084892	Gm14471	-5.46371768	0.029551691	ENSMUSG00000056888	Glipr1	-1.142062868	1.37E-27
ENSMUSG00000017309	Cd300lg	-5.497527706	0.025458469	ENSMUSG00000026080	Chst10	-1.142913371	1.17E-06
ENSMUSG00000115074	Ndor1	-5.546933591	5.88E-05	ENSMUSG00000042606	Hirip3	-1.143417142	1.63E-14
ENSMUSG00000026686	Lmx1a	-5.557054267	0.020261354	ENSMUSG00000061411	Nol4l	-1.143467926	2.88E-16
ENSMUSG00000031409	Tceal6	-5.557841221	0.020830275	ENSMUSG00000049191	Rtl5	-1.143826824	2.12E-39
ENSMUSG00000086313	Gm15940	-5.56658299	0.020415722	ENSMUSG00000111521	Gm48529	-1.145103428	1.90E-35
ENSMUSG00000018776	Slc35g3	-5.567367619	0.020475917	ENSMUSG00000035045	Zc3h12b	-1.145843585	1.08E-09
ENSMUSG00000053963	Stum	-5.56859332	0.0212741	ENSMUSG00000081137	BC022960	-1.147398361	0.034138228
ENSMUSG00000041544	Disp3	-5.594412752	0.046732629	ENSMUSG00000030654	Arl6ip1	-1.149678373	8.77E-262
ENSMUSG00000085918	Gm13032	-5.594412752	0.046732629	ENSMUSG00000026843	Fubp3	-1.151197042	2.36E-124
ENSMUSG00000118234	Gm50115	-5.615144635	0.045116945	ENSMUSG00000020357	Flt4	-1.151488099	2.24E-76
ENSMUSG00000019906	Lin7a	-5.621642496	0.019242111	ENSMUSG00000041889	Shisa4	-1.151551182	4.72E-38
ENSMUSG00000097402	Gm17399	-5.648325364	0.041410987	ENSMUSG00000074785	Plxnc1	-1.151920259	6.85E-07
ENSMUSG00000048217	Nags	-5.651924175	0.041304428	ENSMUSG00000037020	Wdr62	-1.152206235	4.00E-29
ENSMUSG00000008489	Elavl2	-5.655833738	0.01456546	ENSMUSG00000044362	Ccdc89	-1.152981184	0.006686203
ENSMUSG00000029299	Abcg3	-5.6593498	0.015969822	ENSMUSG00000039231	Suv39h1	-1.153144015	6.75E-51
ENSMUSG00000044633	B530045E10Rik	-5.660413973	0.016504114	ENSMUSG00000019845	Tube1	-1.156144489	4.85E-07
ENSMUSG00000085057	Gm13415	-5.663346059	0.014843238	ENSMUSG00000034205	Loxl2	-1.156922238	3.11E-191
ENSMUSG00000043857	Mgat5b	-5.669140863	0.04093851	ENSMUSG00000074039	4930520O04Rik	-1.157582788	0.015948919
ENSMUSG00000029245	Epha5	-5.672038401	0.041963972	ENSMUSG00000035711	Dok3	-1.157825829	0.020866406
ENSMUSG00000103215	Gm38388	-5.673859086	0.04273904	ENSMUSG00000059674	Cdh24	-1.158732853	0.003697245
ENSMUSG00000097346	Rps18-ps4	-5.675817292	0.039311995	ENSMUSG00000025650	Col7a1	-1.16087012	4.04E-16
ENSMUSG00000021336	Slc17a4	-5.694337149	0.01493742	ENSMUSG00000059013	Sh2d3c	-1.162051773	7.29E-06
ENSMUSG00000060183	Cxcl11	-5.704493819	0.041152404	ENSMUSG00000033632	AW554918	-1.162117028	1.08E-10
ENSMUSG00000061578	Ksr2	-5.705803128	0.012102867	ENSMUSG00000097684	Gm26645	-1.162978968	0.001563115
ENSMUSG00000038403	Hjv	-5.733219382	0.037570919	ENSMUSG00000041117	Ccdc8	-1.164409945	1.13E-21
ENSMUSG00000026227	2810459M11Rik	-5.743648511	0.033545916	ENSMUSG00000021592	Arsk	-1.164848222	9.26E-37
ENSMUSG00000086165	Gm15690	-5.802461854	0.009152376	ENSMUSG00000035293	G2e3	-1.165040551	1.13E-58
ENSMUSG00000021999	Cpb2	-5.814046056	0.033729207	ENSMUSG00000018931	Natd1	-1.166869389	1.94E-67
ENSMUSG00000097613	Gm17597	-5.821701336	0.030113776	ENSMUSG00000038011	Dnah10	-1.167108026	0.011286982
ENSMUSG00000015890	Amdhd1	-5.834764451	0.026525803	ENSMUSG00000022021	Diaph3	-1.167283251	1.91E-143
ENSMUSG00000062939	Stat4	-5.836667566	0.00813027	ENSMUSG00000029605	Oas1b	-1.16729072	1.17E-06
ENSMUSG00000025002	Cyp2c55	-5.844163689	0.007973348	ENSMUSG00000078920	Ifi47	-1.168262262	4.88E-66
ENSMUSG00000022181	C6	-5.856707311	0.029500609	ENSMUSG00000085783	Gm9816	-1.168722885	0.005871925
ENSMUSG00000032773	Chrm1	-5.87147375	0.008010127	ENSMUSG00000026669	Mcm10	-1.169221815	6.78E-22
ENSMUSG00000085511	Gm11738	-5.878321178	0.006589699	ENSMUSG00000072964	Bhlhb9	-1.169361379	5.65E-56
ENSMUSG00000025082	Vwa2	-5.881121973	0.006583305	ENSMUSG00000019779	Frk	-1.17046235	1.31E-34
ENSMUSG00000037593	Rskr	-5.882311758	0.00642379	ENSMUSG00000029370	Rassf6	-1.170880605	4.17E-14
ENSMUSG00000031323	Dmrtc1a	-5.920641574	0.005557147	ENSMUSG00000027160	Ccdc34	-1.170912557	1.11E-25
ENSMUSG00000029369	Afm	-5.94021646	0.021791934	ENSMUSG00000036882	Arhgap33	-1.171958498	6.86E-05
ENSMUSG00000043230	Fam124b	-5.966060185	0.005283243	ENSMUSG00000026207	Speg	-1.172711271	8.32E-43
ENSMUSG00000026272	Agxt	-5.979454594	0.021410077	ENSMUSG00000047115	Fam221a	-1.173352451	0.000555676
ENSMUSG00000087004	Gm14154	-6.001151025	0.004122248	ENSMUSG00000049758	Olfr1318	-1.17343873	0.004238171
ENSMUSG00000113833	Gm48264	-6.00570507	0.017359625	ENSMUSG00000005107	Slc2a9	-1.17375038	2.08E-15
ENSMUSG00000002588	Pon1	-6.007772194	0.046623956	ENSMUSG00000026659	Dusp12	-1.173843143	8.04E-15
ENSMUSG00000055730	Ces2a	-6.04429157	0.043396842	ENSMUSG00000064344	mt-Tm	-1.174719062	0.000654602
ENSMUSG00000078235	Fam43b	-6.044465291	0.004126991	ENSMUSG00000096210	H1f0	-1.175101806	8.11E-113
ENSMUSG00000040017	Saa4	-6.079909031	0.04052211	ENSMUSG00000008730	Hipk1	-1.175845004	3.35E-112
ENSMUSG00000028715	Cyp4a14	-6.087035618	0.017142317	ENSMUSG00000102018	Iqank1	-1.177037089	0.030960264
ENSMUSG00000025127	Gcgr	-6.114668114	0.037796042	ENSMUSG00000035151	Elmod2	-1.178326332	2.83E-35
ENSMUSG00000110329	Gm45304	-6.129298716	0.003865992	ENSMUSG00000041757	Plekha6	-1.179425775	3.26E-37
ENSMUSG00000031173	Otc	-6.148609164	0.035286011	ENSMUSG00000038173	Enpp6	-1.179791748	0.002498671
ENSMUSG00000113326	Gm47586	-6.161358962	0.00585533	ENSMUSG00000013033	Adgrl1	-1.180163031	6.80E-58
ENSMUSG00000026725	Tnn	-6.172309813	0.010692015	ENSMUSG00000022803	Popdc2	-1.182027457	2.11E-22
ENSMUSG00000082644	Mup-ps19	-6.181770108	0.03293472	ENSMUSG00000097467	Gm26737	-1.182420472	8.56E-14
ENSMUSG00000086675	Plxna4os2	-6.212480808	0.009488609	ENSMUSG00000091191	Gm17334	-1.182586777	0.042478675
ENSMUSG00000037780	Mbl1	-6.214185655	0.030779677	ENSMUSG00000050107	Haspin	-1.182874129	1.23E-17
ENSMUSG00000023176	Cpn2	-6.214185655	0.030779677	ENSMUSG00000028475	Spaar	-1.184033042	1.20E-20
ENSMUSG00000025482	Odf3	-6.254141123	0.00186034	ENSMUSG00000032177	Pde4a	-1.184592891	3.91E-48
ENSMUSG00000062410	Hsd3b3	-6.257120572	0.003494485	ENSMUSG00000027203	Dut	-1.185488001	1.24E-26
ENSMUSG00000024131	Slc3a1	-6.258011701	0.002679756	ENSMUSG00000022199	Slc22a17	-1.186777638	1.28E-10
ENSMUSG00000045746	B230317F23Rik	-6.304957852	0.001277572	ENSMUSG00000015647	Lama5	-1.18717369	1.21E-82
ENSMUSG00000039097	Rln1	-6.330475474	0.003257476	ENSMUSG00000052676	Zmat1	-1.187218227	2.88E-13

ENSMUSG00000003379	Cd79a	-6.36618976	0.000987238
ENSMUSG00000106269	4930580E04Rik	-6.392573263	0.000771643
ENSMUSG00000043456	Zfp536	-6.446668919	0.000664478
ENSMUSG00000026114	Cnga3	-6.535331801	0.000439684
ENSMUSG00000059852	Kcng2	-6.582907018	0.000368082
ENSMUSG00000042102	Dmgdh	-6.641471024	0.000616694
ENSMUSG00000031410	Nxf7	-6.697979256	0.000205476
ENSMUSG00000035948	Acss3	-6.779454541	0.000174595
ENSMUSG00000045532	C1ql1	-6.798207681	9.90E-05
ENSMUSG00000030317	Timp4	-6.802323475	0.000106377
ENSMUSG00000048337	Npy4r	-6.899673979	5.65E-05
ENSMUSG00000027690	Slc2a2	-7.010706214	5.32E-05
ENSMUSG00000103492	Gm37412	-7.141146028	1.52E-05
ENSMUSG00000074628	Tldc2	-7.352727661	8.24E-06
ENSMUSG00000010064	Slc38a3	-8.579256927	0.000735784
ENSMUSG00000036864	Proser3	-1.188669075	1.52E-06
ENSMUSG00000021803	Cdhr1	-1.188773176	1.86E-05
ENSMUSG00000105135	Gm43667	-1.189318601	0.012484096
ENSMUSG00000022881	Rfc4	-1.191957396	4.73E-14
ENSMUSG00000005370	Msh6	-1.192491476	4.85E-67
ENSMUSG00000069237	Fam8a1	-1.19282112	2.32E-159
ENSMUSG00000109539	Gm44667	-1.193127938	3.19E-06
ENSMUSG00000038168	P3h2	-1.193673455	1.80E-21
ENSMUSG00000112941	Gm48623	-1.194949229	0.00014897
ENSMUSG00000062826	Ces2f	-1.196155097	0.049714627
ENSMUSG00000027820	Mme	-1.197455257	1.76E-14
ENSMUSG00000079465	Col4a3	-1.198941916	3.39E-27
ENSMUSG00000066842	Hmcn1	-1.19900011	6.39E-05
ENSMUSG00000020032	Nuak1	-1.199338353	1.83E-140
ENSMUSG00000004319	Clcn3	-1.201494596	3.30E-62
ENSMUSG00000028698	Pik3r3	-1.203140516	2.19E-63
ENSMUSG00000029156	Sgcb	-1.203632034	9.32E-88
ENSMUSG00000051413	Plagl2	-1.203950149	1.02E-30
ENSMUSG00000036611	Eepd1	-1.204502942	0.011438202
ENSMUSG00000042428	Mgat3	-1.204884078	2.62E-14
ENSMUSG00000036672	Cenpt	-1.205010311	3.50E-47
ENSMUSG00000029442	Wdr66	-1.205487074	3.18E-08
ENSMUSG00000025915	Sgk3	-1.205578095	2.77E-39
ENSMUSG00000030254	Rad18	-1.205584053	5.55E-48
ENSMUSG00000036678	Aaas	-1.20631984	4.79E-64
ENSMUSG00000044783	Hjurp	-1.206734889	9.92E-203
ENSMUSG00000020238	Ncln	-1.206781811	8.98E-110
ENSMUSG00000020785	Camkk1	-1.206801047	1.50E-13
ENSMUSG00000071181	3830408C21Rik	-1.206913663	0.00110458
ENSMUSG00000048096	Lmod1	-1.207352316	1.87E-05
ENSMUSG00000051235	Gen1	-1.207901348	5.21E-22
ENSMUSG00000037253	Mex3c	-1.208666059	2.63E-152
ENSMUSG00000038797	Zscan2	-1.208691465	0.000330372
ENSMUSG00000026700	Tnfsf4	-1.209529724	0.038845348
ENSMUSG00000043415	Otud1	-1.209590235	3.64E-52
ENSMUSG00000115149	9330188P03Rik	-1.210375248	1.17E-26
ENSMUSG00000061533	Cep128	-1.211196459	1.10E-14
ENSMUSG00000023243	Kcnk5	-1.211687936	1.47E-08
ENSMUSG00000039748	Exo1	-1.212428435	2.12E-16
ENSMUSG00000050222	Il17d	-1.217973907	1.51E-06
ENSMUSG00000025758	Plk4	-1.2188519	5.97E-60
ENSMUSG00000032607	Amt	-1.220425842	0.000211219
ENSMUSG00000043587	Pxylp1	-1.221901096	1.34E-68
ENSMUSG00000036752	Tubb4b	-1.222422569	6.60E-171
ENSMUSG00000013236	Ptprs	-1.222622044	7.42E-122
ENSMUSG00000028438	Kif24	-1.222967858	3.92E-09
ENSMUSG00000038600	Atp6v0a4	-1.224131707	0.001824796
ENSMUSG00000110537	Gm4316	-1.224189595	1.73E-05
ENSMUSG00000051278	Zgrf1	-1.225376785	7.45E-19
ENSMUSG00000020053	Igf1	-1.225481762	2.12E-107
ENSMUSG00000002870	Mcm2	-1.225600487	1.82E-129
ENSMUSG00000020439	Smtn	-1.226480198	2.53E-186
ENSMUSG00000005958	Ephb3	-1.22675579	1.39E-21
ENSMUSG00000100967	Gm29666	-1.227277824	0.034126117
ENSMUSG00000029309	Sparcl1	-1.229639427	1.37E-49
ENSMUSG00000095159	Tubb4b-ps1	-1.230036125	2.25E-12
ENSMUSG00000018849	Wwc1	-1.230146936	2.44E-31
ENSMUSG00000030122	Ptms	-1.230203588	4.88E-197
ENSMUSG00000031698	Mylk3	-1.230330161	0.01596495
ENSMUSG00000024451	Arap3	-1.230619525	1.89E-10
ENSMUSG00000031016	Wee1	-1.230804949	1.87E-43
ENSMUSG00000103649	Gm37768	-1.231078756	5.70E-05
ENSMUSG00000007656	Arpp19	-1.231309884	2.85E-148
ENSMUSG00000002799	Jag2	-1.231549426	0.020094352
ENSMUSG00000034271	Jdp2	-1.231793591	1.30E-44
ENSMUSG00000015714	Cers2	-1.232047069	6.24E-196
ENSMUSG00000063354	Slc39a4	-1.235131572	0.004231598
ENSMUSG00000021707	Dhfr	-1.235322599	7.37E-44
ENSMUSG00000025006	Sorbs1	-1.236607629	4.08E-57
ENSMUSG00000043398	Gpr135	-1.237626947	1.09E-05
ENSMUSG00000067215	Usp51	-1.238953695	0.010764248
ENSMUSG00000038047	Haus6	-1.239604892	1.88E-72
ENSMUSG00000104342	Gm36401	-1.241276074	0.000165155
ENSMUSG00000031785	Adgrg1	-1.24166633	0.003490181
ENSMUSG00000042190	Cmklr1	-1.241859582	1.31E-42
ENSMUSG00000049409	Prokr1	-1.242054907	0.024880519
ENSMUSG00000098098	Bvht	-1.242299651	8.24E-14

ENSMUSG00000054196	Cthrc1	-1.243249601	4.27E-18
ENSMUSG00000085399	Foxd2os	-1.244679745	2.69E-10
ENSMUSG00000047003	Zfp41	-1.244839204	2.46E-19
ENSMUSG00000041126	H2az2	-1.244876255	2.82E-51
ENSMUSG00000030795	Fus	-1.245747294	3.60E-179
ENSMUSG00000040424	Hipk4	-1.245967308	0.008365371
ENSMUSG00000032380	Dapk2	-1.246495136	1.35E-05
ENSMUSG00000038070	Cntln	-1.247136806	1.44E-24
ENSMUSG00000000223	Drp2	-1.247821833	2.71E-08
ENSMUSG00000084842	Pabpc1l2b-ps	-1.248696677	0.037225507
ENSMUSG00000042978	Sbk1	-1.249588371	1.11E-11
ENSMUSG00000087179	Gm14230	-1.250796752	3.93E-10
ENSMUSG00000020099	Unc5b	-1.253279963	3.17E-287
ENSMUSG00000037466	Tedc1	-1.253513087	1.63E-31
ENSMUSG00000031146	Plp2	-1.253550515	1.76E-133
ENSMUSG00000062488	Ifit3b	-1.254384388	0.001451074
ENSMUSG00000041828	Abca8a	-1.254959013	1.15E-75
ENSMUSG00000020331	Hcn2	-1.25593527	2.14E-09
ENSMUSG00000001604	Tcea3	-1.258035373	1.12E-21
ENSMUSG00000021811	Dnajc9	-1.258583596	4.41E-76
ENSMUSG00000024663	Rab3il1	-1.259097827	2.22E-21
ENSMUSG00000021714	Cenpk	-1.259142495	1.13E-10
ENSMUSG00000050410	Tcf19	-1.259222828	9.40E-29
ENSMUSG00000057948	Unc13d	-1.259622031	0.019815824
ENSMUSG00000031661	Nkd1	-1.259687164	6.94E-10
ENSMUSG00000062901	Klhl24	-1.260358326	6.21E-85
ENSMUSG00000024134	Six2	-1.261607211	6.65E-17
ENSMUSG00000019699	Akt3	-1.262846616	5.74E-119
ENSMUSG00000029366	Dck	-1.264909518	5.50E-41
ENSMUSG00000109904	Gm45819	-1.264989525	9.55E-05
ENSMUSG00000030528	Blm	-1.265964747	1.55E-26
ENSMUSG00000084353	Gm15452	-1.26625515	0.019561755
ENSMUSG00000025240	Sacm1l	-1.266326628	6.08E-143
ENSMUSG00000017652	Cd40	-1.266947605	1.92E-20
ENSMUSG00000020827	Mink1	-1.267124125	1.42E-81
ENSMUSG00000035778	Ggta1	-1.267177643	2.18E-100
ENSMUSG00000030346	Rad51ap1	-1.267357964	1.21E-27
ENSMUSG00000021990	Spata13	-1.267617565	2.36E-25
ENSMUSG00000087651	1500009L16Rik	-1.267984068	1.45E-20
ENSMUSG00000019992	Mtfr2	-1.268176985	7.18E-16
ENSMUSG00000030031	Kbtbd8	-1.268814219	8.00E-05
ENSMUSG00000105504	Gbp5	-1.269409777	0.00125439
ENSMUSG00000023886	Smoc2	-1.27048481	1.25E-75
ENSMUSG00000061410	Zcchc14	-1.27257752	2.29E-103
ENSMUSG00000106978	N4bp2os	-1.272626498	0.013770867
ENSMUSG00000027009	Itga4	-1.273004383	1.66E-98
ENSMUSG00000023391	Dlx2	-1.273560458	3.15E-07
ENSMUSG00000037940	Inpp4b	-1.274028359	0.000396596
ENSMUSG00000059939	9430015G10Rik	-1.274060094	2.06E-18
ENSMUSG00000015243	Abca1	-1.274223567	7.77E-115
ENSMUSG00000002006	Pdzd4	-1.274355246	1.65E-10
ENSMUSG00000047420	Fam180a	-1.276254756	1.09E-14
ENSMUSG00000003779	Kif20a	-1.276396808	5.39E-189
ENSMUSG00000055805	Fmnl1	-1.278021787	3.35E-06
ENSMUSG00000111681	Gm47640	-1.278161646	4.09E-07
ENSMUSG00000022537	Tmem44	-1.278870323	6.22E-05
ENSMUSG00000029730	Mcm7	-1.282192837	3.58E-98
ENSMUSG00000023942	Slc29a1	-1.283895033	9.55E-133
ENSMUSG00000004127	Trmt10a	-1.284420878	1.52E-35
ENSMUSG00000031880	Rrad	-1.284770241	2.06E-06
ENSMUSG00000030411	Nova2	-1.285033103	0.008443868
ENSMUSG00000043008	Klhl6	-1.286254308	1.23E-103
ENSMUSG00000026610	Esrrg	-1.286378629	0.010575736
ENSMUSG00000028841	Cnksr1	-1.286876857	0.006521183
ENSMUSG00000070576	Mn1	-1.287579613	4.36E-51
ENSMUSG00000023336	Wfdc1	-1.287657256	0.048394227
ENSMUSG00000040372	Gpr63	-1.287797674	1.95E-07
ENSMUSG00000027961	Lrrc39	-1.290520578	5.10E-17
ENSMUSG00000025268	Maged2	-1.290609837	1.75E-147
ENSMUSG00000043885	Slc36a4	-1.291050013	7.60E-118
ENSMUSG00000074480	Mex3a	-1.291531699	2.25E-19
ENSMUSG00000102570	Gm37968	-1.291710573	0.022641914
ENSMUSG00000035095	Fam167a	-1.292553764	0.000603923
ENSMUSG00000019877	Serinc1	-1.292667223	5.22E-259
ENSMUSG00000038644	Pold1	-1.292773899	9.16E-45
ENSMUSG00000033411	Ctdspl2	-1.293074913	3.99E-49
ENSMUSG00000023341	Mx2	-1.293792377	0.000144472

ENSMUSG00000059824	Dbp	-1.294484343	1.76E-12
ENSMUSG00000068876	Cgn	-1.295820639	0.020872351
ENSMUSG00000001029	Icam2	-1.295862758	1.65E-06
ENSMUSG00000026355	Mcm6	-1.295925057	3.53E-163
ENSMUSG00000033446	Lpar6	-1.295951376	2.41E-46
ENSMUSG00000028264	Spaca1	-1.296755848	0.011766609
ENSMUSG00000028702	Rad54l	-1.297393824	1.03E-33
ENSMUSG00000020381	Mrnip	-1.29860206	0.020394749
ENSMUSG00000028614	Ndc1	-1.299236092	9.43E-118
ENSMUSG00000022053	Ebf2	-1.300014889	5.49E-34
ENSMUSG00000031398	Plxna3	-1.300231719	2.14E-14
ENSMUSG00000029822	Osbpl3	-1.300364208	3.27E-68
ENSMUSG00000053004	Hrh1	-1.301478993	0.00786968
ENSMUSG00000116165	Pdxp	-1.302020108	0.004393134
ENSMUSG00000024049	Myom1	-1.302396639	4.12E-07
ENSMUSG00000097084	Foxl1	-1.303693297	0.000493032
ENSMUSG00000094683	Ap3m1-ps	-1.304201367	0.030155478
ENSMUSG00000022833	Ccdc14	-1.304601045	5.94E-16
ENSMUSG00000063568	Jazf1	-1.304864155	2.26E-27
ENSMUSG00000097589	Dleu2	-1.304945327	4.50E-24
ENSMUSG00000032221	Mns1	-1.305600738	2.20E-27
ENSMUSG00000048779	P2ry6	-1.305795107	3.54E-05
ENSMUSG00000022758	P2rx6	-1.30620584	0.009843077
ENSMUSG00000006342	Susd2	-1.307862432	2.95E-11
ENSMUSG00000031290	Lrch2	-1.30895724	4.06E-26
ENSMUSG00000021936	Mapk8	-1.31074923	1.84E-65
ENSMUSG00000049929	Lpar4	-1.311075376	9.57E-12
ENSMUSG00000022150	Dab2	-1.311669586	6.01E-190
ENSMUSG00000040328	Olfr56	-1.31215906	1.51E-16
ENSMUSG00000039542	Ncam1	-1.312662464	7.41E-59
ENSMUSG00000037572	Wdhd1	-1.312711904	5.33E-31
ENSMUSG00000021464	Ror2	-1.314291733	3.71E-26
ENSMUSG00000026193	Fn1	-1.314816593	4.11E-250
ENSMUSG00000113529	Gm47484	-1.314868435	0.001216146
ENSMUSG00000040918	Slc19a2	-1.315269275	8.76E-169
ENSMUSG00000025271	Pfkfb1	-1.315587942	2.73E-08
ENSMUSG00000004267	Eno2	-1.316115167	2.06E-15
ENSMUSG00000028068	Iqgap3	-1.317981443	2.07E-78
ENSMUSG00000049470	Aff4	-1.319521406	3.82E-135
ENSMUSG00000068551	Zfp467	-1.319747945	2.97E-10
ENSMUSG00000056394	Lig1	-1.320833538	1.10E-86
ENSMUSG00000028152	Tspan5	-1.321621469	4.81E-60
ENSMUSG00000052396	Mogat2	-1.32248114	3.89E-11
ENSMUSG00000059323	Tonsl	-1.323099193	7.01E-13
ENSMUSG00000032946	Rasgrp2	-1.32329814	6.09E-06
ENSMUSG00000028228	Cpne3	-1.323319937	9.17E-134
ENSMUSG00000048078	Tenm4	-1.32346794	0.005534933
ENSMUSG00000046341	Gm11223	-1.323775894	0.000173159
ENSMUSG00000028444	Cntfr	-1.325359828	1.86E-06
ENSMUSG00000009575	Cbx5	-1.325519684	3.75E-237
ENSMUSG00000028066	Pmf1	-1.326278069	1.93E-45
ENSMUSG00000047746	Fbxo40	-1.327828325	0.004290059
ENSMUSG00000085042	Abhd11os	-1.329739097	0.011553418
ENSMUSG00000001270	Ckb	-1.329975963	2.18E-212
ENSMUSG00000002265	Peg3	-1.330297821	4.79E-37
ENSMUSG00000114660	Gm47881	-1.333050782	0.006363684
ENSMUSG00000116617	Gm49767	-1.33364202	0.036897862
ENSMUSG00000071984	Fndc1	-1.334214255	5.29E-93
ENSMUSG00000091144	Phf11c	-1.334363459	1.69E-05
ENSMUSG00000028807	Zbtb8a	-1.33631421	1.20E-16
ENSMUSG00000023908	Pkmyt1	-1.337560795	1.15E-33
ENSMUSG00000027641	Rbl1	-1.337925482	7.13E-52
ENSMUSG00000032511	Scn5a	-1.34019111	0.02283989
ENSMUSG00000046204	Pnma2	-1.340337318	0.020116548
ENSMUSG00000075585	6330403L08Rik	-1.34183843	9.59E-50
ENSMUSG00000030322	Mbd4	-1.342999187	2.53E-11
ENSMUSG00000018761	Mpdu1	-1.343792987	4.84E-72
ENSMUSG00000039109	F13a1	-1.344190646	0.017991443
ENSMUSG00000024472	Dcp2	-1.344939675	1.49E-60
ENSMUSG00000074818	Pdzd7	-1.345168728	8.54E-07
ENSMUSG00000027018	Hat1	-1.345928879	5.07E-77
ENSMUSG00000027022	Xirp2	-1.346205276	0.001221573
ENSMUSG00000097493	9930014A18Rik	-1.34690693	6.05E-13
ENSMUSG00000027663	Zmat3	-1.347009175	1.35E-264
ENSMUSG00000057762	Gm6169	-1.349709308	7.60E-10
ENSMUSG00000027217	Tspan18	-1.35075437	2.13E-08
ENSMUSG00000029334	Prkg2	-1.352084072	3.33E-28

ENSMUSG00000027171	Prrg4	-1.353383914	2.33E-26
ENSMUSG00000020681	Ace	-1.353573311	1.13E-126
ENSMUSG00000064343	mt-Tq	-1.353682141	0.005198327
ENSMUSG00000014361	Mertk	-1.354028464	6.80E-200
ENSMUSG00000060572	Mfap2	-1.355443095	0.001380022
ENSMUSG00000039697	Ncoa7	-1.356203975	9.28E-44
ENSMUSG00000034906	Ncaph	-1.356915896	7.66E-77
ENSMUSG00000019866	Crybg1	-1.357888029	1.37E-06
ENSMUSG00000046179	E2f8	-1.358889404	5.69E-45
ENSMUSG00000031196	F8	-1.362061647	2.51E-12
ENSMUSG00000003545	Fosb	-1.362852136	1.57E-10
ENSMUSG00000036867	Smad6	-1.362892828	3.65E-196
ENSMUSG00000024330	Col11a2	-1.363380084	0.026051834
ENSMUSG00000020926	Adam11	-1.363469125	0.039954183
ENSMUSG00000007080	Pole	-1.364819783	2.87E-63
ENSMUSG00000033220	Rac2	-1.365244998	0.036043153
ENSMUSG00000037892	Pcdh18	-1.365761561	1.54E-142
ENSMUSG00000028524	Sgip1	-1.366559104	9.10E-08
ENSMUSG00000045333	Zfp423	-1.367098439	5.59E-08
ENSMUSG00000020641	Rsad2	-1.367607955	0.008457123
ENSMUSG00000068744	Psrc1	-1.372726112	8.72E-98
ENSMUSG00000021965	Ska3	-1.372921721	2.27E-53
ENSMUSG00000048458	Inka2	-1.375467873	1.54E-63
ENSMUSG00000000049	Apoh	-1.37613438	0.012376641
ENSMUSG00000004105	Angptl2	-1.377739521	8.86E-277
ENSMUSG00000008658	Rbfox1	-1.378484051	2.42E-12
ENSMUSG00000071847	Apcdd1	-1.378484349	9.04E-32
ENSMUSG00000048782	Insc	-1.379312582	0.005570719
ENSMUSG00000026828	Galnt5	-1.38065237	5.08E-06
ENSMUSG00000054999	Naaladl1	-1.381269062	0.006562734
ENSMUSG00000049036	Tmem121	-1.383030244	1.49E-12
ENSMUSG00000027833	Shox2	-1.383302476	3.33E-19
ENSMUSG00000105096	Gbp10	-1.383461969	0.030739452
ENSMUSG00000019848	Popdc3	-1.383771618	0.000334034
ENSMUSG00000029638	Glcci1	-1.384807231	1.75E-30
ENSMUSG00000023909	Paqr4	-1.385276377	1.71E-48
ENSMUSG00000037418	Best1	-1.385512892	0.04759355
ENSMUSG00000058290	Espl1	-1.387306397	1.50E-81
ENSMUSG00000031385	Plxnb3	-1.389263653	0.045838525
ENSMUSG00000052516	Robo2	-1.389746652	3.52E-15
ENSMUSG00000079415	Cntf	-1.39024081	0.003571295
ENSMUSG00000112332	4930466K18Rik	-1.39090951	0.039957045
ENSMUSG00000045658	Pid1	-1.393117756	2.12E-41
ENSMUSG00000108957	Gm45235	-1.393637856	0.020165991
ENSMUSG00000005410	Mcm5	-1.393925357	3.65E-119
ENSMUSG00000025104	Hdgfl3	-1.395440324	4.23E-89
ENSMUSG00000087291	Gm11946	-1.396893538	0.006706741
ENSMUSG00000092167	Gm3696	-1.397121631	0.010545073
ENSMUSG00000033610	Pank1	-1.398290711	1.17E-21
ENSMUSG00000055296	Tmem245	-1.398355172	4.17E-23
ENSMUSG00000038917	3930402G23Rik	-1.399533074	0.029022764
ENSMUSG00000006678	Pola1	-1.399567239	1.32E-71
ENSMUSG00000031262	Cenpi	-1.401723111	3.23E-42
ENSMUSG00000024379	Tslp	-1.402737825	1.85E-07
ENSMUSG00000022015	Tnfsf11	-1.402963013	3.60E-05
ENSMUSG00000091952	Gm17709	-1.40314274	0.000309985
ENSMUSG00000005233	Spc25	-1.40330459	9.94E-75
ENSMUSG00000020638	Cmpk2	-1.406919784	0.001378379
ENSMUSG00000027496	Aurka	-1.410712647	8.02E-113
ENSMUSG00000042793	Lgr6	-1.412888165	2.19E-38
ENSMUSG00000044927	H1f10	-1.413170913	1.45E-22
ENSMUSG00000034329	Brip1	-1.414006636	3.61E-27
ENSMUSG00000041515	Irf8	-1.414029387	9.66E-15
ENSMUSG00000074994	Qser1	-1.414382202	1.24E-153
ENSMUSG00000097430	Gm10544	-1.41580398	0.000319399
ENSMUSG00000042807	Hecw2	-1.416267784	3.62E-55
ENSMUSG00000117422	CJ186046Rik	-1.416704679	0.03789599
ENSMUSG00000017146	Brca1	-1.416750781	2.80E-34
ENSMUSG00000028312	Smc2	-1.418000898	5.42E-168
ENSMUSG00000035237	Lcat	-1.418162988	0.000148607
ENSMUSG00000041809	Efhc1	-1.418567521	0.001024571
ENSMUSG00000106864	Gtf3c2	-1.418841913	3.14E-175
ENSMUSG00000087060	Eldr	-1.418973398	0.000284073
ENSMUSG00000050671	Ism2	-1.419132504	0.005252574
ENSMUSG00000031543	Ank1	-1.419166017	7.94E-37
ENSMUSG00000082229	Nap1l2	-1.419575403	0.018236113
ENSMUSG00000030929	Eri2	-1.42019373	1.65E-52

ENSMUSG00000099032	Tcf24	-1.420302851	1.14E-09
ENSMUSG00000017550	Atad5	-1.42239727	2.10E-24
ENSMUSG00000025665	Rps6ka6	-1.422684565	0.008048781
ENSMUSG00000029925	Tbxas1	-1.422878052	0.019707942
ENSMUSG00000062309	Rpp25	-1.423676587	1.04E-09
ENSMUSG00000026605	Cenpf	-1.424719862	7.40E-109
ENSMUSG00000079283	2310009B15Rik	-1.427198151	8.63E-12
ENSMUSG00000045539	Sprr3	-1.428852663	0.001197616
ENSMUSG00000039217	Il18	-1.429044639	0.009162718
ENSMUSG00000010476	Ebf3	-1.429501088	2.21E-60
ENSMUSG00000054206	Gzmm	-1.42956688	0.003412278
ENSMUSG00000045767	B230219D22Rik	-1.430317346	6.36E-217
ENSMUSG00000028873	Cdca8	-1.431365635	3.08E-77
ENSMUSG00000032400	Zwilch	-1.433534747	6.36E-60
ENSMUSG00000001249	Hpn	-1.43377139	0.018721206
ENSMUSG00000030255	Sspn	-1.433809038	5.70E-50
ENSMUSG00000038677	Scube3	-1.434606503	0.017472266
ENSMUSG00000055240	Zfp101	-1.434729279	2.81E-41
ENSMUSG00000074500	Zfp558	-1.434869846	0.000435163
ENSMUSG00000026582	Sele	-1.436344427	5.55E-54
ENSMUSG00000004952	Rasa4	-1.436360908	4.68E-49
ENSMUSG00000037010	Apln	-1.436479022	3.63E-77
ENSMUSG00000042043	Tbca	-1.437864754	4.05E-127
ENSMUSG00000037375	Hhat	-1.438015985	1.27E-20
ENSMUSG00000109086	Gm5596	-1.438683404	0.029828466
ENSMUSG00000095865	Gm13237	-1.439071753	0.048335889
ENSMUSG00000020086	Macroh2a2	-1.439566147	2.10E-35
ENSMUSG00000050675	Gp1ba	-1.439688889	0.001828007
ENSMUSG00000019122	Ccl9	-1.441253516	1.38E-71
ENSMUSG00000068748	Ptprz1	-1.443054364	1.07E-05
ENSMUSG00000027598	Itch	-1.445655467	5.30E-147
ENSMUSG00000043110	Lrrn4	-1.445800308	1.56E-86
ENSMUSG00000033762	Recql4	-1.445986489	4.31E-12
ENSMUSG00000067818	Myl9	-1.445990759	1.07E-119
ENSMUSG00000096472	Cdkn2d	-1.446607074	1.50E-37
ENSMUSG00000040195	Nemp1	-1.447212145	2.71E-39
ENSMUSG00000097391	Mirg	-1.447450908	4.66E-09
ENSMUSG00000023919	Cenpq	-1.447579605	4.31E-46
ENSMUSG00000020023	Tmcc3	-1.448369538	7.33E-65
ENSMUSG00000057897	Camk2b	-1.45063833	7.84E-10
ENSMUSG00000090257	Gm4524	-1.450834062	0.005572148
ENSMUSG00000037474	Dtl	-1.451226059	2.80E-26
ENSMUSG00000116597	Gm536	-1.453075471	0.00418953
ENSMUSG00000089736	Tgfbr3l	-1.453151359	0.024559339
ENSMUSG00000052949	Rnf157	-1.454776491	3.63E-35
ENSMUSG00000028175	Depdc1a	-1.455012886	9.11E-67
ENSMUSG00000039994	Timeless	-1.455481115	1.96E-28
ENSMUSG00000029910	Mad2l1	-1.455700622	2.93E-113
ENSMUSG00000034040	Galnt17	-1.456227978	2.68E-49
ENSMUSG00000017716	Birc5	-1.45713018	1.14E-93
ENSMUSG00000020974	Pole2	-1.457511697	6.54E-27
ENSMUSG00000028399	Ptprd	-1.459954046	3.05E-95
ENSMUSG00000019303	Psmc3ip	-1.462147577	1.74E-13
ENSMUSG00000021798	Ldb3	-1.463229321	1.08E-20
ENSMUSG00000086389	Gm15998	-1.463268457	0.0147476
ENSMUSG00000005583	Mef2c	-1.463497871	1.64E-34
ENSMUSG00000027612	Mmp24	-1.463610594	0.00637739
ENSMUSG00000027329	Spef1	-1.464055814	1.31E-27
ENSMUSG00000055148	Klf2	-1.465129125	8.07E-25
ENSMUSG00000034881	Tbxa2r	-1.46514988	1.09E-08
ENSMUSG00000100975	Gm28875	-1.466292611	7.10E-10
ENSMUSG00000000028	Cdc45	-1.466856488	2.79E-46
ENSMUSG00000020475	Pgam2	-1.467727895	8.15E-10
ENSMUSG00000034349	Smc4	-1.467918572	2.83E-287
ENSMUSG00000024660	Incenp	-1.469084655	7.60E-131
ENSMUSG00000042213	Zfand4	-1.469735186	1.13E-05
ENSMUSG00000044201	Cdc25c	-1.471295806	1.44E-41
ENSMUSG00000027309	4930402H24Rik	-1.47182324	5.01E-242
ENSMUSG00000037108	Zcwpw1	-1.472247425	0.029232489
ENSMUSG00000112346	Gm48768	-1.472320956	0.010100912
ENSMUSG00000095115	Itpripl2	-1.472409761	2.80E-238
ENSMUSG00000042961	Egflam	-1.473477306	0.00146354
ENSMUSG00000043635	Adamts3	-1.473806078	0.001031554
ENSMUSG00000022220	Adcy4	-1.474992222	4.11E-48
ENSMUSG00000038418	Egr1	-1.476696291	6.22E-168
ENSMUSG00000049866	Arl4c	-1.477377951	1.13E-49
ENSMUSG00000113831	Gm49602	-1.478642637	0.001803904

ENSMUSG00000103532	Gm4430	-1.479301405	0.042345871
ENSMUSG00000024598	Fbn2	-1.479727142	2.15E-06
ENSMUSG00000021373	Cap2	-1.481978124	5.57E-32
ENSMUSG00000033715	Akr1c14	-1.482095307	4.30E-32
ENSMUSG00000042812	Foxf1	-1.482206639	4.84E-06
ENSMUSG00000035142	Nubpl	-1.482258812	1.11E-15
ENSMUSG00000031636	Pdlim3	-1.482356907	6.53E-64
ENSMUSG00000033419	Snap91	-1.482958682	0.01168209
ENSMUSG00000029101	Rgs12	-1.485682246	4.18E-158
ENSMUSG00000042029	Ncapg2	-1.486230742	7.63E-150
ENSMUSG00000091568	Gm8206	-1.487091574	0.01054639
ENSMUSG00000051537	Gm5124	-1.487326233	0.0004027
ENSMUSG00000042489	Clspn	-1.487662968	5.57E-69
ENSMUSG00000021286	Zfyve21	-1.489392683	1.90E-42
ENSMUSG00000006585	Cdt1	-1.489490026	5.47E-33
ENSMUSG00000042988	Notum	-1.489599368	0.000713708
ENSMUSG00000022322	Shcbp1	-1.48970962	6.94E-89
ENSMUSG00000031553	Adam3	-1.491744925	0.005666475
ENSMUSG00000054932	Afp	-1.492488622	0.029642842
ENSMUSG00000004891	Nes	-1.49406776	3.60E-236
ENSMUSG00000041431	Ccnb1	-1.494288935	2.50E-165
ENSMUSG00000024989	Cep55	-1.495844767	6.74E-132
ENSMUSG00000029093	Sorcs2	-1.496671619	2.67E-05
ENSMUSG00000086528	Gm15731	-1.496964815	0.001452113
ENSMUSG00000073434	Wdr90	-1.496994723	1.76E-28
ENSMUSG00000051220	Ercc6l	-1.497486055	1.13E-41
ENSMUSG00000022676	Snai2	-1.498154997	4.55E-85
ENSMUSG00000019942	Cdk1	-1.498343172	4.63E-167
ENSMUSG00000024136	Dnase1l2	-1.499084629	0.019686547
ENSMUSG00000024056	Ndc80	-1.499166177	3.56E-60
ENSMUSG00000036040	Adamtsl2	-1.500014176	4.95E-21
ENSMUSG00000027330	Cdc25b	-1.500575299	1.48E-70
ENSMUSG00000053414	Hunk	-1.500830397	0.002870916
ENSMUSG00000074802	Gas2l3	-1.502025439	7.62E-52
ENSMUSG00000113570	Gm5953	-1.502572623	0.038096459
ENSMUSG00000021540	Smad5	-1.502704869	1.56E-177
ENSMUSG00000027075	Slc43a1	-1.503319687	2.99E-07
ENSMUSG00000073144	4930599N23Rik	-1.504172208	0.025280235
ENSMUSG00000027306	Nusap1	-1.506385143	1.43E-106
ENSMUSG00000029096	Htra3	-1.507008725	5.43E-36
ENSMUSG00000040204	Pclaf	-1.510330816	1.36E-68
ENSMUSG00000063232	Serpina11	-1.510408928	0.047917958
ENSMUSG00000031327	Chic1	-1.510628325	2.36E-24
ENSMUSG00000042106	Inka1	-1.511515683	4.86E-20
ENSMUSG00000010601	Apol7a	-1.512062752	0.00083885
ENSMUSG00000027323	Rad51	-1.512062943	5.98E-94
ENSMUSG00000026779	Mastl	-1.512554021	1.17E-84
ENSMUSG00000020330	Hmmr	-1.513110863	2.25E-132
ENSMUSG00000074476	Spc24	-1.513683718	1.20E-47
ENSMUSG00000026955	Sapcd2	-1.514033492	1.04E-20
ENSMUSG00000054320	Lrrc36	-1.514062384	0.041273691
ENSMUSG00000027635	Dsn1	-1.515133357	3.05E-34
ENSMUSG00000004085	Map3k20	-1.516091066	4.28E-138
ENSMUSG00000022686	B3gnt5	-1.516338679	2.54E-05
ENSMUSG00000004099	Dnmt1	-1.516583631	3.02E-185
ENSMUSG00000079056	Kcnip3	-1.516691642	0.000167354
ENSMUSG00000037685	Atp8a1	-1.51736193	3.74E-14
ENSMUSG00000025789	St8sia2	-1.517592712	1.93E-07
ENSMUSG00000022033	Pbk	-1.51769779	3.47E-87
ENSMUSG00000027115	Kif18a	-1.517840079	3.12E-33
ENSMUSG00000022602	Arc	-1.517956764	2.42E-26
ENSMUSG00000002346	Slc25a42	-1.518449683	5.74E-20
ENSMUSG00000071648	Rom1	-1.519021694	1.36E-21
ENSMUSG00000051043	Gprc5c	-1.519332309	0.003770938
ENSMUSG00000026874	Hc	-1.523065376	1.17E-05
ENSMUSG00000046561	Arsj	-1.523448433	1.16E-36
ENSMUSG00000032113	Chek1	-1.5250864	1.61E-21
ENSMUSG00000035493	Tgfbi	-1.526854723	6.33E-81
ENSMUSG00000072980	Oip5	-1.527177563	4.04E-19
ENSMUSG00000025395	Prim1	-1.527194019	4.10E-49
ENSMUSG00000022546	Gpt	-1.528713716	7.06E-10
ENSMUSG00000037725	Ckap2	-1.528804918	1.31E-180
ENSMUSG00000031099	Smarca1	-1.528974856	2.22E-10
ENSMUSG00000041193	Pla2g5	-1.531895425	0.004178723
ENSMUSG00000056579	Tug1	-1.53202427	1.72E-223
ENSMUSG00000037313	Tacc3	-1.532544553	1.80E-141
ENSMUSG00000019961	Tmpo	-1.532586443	4.45E-225

ENSMUSG00000005470	Asf1b	-1.532751261	5.58E-86
ENSMUSG00000023505	Cdca3	-1.533387706	1.87E-80
ENSMUSG00000018451	6330403K07Rik	-1.534624372	0.021360578
ENSMUSG00000022421	Nptxr	-1.537675671	1.95E-21
ENSMUSG00000031637	Lrp2bp	-1.539888339	0.00043189
ENSMUSG00000022483	Col2a1	-1.540507159	2.96E-08
ENSMUSG00000031712	Il15	-1.540533254	4.43E-15
ENSMUSG00000024791	Cdca5	-1.540535781	5.26E-38
ENSMUSG00000030616	Sytl2	-1.541855635	1.46E-33
ENSMUSG00000028782	Adgrb2	-1.542266909	7.54E-06
ENSMUSG00000026708	Cenpl	-1.545145176	1.74E-36
ENSMUSG00000026960	Arl6ip6	-1.545412892	1.81E-53
ENSMUSG00000063388	BC023105	-1.545884682	0.003438896
ENSMUSG00000039735	Fnbp1l	-1.546896236	3.93E-69
ENSMUSG00000054115	Skp2	-1.547478858	3.11E-48
ENSMUSG00000048922	Cdca2	-1.552224091	2.74E-52
ENSMUSG00000026646	Suv39h2	-1.553275652	2.70E-20
ENSMUSG00000037166	Ppp1r14a	-1.55341253	9.39E-12
ENSMUSG00000013663	Pten	-1.554049406	0
ENSMUSG00000027253	Lrp4	-1.555733336	3.99E-21
ENSMUSG00000038252	Ncapd2	-1.561700054	1.22E-223
ENSMUSG00000112794	Gm48878	-1.563716783	5.12E-05
ENSMUSG00000022422	Dscc1	-1.564741818	1.36E-08
ENSMUSG00000078762	Haus5	-1.565188939	6.66E-17
ENSMUSG00000029177	Cenpa	-1.567422599	3.70E-136
ENSMUSG00000028832	Stmn1	-1.569095018	8.18E-131
ENSMUSG00000050394	Armcx6	-1.57027145	2.29E-28
ENSMUSG00000021057	Akap5	-1.571930661	3.84E-33
ENSMUSG00000031965	Tbx20	-1.572909191	0
ENSMUSG00000040852	Plekhh2	-1.573277558	2.45E-149
ENSMUSG00000007097	Atp1a2	-1.574275999	1.43E-05
ENSMUSG00000027379	Bub1	-1.574332894	9.14E-102
ENSMUSG00000024811	Tnks2	-1.574404398	0
ENSMUSG00000094388	Gm8783	-1.575400827	1.79E-11
ENSMUSG00000005950	P2rx5	-1.575922066	1.98E-39
ENSMUSG00000025272	Tro	-1.576401689	1.32E-18
ENSMUSG00000026484	Rnf2	-1.57658474	2.44E-116
ENSMUSG00000031805	Jak3	-1.577386575	4.30E-35
ENSMUSG00000039155	Cdh26	-1.580350985	0.001648076
ENSMUSG00000027508	Pag1	-1.582398165	3.48E-45
ENSMUSG00000054717	Hmgb2	-1.583215742	3.84E-123
ENSMUSG00000032596	Uba7	-1.584760455	4.54E-62
ENSMUSG00000027353	Mcm8	-1.585912122	5.39E-20
ENSMUSG00000075592	Nynrin	-1.587782297	7.90E-107
ENSMUSG00000090363	Gm3512	-1.588879619	0.000151524
ENSMUSG00000035683	Melk	-1.589256164	4.28E-62
ENSMUSG00000034799	Unc13a	-1.591020881	6.09E-11
ENSMUSG00000061048	Cdh3	-1.59111106	0.000351312
ENSMUSG00000030641	Ddias	-1.592459725	7.61E-73
ENSMUSG00000021055	Esr2	-1.59270447	0.009812244
ENSMUSG00000041219	Arhgap11a	-1.595923118	1.46E-212
ENSMUSG00000059900	Tmem40	-1.596171388	0.004312973
ENSMUSG00000046314	Stxbp6	-1.598169484	8.34E-09
ENSMUSG00000026458	Ppfia4	-1.600932762	0.032477076
ENSMUSG00000070713	Gm10282	-1.601226214	0.000275017
ENSMUSG00000032586	Traip	-1.601982496	5.51E-24
ENSMUSG00000067860	Zic3	-1.60351422	0.019319242
ENSMUSG00000003934	Efnb3	-1.603574741	0.005633378
ENSMUSG00000035365	Parpbp	-1.603616272	9.82E-29
ENSMUSG00000001228	Uhrf1	-1.604161712	8.90E-93
ENSMUSG00000041147	Brca2	-1.606120906	7.88E-35
ENSMUSG00000097451	Rian	-1.606201835	1.01E-214
ENSMUSG00000030677	Kif22	-1.609557152	1.18E-148
ENSMUSG00000027699	Ect2	-1.609558197	1.83E-164
ENSMUSG00000101236	Gm6750	-1.610325615	0.023083408
ENSMUSG00000041482	Piezo2	-1.61331057	1.38E-34
ENSMUSG00000071112	Spx	-1.613839833	0.019248324
ENSMUSG00000036264	Fstl4	-1.6139162	0.000157993
ENSMUSG00000032667	Pon2	-1.614754325	1.11E-151
ENSMUSG00000041498	Kif14	-1.614939663	1.06E-54
ENSMUSG00000034883	Lrr1	-1.616771478	2.40E-12
ENSMUSG00000041552	Ptchd1	-1.619208834	3.67E-06
ENSMUSG00000020900	Myh10	-1.619880634	0
ENSMUSG00000025574	Tk1	-1.619882535	1.91E-97
ENSMUSG00000001349	Cnn1	-1.620625824	1.12E-06
ENSMUSG00000046295	Ankle1	-1.621363139	2.48E-22
ENSMUSG00000038486	Sv2a	-1.621724348	6.74E-10

ENSMUSG00000097789	Gm2115	-1.625647964	7.35E-11
ENSMUSG00000028678	Kif2c	-1.626349054	7.49E-138
ENSMUSG00000005268	Prlr	-1.626460273	0.003921645
ENSMUSG00000024795	Kif20b	-1.62967823	1.17E-138
ENSMUSG00000026039	Sgo2a	-1.630331004	6.69E-69
ENSMUSG00000030830	Itgal	-1.630537458	0.007865557
ENSMUSG00000105748	Gm43088	-1.631373481	0.034385439
ENSMUSG00000018983	E2f2	-1.631819228	4.70E-10
ENSMUSG00000034023	Fancd2	-1.632424965	7.96E-38
ENSMUSG00000022836	Mylk	-1.632844221	3.55E-29
ENSMUSG00000023940	Sgo1	-1.632850908	2.36E-69
ENSMUSG00000021594	Srd5a1	-1.632980912	5.11E-17
ENSMUSG00000020542	Myocd	-1.634245776	0.00036084
ENSMUSG00000033597	Caskin1	-1.634668478	0.010234888
ENSMUSG00000067780	Pi15	-1.636726066	1.47E-139
ENSMUSG00000012443	Kif11	-1.636820352	1.76E-175
ENSMUSG00000029648	Flt1	-1.639206786	4.45E-59
ENSMUSG00000103945	Gm38228	-1.643855419	0.037290515
ENSMUSG00000062609	Kcnj15	-1.646540322	1.37E-69
ENSMUSG00000032172	Olfm2	-1.647561792	4.61E-13
ENSMUSG00000071724	Smpd5	-1.649513926	0.006147789
ENSMUSG00000046807	Lrrc75b	-1.649822183	2.56E-20
ENSMUSG00000118138	Gm50322	-1.649918457	6.20E-06
ENSMUSG00000112980	D430020J02Rik	-1.651834738	1.44E-31
ENSMUSG00000037628	Cdkn3	-1.652405077	2.54E-37
ENSMUSG00000009654	Oit3	-1.65310065	4.13E-10
ENSMUSG00000030042	Pole4	-1.65329918	8.02E-106
ENSMUSG00000021572	Cep72	-1.655485902	7.52E-18
ENSMUSG00000007122	Casq1	-1.656499691	0.000319024
ENSMUSG00000001517	Foxm1	-1.657069746	2.86E-195
ENSMUSG00000020914	Top2a	-1.658277494	0
ENSMUSG00000021613	Hapln1	-1.658943383	1.32E-13
ENSMUSG00000026414	Tnnt2	-1.665044119	8.98E-142
ENSMUSG00000041703	Zic5	-1.666237596	0.004667724
ENSMUSG00000022309	Angpt1	-1.666825644	3.42E-12
ENSMUSG00000017499	Cdc6	-1.669852165	5.34E-48
ENSMUSG00000079067	Hmgn2-ps1	-1.670579833	2.80E-05
ENSMUSG00000034522	Zfp395	-1.671818999	6.69E-14
ENSMUSG00000033350	Chst2	-1.672379327	8.59E-47
ENSMUSG00000103726	Gm30074	-1.673084668	0.001456204
ENSMUSG00000003038	Hmgn2	-1.673230151	5.55E-262
ENSMUSG00000015880	Ncapg	-1.673359608	1.32E-125
ENSMUSG00000035455	Fignl1	-1.673553519	1.26E-54
ENSMUSG00000075304	Sp5	-1.673927667	0.004185328
ENSMUSG00000021391	Cenpp	-1.673964733	8.49E-12
ENSMUSG00000021782	Dlg5	-1.674573125	8.83E-172
ENSMUSG00000100980	Gm29100	-1.677887125	0.000171613
ENSMUSG00000037419	Endod1	-1.679824223	1.71E-166
ENSMUSG00000112808	Gm4739	-1.680559052	0.022297613
ENSMUSG00000079491	H2-T10	-1.681007324	2.39E-07
ENSMUSG00000047757	Fancb	-1.681204382	1.91E-28
ENSMUSG00000073733	Cplane2	-1.682227456	0.000346598
ENSMUSG00000034311	Kif4	-1.684777505	1.86E-127
ENSMUSG00000027654	Fam83d	-1.690371447	1.86E-94
ENSMUSG00000031629	Cenpu	-1.690428696	2.90E-19
ENSMUSG00000025931	Paqr8	-1.692273044	6.56E-83
ENSMUSG00000046449	Nexmif	-1.693677343	6.61E-22
ENSMUSG00000014164	Klhl3	-1.694746646	9.69E-07
ENSMUSG00000093661	Eif4e3	-1.69511861	1.18E-29
ENSMUSG00000005611	Mrvi1	-1.69761041	7.81E-36
ENSMUSG00000111977	Gm47163	-1.69769266	0.000330706
ENSMUSG00000072082	Ccnf	-1.699348484	1.03E-111
ENSMUSG00000024168	Tmem204	-1.699958815	4.92E-59
ENSMUSG00000022034	Esco2	-1.701099046	3.50E-36
ENSMUSG00000071042	Rasgrp3	-1.701280755	8.31E-59
ENSMUSG00000038332	Sesn1	-1.701323603	2.80E-30
ENSMUSG00000044469	Tnfaip8l1	-1.702388611	1.03E-10
ENSMUSG00000039376	Synpo2l	-1.704018519	0.028223368
ENSMUSG00000041406	BC055324	-1.704734692	2.82E-45
ENSMUSG00000064043	Trerf1	-1.706453692	6.56E-39
ENSMUSG00000039187	Fanci	-1.706521835	2.38E-34
ENSMUSG00000029298	Gbp9	-1.70734712	3.53E-18
ENSMUSG00000042115	Klhdc8a	-1.707419769	5.10E-92
ENSMUSG00000004473	Clec11a	-1.710877326	1.41E-20
ENSMUSG00000096592	Gm15801	-1.711668042	2.18E-09
ENSMUSG00000018169	Mfng	-1.713681817	0.033797905
ENSMUSG00000034773	Hrob	-1.714465491	3.78E-32

ENSMUSG00000036768	Kif15	-1.715473421	4.53E-72
ENSMUSG00000030216	Wbp11	-1.717532389	2.46E-176
ENSMUSG00000031994	Adamts8	-1.722056028	1.04E-10
ENSMUSG00000027715	Ccna2	-1.722419301	3.29E-162
ENSMUSG00000050505	Pcdh20	-1.724661561	0.001709095
ENSMUSG00000046324	Ermp1	-1.727333748	1.51E-123
ENSMUSG00000021176	Efcab11	-1.727848787	4.46E-12
ENSMUSG00000027335	Adra1d	-1.728222022	0.000105749
ENSMUSG00000047534	Mis18bp1	-1.732728237	3.60E-67
ENSMUSG00000047369	Dnah14	-1.734119366	0.024927773
ENSMUSG00000040605	Bace2	-1.734276719	5.60E-65
ENSMUSG00000097908	4933404O12Rik	-1.736496725	1.64E-13
ENSMUSG00000022177	Haus4	-1.737863539	5.41E-34
ENSMUSG00000028132	Tlcd4	-1.739045754	3.61E-20
ENSMUSG00000051817	Sox12	-1.739175995	1.19E-11
ENSMUSG00000039396	Neil3	-1.740698517	4.29E-40
ENSMUSG00000019945	Cabcoco1	-1.741504494	5.21E-05
ENSMUSG00000040086	Tnni3k	-1.742787573	4.04E-20
ENSMUSG00000020836	Coro6	-1.744180124	3.08E-05
ENSMUSG00000050132	Sarm1	-1.744568147	0.04313612
ENSMUSG00000034024	Cct2	-1.746853563	0
ENSMUSG00000045287	Rtn4rl1	-1.753849611	4.65E-11
ENSMUSG00000024338	Psmb8	-1.753951015	4.74E-22
ENSMUSG00000029438	Bcl7a	-1.754404912	1.85E-60
ENSMUSG00000001403	Ube2c	-1.757911198	4.36E-179
ENSMUSG00000038608	Dock10	-1.757939398	2.88E-70
ENSMUSG00000029843	Slc13a4	-1.758693777	0.004229703
ENSMUSG00000023036	Pcdhgc4	-1.758809223	0.009218251
ENSMUSG00000078773	Rad54b	-1.759421497	2.89E-17
ENSMUSG00000106691	2700029L08Rik	-1.759912194	0.007103704
ENSMUSG00000052544	St6galnac3	-1.761762502	0.043263642
ENSMUSG00000032783	Troap	-1.765953946	2.34E-40
ENSMUSG00000004655	Aqp1	-1.767535451	6.59E-131
ENSMUSG00000020182	Ddc	-1.775247205	3.19E-07
ENSMUSG00000068606	Gm4841	-1.775257138	0.000412527
ENSMUSG00000020583	Matn3	-1.777936651	0.006732633
ENSMUSG00000098318	Lockd	-1.778279149	1.98E-10
ENSMUSG00000050541	Adra1b	-1.778967849	4.29E-22
ENSMUSG00000025742	Prps2	-1.782737286	1.21E-153
ENSMUSG00000024440	Pcdh12	-1.785319881	3.32E-11
ENSMUSG00000117285	Gm49863	-1.786206507	0.004594482
ENSMUSG00000054435	Gimap4	-1.790681824	0.009974925
ENSMUSG00000096727	Psmb9	-1.793266416	1.21E-06
ENSMUSG00000026622	Nek2	-1.797763223	4.86E-193
ENSMUSG00000030867	Plk1	-1.797770444	2.74E-176
ENSMUSG00000020492	Ska2	-1.79859111	1.34E-96
ENSMUSG00000030074	Gxylt2	-1.803796769	1.02E-225
ENSMUSG00000021676	Iqgap2	-1.804026412	3.02E-25
ENSMUSG00000049357	Brd8dc	-1.805364614	5.51E-05
ENSMUSG00000048327	Ckap2l	-1.807700524	1.12E-158
ENSMUSG00000021268	Meg3	-1.807770753	3.59E-118
ENSMUSG00000073600	Prob1	-1.809751628	0.000191964
ENSMUSG00000026023	Cdk15	-1.812236888	0.000282374
ENSMUSG00000046480	Scn4b	-1.813886788	0.011797366
ENSMUSG00000030541	Idh2	-1.814584998	3.77E-155
ENSMUSG00000027469	Tpx2	-1.814687159	3.03E-303
ENSMUSG00000006398	Cdc20	-1.816458846	7.22E-224
ENSMUSG00000038379	Ttk	-1.818037591	1.06E-46
ENSMUSG00000029516	Cit	-1.818292079	9.49E-68
ENSMUSG00000052353	Cemip	-1.818350203	0
ENSMUSG00000085331	Gm11274	-1.819036744	0.037284674
ENSMUSG00000038602	Slc35f1	-1.819608668	2.22E-20
ENSMUSG00000009376	Met	-1.820446442	2.92E-82
ENSMUSG00000032218	Ccnb2	-1.821774114	2.25E-131
ENSMUSG00000045328	Cenpe	-1.823146066	1.45E-269
ENSMUSG00000037624	Kcnk2	-1.824387287	2.84E-118
ENSMUSG00000018470	Kcnab3	-1.824441457	0.000234805
ENSMUSG00000020623	Map2k6	-1.826100207	1.72E-16
ENSMUSG00000021453	Gadd45g	-1.826649761	3.33E-158
ENSMUSG00000050640	Tmem150c	-1.827306313	0.008018204
ENSMUSG00000026255	Efhd1	-1.829188609	0.005721253
ENSMUSG00000028211	Trp53inp1	-1.829373258	0
ENSMUSG00000024579	Pcyox1l	-1.831215041	7.70E-33
ENSMUSG00000050503	Fbxl22	-1.831588198	1.18E-05
ENSMUSG00000047842	Diras2	-1.832376338	6.73E-15
ENSMUSG00000070044	Fam149a	-1.835455403	2.89E-121
ENSMUSG00000009394	Syn2	-1.836704945	1.94E-06

ENSMUSG00000036655	Colec11	-1.837120212	3.17E-15
ENSMUSG00000021822	Plau	-1.840418421	1.97E-234
ENSMUSG00000026211	Obsl1	-1.841145055	1.07E-182
ENSMUSG00000098090	2700099C18Rik	-1.844767711	2.74E-23
ENSMUSG00000037206	Islr	-1.844885712	4.04E-111
ENSMUSG00000093726	Gm20667	-1.845883903	8.49E-09
ENSMUSG00000027977	Ndst3	-1.853758386	8.31E-30
ENSMUSG00000032750	Gab3	-1.85791051	0.001810968
ENSMUSG00000030055	Rab43	-1.858796293	2.55E-215
ENSMUSG00000040084	Bub1b	-1.864448704	5.49E-139
ENSMUSG00000026589	Sec16b	-1.865563464	6.26E-33
ENSMUSG00000117310	Ptp4a1	-1.867575934	0
ENSMUSG00000040260	Daam2	-1.869075006	7.35E-52
ENSMUSG00000082163	Gm14276	-1.870246148	3.05E-08
ENSMUSG00000038943	Prc1	-1.870385995	0
ENSMUSG00000020674	Pxdn	-1.876755517	0
ENSMUSG00000002055	Spag5	-1.878565062	1.13E-135
ENSMUSG00000046591	Ticrr	-1.880049456	1.96E-47
ENSMUSG00000035773	Kiss1r	-1.880430057	0.002863661
ENSMUSG00000020251	Glt8d2	-1.880483582	1.32E-87
ENSMUSG00000017417	Plxdc1	-1.883527972	1.73E-06
ENSMUSG00000000386	Mx1	-1.883642592	0.001576248
ENSMUSG00000031158	Timm17b	-1.887059782	9.94E-60
ENSMUSG00000028718	Stil	-1.891328274	1.77E-74
ENSMUSG00000033752	Mnd1	-1.892658284	2.69E-07
ENSMUSG00000029414	Kntc1	-1.893085445	1.00E-121
ENSMUSG00000116207	Nnt	-1.893395645	0.026674882
ENSMUSG00000024026	Glo1	-1.894050068	1.72E-182
ENSMUSG00000029735	Tpk1	-1.894229523	9.86E-32
ENSMUSG00000025494	Sigirr	-1.895646268	0.007557779
ENSMUSG00000099250	Rn7s2	-1.896159751	0.029305299
ENSMUSG00000025701	Alox5	-1.898221057	1.16E-07
ENSMUSG00000032135	Mcam	-1.898359824	4.11E-79
ENSMUSG00000058589	Anks1b	-1.902131514	7.63E-21
ENSMUSG00000078521	Aunip	-1.903197658	3.12E-19
ENSMUSG00000032254	Kif23	-1.903403592	2.77E-213
ENSMUSG00000111656	Gm47232	-1.904708033	0.010119755
ENSMUSG00000097961	Gm27000	-1.906541901	1.60E-07
ENSMUSG00000010122	Slc47a1	-1.912247486	0.001370327
ENSMUSG00000106139	Gm30648	-1.913202577	8.72E-05
ENSMUSG00000051378	Kif18b	-1.913608571	8.06E-119
ENSMUSG00000051113	Fam71e1	-1.915199439	0.00624818
ENSMUSG00000075502	Kbtbd6	-1.919049083	1.58E-08
ENSMUSG00000043243	Fam129c	-1.920404384	0.000811916
ENSMUSG00000047658	Gal3st3	-1.92517137	2.31E-07
ENSMUSG00000021259	Cyp46a1	-1.926494046	0.000334318
ENSMUSG00000037705	Tecta	-1.931738368	0.005878351
ENSMUSG00000046585	Cfap58	-1.934046545	1.21E-06
ENSMUSG00000078956	Gm14221	-1.935443921	3.23E-06
ENSMUSG00000041688	Amot	-1.938531848	1.85E-211
ENSMUSG00000046618	Olfml2a	-1.940509443	3.12E-09
ENSMUSG00000032595	Cdhr4	-1.943316649	0.006679639
ENSMUSG00000021485	Mxd3	-1.946300843	8.86E-39
ENSMUSG00000020897	Aurkb	-1.94657567	9.54E-113
ENSMUSG00000035458	Tnni3	-1.948811761	0.03238341
ENSMUSG00000027326	Knl1	-1.951248287	1.27E-77
ENSMUSG00000019773	Fbxo5	-1.951446492	3.91E-56
ENSMUSG00000109644	0610005C13Rik	-1.953658094	0.043521496
ENSMUSG00000055210	Foxd2	-1.957398871	3.03E-07
ENSMUSG00000020493	Prr11	-1.959114394	1.14E-149
ENSMUSG00000087305	A430035B10Rik	-1.959892875	0.0440369
ENSMUSG00000048939	Atp13a5	-1.960222532	0.00353863
ENSMUSG00000069793	Slfn9	-1.96862988	4.43E-103
ENSMUSG00000086587	Gm11837	-1.970580977	0.029754013
ENSMUSG00000024459	H2-M5	-1.972591432	4.49E-05
ENSMUSG00000018126	Baiap2l2	-1.975650404	0.020930334
ENSMUSG00000118506	1700094D03Rik	-1.976917596	0.001479096
ENSMUSG00000028555	Ttc39a	-1.979025736	2.47E-13
ENSMUSG00000051457	Spn	-1.982800238	9.49E-13
ENSMUSG00000108049	Gm44168	-1.983806179	3.77E-06
ENSMUSG00000022360	Atad2	-1.983995899	3.23E-231
ENSMUSG00000025507	Pidd1	-1.984583561	2.67E-85
ENSMUSG00000021367	Edn1	-1.985192793	2.84E-134
ENSMUSG00000040310	Alx4	-1.990534971	1.39E-08
ENSMUSG00000027331	Knstrn	-1.990788997	4.16E-183
ENSMUSG00000040856	Dlk1	-1.996232756	2.10E-05
ENSMUSG00000053263	Gm12592	-1.997326846	0.000866736

ENSMUSG00000068101	Cenpm	-1.998399241	3.63E-25
ENSMUSG00000032517	Mobp	-2.001386305	0.002923064
ENSMUSG00000082676	Gm11843	-2.007859194	0.006939681
ENSMUSG00000036862	Dchs1	-2.008166144	5.71E-40
ENSMUSG00000079362	Gm43302	-2.010133785	2.00E-07
ENSMUSG00000019326	Aoc3	-2.019034309	2.92E-10
ENSMUSG00000071414	Gm6736	-2.021994154	0.039898003
ENSMUSG00000034818	Celf5	-2.023247808	4.32E-18
ENSMUSG00000078937	Cpt1b	-2.023833154	0.002085865
ENSMUSG00000000244	Tspan32	-2.02466412	3.37E-05
ENSMUSG00000038552	Fndc4	-2.025568744	2.65E-14
ENSMUSG00000044966	Fbxo48	-2.027680078	8.11E-10
ENSMUSG00000020808	Pimreg	-2.028485595	1.64E-85
ENSMUSG00000020057	Dram1	-2.030287006	4.72E-129
ENSMUSG00000020185	E2f7	-2.033494843	4.29E-78
ENSMUSG00000020787	P2rx1	-2.033652616	0.001442883
ENSMUSG00000026768	Itga8	-2.034048994	1.16E-59
ENSMUSG00000066554	Gm10167	-2.034437562	0.004418717
ENSMUSG00000041633	Kctd12b	-2.035648565	1.31E-88
ENSMUSG00000056481	Cd248	-2.0361943	1.72E-227
ENSMUSG00000043165	Lor	-2.037201903	7.25E-20
ENSMUSG00000031780	Ccl17	-2.04117765	6.22E-13
ENSMUSG00000026051	Ecrg4	-2.041259181	8.86E-102
ENSMUSG00000032841	Prr5l	-2.045888705	4.08E-99
ENSMUSG00000031756	Cenpn	-2.049116781	5.54E-58
ENSMUSG00000033544	Angptl1	-2.049274795	9.23E-06
ENSMUSG00000070348	Ccnd1	-2.05230807	0
ENSMUSG00000086537	Gnasas1	-2.054539837	0.041865692
ENSMUSG00000031004	Mki67	-2.057753237	0
ENSMUSG00000057751	Megf6	-2.061650479	0.004596048
ENSMUSG00000040270	Bach2	-2.061941319	7.93E-15
ENSMUSG00000032009	Sesn3	-2.064946906	1.39E-132
ENSMUSG00000110575	6330537M06Rik	-2.066000298	2.01E-05
ENSMUSG00000029121	Crmp1	-2.06946629	1.01E-11
ENSMUSG00000042155	Klhl23	-2.070492689	2.91E-37
ENSMUSG00000086763	Plxna4os1	-2.074111878	0.000918284
ENSMUSG00000037544	Dlgap5	-2.075049961	2.08E-181
ENSMUSG00000096039	D830030K20Rik	-2.083663671	0.027038049
ENSMUSG00000021624	Cd180	-2.090026586	8.63E-38
ENSMUSG00000034413	Neurl1b	-2.091657607	5.69E-25
ENSMUSG00000026675	Hsd17b7	-2.09222812	0
ENSMUSG00000060795	Gm13363	-2.093238902	0.003267907
ENSMUSG00000028445	Enho	-2.093790503	3.73E-08
ENSMUSG00000044313	Mab21l3	-2.094278333	2.95E-13
ENSMUSG00000033368	Trim69	-2.094872982	0.023030473
ENSMUSG00000079553	Kifc1	-2.097003264	5.21E-135
ENSMUSG00000028687	Mutyh	-2.100073713	1.97E-10
ENSMUSG00000034614	Pik3ip1	-2.103842073	4.82E-37
ENSMUSG00000078234	Klhdc7a	-2.111028124	1.42E-11
ENSMUSG00000024534	Sncaip	-2.111052942	9.20E-08
ENSMUSG00000035189	Ano4	-2.113358557	5.67E-11
ENSMUSG00000020798	Spns3	-2.123789759	0.009025507
ENSMUSG00000029167	Ppargc1a	-2.124383644	0.001330159
ENSMUSG00000056174	Col8a2	-2.13286397	0.000490986
ENSMUSG00000024525	Impa2	-2.134516321	4.87E-76
ENSMUSG00000062151	Unc13c	-2.139450532	2.55E-17
ENSMUSG00000040723	Rcsd1	-2.141001932	1.02E-261
ENSMUSG00000026196	Bard1	-2.143492535	1.62E-36
ENSMUSG00000100798	Gm19589	-2.144686958	0.030171437
ENSMUSG00000099021	Rn7s1	-2.151543853	0.029207648
ENSMUSG00000062510	Nsl1	-2.157449836	1.95E-40
ENSMUSG00000036223	Ska1	-2.158096056	4.63E-28
ENSMUSG00000004791	Pgf	-2.159991616	5.27E-36
ENSMUSG00000041923	Nol4	-2.162021484	4.69E-05
ENSMUSG00000022211	Carmil3	-2.163271497	0.026803629
ENSMUSG00000011263	Exoc3l2	-2.16452589	9.61E-06
ENSMUSG00000043372	Hexim2	-2.170616938	0.000187049
ENSMUSG00000024301	Kifc5b	-2.170940228	5.93E-63
ENSMUSG00000056531	Ccdc18	-2.171790179	2.40E-11
ENSMUSG00000108218	Olfr1372-ps1	-2.172398686	1.86E-22
ENSMUSG00000039257	Vstm2b	-2.175305613	0.041613147
ENSMUSG00000085995	Gm2788	-2.176029318	0.002853639
ENSMUSG00000037337	Map4k1	-2.181699791	0.00969906
ENSMUSG00000031137	Fgf13	-2.182558034	7.26E-26
ENSMUSG00000051517	Arhgef39	-2.18459705	4.46E-26
ENSMUSG00000036902	Neto2	-2.187855807	5.46E-24
ENSMUSG00000052160	Pld4	-2.188426043	0.003887801

ENSMUSG00000021379	Id4	-2.194956223	2.03E-24
ENSMUSG00000041064	Pif1	-2.195114604	2.99E-96
ENSMUSG00000021697	Depdc1b	-2.217180442	9.15E-22
ENSMUSG00000005357	Slc1a6	-2.22122659	4.22E-56
ENSMUSG00000068246	Apol9b	-2.225717268	6.85E-53
ENSMUSG00000057606	Colq	-2.227931754	0.000124612
ENSMUSG00000034059	Ypel4	-2.229293245	3.49E-08
ENSMUSG00000072720	Myo18b	-2.23007484	3.19E-30
ENSMUSG00000042251	Pm20d1	-2.236501576	4.01E-15
ENSMUSG00000071547	Nt5dc2	-2.246055313	8.81E-108
ENSMUSG00000014077	Chp1	-2.248232935	0
ENSMUSG00000038146	Notch3	-2.258399755	1.06E-101
ENSMUSG00000108176	Gm43965	-2.262119633	0.002155591
ENSMUSG00000025201	Bloc1s2-ps	-2.26693132	0.039102106
ENSMUSG00000085873	Ttc39aos1	-2.270934021	0.001872674
ENSMUSG00000006235	Epor	-2.278970848	3.13E-06
ENSMUSG00000025610	Map3k7cl	-2.27918121	4.93E-19
ENSMUSG00000047638	Nr1h4	-2.28576021	8.38E-71
ENSMUSG00000004880	Lbr	-2.286331351	9.24E-257
ENSMUSG00000042873	Lhfpl4	-2.287474102	6.79E-08
ENSMUSG00000085006	BC021767	-2.288780325	1.55E-06
ENSMUSG00000034457	Eda2r	-2.293126657	3.36E-12
ENSMUSG00000068859	Sp9	-2.296705807	0.000693899
ENSMUSG00000037991	Rmi2	-2.29939182	6.49E-07
ENSMUSG00000061068	Mcpt4	-2.302841586	0.001540225
ENSMUSG00000113909	Gm36377	-2.311463723	1.11E-07
ENSMUSG00000053693	Mast1	-2.314162207	0.000793732
ENSMUSG00000022353	Mtss1	-2.314191	2.25E-108
ENSMUSG00000032291	Crabp1	-2.316423612	1.09E-08
ENSMUSG00000112358	Gm33869	-2.3166748	0.003279795
ENSMUSG00000033207	Mamdc2	-2.318492423	1.67E-78
ENSMUSG00000033847	Pla2g4c	-2.320994292	8.93E-05
ENSMUSG00000032648	Pygm	-2.323718592	2.11E-16
ENSMUSG00000031351	Zfp185	-2.324210584	0.000211181
ENSMUSG00000064032	Gm10143	-2.325254963	6.64E-07
ENSMUSG00000113654	Gm30893	-2.327772379	0.029175651
ENSMUSG00000037846	Rtkn2	-2.333872073	1.92E-12
ENSMUSG00000106062	Gm43820	-2.341893593	2.72E-07
ENSMUSG00000030470	Csrp3	-2.345270593	0.026329306
ENSMUSG00000106717	Gm42798	-2.345853178	0.04859275
ENSMUSG00000033871	Ppargc1b	-2.349954614	1.92E-05
ENSMUSG00000100635	Gm29157	-2.351748707	0.020691102
ENSMUSG00000029570	Lfng	-2.359847571	3.48E-25
ENSMUSG00000036339	Tmem260	-2.371678158	1.53E-111
ENSMUSG00000038128	Camk4	-2.372088178	0.007968065
ENSMUSG00000085419	Gm11734	-2.378204618	0.013992017
ENSMUSG00000072437	Nanos1	-2.380065607	1.90E-80
ENSMUSG00000041329	Atp1b2	-2.38687397	1.32E-08
ENSMUSG00000010362	Rdm1	-2.39154672	0.000159794
ENSMUSG00000046186	Cd109	-2.399035553	0
ENSMUSG00000032698	Lmo2	-2.399418084	1.43E-111
ENSMUSG00000037652	Phc3	-2.403067927	1.23E-235
ENSMUSG00000026873	Phf19	-2.403100848	2.95E-95
ENSMUSG00000078640	Gm11627	-2.412179367	0.000948337
ENSMUSG00000054675	Tmem119	-2.41758125	2.64E-129
ENSMUSG00000030699	Tbx6	-2.425107187	0.042810205
ENSMUSG00000084797	Gm14321	-2.426721808	0.026100876
ENSMUSG00000017446	C1qtnf1	-2.434968714	2.81E-99
ENSMUSG00000052485	Tmem171	-2.446901804	1.20E-14
ENSMUSG00000025666	Tmem47	-2.450754243	3.34E-171
ENSMUSG00000081769	Gm12216	-2.464367566	0.017065683
ENSMUSG00000023328	Ache	-2.469301592	0.021850016
ENSMUSG00000033849	B3galt2	-2.473608095	9.72E-08
ENSMUSG00000044365	Cxxc4	-2.474213577	2.04E-05
ENSMUSG00000111293	Gm34006	-2.479479237	0.007121674
ENSMUSG00000104965	Gm43437	-2.482427992	4.39E-06
ENSMUSG00000070425	Xntrpc	-2.493545541	1.92E-07
ENSMUSG00000023015	Racgap1	-2.507185946	7.22E-249
ENSMUSG00000042834	Nrep	-2.522641402	0
ENSMUSG00000059901	Adamts14	-2.534650221	3.26E-134
ENSMUSG00000032500	Dclk3	-2.540278053	2.45E-05
ENSMUSG00000103558	Gm38220	-2.543648578	0.033710035
ENSMUSG00000026683	Nuf2	-2.544194871	2.70E-215
ENSMUSG00000013367	Iglon5	-2.556873974	7.65E-10
ENSMUSG00000053007	Creb5	-2.562298161	1.28E-56
ENSMUSG00000030319	Cand2	-2.56301062	3.23E-211
ENSMUSG00000022821	Hgd	-2.575404519	0.034995048

ENSMUSG00000020866	Cacna1g	-2.576479909	0.003030481
ENSMUSG00000079363	Gbp4	-2.582715569	0.000564537
ENSMUSG00000034687	Fras1	-2.59412095	0.003896928
ENSMUSG00000021448	Shc3	-2.599569421	8.94E-10
ENSMUSG00000037995	Igsf9	-2.600336017	7.52E-16
ENSMUSG00000020905	Usp43	-2.600643669	0.029958096
ENSMUSG00000048485	Zbtb8b	-2.602316452	2.06E-12
ENSMUSG00000044461	Shisa2	-2.602982222	3.52E-08
ENSMUSG00000013611	Snx31	-2.618677369	0.011008548
ENSMUSG00000047819	Tigd4	-2.628379762	0.024191127
ENSMUSG00000082292	Gm12250	-2.630476674	0.000286717
ENSMUSG00000111857	1190001M18Rik	-2.630566484	0.000853571
ENSMUSG00000020155	Kcnmb1	-2.646076508	2.03E-12
ENSMUSG00000050666	Vstm4	-2.651749206	2.34E-06
ENSMUSG00000085091	Egfros	-2.662917456	3.96E-05
ENSMUSG00000049241	Hcar1	-2.664350161	2.28E-05
ENSMUSG00000026548	Slamf9	-2.67481495	3.74E-22
ENSMUSG00000029861	Fam131b	-2.675010355	1.16E-16
ENSMUSG00000038526	Car14	-2.694302384	0.029935115
ENSMUSG00000048574	Ccnb1-ps	-2.719591218	0.006773822
ENSMUSG00000109056	A630009H07Rik	-2.727007184	0.000468895
ENSMUSG00000103569	Gm38067	-2.727053872	0.048971399
ENSMUSG00000039899	Fgl2	-2.729308233	3.05E-200
ENSMUSG00000026715	Serpinc1	-2.731681527	0.031895917
ENSMUSG00000033952	Aspm	-2.744311731	3.04E-256
ENSMUSG00000097519	4930558J18Rik	-2.75711598	9.28E-07
ENSMUSG00000045102	Poln	-2.7630367	6.93E-06
ENSMUSG00000009281	Rarres2	-2.773403126	2.02E-12
ENSMUSG00000027489	Necab3	-2.778263406	0.008450839
ENSMUSG00000057346	Apol9a	-2.780025644	9.94E-27
ENSMUSG00000109134	Gm45076	-2.784742818	0.000754162
ENSMUSG00000025076	Casp7	-2.795319077	6.90E-115
ENSMUSG00000046613	Vwa5b2	-2.798421176	0.049183262
ENSMUSG00000053646	Plxnb1	-2.804362854	9.23E-21
ENSMUSG00000097495	Gm26651	-2.80684484	0.003353791
ENSMUSG00000022464	Slc38a4	-2.807544448	8.17E-60
ENSMUSG00000038540	Tmc3	-2.812862908	2.04E-07
ENSMUSG00000053024	Cntn2	-2.822246758	1.96E-13
ENSMUSG00000051726	Kcnf1	-2.833231941	1.43E-07
ENSMUSG00000039556	Ppp1r3f	-2.834699041	1.94E-17
ENSMUSG00000112716	4930471E19Rik	-2.845863934	0.012344301
ENSMUSG00000048126	Col6a3	-2.847555269	0
ENSMUSG00000028011	Tdo2	-2.860987696	0.01460082
ENSMUSG00000031778	Cx3cl1	-2.862099871	0
ENSMUSG00000035383	Pmch	-2.866708148	0.017712094
ENSMUSG00000039814	Xkr5	-2.882533558	1.08E-16
ENSMUSG00000108592	Gm38973	-2.885850158	0.037155911
ENSMUSG00000073295	Nudt11	-2.895484097	2.37E-16
ENSMUSG00000050014	Apol10b	-2.904858868	0.000463323
ENSMUSG00000015452	Ager	-2.925689608	0.002735338
ENSMUSG00000117964	Gm36043	-2.935970576	0.023154828
ENSMUSG00000043496	Tril	-2.948358923	1.07E-44
ENSMUSG00000041670	Rims1	-2.96477021	0.001140542
ENSMUSG00000046908	Ltb4r1	-2.965899247	0.003565445
ENSMUSG00000078922	Tgtp1	-2.973597608	2.84E-19
ENSMUSG00000026564	Dusp27	-2.984130646	1.81E-44
ENSMUSG00000036306	Lzts1	-2.988488876	1.27E-07
ENSMUSG00000038224	Serpinf2	-3.004216479	0.00500158
ENSMUSG00000026126	Ptpn18	-3.030163025	0.000352006
ENSMUSG00000052854	Nrk	-3.042944879	1.27E-104
ENSMUSG00000055027	Smyd1	-3.049568258	3.48E-09
ENSMUSG00000079355	Ackr4	-3.060954477	6.80E-111
ENSMUSG00000024155	Meiob	-3.069224619	0.000658488
ENSMUSG00000103049	Gm37311	-3.079152495	0.019066304
ENSMUSG00000048402	Gli2	-3.079874457	0.048235283
ENSMUSG00000068522	Aard	-3.088645895	1.08E-08
ENSMUSG00000029838	Ptn	-3.090473629	0
ENSMUSG00000034855	Cxcl10	-3.091942194	5.37E-05
ENSMUSG00000112932	Gm48308	-3.102022655	0.000117115
ENSMUSG00000040253	Gbp7	-3.131228552	1.15E-21
ENSMUSG00000078921	Tgtp2	-3.153563617	5.30E-12
ENSMUSG00000022032	Scara5	-3.194434172	4.45E-22
ENSMUSG00000110299	Gm17910	-3.195173576	0.014469381
ENSMUSG00000029102	Hgfac	-3.202682068	0.011035923
ENSMUSG00000092242	Gm20515	-3.242325214	0.04146467
ENSMUSG00000021200	Asb2	-3.263462779	0.000154329
ENSMUSG00000114664	Gm48639	-3.271659133	0.0147476

ENSMUSG00000079173	Zan	-3.307264523	0.004469638
ENSMUSG00000057880	Abat	-3.327448689	3.71E-207
ENSMUSG00000047146	Tet1	-3.338842019	5.37E-26
ENSMUSG00000055489	Ano5	-3.351201724	0.029781479
ENSMUSG00000036295	Lrrn3	-3.352029051	1.01E-14
ENSMUSG00000033590	Myo5c	-3.359229151	7.57E-07
ENSMUSG00000025422	Agap2	-3.361477569	0.017856024
ENSMUSG00000097993	Ptprv	-3.365119566	0.007391046
ENSMUSG00000092192	Dnaaf4	-3.384429709	0.049519025
ENSMUSG00000104517	Gm18407	-3.397327712	0.03907075
ENSMUSG00000045534	Kcna5	-3.399628347	2.37E-16
ENSMUSG00000038094	Atp13a4	-3.473683344	0.028883255
ENSMUSG00000079588	Tmem182	-3.51280676	7.86E-21
ENSMUSG00000001663	Gstt1	-3.528296379	1.10E-55
ENSMUSG00000051920	Rspo2	-3.545150551	5.68E-07
ENSMUSG00000045733	Sprn	-3.554430602	9.61E-05
ENSMUSG00000020017	Hal	-3.586478792	0.020048914
ENSMUSG00000030310	Slc6a1	-3.631491262	0.001772685
ENSMUSG00000113334	D030007L05Rik	-3.639169984	0.011129955
ENSMUSG00000114409	Gm9042	-3.650686211	0.015334407
ENSMUSG00000079304	Tex52	-3.65999858	0.02604823
ENSMUSG00000090394	4930523C07Rik	-3.698313844	1.01E-231
ENSMUSG00000118607	AC147806.2	-3.725086106	0.004232046
ENSMUSG00000086103	Gm11832	-3.728316496	0.023684628
ENSMUSG00000086584	Gm12002	-3.746183635	0.009342968
ENSMUSG00000079681	Zglp1	-3.746874135	0.024591782
ENSMUSG00000082196	Gm14231	-3.806220421	0.000654373
ENSMUSG00000045087	S1pr5	-3.813458856	8.38E-06
ENSMUSG00000027875	Hmgcs2	-3.860981001	3.79E-09
ENSMUSG00000019737	Syne4	-3.863485883	0.003011375
ENSMUSG00000030048	Gkn3	-3.879247547	0.000900804
ENSMUSG00000066170	E230001N04Rik	-3.98736948	0.002910534
ENSMUSG00000035407	Kank4	-3.997228879	2.69E-06
ENSMUSG00000079679	Vwde	-4.011163418	0.001255806
ENSMUSG00000023070	Rgn	-4.018136901	0.002568971
ENSMUSG00000002324	Rec8	-4.02237152	0.000129462
ENSMUSG00000035283	Adrb1	-4.053517611	1.35E-10
ENSMUSG00000058396	Gpr182	-4.059372115	7.87E-76
ENSMUSG00000003872	Lin7b	-4.137503386	0.005986683
ENSMUSG00000090125	Pou3f1	-4.142440253	0.000484449
ENSMUSG00000041205	Map6d1	-4.182973686	7.66E-07
ENSMUSG00000116777	Zfp520-ps	-4.258139391	0.026110411
ENSMUSG00000015947	Fcgr1	-4.259353728	0.000952781
ENSMUSG00000104507	A430027H14Rik	-4.334298368	0.000217475
ENSMUSG00000043020	Wdr63	-4.3727406	0.020318216
ENSMUSG00000029685	Asb15	-4.378460214	4.08E-10
ENSMUSG00000039304	Tnfsf10	-4.401016847	4.22E-13
ENSMUSG00000030317	Timp4	-4.409178765	0.003896928
ENSMUSG00000025189	Cnnm1	-4.518046312	0.00011194
ENSMUSG00000103357	Gm7804	-4.635550109	0.043329718
ENSMUSG00000107620	Gm44256	-4.729337907	0.038963431
ENSMUSG00000070644	Etnk2	-4.894899219	3.08E-15
ENSMUSG00000046999	1110032F04Rik	-5.056511242	3.88E-46
ENSMUSG00000084088	Gm12941	-5.181853936	0.026383568
ENSMUSG00000107443	Gm7468	-5.223544192	0.007733293
ENSMUSG00000118655	AC156032.1	-5.246534546	0.049988532
ENSMUSG00000026224	4933407L21Rik	-5.257520979	0.042294401
ENSMUSG00000113670	Gm49686	-5.258261927	0.007383277
ENSMUSG00000085881	Gm15912	-5.262281413	0.044004058
ENSMUSG00000108332	D530033B14Rik	-5.311484436	0.037891866
ENSMUSG00000038236	Hoxa7	-5.31386259	0.036882385
ENSMUSG00000102897	Gm37853	-5.317753453	0.037381349
ENSMUSG00000083223	Gm15016	-5.317838366	0.035600457
ENSMUSG00000051596	Otop1	-5.325671831	0.035914043
ENSMUSG00000082630	Gm14834	-5.325671831	0.035914043
ENSMUSG00000082964	Rpl13-ps5	-5.326787268	0.045118451
ENSMUSG00000091002	Tcerg1l	-5.371983224	0.032747143
ENSMUSG00000097069	Gm16998	-5.383261636	0.033410048
ENSMUSG00000117079	Gm41611	-5.383493188	0.031027632
ENSMUSG00000087198	Tmem274	-5.385671514	0.029987818
ENSMUSG00000109555	Gm44891	-5.387852233	0.030739452
ENSMUSG00000083921	Gm15750	-5.387852233	0.030739452
ENSMUSG00000096883	Shisa8	-5.431451229	0.028763489
ENSMUSG00000110787	Gm6667	-5.433641638	0.028167985
ENSMUSG00000100094	1810008I18Rik	-5.434427859	0.026663538
ENSMUSG00000002100	Mybpc3	-5.436603493	0.000312813
ENSMUSG00000003051	Elf3	-5.440183764	0.027309201

ENSMUSG00000114246	Gm48603	-5.442581349	0.026691865
ENSMUSG00000070388	Fbxo39	-5.443278674	0.026120009
ENSMUSG00000085016	Gm11335	-5.44676448	0.025913176
ENSMUSG00000097738	4930445N18Rik	-5.449632141	0.027060318
ENSMUSG00000099384	1700110C19Rik	-5.449705114	0.026851628
ENSMUSG00000031448	Adprhl1	-5.49218047	0.022461494
ENSMUSG00000071342	Lsmem1	-5.504037384	0.022192136
ENSMUSG00000102246	9430037O13Rik	-5.505525595	0.022895006
ENSMUSG00000098434	2010110E17Rik	-5.540765889	0.02857946
ENSMUSG00000017309	Cd300lg	-5.541633946	0.020436962
ENSMUSG00000038997	Asb17	-5.541633946	0.020436962
ENSMUSG00000085837	Kcnmb4os2	-5.543387215	0.045947659
ENSMUSG00000102605	Gm37264	-5.547709635	0.019053171
ENSMUSG00000022809	Nr1i2	-5.552523832	0.01832338
ENSMUSG00000025983	Ccdc150	-5.554462577	0.018234327
ENSMUSG00000024409	Psors1c2	-5.55589449	0.018678403
ENSMUSG00000014686	Ceacam16	-5.568805203	0.043674882
ENSMUSG00000036110	Slc17a2	-5.586636706	0.042034296
ENSMUSG00000044933	Sstr3	-5.592656327	0.01889443
ENSMUSG00000109729	Gm45418	-5.593396348	0.040861356
ENSMUSG00000091722	Siah3	-5.595043488	0.041038672
ENSMUSG00000090863	A530084C06Rik	-5.597982439	0.016254458
ENSMUSG00000026686	Lmx1a	-5.601180432	0.016182071
ENSMUSG00000087340	Gm15228	-5.603123102	0.016053697
ENSMUSG00000086313	Gm15940	-5.611627492	0.016232936
ENSMUSG00000059891	Tsks	-5.614780099	0.041172651
ENSMUSG00000008482	Rnf151	-5.620275897	0.038675105
ENSMUSG00000031250	Tnmd	-5.620905143	0.038739956
ENSMUSG00000019368	Sec14l4	-5.635264045	0.040009449
ENSMUSG00000010080	Epn3	-5.636789988	0.037668817
ENSMUSG00000036594	H2-Aa	-5.637202538	0.039091597
ENSMUSG00000118234	Gm50115	-5.658287343	0.035707778
ENSMUSG00000019906	Lin7a	-5.665843714	0.01523059
ENSMUSG00000087361	0610043K17Rik	-5.671189208	0.034406844
ENSMUSG00000069917	Hba-a2	-5.6749931	0.043266771
ENSMUSG00000106108	Gm43221	-5.676687901	0.038995674
ENSMUSG00000115681	Gm2682	-5.687790967	0.033058277
ENSMUSG00000046108	Il17c	-5.695899902	0.012127908
ENSMUSG00000086236	5830418P13Rik	-5.699603204	0.011579006
ENSMUSG00000044633	B530045E10Rik	-5.706396285	0.012952912
ENSMUSG00000070280	Slc22a14	-5.710599509	0.013063475
ENSMUSG00000101823	Gm29438	-5.720955295	0.030739452
ENSMUSG00000102900	Gm37811	-5.761105846	0.03046556
ENSMUSG00000058398	Prss43	-5.765650191	0.027373457
ENSMUSG00000025172	Ankrd2	-5.766787009	0.027254415
ENSMUSG00000117046	Gm19183	-5.791900269	0.025472139
ENSMUSG00000072963	Gm10447	-5.796290193	0.009092526
ENSMUSG00000111094	Gm34425	-5.812311019	0.024273054
ENSMUSG00000114860	Gm49291	-5.813125154	0.012238567
ENSMUSG00000081633	Gm8522	-5.823900755	0.023783865
ENSMUSG00000086845	Gm13010	-5.82799082	0.008859392
ENSMUSG00000086602	Gm15609	-5.8412597	0.007063266
ENSMUSG00000021999	Cpb2	-5.85752706	0.026055699
ENSMUSG00000050625	Ccdc121	-5.877690286	0.020837097
ENSMUSG00000015890	Amdhd1	-5.877829057	0.020452756
ENSMUSG00000062939	Stat4	-5.880749695	0.006345692
ENSMUSG00000050473	Slc35d3	-5.889478593	0.006638675
ENSMUSG00000110884	Gm47416	-5.890337809	0.006485547
ENSMUSG00000042567	Nek10	-5.894080986	0.022299793
ENSMUSG00000066071	Cyp4a12a	-5.894544702	0.048617219
ENSMUSG00000078650	G6pc	-5.894544702	0.048617219
ENSMUSG00000022181	C6	-5.900318484	0.022603538
ENSMUSG00000020932	Gfap	-5.920741495	0.005776695
ENSMUSG00000104677	Gm43376	-5.922360482	0.005246124
ENSMUSG00000085201	Nr6a1os	-5.923415996	0.005137321
ENSMUSG00000025082	Vwa2	-5.925468707	0.005138989
ENSMUSG00000061082	Plac1	-5.925628447	0.005631108
ENSMUSG00000030378	Sult2a8	-5.935179285	0.044893076
ENSMUSG00000029656	C8b	-5.935179285	0.044893076
ENSMUSG00000103238	Gm36527	-5.940851302	0.017386305
ENSMUSG00000083534	H2-M6-ps	-5.940851302	0.017386305
ENSMUSG00000050926	Dcaf12l2	-5.969764545	0.004929874
ENSMUSG00000031138	F9	-5.974700295	0.041490187
ENSMUSG00000105699	Gm43703	-5.976979661	0.005384862
ENSMUSG00000029369	Afm	-5.984343944	0.016535542
ENSMUSG00000089989	Gm45713	-5.991314306	0.040261386
ENSMUSG00000059406	Tmprss9	-6.000228415	0.004575154

ENSMUSG00000045231	BC106179	-6.000755408	0.015167464
ENSMUSG00000067724	Gbx1	-6.007967254	0.003754038
ENSMUSG00000043230	Fam124b	-6.01152694	0.004075305
ENSMUSG00000102850	Gm37082	-6.018202848	0.004875406
ENSMUSG00000110790	Gm47079	-6.032592531	0.013331648
ENSMUSG00000034115	Scn11a	-6.041490315	0.004474711
ENSMUSG00000093759	Scarletltr	-6.046983165	0.003543036
ENSMUSG00000005237	Dnah2	-6.051984889	0.003998489
ENSMUSG00000055730	Ces2a	-6.087153922	0.033020543
ENSMUSG00000062074	Ccn6	-6.088744471	0.003108854
ENSMUSG00000098832	Kdm4dl	-6.105747047	0.004476771
ENSMUSG00000111079	Gm47112	-6.115273783	0.002696318
ENSMUSG00000027793	Ccna1	-6.136181671	0.010229502
ENSMUSG00000021364	Elovl2	-6.140465545	0.003001942
ENSMUSG00000117477	Gm50092	-6.150339826	0.002090676
ENSMUSG00000025127	Gcgr	-6.157529826	0.0284439
ENSMUSG00000039653	Baat	-6.157529826	0.0284439
ENSMUSG00000107989	Gm19692	-6.158382874	0.00215865
ENSMUSG00000030046	Bmp10	-6.190163443	0.001785213
ENSMUSG00000118021	Gm50130	-6.19094967	0.001731202
ENSMUSG00000031173	Otc	-6.191470713	0.026413506
ENSMUSG00000116121	Gm49486	-6.205168213	0.008311633
ENSMUSG00000028784	Spocd1	-6.213958558	0.001805308
ENSMUSG00000045238	A730035I17Rik	-6.238004278	0.007389193
ENSMUSG00000002007	Srpk3	-6.24545622	0.007328383
ENSMUSG00000052142	Rasal3	-6.248837597	0.001830369
ENSMUSG00000086675	Plxna4os2	-6.255585813	0.006964773
ENSMUSG00000037780	Mbl1	-6.257046445	0.022804899
ENSMUSG00000023176	Cpn2	-6.257046445	0.022804899
ENSMUSG00000099465	Gm3830	-6.293301609	0.001259681
ENSMUSG00000025482	Odf3	-6.298822227	0.001384253
ENSMUSG00000062410	Hsd3b3	-6.300399697	0.002587006
ENSMUSG00000032691	Nlrp3	-6.316140707	0.001307005
ENSMUSG00000038132	Rbm24	-6.34605919	0.001162084
ENSMUSG00000087268	Gm14486	-6.352081662	0.000911015
ENSMUSG00000091736	Yy2	-6.352279768	2.42E-06
ENSMUSG00000022055	Nefl	-6.363318774	0.001175201
ENSMUSG00000057074	Ces1g	-6.368133157	0.001163875
ENSMUSG00000078127	Fam170b	-6.383273128	0.00101017
ENSMUSG00000058794	Nfe2	-6.415766088	0.000680716
ENSMUSG00000086150	Bach2os	-6.435078222	0.000753751
ENSMUSG00000087129	Gm16316	-6.495582314	0.000690849
ENSMUSG00000104187	Gm37820	-6.524395592	0.000359159
ENSMUSG00000064140	Trim38	-6.548502122	0.000347375
ENSMUSG00000116307	Gm49490	-6.576494008	0.000312399
ENSMUSG00000086015	4833417C18Rik	-6.607325021	0.00028759
ENSMUSG00000037542	Aldh8a1	-6.642942536	0.000300155
ENSMUSG00000066975	Cryba4	-6.769860599	0.000104848
ENSMUSG00000060212	Pcnx2	-6.775437087	0.000108352
ENSMUSG00000072673	Gm10392	-6.802691442	9.57E-05
ENSMUSG00000102418	Sh2d1b1	-6.939672265	4.55E-05
ENSMUSG00000039070	Cpa4	-6.962136175	4.94E-05
ENSMUSG00000038583	Pln	-6.974863459	4.38E-05
ENSMUSG00000060843	Ctnna3	-7.048025841	2.10E-05
ENSMUSG00000054966	Lmntd1	-7.09164854	2.49E-05
ENSMUSG00000026205	Slc23a3	-7.190560588	8.43E-06
ENSMUSG00000060371	Caln1	-8.002920522	4.22E-08
ENSMUSG00000029797	Sspo	-8.17598581	1.10E-08
ENSMUSG00000027559	Car3	-8.512608656	0.000410597
ENSMUSG00000003477	Inmt	-8.610365023	0.000838192
ENSMUSG00000010064	Slc38a3	-8.622666343	0.000511025

Supplemental Table 2. Differentially expressed genes in TGFβ-treated IAMP knockdown cardiac fibroblasts

siR-1+TGFβ vs siNC+TGFβ

ensembl_gene_id	gene_name	log2FoldChange	padj
ENSMUSG00000027364	Usp50	9.950962002	0.039624681
ENSMUSG00000097414	B130046B21Rik	3.687212718	0.005675522
ENSMUSG00000076617	Ighm	2.755842403	0.001027807
ENSMUSG00000037652	Phc3	2.697400889	5.63E-230
ENSMUSG00000020826	Nos2	2.518335659	2.57E-183
ENSMUSG00000022032	Scara5	2.388928542	1.52E-09
ENSMUSG00000046203	Sprr2g	2.300966479	4.77E-16
ENSMUSG00000112963	Gm6093	2.147851411	2.23E-07
ENSMUSG00000071036	Gm10309	2.120429554	0.002168705
ENSMUSG00000005413	Hmox1	2.090077375	0
ENSMUSG00000105852	Gm42890	2.062841341	7.04E-08
ENSMUSG00000032878	Ccdc85a	1.95926709	8.83E-11
ENSMUSG00000079092	Prl2c2	1.882360337	1.02E-42
ENSMUSG00000091968	Gm17115	1.871429378	0.003929319
ENSMUSG00000096988	A930029G22Rik	1.842623734	0.012763538
ENSMUSG00000050069	Grem2	1.832969947	4.29E-32
ENSMUSG00000055030	Sprr2e	1.821504818	1.90E-08
ENSMUSG00000023914	Mep1a	1.80363608	0.002555315
ENSMUSG00000056457	Prl2c3	1.790435578	1.01E-11
ENSMUSG00000031549	Ido2	1.728308829	0.001530204
ENSMUSG00000021700	Rab3c	1.699387473	5.66E-05
ENSMUSG00000032257	Ankk1	1.683762428	0.028956863
ENSMUSG00000062345	Serpinb2	1.675605363	1.97E-05
ENSMUSG00000099041	Gm28035	1.669383402	1.54E-05
ENSMUSG00000092021	Gbp11	1.667349446	2.62E-09
ENSMUSG00000049128	Ivl	1.661042179	7.82E-06
ENSMUSG00000078868	Gm14412	1.656020518	0.011891867
ENSMUSG00000085054	Gm15834	1.646797607	0.004665466
ENSMUSG00000028354	Fmn2	1.644781443	6.84E-05
ENSMUSG00000039579	Grin3a	1.640431461	0.000477095
ENSMUSG00000031596	Slc7a2	1.611517102	3.39E-277
ENSMUSG00000050578	Mmp13	1.610382296	2.63E-28
ENSMUSG00000049100	Pcdh10	1.593036347	0.000126925
ENSMUSG00000024331	Dsc2	1.589231031	0.005333984
ENSMUSG00000019102	Aldh3a1	1.574735331	7.54E-41
ENSMUSG00000047344	Lancl3	1.564695372	6.55E-10
ENSMUSG00000057933	Gsta2	1.54162045	0.001616692
ENSMUSG00000111709	Gm3776	1.541225662	0.000239518
ENSMUSG00000029417	Cxcl9	1.54113794	3.54E-06
ENSMUSG00000063415	Cyp26b1	1.529420328	7.87E-40
ENSMUSG00000029379	Cxcl3	1.500286006	4.62E-33
ENSMUSG00000042244	Pglyrp3	1.497095864	0.000237662
ENSMUSG00000019966	Kitl	1.482970982	3.67E-110
ENSMUSG00000117604	Gm33228	1.47841442	3.18E-05
ENSMUSG00000094786	Gm14403	1.477061262	6.61E-09
ENSMUSG00000027832	Ptx3	1.463539171	6.05E-179
ENSMUSG00000056494	Cngb3	1.455141788	6.92E-15
ENSMUSG00000109297	Gm31522	1.447512117	6.08E-06
ENSMUSG00000102780	Gm38253	1.400091363	0.007526848
ENSMUSG00000002997	Prkar2b	1.390095654	1.08E-82
ENSMUSG00000020676	Ccl11	1.371124764	0.004443002
ENSMUSG00000020264	Slc36a2	1.368326198	8.68E-12
ENSMUSG00000033196	Myh2	1.364433016	0.008829937
ENSMUSG00000020010	Vnn3	1.358616108	2.63E-12
ENSMUSG00000090942	F830016B08Rik	1.351532814	0.000203117
ENSMUSG00000078866	Zfp970	1.346360577	1.57E-43
ENSMUSG00000049511	Htr1b	1.323184644	0.000471172
ENSMUSG00000060550	H2-Q7	1.322154673	2.04E-79
ENSMUSG00000029335	Bmp3	1.308842683	2.49E-05
ENSMUSG00000051228	Nyx	1.287905883	0.029782972
ENSMUSG00000110378	Gm45242	1.28740939	0.028625848
ENSMUSG00000084996	Gm11419	1.280988929	0.049906281
ENSMUSG00000086111	Gm15326	1.276643112	0.010743433
ENSMUSG00000011008	Mcoln2	1.271272473	0.000110648
ENSMUSG00000087001	Gm15475	1.264867611	0.04842892
ENSMUSG00000021732	Fgf10	1.257546892	0.036220388
ENSMUSG00000079645	Gm17193	1.255770861	0.044265743
ENSMUSG00000090145	Ugt1a6b	1.255480082	1.64E-12
ENSMUSG00000069540	Gm4925	1.252806541	0.006941427
ENSMUSG00000049928	Glp2r	1.246343033	6.21E-08
ENSMUSG00000006345	Ggt1	1.237186824	4.37E-12
ENSMUSG00000073409	H2-Q6	1.230231328	1.40E-23
ENSMUSG00000036144	Meox2	1.221050101	3.41E-31
ENSMUSG00000056054	S100a8	1.218531193	0.017101812

siR-2+TGFβ vs siNC+TGFβ

ensembl_gene_id	gene_name	log2FoldChange	padj
ENSMUSG00000027364	Usp50	10.80381705	2.07E-17
ENSMUSG00000089865	Gm44503	4.380565066	1.01E-05
ENSMUSG00000102549	Gm38137	4.261432986	0.000230549
ENSMUSG00000097414	B130046B21Rik	3.569530649	0.001849121
ENSMUSG00000021214	Akr1c18	3.374265512	0.000326145
ENSMUSG00000026011	Ctla4	2.711402543	0.001284911
ENSMUSG00000090171	Ugt1a2	2.545385153	0.01263972
ENSMUSG00000113311	Gm47428	2.49423556	0.00297781
ENSMUSG00000050069	Grem2	2.426330702	2.97E-64
ENSMUSG00000057933	Gsta2	2.316652893	2.74E-09
ENSMUSG00000055030	Sprr2e	2.253966665	2.27E-14
ENSMUSG00000056054	S100a8	2.109415882	5.37E-07
ENSMUSG00000028354	Fmn2	2.084573738	1.74E-09
ENSMUSG00000079092	Prl2c2	2.059866782	1.10E-57
ENSMUSG00000044176	Spink10	2.018329301	3.06E-07
ENSMUSG00000111709	Gm3776	2.00608294	2.39E-08
ENSMUSG00000050578	Mmp13	1.965901711	2.98E-47
ENSMUSG00000062077	Trim54	1.935152602	0.00670544
ENSMUSG00000097657	Gm7389	1.933064727	0.024552417
ENSMUSG00000115109	Gm49032	1.893191191	0.026369477
ENSMUSG00000085054	Gm15834	1.886393392	0.000592636
ENSMUSG00000032532	Cck	1.875736167	9.90E-25
ENSMUSG00000032878	Ccdc85a	1.868422733	8.50E-09
ENSMUSG00000029379	Cxcl3	1.854986555	4.29E-60
ENSMUSG00000056457	Prl2c3	1.848654264	2.51E-13
ENSMUSG00000099041	Gm28035	1.847555013	3.61E-07
ENSMUSG00000110344	Gm45716	1.809750964	6.60E-06
ENSMUSG00000096988	A930029G22Rik	1.75049229	0.016336367
ENSMUSG00000005373	Mlxipl	1.74457558	0.025478946
ENSMUSG00000094151	Gm7233	1.735396949	0.021689005
ENSMUSG00000047344	Lancl3	1.710484811	1.07E-11
ENSMUSG00000062345	Serpinb2	1.691032864	0.000224871
ENSMUSG00000079339	Ifit1bl1	1.676284274	1.39E-09
ENSMUSG00000050635	Sprr2f	1.645775704	0.038798478
ENSMUSG00000078868	Gm14412	1.624390344	0.019047287
ENSMUSG00000056836	Gm6851	1.621088282	0.007876852
ENSMUSG00000092072	Gm4540	1.620657478	0.016676341
ENSMUSG00000020676	Ccl11	1.564401676	0.000527564
ENSMUSG00000046203	Sprr2g	1.558837395	1.32E-05
ENSMUSG00000074183	Gsta1	1.55679302	3.12E-12
ENSMUSG00000019966	Kitl	1.542703025	4.41E-122
ENSMUSG00000042717	Ppp1r3a	1.517475834	0.013860978
ENSMUSG00000024912	Fosl1	1.482394065	2.56E-31
ENSMUSG00000095512	Gm17222	1.457874314	0.016398445
ENSMUSG00000063364	3300002I08Rik	1.425611905	0.033575747
ENSMUSG00000112963	Gm6093	1.409499532	0.009835607
ENSMUSG00000024331	Dsc2	1.403034038	0.015908694
ENSMUSG00000040715	Rsc1a1	1.390238884	0.006722121
ENSMUSG00000020826	Nos2	1.387815793	1.25E-45
ENSMUSG00000040828	Catsperd	1.387524116	0.023673495
ENSMUSG00000060924	Csmd1	1.385935482	0.005280117
ENSMUSG00000025503	Taldo1	1.380112703	2.75E-220
ENSMUSG00000027737	Slc7a11	1.379684326	5.68E-83
ENSMUSG00000031596	Slc7a2	1.378740993	1.78E-186
ENSMUSG00000050533	Gm9845	1.333567806	0.023743377
ENSMUSG00000109251	E230032D23Rik	1.333565933	0.011297673
ENSMUSG00000032265	Tent5a	1.323483761	1.55E-73
ENSMUSG00000027832	Ptx3	1.319566843	5.42E-29
ENSMUSG00000019880	Rspo3	1.311529033	1.77E-20
ENSMUSG00000113529	Gm47484	1.294741666	0.005995549
ENSMUSG00000031762	Mt2	1.274829425	1.07E-85
ENSMUSG00000035373	Ccl7	1.270077989	0
ENSMUSG00000039579	Grin3a	1.267773276	0.020095052
ENSMUSG00000104876	Trdc	1.259833467	2.52E-30
ENSMUSG00000002289	Angptl4	1.253673961	4.97E-99
ENSMUSG00000021700	Rab3c	1.236246488	0.014405594
ENSMUSG00000005413	Hmox1	1.228902251	2.29E-201
ENSMUSG00000025037	Maoa	1.220746938	8.30E-175
ENSMUSG00000019102	Aldh3a1	1.215774852	8.51E-21
ENSMUSG00000086111	Gm15326	1.215410857	0.024167935
ENSMUSG00000035041	Creb3l3	1.215119583	1.08E-07
ENSMUSG00000043263	Ifi209	1.207316776	0.002960118
ENSMUSG00000109536	9330162G02Rik	1.20525375	0.009318095
ENSMUSG00000031289	Il13ra2	1.203742741	2.42E-41

ENSMUSG00000027188	Pamr1	1.206076421	6.39E-60	ENSMUSG00000106961	Gm43128	1.198931326	0.003835282
ENSMUSG00000027820	Mme	1.204468521	1.09E-34	ENSMUSG00000032348	Gsta4	1.197685617	1.13E-17
ENSMUSG00000056643	Chst13	1.198750977	0.018806935	ENSMUSG00000050157	Gm867	1.192148473	0.005624447
ENSMUSG00000091405	H4c14	1.187691665	0.016902019	ENSMUSG00000055301	Adh7	1.19129819	0.000879937
ENSMUSG00000105096	Gbp10	1.185344753	3.35E-14	ENSMUSG00000029417	Cxcl9	1.188361206	0.001263597
ENSMUSG00000085977	Gm5970	1.178600842	3.59E-05	ENSMUSG00000063193	Cd300lb	1.186843498	0.000708441
ENSMUSG00000031289	Il13ra2	1.178119397	7.56E-46	ENSMUSG00000098158	Gm4804	1.178131903	5.96E-09
ENSMUSG00000110393	Gm36445	1.176743905	0.003390617	ENSMUSG00000049511	Htr1b	1.178054152	0.003865244
ENSMUSG00000028780	Sema3c	1.17671593	1.23E-121	ENSMUSG00000011008	Mcoln2	1.17504284	0.001280199
ENSMUSG00000028024	Enpep	1.176701331	3.01E-06	ENSMUSG00000042244	Pglyrp3	1.17469499	0.015648192
ENSMUSG00000044176	Spink10	1.168563434	0.031629475	ENSMUSG00000040466	Blvrb	1.157662141	2.45E-103
ENSMUSG00000028124	Gclm	1.168531185	1.75E-177	ENSMUSG00000045362	Tnfrsf26	1.156263489	9.53E-206
ENSMUSG00000002289	Angptl4	1.161107439	3.41E-104	ENSMUSG00000032802	Srxn1	1.15569304	1.55E-138
ENSMUSG00000086320	Gm12840	1.148308958	0.002137052	ENSMUSG00000030187	Klra2	1.151535189	0.01264438
ENSMUSG00000027737	Slc7a11	1.147346796	1.31E-50	ENSMUSG00000030162	Olr1	1.15105563	3.21E-91
ENSMUSG00000063286	Gm8995	1.146537043	2.85E-13	ENSMUSG00000097318	1700007L15Rik	1.149797117	0.043867745
ENSMUSG00000025993	Slc40a1	1.142530949	1.42E-15	ENSMUSG00000045932	Ifit2	1.149204596	0.022227503
ENSMUSG00000049115	Agtr1a	1.13647431	3.04E-26	ENSMUSG00000052180	Serpinb6c	1.144408864	1.27E-05
ENSMUSG00000030187	Klra2	1.134578042	0.015882626	ENSMUSG00000105852	Gm42890	1.138926643	0.040381707
ENSMUSG00000025037	Maoa	1.131163723	2.07E-167	ENSMUSG00000079101	Esd-ps	1.136427249	0.030413303
ENSMUSG00000019775	Rgs17	1.125254414	1.72E-36	ENSMUSG00000049928	Glp2r	1.133261445	4.42E-06
ENSMUSG00000096929	A330023F24Rik	1.124598899	2.08E-05	ENSMUSG00000044349	Snhg11	1.111368637	8.65E-56
ENSMUSG00000012428	Steap4	1.117479339	1.78E-118	ENSMUSG00000021478	Drd1	1.109659316	0.006769632
ENSMUSG00000045932	Ifit2	1.112269431	0.001754991	ENSMUSG00000034634	Ly6d	1.108014865	0.024658963
ENSMUSG00000021478	Drd1	1.103856923	0.004389799	ENSMUSG00000089698	Gm2541	1.099697982	0.029486692
ENSMUSG00000118361	Gm50237	1.092017448	0.031976241	ENSMUSG00000020334	Slc22a4	1.078284392	1.62E-06
ENSMUSG00000087611	4930458D05Rik	1.088076268	0.04808327	ENSMUSG00000014030	Pax5	1.078071016	1.38E-15
ENSMUSG00000025503	Taldo1	1.076415204	7.79E-117	ENSMUSG00000066677	Ifi208	1.075120566	6.93E-05
ENSMUSG00000032265	Tent5a	1.070532746	2.04E-53	ENSMUSG00000025194	Abcc2	1.073513947	0.046277678
ENSMUSG00000045441	Gprin3	1.069213628	0.005082255	ENSMUSG00000058427	Cxcl2	1.073483173	2.53E-28
ENSMUSG00000026166	Ccl20	1.068554748	7.31E-24	ENSMUSG00000112035	Gm49335	1.070936873	0.037463882
ENSMUSG00000102602	A930004J17Rik	1.067308802	0.000419247	ENSMUSG00000036144	Meox2	1.07053535	2.70E-27
ENSMUSG00000104876	Trdc	1.062977287	2.86E-21	ENSMUSG00000090942	F830016B08Rik	1.06890131	0.005117745
ENSMUSG00000055413	H2-Q5	1.061355927	1.99E-28	ENSMUSG00000058542	Gm15590	1.067929924	0.001141139
ENSMUSG00000021466	Ptch1	1.05687755	2.50E-35	ENSMUSG00000032487	Ptgs2	1.057103442	9.02E-94
ENSMUSG00000027408	Cpxm1	1.053247362	9.18E-126	ENSMUSG00000056643	Chst13	1.054932874	0.041707575
ENSMUSG00000004328	Hif3a	1.044524024	0.008439044	ENSMUSG00000002997	Prkar2b	1.051188954	1.13E-37
ENSMUSG00000097149	G630030J09Rik	1.042578037	0.047782078	ENSMUSG00000032350	Gclc	1.050200111	5.65E-95
ENSMUSG00000051022	Hs3st1	1.038429355	6.42E-14	ENSMUSG00000067219	Nipal1	1.040645235	0.000445454
ENSMUSG00000035373	Ccl7	1.034659702	5.16E-199	ENSMUSG00000022790	Igsf11	1.033822073	0.0040635
ENSMUSG00000033715	Akr1c14	1.033002967	1.95E-23	ENSMUSG00000029371	Cxcl5	1.031667504	1.20E-170
ENSMUSG00000016496	Cd274	1.032177329	7.80E-32	ENSMUSG00000094989	Rpl9-ps4	1.01488836	0.017484005
ENSMUSG00000085786	Gm15987	1.028407229	3.26E-07	ENSMUSG00000043613	Mmp3	1.012279049	4.74E-267
ENSMUSG00000086513	9130208D14Rik	1.025122656	0.000407738	ENSMUSG00000002565	Scin	1.007368277	0.008668938
ENSMUSG00000032348	Gsta4	1.025078329	1.11E-14	ENSMUSG00000003948	Mmd	1.004501952	8.66E-59
ENSMUSG00000038178	Slc43a2	1.018458103	5.89E-10	ENSMUSG00000063286	Gm8995	1.004355047	0.005106742
ENSMUSG00000031762	Mt2	1.018230652	8.31E-94	ENSMUSG00000049676	Catsperg1	0.999816282	0.000297953
ENSMUSG00000068587	Mgam	1.012580686	0.004136513	ENSMUSG00000023473	Celsr3	0.998720222	0.001851299
ENSMUSG00000074213	Gm10642	1.012116186	0.00050294	ENSMUSG00000092274	Neat1	0.997037751	7.11E-81
ENSMUSG00000050944	Efcab5	1.010552784	0.026203187	ENSMUSG00000090145	Ugt1a6b	0.994468577	1.25E-06
ENSMUSG00000022425	Enpp2	1.010061751	1.86E-37	ENSMUSG00000040907	Atp1a3	0.993861552	2.97E-08
ENSMUSG00000030257	Srgap3	1.005263923	1.04E-55	ENSMUSG00000049422	Chchd10	0.9921084	1.95E-09
ENSMUSG00000037747	Phyhipl	1.001876904	0.043842233	ENSMUSG00000028124	Gclm	0.989099315	1.33E-97
ENSMUSG00000039519	Cyp7b1	0.996603481	1.76E-05	ENSMUSG00000118106	AC115752.1	0.988392997	0.045979874
ENSMUSG00000030022	Adamts9	0.99504368	7.15E-40	ENSMUSG00000105194	Gm6755	0.977451483	0.004481561
ENSMUSG00000105843	Gm19439	0.994087965	0.028841713	ENSMUSG00000025993	Slc40a1	0.976097384	2.59E-10
ENSMUSG00000055480	Zfp458	0.98886278	0.000256138	ENSMUSG00000110588	Gm45774	0.974402445	0.000215509
ENSMUSG00000018986	Slfn3	0.988263773	0.013318057	ENSMUSG00000095545	Zfp969	0.972489424	0.042784796
ENSMUSG00000098158	Gm4804	0.987727641	2.66E-06	ENSMUSG00000075000	Nrbf2	0.97090955	1.18E-30
ENSMUSG00000092277	Gm19684	0.986221404	0.000189336	ENSMUSG00000041620	Mmp1b	0.970050105	0.000112553
ENSMUSG00000074934	Grem1	0.98184081	0.004502002	ENSMUSG00000004098	Col5a3	0.968421622	2.45E-48
ENSMUSG00000035105	Egln3	0.980817547	1.89E-13	ENSMUSG00000022408	Fam83f	0.966983007	0.000113641
ENSMUSG00000006818	Sod2	0.979912862	4.06E-141	ENSMUSG00000027611	Procr	0.966735745	9.89E-76
ENSMUSG00000024042	Sik1	0.973251767	1.10E-13	ENSMUSG00000037849	Ifi206	0.954864552	0.003862498
ENSMUSG00000047878	A4galt	0.96909466	1.09E-30	ENSMUSG00000066245	Gm10156	0.950296529	0.029889073
ENSMUSG00000029380	Cxcl1	0.968173834	1.23E-138	ENSMUSG00000096929	A330023F24Rik	0.945066303	0.000734514
ENSMUSG00000056856	Jakmip3	0.968043114	0.040177421	ENSMUSG00000109297	Gm31522	0.939988925	0.014683684
ENSMUSG00000024076	Vit	0.96513908	8.05E-12	ENSMUSG00000018916	Csf2	0.922512606	0.000443527
ENSMUSG00000095648	Gm2004	0.96251085	0.005196412	ENSMUSG00000086513	9130208D14Rik	0.91956669	5.65E-08
ENSMUSG00000017737	Mmp9	0.961148252	1.22E-05	ENSMUSG00000021466	Ptch1	0.919044796	1.60E-25
ENSMUSG00000033327	Tnxb	0.958411197	2.52E-50	ENSMUSG00000005686	Ampd3	0.91881739	1.55E-102
ENSMUSG00000018500	Adora2b	0.95547571	1.65E-07	ENSMUSG00000020641	Rsad2	0.916004417	0.000182221
ENSMUSG00000063558	Aox1	0.951822301	1.56E-36	ENSMUSG00000025746	Il6	0.9149118	1.06E-72
ENSMUSG00000029206	Nsun7	0.950106453	8.51E-05	ENSMUSG00000023088	Abcc1	0.906213635	3.36E-90
ENSMUSG00000032350	Gclc	0.946826302	1.51E-77	ENSMUSG00000000340	Dbt	0.904973013	2.46E-29
ENSMUSG00000034071	Zfp551	0.94580086	0.011854103	ENSMUSG00000021575	Ahrr	0.904869985	0.037160196
ENSMUSG00000037362	Ccn3	0.94433685	4.84E-21	ENSMUSG00000015843	Rxrg	0.899246838	0.042037976

ENSMUSG00000085875	Gm12905	0.943583938	0.001546177
ENSMUSG00000025780	Itih5	0.942147861	7.22E-23
ENSMUSG00000073491	Ifi213	0.939035159	0.002694166
ENSMUSG00000074183	Gsta1	0.938549014	0.000289069
ENSMUSG00000079363	Gbp4	0.933874204	0.002236613
ENSMUSG00000044337	Ackr3	0.931723644	9.43E-39
ENSMUSG00000047497	Adamts12	0.931617914	1.49E-45
ENSMUSG00000044749	Abca6	0.931542314	6.88E-07
ENSMUSG00000109771	Gm35315	0.931498876	0.034409599
ENSMUSG00000090093	Gm14399	0.930003543	0.000160516
ENSMUSG00000036330	Slc18a1	0.929765825	0.0113569
ENSMUSG00000050896	Rtn4rl2	0.929676128	9.86E-17
ENSMUSG00000037849	Ifi206	0.92948508	0.006111914
ENSMUSG00000039699	Batf2	0.927837163	5.84E-05
ENSMUSG00000040170	Fmo2	0.924649576	1.08E-19
ENSMUSG00000000386	Mx1	0.924330643	0.04836933
ENSMUSG00000116946	Gm41442	0.921868748	0.032839963
ENSMUSG00000044566	Cage1	0.916277509	0.040395777
ENSMUSG00000039982	Dtx4	0.91604044	1.53E-61
ENSMUSG00000004098	Col5a3	0.915443905	4.81E-60
ENSMUSG00000024066	Xdh	0.914335817	2.59E-16
ENSMUSG00000058427	Cxcl2	0.912317598	6.82E-21
ENSMUSG00000117964	Gm36043	0.907845488	0.004381818
ENSMUSG00000074519	Zfp971	0.90741181	4.00E-05
ENSMUSG00000039457	Ppl	0.901677364	1.63E-07
ENSMUSG00000032802	Srxn1	0.901519212	3.89E-97
ENSMUSG00000050860	Phospho1	0.897073987	1.09E-06
ENSMUSG00000041620	Mmp1b	0.893668093	0.0001257
ENSMUSG00000037428	Vgf	0.885593164	2.08E-06
ENSMUSG00000039963	Ccdc40	0.884701868	0.03685758
ENSMUSG00000038276	Asic3	0.882033214	1.44E-10
ENSMUSG00000021575	Ahrr	0.880307348	0.049703975
ENSMUSG00000034855	Cxcl10	0.878037193	0.042377806
ENSMUSG00000066677	Ifi208	0.878028235	0.001707987
ENSMUSG00000060961	Slc4a4	0.872718935	1.21E-14
ENSMUSG00000000732	Icosl	0.867373434	2.98E-10
ENSMUSG00000074207	Adh1	0.866995154	0.03204416
ENSMUSG00000058624	Gda	0.864033211	1.69E-24
ENSMUSG00000050395	Tnfsf15	0.863766735	0.000827991
ENSMUSG00000032532	Cck	0.863610369	0.000251474
ENSMUSG00000109408	A930037H05Rik	0.859981937	0.013343432
ENSMUSG00000005800	Mmp8	0.859505397	9.75E-17
ENSMUSG00000027339	Rassf2	0.858073071	0.005314511
ENSMUSG00000026532	Spta1	0.857518668	2.93E-46
ENSMUSG00000000730	Dnmt3l	0.856553591	1.37E-10
ENSMUSG00000041912	Tdrkh	0.854533029	0.016663514
ENSMUSG00000022797	Tfrc	0.854468125	1.24E-30
ENSMUSG00000040181	Fmo1	0.853758429	5.00E-08
ENSMUSG00000043017	Ptgir	0.851012872	7.81E-25
ENSMUSG00000025888	Casp1	0.847848527	0.000785439
ENSMUSG00000034706	Dnaic2	0.845462287	0.002719185
ENSMUSG00000029371	Cxcl5	0.844473625	5.98E-100
ENSMUSG00000027611	Procr	0.842544549	5.53E-56
ENSMUSG00000078496	Zfp982	0.842244616	0.000140357
ENSMUSG00000025019	Lcor	0.841328903	4.05E-08
ENSMUSG00000074521	Gm14327	0.84096187	0.000662608
ENSMUSG00000095193	Gm20939	0.840370253	5.42E-06
ENSMUSG00000110256	Gm45412	0.840308711	0.024014621
ENSMUSG00000024379	Tslp	0.839497031	0.003350931
ENSMUSG00000000340	Dbt	0.83870166	3.70E-26
ENSMUSG00000034997	Htr2a	0.838337311	3.34E-28
ENSMUSG00000019880	Rspo3	0.83550345	9.39E-08
ENSMUSG00000048644	Ctxn1	0.833466778	9.17E-09
ENSMUSG00000040466	Blvrb	0.832501626	3.24E-53
ENSMUSG00000044349	Snhg11	0.826249004	7.20E-32
ENSMUSG00000037406	Htra4	0.822849887	2.06E-09
ENSMUSG00000030110	Ret	0.822209272	6.54E-13
ENSMUSG00000018916	Csf2	0.821628609	0.003437432
ENSMUSG00000079575	Rbpj-ps3	0.820040769	1.21E-05
ENSMUSG00000042254	Cilp	0.819394931	1.04E-19
ENSMUSG00000045392	Olfr1033	0.818390493	0.010044252
ENSMUSG00000068606	Gm4841	0.817854273	2.87E-05
ENSMUSG00000029298	Gbp9	0.81751362	0.000215964
ENSMUSG00000032661	Oas3	0.814871269	0.001053167
ENSMUSG00000014030	Pax5	0.813187227	3.78E-08
ENSMUSG00000003949	Hlf	0.810978502	3.15E-05
ENSMUSG00000038296	Galnt18	0.807556539	0.000126544
ENSMUSG00000031765	Mt1	0.898757112	1.87E-65
ENSMUSG00000040584	Abcb1a	0.890640487	1.98E-10
ENSMUSG00000020407	Upp1	0.882151954	0.00392617
ENSMUSG00000021996	Esd	0.882004322	6.80E-145
ENSMUSG00000059588	Calcrl	0.874991461	1.11E-28
ENSMUSG00000085875	Gm12905	0.871434313	0.007108245
ENSMUSG00000094786	Gm14403	0.870616461	0.024483522
ENSMUSG00000073489	Ifi204	0.867542365	1.72E-06
ENSMUSG00000037406	Htra4	0.864787459	8.98E-10
ENSMUSG00000056494	Cngb3	0.858440768	0.00089162
ENSMUSG00000097027	Gm26559	0.854798401	0.018658494
ENSMUSG00000078894	2210418O10Rik	0.853699918	1.29E-05
ENSMUSG00000042379	Esm1	0.85310677	2.59E-14
ENSMUSG00000006014	Prg4	0.852165575	2.81E-41
ENSMUSG00000066755	Tnfsf18	0.84981832	3.08E-16
ENSMUSG00000033066	Gas7	0.848274399	5.52E-95
ENSMUSG00000028583	Pdpn	0.846926129	5.72E-139
ENSMUSG00000050395	Tnfsf15	0.84691381	0.003965368
ENSMUSG00000060550	H2-Q7	0.845638743	4.70E-35
ENSMUSG00000032420	Nt5e	0.840423166	1.68E-21
ENSMUSG00000115785	Gm6740	0.835477243	7.40E-05
ENSMUSG00000024066	Xdh	0.832622658	2.05E-09
ENSMUSG00000089726	Mir17hg	0.829095395	0.046352415
ENSMUSG00000085977	Gm5970	0.827615413	0.01286229
ENSMUSG00000063415	Cyp26b1	0.826034889	7.42E-08
ENSMUSG00000097000	Gm17435	0.824556515	5.34E-07
ENSMUSG00000020152	Actr2	0.823579181	1.49E-70
ENSMUSG00000027713	1810062G17Rik	0.823254383	0.013567341
ENSMUSG00000108402	9430064I24Rik	0.812910104	0.03987651
ENSMUSG00000078771	Evi2a	0.810935451	3.31E-12
ENSMUSG00000056758	Hmga2	0.807284443	6.89E-12
ENSMUSG00000028691	Prdx1	0.805837149	4.07E-95
ENSMUSG00000022864	D16Ertd472e	0.805375506	5.47E-11
ENSMUSG00000027820	Mme	0.800525993	8.07E-13
ENSMUSG00000000730	Dnmt3l	0.797809581	8.80E-09
ENSMUSG00000079575	Rbpj-ps3	0.796250089	3.41E-05
ENSMUSG00000031425	Plp1	0.795798723	0.000214489
ENSMUSG00000078866	Zfp970	0.790117812	3.82E-12
ENSMUSG00000039934	Gsap	0.789556162	5.21E-15
ENSMUSG00000090222	Ifi203-ps	0.78638237	0.000552737
ENSMUSG00000015568	Lpl	0.78529293	3.93E-77
ENSMUSG00000021213	Akr1c13	0.785162953	2.14E-11
ENSMUSG00000095648	Gm2004	0.785131668	0.040551317
ENSMUSG00000017929	B4galt5	0.784874356	1.45E-104
ENSMUSG00000039699	Batf2	0.784496538	0.000761416
ENSMUSG00000016496	Cd274	0.782722765	9.71E-17
ENSMUSG00000026525	Opn3	0.780617119	0.002115678
ENSMUSG00000030218	Mgp	0.776830609	2.68E-53
ENSMUSG00000015837	Sqstm1	0.775120655	8.36E-127
ENSMUSG00000082062	Ftl2-ps	0.773613171	0.001372284
ENSMUSG00000037722	Gnpnat1	0.769776358	2.39E-15
ENSMUSG00000095457	Gm8989	0.767194262	0.000709117
ENSMUSG00000006818	Sod2	0.765993751	6.08E-79
ENSMUSG00000095649	Gm8979	0.763468862	0.026937923
ENSMUSG00000033327	Tnxb	0.76332515	2.12E-20
ENSMUSG00000038178	Slc43a2	0.76327479	0.000133265
ENSMUSG00000086922	Gm13835	0.762895156	0.049437995
ENSMUSG00000029553	Tfec	0.762064269	2.03E-18
ENSMUSG00000019732	Calr3	0.761286983	0.007637831
ENSMUSG00000027792	Bche	0.759895002	0.000818456
ENSMUSG00000027188	Pamr1	0.754147236	3.25E-20
ENSMUSG00000064267	Hvcn1	0.754146511	2.21E-55
ENSMUSG00000050697	Prkaa1	0.75397667	6.52E-43
ENSMUSG00000047022	Mipol1	0.752180962	0.000403654
ENSMUSG00000092277	Gm19684	0.751243121	0.012601727
ENSMUSG00000054072	Iigp1	0.747839411	0.006066125
ENSMUSG00000074151	Nlrc5	0.7470251	0.007181786
ENSMUSG00000024810	Il33	0.743113024	0.027593402
ENSMUSG00000031519	Asb5	0.742189924	4.98E-53
ENSMUSG00000027834	Serpini1	0.742102697	6.25E-18
ENSMUSG00000028179	Cth	0.742095775	3.68E-06
ENSMUSG00000047878	A4galt	0.741695223	1.31E-15
ENSMUSG00000028974	Dffa	0.741512812	8.97E-12
ENSMUSG00000033213	AA467197	0.740481265	9.23E-11
ENSMUSG00000066632	Pgk1-rs7	0.740138538	0.037094131
ENSMUSG00000024737	Slc15a3	0.739935667	1.96E-25
ENSMUSG00000026701	Prdx6	0.738885263	3.53E-105

ENSMUSG00000079038	D130040H23Rik	0.804278493	0.012907333	ENSMUSG00000020601	Trib2	0.733037268	6.24E-55
ENSMUSG00000043613	Mmp3	0.802878562	2.17E-185	ENSMUSG00000025779	Ly96	0.732207164	1.45E-12
ENSMUSG00000045573	Penk	0.802000337	0.000106415	ENSMUSG00000078889	Gm14288	0.732114712	0.018552851
ENSMUSG00000016349	Eef1a2	0.801994681	1.03E-16	ENSMUSG00000034706	Dnaic2	0.730173696	0.01597466
ENSMUSG00000097027	Gm26559	0.801650527	0.01911366	ENSMUSG00000076036	Gm22133	0.729524314	0.000116111
ENSMUSG00000025746	Il6	0.801494372	4.12E-60	ENSMUSG00000050370	Ch25h	0.727926288	6.71E-54
ENSMUSG00000048376	F2r	0.800619741	9.71E-106	ENSMUSG00000027859	Ngf	0.726831168	3.70E-51
ENSMUSG00000032849	Abcc4	0.798701265	1.09E-33	ENSMUSG00000046223	Plaur	0.726827597	2.99E-37
ENSMUSG00000067235	H2-Q10	0.797636233	9.29E-05	ENSMUSG00000093553	Gm20633	0.720272285	0.018347675
ENSMUSG00000035042	Ccl5	0.795009984	3.87E-15	ENSMUSG00000037362	Ccn3	0.719364863	2.87E-09
ENSMUSG00000024661	Fth1	0.792254962	1.98E-145	ENSMUSG00000057522	Spop	0.719178213	4.40E-46
ENSMUSG00000079491	H2-T10	0.790916563	1.88E-14	ENSMUSG00000073010	Gm5127	0.717060013	0.008908628
ENSMUSG00000032735	Ablim3	0.788758393	2.94E-06	ENSMUSG00000048376	F2r	0.715853795	6.00E-79
ENSMUSG00000089756	Zfp966	0.788478032	0.042232791	ENSMUSG00000056220	Pla2g4a	0.715830205	6.43E-36
ENSMUSG00000073016	Uprt	0.786826619	5.78E-07	ENSMUSG00000022122	Ednrb	0.713886631	0.001265933
ENSMUSG00000107215	Gm43197	0.784467776	2.49E-05	ENSMUSG00000063558	Aox1	0.712928371	7.19E-19
ENSMUSG00000003282	Plag1	0.782097984	0.013691791	ENSMUSG00000035725	Prkx	0.712035444	9.19E-38
ENSMUSG00000036334	Igsf10	0.780736343	1.52E-13	ENSMUSG00000073490	Ifi207	0.709852187	1.59E-05
ENSMUSG00000004113	Cacna1b	0.779922925	0.013392295	ENSMUSG00000073491	Ifi213	0.70885545	0.04885027
ENSMUSG00000005686	Ampd3	0.779025041	1.27E-72	ENSMUSG00000073016	Uprt	0.704856394	1.96E-05
ENSMUSG00000105504	Gbp5	0.77896392	0.000388945	ENSMUSG00000079260	Tmppe	0.703498337	0.000291018
ENSMUSG00000008540	Mgst1	0.778494163	3.78E-78	ENSMUSG00000036478	Btg1	0.703006545	9.68E-27
ENSMUSG00000108402	9430064I24Rik	0.775084876	0.036034497	ENSMUSG00000031608	Galnt7	0.701382534	5.10E-15
ENSMUSG00000020152	Actr2	0.774867203	5.28E-59	ENSMUSG00000044424	Gm9493	0.701037756	0.019316235
ENSMUSG00000027208	Fgf7	0.77142865	6.25E-58	ENSMUSG00000028751	Pla2g2e	0.69757423	4.94E-09
ENSMUSG00000039431	Mtmr7	0.770410162	0.015589058	ENSMUSG00000037104	Socs5	0.697481816	1.34E-46
ENSMUSG00000000934	Top1mt	0.769114831	1.08E-10	ENSMUSG00000039062	Anpep	0.695322687	3.59E-50
ENSMUSG00000116812	Gm2792	0.767907081	3.66E-08	ENSMUSG00000005803	Sqor	0.692957277	6.15E-31
ENSMUSG00000098050	Gm5345	0.767840153	2.85E-10	ENSMUSG00000054203	Ifi205	0.69222252	0.000155592
ENSMUSG00000054072	Iigp1	0.767210147	0.00032007	ENSMUSG00000022797	Tfrc	0.689334661	6.68E-18
ENSMUSG00000026185	Igfbp5	0.767007391	5.50E-06	ENSMUSG00000041827	Oasl1	0.688420084	0.004499054
ENSMUSG00000092200	Tnxa	0.765739419	0.023167643	ENSMUSG00000078901	Gm14440	0.687869899	0.028032927
ENSMUSG00000087684	1200007C13Rik	0.765271798	0.007339829	ENSMUSG00000089940	Gm4117	0.687202801	0.004360928
ENSMUSG00000073489	Ifi204	0.763312475	1.15E-07	ENSMUSG00000018363	Smurf2	0.686929674	2.10E-61
ENSMUSG00000046593	Tmem215	0.762825878	0.007298382	ENSMUSG00000073514	Dok6	0.686217433	0.005773614
ENSMUSG00000052563	D930048N14Rik	0.759899901	0.031943272	ENSMUSG00000117234	Gm7818	0.68546464	0.037975993
ENSMUSG00000023088	Abcc1	0.75987755	3.07E-68	ENSMUSG00000116875	Morf4l1-ps1	0.684302605	0.027101281
ENSMUSG00000059588	Calcrl	0.757148353	5.44E-25	ENSMUSG00000044703	Phf11a	0.683957626	9.10E-06
ENSMUSG00000022788	Fgd4	0.75497933	4.99E-13	ENSMUSG00000003849	Nqo1	0.682771611	1.89E-21
ENSMUSG00000101013	A630072M18Rik	0.750169874	1.69E-05	ENSMUSG00000032561	Acpp	0.681820301	0.017343938
ENSMUSG00000003849	Nqo1	0.750132032	4.36E-26	ENSMUSG00000031209	Heph	0.679663768	1.55E-36
ENSMUSG00000041958	Pigs	0.74941238	1.88E-68	ENSMUSG00000046711	Hmga1	0.678708422	1.56E-43
ENSMUSG00000084289	Gm6977	0.748807256	0.009316369	ENSMUSG00000035762	Tmem161b	0.678457538	1.56E-07
ENSMUSG00000117234	Gm7818	0.748672371	0.028102575	ENSMUSG00000025591	Tma16	0.677734898	1.40E-13
ENSMUSG00000095649	Gm8979	0.747510957	0.030155596	ENSMUSG00000039031	Arhgap18	0.677654808	1.29E-09
ENSMUSG00000026822	Lcn2	0.744567	1.20E-116	ENSMUSG00000026094	Stk17b	0.676103344	4.58E-56
ENSMUSG00000037994	Slc9b2	0.744283822	0.00407121	ENSMUSG00000055413	H2-Q5	0.674266163	8.60E-11
ENSMUSG00000020641	Rsad2	0.744103049	0.000688466	ENSMUSG00000104913	Gm6560	0.674018517	0.005760159
ENSMUSG00000020334	Slc22a4	0.744061271	0.004163135	ENSMUSG00000073147	5031425E22Rik	0.672735265	3.96E-09
ENSMUSG00000015134	Aldh1a3	0.742385473	0.000161937	ENSMUSG00000029380	Cxcl1	0.67088547	2.88E-47
ENSMUSG00000028072	Ntrk1	0.739858164	0.000728678	ENSMUSG00000066362	Rps13-ps1	0.670356788	0.008073401
ENSMUSG00000048616	Nog	0.738215772	0.004691569	ENSMUSG00000046687	Gm5424	0.669924247	0.019230926
ENSMUSG00000004038	Gstm3	0.737279501	2.03E-09	ENSMUSG00000054555	Adam12	0.66781309	1.69E-25
ENSMUSG00000085829	Gm4285	0.736219672	0.005109379	ENSMUSG00000008540	Mgst1	0.666972081	1.49E-51
ENSMUSG00000014773	Dll1	0.732100582	0.047175749	ENSMUSG00000054280	Prr14l	0.666545117	3.94E-16
ENSMUSG00000094942	Gm3604	0.731722426	0.023947863	ENSMUSG00000061104	Sap18b	0.666288539	0.026896357
ENSMUSG00000073490	Ifi207	0.731557058	1.48E-06	ENSMUSG00000044026	Slc35g1	0.664933255	0.001063896
ENSMUSG00000035121	Neil2	0.729299903	0.002013026	ENSMUSG00000024360	Etf1	0.664908163	2.24E-72
ENSMUSG00000057054	Inca1	0.728123826	0.026683183	ENSMUSG00000020638	Cmpk2	0.663915791	0.005552613
ENSMUSG00000028517	Plpp3	0.725646518	4.02E-60	ENSMUSG00000025321	Itgb8	0.663157959	3.75E-10
ENSMUSG00000034755	Pcdh11x	0.722027532	0.005584315	ENSMUSG00000045868	Gvin1	0.663052782	0.000341391
ENSMUSG00000058135	Gstm1	0.721692237	1.18E-110	ENSMUSG00000096965	3300005D01Rik	0.662980271	5.12E-42
ENSMUSG00000097804	Gm16685	0.721249487	0.015533065	ENSMUSG00000006345	Ggt1	0.661784272	0.002110717
ENSMUSG00000075000	Nrbf2	0.720692357	5.67E-17	ENSMUSG00000115338	Pnp	0.661680912	7.23E-33
ENSMUSG00000020434	4921536K21Rik	0.720113071	0.033830468	ENSMUSG00000073409	H2-Q6	0.661381263	7.18E-07
ENSMUSG00000027995	Tlr2	0.719801878	1.05E-36	ENSMUSG00000048076	Arf1	0.660737326	8.79E-76
ENSMUSG00000038357	Camp	0.719673246	2.78E-05	ENSMUSG00000007564	Ppp2r1a	0.659568132	1.37E-75
ENSMUSG00000079339	Ifit1bl1	0.718781405	0.011907611	ENSMUSG00000020696	Rffl	0.65861917	4.16E-14
ENSMUSG00000025964	Adam23	0.717017084	8.65E-18	ENSMUSG00000078606	Gm4070	0.658048745	0.000735183
ENSMUSG00000037104	Socs5	0.713460138	7.12E-45	ENSMUSG00000031400	G6pdx	0.657449799	8.04E-68
ENSMUSG00000060240	Cend1	0.712200754	0.031153023	ENSMUSG00000040152	Thbs1	0.654718172	2.42E-41
ENSMUSG00000031936	Hephl1	0.711755332	0.000188744	ENSMUSG00000093765	Gm20658	0.654259041	0.029672393
ENSMUSG00000090222	Ifi203-ps	0.706674327	0.002084374	ENSMUSG00000032849	Abcc4	0.653081167	1.85E-21
ENSMUSG00000042616	Oscp1	0.706026132	0.000695127	ENSMUSG00000107215	Gm43197	0.652281464	0.000589246
ENSMUSG00000028420	Tmem38b	0.70550907	3.05E-24	ENSMUSG00000030257	Srgap3	0.651964583	3.23E-19
ENSMUSG00000028076	Cd1d1	0.704911047	1.40E-06	ENSMUSG00000078249	Hmga1b	0.650019338	1.21E-11
ENSMUSG00000072066	6720489N17Rik	0.704521476	0.017535267	ENSMUSG00000009185	Ccl8	0.647813073	6.35E-22

ENSMUSG00000085615	A330035P11Rik	0.704135263	0.006708483	ENSMUSG00000027610	Gss	0.64777879	2.70E-25
ENSMUSG00000024887	Asah2	0.702640346	8.03E-23	ENSMUSG00000029287	Tgfbr3	0.6470941	3.96E-19
ENSMUSG00000084817	Gm5526	0.702411474	0.004537801	ENSMUSG00000015536	Mocs2	0.644096518	1.11E-17
ENSMUSG00000026204	Ptprn	0.699231127	1.92E-37	ENSMUSG00000000934	Top1mt	0.638699336	1.79E-07
ENSMUSG00000025591	Tma16	0.69709848	4.71E-17	ENSMUSG00000003949	Hlf	0.638531313	0.002972066
ENSMUSG00000035929	H2-Q4	0.696855756	5.03E-48	ENSMUSG00000026536	Ifi211	0.637734444	9.54E-06
ENSMUSG00000047910	Pcdhb16	0.69600202	0.000414948	ENSMUSG00000047414	Flrt2	0.636517267	1.48E-12
ENSMUSG00000035258	Abi3bp	0.695642514	0.000169831	ENSMUSG00000027995	Tlr2	0.634720273	9.82E-23
ENSMUSG00000013584	Aldh1a2	0.693354343	3.85E-54	ENSMUSG00000002308	Cd320	0.633755605	5.44E-09
ENSMUSG00000038496	Slc19a3	0.693101238	0.00078243	ENSMUSG00000098934	Gm18853	0.633111208	0.00142878
ENSMUSG00000051029	Serpinb1b	0.692861211	0.005031944	ENSMUSG00000049734	Trex1	0.632898688	1.60E-05
ENSMUSG00000073980	Gm9574	0.687752604	0.036780639	ENSMUSG00000036390	Gadd45a	0.63256949	5.44E-24
ENSMUSG00000097397	Gm16861	0.68764262	0.033348378	ENSMUSG00000022126	Acod1	0.632334871	0.010773308
ENSMUSG00000026072	Il1r1	0.686442896	3.19E-37	ENSMUSG00000007594	Hapln4	0.631913015	9.66E-05
ENSMUSG00000053835	H2-T24	0.686058563	0.000800099	ENSMUSG00000061436	Hipk2	0.630175135	0.000258679
ENSMUSG00000022144	Gdnf	0.685827101	4.34E-10	ENSMUSG00000041355	Ssr2	0.63013215	4.28E-45
ENSMUSG00000038067	Csf3	0.685363882	0.032067154	ENSMUSG00000014905	Dnajb9	0.629897727	1.59E-26
ENSMUSG00000096696	Zfp960	0.685348908	0.00016411	ENSMUSG00000038357	Camp	0.628165569	0.000670029
ENSMUSG00000079343	C1s2	0.682686828	0.016540468	ENSMUSG00000027962	Vcam1	0.627822862	9.16E-44
ENSMUSG00000076036	Gm22133	0.682445277	0.001744761	ENSMUSG00000047996	Prrg1	0.627615627	2.86E-08
ENSMUSG00000016206	H2-M3	0.682247056	4.00E-11	ENSMUSG00000028961	Pgd	0.627431046	9.18E-62
ENSMUSG00000116560	Gm2808	0.681340378	0.013570183	ENSMUSG00000022425	Enpp2	0.624272086	4.48E-11
ENSMUSG00000063018	2010204K13Rik	0.681212464	0.028951641	ENSMUSG00000028838	Extl1	0.622666258	0.046976931
ENSMUSG00000111394	Gm49759	0.680905226	3.29E-15	ENSMUSG00000032661	Oas3	0.616396418	0.02793402
ENSMUSG00000020620	Abca8b	0.680266779	8.81E-16	ENSMUSG00000026864	Hspa5	0.614245743	7.32E-83
ENSMUSG00000091199	Gm2619	0.679057285	0.003613229	ENSMUSG00000023915	Tnfrsf21	0.612314504	0.001132605
ENSMUSG00000053846	Lipg	0.67563152	6.77E-14	ENSMUSG00000022800	Fyttd1	0.612231277	1.50E-17
ENSMUSG00000079677	Fdx2	0.670065984	2.38E-05	ENSMUSG00000021549	Rasa1	0.61165526	1.90E-41
ENSMUSG00000063354	Slc39a4	0.668845143	1.81E-19	ENSMUSG00000041235	Chd7	0.611606117	0.001048771
ENSMUSG00000038555	Reep2	0.668043989	0.002091985	ENSMUSG00000048347	Pcdhb18	0.611432176	0.02980593
ENSMUSG00000104713	Gbp6	0.668018886	1.27E-06	ENSMUSG00000045817	Zfp36l2	0.610478973	2.18E-21
ENSMUSG00000037999	Arap2	0.667879399	4.73E-12	ENSMUSG00000041958	Pigs	0.609669917	1.21E-44
ENSMUSG00000017390	Aldoc	0.667845558	0.003150794	ENSMUSG00000015652	Steap1	0.607919601	1.03E-11
ENSMUSG00000032625	Thsd7a	0.667118387	1.26E-11	ENSMUSG00000018068	Ints2	0.60644208	3.69E-08
ENSMUSG00000056116	H2-T22	0.667029452	5.69E-37	ENSMUSG00000021040	Slirp	0.605124841	1.75E-05
ENSMUSG00000020642	Rnf144a	0.666917568	2.40E-08	ENSMUSG00000079505	Gm11131	0.604420013	0.012526343
ENSMUSG00000092274	Neat1	0.666138383	1.81E-09	ENSMUSG00000041439	Mfsd6	0.60402522	3.28E-19
ENSMUSG00000064105	Cnnm2	0.666001304	6.08E-08	ENSMUSG00000037225	Fgf2	0.603811655	6.24E-39
ENSMUSG00000029553	Tfec	0.665886296	1.07E-11	ENSMUSG00000071335	Mfsd4b3-ps	0.601845896	0.041480038
ENSMUSG00000039005	Tlr4	0.665479308	8.59E-15	ENSMUSG00000024014	Pim1	0.599804406	2.69E-27
ENSMUSG00000028716	Pdzk1ip1	0.663724859	0.037187538	ENSMUSG00000069184	Zfp72	0.599686305	0.027563958
ENSMUSG00000031765	Mt1	0.662221344	5.14E-47	ENSMUSG00000034993	Vat1	0.599002118	2.98E-45
ENSMUSG00000027797	Dclk1	0.661745286	7.98E-22	ENSMUSG00000028766	Alpl	0.596199204	5.67E-08
ENSMUSG00000031340	Gabre	0.660464387	1.48E-14	ENSMUSG00000074519	Zfp971	0.595718213	0.033631731
ENSMUSG00000053626	Tll1	0.659285048	3.65E-07	ENSMUSG00000013584	Aldh1a2	0.593948988	3.05E-26
ENSMUSG00000024806	Mlana	0.658343905	0.000127173	ENSMUSG00000080115	Eef1akmt3	0.592564526	0.000504938
ENSMUSG00000079505	Gm11131	0.658082015	0.002465984	ENSMUSG00000027399	Il1a	0.592304723	0.018059358
ENSMUSG00000006014	Prg4	0.658047481	2.86E-25	ENSMUSG00000034612	Chst11	0.592250743	1.37E-35
ENSMUSG00000074220	Zfp382	0.657142663	0.044700783	ENSMUSG00000043085	Tmem82	0.592058428	0.006416885
ENSMUSG00000054555	Adam12	0.655849685	9.40E-35	ENSMUSG00000117628	Gm50012	0.591676148	0.006882351
ENSMUSG00000025790	Slco3a1	0.654179723	2.95E-34	ENSMUSG00000103653	Gstp-ps	0.589746269	1.33E-17
ENSMUSG00000026043	Col3a1	0.653912225	3.52E-64	ENSMUSG00000027412	Lpin3	0.589652674	1.63E-20
ENSMUSG00000025911	Adhfe1	0.653867356	1.43E-08	ENSMUSG00000104222	Gm7292	0.589343154	3.48E-05
ENSMUSG00000082292	Gm12250	0.652492741	6.01E-05	ENSMUSG00000040328	Olfr56	0.588192208	0.000411038
ENSMUSG00000039157	Fam102a	0.651818816	1.58E-09	ENSMUSG00000037999	Arap2	0.587767004	1.36E-08
ENSMUSG00000027254	Map1a	0.650037287	4.99E-42	ENSMUSG00000028864	Hgf	0.586991522	0.000626694
ENSMUSG00000078606	Gm4070	0.649857562	0.000463767	ENSMUSG00000060594	Layn	0.586482635	1.14E-28
ENSMUSG00000006611	Hfe	0.649224631	5.74E-17	ENSMUSG00000040747	Cd53	0.585874533	0.000957274
ENSMUSG00000058093	Zfp729b	0.647233586	6.72E-10	ENSMUSG00000035385	Ccl2	0.585219198	3.64E-56
ENSMUSG00000028033	Kcnq5	0.646760725	2.41E-13	ENSMUSG00000025357	Dgka	-0.58743397	8.70E-06
ENSMUSG00000038521	C1s1	0.645230331	2.33E-48	ENSMUSG00000111977	Gm47163	-0.58778771	0.044042407
ENSMUSG00000079260	Tmppe	0.644656694	0.00086802	ENSMUSG00000051951	Xkr4	-0.588074437	0.034659892
ENSMUSG00000045868	Gvin1	0.64463622	0.000220628	ENSMUSG00000025271	Pfkfb1	-0.588105415	0.029538487
ENSMUSG00000103309	BC037039	0.644298036	2.13E-06	ENSMUSG00000020836	Coro6	-0.588214937	7.65E-08
ENSMUSG00000036278	Macrod1	0.641497503	9.27E-13	ENSMUSG00000001018	Snapin	-0.588519899	1.13E-17
ENSMUSG00000041945	Mfsd9	0.641123323	0.000186827	ENSMUSG00000015714	Cers2	-0.588534866	1.27E-34
ENSMUSG00000022126	Acod1	0.640501888	0.000748033	ENSMUSG00000061086	Myl4	-0.588852542	0.000242192
ENSMUSG00000078566	Bnip3	0.640028111	1.54E-16	ENSMUSG00000024590	Lmnb1	-0.589369999	2.21E-06
ENSMUSG00000079362	Gm43302	0.639595036	0.002726863	ENSMUSG00000029860	Zyx	-0.590761276	1.75E-54
ENSMUSG00000046031	Calhm6	0.639296638	0.014342785	ENSMUSG00000031595	Pdgfrl	-0.591550735	4.22E-21
ENSMUSG00000072621	Slfn10-ps	0.638061417	0.000222121	ENSMUSG00000045216	Hs6st1	-0.591770728	5.10E-17
ENSMUSG00000034438	Gbp8	0.636808669	0.035106342	ENSMUSG00000022296	Baalc	-0.592057053	0.049804586
ENSMUSG00000078894	2210418O10Rik	0.636313033	0.001504317	ENSMUSG00000046417	Lrrc75a	-0.592483709	3.07E-21
ENSMUSG00000060568	Fam78b	0.631815949	1.54E-14	ENSMUSG00000102252	Snrpn	-0.592758602	3.17E-12
ENSMUSG00000022800	Fyttd1	0.631768909	6.22E-19	ENSMUSG00000022309	Angpt1	-0.592913992	0.005223604
ENSMUSG00000046982	Tshz1	0.630655862	6.03E-19	ENSMUSG00000042029	Ncapg2	-0.593753431	4.38E-06
ENSMUSG00000054280	Prr14l	0.630537927	2.94E-11	ENSMUSG00000055493	Epm2a	-0.594403469	0.003316985

ENSMUSG00000110148	5830408C22Rik	0.630380176	0.003499075
ENSMUSG00000075010	AW112010	0.629861973	0.023485702
ENSMUSG00000038594	Cep85l	0.628818086	0.001468312
ENSMUSG00000024737	Slc15a3	0.627666841	7.92E-31
ENSMUSG00000025645	Ccdc51	0.62634198	0.003942339
ENSMUSG00000046805	Mpeg1	0.625536849	8.32E-15
ENSMUSG00000020277	Pfkl	0.624575038	5.02E-35
ENSMUSG00000074136	4930513N10Rik	0.62436378	0.037296966
ENSMUSG00000036197	Gxylt1	0.624254405	3.72E-17
ENSMUSG00000024140	Epas1	0.624101859	8.05E-58
ENSMUSG00000035561	Aldh1b1	0.623433084	0.011193123
ENSMUSG00000070315	4930581F22Rik	0.623098141	0.005622744
ENSMUSG00000107369	Gstm2-ps1	0.62300568	0.013319594
ENSMUSG00000059423	Zfp933	0.62186648	2.79E-07
ENSMUSG00000024589	Nedd4l	0.621825128	2.86E-31
ENSMUSG00000028270	Gbp2	0.620415918	3.19E-07
ENSMUSG00000015536	Mocs2	0.619694518	7.89E-17
ENSMUSG00000095457	Gm8989	0.619273562	0.010018189
ENSMUSG00000045294	Insig1	0.618635694	3.13E-68
ENSMUSG00000025909	Sntg1	0.618013398	0.0012543
ENSMUSG00000038527	C1rl	0.617250356	7.32E-17
ENSMUSG00000087138	Gm15545	0.616424368	0.026371485
ENSMUSG00000000794	Kcnn3	0.615567272	8.80E-07
ENSMUSG00000044026	Slc35g1	0.610459756	0.003176486
ENSMUSG00000028128	F3	0.609207457	1.29E-23
ENSMUSG00000050240	Hic2	0.609164289	0.042635661
ENSMUSG00000067430	Zfp763	0.608830286	0.010256635
ENSMUSG00000096727	Psmb9	0.608058858	3.51E-09
ENSMUSG00000039529	Atp8b1	0.607560756	1.57E-17
ENSMUSG00000106874	Gm20186	0.607413438	0.000683495
ENSMUSG00000040280	Ndufa4l2	0.605842227	0.001151255
ENSMUSG00000045362	Tnfrsf26	0.60479745	9.17E-54
ENSMUSG00000016382	Pls3	0.604419753	2.61E-58
ENSMUSG00000036006	Ripor2	0.604259521	0.020039955
ENSMUSG00000021069	Pygl	0.603956752	4.25E-08
ENSMUSG00000063388	BC023105	0.603930867	0.000238187
ENSMUSG00000031480	Thsd1	0.603918673	0.001274459
ENSMUSG00000032024	Clmp	0.603820431	8.09E-29
ENSMUSG00000024085	Man2a1	0.603542619	3.99E-24
ENSMUSG00000030340	Scnn1a	0.602290129	0.02989621
ENSMUSG00000078862	Gm14326	0.600044621	7.99E-05
ENSMUSG00000030162	Olr1	0.599832442	6.70E-23
ENSMUSG00000068874	Selenbp1	0.599621835	2.04E-05
ENSMUSG00000026701	Prdx6	0.5984342	5.93E-76
ENSMUSG00000011256	Adam19	0.597996318	1.91E-49
ENSMUSG00000002489	Tiam1	0.597357435	3.48E-23
ENSMUSG00000041828	Abca8a	0.596985574	3.69E-13
ENSMUSG00000032420	Nt5e	0.595704428	5.46E-11
ENSMUSG00000040026	Saa3	0.595359441	4.69E-31
ENSMUSG00000036769	Wdr44	0.595320344	6.16E-14
ENSMUSG00000027399	Il1a	0.59478627	0.010243908
ENSMUSG00000069184	Zfp72	0.594527026	0.025840226
ENSMUSG00000031608	Galnt7	0.594341133	1.67E-11
ENSMUSG00000033487	Fndc3a	0.59321238	3.39E-22
ENSMUSG00000042256	Ptchd4	0.592986096	0.008037365
ENSMUSG00000078867	Gm14418	0.591658097	0.021956381
ENSMUSG00000040663	Clcf1	0.590900039	3.99E-12
ENSMUSG00000021996	Esd	0.589514086	1.08E-55
ENSMUSG00000035725	Prkx	0.589454018	3.56E-26
ENSMUSG00000054203	Ifi205	0.589182551	8.58E-05
ENSMUSG00000029231	Pdgfra	0.587978573	4.29E-34
ENSMUSG00000095362	Gm14325	0.587837981	2.03E-05
ENSMUSG00000004317	Clcn5	0.587717793	9.86E-17
ENSMUSG00000038217	Tlcd2	0.587596806	1.47E-14
ENSMUSG00000026675	Hsd17b7	0.587483448	6.65E-27
ENSMUSG00000015305	Sash1	0.586893854	3.00E-40
ENSMUSG00000073427	Gm4924	0.586655859	0.016984864
ENSMUSG00000035275	Raver2	-0.585318506	0.000859567
ENSMUSG00000031700	Gpt2	-0.585764706	3.11E-09
ENSMUSG00000026930	Gpsm1	-0.586453605	4.93E-25
ENSMUSG00000032085	Tagln	-0.586624847	4.51E-61
ENSMUSG00000031840	Rab3a	-0.586986288	1.23E-05
ENSMUSG00000056888	Glipr1	-0.587256867	4.97E-17
ENSMUSG00000020303	Stc2	-0.588178108	1.13E-23
ENSMUSG00000041559	Fmod	-0.590767307	6.72E-35
ENSMUSG00000027469	Tpx2	-0.590877961	7.33E-19
ENSMUSG00000021253	Tgfb3	-0.592074539	8.24E-37

ENSMUSG00000033589	Reep4	-0.595230576	1.31E-12
ENSMUSG00000042043	Tbca	-0.596717894	5.62E-14
ENSMUSG00000068744	Psrc1	-0.597419201	7.53E-20
ENSMUSG00000002835	Chaf1a	-0.598035352	4.04E-11
ENSMUSG00000031024	Denn2b	-0.598381287	2.80E-45
ENSMUSG00000037211	Spry1	-0.599208666	7.32E-40
ENSMUSG00000034024	Cct2	-0.59944768	1.30E-49
ENSMUSG00000091971	Hspa1a	-0.599501003	9.80E-11
ENSMUSG00000021950	Anxa8	-0.599992412	7.27E-19
ENSMUSG00000006651	Aplp1	-0.600087272	6.65E-18
ENSMUSG00000059022	Kcp	-0.600547265	0.000216108
ENSMUSG00000098098	Bvht	-0.600738419	0.002691289
ENSMUSG00000003038	Hmgn2	-0.600862182	8.59E-19
ENSMUSG00000030035	Wbp1	-0.601055549	5.27E-16
ENSMUSG00000059479	B3gnt8	-0.602928559	0.001192528
ENSMUSG00000047767	Atg16l2	-0.60354124	1.68E-08
ENSMUSG00000030528	Blm	-0.603938481	0.001699312
ENSMUSG00000074766	Ism1	-0.603998184	2.32E-09
ENSMUSG00000078994	Zfp429	-0.604059553	1.34E-05
ENSMUSG00000026494	Kif26b	-0.604855677	2.24E-21
ENSMUSG00000097467	Gm26737	-0.60496234	0.001011739
ENSMUSG00000029101	Rgs12	-0.604973187	3.87E-22
ENSMUSG00000020827	Mink1	-0.605019818	6.67E-17
ENSMUSG00000029822	Osbpl3	-0.605393124	1.50E-06
ENSMUSG00000022096	Hr	-0.605914097	1.59E-07
ENSMUSG00000008136	Fhl2	-0.606100595	1.75E-48
ENSMUSG00000037979	Ccdc92	-0.606296217	0.017023003
ENSMUSG00000028078	Dclk2	-0.607991245	0.013621516
ENSMUSG00000041688	Amot	-0.608455941	3.37E-06
ENSMUSG00000046623	Gjb4	-0.608469677	0.016717709
ENSMUSG00000070576	Mn1	-0.608925414	1.42E-18
ENSMUSG00000090084	Srpx	-0.609292953	0.005444292
ENSMUSG00000037820	Tgm2	-0.609818734	5.35E-36
ENSMUSG00000021286	Zfyve21	-0.60998976	5.91E-10
ENSMUSG00000024190	Dusp1	-0.610171082	1.29E-13
ENSMUSG00000034799	Unc13a	-0.61027429	0.022239005
ENSMUSG00000021597	Slf1	-0.610905918	0.000545466
ENSMUSG00000040605	Bace2	-0.611360628	1.95E-11
ENSMUSG00000037474	Dtl	-0.611945292	0.001799037
ENSMUSG00000038781	Stap2	-0.612219594	0.000939549
ENSMUSG00000033405	Nudt15	-0.61309009	0.025630245
ENSMUSG00000032011	Thy1	-0.613343413	4.94E-43
ENSMUSG00000034457	Eda2r	-0.614463197	3.04E-22
ENSMUSG00000045763	Basp1	-0.614929451	2.26E-42
ENSMUSG00000022661	Cd200	-0.614930681	1.62E-15
ENSMUSG00000020032	Nuak1	-0.615158762	7.14E-35
ENSMUSG00000020303	Stc2	-0.615431802	3.85E-27
ENSMUSG00000046186	Cd109	-0.616010831	3.58E-25
ENSMUSG00000021811	Dnajc9	-0.616282746	2.60E-13
ENSMUSG00000079164	Tlr5	-0.617082378	0.007883394
ENSMUSG00000105703	Gm43305	-0.617367309	0.019300866
ENSMUSG00000068923	Syt11	-0.617857102	1.37E-08
ENSMUSG00000049313	Sorl1	-0.618070339	0.001310821
ENSMUSG00000040488	Ltbp4	-0.619939771	3.38E-23
ENSMUSG00000052353	Cemip	-0.620088305	2.96E-61
ENSMUSG00000007207	Stx1a	-0.620309714	7.43E-09
ENSMUSG00000034329	Brip1	-0.620718905	0.004971703
ENSMUSG00000027460	Angpt4	-0.621301889	8.25E-16
ENSMUSG00000036412	Arsi	-0.621973904	0.001938635
ENSMUSG00000044881	Coa4	-0.622072778	0.01452812
ENSMUSG00000037440	Vnn1	-0.622541574	6.94E-25
ENSMUSG00000032243	Itga11	-0.62261615	6.55E-29
ENSMUSG00000044927	H1f10	-0.622824297	9.58E-07
ENSMUSG00000073002	Vamp5	-0.622977889	3.66E-20
ENSMUSG00000025265	Fgd1	-0.62320647	8.61E-12
ENSMUSG00000041482	Piezo2	-0.623757713	5.34E-17
ENSMUSG00000048458	Inka2	-0.623856374	4.47E-14
ENSMUSG00000039405	Prss23	-0.624813941	9.44E-46
ENSMUSG00000024521	Pmaip1	-0.62524222	1.48E-13
ENSMUSG00000046808	Atp10d	-0.625399274	0.009311519
ENSMUSG00000031748	Gnao1	-0.625407673	0.000150695
ENSMUSG00000020758	Itgb4	-0.625433126	0.017910838
ENSMUSG00000040204	Pclaf	-0.625681704	1.56E-06
ENSMUSG00000056888	Glipr1	-0.626497361	1.08E-14
ENSMUSG00000020646	Mboat2	-0.626624058	0.000829208
ENSMUSG00000036098	Myrf	-0.627209006	9.26E-33
ENSMUSG00000026548	Slamf9	-0.627795152	1.42E-05

ENSMUSG00000023013	Aqp2	-0.592108363	0.033115848
ENSMUSG00000021464	Ror2	-0.59219358	0.00010188
ENSMUSG00000038147	Cd84	-0.592468003	0.024362699
ENSMUSG00000037960	Card19	-0.592918116	3.12E-26
ENSMUSG00000028591	Pramef12	-0.593090245	0.002760962
ENSMUSG00000009614	Sardh	-0.593214249	2.06E-25
ENSMUSG00000037493	Cib2	-0.593779634	0.004102993
ENSMUSG00000029228	Lnx1	-0.596322656	0.042493129
ENSMUSG00000072082	Ccnf	-0.60061751	5.26E-06
ENSMUSG00000025762	Larp1b	-0.601385913	4.80E-14
ENSMUSG00000023927	Satb1	-0.6028332	0.044283515
ENSMUSG00000040350	Trim7	-0.603057089	2.86E-08
ENSMUSG00000035413	Tmem98	-0.607407191	5.06E-46
ENSMUSG00000068882	Ssb	-0.607760838	1.07E-49
ENSMUSG00000051811	Cox6b2	-0.60845367	0.001090503
ENSMUSG00000047881	Rell1	-0.608621416	3.13E-11
ENSMUSG00000026315	Serpinb8	-0.610517578	1.19E-06
ENSMUSG00000007207	Stx1a	-0.612200751	7.83E-09
ENSMUSG00000062075	Lmnb2	-0.612245256	2.85E-06
ENSMUSG00000037664	Cdkn1c	-0.612421717	0.035574122
ENSMUSG00000024640	Psat1	-0.612638866	2.48E-14
ENSMUSG00000031119	Gpc4	-0.614577453	1.25E-53
ENSMUSG00000024190	Dusp1	-0.615518312	1.64E-25
ENSMUSG00000097789	Gm2115	-0.616448951	0.005933113
ENSMUSG00000021411	Pxdc1	-0.616550895	4.71E-24
ENSMUSG00000000686	Abhd15	-0.61718724	0.000166901
ENSMUSG00000029687	Ezh2	-0.618589984	1.88E-07
ENSMUSG00000026429	Ube2t	-0.618820869	0.009990057
ENSMUSG00000070461	9230112E08Rik	-0.621199091	1.81E-07
ENSMUSG00000031661	Nkd1	-0.621284766	0.003306275
ENSMUSG00000030793	Pycard	-0.622003572	0.000308218
ENSMUSG00000020256	Aldh1l2	-0.622760074	6.03E-21
ENSMUSG00000069792	Wfdc17	-0.623162622	0.015454966
ENSMUSG00000039137	Whrn	-0.623282376	2.61E-09
ENSMUSG00000022528	Hes1	-0.623395874	1.32E-12
ENSMUSG00000060600	Eno3	-0.623448857	6.92E-05
ENSMUSG00000030074	Gxylt2	-0.624655274	1.53E-33
ENSMUSG00000026039	Sgo2a	-0.62476458	0.000345505
ENSMUSG00000051378	Kif18b	-0.626457861	2.31E-10
ENSMUSG00000051331	Cacna1c	-0.626788835	4.36E-12
ENSMUSG00000074918	Inafm2	-0.627562323	1.39E-15
ENSMUSG00000048327	Ckap2l	-0.627805969	3.04E-08
ENSMUSG00000026228	Htr2b	-0.631169294	0.000426997
ENSMUSG00000071359	Tbpl1	-0.631446623	7.76E-14
ENSMUSG00000016756	Cmah	-0.631785501	0.032727634
ENSMUSG00000035783	Acta2	-0.63321354	1.19E-81
ENSMUSG00000022199	Slc22a17	-0.636593688	4.16E-08
ENSMUSG00000028194	Ddah1	-0.642226955	3.45E-61
ENSMUSG00000048387	Osr1	-0.643894861	2.82E-15
ENSMUSG00000005355	Casp14	-0.647862344	0.048624548
ENSMUSG00000002020	Ltbp2	-0.648237817	7.40E-47
ENSMUSG00000094483	Purb	-0.649810651	3.36E-29
ENSMUSG00000023919	Cenpq	-0.652129569	0.000753459
ENSMUSG00000055980	Irs1	-0.652200763	9.86E-17
ENSMUSG00000026278	Bok	-0.652420396	3.13E-14
ENSMUSG00000056091	St3gal5	-0.652421682	1.44E-41
ENSMUSG00000037887	Dusp8	-0.653955013	3.45E-08
ENSMUSG00000030677	Kif22	-0.654359595	5.29E-10
ENSMUSG00000002104	Rapsn	-0.654978499	0.001523685
ENSMUSG00000028581	Laptm5	-0.65574184	3.98E-08
ENSMUSG00000024486	Hbegf	-0.65656705	2.04E-31
ENSMUSG00000029608	Rph3a	-0.657066508	0.015449302
ENSMUSG00000028445	Enho	-0.658066434	0.000120423
ENSMUSG00000033207	Mamdc2	-0.659126003	1.05E-25
ENSMUSG00000056234	Ncoa4	-0.659325188	2.36E-35
ENSMUSG00000020186	Csrp2	-0.659548756	6.88E-39
ENSMUSG00000019851	Perp	-0.660742973	4.77E-10
ENSMUSG00000027715	Ccna2	-0.661423347	5.49E-12
ENSMUSG00000054793	Cadm4	-0.661578658	0.03563846
ENSMUSG00000035403	Crb2	-0.661888563	0.005370704
ENSMUSG00000017716	Birc5	-0.66370904	1.11E-07
ENSMUSG00000027508	Pag1	-0.663709085	2.16E-05
ENSMUSG00000024030	Abcg1	-0.664692136	2.63E-28
ENSMUSG00000038642	Ctss	-0.667685445	5.51E-15
ENSMUSG00000028884	Rpa2	-0.667810104	9.72E-07
ENSMUSG00000031776	Arl2bp	-0.669028984	1.32E-47
ENSMUSG00000027313	Chac1	-0.670331908	2.86E-15
ENSMUSG00000073910	Mob3b	-0.62796881	0.038813871
ENSMUSG00000051811	Cox6b2	-0.628812504	0.001312378
ENSMUSG00000073434	Wdr90	-0.628928968	0.012741658
ENSMUSG00000037621	Atoh8	-0.630999715	2.62E-25
ENSMUSG00000030978	Rrm1	-0.631139313	5.48E-42
ENSMUSG00000031995	St14	-0.631271637	1.36E-06
ENSMUSG00000026669	Mcm10	-0.631783966	0.000105483
ENSMUSG00000036885	Arhgef26	-0.631852049	1.95E-08
ENSMUSG00000069910	Spdl1	-0.632085879	0.000234706
ENSMUSG00000083307	AA414768	-0.632308634	9.74E-09
ENSMUSG00000028933	Xrcc2	-0.63274387	0.02535023
ENSMUSG00000022199	Slc22a17	-0.633309366	3.69E-07
ENSMUSG00000032122	Slc37a2	-0.635946915	0.000100509
ENSMUSG00000064043	Trerf1	-0.636754316	8.60E-09
ENSMUSG00000036036	Zfp57	-0.637181098	4.56E-10
ENSMUSG00000022177	Haus4	-0.637214455	0.000138587
ENSMUSG00000030122	Ptms	-0.637329882	1.17E-37
ENSMUSG00000029521	Chek2	-0.637687624	5.83E-11
ENSMUSG00000031158	Timm17b	-0.638496072	1.82E-11
ENSMUSG00000028807	Zbtb8a	-0.640157797	0.000741828
ENSMUSG00000020782	Llgl2	-0.642437028	2.33E-09
ENSMUSG00000009281	Rarres2	-0.642939191	1.69E-05
ENSMUSG00000040852	Plekhh2	-0.643232052	1.17E-19
ENSMUSG00000023885	Thbs2	-0.643367772	6.44E-67
ENSMUSG00000022833	Ccdc14	-0.644845577	0.015207854
ENSMUSG00000023990	Tfeb	-0.644960897	1.72E-05
ENSMUSG00000035314	Gdpd5	-0.646419962	1.08E-05
ENSMUSG00000042489	Clspn	-0.646438089	0.000198809
ENSMUSG00000020086	Macroh2a2	-0.646738891	3.63E-07
ENSMUSG00000031478	Nek3	-0.647865063	0.010716332
ENSMUSG00000024548	Setbp1	-0.648558376	7.70E-12
ENSMUSG00000037353	Letmd1	-0.649273397	3.49E-20
ENSMUSG00000019773	Fbxo5	-0.649441102	0.003354473
ENSMUSG00000042099	Kank3	-0.649740933	0.000133988
ENSMUSG00000026235	Epha4	-0.649953005	0.011241196
ENSMUSG00000034205	Loxl2	-0.65011042	2.73E-56
ENSMUSG00000006205	Htra1	-0.652031116	1.05E-26
ENSMUSG00000036902	Neto2	-0.652304477	0.005154672
ENSMUSG00000046561	Arsj	-0.653032236	0.00583599
ENSMUSG00000049556	Lingo1	-0.653369004	0.001818327
ENSMUSG00000029669	Tspan12	-0.653773037	2.64E-13
ENSMUSG00000018387	Shroom1	-0.654284666	1.67E-06
ENSMUSG00000028944	Prkag2	-0.655094403	3.87E-15
ENSMUSG00000038264	Sema7a	-0.65533274	0.00064948
ENSMUSG00000002020	Ltbp2	-0.655458196	9.94E-51
ENSMUSG00000026930	Gpsm1	-0.656320395	1.09E-27
ENSMUSG00000029687	Ezh2	-0.658556147	1.01E-07
ENSMUSG00000038644	Pold1	-0.658883408	1.26E-05
ENSMUSG00000010476	Ebf3	-0.659046051	5.33E-17
ENSMUSG00000021464	Ror2	-0.660058415	4.29E-06
ENSMUSG00000090115	Usp49	-0.66028601	7.87E-09
ENSMUSG00000040253	Gbp7	-0.661142689	0.000338616
ENSMUSG00000028066	Pmf1	-0.661155609	5.31E-08
ENSMUSG00000039713	Plekhg5	-0.661844743	3.60E-19
ENSMUSG00000026413	Pkp1	-0.662439137	0.018541301
ENSMUSG00000018217	Pmp22	-0.66249179	9.92E-94
ENSMUSG00000032009	Sesn3	-0.663809386	8.49E-12
ENSMUSG00000051177	Plcb1	-0.664268233	0.000447833
ENSMUSG00000025507	Pidd1	-0.664764929	1.49E-12
ENSMUSG00000034285	Nipsnap1	-0.665911967	0.00360401
ENSMUSG00000038332	Sesn1	-0.666447732	9.52E-08
ENSMUSG00000035246	Pcyt1b	-0.667460551	2.49E-08
ENSMUSG00000104063	Pcdhgb7	-0.667688191	0.001884279
ENSMUSG00000022048	Dpysl2	-0.667690813	7.52E-21
ENSMUSG00000038600	Atp6v0a4	-0.668165974	0.02270428
ENSMUSG00000014303	Glis2	-0.668225533	9.85E-32
ENSMUSG00000032815	Fanca	-0.66836408	0.000111858
ENSMUSG00000050107	Haspin	-0.668740691	0.007681746
ENSMUSG00000024660	Incenp	-0.668895063	4.80E-16
ENSMUSG00000011171	Vipr2	-0.668999721	0.019187407
ENSMUSG00000004267	Eno2	-0.670055538	1.94E-07
ENSMUSG00000069808	Fam57a	-0.671422052	2.21E-14
ENSMUSG00000034317	Trim59	-0.671613327	1.02E-17
ENSMUSG00000007379	Dennd2c	-0.672049476	0.001226375
ENSMUSG00000090877	Hspa1b	-0.67304623	7.93E-16
ENSMUSG00000017897	Eya2	-0.67328749	4.14E-07
ENSMUSG00000046329	Slc25a23	-0.673309432	4.19E-09

ENSMUSG00000018387	Shroom1	-0.670527643	7.60E-07
ENSMUSG00000042834	Nrep	-0.670900626	2.37E-10
ENSMUSG00000051225	Fam83a	-0.671364898	0.001113429
ENSMUSG00000002058	Unc119	-0.671832934	5.97E-26
ENSMUSG00000101462	Gm3052	-0.674126209	0.023450646
ENSMUSG00000030109	Slc6a12	-0.676031427	0.013947778
ENSMUSG00000060716	Plekhh1	-0.677618597	0.035849965
ENSMUSG00000032281	Acsbg1	-0.677841777	0.000105244
ENSMUSG00000020866	Cacna1g	-0.679408218	0.001223636
ENSMUSG00000037060	Cavin3	-0.6799443	9.81E-41
ENSMUSG00000042357	Gjb5	-0.682584762	6.68E-09
ENSMUSG00000051339	2900026A02Rik	-0.683055128	1.56E-19
ENSMUSG00000031292	Cdkl5	-0.684361142	0.000123693
ENSMUSG00000024065	Ehd3	-0.685982353	4.59E-14
ENSMUSG00000024989	Cep55	-0.68618259	4.69E-08
ENSMUSG00000020583	Matn3	-0.688966414	0.002647139
ENSMUSG00000047496	Rnf152	-0.689026739	2.00E-05
ENSMUSG00000025551	Fgf14	-0.691110104	0.004713105
ENSMUSG00000024590	Lmnb1	-0.691785528	3.40E-10
ENSMUSG00000006205	Htra1	-0.692838628	3.43E-43
ENSMUSG00000042190	Cmklr1	-0.693781854	6.49E-17
ENSMUSG00000029093	Sorcs2	-0.694055339	6.94E-05
ENSMUSG00000090125	Pou3f1	-0.695056132	0.013046468
ENSMUSG00000026605	Cenpf	-0.696365464	1.08E-07
ENSMUSG00000054136	Adm2	-0.699123142	0.004147741
ENSMUSG00000021485	Mxd3	-0.699386949	0.035936618
ENSMUSG00000028873	Cdca8	-0.701838108	9.86E-09
ENSMUSG00000071537	Klrg2	-0.704492277	0.00018499
ENSMUSG00000045777	Ifitm10	-0.707027348	4.59E-06
ENSMUSG00000031972	Acta1	-0.707443978	0.009533952
ENSMUSG00000017718	Afmid	-0.708568773	0.017660625
ENSMUSG00000025150	Cbr2	-0.70857162	0.004347992
ENSMUSG00000049001	Ndnf	-0.708988883	2.57E-05
ENSMUSG00000020846	Rflnb	-0.709340216	4.78E-43
ENSMUSG00000073910	Mob3b	-0.709776731	0.01045864
ENSMUSG00000079022	Col22a1	-0.709821813	0.009468289
ENSMUSG00000032783	Troap	-0.711141214	1.75E-05
ENSMUSG00000048368	Omd	-0.711353407	0.021840439
ENSMUSG00000098090	2700099C18Rik	-0.711769565	0.01448118
ENSMUSG00000027496	Aurka	-0.711982451	9.20E-14
ENSMUSG00000016995	Matn4	-0.712019022	0.006736324
ENSMUSG00000025701	Alox5	-0.712272218	0.007774602
ENSMUSG00000003585	Sec14l2	-0.716845922	0.001301914
ENSMUSG00000044456	Rin3	-0.717255088	6.26E-09
ENSMUSG00000027199	Gatm	-0.719505821	0.003402932
ENSMUSG00000046668	Cxxc5	-0.721754409	2.83E-20
ENSMUSG00000026413	Pkp1	-0.724302006	0.011549655
ENSMUSG00000030091	Nup210	-0.724967058	0.041291657
ENSMUSG00000089812	Gm15867	-0.725691567	0.041503151
ENSMUSG00000020897	Aurkb	-0.72584606	9.95E-09
ENSMUSG00000085667	Gm12992	-0.726972941	0.03163994
ENSMUSG00000020182	Ddc	-0.727288697	0.025453961
ENSMUSG00000047281	Sfn	-0.72776663	1.22E-11
ENSMUSG00000001493	Meox1	-0.728429582	2.43E-31
ENSMUSG00000084128	Esrp2	-0.729662709	1.87E-07
ENSMUSG00000022803	Popdc2	-0.73161972	4.41E-06
ENSMUSG00000026480	Ncf2	-0.731713467	0.000430879
ENSMUSG00000027223	Mapk8ip1	-0.734055269	1.92E-13
ENSMUSG00000019942	Cdk1	-0.734279605	3.68E-23
ENSMUSG00000021747	4930452B06Rik	-0.734418642	0.016270447
ENSMUSG00000021453	Gadd45g	-0.736156557	6.60E-74
ENSMUSG00000047638	Nr1h4	-0.73814461	8.98E-06
ENSMUSG00000044469	Tnfaip8l1	-0.745261661	0.026516064
ENSMUSG00000043008	Klhl6	-0.746335834	7.26E-22
ENSMUSG00000024899	Papss2	-0.747321118	1.13E-65
ENSMUSG00000004730	Adgre1	-0.747648171	1.64E-06
ENSMUSG00000032172	Olfm2	-0.747845635	5.70E-14
ENSMUSG00000035916	Ptprq	-0.748734752	0.000383188
ENSMUSG00000033952	Aspm	-0.748836182	2.94E-07
ENSMUSG00000037166	Ppp1r14a	-0.750175024	7.35E-13
ENSMUSG00000027331	Knstrn	-0.751078919	1.58E-09
ENSMUSG00000037628	Cdkn3	-0.755095915	0.000215706
ENSMUSG00000079018	Ly6c1	-0.755295542	1.22E-08
ENSMUSG00000023015	Racgap1	-0.755478217	5.22E-23
ENSMUSG00000027831	Veph1	-0.756776383	0.014672234
ENSMUSG00000027574	Nkain4	-0.757092284	0.013303989
ENSMUSG00000003283	Hck	-0.757425733	2.30E-08

ENSMUSG00000019122	Ccl9	-0.673514491	2.12E-13
ENSMUSG00000003378	Grik5	-0.673532485	2.83E-14
ENSMUSG00000062380	Tubb3	-0.674132249	5.43E-19
ENSMUSG00000026708	Cenpl	-0.674869233	0.008210579
ENSMUSG00000025795	Rassf3	-0.675042764	3.00E-14
ENSMUSG00000005148	Klf5	-0.675162216	0.000369975
ENSMUSG00000028698	Pik3r3	-0.675419728	3.74E-14
ENSMUSG00000035829	Ppp1r26	-0.675894886	0.043744374
ENSMUSG00000020186	Csrp2	-0.676701752	3.18E-45
ENSMUSG00000035305	Ror1	-0.677428231	6.73E-06
ENSMUSG00000021367	Edn1	-0.677524126	6.92E-23
ENSMUSG00000086825	Gm15675	-0.67753061	8.60E-06
ENSMUSG00000001228	Uhrf1	-0.678406989	3.35E-07
ENSMUSG00000024811	Tnks2	-0.680237621	9.56E-36
ENSMUSG00000006678	Pola1	-0.680255324	1.51E-05
ENSMUSG00000024026	Glo1	-0.680457856	7.85E-27
ENSMUSG00000105813	Gm42928	-0.680887756	0.009561351
ENSMUSG00000091475	Cerox1	-0.681374202	0.009980012
ENSMUSG00000060301	2610008E11Rik	-0.681902207	1.14E-14
ENSMUSG00000017146	Brca1	-0.682358856	2.28E-05
ENSMUSG00000036086	Zranb3	-0.682446199	0.001129727
ENSMUSG00000029675	Eln	-0.682684111	4.48E-38
ENSMUSG00000022297	Fzd6	-0.683293417	5.82E-26
ENSMUSG00000000126	Wnt9a	-0.683306257	7.08E-14
ENSMUSG00000032667	Pon2	-0.68354072	1.44E-43
ENSMUSG00000020238	Ncln	-0.685402057	2.06E-40
ENSMUSG00000039231	Suv39h1	-0.685761496	1.76E-09
ENSMUSG00000037509	Arhgef4	-0.686482251	0.004415263
ENSMUSG00000028399	Ptprd	-0.687300097	1.09E-17
ENSMUSG00000026414	Tnnt2	-0.687392016	0.000200561
ENSMUSG00000037960	Card19	-0.687512378	2.42E-19
ENSMUSG00000036596	Cpz	-0.688115019	0.047998342
ENSMUSG00000045659	Plekha7	-0.688183412	2.50E-25
ENSMUSG00000028194	Ddah1	-0.689091605	1.01E-76
ENSMUSG00000000253	Gmpr	-0.689581453	0.003119408
ENSMUSG00000014846	Tppp3	-0.690045817	0.013135481
ENSMUSG00000033031	Cip2a	-0.690783457	5.30E-11
ENSMUSG00000009376	Met	-0.690986719	1.67E-05
ENSMUSG00000036768	Kif15	-0.693041608	0.002606083
ENSMUSG00000047757	Fancb	-0.693963351	0.004420243
ENSMUSG00000036867	Smad6	-0.694690577	9.13E-30
ENSMUSG00000000093	Tbx2	-0.69487953	0.000242192
ENSMUSG00000045827	Serpinb9	-0.695243398	2.24E-37
ENSMUSG00000040447	Spns2	-0.697819193	0.000164731
ENSMUSG00000021390	Ogn	-0.698892038	3.27E-64
ENSMUSG00000020846	Rflnb	-0.699610204	1.24E-51
ENSMUSG00000046432	Bex3	-0.700185937	1.08E-25
ENSMUSG00000031451	Gas6	-0.700754186	7.76E-76
ENSMUSG00000023349	Clec4n	-0.701024091	0.0498193
ENSMUSG00000004655	Aqp1	-0.701412716	2.84E-06
ENSMUSG00000020331	Hcn2	-0.701831546	4.45E-06
ENSMUSG00000040612	Ildr2	-0.702015783	3.98E-08
ENSMUSG00000048960	Prex2	-0.702083927	2.37E-21
ENSMUSG00000028701	Lurap1	-0.702633133	0.005332415
ENSMUSG00000031951	Tmem231	-0.702753847	4.03E-06
ENSMUSG00000037572	Wdhd1	-0.703404633	0.000234919
ENSMUSG00000021047	Nova1	-0.703612428	0.002314594
ENSMUSG00000030074	Gxylt2	-0.704084408	3.79E-39
ENSMUSG00000021594	Srd5a1	-0.704173461	0.00068774
ENSMUSG00000028832	Stmn1	-0.705545098	1.25E-21
ENSMUSG00000036904	Fzd8	-0.705726448	1.66E-27
ENSMUSG00000049115	Agtr1a	-0.705907515	4.04E-06
ENSMUSG00000037466	Tedc1	-0.706376897	3.49E-06
ENSMUSG00000031849	Comp	-0.706848518	2.74E-06
ENSMUSG00000039813	Tbc1d2	-0.706890751	1.39E-06
ENSMUSG00000019214	Chtf18	-0.707554865	0.000661747
ENSMUSG00000012609	Ttll5	-0.708172611	4.11E-19
ENSMUSG00000038319	Kcnh2	-0.708501905	0.013328925
ENSMUSG00000022367	Has2	-0.709601396	0.004054084
ENSMUSG00000027223	Mapk8ip1	-0.709944359	1.18E-11
ENSMUSG00000054850	Smim10l2a	-0.710131462	0.002442408
ENSMUSG00000026429	Ube2t	-0.710204176	0.001087389
ENSMUSG00000027338	Prnd	-0.710220523	3.39E-15
ENSMUSG00000026039	Sgo2a	-0.710675253	5.13E-05
ENSMUSG00000039116	Adgrg6	-0.711241256	5.01E-11
ENSMUSG00000000489	Pdgfb	-0.711601399	6.81E-11
ENSMUSG00000050315	Synpo2	-0.711772841	3.06E-41

ENSMUSG00000026548	Slamf9	-0.758065618	1.24E-07
ENSMUSG00000050345	4930486L24Rik	-0.759594944	0.012665367
ENSMUSG00000070867	Trabd2b	-0.759723446	5.48E-15
ENSMUSG00000029570	Lfng	-0.759884012	0.026675292
ENSMUSG00000020053	Igf1	-0.765602173	5.44E-34
ENSMUSG00000037313	Tacc3	-0.765727335	1.83E-23
ENSMUSG00000063428	Ddo	-0.768089144	0.037869692
ENSMUSG00000023505	Cdca3	-0.772459435	8.80E-22
ENSMUSG00000008090	Fgfrl1	-0.773486453	7.53E-40
ENSMUSG00000029177	Cenpa	-0.773618243	1.99E-12
ENSMUSG00000085584	Rtl9	-0.774927214	0.048133127
ENSMUSG00000050503	Fbxl22	-0.779257998	0.00263447
ENSMUSG00000041431	Ccnb1	-0.7816684	8.39E-11
ENSMUSG00000001507	Itga3	-0.791053264	3.82E-27
ENSMUSG00000032380	Dapk2	-0.795268006	1.55E-06
ENSMUSG00000021388	Aspn	-0.797554753	3.42E-49
ENSMUSG00000021208	Ifi27l2b	-0.797613498	0.005765247
ENSMUSG00000023039	Krt7	-0.79843802	1.14E-05
ENSMUSG00000046807	Lrrc75b	-0.80064492	3.05E-14
ENSMUSG00000007097	Atp1a2	-0.80242266	2.44E-07
ENSMUSG00000031780	Ccl17	-0.804137194	4.52E-18
ENSMUSG00000059602	Syn3	-0.808485116	0.006587078
ENSMUSG00000019789	Hey2	-0.808761148	0.01429868
ENSMUSG00000042367	Gjb3	-0.809701337	3.77E-18
ENSMUSG00000028655	Mfsd2a	-0.814183343	2.12E-20
ENSMUSG00000029053	Prkcz	-0.814532944	0.012190424
ENSMUSG00000045672	Col27a1	-0.820339464	2.79E-08
ENSMUSG00000037544	Dlgap5	-0.820341186	2.17E-15
ENSMUSG00000030217	Art4	-0.823830862	4.63E-10
ENSMUSG00000051517	Arhgef39	-0.824297703	0.027095152
ENSMUSG00000004633	Chn2	-0.826010934	8.08E-05
ENSMUSG00000032221	Mns1	-0.826502674	3.24E-07
ENSMUSG00000005951	Shpk	-0.829401832	1.90E-08
ENSMUSG00000070469	Adamtsl3	-0.829424992	5.28E-43
ENSMUSG00000020155	Kcnmb1	-0.829927028	0.00042759
ENSMUSG00000021730	Hcn1	-0.830806115	0.015543517
ENSMUSG00000005357	Slc1a6	-0.832255965	9.57E-31
ENSMUSG00000073600	Prob1	-0.833126962	0.046188906
ENSMUSG00000029334	Prkg2	-0.838444134	0.000591899
ENSMUSG00000046447	Camk2n1	-0.840478429	1.68E-55
ENSMUSG00000020330	Hmmr	-0.841887423	3.39E-29
ENSMUSG00000111291	Gm48604	-0.842429511	0.02917004
ENSMUSG00000057606	Colq	-0.845929438	0.027024649
ENSMUSG00000030772	Dkk3	-0.847288611	8.47E-94
ENSMUSG00000033544	Angptl1	-0.847589231	0.01589704
ENSMUSG00000040666	Sh3bgr	-0.85138436	0.024505631
ENSMUSG00000027966	Col11a1	-0.853601863	2.44E-97
ENSMUSG00000034738	Nostrin	-0.859610321	0.006099599
ENSMUSG00000022240	Ctnnd2	-0.86373207	2.11E-07
ENSMUSG00000032135	Mcam	-0.867232661	1.84E-28
ENSMUSG00000040093	Bmf	-0.867344635	0.000388247
ENSMUSG00000021798	Ldb3	-0.868792277	0.000426792
ENSMUSG00000015733	Capza2	-0.871543584	3.04E-74
ENSMUSG00000032254	Kif23	-0.871584561	9.57E-11
ENSMUSG00000019577	Pdk4	-0.872165932	9.51E-14
ENSMUSG00000050014	Apol10b	-0.872749878	9.91E-05
ENSMUSG00000003779	Kif20a	-0.87386692	3.36E-36
ENSMUSG00000034203	Chchd4	-0.874735599	8.29E-10
ENSMUSG00000066705	Fxyd6	-0.875359986	1.49E-113
ENSMUSG00000108456	4732496C06Rik	-0.881564526	0.025307879
ENSMUSG00000017692	Rhbdl3	-0.886185087	0.012323607
ENSMUSG00000068522	Aard	-0.888485192	0.049703975
ENSMUSG00000001403	Ube2c	-0.894317662	1.50E-46
ENSMUSG00000035775	Krt20	-0.897893488	0.036273481
ENSMUSG00000027861	Casq2	-0.900925286	0.027564195
ENSMUSG00000054675	Tmem119	-0.903224494	1.32E-50
ENSMUSG00000053101	Gpr141	-0.903822632	0.040806684
ENSMUSG00000048782	Insc	-0.910736558	4.52E-07
ENSMUSG00000024206	Rfx2	-0.912941792	0.000901743
ENSMUSG00000029859	Epha1	-0.915757035	0.043722479
ENSMUSG00000026955	Sapcd2	-0.916640813	0.00302176
ENSMUSG00000006398	Cdc20	-0.91682003	2.00E-19
ENSMUSG00000037235	Mxd4	-0.918051145	4.17E-74
ENSMUSG00000067818	Myl9	-0.922779531	1.29E-118
ENSMUSG00000058669	Nkx2-9	-0.923717245	0.011282121
ENSMUSG00000021391	Cenpp	-0.926648416	0.017362884
ENSMUSG00000038943	Prc1	-0.928777494	5.19E-29
ENSMUSG00000004187	Kifc2	-0.711857847	0.019566887
ENSMUSG00000021747	4930452B06Rik	-0.712947524	0.045153012
ENSMUSG00000032092	Mpzl2	-0.713077	0.015361015
ENSMUSG00000042340	Ctf1	-0.713249928	0.002679545
ENSMUSG00000031965	Tbx20	-0.713499935	6.46E-60
ENSMUSG00000043110	Lrrn4	-0.714136909	3.84E-05
ENSMUSG00000042834	Nrep	-0.714477951	4.34E-12
ENSMUSG00000000686	Abhd15	-0.715210851	4.02E-05
ENSMUSG00000030621	Me3	-0.715410892	0.000106239
ENSMUSG00000029910	Mad2l1	-0.715942445	3.53E-14
ENSMUSG00000055745	Rtl6	-0.71594623	2.30E-09
ENSMUSG00000087088	Gm16638	-0.71616772	0.01340233
ENSMUSG00000029438	Bcl7a	-0.716229286	2.80E-05
ENSMUSG00000036672	Cenpt	-0.716333419	2.99E-17
ENSMUSG00000031861	Lpar2	-0.716536044	1.60E-07
ENSMUSG00000029869	Ephb6	-0.7174265	1.79E-11
ENSMUSG00000038152	5033430I15Rik	-0.718033538	0.037028446
ENSMUSG00000037493	Cib2	-0.71837741	0.001142361
ENSMUSG00000051339	2900026A02Rik	-0.718867994	1.36E-22
ENSMUSG00000021998	Lcp1	-0.718896781	6.82E-07
ENSMUSG00000026736	4930426L09Rik	-0.720227001	0.024556938
ENSMUSG00000021411	Pxdc1	-0.72168215	1.16E-27
ENSMUSG00000023886	Smoc2	-0.723243291	2.69E-88
ENSMUSG00000001119	Col6a1	-0.723275154	5.20E-39
ENSMUSG00000025856	Pdgfa	-0.723668636	6.11E-28
ENSMUSG00000040502	Marchf9	-0.723844511	7.64E-05
ENSMUSG00000030905	Crym	-0.725030783	5.62E-12
ENSMUSG00000025094	Slc18a2	-0.725179281	0.033349447
ENSMUSG00000009654	Oit3	-0.72528653	0.016686189
ENSMUSG00000027508	Pag1	-0.725527169	5.06E-06
ENSMUSG00000050222	Il17d	-0.727029794	0.001078767
ENSMUSG00000040552	C3ar1	-0.727366824	0.000202194
ENSMUSG00000047115	Fam221a	-0.727982864	0.016477042
ENSMUSG00000042812	Foxf1	-0.730948692	0.000540497
ENSMUSG00000040258	Nxph4	-0.731491537	0.041770969
ENSMUSG00000050777	Tmem37	-0.731811875	2.80E-18
ENSMUSG00000024300	Myo1f	-0.732048135	0.022787467
ENSMUSG00000032380	Dapk2	-0.732431817	2.95E-07
ENSMUSG00000072572	Slc39a2	-0.734455268	0.041961059
ENSMUSG00000021262	Evl	-0.7346516	1.80E-12
ENSMUSG00000000223	Drp2	-0.73518456	5.26E-05
ENSMUSG00000072980	Oip5	-0.735618304	0.014991028
ENSMUSG00000056394	Lig1	-0.736713163	5.80E-15
ENSMUSG00000007655	Cav1	-0.737837909	4.92E-35
ENSMUSG00000029765	Plxna4	-0.738734004	3.98E-25
ENSMUSG00000049791	Fzd4	-0.740839171	1.13E-10
ENSMUSG00000036339	Tmem260	-0.741161761	4.42E-15
ENSMUSG00000075593	Gal3st4	-0.741194018	0.000536588
ENSMUSG00000024663	Rab3il1	-0.741876662	1.87E-20
ENSMUSG00000043252	Tmem64	-0.742271426	2.20E-27
ENSMUSG00000022034	Esco2	-0.74314313	0.004712222
ENSMUSG00000052397	Ezr	-0.743345988	4.68E-47
ENSMUSG00000036565	Ttyh3	-0.743880779	5.11E-22
ENSMUSG00000046668	Cxxc5	-0.744094708	3.20E-20
ENSMUSG00000097961	Gm27000	-0.746471926	0.046344693
ENSMUSG00000108350	Gm44950	-0.746669563	0.048301342
ENSMUSG00000031805	Jak3	-0.749031645	2.06E-09
ENSMUSG00000045658	Pid1	-0.749517215	1.06E-31
ENSMUSG00000041889	Shisa4	-0.751075288	1.61E-31
ENSMUSG00000057604	Lmcd1	-0.75315165	1.01E-33
ENSMUSG00000067586	S1pr3	-0.754321022	2.44E-05
ENSMUSG00000026831	1700007K13Rik	-0.755041979	6.96E-09
ENSMUSG00000044006	Cilp2	-0.755338888	0.039702294
ENSMUSG00000022206	Npr3	-0.756317838	8.80E-05
ENSMUSG00000054717	Hmgb2	-0.759638598	4.69E-12
ENSMUSG00000030688	Stard10	-0.759985461	2.53E-08
ENSMUSG00000062591	Tubb4a	-0.761575751	4.11E-28
ENSMUSG00000028702	Rad54l	-0.762209934	1.45E-05
ENSMUSG00000051220	Ercc6l	-0.762477572	0.00011176
ENSMUSG00000112794	Gm48878	-0.762544967	0.001877191
ENSMUSG00000059602	Syn3	-0.762717482	0.010542271
ENSMUSG00000025650	Col7a1	-0.764244535	5.84E-13
ENSMUSG00000035493	Tgfbi	-0.76610827	1.16E-14
ENSMUSG00000048779	P2ry6	-0.766508091	1.61E-10
ENSMUSG00000104476	Gm38211	-0.766611468	0.021899859
ENSMUSG00000023345	Poc1a	-0.766789165	0.000179473
ENSMUSG00000029730	Mcm7	-0.767299222	2.81E-23

ENSMUSG00000059645	Gm7361	-0.930283472	0.031368436
ENSMUSG00000015222	Map2	-0.930596884	2.34E-26
ENSMUSG00000025789	St8sia2	-0.930741564	0.008433449
ENSMUSG00000039542	Ncam1	-0.935703955	2.31E-77
ENSMUSG00000030701	Plekhb1	-0.936488781	0.00988407
ENSMUSG00000040310	Alx4	-0.937058256	0.002491289
ENSMUSG00000024525	Impa2	-0.938213883	2.64E-05
ENSMUSG00000050271	Prag1	-0.939686896	3.79E-56
ENSMUSG00000062785	Kcnc3	-0.949913749	0.042088707
ENSMUSG00000079317	Trappc2	-0.952647113	6.67E-24
ENSMUSG00000018849	Wwc1	-0.953839043	4.50E-11
ENSMUSG00000004892	Bcan	-0.95693294	0.008928847
ENSMUSG00000023232	Serinc2	-0.965168436	4.46E-20
ENSMUSG00000027499	Pkia	-0.967789435	4.44E-31
ENSMUSG00000044365	Cxxc4	-0.970799953	0.00585108
ENSMUSG00000031098	Syt8	-0.971003534	0.013666986
ENSMUSG00000032218	Ccnb2	-0.971578297	4.30E-26
ENSMUSG00000017446	C1qtnf1	-0.974523459	1.81E-11
ENSMUSG00000039476	Prrx2	-0.976954845	3.77E-05
ENSMUSG00000021057	Akap5	-0.978301758	3.50E-14
ENSMUSG00000036596	Cpz	-0.98403629	0.002466937
ENSMUSG00000043333	Rhbdl2	-0.995955726	0.000183513
ENSMUSG00000032374	Plod2	-1.006486602	4.67E-192
ENSMUSG00000021322	Aoah	-1.009674125	0.006736324
ENSMUSG00000051041	Olfml1	-1.011758255	8.64E-08
ENSMUSG00000070348	Ccnd1	-1.017237632	9.19E-119
ENSMUSG00000027329	Spef1	-1.018146202	9.60E-08
ENSMUSG00000105176	Gm43668	-1.018296476	0.042794625
ENSMUSG00000020150	Gamt	-1.024560197	2.67E-07
ENSMUSG00000031137	Fgf13	-1.034012662	2.08E-05
ENSMUSG00000057751	Megf6	-1.034126383	2.03E-13
ENSMUSG00000001349	Cnn1	-1.035913803	6.00E-16
ENSMUSG00000009394	Syn2	-1.041215803	3.10E-19
ENSMUSG00000018648	Dusp14	-1.044951451	1.16E-08
ENSMUSG00000041064	Pif1	-1.04498255	1.68E-06
ENSMUSG00000027107	Chrna1	-1.052867148	3.23E-05
ENSMUSG00000030607	Acan	-1.057506637	0.00014804
ENSMUSG00000022296	Baalc	-1.058265886	3.11E-05
ENSMUSG00000027335	Adra1d	-1.061831534	0.00429019
ENSMUSG00000021803	Cdhr1	-1.063174022	0.007798508
ENSMUSG00000009214	Mymk	-1.064344913	0.045763179
ENSMUSG00000104476	Gm38211	-1.069454579	0.000813619
ENSMUSG00000027875	Hmgcs2	-1.071039815	0.000742878
ENSMUSG00000032291	Crabp1	-1.071068738	3.31E-06
ENSMUSG00000071604	Fam189a2	-1.07282144	3.06E-10
ENSMUSG00000071112	Spx	-1.072855224	0.014227504
ENSMUSG00000086555	Gm13446	-1.074827947	0.028887731
ENSMUSG00000068859	Sp9	-1.076349793	0.001615016
ENSMUSG00000044201	Cdc25c	-1.080430193	2.09E-07
ENSMUSG00000036913	Trim67	-1.081692681	0.034493425
ENSMUSG00000031230	Fgf16	-1.084257196	0.002779913
ENSMUSG00000030867	Plk1	-1.090582444	2.00E-53
ENSMUSG00000024697	Gna14	-1.094453927	0.000103455
ENSMUSG00000108634	Gm38534	-1.107513808	0.012097484
ENSMUSG00000026068	Il18rap	-1.108270163	0.026694745
ENSMUSG00000103932	Gm36963	-1.110389671	0.019062445
ENSMUSG00000020793	Galr2	-1.124041871	0.017111672
ENSMUSG00000067561	Dmrtc1c2	-1.127613116	0.032124856
ENSMUSG00000037336	Mfsd2b	-1.127701297	0.000824437
ENSMUSG00000044005	Gls2	-1.135103977	0.01158167
ENSMUSG00000044139	Prss53	-1.138873476	0.002189204
ENSMUSG00000029120	Ppp2r2c	-1.140165754	0.019324059
ENSMUSG00000051457	Spn	-1.140301963	6.54E-06
ENSMUSG00000072720	Myo18b	-1.140556284	2.26E-05
ENSMUSG00000032744	Heyl	-1.153782312	7.70E-21
ENSMUSG00000067562	Dmrtc1c1	-1.155882078	0.038755094
ENSMUSG00000048582	Gja3	-1.156803051	0.000174975
ENSMUSG00000048939	Atp13a5	-1.164628804	1.81E-07
ENSMUSG00000051359	Ncald	-1.177823213	1.18E-18
ENSMUSG00000039883	Lrrc17	-1.185731826	0.000117319
ENSMUSG00000028782	Adgrb2	-1.18763368	9.70E-05
ENSMUSG00000042109	Csdc2	-1.190096268	5.33E-73
ENSMUSG00000064272	Gpbar1	-1.191626372	0.019883503
ENSMUSG00000042115	Klhdc8a	-1.197055812	6.15E-06
ENSMUSG00000025650	Col7a1	-1.197562439	2.47E-32
ENSMUSG00000035104	Eva1a	-1.205219744	0.000982496
ENSMUSG00000049538	Adamts16	-1.206363683	0.048551082

ENSMUSG00000007039	Ddah2	-0.767931676	1.12E-25
ENSMUSG00000038168	P3h2	-0.769290129	1.22E-09
ENSMUSG00000030793	Pycard	-0.769445098	0.000396893
ENSMUSG00000022881	Rfc4	-0.770103773	0.000809427
ENSMUSG00000058290	Espl1	-0.770919266	9.78E-08
ENSMUSG00000025207	Sema4g	-0.771135675	0.021518108
ENSMUSG00000019851	Perp	-0.77133084	4.72E-12
ENSMUSG00000005355	Casp14	-0.772217995	0.014836952
ENSMUSG00000033295	Ptprf	-0.772871679	3.78E-35
ENSMUSG00000026196	Bard1	-0.773412498	0.00657301
ENSMUSG00000032400	Zwilch	-0.774106466	9.51E-14
ENSMUSG00000035842	Ddx11	-0.776349242	6.08E-05
ENSMUSG00000031070	Mrgprf	-0.777033773	2.88E-08
ENSMUSG00000087408	Cers1	-0.777192114	0.00759167
ENSMUSG00000060600	Eno3	-0.77747417	1.57E-05
ENSMUSG00000025701	Alox5	-0.777528968	0.003168363
ENSMUSG00000103332	Pcdhga2	-0.778825634	0.040245983
ENSMUSG00000030096	Slc6a6	-0.778905243	2.84E-91
ENSMUSG00000027353	Mcm8	-0.779113305	0.005092104
ENSMUSG00000020492	Ska2	-0.781333843	5.91E-15
ENSMUSG00000071547	Nt5dc2	-0.782120793	1.55E-26
ENSMUSG00000032744	Heyl	-0.783346039	4.56E-10
ENSMUSG00000038552	Fndc4	-0.783492576	0.014745521
ENSMUSG00000020173	Cobl	-0.783523529	2.90E-05
ENSMUSG00000020649	Rrm2	-0.783806409	1.59E-27
ENSMUSG00000028251	Tstd3	-0.785729436	8.00E-12
ENSMUSG00000028497	Hacd4	-0.786451553	5.24E-25
ENSMUSG00000005950	P2rx5	-0.787836476	7.88E-05
ENSMUSG00000054889	Dsp	-0.789069639	1.73E-10
ENSMUSG00000026315	Serpinb8	-0.78931314	2.75E-09
ENSMUSG00000019230	Lhx9	-0.789756813	0.019393648
ENSMUSG00000056481	Cd248	-0.789814666	6.02E-20
ENSMUSG00000028664	Ephb2	-0.790414612	3.83E-19
ENSMUSG00000004473	Clec11a	-0.79069954	3.75E-08
ENSMUSG00000026986	Hnmt	-0.791474635	0.024227238
ENSMUSG00000027199	Gatm	-0.794247751	0.005245904
ENSMUSG00000045005	Fzd5	-0.79713604	9.58E-20
ENSMUSG00000031698	Mylk3	-0.797408402	0.036042475
ENSMUSG00000001655	Hoxc13	-0.797573854	0.002462294
ENSMUSG00000045667	Smtnl2	-0.798850979	1.09E-12
ENSMUSG00000055809	Dnaaf3	-0.799373782	2.98E-05
ENSMUSG00000024501	Dpysl3	-0.799529555	5.21E-79
ENSMUSG00000032841	Prr5l	-0.800079236	2.40E-05
ENSMUSG00000055980	Irs1	-0.80016674	2.41E-23
ENSMUSG00000024043	Arhgap28	-0.800228982	4.45E-14
ENSMUSG00000027858	Tspan2	-0.800365299	6.93E-10
ENSMUSG00000034258	Flvcr2	-0.800999459	0.00691934
ENSMUSG00000055653	Gpc3	-0.80107137	0.000135218
ENSMUSG00000063873	Slc24a3	-0.801311484	8.97E-22
ENSMUSG00000030319	Cand2	-0.801869568	2.79E-20
ENSMUSG00000027932	Slc27a3	-0.802658221	8.29E-13
ENSMUSG00000049630	C1ql3	-0.803461894	6.91E-06
ENSMUSG00000020098	Pcbd1	-0.804412853	0.001761145
ENSMUSG00000020900	Myh10	-0.804966724	5.04E-42
ENSMUSG00000027134	Lpcat4	-0.805288464	1.09E-22
ENSMUSG00000097015	Gm26705	-0.806723044	0.010762171
ENSMUSG00000097789	Gm2115	-0.807765014	8.66E-05
ENSMUSG00000033763	Mtss2	-0.808573748	1.54E-27
ENSMUSG00000051048	P4ha3	-0.809198711	9.66E-20
ENSMUSG00000034792	Gna15	-0.809450595	0.01597466
ENSMUSG00000030091	Nup210	-0.809573954	0.027865996
ENSMUSG00000022123	Scel	-0.809821293	6.47E-45
ENSMUSG00000112148	Lilrb4a	-0.810400895	1.83E-06
ENSMUSG00000000325	Arvcf	-0.81069091	4.85E-15
ENSMUSG00000024697	Gna14	-0.810968863	0.004159813
ENSMUSG00000089812	Gm15867	-0.811119976	0.022878159
ENSMUSG00000049281	Scn3b	-0.812566533	5.31E-06
ENSMUSG00000025574	Tk1	-0.812591092	2.35E-10
ENSMUSG00000048040	Arxes2	-0.812695282	4.80E-22
ENSMUSG00000013415	Igf2bp1	-0.812872014	0.000213915
ENSMUSG00000094248	Hist1h2ao	-0.813404699	0.004553233
ENSMUSG00000018819	Lsp1	-0.81543627	2.30E-76
ENSMUSG00000024795	Kif20b	-0.816050909	6.46E-11
ENSMUSG00000043008	Klhl6	-0.81648759	7.57E-29
ENSMUSG00000049001	Ndnf	-0.816980141	1.85E-05
ENSMUSG00000048216	Gpr85	-0.817257423	0.000186177
ENSMUSG00000039556	Ppp1r3f	-0.817968469	0.002967985

ENSMUSG00000100980	Gm29100	-1.206860602	0.014166864
ENSMUSG00000094543	Gm8212	-1.210877024	0.044729509
ENSMUSG00000054196	Cthrc1	-1.228578669	4.74E-90
ENSMUSG00000025610	Map3k7cl	-1.235857572	3.76E-31
ENSMUSG00000055027	Smyd1	-1.236000835	0.01081218
ENSMUSG00000026051	Ecrg4	-1.259318249	5.44E-09
ENSMUSG00000035615	Frmpd1	-1.260727985	1.68E-15
ENSMUSG00000030827	Fgf21	-1.263028974	2.32E-08
ENSMUSG00000053647	Gper1	-1.267712936	4.38E-08
ENSMUSG00000084274	Gm12504	-1.270967306	0.013314348
ENSMUSG00000021703	Serinc5	-1.296887083	2.39E-43
ENSMUSG00000032017	Grik4	-1.324235129	0.035788749
ENSMUSG00000032202	Rab27a	-1.325253306	0.000384385
ENSMUSG00000047443	Erfe	-1.325928098	8.31E-42
ENSMUSG00000021423	Ly86	-1.333248333	0.005444188
ENSMUSG00000053025	Sv2b	-1.334271633	0.001949384
ENSMUSG00000040086	Tnni3k	-1.345154783	2.05E-10
ENSMUSG00000024210	Ip6k3	-1.346949934	0.000207223
ENSMUSG00000026564	Dusp27	-1.34813233	7.18E-09
ENSMUSG00000028558	Calr4	-1.354921676	3.48E-14
ENSMUSG00000029352	Crybb3	-1.361398585	0.016571195
ENSMUSG00000091476	Catspere2	-1.369275659	0.008016185
ENSMUSG00000037962	Rflna	-1.36941271	0.009852879
ENSMUSG00000090122	Kcne1l	-1.374087274	2.23E-08
ENSMUSG00000037379	Spon2	-1.383893359	1.17E-42
ENSMUSG00000075408	Smim41	-1.386084124	0.00241273
ENSMUSG00000036295	Lrrn3	-1.393410381	0.027811424
ENSMUSG00000054934	Kcnmb4	-1.401083041	0.017660625
ENSMUSG00000002324	Rec8	-1.409457963	0.003076884
ENSMUSG00000044550	Tceal3	-1.413109503	0.000283232
ENSMUSG00000024910	Ctsw	-1.419501788	7.82E-06
ENSMUSG00000025475	Adgra1	-1.424210196	2.92E-09
ENSMUSG00000079173	Zan	-1.448442751	0.034477819
ENSMUSG00000113909	Gm36377	-1.465538768	6.10E-22
ENSMUSG00000036040	Adamtsl2	-1.467912281	1.56E-153
ENSMUSG00000021118	Plek2	-1.467934809	0.031237852
ENSMUSG00000028626	Col9a2	-1.523898798	0.015526086
ENSMUSG00000024176	Sox8	-1.56483589	4.86E-07
ENSMUSG00000115232	Gm49378	-1.638237014	3.51E-06
ENSMUSG00000026468	Lhx4	-1.647437586	0.004380118
ENSMUSG00000026308	Klhl30	-1.687017948	8.88E-72
ENSMUSG00000106062	Gm43820	-1.736674819	0.010700949
ENSMUSG00000070644	Etnk2	-1.739136691	0.01784128
ENSMUSG00000000889	Dbh	-1.742627203	3.33E-06
ENSMUSG00000033998	Kcnk1	-1.793962883	2.54E-07
ENSMUSG00000021751	Acox2	-1.825946645	0.000304877
ENSMUSG00000097239	Gm27029	-1.830271182	0.005951526
ENSMUSG00000058914	C1qtnf3	-1.880027604	8.50E-14
ENSMUSG00000031553	Adam3	-2.006224632	0.003761073
ENSMUSG00000085008	Dbhos	-2.076792841	5.98E-06
ENSMUSG00000090394	4930523C07Rik	-2.235611166	2.00E-91
ENSMUSG00000094365	Gm21982	-2.343414056	0.000347159
ENSMUSG00000006542	Prkag3	-2.402789285	0.000129571
ENSMUSG00000062393	Dgkk	-2.510353905	2.16E-07

ENSMUSG00000084128	Esrp2	-0.81821334	6.90E-08
ENSMUSG00000019961	Tmpo	-0.82088235	4.45E-40
ENSMUSG00000027966	Col11a1	-0.821004367	5.53E-74
ENSMUSG00000037060	Cavin3	-0.821468614	2.10E-43
ENSMUSG00000039323	Igfbp2	-0.82326034	2.34E-44
ENSMUSG00000046179	E2f8	-0.824058244	1.78E-06
ENSMUSG00000019303	Psmc3ip	-0.824276268	0.009767237
ENSMUSG00000074121	Ntf5	-0.826579172	3.53E-05
ENSMUSG00000022840	Adcy5	-0.826714847	0.028584012
ENSMUSG00000022040	Ephx2	-0.827303016	3.38E-15
ENSMUSG00000005470	Asf1b	-0.827344426	1.38E-10
ENSMUSG00000027323	Rad51	-0.828123171	8.32E-10
ENSMUSG00000042190	Cmklr1	-0.828137592	1.68E-21
ENSMUSG00000041219	Arhgap11a	-0.828527169	6.46E-21
ENSMUSG00000097124	A530020G20Rik	-0.829472458	0.029455974
ENSMUSG00000024791	Cdca5	-0.831425137	2.43E-06
ENSMUSG00000039542	Ncam1	-0.831868991	4.24E-43
ENSMUSG00000024534	Sncaip	-0.83302818	0.000719179
ENSMUSG00000034206	Polq	-0.834053747	5.26E-05
ENSMUSG00000076437	Selenoh	-0.840043202	2.31E-08
ENSMUSG00000063632	Sox11	-0.841665974	0.000581778
ENSMUSG00000070044	Fam149a	-0.841880771	9.21E-37
ENSMUSG00000094777	Hist1h2ap	-0.842124076	0.000469828
ENSMUSG00000043165	Lor	-0.842729023	0.003860669
ENSMUSG00000018012	Rac3	-0.843870824	0.005023081
ENSMUSG00000067158	Col4a4	-0.844107026	2.77E-21
ENSMUSG00000043556	Fbxl7	-0.844967624	1.12E-14
ENSMUSG00000097697	4833412C05Rik	-0.845087009	0.010268377
ENSMUSG00000051817	Sox12	-0.846000797	4.78E-10
ENSMUSG00000037813	D630003M21Rik	-0.846394065	0.001510065
ENSMUSG00000020990	Cdkl1	-0.846939559	0.008426697
ENSMUSG00000027330	Cdc25b	-0.847888089	4.64E-09
ENSMUSG00000045273	Cenph	-0.848908735	4.41E-05
ENSMUSG00000030701	Plekhb1	-0.849497346	0.039017423
ENSMUSG00000027253	Lrp4	-0.850222323	1.40E-12
ENSMUSG00000027827	Kcnab1	-0.850858438	3.59E-44
ENSMUSG00000034413	Neurl1b	-0.857083953	0.016687557
ENSMUSG00000028873	Cdca8	-0.857670841	3.10E-12
ENSMUSG00000033207	Mamdc2	-0.859249572	5.40E-39
ENSMUSG00000030109	Slc6a12	-0.860510672	0.000374701
ENSMUSG00000043333	Rhbdl2	-0.860621695	0.015366377
ENSMUSG00000025006	Sorbs1	-0.86191336	1.33E-15
ENSMUSG00000039899	Fgl2	-0.86299001	0.000395124
ENSMUSG00000020150	Gamt	-0.864222172	0.001074952
ENSMUSG00000043496	Tril	-0.86497527	0.007646204
ENSMUSG00000002012	Pnck	-0.865344509	0.042762093
ENSMUSG00000039748	Exo1	-0.866045707	0.000377841
ENSMUSG00000004880	Lbr	-0.866772557	8.73E-15
ENSMUSG00000022021	Diaph3	-0.867780043	2.14E-19
ENSMUSG00000070469	Adamtsl3	-0.868821096	5.95E-46
ENSMUSG00000023908	Pkmyt1	-0.868838038	9.03E-13
ENSMUSG00000041362	Shtn1	-0.874960894	1.10E-05
ENSMUSG00000113853	Gm47583	-0.875927131	0.039702294
ENSMUSG00000020674	Pxdn	-0.876362419	5.41E-71
ENSMUSG00000078794	Dact3	-0.876679899	1.91E-46
ENSMUSG00000093979	Gm2237	-0.876782719	0.016007915
ENSMUSG00000031636	Pdlim3	-0.878291878	2.39E-29
ENSMUSG00000026870	Cutal	-0.87892729	0.04609678
ENSMUSG00000053886	Sh2d4a	-0.879257917	3.18E-06
ENSMUSG00000038677	Scube3	-0.880214482	0.013213321
ENSMUSG00000023919	Cenpq	-0.880900458	1.39E-06
ENSMUSG00000041757	Plekha6	-0.882376749	3.47E-06
ENSMUSG00000007080	Pole	-0.882681412	6.95E-11
ENSMUSG00000061533	Cep128	-0.883021002	7.84E-08
ENSMUSG00000048355	Arxes1	-0.883288394	9.89E-13
ENSMUSG00000052504	Epha3	-0.883391613	0.00183836
ENSMUSG00000025076	Casp7	-0.885129865	1.31E-16
ENSMUSG00000002104	Rapsn	-0.885598503	2.31E-05
ENSMUSG00000003070	Efna2	-0.887396083	0.006170279
ENSMUSG00000020155	Kcnmb1	-0.887485473	0.000223495
ENSMUSG00000006342	Susd2	-0.890177094	3.27E-35
ENSMUSG00000074743	Thbd	-0.890599545	9.03E-42
ENSMUSG00000078521	Aunip	-0.891498453	0.020765729
ENSMUSG00000024747	Aldh1a7	-0.892585369	0.002224597
ENSMUSG00000035413	Tmem98	-0.892638666	9.28E-79
ENSMUSG00000020102	Slc16a7	-0.894745253	0.049888021
ENSMUSG00000032085	Tagln	-0.896692999	2.81E-113

ENSMUSG00000027977	Ndst3	-0.897236038	0.000376929
ENSMUSG00000027160	Ccdc34	-0.898185928	4.24E-12
ENSMUSG00000016756	Cmah	-0.898588522	0.003383258
ENSMUSG00000068762	Gstm6	-0.899648191	7.74E-09
ENSMUSG00000025352	Gdf11	-0.899664305	3.23E-15
ENSMUSG00000057967	Fgf18	-0.902515171	2.56E-08
ENSMUSG00000038816	Ctnnal1	-0.902579062	2.80E-11
ENSMUSG00000047534	Mis18bp1	-0.904547877	6.68E-09
ENSMUSG00000042251	Pm20d1	-0.904789823	1.71E-15
ENSMUSG00000039396	Neil3	-0.904881847	1.71E-07
ENSMUSG00000058396	Gpr182	-0.905716495	0.044042407
ENSMUSG00000041957	Pkp2	-0.906116179	1.81E-05
ENSMUSG00000005410	Mcm5	-0.906646759	2.58E-22
ENSMUSG00000021803	Cdhr1	-0.906648216	0.032273207
ENSMUSG00000021253	Tgfb3	-0.906759909	1.61E-78
ENSMUSG00000002274	Metrn	-0.908786278	7.08E-14
ENSMUSG00000042985	Upk3b	-0.9087891	5.05E-13
ENSMUSG00000046694	Tent5b	-0.910280857	0.000182293
ENSMUSG00000027811	4930579G24Rik	-0.910343179	2.96E-07
ENSMUSG00000018830	Myh11	-0.910759603	3.73E-05
ENSMUSG00000035683	Melk	-0.912860902	2.95E-10
ENSMUSG00000005233	Spc25	-0.913302612	1.57E-19
ENSMUSG00000035456	Prdm8	-0.914602159	0.033427318
ENSMUSG00000051832	E230016K23Rik	-0.914879424	0.002401357
ENSMUSG00000020475	Pgam2	-0.91533669	0.019894336
ENSMUSG00000022322	Shcbp1	-0.916134045	3.31E-08
ENSMUSG00000002058	Unc119	-0.917101337	7.82E-39
ENSMUSG00000104279	Gm38300	-0.918950778	0.042562353
ENSMUSG00000022836	Mylk	-0.920922395	6.02E-08
ENSMUSG00000045672	Col27a1	-0.921568264	3.09E-10
ENSMUSG00000098650	Commd1b	-0.922791084	0.021872091
ENSMUSG00000030217	Art4	-0.922796184	3.87E-09
ENSMUSG00000050503	Fbxl22	-0.927210896	0.000600681
ENSMUSG00000027220	Syt13	-0.927277188	4.04E-05
ENSMUSG00000026779	Mastl	-0.927576489	1.79E-16
ENSMUSG00000039239	Tgfb2	-0.928087601	4.28E-41
ENSMUSG00000042155	Klhl23	-0.928388573	0.013011639
ENSMUSG00000046447	Camk2n1	-0.929152958	3.47E-77
ENSMUSG00000000037	Scml2	-0.92933902	0.010834127
ENSMUSG00000035455	Fignl1	-0.929956587	3.58E-05
ENSMUSG00000048368	Omd	-0.930189838	0.003415987
ENSMUSG00000027676	Ccdc39	-0.932137768	0.006553541
ENSMUSG00000090125	Pou3f1	-0.932292555	0.001790775
ENSMUSG00000015647	Lama5	-0.934064511	1.84E-29
ENSMUSG00000022033	Pbk	-0.934163547	2.07E-08
ENSMUSG00000030346	Rad51ap1	-0.934611292	1.06E-06
ENSMUSG00000041406	BC055324	-0.934972564	0.000334209
ENSMUSG00000009628	Tex15	-0.936527138	0.025630245
ENSMUSG00000055761	Nkain3	-0.937098099	0.007370859
ENSMUSG00000059645	Gm7361	-0.937199342	0.024705138
ENSMUSG00000066705	Fxyd6	-0.937681303	1.39E-111
ENSMUSG00000072082	Ccnf	-0.938421463	5.38E-09
ENSMUSG00000032172	Olfm2	-0.939257352	1.87E-18
ENSMUSG00000037020	Wdr62	-0.939295966	1.04E-17
ENSMUSG00000074476	Spc24	-0.93981074	4.88E-07
ENSMUSG00000040084	Bub1b	-0.940120597	2.39E-14
ENSMUSG00000029093	Sorcs2	-0.940532042	2.69E-08
ENSMUSG00000026463	Atp2b4	-0.940609502	1.04E-08
ENSMUSG00000060586	H2-Eb1	-0.941015899	0.027961562
ENSMUSG00000031119	Gpc4	-0.94229221	2.97E-107
ENSMUSG00000042745	Id1	-0.942670732	1.58E-13
ENSMUSG00000029436	Mmp17	-0.942951643	0.000853746
ENSMUSG00000062785	Kcnc3	-0.947739595	0.049429877
ENSMUSG00000027499	Pkia	-0.94827347	2.85E-31
ENSMUSG00000025154	Arhgap19	-0.948345913	0.005176706
ENSMUSG00000039476	Prrx2	-0.949991652	2.71E-05
ENSMUSG00000001517	Foxm1	-0.950312709	4.34E-21
ENSMUSG00000032648	Pygm	-0.950681403	0.000965978
ENSMUSG00000024579	Pcyox1l	-0.95174551	3.76E-12
ENSMUSG00000048939	Atp13a5	-0.951770546	1.99E-06
ENSMUSG00000023909	Paqr4	-0.952155361	4.99E-15
ENSMUSG00000030515	Tarsl2	-0.952576758	2.07E-09
ENSMUSG00000031637	Lrp2bp	-0.953478965	0.016762034
ENSMUSG00000029716	Tfr2	-0.953527168	1.31E-06
ENSMUSG00000102752	Gm7694	-0.956565301	0.022381423
ENSMUSG00000058656	Samd12	-0.957174684	2.32E-05
ENSMUSG00000038252	Ncapd2	-0.957849419	3.21E-19

ENSMUSG00000038379	Ttk	-0.958525123	9.60E-08
ENSMUSG00000046591	Ticrr	-0.961607796	0.001120508
ENSMUSG00000041559	Fmod	-0.962527987	4.11E-88
ENSMUSG00000037552	Plekhg2	-0.965191008	4.76E-114
ENSMUSG00000116716	Gm49643	-0.965342363	0.007373628
ENSMUSG00000037035	Inhbb	-0.965424836	1.71E-20
ENSMUSG00000034906	Ncaph	-0.965781121	2.28E-15
ENSMUSG00000043079	Synpo	-0.967269269	5.42E-97
ENSMUSG00000082226	Gm715	-0.967985632	0.014576892
ENSMUSG00000040350	Trim7	-0.968336141	8.72E-18
ENSMUSG00000000359	Rem1	-0.968391945	0.000338003
ENSMUSG00000103037	Pcdhgb1	-0.968984957	0.00376797
ENSMUSG00000055148	Klf2	-0.969387344	1.51E-12
ENSMUSG00000042757	Tmem108	-0.970376931	0.010646672
ENSMUSG00000029838	Ptn	-0.971022388	2.63E-05
ENSMUSG00000086746	Gm15222	-0.972127848	0.002761679
ENSMUSG00000017716	Birc5	-0.972248013	2.18E-13
ENSMUSG00000039257	Vstm2b	-0.972421641	0.003886104
ENSMUSG00000024998	Plce1	-0.975519982	2.24E-19
ENSMUSG00000042284	Itga1	-0.976674192	2.82E-31
ENSMUSG00000031906	Smpd3	-0.977340874	1.23E-07
ENSMUSG00000030616	Sytl2	-0.977775941	1.14E-09
ENSMUSG00000062694	Cav3	-0.979739895	5.32E-06
ENSMUSG00000062510	Nsl1	-0.981639987	0.00061706
ENSMUSG00000020053	Igf1	-0.982027751	3.10E-58
ENSMUSG00000034245	Hdac11	-0.98255746	2.35E-07
ENSMUSG00000029381	Shroom3	-0.98284881	6.94E-25
ENSMUSG00000062826	Ces2f	-0.983023082	2.80E-05
ENSMUSG00000020182	Ddc	-0.983362556	0.002690637
ENSMUSG00000049758	Olfr1318	-0.983453537	0.012461562
ENSMUSG00000040260	Daam2	-0.983914672	9.16E-05
ENSMUSG00000032128	Robo3	-0.985369077	0.026805178
ENSMUSG00000028716	Pdzk1ip1	-0.986985508	0.01934719
ENSMUSG00000102543	Pcdhgc5	-0.989401605	0.010731562
ENSMUSG00000030623	Prss23os	-0.989560365	0.010622684
ENSMUSG00000026077	Npas2	-0.990131598	6.98E-05
ENSMUSG00000028068	Iqgap3	-0.991394476	5.01E-21
ENSMUSG00000021974	Fgf9	-0.99148908	9.89E-08
ENSMUSG00000047281	Sfn	-0.992332546	6.27E-25
ENSMUSG00000040093	Bmf	-0.993138287	0.00069295
ENSMUSG00000034161	Scx	-0.994270899	1.33E-18
ENSMUSG00000003779	Kif20a	-0.994564045	8.81E-43
ENSMUSG00000030849	Fgfr2	-0.995425766	2.68E-05
ENSMUSG00000031230	Fgf16	-0.996275112	0.010837764
ENSMUSG00000028718	Stil	-0.997243916	9.99E-13
ENSMUSG00000035407	Kank4	-0.997353709	0.027006655
ENSMUSG00000050556	Kcnb1	-0.997430938	0.000917804
ENSMUSG00000023940	Sgo1	-0.998332336	1.68E-06
ENSMUSG00000034685	Fam171a2	-1.000085258	9.53E-13
ENSMUSG00000039376	Synpo2l	-1.000098469	0.026415611
ENSMUSG00000017718	Afmid	-1.001198491	0.000144597
ENSMUSG00000027326	Knl1	-1.002041186	4.77E-06
ENSMUSG00000024056	Ndc80	-1.002821461	8.78E-08
ENSMUSG00000052396	Mogat2	-1.003162848	2.75E-18
ENSMUSG00000022546	Gpt	-1.003869779	0.00416484
ENSMUSG00000079654	Prrt4	-1.003952425	2.33E-17
ENSMUSG00000059323	Tonsl	-1.004064602	1.74E-07
ENSMUSG00000027715	Ccna2	-1.005114889	2.19E-22
ENSMUSG00000111521	Gm48529	-1.005467629	0.005663979
ENSMUSG00000027496	Aurka	-1.006146613	6.75E-24
ENSMUSG00000032586	Traip	-1.006293113	5.32E-08
ENSMUSG00000079553	Kifc1	-1.006356622	1.38E-23
ENSMUSG00000071847	Apcdd1	-1.008538975	6.61E-13
ENSMUSG00000048387	Osr1	-1.008801447	3.36E-36
ENSMUSG00000028687	Mutyh	-1.009067123	0.007883394
ENSMUSG00000038146	Notch3	-1.010450264	6.74E-28
ENSMUSG00000028655	Mfsd2a	-1.011814368	3.41E-19
ENSMUSG00000022803	Popdc2	-1.013657316	1.48E-09
ENSMUSG00000081137	BC022960	-1.014696812	0.003578853
ENSMUSG00000018648	Dusp14	-1.015006842	2.90E-07
ENSMUSG00000075590	Nrbp2	-1.015162378	3.65E-19
ENSMUSG00000019577	Pdk4	-1.015827822	2.80E-20
ENSMUSG00000031756	Cenpn	-1.019647982	2.42E-09
ENSMUSG00000019945	Cabcoco1	-1.0200573	0.025897148
ENSMUSG00000021259	Cyp46a1	-1.021288808	0.049161267
ENSMUSG00000068101	Cenpm	-1.021797932	6.89E-06
ENSMUSG00000040037	Negr1	-1.02180014	0.023294501

ENSMUSG00000036777	Anln	-1.021976279	2.51E-42
ENSMUSG00000021965	Ska3	-1.022538324	3.18E-06
ENSMUSG00000039943	Plcb4	-1.02310922	2.40E-50
ENSMUSG00000085584	Rtl9	-1.023562569	0.006166999
ENSMUSG00000030677	Kif22	-1.024118271	1.38E-20
ENSMUSG00000114053	Gm35330	-1.028127241	0.035836517
ENSMUSG00000029671	Wnt16	-1.03166947	0.000110906
ENSMUSG00000019942	Cdk1	-1.03182417	4.41E-53
ENSMUSG00000026622	Nek2	-1.032338641	1.11E-15
ENSMUSG00000037725	Ckap2	-1.03250191	2.66E-47
ENSMUSG00000021453	Gadd45g	-1.033259286	5.05E-122
ENSMUSG00000020963	Tshr	-1.034780661	0.003032712
ENSMUSG00000031004	Mki67	-1.037768252	4.26E-20
ENSMUSG00000056328	Myh1	-1.038152353	0.000940522
ENSMUSG00000052516	Robo2	-1.039909169	6.62E-08
ENSMUSG00000087067	Gm11532	-1.0402849	0.028252215
ENSMUSG00000035783	Acta2	-1.044822314	1.57E-180
ENSMUSG00000015880	Ncapg	-1.04519055	5.57E-12
ENSMUSG00000028444	Cntfr	-1.045213967	7.25E-07
ENSMUSG00000020185	E2f7	-1.045637308	1.26E-10
ENSMUSG00000025083	Afap1l2	-1.047180053	3.43E-35
ENSMUSG00000067276	Capn6	-1.047921943	1.93E-11
ENSMUSG00000030772	Dkk3	-1.04795672	1.96E-139
ENSMUSG00000024065	Ehd3	-1.05006486	1.52E-26
ENSMUSG00000038738	Shank1	-1.051379582	0.001218332
ENSMUSG00000056091	St3gal5	-1.052141209	1.53E-63
ENSMUSG00000026443	Lrrn2	-1.053202104	2.68E-08
ENSMUSG00000048126	Col6a3	-1.053240333	5.47E-07
ENSMUSG00000049036	Tmem121	-1.054040624	0.000152269
ENSMUSG00000093405	Gm20684	-1.055979012	0.034193073
ENSMUSG00000070348	Ccnd1	-1.056387327	5.71E-102
ENSMUSG00000082229	Nap1l2	-1.056506808	0.003767994
ENSMUSG00000025475	Adgra1	-1.056804776	8.46E-06
ENSMUSG00000021835	Bmp4	-1.058177546	2.98E-60
ENSMUSG00000038618	Rassf7	-1.05985019	2.74E-06
ENSMUSG00000031778	Cx3cl1	-1.060787825	9.19E-112
ENSMUSG00000044456	Rin3	-1.060950638	1.20E-15
ENSMUSG00000044313	Mab21l3	-1.062743344	1.89E-07
ENSMUSG00000001739	Cldn15	-1.063263627	9.95E-24
ENSMUSG00000046314	Stxbp6	-1.064175919	0.020392235
ENSMUSG00000024206	Rfx2	-1.065431564	0.000361079
ENSMUSG00000012443	Kif11	-1.065577246	1.79E-16
ENSMUSG00000062309	Rpp25	-1.065817463	0.000430616
ENSMUSG00000021391	Cenpp	-1.066419122	0.002192437
ENSMUSG00000027217	Tspan18	-1.066750339	4.99E-24
ENSMUSG00000005951	Shpk	-1.066996383	2.70E-09
ENSMUSG00000040310	Alx4	-1.06907023	0.000775862
ENSMUSG00000057880	Abat	-1.069921295	2.97E-39
ENSMUSG00000032202	Rab27a	-1.071469	0.003801671
ENSMUSG00000047986	Palm3	-1.072856847	0.044062771
ENSMUSG00000038732	Mboat1	-1.074041379	0.000906593
ENSMUSG00000018566	Slc2a4	-1.074823625	0.028789074
ENSMUSG00000026873	Phf19	-1.07507361	1.74E-07
ENSMUSG00000071537	Klrg2	-1.075394138	8.24E-09
ENSMUSG00000035104	Eva1a	-1.077515715	0.002817611
ENSMUSG00000026594	Ralgps2	-1.078504003	3.91E-21
ENSMUSG00000035365	Parpbp	-1.083150019	6.56E-09
ENSMUSG00000029695	Aass	-1.084938352	8.56E-06
ENSMUSG00000022353	Mtss1	-1.085404128	5.99E-11
ENSMUSG00000046807	Lrrc75b	-1.086437899	2.27E-22
ENSMUSG00000115232	Gm49378	-1.087279751	0.00025655
ENSMUSG00000028175	Depdc1a	-1.087776623	3.27E-08
ENSMUSG00000068748	Ptprz1	-1.09210479	2.99E-33
ENSMUSG00000042428	Mgat3	-1.092931037	1.55E-16
ENSMUSG00000024827	Gldc	-1.094356732	2.25E-19
ENSMUSG00000031262	Cenpi	-1.095574449	4.83E-12
ENSMUSG00000039187	Fanci	-1.096047679	5.52E-06
ENSMUSG00000032783	Troap	-1.096360173	5.82E-11
ENSMUSG00000063296	Tmem117	-1.096883241	0.012615566
ENSMUSG00000025395	Prim1	-1.099243639	2.01E-08
ENSMUSG00000079465	Col4a3	-1.09942281	1.44E-32
ENSMUSG00000020897	Aurkb	-1.100192618	9.05E-12
ENSMUSG00000057074	Ces1g	-1.100684735	0.011678783
ENSMUSG00000053101	Gpr141	-1.100793436	0.023510506
ENSMUSG00000093593	Gm20683	-1.103344245	5.10E-06
ENSMUSG00000008090	Fgfrl1	-1.103864924	1.98E-81
ENSMUSG00000045725	Prr15	-1.108217051	0.008078795

ENSMUSG00000037628	Cdkn3	-1.10868585	1.70E-07
ENSMUSG00000000567	Sox9	-1.10887277	2.25E-47
ENSMUSG00000045539	Sprr3	-1.109055366	4.40E-06
ENSMUSG00000027699	Ect2	-1.109649494	1.13E-17
ENSMUSG00000004085	Map3k20	-1.110347457	2.50E-52
ENSMUSG00000034818	Celf5	-1.112772862	1.42E-16
ENSMUSG00000026051	Ecrg4	-1.115431655	4.48E-07
ENSMUSG00000034773	Hrob	-1.116075853	7.10E-09
ENSMUSG00000098318	Lockd	-1.116800566	0.000357306
ENSMUSG00000041498	Kif14	-1.117158176	2.86E-05
ENSMUSG00000026955	Sapcd2	-1.118333981	0.000564136
ENSMUSG00000027896	Slc16a4	-1.118511765	2.59E-09
ENSMUSG00000020493	Prr11	-1.119033033	1.70E-25
ENSMUSG00000074577	Ripor3	-1.119737123	2.14E-11
ENSMUSG00000048922	Cdca2	-1.122945977	8.52E-14
ENSMUSG00000032135	Mcam	-1.127178242	1.42E-40
ENSMUSG00000102692	Dchs2	-1.1279284	1.48E-10
ENSMUSG00000037313	Tacc3	-1.128113486	3.21E-51
ENSMUSG00000032010	Usp2	-1.130366564	6.08E-08
ENSMUSG00000037664	Cdkn1c	-1.131751467	0.000176648
ENSMUSG00000032221	Mns1	-1.132098345	3.49E-10
ENSMUSG00000028445	Enho	-1.132918139	4.28E-11
ENSMUSG00000090122	Kcne1l	-1.133607992	3.72E-06
ENSMUSG00000024395	Lims2	-1.134381674	4.36E-43
ENSMUSG00000027654	Fam83d	-1.139932797	4.15E-25
ENSMUSG00000020808	Pimreg	-1.141978362	6.51E-20
ENSMUSG00000020340	Cyfip2	-1.14364101	4.36E-05
ENSMUSG00000026605	Cenpf	-1.145161545	8.68E-18
ENSMUSG00000023505	Cdca3	-1.145477004	6.60E-39
ENSMUSG00000054493	Gm9947	-1.147551139	0.031994885
ENSMUSG00000020914	Top2a	-1.150257247	6.10E-57
ENSMUSG00000027379	Bub1	-1.152808122	1.05E-16
ENSMUSG00000072437	Nanos1	-1.156274911	3.67E-10
ENSMUSG00000045733	Sprn	-1.15785266	0.027581492
ENSMUSG00000078640	Gm11627	-1.158768673	0.004877762
ENSMUSG00000003283	Hck	-1.158821103	7.70E-19
ENSMUSG00000054196	Cthrc1	-1.15883691	1.44E-83
ENSMUSG00000027115	Kif18a	-1.161776554	1.20E-08
ENSMUSG00000055805	Fmnl1	-1.164145165	4.36E-06
ENSMUSG00000024301	Kifc5b	-1.164448106	6.81E-11
ENSMUSG00000071042	Rasgrp3	-1.167772454	1.06E-45
ENSMUSG00000075334	Rprm	-1.168504815	0.00033686
ENSMUSG00000071984	Fndc1	-1.170308071	2.44E-118
ENSMUSG00000035916	Ptprq	-1.171465976	1.72E-07
ENSMUSG00000041052	Slc7a13	-1.172225146	0.003902297
ENSMUSG00000029516	Cit	-1.173980609	1.33E-16
ENSMUSG00000045903	Npas4	-1.174084774	0.001308985
ENSMUSG00000038917	3930402G23Rik	-1.174458453	0.016156742
ENSMUSG00000034881	Tbxa2r	-1.176174285	1.16E-28
ENSMUSG00000035578	Iqcg	-1.176630199	0.000935411
ENSMUSG00000001507	Itga3	-1.176928324	1.24E-44
ENSMUSG00000024989	Cep55	-1.177050634	2.02E-14
ENSMUSG00000031629	Cenpu	-1.179128396	4.02E-05
ENSMUSG00000050541	Adra1b	-1.179634674	0.000635026
ENSMUSG00000028464	Tpm2	-1.180622401	9.02E-121
ENSMUSG00000029120	Ppp2r2c	-1.181547914	0.004695274
ENSMUSG00000005611	Mrvi1	-1.181929304	1.06E-15
ENSMUSG00000031137	Fgf13	-1.183227312	1.02E-06
ENSMUSG00000029414	Kntc1	-1.183229087	2.60E-16
ENSMUSG00000028782	Adgrb2	-1.18338252	0.000450074
ENSMUSG00000048794	Cfap100	-1.184216326	0.005953891
ENSMUSG00000050271	Prag1	-1.18463542	1.05E-88
ENSMUSG00000000031	H19	-1.18849473	5.92E-08
ENSMUSG00000014077	Chp1	-1.188925193	1.89E-111
ENSMUSG00000041431	Ccnb1	-1.189505141	5.07E-39
ENSMUSG00000007122	Casq1	-1.190900993	0.003574031
ENSMUSG00000002055	Spag5	-1.194537911	5.91E-20
ENSMUSG00000108126	Gm43909	-1.198865211	0.031101982
ENSMUSG00000031825	Crispld2	-1.200584277	1.49E-70
ENSMUSG00000027306	Nusap1	-1.203018301	3.69E-27
ENSMUSG00000031661	Nkd1	-1.207283147	6.95E-08
ENSMUSG00000097908	4933404O12Rik	-1.208268117	7.40E-07
ENSMUSG00000004633	Chn2	-1.209663601	7.14E-09
ENSMUSG00000098090	2700099C18Rik	-1.210876674	1.09E-05
ENSMUSG00000051379	Flrt3	-1.211212598	2.15E-16
ENSMUSG00000027469	Tpx2	-1.21532047	5.72E-63
ENSMUSG00000024525	Impa2	-1.218790291	5.50E-07

ENSMUSG00000021200	Asb2	-1.220545615	0.000478242
ENSMUSG00000039628	Hs3st6	-1.222553683	0.021220485
ENSMUSG00000020330	Hmmr	-1.226117747	6.64E-51
ENSMUSG00000001403	Ube2c	-1.231232064	6.67E-77
ENSMUSG00000015452	Ager	-1.231948546	0.007390267
ENSMUSG00000027875	Hmgcs2	-1.233055558	0.01103915
ENSMUSG00000068859	Sp9	-1.234646206	9.24E-05
ENSMUSG00000028678	Kif2c	-1.235906738	3.65E-21
ENSMUSG00000039193	Nlrc4	-1.238860503	0.048118997
ENSMUSG00000031780	Ccl17	-1.246032338	2.20E-40
ENSMUSG00000079588	Tmem182	-1.247266059	0.025115571
ENSMUSG00000086006	Gm13293	-1.248553411	3.51E-05
ENSMUSG00000035403	Crb2	-1.248933755	2.37E-07
ENSMUSG00000040086	Tnni3k	-1.249874434	1.37E-06
ENSMUSG00000029859	Epha1	-1.252016809	0.009696669
ENSMUSG00000051331	Cacna1c	-1.252695353	1.04E-30
ENSMUSG00000053646	Plxnb1	-1.254718152	3.41E-06
ENSMUSG00000037166	Ppp1r14a	-1.259436704	1.29E-25
ENSMUSG00000044522	A730020M07Rik	-1.261359992	2.25E-05
ENSMUSG00000025789	St8sia2	-1.262942439	0.00034264
ENSMUSG00000116597	Gm536	-1.26901721	0.019473051
ENSMUSG00000001988	Npas1	-1.269665255	0.028280884
ENSMUSG00000009246	Trpm5	-1.270144556	0.000675651
ENSMUSG00000047638	Nr1h4	-1.274302797	7.74E-14
ENSMUSG00000042672	Dcst1	-1.275327896	0.036263134
ENSMUSG00000044550	Tceal3	-1.276632299	0.00067109
ENSMUSG00000029177	Cenpa	-1.278387817	3.99E-34
ENSMUSG00000067215	Usp51	-1.281922311	0.021081425
ENSMUSG00000027318	Adam33	-1.284143231	0.007629041
ENSMUSG00000023015	Racgap1	-1.284896126	1.24E-40
ENSMUSG00000050014	Apol10b	-1.28861034	3.42E-08
ENSMUSG00000048327	Ckap2l	-1.288725545	4.03E-31
ENSMUSG00000026023	Cdk15	-1.295797802	0.008974741
ENSMUSG00000051378	Kif18b	-1.301156513	4.76E-33
ENSMUSG00000049721	Gal3st1	-1.309410184	6.00E-23
ENSMUSG00000051537	Gm5124	-1.311281659	0.02651938
ENSMUSG00000026610	Esrrg	-1.311557842	0.006701589
ENSMUSG00000045328	Cenpe	-1.313241419	1.74E-40
ENSMUSG00000030468	Siglecg	-1.314751742	1.68E-51
ENSMUSG00000032500	Dclk3	-1.321900817	8.43E-18
ENSMUSG00000035095	Fam167a	-1.325023754	0.000948044
ENSMUSG00000032291	Crabp1	-1.327366777	8.13E-09
ENSMUSG00000023036	Pcdhgc4	-1.328871344	0.005708475
ENSMUSG00000041423	Paqr6	-1.330945791	2.10E-06
ENSMUSG00000092627	D130058E05Rik	-1.331305836	0.036094789
ENSMUSG00000048636	A730049H05Rik	-1.336217027	0.000100341
ENSMUSG00000020847	Rph3al	-1.337588007	0.001110704
ENSMUSG00000022240	Ctnnd2	-1.337624484	2.00E-14
ENSMUSG00000032254	Kif23	-1.348395901	2.54E-24
ENSMUSG00000016995	Matn4	-1.349971766	3.08E-05
ENSMUSG00000029576	Radil	-1.353975184	1.62E-09
ENSMUSG00000106707	Gm43748	-1.354205832	0.009966001
ENSMUSG00000037169	Mycn	-1.356431355	4.70E-05
ENSMUSG00000041064	Pif1	-1.358972429	2.17E-07
ENSMUSG00000051359	Ncald	-1.359969945	2.40E-16
ENSMUSG00000047842	Diras2	-1.360033194	0.013816479
ENSMUSG00000037336	Mfsd2b	-1.360850657	5.44E-05
ENSMUSG00000048782	Insc	-1.361187296	4.16E-13
ENSMUSG00000024176	Sox8	-1.36460222	3.29E-06
ENSMUSG00000037337	Map4k1	-1.370280283	0.002286739
ENSMUSG00000048096	Lmod1	-1.371450661	3.02E-32
ENSMUSG00000030600	Lrfn1	-1.374853189	0.00017035
ENSMUSG00000026683	Nuf2	-1.38298016	1.77E-25
ENSMUSG00000034023	Fancd2	-1.383333866	9.95E-09
ENSMUSG00000003469	Phyhip	-1.38343282	1.58E-05
ENSMUSG00000034324	Tmem132c	-1.385333374	0.012222227
ENSMUSG00000074923	Pak6	-1.385534302	0.022804217
ENSMUSG00000067818	Myl9	-1.386700451	6.35E-238
ENSMUSG00000042109	Csdc2	-1.387566345	2.47E-89
ENSMUSG00000031351	Zfp185	-1.388572956	2.12E-07
ENSMUSG00000056531	Ccdc18	-1.389527698	0.000356514
ENSMUSG00000045102	Poln	-1.391247871	0.021611363
ENSMUSG00000095332	Gm9821	-1.393978366	0.007603928
ENSMUSG00000026308	Klhl30	-1.394838407	3.66E-45
ENSMUSG00000032218	Ccnb2	-1.395163877	1.75E-40
ENSMUSG00000021379	Id4	-1.396355699	3.75E-05
ENSMUSG00000056174	Col8a2	-1.396504069	1.83E-32

ENSMUSG00000028287	1700009N14Rik	-1.396556926	0.018443846
ENSMUSG00000022899	Slc15a2	-1.39717229	0.023283812
ENSMUSG00000103932	Gm36963	-1.39825695	0.001043398
ENSMUSG00000038943	Prc1	-1.399175853	5.99E-53
ENSMUSG00000034311	Kif4	-1.402756826	3.21E-30
ENSMUSG00000053007	Creb5	-1.403482271	3.03E-15
ENSMUSG00000027331	Knstrn	-1.406494381	6.49E-29
ENSMUSG00000082738	Gm15422	-1.41676638	0.038486707
ENSMUSG00000009394	Syn2	-1.421823503	2.71E-24
ENSMUSG00000035775	Krt20	-1.424461542	0.000807782
ENSMUSG00000070867	Trabd2b	-1.433146146	1.12E-36
ENSMUSG00000033952	Aspm	-1.433507084	8.54E-17
ENSMUSG00000006398	Cdc20	-1.434804306	1.79E-38
ENSMUSG00000044201	Cdc25c	-1.436337537	4.04E-11
ENSMUSG00000062151	Unc13c	-1.448179428	0.029605082
ENSMUSG00000019789	Hey2	-1.452870834	9.22E-06
ENSMUSG00000037379	Spon2	-1.459132128	9.12E-39
ENSMUSG00000026564	Dusp27	-1.459629623	2.34E-10
ENSMUSG00000025930	Msc	-1.46111564	1.02E-08
ENSMUSG00000021057	Akap5	-1.465264255	1.31E-24
ENSMUSG00000057719	Sh3rf2	-1.468910788	0.045525684
ENSMUSG00000029570	Lfng	-1.473210268	0.000154589
ENSMUSG00000097495	Gm26651	-1.479561328	0.042679486
ENSMUSG00000018849	Wwc1	-1.481398678	3.00E-22
ENSMUSG00000024910	Ctsw	-1.485821515	1.34E-06
ENSMUSG00000030867	Plk1	-1.486197546	1.66E-70
ENSMUSG00000049092	Gpr137c	-1.488512013	0.018329281
ENSMUSG00000015222	Map2	-1.48894715	3.82E-62
ENSMUSG00000021798	Ldb3	-1.490284883	5.31E-09
ENSMUSG00000035615	Frmpd1	-1.493814879	2.68E-14
ENSMUSG00000031553	Adam3	-1.502436686	0.026726942
ENSMUSG00000037544	Dlgap5	-1.505302968	1.21E-40
ENSMUSG00000013367	Iglon5	-1.506276403	8.95E-12
ENSMUSG00000037736	Limch1	-1.506424169	2.31E-51
ENSMUSG00000109674	Gm45470	-1.513426797	0.000110915
ENSMUSG00000047146	Tet1	-1.51835802	0.000531779
ENSMUSG00000000244	Tspan32	-1.521190262	0.000456982
ENSMUSG00000036040	Adamtsl2	-1.525197667	2.27E-164
ENSMUSG00000030731	Syt3	-1.529282156	0.016482147
ENSMUSG00000021751	Acox2	-1.530725244	0.005527951
ENSMUSG00000115388	Eppk1	-1.53425814	0.038957241
ENSMUSG00000021613	Hapln1	-1.53789946	0.00049305
ENSMUSG00000086390	1810019D21Rik	-1.538523312	0.001832737
ENSMUSG00000040424	Hipk4	-1.540322857	0.046877635
ENSMUSG00000038486	Sv2a	-1.557832882	0.008205532
ENSMUSG00000038354	Ankrd35	-1.566886155	0.009462798
ENSMUSG00000021087	Rtn1	-1.569204116	4.79E-66
ENSMUSG00000020583	Matn3	-1.575005023	9.02E-11
ENSMUSG00000017446	C1qtnf1	-1.582404158	1.90E-24
ENSMUSG00000075408	Smim41	-1.584337693	0.000667077
ENSMUSG00000013611	Snx31	-1.585179822	0.014576892
ENSMUSG00000025500	Lmntd2	-1.592760172	0.048136397
ENSMUSG00000020431	Adcy1	-1.593015452	7.74E-09
ENSMUSG00000078234	Klhdc7a	-1.594901717	8.69E-28
ENSMUSG00000029861	Fam131b	-1.59616309	0.001070906
ENSMUSG00000039883	Lrrc17	-1.600795213	1.93E-06
ENSMUSG00000030737	Slco2b1	-1.601854811	0.032215714
ENSMUSG00000021572	Cep72	-1.604923745	6.43E-05
ENSMUSG00000023039	Krt7	-1.612245197	1.47E-15
ENSMUSG00000079355	Ackr4	-1.614640776	1.16E-12
ENSMUSG00000071531	Gprin2	-1.617215374	0.022292273
ENSMUSG00000072809	9330160F10Rik	-1.620186412	0.001078318
ENSMUSG00000032502	Stac	-1.620944281	0.00485929
ENSMUSG00000047013	Fbxo41	-1.622878193	0.022607402
ENSMUSG00000072720	Myo18b	-1.623058921	2.64E-10
ENSMUSG00000061048	Cdh3	-1.63110896	6.33E-07
ENSMUSG00000100980	Gm29100	-1.633337161	0.006406637
ENSMUSG00000025610	Map3k7cl	-1.634038189	4.76E-45
ENSMUSG00000085008	Dbhos	-1.635627925	0.000576405
ENSMUSG00000024210	Ip6k3	-1.640036582	7.29E-05
ENSMUSG00000007097	Atp1a2	-1.644598248	8.25E-30
ENSMUSG00000001663	Gstt1	-1.650233296	6.29E-40
ENSMUSG00000040016	Ptger3	-1.65627603	0.00855928
ENSMUSG00000036766	Dner	-1.663608887	9.52E-14
ENSMUSG00000051435	Fhad1	-1.669963019	0.035860312
ENSMUSG00000032013	Trim29	-1.673750767	0.015723924
ENSMUSG00000025932	Eya1	-1.675161119	0.000291528

ENSMUSG00000063430	Wscd2	-1.680003329	8.92E-10
ENSMUSG00000102331	Gm19938	-1.688164361	0.003603869
ENSMUSG00000000889	Dbh	-1.693749587	5.39E-06
ENSMUSG00000040666	Sh3bgr	-1.694386138	3.38E-07
ENSMUSG00000045532	C1ql1	-1.705951462	0.017866346
ENSMUSG00000034353	Ramp1	-1.721683361	0.000720753
ENSMUSG00000024211	Grm8	-1.721946452	0.028453047
ENSMUSG00000042115	Klhdc8a	-1.721985828	1.08E-11
ENSMUSG00000052854	Nrk	-1.73479066	6.55E-09
ENSMUSG00000051041	Olfml1	-1.738332349	1.84E-16
ENSMUSG00000070803	Cited4	-1.742874504	0.009468878
ENSMUSG00000063063	Ctnna2	-1.743114667	0.005106742
ENSMUSG00000097111	Peak1os	-1.746469098	0.001896469
ENSMUSG00000054675	Tmem119	-1.755624739	1.44E-138
ENSMUSG00000057606	Colq	-1.759408675	0.000235338
ENSMUSG00000047443	Erfe	-1.78143018	5.55E-74
ENSMUSG00000071604	Fam189a2	-1.783679167	9.24E-27
ENSMUSG00000019326	Aoc3	-1.78479602	1.07E-14
ENSMUSG00000002324	Rec8	-1.789830673	6.35E-05
ENSMUSG00000031098	Syt8	-1.800892836	1.63E-05
ENSMUSG00000023328	Ache	-1.804707662	0.00369921
ENSMUSG00000109941	Exosc6	-1.805129722	0.042254964
ENSMUSG00000111361	Gm47445	-1.806713435	0.009210248
ENSMUSG00000051517	Arhgef39	-1.810466513	7.34E-10
ENSMUSG00000068522	Aard	-1.814865052	0.000200496
ENSMUSG00000031323	Dmrtc1a	-1.82959583	0.040049336
ENSMUSG00000023391	Dlx2	-1.845537209	0.002013718
ENSMUSG00000005357	Slc1a6	-1.849265085	1.43E-83
ENSMUSG00000002578	Ikzf4	-1.850951976	0.002317559
ENSMUSG00000039097	Rln1	-1.852384258	0.0417262
ENSMUSG00000034687	Fras1	-1.860072009	0.000290048
ENSMUSG00000018451	6330403K07Rik	-1.865088006	0.001315581
ENSMUSG00000037962	Rflna	-1.868864922	0.000868641
ENSMUSG00000020866	Cacna1g	-1.890064945	1.75E-17
ENSMUSG00000033544	Angptl1	-1.893756735	3.92E-07
ENSMUSG00000113909	Gm36377	-1.894746272	8.90E-32
ENSMUSG00000044461	Shisa2	-1.901639228	0.000671233
ENSMUSG00000001763	Tspan33	-1.91209543	0.033336037
ENSMUSG00000117029	Gm36201	-1.928411367	0.0025073
ENSMUSG00000086587	Gm11837	-1.929981609	0.029684463
ENSMUSG00000019905	Gprc6a	-1.941741532	0.029614263
ENSMUSG00000086233	Gm11816	-1.952960179	0.008149997
ENSMUSG00000027356	Fermt1	-1.955947016	0.001050838
ENSMUSG00000058914	C1qtnf3	-1.978638407	5.57E-17
ENSMUSG00000001349	Cnn1	-1.99476412	1.79E-43
ENSMUSG00000053024	Cntn2	-1.995426868	0.048498178
ENSMUSG00000028626	Col9a2	-2.003494261	0.001690368
ENSMUSG00000053263	Gm12592	-2.003936015	0.008584454
ENSMUSG00000033998	Kcnk1	-2.020155511	2.17E-08
ENSMUSG00000030607	Acan	-2.037773231	5.49E-12
ENSMUSG00000028558	Calr4	-2.072501165	3.13E-18
ENSMUSG00000079173	Zan	-2.093037229	0.048764237
ENSMUSG00000025150	Cbr2	-2.144502643	8.93E-14
ENSMUSG00000048402	Gli2	-2.145748351	9.36E-23
ENSMUSG00000036896	C1qc	-2.154108829	0.015776629
ENSMUSG00000113346	Eprn	-2.160238598	1.01E-05
ENSMUSG00000036905	C1qb	-2.162930945	0.017252384
ENSMUSG00000067561	Dmrtc1c2	-2.176761202	0.002631467
ENSMUSG00000039013	Siglecf	-2.248107388	0.009692933
ENSMUSG00000049093	Il23r	-2.270380796	0.020269104
ENSMUSG00000027335	Adra1d	-2.28896098	8.04E-08
ENSMUSG00000067562	Dmrtc1c1	-2.306166146	0.001271858
ENSMUSG00000029211	Gabra4	-2.325919869	0.026891938
ENSMUSG00000055027	Smyd1	-2.347021378	3.21E-07
ENSMUSG00000044400	Sowahd	-2.400613799	0.000822899
ENSMUSG00000049538	Adamts16	-2.406289451	0.005297311
ENSMUSG00000057751	Megf6	-2.428470657	1.18E-64
ENSMUSG00000055003	Lrtm2	-2.459644777	0.000264045
ENSMUSG00000090394	4930523C07Rik	-2.660193677	1.27E-99
ENSMUSG00000020542	Myocd	-2.676051086	0.00873126
ENSMUSG00000048485	Zbtb8b	-2.682561757	6.46E-07
ENSMUSG00000031364	Grpr	-2.705568079	0.014397002
ENSMUSG00000023999	Kif6	-2.762185954	0.000577893
ENSMUSG00000033597	Caskin1	-2.843091799	0.010178579
ENSMUSG00000062393	Dgkk	-2.853715838	1.42E-09
ENSMUSG00000015599	Ttbk1	-3.373383855	0.000622321
ENSMUSG00000094365	Gm21982	-3.783727365	0.003644567

ENSMUSG00000104793	Gm43756	-7.913338825	1.34E-06
ENSMUSG00000006542	Prkag3	-8.364063771	4.57E-09

Supplemental Table 3. Echocardiography examination of IAMP KO mice and control littermates at different stages

	10 weeks		7 months	
	**Ctrl (N=5)**	**KO (N=4)**	**Ctrl (N=3)**	**KO (N=3)**
LVAW;d (mm)	0.735 ± 0.02	0.749 ± 0.026	0.832 ± 0.026	0.802 ± 0.026
LVAW;s (mm)	1.406 ± 0.064	1.475 ± 0.026	1.558 ± 0.094	1.497 ± 0.198
LVID;d (mm)	3.376 ± 0.149	3.357 ± 0.098	3.569 ± 0.367	3.448 ± 0.045
LVID;s (mm)	1.651 ± 0.076	1.645 ± 0.043	1.754 ± 0.159	1.694 ± 0.094
LVPW;d (mm)	0.726 ± 0.032	0.749 ± 0.026	0.832 ± 0.026	0.817 ± 0.045
LVPW;s (mm)	1.425 ± 0.076	1.395 ± 0.043	1.633 ± 0.045	1.543 ± 0.079
EF (%)	83.194 ± 2.264	83.163 ± 2.161	82.906 ± 0.363	82.955 ± 2.761
FS (%)	51.026 ± 2.597	50.963 ± 2.503	50.804 ± 0.698	50.854 ± 3.213
LV Mass (mg)	78.824 ± 8.011	80.658 ± 4.264	103.834 ± 20.786	93.867 ± 6.98
LV Mass (Corrected, mg)	63.059 ± 6.409	64.526 ± 3.411	83.067 ± 16.629	75.093 ± 5.584
LV Vol;d (µL)	46.732 ± 5.126	46.06 ± 3.21	53.904 ± 12.96	49.081 ± 1.556
LV Vol;s (µL)	7.808 ± 0.918	7.707 ± 0.523	9.193 ± 2.113	8.343 ± 1.177
Heart Rate (BPM)	669.89 ± 30.168	662.727 ± 32.709	686.696 ± 6.177	659.434 ± 41.775

LVAW;d, LV anterior wall thickness at end-diastole; LVAW;s, LV anterior wall thickness at end-systole; LVID;d, LV internal dimension at end-diastole; LVID;s, LV internal dimension at end-systole; LVPW;d, LV posterior wall thickness at end-diastole; LVPW;s, LV posterior wall thickness at end-systole; EF, ejection fraction; FS, fraction shortening; LV Vol;d, LV volume at end-diastole; LV Vol;s, LV volume at end-systole.

Supplemental Table 4. Echocardiography examination of IAMP KO mice and Ctrl littermates 2 weeks after I/R surgery

	Sham		I/R	
	**Ctrl (N=7)**	**KO (N=7)**	**Ctrl (N=8)**	**KO (N=10)**
LVAW;d (mm)	0.745 ± 0.044	0.745 ± 0.078	0.561 ± 0.108**	0.413 ± 0.143$^{\#}$
LVAW;s (mm)	1.497 ± 0.087	1.426 ± 0.058	1.027 ± 0.215***	0.726 ± 0.196$^{\#\#}$
LVID;d (mm)	3.526 ± 0.163	3.442 ± 0.164	4.458 ± 0.304****	5.336 ± 0.644$^{\#\#}$
LVID;s (mm)	1.756 ± 0.086	1.692 ± 0.043	3.045 ± 0.411****	4.519 ± 0.688$^{\#\#\#\#}$
LVPW;d (mm)	0.758 ± 0.022	0.765 ± 0.049	0.76 ± 0.137	0.594 ± 0.169$^{\#}$
LVPW;s (mm)	1.504 ± 0.041	1.458 ± 0.129	1.31 ± 0.247	0.907 ± 0.243$^{\#\#}$
EF (%)	82.347 ± 1.244	82.931 ± 1.822	59.794 ± 8.244****	32.301 ± 9.043$^{\#\#\#\#}$
FS (%)	50.175 ± 1.384	50.771 ± 2.18	31.924 ± 5.735****	15.547 ± 4.69$^{\#\#\#\#}$
LV Mass (mg)	87.973 ± 7.893	85.449 ± 12.858	111.016 ± 22.585*	106.821 ± 19.569
LV Mass (Corrected, mg)	70.378 ± 6.314	68.359 ± 10.286	88.813 ± 18.068*	85.457 ± 15.655
LV Vol;d (µL)	51.929 ± 5.798	49.001 ± 5.618	90.954 ± 13.769****	139.988 ± 41.177$^{\#\#}$
LV Vol;s (µL)	9.162 ± 1.105	8.291 ± 0.541	37.249 ± 10.954****	96.218 ± 34.371$^{\#\#\#}$
Heart Rate (BPM)	633.652 ± 27.676	638.624 ± 31.463	632.723 ± 23.989	638.37 ± 47.831

Ctrl_Sham vs. Ctrl_I/R: *: $P < 0.05$, **: $P < 0.01$, ***: $P < 0.001$, ****: $P < 0.0001$; Ctrl_I/R vs. KO_I/R: $^{\#}$: $P < 0.05$, $^{\#\#}$: $P < 0.01$, $^{\#\#\#}$: $P < 0.001$, $^{\#\#\#\#}$: $P < 0.0001$. LVAW;d, LV anterior wall thickness at end-diastole; LVAW;s, LV anterior wall thickness at end-systole; LVID;d, LV internal dimension at end-diastole; LVID;s, LV internal dimension at end-systole; LVPW;d, LV posterior wall thickness at end-diastole; LVPW;s, LV posterior wall thickness at end-systole; EF, ejection fraction; FS, fraction shortening; LV Vol;d, LV volume at end-diastole; LV Vol;s, LV volume at end-systole. Statistical significance indicated in this table was determined using a two-tailed unpaired t-test to assess differences between two groups

Supplemental Table 5. Differentially expressed genes in IL-17-treated IAMP knockdown cardiac fibroblasts

siR-1+IL17 vs siNC+IL17

ensembl_gene_id	gene_name	log2FoldChange	padj
ENSMUSG00000112084	Gm10773	7.088333931	5.70478E-05
ENSMUSG00000109045	Gm45084	6.980026224	9.65741E-05
ENSMUSG00000085884	Gm15342	6.836466565	0.000100416
ENSMUSG00000102419	Gm36940	6.788117673	0.000251884
ENSMUSG00000104292	Gm38042	6.760202877	0.000246154
ENSMUSG00000092390	Gm20541	6.757309634	0.000217039
ENSMUSG00000047108	Dnajb7	6.64806894	0.000340287
ENSMUSG00000115025	Gm4240	6.592176852	0.000350734
ENSMUSG00000098243	Gm4258	6.56223135	0.00048096
ENSMUSG00000022523	Fgf12	6.500411129	0.000642263
ENSMUSG00000108543	Gm44735	6.468873015	0.000885585
ENSMUSG00000043670	Diras1	6.466099916	0.001149031
ENSMUSG00000089997	1810020O05Rik	6.370015081	0.001046736
ENSMUSG00000112146	Gm46210	6.335514117	0.001205963
ENSMUSG00000025480	Syce1	6.332720151	0.001529643
ENSMUSG00000081801	Dnmt3l-ps1	6.265149088	0.007912898
ENSMUSG00000103506	Gm38376	6.264530366	0.001919002
ENSMUSG00000082315	Gm16523	6.262157817	0.00804133
ENSMUSG00000085055	Gm15958	6.261876322	0.001748134
ENSMUSG00000108359	C430039J01Rik	6.232128758	0.011614621
ENSMUSG00000037318	Traf3ip3	6.187412567	0.004734701
ENSMUSG00000108084	Gm7932	6.186369858	0.003455482
ENSMUSG00000086324	Gm15564	6.185310661	0.002711458
ENSMUSG00000082794	Gm6806	6.176326411	1.39156E-06
ENSMUSG00000108460	Gm39041	6.149575981	0.011213243
ENSMUSG00000059562	Ccdc154	6.148922635	0.002821865
ENSMUSG00000103220	Gm37728	6.148362453	0.011150746
ENSMUSG00000105701	Gm42587	6.148266458	0.002729765
ENSMUSG00000085445	Gm16348	6.111720873	0.012797912
ENSMUSG00000004948	Zp3	6.106743698	0.003205298
ENSMUSG00000010142	Tnfrsf13b	6.06610149	0.004276281
ENSMUSG00000107162	Gm18593	6.065235389	0.00381137
ENSMUSG00000025658	Cnksr2	6.064829221	0.003830173
ENSMUSG00000103937	Gm37186	6.022609451	0.015918023
ENSMUSG00000023159	Psg29	6.019912666	0.005094424
ENSMUSG00000103739	Gm37653	5.984818159	0.02465619
ENSMUSG00000111301	Gm30671	5.982983601	0.020080861
ENSMUSG00000050844	1700020N01Rik	5.980253353	0.01817581
ENSMUSG00000022824	Muc13	5.978290381	0.008131076
ENSMUSG00000022901	Cd86	5.978037259	0.005397962
ENSMUSG00000054568	Usp17la	5.97613065	0.044972342
ENSMUSG00000084769	Gm12061	5.976112539	0.005873104
ENSMUSG00000027073	Prg2	5.974874796	0.006864314
ENSMUSG00000103657	Gm37204	5.934278167	0.00706619
ENSMUSG00000114036	Gm48228	5.932396385	0.020318959
ENSMUSG00000114501	Gm48582	5.889687706	0.02520513
ENSMUSG00000104938	Gm42840	5.88836168	0.010082791
ENSMUSG00000022763	Aifm3	5.886293783	0.023044909
ENSMUSG00000032099	Pate4	5.886293783	0.023044909
ENSMUSG00000106720	Gm43795	5.886293783	0.023044909
ENSMUSG00000106110	Gm43571	5.885822661	0.00813361
ENSMUSG00000114472	Gm38397	5.885809272	0.023004167
ENSMUSG00000107676	Gm44178	5.884871488	0.008052754
ENSMUSG00000112388	AI463170	5.884379221	0.009729383
ENSMUSG00000099543	Gm29322	5.884066959	0.009034296
ENSMUSG00000024798	Htr7	5.837037252	0.009476215
ENSMUSG00000116450	Gm49534	5.832241036	0.026688578
ENSMUSG00000112317	Gm47580	5.789939865	0.031860316
ENSMUSG00000103232	Gm37160	5.789419989	0.031203622
ENSMUSG00000108067	Gm43953	5.787678107	0.012292935
ENSMUSG00000041479	Syt15	5.784229691	0.030020343
ENSMUSG00000118282	Gm4840	5.783455482	0.011324579
ENSMUSG00000037446	Tulp1	5.737754457	0.034526467
ENSMUSG00000103747	Gm38236	5.733609806	0.013333996
ENSMUSG00000103199	Gm37648	5.733279573	0.012990842
ENSMUSG00000107534	Gm43920	5.72938454	0.015788321
ENSMUSG00000034472	Rasd2	5.728823381	0.034041445
ENSMUSG00000116766	Gm49702	5.677836243	0.015828137
ENSMUSG00000105478	Gm43285	5.676939797	0.018185346
ENSMUSG00000112058	Gm47593	5.67607426	0.039693129
ENSMUSG00000086047	9530046B11Rik	5.675981007	0.016563023
ENSMUSG00000087247	Alkal1	5.675045413	0.018269669
ENSMUSG00000108784	4930517G19Rik	5.674867217	0.039342521
ENSMUSG00000031837	Necab2	5.672546714	0.042329372

siR-2+IL17 vs siNC+IL17

ensembl_gene_id	gene_name	log2FoldChange	padj
ENSMUSG00000090527	Aadacl2fm2	7.693993543	3.14773E-07
ENSMUSG00000047222	Rnase2a	7.679895724	6.45466E-07
ENSMUSG00000025480	Syce1	7.452102029	2.07599E-06
ENSMUSG00000106632	Gm33466	7.420121966	2.77132E-06
ENSMUSG00000030483	Cyp2b10	7.354985581	3.35257E-06
ENSMUSG00000078308	Gm47854	7.042651305	3.03958E-05
ENSMUSG00000046323	Dppa3	6.934832814	3.6827E-05
ENSMUSG00000035131	Brinp3	6.913693323	9.18767E-05
ENSMUSG00000054266	Serpinb9d	6.666518142	0.000217232
ENSMUSG00000082240	Gm14380	6.585500866	0.000262725
ENSMUSG00000101849	Gm28386	6.436438505	0.000541472
ENSMUSG00000118153	Gm50288	6.435714357	0.000554599
ENSMUSG00000044338	Aplnr	6.40444226	0.000848231
ENSMUSG00000033255	Gm5134	6.371693868	0.000722336
ENSMUSG00000010142	Tnfrsf13b	6.337800781	0.001066911
ENSMUSG00000107162	Gm18593	6.305538077	0.001141915
ENSMUSG00000021991	Cacna2d3	6.304660164	0.00124473
ENSMUSG00000108815	Gm49388	6.283305236	0.000458987
ENSMUSG00000054046	Klk13	6.235386489	0.00164293
ENSMUSG00000081189	Hspd1-ps4	6.235181447	0.001807803
ENSMUSG00000110500	Gm32568	6.197641289	0.001590009
ENSMUSG00000085880	Gm15229	6.160311904	0.002210325
ENSMUSG00000086709	Gm16263	6.160187594	0.002077039
ENSMUSG00000022523	Fgf12	6.159824543	0.002229442
ENSMUSG00000027073	Prg2	6.12248811	0.002243308
ENSMUSG00000109853	Gm33045	6.12199783	0.002194379
ENSMUSG00000113387	Gm31508	6.120448952	0.003362673
ENSMUSG00000105701	Gm42587	6.083343847	0.00303122
ENSMUSG00000086047	9530046B11Rik	6.082540686	0.002549852
ENSMUSG00000041248	Kcnj1	6.081603865	0.011730458
ENSMUSG00000052270	Fpr2	6.08059127	0.012818018
ENSMUSG00000083622	Gm15570	6.043511137	0.013257687
ENSMUSG00000117182	Gm18068	6.04299757	0.004413978
ENSMUSG00000108543	Gm44735	6.042701029	0.003642193
ENSMUSG00000047108	Dnajb7	6.001421388	0.006236209
ENSMUSG00000023153	Tmem52	5.999189839	0.004427113
ENSMUSG00000102657	Gm37899	5.958668596	0.027116148
ENSMUSG00000037318	Traf3ip3	5.914314584	0.020429055
ENSMUSG00000087190	D430001F17Rik	5.913493825	0.006128558
ENSMUSG00000106361	Gm35066	5.913355117	0.005947825
ENSMUSG00000106648	Gm43176	5.912545439	0.018961607
ENSMUSG00000019892	Lrriq1	5.911960912	0.018741575
ENSMUSG00000110986	Gm20276	5.911483886	0.006506763
ENSMUSG00000026523	Wdr64	5.86580209	0.021013857
ENSMUSG00000085523	Gm15945	5.865312721	0.008166792
ENSMUSG00000106720	Gm43795	5.820643438	0.028253133
ENSMUSG00000103199	Gm37648	5.820207686	0.008606783
ENSMUSG00000085757	Tslrn1	5.820207686	0.008606783
ENSMUSG00000046240	Hepacam	5.820127681	0.007588796
ENSMUSG00000023964	Calcr	5.820011987	0.007665917
ENSMUSG00000089822	Gm15759	5.819880511	0.007995983
ENSMUSG00000060913	Trim55	5.819476943	0.024118688
ENSMUSG00000115025	Gm4240	5.819033623	0.007302371
ENSMUSG00000032719	Sbspon	5.818569121	0.023742477
ENSMUSG00000020436	Gabrg2	5.818244471	0.008651046
ENSMUSG00000113604	Gm19786	5.770688317	0.008425114
ENSMUSG00000086324	Gm15564	5.77035113	0.008517241
ENSMUSG00000100147	1700047M11Rik	5.770331674	0.027019138
ENSMUSG00000083339	Gm11693	5.770231203	0.008701191
ENSMUSG00000112058	Gm47593	5.770159504	0.026912067
ENSMUSG00000106709	Gm30270	5.770124563	0.010734404
ENSMUSG00000102160	Gm36944	5.769297699	0.02726679
ENSMUSG00000102415	A430110C17Rik	5.721667958	0.032979494
ENSMUSG00000107534	Gm43920	5.720701269	0.033049684
ENSMUSG00000059898	Dsc3	5.720321289	0.033075152
ENSMUSG00000086498	Gm12681	5.719301655	0.01101946
ENSMUSG00000086021	Gm15767	5.719301655	0.01101946
ENSMUSG00000061527	Krt5	5.667650488	0.036427454
ENSMUSG00000042641	Rgsl1	5.667483604	0.03584716
ENSMUSG00000087430	Gm13405	5.666284362	0.014109703
ENSMUSG00000107430	Gm31532	5.614742368	0.04161217
ENSMUSG00000024500	Ppp2r2b	5.614685839	0.039734751
ENSMUSG00000104000	Gm38335	5.613552376	0.014960477
ENSMUSG00000108105	Gm5340	5.613479564	0.01496168

ENSMUSG00000102930	Gm38115	5.622193954	0.026698397
ENSMUSG00000108689	Gm45354	5.620318779	0.019140286
ENSMUSG00000026523	Wdr64	5.565884096	0.049606827
ENSMUSG00000114555	Gm48706	5.564611382	0.02285222
ENSMUSG00000117390	Gm50080	5.564394446	0.026986355
ENSMUSG00000086709	Gm16263	5.564017108	0.022455246
ENSMUSG00000082429	Gm13171	5.564017108	0.022455246
ENSMUSG00000081782	Gm15152	5.563405777	0.024721626
ENSMUSG00000116924	4933401D09Rik	5.500511863	0.027819328
ENSMUSG00000020734	Grin2c	5.499638499	0.027875692
ENSMUSG00000111017	Gm48314	5.439839868	0.033612654
ENSMUSG00000102160	Gm36944	5.438542065	0.031931197
ENSMUSG00000056600	Olfr90	5.437469373	0.031308024
ENSMUSG00000114081	Gm48904	5.43706429	0.032742408
ENSMUSG00000107865	Gm44417	5.405026822	0.006868609
ENSMUSG00000105397	Gm43471	5.373846196	0.044195159
ENSMUSG00000085229	Gm11672	5.372479081	0.039602044
ENSMUSG00000085897	Gm11604	5.370421127	0.04051293
ENSMUSG00000045036	Tmem232	5.369084666	0.038013858
ENSMUSG00000084783	Gm15419	5.368638659	0.038303951
ENSMUSG00000097072	Foxl2os	5.367991692	0.044719395
ENSMUSG00000103001	A930005N03Rik	5.335427663	0.008113403
ENSMUSG00000025946	Pth2r	5.299725144	0.04993872
ENSMUSG00000103591	Gm38365	5.299681192	0.044953899
ENSMUSG00000102594	Gm38381	5.299436147	0.045468775
ENSMUSG00000108741	Gm44553	5.227839581	0.011326648
ENSMUSG00000103373	Gm37238	5.161534189	1.01451E-05
ENSMUSG00000082855	Gm14537	4.874719077	0.029279774
ENSMUSG00000109523	Gdf1	4.869769762	0.041469654
ENSMUSG00000083945	Gm15242	4.777724576	0.005845398
ENSMUSG00000100303	2600014E21Rik	4.574343837	7.29278E-10
ENSMUSG00000081884	Gm11821	4.474099204	0.018100914
ENSMUSG00000027925	Sprr2j-ps	4.285604812	5.22791E-11
ENSMUSG00000102258	Gm38257	4.249588945	0.001578883
ENSMUSG00000099021	Rn7s1	4.22926882	1.95217E-05
ENSMUSG00000085585	Gm12223	4.210602516	0.045932973
ENSMUSG00000099250	Rn7s2	4.148970553	6.31981E-06
ENSMUSG00000113665	Gm47603	4.067905442	0.03473575
ENSMUSG00000020264	Slc36a2	3.990884116	0.001628903
ENSMUSG00000102672	Gm37105	3.990519046	0.023438602
ENSMUSG00000021700	Rab3c	3.860754034	6.22087E-25
ENSMUSG00000106636	Gm43813	3.776432436	0.012469338
ENSMUSG00000116707	Gm49590	3.702933651	0.006241318
ENSMUSG00000107436	Gm44416	3.627220407	0.025764378
ENSMUSG00000028128	F3	3.548504754	0
ENSMUSG00000102662	Gm38377	3.525321016	0.030212429
ENSMUSG00000098387	Pet117	3.520923313	0.043488285
ENSMUSG00000103970	Gm37678	3.516954297	0.029246387
ENSMUSG00000041620	Mmp1b	3.508785479	1.58103E-39
ENSMUSG00000021070	Bdkrb2	3.383676011	6.47425E-25
ENSMUSG00000106682	Gm42648	3.366797074	0.037970023
ENSMUSG00000028354	Fmn2	3.330151644	3.95743E-10
ENSMUSG00000046093	Hpcal4	3.307246261	0.028769173
ENSMUSG00000062737	Prl3d2	3.287757258	0.025764378
ENSMUSG00000046259	Sprr2h	3.274738598	7.51779E-19
ENSMUSG00000055030	Sprr2e	3.253520781	1.06338E-36
ENSMUSG00000038357	Camp	3.202259804	2.21459E-05
ENSMUSG00000064115	Cadm2	3.191231351	0.039602044
ENSMUSG00000087397	Rapgef3os2	3.174880457	0.013497411
ENSMUSG00000103818	Gm38009	3.151583574	0.007276403
ENSMUSG00000052271	Bhlha15	3.106713363	2.90322E-21
ENSMUSG00000097919	Gm27021	3.097077279	0.012976099
ENSMUSG00000027408	Cpxm1	3.01946979	0
ENSMUSG00000006490	Prl8a9	3.016394809	1.66905E-26
ENSMUSG00000091572	Vmn2r3	3.016218563	0.010268697
ENSMUSG00000107497	Gm44957	2.946312237	0.007064598
ENSMUSG00000036381	P2ry14	2.938720167	2.0581E-15
ENSMUSG00000056445	Hoxaas2	2.925539523	0.044413285
ENSMUSG00000026166	Ccl20	2.887748702	0
ENSMUSG00000086134	Gm16159	2.8832313	0.000976518
ENSMUSG00000050069	Grem2	2.87478352	9.13523E-20
ENSMUSG00000015134	Aldh1a3	2.865025473	0
ENSMUSG00000055561	Spink5	2.739629576	0.029664181
ENSMUSG00000112822	A130012E19Rik	2.732256158	0.011240997
ENSMUSG00000036805	Noxa1	2.723254887	0.04447129
ENSMUSG00000020374	Rasgef1c	2.719614637	3.11139E-13
ENSMUSG00000098132	Rassf10	2.704986995	0.000104767

ENSMUSG00000037982	Gm9725	5.61336426	0.014598896
ENSMUSG00000112168	Gm34776	5.613060346	0.016114782
ENSMUSG00000074256	Gm10655	5.61252837	0.018861083
ENSMUSG00000097286	Gm26684	5.6120999	0.015902348
ENSMUSG00000116766	Gm49702	5.55813348	0.046052517
ENSMUSG00000026765	Lypd6b	5.556936327	0.017901204
ENSMUSG00000103506	Gm38376	5.556936327	0.017901204
ENSMUSG00000079008	Gm14124	5.556759622	0.017907506
ENSMUSG00000102365	Gm37045	5.556292786	0.017503303
ENSMUSG00000108060	4921529L05Rik	5.555584558	0.018399678
ENSMUSG00000111308	Gm48710	5.49657901	0.022302786
ENSMUSG00000107865	Gm44417	5.444384991	0.025074163
ENSMUSG00000085546	Gm14252	5.43716682	0.028267168
ENSMUSG00000114036	Gm48228	5.437104576	0.027534225
ENSMUSG00000117350	Gm49975	5.436211534	0.026703448
ENSMUSG00000093453	Vmn1r-ps146	5.373094653	0.035973261
ENSMUSG00000050635	Sprr2f	5.348081324	2.14218E-59
ENSMUSG00000087290	Gm15866	5.305845698	0.038880152
ENSMUSG00000050921	P2ry10	5.305168965	0.035840029
ENSMUSG00000041380	Htr2c	5.304913887	0.035320681
ENSMUSG00000026241	Nppc	5.304470738	0.035892538
ENSMUSG00000102549	Gm38137	5.281171279	4.75732E-09
ENSMUSG00000089371	Mir1938	5.234946689	0.043196748
ENSMUSG00000113370	Gm46348	5.233723591	0.04452654
ENSMUSG00000070465	Gm9696	5.090512987	1.55506E-17
ENSMUSG00000027925	Sprr2j-ps	4.909829613	1.7964E-17
ENSMUSG00000082794	Gm6806	4.798342454	0.00246247
ENSMUSG00000116210	Spn-ps	4.658987845	0.001118196
ENSMUSG00000020676	Ccl11	4.633059711	7.3442E-47
ENSMUSG00000100303	2600014E21Rik	4.568649754	8.17985E-10
ENSMUSG00000028354	Fmn2	4.414884435	2.38819E-29
ENSMUSG00000106915	Gm42655	4.373337697	0.004581966
ENSMUSG00000086962	Gm12248	4.283314877	0.007705631
ENSMUSG00000103373	Gm37238	4.204037578	0.006785396
ENSMUSG00000074449	Gm15319	4.186487928	0.029532936
ENSMUSG00000116755	6430553K19Rik	4.023515377	0.001949445
ENSMUSG00000103822	6030460B20Rik	4.01952842	0.018487204
ENSMUSG00000062345	Serpinb2	4.011332441	0
ENSMUSG00000094125	Gm13698	4.010116107	0.041078296
ENSMUSG00000042845	Wfdc12	3.998986699	0.006129219
ENSMUSG00000097322	A530083I20Rik	3.961007306	0.036566797
ENSMUSG00000068587	Mgam	3.925357678	2.20267E-72
ENSMUSG00000021943	Gdf10	3.909434096	0.005795403
ENSMUSG00000111709	Gm3776	3.84901757	1.89304E-44
ENSMUSG00000055030	Sprr2e	3.832477894	2.57087E-63
ENSMUSG00000046203	Sprr2g	3.823977704	9.10875E-84
ENSMUSG00000031098	Syt8	3.76041703	0.046966811
ENSMUSG00000046093	Hpcal4	3.737312043	0.003277504
ENSMUSG00000084771	A230072E10Rik	3.705486849	0.021486611
ENSMUSG00000109369	Vmn2r-ps88	3.698438546	0.000723703
ENSMUSG00000027869	Hsd3b6	3.656731165	0.000684845
ENSMUSG00000098387	Pet117	3.641230422	0.007344107
ENSMUSG00000050069	Grem2	3.6144412	3.99615E-51
ENSMUSG00000079105	C7	3.582234437	0.022863529
ENSMUSG00000028766	Alpl	3.567721705	7.9914E-205
ENSMUSG00000050830	Vwc2	3.535019857	0.02248207
ENSMUSG00000102258	Gm38257	3.512680967	0.031909918
ENSMUSG00000104348	Gm37691	3.512277243	0.024132127
ENSMUSG00000022018	Rgcc	3.506257579	0.012302046
ENSMUSG00000049537	Tecrl	3.465076611	0.013456919
ENSMUSG00000048834	Vstm2a	3.413291995	8.37507E-20
ENSMUSG00000094092	Gm15921	3.410050959	0.036525622
ENSMUSG00000055561	Spink5	3.409222729	0.000274948
ENSMUSG00000056054	S100a8	3.395648344	5.64042E-06
ENSMUSG00000090326	Dthd1	3.374614165	0.036682189
ENSMUSG00000042212	Sprr2d	3.311567741	0.00368031
ENSMUSG00000074179	Gm10639	3.28553421	0.048997454
ENSMUSG00000110397	Gm45540	3.269200779	0.000880477
ENSMUSG00000086711	Gm15482	3.259543301	0.042999088
ENSMUSG00000105151	Gm43241	3.254520701	0.018999066
ENSMUSG00000074183	Gsta1	3.248319225	2.23956E-58
ENSMUSG00000105102	Gm35507	3.232390445	0.042577135
ENSMUSG00000029379	Cxcl3	3.197642606	0
ENSMUSG00000051022	Hs3st1	3.184665379	2.1533E-126
ENSMUSG00000044176	Spink10	3.154626738	4.93977E-25
ENSMUSG00000090145	Ugt1a6b	3.140636395	1.72051E-29
ENSMUSG00000051361	6030498E09Rik	3.129306782	0.000425938

ENSMUSG00000047497	Adamts12	2.687801483	0	ENSMUSG00000038357	Camp	3.125516879	5.49907E-05
ENSMUSG00000100816	Gm28321	2.67541937	0.000198161	ENSMUSG00000097919	Gm27021	3.108734827	0.017525401
ENSMUSG00000046203	Sprr2g	2.672804755	8.22023E-25	ENSMUSG00000037996	Slc24a2	3.090278205	0.000199807
ENSMUSG00000005413	Hmox1	2.65116216	0	ENSMUSG00000031551	Ido1	3.066477683	0.003306135
ENSMUSG00000117604	Gm33228	2.641825939	3.59772E-51	ENSMUSG00000026185	Igfbp5	3.054592056	1.534E-101
ENSMUSG00000031289	Il13ra2	2.627688777	7.25302E-63	ENSMUSG00000057933	Gsta2	3.049720737	2.13817E-39
ENSMUSG00000032878	Ccdc85a	2.627186976	8.99519E-12	ENSMUSG00000001865	Cpa3	3.011009811	0.001322664
ENSMUSG00000085925	Rtl1	2.62686331	0.024949565	ENSMUSG00000021070	Bdkrb2	3.004368065	2.38345E-16
ENSMUSG00000040026	Saa3	2.599038996	1.88152E-17	ENSMUSG00000045573	Penk	2.951830753	2.23047E-35
ENSMUSG00000108353	Gm45205	2.597593636	0.049009132	ENSMUSG00000085781	Gm15640	2.949418206	2.62293E-71
ENSMUSG00000040896	Kcnd3	2.566599198	2.289E-12	ENSMUSG00000117604	Gm33228	2.933039321	2.60342E-75
ENSMUSG00000107118	Gm42986	2.533396639	0.009171848	ENSMUSG00000103266	Gm36979	2.928352697	0.027318558
ENSMUSG00000037994	Slc9b2	2.527065972	1.23075E-09	ENSMUSG00000044349	Snhg11	2.9195288	1.0665E-136
ENSMUSG00000034391	Fbxo15	2.472046881	0.025052864	ENSMUSG00000076870	Trdj1	2.896677318	0.041057757
ENSMUSG00000062345	Serpinb2	2.470159949	2.4421E-256	ENSMUSG00000009185	Ccl8	2.86904941	5.75733E-33
ENSMUSG00000029379	Cxcl3	2.412978301	1.039E-191	ENSMUSG00000087659	Gm12606	2.856330095	0.000172051
ENSMUSG00000114996	Gm48958	2.393376759	0.035728656	ENSMUSG00000106208	Gm4734	2.849673595	0.000521432
ENSMUSG00000052496	Pkdrej	2.360643129	0.002259473	ENSMUSG00000104116	Gm37296	2.81672732	0.000104611
ENSMUSG00000111709	Gm3776	2.346435932	3.0497E-09	ENSMUSG00000112795	Gm2027	2.807916074	0.013113806
ENSMUSG00000018916	Csf2	2.343917005	3.09261E-78	ENSMUSG00000042244	Pglyrp3	2.781893522	0.038656284
ENSMUSG00000050635	Sprr2f	2.333483621	0.001044239	ENSMUSG00000103907	Gm37498	2.718052626	0.025007101
ENSMUSG00000005800	Mmp8	2.322470932	1.57751E-24	ENSMUSG00000100843	Gm8307	2.685499483	0.004952225
ENSMUSG00000068794	Col28a1	2.303236317	1.1805E-171	ENSMUSG00000006014	Prg4	2.683040817	1.0511E-239
ENSMUSG00000050578	Mmp13	2.297974597	1.35971E-73	ENSMUSG00000068231	Vmn1r43	2.677312849	0.043142328
ENSMUSG00000103720	Gm37094	2.230719313	0.010000982	ENSMUSG00000102158	Gm34342	2.667731593	0.046308608
ENSMUSG00000116606	Gm10479	2.229462549	0.02496665	ENSMUSG00000028373	Astn2	2.641556045	5.22759E-10
ENSMUSG00000102856	Gm37084	2.20357233	0.001446951	ENSMUSG00000056073	Grik2	2.639339938	1.436E-37
ENSMUSG00000020676	Ccl11	2.200337831	0.000288451	ENSMUSG00000068794	Col28a1	2.618122915	6.7381E-221
ENSMUSG00000068587	Mgam	2.199088679	4.48007E-12	ENSMUSG00000109864	Eid3	2.612399419	2.9028E-39
ENSMUSG00000034981	Parm1	2.191444715	4.5846E-195	ENSMUSG00000005800	Mmp8	2.599328778	3.96442E-34
ENSMUSG00000050587	Lrrc4c	2.191333784	1.25342E-11	ENSMUSG00000020374	Rasgef1c	2.596831951	1.27231E-12
ENSMUSG00000075224	Lrrc55	2.180043352	0.000657634	ENSMUSG00000032878	Ccdc85a	2.580323906	1.49665E-10
ENSMUSG00000021478	Drd1	2.178036514	2.72603E-06	ENSMUSG00000101199	Gm9687	2.579371842	0.005049584
ENSMUSG00000054215	Sprr2k	2.177168355	0.00018684	ENSMUSG00000025889	Snca	2.576655601	0.001839983
ENSMUSG00000038259	Gdf5	2.175358483	1.90368E-07	ENSMUSG00000020014	Cfap54	2.573490516	0.002840409
ENSMUSG00000103065	Gm20236	2.171035883	0.015078536	ENSMUSG00000027408	Cpxm1	2.561900638	0
ENSMUSG00000037738	Nek5	2.145514606	2.67121E-06	ENSMUSG00000046259	Sprr2h	2.560772413	2.76498E-09
ENSMUSG00000089744	Gm16146	2.145166149	0.000370011	ENSMUSG00000024331	Dsc2	2.554452324	1.0605E-13
ENSMUSG00000109771	Gm35315	2.136791078	1.09444E-07	ENSMUSG00000007682	Dio2	2.546018406	3.38973E-75
ENSMUSG00000108122	Gm44116	2.134569471	0.001071833	ENSMUSG00000034981	Parm1	2.540712098	9.868E-282
ENSMUSG00000105382	Gm43339	2.133791227	0.007131226	ENSMUSG00000107134	Gm42528	2.536784157	4.29021E-15
ENSMUSG00000026185	Igfbp5	2.116781047	2.45799E-42	ENSMUSG00000044017	Adgrd1	2.536723195	1.19231E-45
ENSMUSG00000097675	1700101I11Rik	2.113214458	2.33949E-06	ENSMUSG00000050578	Mmp13	2.530972674	9.2969E-101
ENSMUSG00000036330	Slc18a1	2.106610503	1.72765E-08	ENSMUSG00000079657	Rab26	2.530833316	2.3026E-08
ENSMUSG00000028031	Dkk2	2.104870786	1.88464E-25	ENSMUSG00000096361	Gm5814	2.521779658	0.028805519
ENSMUSG00000034755	Pcdh11x	2.103967551	3.36795E-08	ENSMUSG00000028128	F3	2.518012649	0
ENSMUSG00000027399	Il1a	2.092249291	2.65413E-13	ENSMUSG00000037196	Pacrg	2.506911723	0.044959258
ENSMUSG00000104293	Gm38043	2.089813427	0.031145592	ENSMUSG00000025503	Taldo1	2.492234318	0
ENSMUSG00000030623	Prss23os	2.082648851	1.1971E-08	ENSMUSG00000050808	Muc15	2.489117439	1.06439E-25
ENSMUSG00000051022	Hs3st1	2.063763207	1.70202E-39	ENSMUSG00000104801	Gm43834	2.478262031	0.000101331
ENSMUSG00000107478	Gm45234	2.048829039	0.001485446	ENSMUSG00000063632	Sox11	2.45715589	5.04422E-24
ENSMUSG00000111424	Gm48478	2.01598759	0.00252244	ENSMUSG00000048029	Eno4	2.45379422	0.01106704
ENSMUSG00000006567	Atp7b	1.997913834	0.000737036	ENSMUSG00000036381	P2ry14	2.450878995	2.86933E-08
ENSMUSG00000032532	Cck	1.990521046	9.899E-05	ENSMUSG00000038071	Npy6r	2.436534363	1.81574E-08
ENSMUSG00000025889	Snca	1.981774313	0.038293233	ENSMUSG00000045392	Olfr1033	2.434685251	2.5742E-58
ENSMUSG00000085180	AI838599	1.971786512	1.59836E-05	ENSMUSG00000048747	E130114P18Rik	2.424041327	0.047504126
ENSMUSG00000066755	Tnfsf18	1.953763579	0	ENSMUSG00000104876	Trdc	2.418715198	8.52042E-22
ENSMUSG00000054598	9130230L23Rik	1.944283781	0.044972342	ENSMUSG00000057000	Nxf3	2.411971334	1.66742E-12
ENSMUSG00000038496	Slc19a3	1.940438812	4.01359E-11	ENSMUSG00000115759	Gm18787	2.41154699	4.03009E-15
ENSMUSG00000028602	Tnfrsf8	1.938751958	0.000541301	ENSMUSG00000062342	Serpinb9e	2.399594416	0.0001472
ENSMUSG00000049100	Pcdh10	1.926776007	7.24417E-05	ENSMUSG00000018500	Adora2b	2.397419557	4.8117E-225
ENSMUSG00000038067	Csf3	1.919148037	9.20089E-10	ENSMUSG00000117335	Gm49870	2.390895456	0.00359133
ENSMUSG00000103408	Gm37933	1.899459254	0.034287696	ENSMUSG00000040026	Saa3	2.390257392	1.0791E-13
ENSMUSG00000026235	Epha4	1.892741154	1.33257E-38	ENSMUSG00000079138	Gm8818	2.377860928	1.26729E-24
ENSMUSG00000049511	Htr1b	1.889070449	5.81228E-16	ENSMUSG00000038259	Gdf5	2.365136151	1.9847E-09
ENSMUSG00000036437	Npy1r	1.868926894	0.004451208	ENSMUSG00000049676	Catsperg1	2.354774361	2.89453E-16
ENSMUSG00000021573	Tppp	1.868365941	0.017640007	ENSMUSG00000026235	Epha4	2.348884258	8.16694E-74
ENSMUSG00000103976	Gm37677	1.857738543	0.000482524	ENSMUSG00000033854	Kcnk10	2.340690954	3.8939E-28
ENSMUSG00000043613	Mmp3	1.85186401	0	ENSMUSG00000019966	Kitl	2.333985113	0
ENSMUSG00000092539	Gm20468	1.849962481	0.023216684	ENSMUSG00000050587	Lrrc4c	2.315964723	7.01381E-13
ENSMUSG00000025503	Taldo1	1.845664223	0	ENSMUSG00000086124	A530076I17Rik	2.313542914	0.009684179
ENSMUSG00000021732	Fgf10	1.842189515	1.76743E-36	ENSMUSG00000073761	4933427I04Rik	2.311349628	0.015824735
ENSMUSG00000003949	Hlf	1.839355071	2.93354E-12	ENSMUSG00000115898	Gm49524	2.306280199	0.022972298
ENSMUSG00000027832	Ptx3	1.837290412	0	ENSMUSG00000115099	1700087I21Rik	2.29824871	7.93837E-05
ENSMUSG00000038276	Asic3	1.821450428	4.93272E-10	ENSMUSG00000111052	Gm20139	2.288293989	0.037210973
ENSMUSG00000048644	Ctxn1	1.805401078	3.18784E-10	ENSMUSG00000032348	Gsta4	2.286399797	2.62016E-95
ENSMUSG00000000730	Dnmt3l	1.797960515	1.4721E-24	ENSMUSG00000043003	Rasef	2.278545516	1.22154E-05

ENSMUSG00000035373	Ccl7	1.787556594	0	ENSMUSG00000031613	Hpgd	2.26064592	1.88472E-50
ENSMUSG00000097292	A230107N01Rik	1.779115654	0.023438602	ENSMUSG00000003949	Hlf	2.247958479	5.51221E-27
ENSMUSG00000024331	Dsc2	1.773793338	4.19789E-05	ENSMUSG00000046598	Bdh1	2.241639133	1.47624E-38
ENSMUSG00000036197	Gxylt1	1.771329553	0	ENSMUSG00000025784	Clec3b	2.231368058	4.56851E-08
ENSMUSG00000058665	En1	1.768742335	4.65189E-14	ENSMUSG00000047884	Klk9	2.222130897	0.002626665
ENSMUSG00000090145	Ugt1a6b	1.767630064	5.38604E-06	ENSMUSG00000041620	Mmp1b	2.212478535	1.50045E-09
ENSMUSG00000115483	Gm9732	1.751633384	0.044207977	ENSMUSG00000049100	Pcdh10	2.203015357	3.91068E-07
ENSMUSG00000074183	Gsta1	1.751181259	3.86262E-10	ENSMUSG00000032845	Alpk2	2.197774116	1.89132E-22
ENSMUSG00000087001	Gm15475	1.746594407	0.005832784	ENSMUSG00000052180	Serpinb6c	2.187482115	3.36492E-30
ENSMUSG00000100987	Gm1627	1.746142764	0.033108166	ENSMUSG00000050395	Tnfsf15	2.186662944	0
ENSMUSG00000032257	Ankk1	1.726075571	0.001808785	ENSMUSG00000027375	Mal	2.186346732	2.71625E-63
ENSMUSG00000115667	Gm10389	1.720931698	0.01256562	ENSMUSG00000087674	4930447M23Rik	2.167798012	0.004779245
ENSMUSG00000056413	Adap1	1.710374034	0.000479899	ENSMUSG00000031289	Il13ra2	2.167612513	5.97007E-38
ENSMUSG00000093388	Gm22655	1.705845177	0.027412252	ENSMUSG00000040009	Gnaz	2.164416008	0.04808119
ENSMUSG00000099803	Gm28863	1.694421277	0.015660126	ENSMUSG00000048138	Dmrt2	2.155640584	0.000126555
ENSMUSG00000046593	Tmem215	1.676862935	1.24318E-11	ENSMUSG00000019832	Rab32	2.151703275	0
ENSMUSG00000052026	Slc6a7	1.672836159	0.001004437	ENSMUSG00000091573	Serpina3d-ps	2.147946403	0.021748798
ENSMUSG00000092341	Malat1	1.662927159	0.001339508	ENSMUSG00000044626	Liph	2.145197546	1.4812E-08
ENSMUSG00000071036	Gm10309	1.651595636	0.0028305	ENSMUSG00000079101	Esd-ps	2.142403094	3.13971E-06
ENSMUSG00000096992	Gm26788	1.651557746	0.001122143	ENSMUSG00000061654	Spry3	2.141234071	6.95874E-29
ENSMUSG00000109864	Eid3	1.647588927	2.05618E-11	ENSMUSG00000036437	Npy1r	2.125980822	0.000106348
ENSMUSG00000044288	Cnr1	1.626870627	0.003436963	ENSMUSG00000103195	Gm37644	2.112468569	0.000222073
ENSMUSG00000019326	Aoc3	1.620975851	7.88675E-17	ENSMUSG00000085826	Gm15638	2.111404639	2.27689E-08
ENSMUSG00000021313	Ryr2	1.615135144	0.048123188	ENSMUSG00000002565	Scin	2.102869294	2.5561E-102
ENSMUSG00000000934	Top1mt	1.608452476	8.8575E-22	ENSMUSG00000021214	Akr1c18	2.094116649	8.43619E-07
ENSMUSG00000030134	Rasgef1a	1.598884095	5.91062E-07	ENSMUSG00000115414	Gm49257	2.092891097	0.000152177
ENSMUSG00000017737	Mmp9	1.598472577	0.000353489	ENSMUSG00000086358	Gm13270	2.092340236	0.000137929
ENSMUSG00000106121	Gm42679	1.596489227	1.47599E-12	ENSMUSG00000072934	Trpc5os	2.089538728	1.0661E-05
ENSMUSG00000022206	Npr3	1.584072847	6.2391E-127	ENSMUSG00000029378	Areg	2.080217265	1.87623E-07
ENSMUSG00000072934	Trpc5os	1.578746796	0.005702296	ENSMUSG00000033453	Adamts15	2.070833433	0.003662005
ENSMUSG00000020123	Avpr1a	1.576496909	1.46377E-55	ENSMUSG00000086320	Gm12840	2.06477645	4.52417E-42
ENSMUSG00000042436	Mfap4	1.571381593	9.63507E-51	ENSMUSG00000039099	Wdr93	2.06323014	5.89555E-05
ENSMUSG00000044349	Snhg11	1.56507027	2.74995E-30	ENSMUSG00000045294	Insig1	2.048226643	4.5264E-238
ENSMUSG00000020427	Igfbp3	1.563610832	2.2085E-160	ENSMUSG00000002459	Rgs20	2.043439519	0.007540725
ENSMUSG00000107696	Gm44171	1.557986939	0.005963453	ENSMUSG00000028362	Tnfsf8	2.042918971	3.2971E-11
ENSMUSG00000063234	Gpr84	1.541200286	0.02301214	ENSMUSG00000054944	5330416C01Rik	2.042440991	0.033345747
ENSMUSG00000022321	Cdh10	1.540551071	0.004433829	ENSMUSG00000027360	Hdc	2.039275504	3.691E-09
ENSMUSG00000050092	Sprr2b	1.525060139	1.77634E-06	ENSMUSG00000073514	Dok6	2.038963287	8.26105E-08
ENSMUSG00000105679	Gm43338	1.524752698	0.019514335	ENSMUSG00000102030	Gm29106	2.035005932	0.000312928
ENSMUSG00000031340	Gabre	1.521734594	9.61242E-26	ENSMUSG00000035258	Abi3bp	2.030877933	3.49511E-42
ENSMUSG00000047330	Kcne4	1.516832664	1.2521E-189	ENSMUSG00000097675	1700101I11Rik	2.030747684	3.57056E-06
ENSMUSG00000085440	Sorbs2os	1.514318417	0.048486622	ENSMUSG00000047686	Rtl3	2.020940545	2.36631E-10
ENSMUSG00000048376	F2r	1.506946564	0	ENSMUSG00000024810	Il33	2.019742111	2.1977E-101
ENSMUSG00000103195	Gm37644	1.505659803	0.035778824	ENSMUSG00000032420	Nt5e	2.016869833	1.1425E-105
ENSMUSG00000078496	Zfp982	1.501926176	8.09201E-17	ENSMUSG00000020059	Sycp3	2.010928701	0.014624895
ENSMUSG00000031762	Mt2	1.494617423	0	ENSMUSG00000082806	Rpl13-ps1	2.007483502	0.02215159
ENSMUSG00000051367	Six1	1.491118519	2.76853E-16	ENSMUSG00000035513	Ntng2	2.001661701	3.47902E-32
ENSMUSG00000037185	Krt80	1.489436707	2.21762E-32	ENSMUSG00000030433	Sbk2	1.992524521	0.039734751
ENSMUSG00000039865	Slc44a3	1.487835055	2.25351E-05	ENSMUSG00000028602	Tnfrsf8	1.990952526	7.69221E-05
ENSMUSG00000028373	Astn2	1.478327974	0.021043589	ENSMUSG00000030623	Prss23os	1.989064593	2.39615E-09
ENSMUSG00000058427	Cxcl2	1.47313516	2.27667E-42	ENSMUSG00000003665	Has1	1.986798629	5.6916E-48
ENSMUSG00000024401	Tnf	1.467179425	0.003909451	ENSMUSG00000085582	3110099E03Rik	1.983977595	0.006876814
ENSMUSG00000113985	9130015A21Rik	1.465007239	3.02829E-06	ENSMUSG00000043613	Mmp3	1.977501294	0
ENSMUSG00000116338	7530414M10Rik	1.464803728	0.041774244	ENSMUSG00000027399	Il1a	1.96988556	4.70387E-13
ENSMUSG00000037428	Vgf	1.463995882	2.31044E-10	ENSMUSG00000019880	Rspo3	1.969427861	9.97622E-19
ENSMUSG00000023039	Krt7	1.459232935	2.64148E-08	ENSMUSG00000049511	Htr1b	1.968576059	1.31893E-18
ENSMUSG00000056427	Slit3	1.45438423	1.73883E-41	ENSMUSG00000025037	Maoa	1.965824459	0
ENSMUSG00000042659	Arrdc4	1.447370151	8.90058E-85	ENSMUSG00000026399	Cd55	1.96528304	0
ENSMUSG00000057522	Spop	1.440288758	0	ENSMUSG00000111818	Gm17749	1.960005469	0.015606383
ENSMUSG00000026822	Lcn2	1.433526281	1.80634E-61	ENSMUSG00000026114	Cnga3	1.957081574	0.00024279
ENSMUSG00000035258	Abi3bp	1.432243196	9.67115E-16	ENSMUSG00000078866	Zfp970	1.949482912	4.52831E-55
ENSMUSG00000052605	Isoc2b	1.424466941	3.92765E-10	ENSMUSG00000071713	Csf2rb	1.947805329	1.84858E-07
ENSMUSG00000023045	Soat2	1.41921617	0.01927539	ENSMUSG00000047330	Kcne4	1.937261899	0
ENSMUSG00000050447	Lypd6	1.4174319	0.042802798	ENSMUSG00000056413	Adap1	1.933337703	3.775E-05
ENSMUSG00000049128	Ivl	1.416008762	2.91618E-35	ENSMUSG00000043085	Tmem82	1.930194136	9.81619E-08
ENSMUSG00000085058	8030453O22Rik	1.412751082	2.26299E-05	ENSMUSG00000033377	Palmd	1.929481557	1.03108E-11
ENSMUSG00000014813	Stc1	1.41068835	9.60415E-13	ENSMUSG00000027832	Ptx3	1.927254574	0
ENSMUSG00000036006	Ripor2	1.410056423	2.08403E-33	ENSMUSG00000026971	Itgb6	1.9259853	4.93227E-09
ENSMUSG00000045294	Insig1	1.40864589	3.9018E-118	ENSMUSG00000026888	Grb14	1.92503318	5.1034E-132
ENSMUSG00000029371	Cxcl5	1.408204351	0	ENSMUSG00000094786	Gm14403	1.9244161	1.05165E-14
ENSMUSG00000028780	Sema3c	1.40750009	7.46931E-82	ENSMUSG00000098132	Rassf10	1.923685112	0.021622385
ENSMUSG00000018500	Adora2b	1.39877135	6.65304E-67	ENSMUSG00000021478	Drd1	1.908889671	5.82727E-05
ENSMUSG00000050860	Phospho1	1.39682287	9.98311E-12	ENSMUSG00000086040	Wipf3	1.907699924	3.10088E-11
ENSMUSG00000034993	Vat1	1.396300593	0	ENSMUSG00000078868	Gm14412	1.904983731	3.83019E-05
ENSMUSG00000049721	Gal3st1	1.393377158	8.52017E-06	ENSMUSG00000022122	Ednrb	1.904673671	4.51273E-46
ENSMUSG00000107810	Gm18609	1.39058117	0.000124183	ENSMUSG00000034810	Scn7a	1.899381929	2.1167E-267
ENSMUSG00000101678	Gm29609	1.390341681	0.000677239	ENSMUSG00000030672	Mylpf	1.898654004	2.89255E-06

ENSMUSG00000073514	Dok6	1.38873474	0.001002256	ENSMUSG00000041347	Bdkrb1	1.89846576	1.33479E-05
ENSMUSG00000074024	4632427E13Rik	1.387566234	4.33587E-13	ENSMUSG00000092274	Neat1	1.886885615	0
ENSMUSG00000006345	Ggt1	1.386166273	0.000204004	ENSMUSG00000078439	Smim24	1.885959599	0.000102955
ENSMUSG00000092200	Tnxa	1.385755002	0.000209853	ENSMUSG00000036218	Pdzrn4	1.879837746	0.001871836
ENSMUSG00000044788	Fads6	1.384510275	1.28575E-33	ENSMUSG00000020334	Slc22a4	1.876143953	4.045E-15
ENSMUSG00000049252	Lrp1b	1.383387091	0.030466246	ENSMUSG00000025321	Itgb8	1.874814601	1.2009E-181
ENSMUSG00000101086	Gm28651	1.380391139	0.02206117	ENSMUSG00000065952	Rps23rg1	1.871113667	0.02378561
ENSMUSG00000032332	Col12a1	1.374792097	0	ENSMUSG00000085058	8030453O22Rik	1.861396732	4.68007E-10
ENSMUSG00000103593	Gm37352	1.373274255	1.48187E-06	ENSMUSG00000042659	Arrdc4	1.846766243	3.4511E-149
ENSMUSG00000053615	Gm9913	1.371861824	1.61833E-09	ENSMUSG00000031340	Gabre	1.837402473	1.64348E-39
ENSMUSG00000009185	Ccl8	1.362474233	2.03061E-05	ENSMUSG00000016349	Eef1a2	1.826651642	9.28018E-32
ENSMUSG00000055480	Zfp458	1.358993082	3.02724E-14	ENSMUSG00000028974	Dffa	1.817454219	2.44428E-91
ENSMUSG00000030022	Adamts9	1.357382225	1.8948E-165	ENSMUSG00000036144	Meox2	1.812167965	2.82068E-50
ENSMUSG00000064202	4430402I18Rik	1.351926652	0.032513746	ENSMUSG00000090516	Rps11-ps1	1.810404054	0.034992379
ENSMUSG00000085781	Gm15640	1.348469661	3.49348E-08	ENSMUSG00000030187	Klra2	1.808083308	1.5924E-05
ENSMUSG00000041958	Pigs	1.344364865	8.4755E-131	ENSMUSG00000095054	Gm13146	1.806501479	0.026035394
ENSMUSG00000102691	Gm37780	1.338022458	0.002455594	ENSMUSG00000045319	Proser2	1.804073103	0.045222054
ENSMUSG00000019966	Kitl	1.336096644	5.547E-295	ENSMUSG00000111148	Gm2774	1.80382763	0.040043903
ENSMUSG00000114544	Gm49146	1.330543597	0.00610084	ENSMUSG00000113548	Gm34047	1.803207248	0.025624515
ENSMUSG00000041945	Mfsd9	1.32816306	5.64455E-14	ENSMUSG00000048647	Exd1	1.802803033	3.70182E-09
ENSMUSG00000073274	Gm14636	1.327046212	0.009277244	ENSMUSG00000037463	Fbxo27	1.80077228	0.004346886
ENSMUSG00000097111	Peak1os	1.322475171	0.022565106	ENSMUSG00000050092	Sprr2b	1.800369466	4.96805E-09
ENSMUSG00000028583	Pdpn	1.320565412	0	ENSMUSG00000085180	AI838599	1.790989335	8.97865E-05
ENSMUSG00000028370	Pappa	1.319521711	3.7214E-113	ENSMUSG00000021303	Gng4	1.790251995	9.80085E-07
ENSMUSG00000043085	Tmem82	1.316973224	0.005278321	ENSMUSG00000055301	Adh7	1.783119834	9.95861E-23
ENSMUSG00000094786	Gm14403	1.315662584	3.88133E-06	ENSMUSG00000027562	Car2	1.782279778	0.019472904
ENSMUSG00000031853	Map3k21	1.314072657	0.001764949	ENSMUSG00000102684	Gm37374	1.780799039	0.038292215
ENSMUSG00000106426	Gm36211	1.308614388	0.000259333	ENSMUSG00000097254	C430042M11Rik	1.777481527	9.55844E-05
ENSMUSG00000004885	Crabp2	1.308429622	0.000586515	ENSMUSG00000105204	Gm43738	1.766222471	0.005634655
ENSMUSG00000024806	Mlana	1.305441748	3.5831E-10	ENSMUSG00000072769	Gm10419	1.762811179	0.000605721
ENSMUSG00000084817	Gm5526	1.303512496	5.60129E-08	ENSMUSG00000043263	Ifi209	1.760114847	0.00672218
ENSMUSG00000020334	Slc22a4	1.302317817	4.86376E-06	ENSMUSG00000097712	Gm10532	1.757534098	0.010961812
ENSMUSG00000039701	Usp53	1.301264013	1.0126E-138	ENSMUSG00000029321	Slc10a6	1.75604528	1.7693E-09
ENSMUSG00000022144	Gdnf	1.300854428	1.41289E-31	ENSMUSG00000106149	Gm43430	1.754060941	0.002414464
ENSMUSG00000038555	Reep2	1.298965804	6.63003E-05	ENSMUSG00000050447	Lypd6	1.751734288	0.001136018
ENSMUSG00000034258	Flvcr2	1.296359048	1.45853E-07	ENSMUSG00000071226	Cecr2	1.749592753	5.00738E-05
ENSMUSG00000064105	Cnnm2	1.293237724	6.61098E-14	ENSMUSG00000000730	Dnmt3l	1.744763671	3.16493E-24
ENSMUSG00000114469	C730002L08Rik	1.293043203	0.007101741	ENSMUSG00000109517	Gm44763	1.742826177	1.95016E-06
ENSMUSG00000020826	Nos2	1.289789185	3.0607E-66	ENSMUSG00000053930	Shisa6	1.740387476	0.021053332
ENSMUSG00000022383	Ppara	1.288647527	0.002783368	ENSMUSG00000050721	Plekho2	1.73895206	0
ENSMUSG00000041649	Klf8	1.28563392	8.1487E-07	ENSMUSG00000021091	Serpina3n	1.735495779	0
ENSMUSG00000050989	Selenon	1.28546336	4.2185E-224	ENSMUSG00000048142	Nat8l	1.730847583	9.68246E-06
ENSMUSG00000112963	Gm6093	1.285101442	9.2032E-09	ENSMUSG00000073077	Cfap47	1.730118352	0.034362607
ENSMUSG00000060961	Slc4a4	1.266617988	1.30898E-46	ENSMUSG00000044337	Ackr3	1.725905921	2.14218E-59
ENSMUSG00000073433	Arhgdig	1.26527596	0.003366604	ENSMUSG00000032532	Cck	1.723714078	0.005995621
ENSMUSG00000063018	2010204K13Rik	1.257075141	0.002610626	ENSMUSG00000048706	Lurap1l	1.712516878	7.75669E-38
ENSMUSG00000038296	Galnt18	1.256913872	1.7762E-35	ENSMUSG00000105565	Gm43566	1.707395904	4.81808E-07
ENSMUSG00000006014	Prg4	1.252836288	1.00433E-35	ENSMUSG00000028327	Stra6l	1.695366531	0.005153803
ENSMUSG00000105194	Gm6755	1.246932339	0.041171037	ENSMUSG00000030553	Pgpep1l	1.694383249	1.92161E-08
ENSMUSG00000032420	Nt5e	1.238907612	2.33998E-36	ENSMUSG00000033177	Tmprss7	1.693433255	0.010820394
ENSMUSG00000105565	Gm43566	1.237380983	0.001769409	ENSMUSG00000035506	Slc12a8	1.685153644	7.51723E-13
ENSMUSG00000021575	Ahrr	1.23672074	5.02711E-10	ENSMUSG00000021758	Ddx4	1.682333685	0.000340157
ENSMUSG00000067365	Tmem128	1.235790689	2.6737E-113	ENSMUSG00000052605	Isoc2b	1.68225323	3.692E-15
ENSMUSG00000040606	Kazn	1.233183343	3.38502E-57	ENSMUSG00000106457	Gm42585	1.678475741	0.048179638
ENSMUSG00000039519	Cyp7b1	1.229216863	3.22326E-12	ENSMUSG00000034755	Pcdh11x	1.674361822	6.25655E-05
ENSMUSG00000041696	Rasl12	1.2285234	1.88295E-15	ENSMUSG00000066755	Tnfsf18	1.669144402	2.0174E-192
ENSMUSG00000097028	Ptgs2os	1.222538814	9.51124E-06	ENSMUSG00000054545	Ugt1a6a	1.667175304	2.80833E-66
ENSMUSG00000052430	Bmpr1b	1.221527864	1.10161E-10	ENSMUSG00000037652	Phc3	1.662828429	0.017099831
ENSMUSG00000090093	Gm14399	1.219432095	7.8533E-09	ENSMUSG00000050751	Pgbd5	1.65773627	0.000839569
ENSMUSG00000000340	Dbt	1.214823102	1.83419E-52	ENSMUSG00000096917	2500002B13Rik	1.655035402	0.007609113
ENSMUSG00000029335	Bmp3	1.214528124	0.00141347	ENSMUSG00000017400	Stac2	1.651487139	5.18979E-13
ENSMUSG00000021758	Ddx4	1.211541386	0.029273212	ENSMUSG00000045362	Tnfrsf26	1.637762761	0
ENSMUSG00000031936	Hephl1	1.211341304	0.000163435	ENSMUSG00000106205	C230096K16Rik	1.634559583	0.000872194
ENSMUSG00000030407	Qpctl	1.208248093	1.63854E-49	ENSMUSG00000020363	Gfpt2	1.625847878	0
ENSMUSG00000015702	Anxa9	1.207517094	0.001212757	ENSMUSG00000044734	Serpinb1a	1.616008163	8.9167E-151
ENSMUSG00000074766	Ism1	1.205114865	8.95421E-15	ENSMUSG00000113985	9130015A21Rik	1.61589543	9.52295E-09
ENSMUSG00000106339	Gm43489	1.204030391	8.53814E-05	ENSMUSG00000022894	Adamts5	1.610526504	2.683E-282
ENSMUSG00000110393	Gm36445	1.20306749	0.005236916	ENSMUSG00000053615	Gm9913	1.608253233	1.08885E-14
ENSMUSG00000020473	Aebp1	1.199788702	0	ENSMUSG00000104861	3110039M20Rik	1.602475158	0.001023049
ENSMUSG00000063245	Zfp993	1.199426545	4.07484E-24	ENSMUSG00000101625	Gm29371	1.599619554	5.25605E-06
ENSMUSG00000041842	Fhdc1	1.199219349	3.05512E-98	ENSMUSG00000063458	Lrmda	1.596130019	1.47915E-06
ENSMUSG00000084289	Gm6977	1.196241623	0.049294268	ENSMUSG00000023826	Prkn	1.596045428	0.002446332
ENSMUSG00000104476	Gm38211	1.195929593	0.034894117	ENSMUSG00000021732	Fgf10	1.596040592	1.14594E-25
ENSMUSG00000002565	Scin	1.195482369	8.82366E-21	ENSMUSG00000106617	Gm36266	1.592350067	0.0321142
ENSMUSG00000005125	Ndrg1	1.194498164	1.35978E-47	ENSMUSG00000048636	A730049H05Rik	1.590921498	5.51424E-15
ENSMUSG00000107879	9330102E08Rik	1.193648288	2.99914E-05	ENSMUSG00000008540	Mgst1	1.583981376	0
ENSMUSG00000039405	Prss23	1.191723624	0	ENSMUSG00000064202	4430402I18Rik	1.579863962	0.001069922

ENSMUSG00000024529	Lox	1.188805316	0
ENSMUSG00000086320	Gm12840	1.188720645	1.82535E-09
ENSMUSG00000002489	Tiam1	1.188652206	1.11972E-64
ENSMUSG00000050022	Amz1	1.188375647	4.10575E-09
ENSMUSG00000025933	Tmem14a	1.186246134	4.15303E-06
ENSMUSG00000026701	Prdx6	1.182836126	3.4702E-227
ENSMUSG00000050721	Plekho2	1.181378798	1.1597E-218
ENSMUSG00000071650	Ganab	1.173815678	3.6899E-260
ENSMUSG00000007564	Ppp2r1a	1.172127167	4.4377E-286
ENSMUSG00000049300	Prmt6	1.172017593	1.32088E-34
ENSMUSG00000011256	Adam19	1.170940405	0
ENSMUSG00000048616	Nog	1.170234078	4.85969E-05
ENSMUSG00000045573	Penk	1.167294813	0.003097112
ENSMUSG00000021098	4930447C04Rik	1.167188321	0.000986051
ENSMUSG00000046589	Lrrc8e	1.156889967	1.10492E-05
ENSMUSG00000063632	Sox11	1.151774846	0.001337048
ENSMUSG00000040663	Clcf1	1.151494683	2.1881E-100
ENSMUSG00000019880	Rspo3	1.14840122	1.16592E-05
ENSMUSG00000028766	Alpl	1.148205058	1.26885E-10
ENSMUSG00000038679	Trps1	1.145893612	6.53458E-81
ENSMUSG00000069540	Gm4925	1.144949097	0.021646113
ENSMUSG00000021091	Serpina3n	1.142687182	0
ENSMUSG00000104876	Trdc	1.14226533	0.00343581
ENSMUSG00000020431	Adcy1	1.140727559	2.60844E-11
ENSMUSG00000024661	Fth1	1.140282701	0
ENSMUSG00000037362	Ccn3	1.138701158	9.68884E-50
ENSMUSG00000110411	Gm45457	1.137698769	0.040725634
ENSMUSG00000045362	Tnfrsf26	1.1366981	5.9299E-138
ENSMUSG00000098009	Gm5597	1.136106784	0.028457188
ENSMUSG00000028420	Tmem38b	1.132516473	1.29237E-79
ENSMUSG00000090634	Gm8126	1.129633797	0.027437058
ENSMUSG00000025892	Gria4	1.129138264	1.77711E-32
ENSMUSG00000045201	Lrrc3b	1.128242389	5.34289E-13
ENSMUSG00000031596	Slc7a2	1.127171168	3.1309E-302
ENSMUSG00000075000	Nrbf2	1.124185813	3.52976E-41
ENSMUSG00000084996	Gm11419	1.123553209	0.001561947
ENSMUSG00000019852	Arfgef3	1.122689141	5.54104E-05
ENSMUSG00000028539	Artn	1.121805492	0.006296115
ENSMUSG00000090564	A430057M04Rik	1.118660318	1.88587E-08
ENSMUSG00000028974	Dffa	1.115692117	5.84969E-26
ENSMUSG00000022800	Fyttd1	1.115500346	9.8817E-167
ENSMUSG00000110588	Gm45774	1.109726001	2.79472E-10
ENSMUSG00000031765	Mt1	1.108596254	1.5995E-156
ENSMUSG00000032802	Srxn1	1.108540756	3.657E-175
ENSMUSG00000054545	Ugt1a6a	1.108540718	2.01855E-17
ENSMUSG00000034810	Scn7a	1.106668337	1.41917E-71
ENSMUSG00000097318	1700007L15Rik	1.106658483	0.013363235
ENSMUSG00000006462	A530013C23Rik	1.104406329	0.017056374
ENSMUSG00000048706	Lurap1l	1.103676302	2.25758E-12
ENSMUSG00000037104	Socs5	1.103137873	1.4153E-206
ENSMUSG00000041912	Tdrkh	1.101900591	2.39689E-10
ENSMUSG00000006818	Sod2	1.100589419	3.4049E-202
ENSMUSG00000011008	Mcoln2	1.10033494	7.22111E-12
ENSMUSG00000034220	Gpc1	1.099806319	2.92565E-64
ENSMUSG00000002997	Prkar2b	1.095571604	8.1658E-270
ENSMUSG00000022514	Il1rap	1.095519042	0
ENSMUSG00000033327	Tnxb	1.095502549	2.4578E-113
ENSMUSG00000104917	Gm43289	1.093844278	0.007362898
ENSMUSG00000028362	Tnfsf8	1.093828804	0.005921269
ENSMUSG00000074934	Grem1	1.092810702	1.32534E-25
ENSMUSG00000079677	Fdx2	1.09279858	0.032942475
ENSMUSG00000109297	Gm31522	1.092233734	0.00028266
ENSMUSG00000031383	Dusp9	1.092119368	0.002846923
ENSMUSG00000026888	Grb14	1.090741143	4.81057E-22
ENSMUSG00000111082	Gm30934	1.089267812	0.024476075
ENSMUSG00000028124	Gclm	1.087969622	9.999E-199
ENSMUSG00000078664	Sprr2a1	1.085576522	0.022095153
ENSMUSG00000030170	Wnt5b	1.082152564	0.007207131
ENSMUSG00000021190	Lgmn	1.081998077	3.3875E-176
ENSMUSG00000021090	Lrrc9	1.079488232	0.042183882
ENSMUSG00000042616	Oscp1	1.077454747	1.21138E-05
ENSMUSG00000022094	Slc39a14	1.076075117	1.898E-208
ENSMUSG00000046169	Adamts6	1.076007854	2.21806E-25
ENSMUSG00000021130	Galnt16	1.075430725	2.21443E-06
ENSMUSG00000024164	C3	1.072966343	2.59599E-13
ENSMUSG00000115785	Gm6740	1.070937072	0.000252411
ENSMUSG00000072940	Gm10443	1.066203801	0.039810816
ENSMUSG00000106339	Gm43489	1.57876575	1.50912E-07
ENSMUSG00000095512	Gm17222	1.575363896	0.009870776
ENSMUSG00000078952	Lncenc1	1.575216876	8.30891E-05
ENSMUSG00000064105	Cnnm2	1.569983781	2.15224E-22
ENSMUSG00000114469	C730002L08Rik	1.562884574	0.000355865
ENSMUSG00000017390	Aldoc	1.561246161	1.24032E-10
ENSMUSG00000025746	Il6	1.560529567	0
ENSMUSG00000026525	Opn3	1.560408831	1.44789E-10
ENSMUSG00000024897	Apba1	1.557180724	6.91111E-80
ENSMUSG00000026043	Col3a1	1.554337175	0
ENSMUSG00000044788	Fads6	1.550827149	7.96683E-48
ENSMUSG00000030162	Olr1	1.549277889	0
ENSMUSG00000024479	Mal2	1.541692648	0.000267676
ENSMUSG00000032179	Bmp5	1.539557968	0.003762447
ENSMUSG00000056296	Synpr	1.538735127	3.8887E-07
ENSMUSG00000115667	Gm10389	1.537110014	0.038077331
ENSMUSG00000047414	Flrt2	1.535229404	1.4234E-256
ENSMUSG00000020774	Aspa	1.53257514	0.007188457
ENSMUSG00000022861	Dgkg	1.53104466	0.000452657
ENSMUSG00000101086	Gm28651	1.530980646	0.004976098
ENSMUSG00000000934	Top1mt	1.529545303	9.23399E-23
ENSMUSG00000058427	Cxcl2	1.527255933	4.17813E-45
ENSMUSG00000014813	Stc1	1.526491787	1.1829E-14
ENSMUSG00000040978	Gm11992	1.519761836	0.017152304
ENSMUSG00000040035	Disp2	1.509497679	4.95913E-05
ENSMUSG00000105211	Gm47302	1.503149346	0.000502272
ENSMUSG00000044583	Tlr7	1.502598576	5.53574E-05
ENSMUSG00000037622	Wdtc1	1.495270837	4.1419E-101
ENSMUSG00000105651	1700017M07Rik	1.492983412	0.020718689
ENSMUSG00000025950	Idh1	1.486971693	0
ENSMUSG00000056427	Slit3	1.485672115	1.58678E-46
ENSMUSG00000032802	Srxn1	1.481899891	0
ENSMUSG00000035373	Ccl7	1.480641178	0
ENSMUSG00000075000	Nrbf2	1.480631174	1.39147E-76
ENSMUSG00000059588	Calcrl	1.479828277	1.5843E-174
ENSMUSG00000035863	Palm	1.477516706	8.9994E-156
ENSMUSG00000028583	Pdpn	1.477490981	0
ENSMUSG00000115785	Gm6740	1.477120342	1.51858E-09
ENSMUSG00000104476	Gm38211	1.47627277	0.00115965
ENSMUSG00000052125	F730043M19Rik	1.475675167	6.09941E-40
ENSMUSG00000000182	Fgf23	1.475173646	1.28948E-19
ENSMUSG00000020227	Irak3	1.471809818	1.38824E-58
ENSMUSG00000021687	Scamp1	1.469870443	1.8124E-188
ENSMUSG00000031762	Mt2	1.469003571	1.4896E-161
ENSMUSG00000039519	Cyp7b1	1.467339445	2.64069E-19
ENSMUSG00000085143	Gm11520	1.466537509	0.013677917
ENSMUSG00000038555	Reep2	1.465884003	3.65287E-06
ENSMUSG00000079055	Slc8a3	1.462067323	0.002722784
ENSMUSG00000022362	Gm29394	1.460909374	0.047312889
ENSMUSG00000028036	Ptgfr	1.460370235	0.007101781
ENSMUSG00000026198	Abcb6	1.460332646	1.24314E-31
ENSMUSG00000048644	Ctxn1	1.458204304	4.3172E-05
ENSMUSG00000005803	Sqor	1.454523929	9.3521E-157
ENSMUSG00000114018	Gm36495	1.450563725	0.029516175
ENSMUSG00000023991	Foxp4	1.446021326	8.15867E-68
ENSMUSG00000043342	Hoxd9	1.442249196	0.014782535
ENSMUSG00000079012	Serpina3m	1.44185184	1.84756E-06
ENSMUSG00000087213	2810408I11Rik	1.439223544	2.59131E-16
ENSMUSG00000021996	Esd	1.438558792	0
ENSMUSG00000006273	Atp6v1b2	1.437541225	0
ENSMUSG00000078942	Naip6	1.437455033	0.001546548
ENSMUSG00000075254	Heg1	1.43551455	0
ENSMUSG00000024053	Emilin2	1.429172139	4.4222E-178
ENSMUSG00000004885	Crabp2	1.428500605	7.21459E-05
ENSMUSG00000105194	Gm6755	1.427725282	0.008139505
ENSMUSG00000105891	A230001M10Rik	1.426826418	0.00535926
ENSMUSG00000031400	G6pdx	1.425812746	0
ENSMUSG00000067219	Nipal1	1.425153699	3.26919E-96
ENSMUSG00000026442	Nfasc	1.422512857	3.5448E-06
ENSMUSG00000037738	Nek5	1.419485943	0.010856698
ENSMUSG00000030022	Adamts9	1.419370232	8.636E-200
ENSMUSG00000007564	Ppp2r1a	1.416717126	0
ENSMUSG00000109305	Smim38	1.414006041	0.025533256
ENSMUSG00000045441	Gprin3	1.408153277	0.009796531
ENSMUSG00000117234	Gm7818	1.404322428	1.80308E-05
ENSMUSG00000039384	Dusp10	1.4021805	3.9873E-116
ENSMUSG00000105867	Gm42517	1.399659385	0.00032034

ENSMUSG00000092274	Neat1	1.065299017	9.16251E-44
ENSMUSG00000043811	Rtn4r	1.065198073	2.79525E-08
ENSMUSG00000110256	Gm45412	1.06503855	0.004058968
ENSMUSG00000068893	Sprr2a2	1.064631126	0.019451174
ENSMUSG00000042647	Acad12	1.064144309	1.2656E-07
ENSMUSG00000032068	Plet1	1.060559751	8.91144E-05
ENSMUSG00000052125	F730043M19Rik	1.057213253	6.67041E-18
ENSMUSG00000028751	Pla2g2e	1.05616417	3.55404E-05
ENSMUSG00000078700	D030028A08Rik	1.0561189	6.31716E-05
ENSMUSG00000046982	Tshz1	1.05573013	1.6144E-66
ENSMUSG00000020044	Timp3	1.05344614	0
ENSMUSG00000024360	Etf1	1.049042767	9.1301E-304
ENSMUSG00000064267	Hvcn1	1.048910176	3.90237E-48
ENSMUSG00000084350	Znf41-ps	1.048878809	6.99823E-41
ENSMUSG00000025192	Entpd7	1.048818918	2.02782E-31
ENSMUSG00000006241	Ccdc159	1.047108386	0.005565304
ENSMUSG00000050335	Lgals3	1.044802318	5.1897E-101
ENSMUSG00000054280	Prr14l	1.039583557	6.04176E-64
ENSMUSG00000021831	Ero1l	1.033942096	2.4723E-220
ENSMUSG00000069184	Zfp72	1.03326488	5.72689E-05
ENSMUSG00000027208	Fgf7	1.032360827	1.199E-108
ENSMUSG00000015536	Mocs2	1.030057832	2.61843E-65
ENSMUSG00000036242	Armh4	1.029684756	1.14304E-05
ENSMUSG00000109282	Gm45188	1.025466849	0.036009817
ENSMUSG00000036144	Meox2	1.024953826	1.6951E-13
ENSMUSG00000026475	Rgs16	1.02463789	2.605E-152
ENSMUSG00000034936	Arl4d	1.023524738	0.01420816
ENSMUSG00000060149	BC002059	1.023151482	1.01191E-07
ENSMUSG00000106825	2510016D11Rik	1.022593829	0.000758617
ENSMUSG00000029778	Adcyap1r1	1.022437196	8.6868E-171
ENSMUSG00000030257	Srgap3	1.022147918	3.00687E-63
ENSMUSG00000050896	Rtn4rl2	1.022085852	1.22217E-06
ENSMUSG00000030443	Zfp583	1.019800146	0.000265586
ENSMUSG00000067916	Zfp991	1.014071371	1.07348E-45
ENSMUSG00000107608	Gm43863	1.010828775	0.01480635
ENSMUSG00000062300	Nectin2	1.01009926	2.5336E-104
ENSMUSG00000039005	Tlr4	1.009572992	1.97394E-79
ENSMUSG00000041439	Mfsd6	1.007240722	8.54885E-37
ENSMUSG00000001910	Nacc1	1.006640305	7.8382E-127
ENSMUSG00000089698	Gm2541	1.005796086	0.01579655
ENSMUSG00000054640	Slc8a1	1.002583495	3.23331E-33
ENSMUSG00000117234	Gm7818	1.002255012	0.007213959
ENSMUSG00000031749	St3gal2	0.997886176	1.10823E-66
ENSMUSG00000046318	Ccbe1	0.996510549	3.34018E-20
ENSMUSG00000008540	Mgst1	0.994703796	1.42977E-94
ENSMUSG00000042997	Nhlrc3	0.9942618	4.57916E-51
ENSMUSG00000047810	Ccdc88b	0.994082954	1.88354E-05
ENSMUSG00000025746	Il6	0.989180405	3.1596E-133
ENSMUSG00000060568	Fam78b	0.987997581	1.48562E-32
ENSMUSG00000057933	Gsta2	0.987541938	0.027456718
ENSMUSG00000096929	A330023F24Rik	0.984506996	0.000258907
ENSMUSG00000021123	Rdh12	0.984112512	0.013190867
ENSMUSG00000079215	Zfp664	0.982846576	7.80413E-81
ENSMUSG00000032625	Thsd7a	0.982601541	1.44991E-51
ENSMUSG00000045392	Olfr1033	0.980686167	1.4369E-05
ENSMUSG00000031906	Smpd3	0.98005965	9.3161E-35
ENSMUSG00000075254	Heg1	0.979436591	3.6071E-197
ENSMUSG00000103313	Gm38357	0.978118491	3.47786E-05
ENSMUSG00000048826	Dact2	0.977547014	0.010346829
ENSMUSG00000051951	Xkr4	0.97292457	0.001078076
ENSMUSG00000037747	Phyhipl	0.972427208	1.65858E-05
ENSMUSG00000021567	Nkd2	0.972181244	1.2355E-130
ENSMUSG00000104263	9430062P05Rik	0.969734179	0.003375254
ENSMUSG00000024600	Slc27a6	0.9692545	0.001013079
ENSMUSG00000107603	Gm43921	0.967665965	0.026670038
ENSMUSG00000029380	Cxcl1	0.967124287	2.5856E-140
ENSMUSG00000052428	Tmco1	0.965468891	1.66759E-81
ENSMUSG00000118383	Gm50321	0.964085023	1.49762E-15
ENSMUSG00000025037	Maoa	0.963795742	2.5245E-179
ENSMUSG00000097057	Gm17638	0.963483419	0.01820095
ENSMUSG00000039252	Lgi2	0.960600451	7.04818E-28
ENSMUSG00000037622	Wdtc1	0.960478264	1.0982E-31
ENSMUSG00000038963	Slco4a1	0.95852357	1.98377E-27
ENSMUSG00000023991	Foxp4	0.95515667	3.52419E-28
ENSMUSG00000029844	Hoxa1	0.954537184	0.003670178
ENSMUSG00000026043	Col3a1	0.953623265	0
ENSMUSG00000003534	Ddr1	0.952920956	5.8631E-164
ENSMUSG00000032265	Tent5a	1.395580292	2.8721E-221
ENSMUSG00000092341	Malat1	1.392450627	0.038453932
ENSMUSG00000072949	Acot1	1.392257158	1.88694E-10
ENSMUSG00000032688	Malt1	1.389710914	1.3056E-99
ENSMUSG00000037362	Ccn3	1.388141367	4.49311E-96
ENSMUSG00000069727	Zfp975	1.388040099	2.77836E-15
ENSMUSG00000025921	Rdh10	1.386961898	4.15068E-55
ENSMUSG00000019775	Rgs17	1.386205693	1.05278E-33
ENSMUSG00000050896	Rtn4rl2	1.385915497	5.06067E-14
ENSMUSG00000028420	Tmem38b	1.382549757	3.9364E-135
ENSMUSG00000050994	Adgb	1.378731358	1.51827E-05
ENSMUSG00000040569	Slc26a7	1.37542083	0.028183219
ENSMUSG00000056771	Gm10010	1.375237089	0.033252723
ENSMUSG00000085666	Tdg-ps2	1.374614496	0.002156677
ENSMUSG00000039616	Mocos	1.373383054	4.73857E-55
ENSMUSG00000042647	Acad12	1.372364717	3.90042E-14
ENSMUSG00000025128	Bhlhe22	1.368331389	0.016017782
ENSMUSG00000055430	Nap1l5	1.36622794	1.01698E-12
ENSMUSG00000025911	Adhfe1	1.36428567	1.5515E-23
ENSMUSG00000078566	Bnip3	1.363493681	1.1883E-92
ENSMUSG00000030443	Zfp583	1.363366701	7.06361E-08
ENSMUSG00000006517	Mvd	1.359228133	5.4929E-53
ENSMUSG00000024360	Etf1	1.35465692	0
ENSMUSG00000107879	9330102E08Rik	1.354337606	1.80632E-07
ENSMUSG00000031377	Bmx	1.353888867	2.0796E-21
ENSMUSG00000029727	Cyp3a13	1.350689285	0.040964357
ENSMUSG00000020806	Rhbdf2	1.348000251	3.1168E-169
ENSMUSG00000040183	Ankrd6	1.344001391	2.0528E-16
ENSMUSG00000078838	Gm17382	1.341104974	0.000655986
ENSMUSG00000033213	AA467197	1.340529147	1.37563E-15
ENSMUSG00000015134	Aldh1a3	1.340032835	9.80346E-67
ENSMUSG00000028670	Lypla2	1.339899668	5.5078E-148
ENSMUSG00000097610	A930012L18Rik	1.33022514	0.024892944
ENSMUSG00000026473	Glul	1.328158612	6.8691E-243
ENSMUSG00000097462	9530026P05Rik	1.326887972	0.024103152
ENSMUSG00000024381	Bin1	1.324494043	7.2837E-209
ENSMUSG00000098009	Gm5597	1.323122521	0.004391528
ENSMUSG00000096929	A330023F24Rik	1.320296762	2.08083E-09
ENSMUSG00000060240	Cend1	1.320250759	0.048251087
ENSMUSG00000021098	4930447C04Rik	1.319973711	0.000117699
ENSMUSG00000020407	Upp1	1.309226408	1.55248E-15
ENSMUSG00000019139	Isyna1	1.307484564	2.23415E-93
ENSMUSG00000020901	Pik3r5	1.306466036	1.50914E-30
ENSMUSG00000050989	Selenon	1.306345666	2.7694E-268
ENSMUSG00000097804	Gm16685	1.306059076	0.000368573
ENSMUSG00000006262	Mob1b	1.305367332	4.1689E-222
ENSMUSG00000117628	Gm50012	1.29906543	2.6224E-13
ENSMUSG00000020121	Srgap1	1.299054406	2.0922E-106
ENSMUSG00000026202	Tuba4a	1.298695501	1.8625E-180
ENSMUSG00000028803	Nipal3	1.29771771	1.83046E-13
ENSMUSG00000107810	Gm18609	1.297656561	0.000103332
ENSMUSG00000097027	Gm26559	1.297374309	0.002056249
ENSMUSG00000022790	Igsf11	1.296823096	2.06627E-17
ENSMUSG00000000416	Cttnbp2	1.29645452	0.032395886
ENSMUSG00000031438	Rnf128	1.295488278	6.62283E-73
ENSMUSG00000022323	Rida	1.293754076	5.34206E-38
ENSMUSG00000080862	Gm14523	1.290296339	0.03033961
ENSMUSG00000019102	Aldh3a1	1.28861254	9.00085E-20
ENSMUSG00000116439	Gm49528	1.283704922	0.000692864
ENSMUSG00000106933	Gm43621	1.280588595	0.042537424
ENSMUSG00000029134	Plb1	1.278379344	0.043591278
ENSMUSG00000022456	Septin3	1.277613642	1.49101E-16
ENSMUSG00000056671	Prelid2	1.275468019	9.82286E-09
ENSMUSG00000032419	Tbx18	1.275447825	1.9065E-134
ENSMUSG00000039395	Mreg	1.273584686	1.5771E-06
ENSMUSG00000069540	Gm4925	1.272601638	0.005483594
ENSMUSG00000061118	Dnajc30	1.272095167	3.82022E-24
ENSMUSG00000054280	Prr14l	1.270675347	4.41799E-96
ENSMUSG00000038415	Foxq1	1.270415191	0.010784301
ENSMUSG00000058624	Gda	1.267886899	9.9112E-153
ENSMUSG00000042616	Oscp1	1.267307816	2.5808E-09
ENSMUSG00000041439	Mfsd6	1.258002203	7.88742E-60
ENSMUSG00000030203	Dusp16	1.253680334	1.8836E-138
ENSMUSG00000028751	Pla2g2e	1.251936036	8.28354E-07
ENSMUSG00000039865	Slc44a3	1.25073061	0.000299217
ENSMUSG00000020277	Pfkl	1.250353972	2.6528E-216
ENSMUSG00000101037	Gm28424	1.250316492	0.000346903

ENSMUSG00000047187	Rab2a	0.951530737	4.2689E-216	ENSMUSG00000104667	Gm4961	1.249812505	3.46067E-09
ENSMUSG00000044471	Lncpint	0.950819584	0.003202492	ENSMUSG00000031266	Gla	1.24909481	1.3897E-177
ENSMUSG00000061118	Dnajc30	0.949863745	5.58552E-11	ENSMUSG00000022223	Sdr39u1	1.249082971	2.07634E-07
ENSMUSG00000030725	Lipt2	0.948250092	0.000419887	ENSMUSG00000115338	Pnp	1.248327909	3.9464E-198
ENSMUSG00000052102	Gnpda1	0.947935492	1.82757E-90	ENSMUSG00000044026	Slc35g1	1.24692768	3.1081E-20
ENSMUSG00000022797	Tfrc	0.947095229	8.4902E-123	ENSMUSG00000047187	Rab2a	1.24481556	0
ENSMUSG00000025373	Rnf41	0.946114966	2.14256E-72	ENSMUSG00000102117	Rpsa-ps1	1.244365562	0.049325096
ENSMUSG00000110605	Gm32856	0.945916949	6.65863E-05	ENSMUSG00000096965	3300005D01Rik	1.242593453	9.48109E-87
ENSMUSG00000022844	Pdia5	0.945006897	1.60017E-91	ENSMUSG00000074384	AI429214	1.24234197	3.95522E-19
ENSMUSG00000039646	Vasn	0.940862872	8.1221E-135	ENSMUSG00000028072	Ntrk1	1.238706347	0.003948353
ENSMUSG00000029577	Ube3b	0.940626803	1.16746E-65	ENSMUSG00000026360	Rgs2	1.23706754	5.69295E-37
ENSMUSG00000033361	Prrg3	0.937470282	1.68089E-43	ENSMUSG00000027831	Veph1	1.233536814	6.30963E-06
ENSMUSG00000063275	Hacd1	0.936609194	6.58927E-36	ENSMUSG00000047446	Arl4a	1.233397791	2.8275E-30
ENSMUSG00000032216	Nedd4	0.935645437	6.9377E-277	ENSMUSG00000050697	Prkaa1	1.233382822	1.1932E-130
ENSMUSG00000026525	Opn3	0.935243294	0.000246437	ENSMUSG00000030560	Ctsc	1.233182076	1.93864E-50
ENSMUSG00000035107	Dcbld2	0.932462427	1.0727E-143	ENSMUSG00000104728	Gm42462	1.231912928	0.001804467
ENSMUSG00000034612	Chst11	0.931010249	2.3007E-200	ENSMUSG00000028402	Mpdz	1.230700794	1.0309E-272
ENSMUSG00000031520	Vegfc	0.930190334	1.57568E-49	ENSMUSG00000037185	Krt80	1.229305738	4.09922E-20
ENSMUSG00000100658	F730311O21Rik	0.930097907	2.18544E-06	ENSMUSG00000053117	E330013P04Rik	1.228846131	0.0163997
ENSMUSG00000002289	Angptl4	0.928879407	3.3162E-250	ENSMUSG00000057522	Spop	1.225753535	1.2893E-224
ENSMUSG00000021420	Fars2	0.927576949	7.52423E-13	ENSMUSG00000112963	Gm6093	1.224050665	1.36348E-09
ENSMUSG00000098022	Zfp82	0.926812761	0.00017198	ENSMUSG00000015533	Itga2	1.223764523	6.39469E-41
ENSMUSG00000037636	Slc25a43	0.921087732	1.08829E-13	ENSMUSG00000068893	Sprr2a2	1.222904886	0.002075017
ENSMUSG00000016024	Lbp	0.920503981	8.88479E-21	ENSMUSG00000030946	Lhpp	1.222856002	1.76297E-18
ENSMUSG00000040867	Begain	0.920431281	0.003247749	ENSMUSG00000032776	Mctp2	1.222712993	1.17427E-08
ENSMUSG00000015957	Wnt11	0.919978613	3.98603E-17	ENSMUSG00000117278	Gm36684	1.221863352	0.036222919
ENSMUSG00000028033	Kcnq5	0.919971791	3.10896E-16	ENSMUSG00000034762	Glis1	1.221209313	0.011205209
ENSMUSG00000116802	Gm5165	0.918052223	0.000111584	ENSMUSG00000025964	Adam23	1.220935151	4.37507E-16
ENSMUSG00000108368	Gm45053	0.916642594	0.018467261	ENSMUSG00000045414	Dipk2a	1.219562725	1.0311E-102
ENSMUSG00000050395	Tnfsf15	0.915468727	3.73199E-79	ENSMUSG00000052430	Bmpr1b	1.21369613	7.15506E-09
ENSMUSG00000032375	Aph1b	0.914502994	6.2837E-11	ENSMUSG00000022372	Sla	1.210857313	0.049645208
ENSMUSG00000037035	Inhbb	0.911330227	1.12984E-53	ENSMUSG00000023473	Celsr3	1.2097198	1.92234E-08
ENSMUSG00000047632	Fgfbp3	0.909124334	1.00415E-05	ENSMUSG00000089698	Gm2541	1.208434427	0.001035983
ENSMUSG00000001436	Slc19a1	0.909078736	2.85281E-10	ENSMUSG00000059182	Skap2	1.206700423	3.31151E-86
ENSMUSG00000031558	Slit2	0.908550408	1.99163E-71	ENSMUSG00000040855	Reps2	1.203491237	1.91877E-07
ENSMUSG00000020227	Irak3	0.905823421	1.22076E-15	ENSMUSG00000048376	F2r	1.202914507	1.2481E-296
ENSMUSG00000091780	Sco2	0.905476385	4.74332E-07	ENSMUSG00000073565	Prr16	1.202283162	2.30165E-06
ENSMUSG00000038256	Bcl9	0.905429899	1.26432E-25	ENSMUSG00000048240	Gng7	1.201304278	1.05831E-05
ENSMUSG00000036368	Rmdn2	0.900396106	7.958E-30	ENSMUSG00000038217	Tlcd2	1.198950814	1.07025E-49
ENSMUSG00000024261	Syt4	0.898802769	0.022843863	ENSMUSG00000039089	L3mbtl3	1.196043821	4.4211E-110
ENSMUSG00000072294	Klf12	0.8986231	3.85897E-08	ENSMUSG00000015536	Mocs2	1.195235932	5.3797E-93
ENSMUSG00000040714	Klc3	0.89824376	4.18367E-07	ENSMUSG00000046056	Sbsn	1.187257316	1.85008E-05
ENSMUSG00000058248	Kcnh1	0.897972014	0.040387889	ENSMUSG00000090124	Ugt1a7c	1.185366689	8.51386E-58
ENSMUSG00000027737	Slc7a11	0.896787454	1.81243E-56	ENSMUSG00000025194	Abcc2	1.185181947	0.00588653
ENSMUSG00000047562	Mmp10	0.896391621	5.24435E-09	ENSMUSG00000047731	Wbp1l	1.184194585	3.522E-265
ENSMUSG00000052392	Acot4	0.896155788	0.019995428	ENSMUSG00000040896	Kcnd3	1.18394062	0.035495198
ENSMUSG00000087672	Gm15122	0.887022732	0.045748615	ENSMUSG00000001300	Efnb2	1.183727119	6.6044E-157
ENSMUSG00000049580	Tsku	0.88679721	1.2148E-159	ENSMUSG00000092200	Tnxa	1.176014499	0.004596172
ENSMUSG00000020866	Cacna1g	0.885348291	0.008632028	ENSMUSG00000044854	1700056E22Rik	1.17585023	0.001145878
ENSMUSG00000026202	Tuba4a	0.884905848	1.18076E-80	ENSMUSG00000110344	Gm45716	1.175819454	1.16086E-05
ENSMUSG00000073000	Gm10451	0.883740555	1.30669E-05	ENSMUSG00000031765	Mt1	1.173794092	3.6258E-162
ENSMUSG00000022519	Srl	0.883667941	0.001583407	ENSMUSG00000029136	Rbks	1.171585476	8.78102E-07
ENSMUSG00000056457	Prl2c3	0.882890369	3.8295E-17	ENSMUSG00000027678	Ncoa3	1.171351985	7.9911E-116
ENSMUSG00000073234	Gm8773	0.882577406	1.87616E-06	ENSMUSG00000097166	9330179D12Rik	1.171338352	0.028326481
ENSMUSG00000030110	Ret	0.882423334	2.57505E-11	ENSMUSG00000070871	Ccnyl1	1.169235997	5.857E-266
ENSMUSG00000070323	Mmp27	0.881407018	4.12713E-09	ENSMUSG00000068874	Selenbp1	1.169069352	7.97826E-46
ENSMUSG00000095990	Zfp97	0.880394408	2.5044E-08	ENSMUSG00000039958	Etfbkmt	1.166147018	7.91575E-13
ENSMUSG00000041052	Slc7a13	0.877151944	0.014739431	ENSMUSG00000034220	Gpc1	1.164996169	4.50533E-79
ENSMUSG00000062040	Zfp27	0.877069994	1.12537E-12	ENSMUSG00000033361	Prrg3	1.164267831	1.09438E-76
ENSMUSG00000055150	Zfp78	0.875670389	0.004257612	ENSMUSG00000031879	Ciao2b	1.164057046	2.87527E-36
ENSMUSG00000022425	Enpp2	0.87449874	5.015E-05	ENSMUSG00000041695	Kcnj2	1.159272075	6.00611E-13
ENSMUSG00000108461	AV356131	0.873525262	0.034670829	ENSMUSG00000019936	Epyc	1.158501261	0.000292303
ENSMUSG00000041891	Lman1	0.871750272	4.1081E-140	ENSMUSG00000019850	Tnfaip3	1.158284059	7.89445E-73
ENSMUSG00000005087	Cd44	0.87084233	1.3235E-267	ENSMUSG00000028278	Rragd	1.155005005	2.01898E-67
ENSMUSG00000087590	Epb41l4aos	0.869486453	0.000462427	ENSMUSG00000047022	Mipol1	1.153971305	1.74887E-09
ENSMUSG00000107402	4732416N19Rik	0.869407009	0.040902602	ENSMUSG00000042581	Thsd7b	1.15393294	2.91493E-08
ENSMUSG00000024327	Slc39a7	0.869011184	1.8285E-05	ENSMUSG00000036478	Btg1	1.151753609	9.4988E-155
ENSMUSG00000048264	Dip2c	0.86872962	3.21451E-59	ENSMUSG00000021069	Pygl	1.151384698	2.10239E-24
ENSMUSG00000028369	Svep1	0.868345445	2.4199E-114	ENSMUSG00000064267	Hvcn1	1.148407323	3.71891E-58
ENSMUSG00000079138	Gm8818	0.867898723	0.016415055	ENSMUSG00000029577	Ube3b	1.146972245	5.9196E-127
ENSMUSG00000001300	Efnb2	0.867438715	3.9494E-74	ENSMUSG00000078664	Sprr2a1	1.14653927	0.007557612
ENSMUSG00000003617	Cp	0.864041241	7.3351E-190	ENSMUSG00000044229	Nxpe4	1.145595823	1.83147E-38
ENSMUSG00000089756	Zfp966	0.863950197	0.009410646	ENSMUSG00000015869	Prpsap1	1.145000706	4.9044E-114
ENSMUSG00000036432	Siah2	0.862343155	1.72271E-16	ENSMUSG00000046589	Lrrc8e	1.144640092	6.87141E-06
ENSMUSG00000040006	Ginm1	0.862310798	4.67553E-63	ENSMUSG00000028637	Ccdc30	1.142414035	0.001347115
ENSMUSG00000026177	Slc11a1	0.861378738	0.009311852	ENSMUSG00000096606	Tpbgl	1.142032262	0.000442633
ENSMUSG00000032115	Hyou1	0.860968046	1.4704E-113	ENSMUSG00000039633	Lonrf1	1.140725165	1.20134E-10

ENSMUSG00000026189	Pecr	0.859114251	9.2105E-07	ENSMUSG00000062380	Tubb3	1.137893388	1.0538E-188
ENSMUSG00000057359	Gm17494	0.858841897	1.61304E-06	ENSMUSG00000059439	Bcas3	1.137841683	3.96929E-60
ENSMUSG00000020806	Rhbdf2	0.856267393	9.92443E-61	ENSMUSG00000027287	Snap23	1.137434496	1.3732E-157
ENSMUSG00000114210	A330084C13Rik	0.856108967	0.001410269	ENSMUSG00000001910	Nacc1	1.136467662	8.2137E-167
ENSMUSG00000060733	Ipmk	0.854154359	3.25969E-34	ENSMUSG00000024533	Spire1	1.136294225	1.62281E-58
ENSMUSG00000028600	Podn	0.854048654	0.00248937	ENSMUSG00000031379	Pir	1.135502058	1.37172E-52
ENSMUSG00000072774	Zfp951	0.853574581	2.3593E-05	ENSMUSG00000039031	Arhgap18	1.132806588	1.22602E-64
ENSMUSG00000098050	Gm5345	0.85162955	1.03125E-10	ENSMUSG00000026939	Tmem141	1.131834603	5.80145E-14
ENSMUSG00000072761	Gm6712	0.851394762	0.042320574	ENSMUSG00000037104	Socs5	1.129659364	2.1718E-214
ENSMUSG00000097397	Gm16861	0.851319442	0.047882097	ENSMUSG00000039629	Strip2	1.128910227	2.39408E-08
ENSMUSG00000052957	Gas1	0.849988794	7.53895E-21	ENSMUSG00000050050	Ccdc158	1.128419447	0.047277742
ENSMUSG00000034674	Tdg	0.848267571	2.63411E-90	ENSMUSG00000062168	Ppef1	1.128340447	1.21137E-05
ENSMUSG00000018068	Ints2	0.847802835	3.57638E-41	ENSMUSG00000106121	Gm42679	1.128030343	0.000182135
ENSMUSG00000096696	Zfp960	0.847658292	2.53348E-06	ENSMUSG00000040907	Atp1a3	1.127807582	0.039515326
ENSMUSG00000055912	Tmem150a	0.846583215	7.35775E-50	ENSMUSG00000041112	Elmo1	1.125075988	4.82233E-43
ENSMUSG00000052727	Map1b	0.845408261	2.00683E-33	ENSMUSG00000024066	Xdh	1.1243415	4.7134E-184
ENSMUSG00000022376	Adcy8	0.844451603	0.001360475	ENSMUSG00000036782	Klhl13	1.123738702	4.5136E-189
ENSMUSG00000025731	Mettl26	0.843590544	0.000241833	ENSMUSG00000031963	Bmper	1.123158201	1.9151E-169
ENSMUSG00000028883	Sema3a	0.842542126	2.60816E-05	ENSMUSG00000086728	Man2c1os	1.121195921	0.032133082
ENSMUSG00000004098	Col5a3	0.842238572	5.11326E-44	ENSMUSG00000047260	Emc6	1.120188155	8.94386E-70
ENSMUSG00000039740	Alg2	0.842217088	3.8616E-46	ENSMUSG00000039956	Mrap	1.119445406	1.09249E-14
ENSMUSG00000001482	Def8	0.842020942	8.62716E-34	ENSMUSG00000021271	Zfp839	1.117967054	5.84899E-12
ENSMUSG00000094707	A830019P07Rik	0.84042594	0.047375603	ENSMUSG00000074521	Gm14327	1.115079862	5.85873E-09
ENSMUSG00000048076	Arf1	0.837875912	7.0817E-159	ENSMUSG00000031729	Ist1	1.114944903	2.7004E-147
ENSMUSG00000028664	Ephb2	0.837189649	4.32737E-25	ENSMUSG00000074796	Slc4a11	1.113107151	4.96727E-06
ENSMUSG00000017607	Tns4	0.836414968	1.39399E-11	ENSMUSG00000027224	Duoxa1	1.112872665	0.000764899
ENSMUSG00000116656	Gm49708	0.83547488	4.51221E-05	ENSMUSG00000025591	Tma16	1.11224208	5.99593E-48
ENSMUSG00000097068	Gm26760	0.833475224	0.01332716	ENSMUSG00000021508	Cxcl14	1.110458632	5.9111E-18
ENSMUSG00000052396	Mogat2	0.832944689	0.000406956	ENSMUSG00000025373	Rnf41	1.110359748	6.2759E-113
ENSMUSG00000002908	Kcnn1	0.831651908	0.019270265	ENSMUSG00000024621	Csf1r	1.109851745	6.77697E-32
ENSMUSG00000048240	Gng7	0.831599404	0.009299113	ENSMUSG00000041132	N4bp2l1	1.108041362	0.001804467
ENSMUSG00000016918	Sulf1	0.831518532	3.2464E-254	ENSMUSG00000028179	Cth	1.107122655	1.15101E-08
ENSMUSG00000063415	Cyp26b1	0.831089973	1.27967E-34	ENSMUSG00000032350	Gclc	1.106494537	1.5436E-241
ENSMUSG00000031266	Gla	0.830777775	9.33614E-64	ENSMUSG00000021567	Nkd2	1.104900785	2.3062E-106
ENSMUSG00000057969	Sema3b	0.829024853	2.68086E-09	ENSMUSG00000049422	Chchd10	1.104058213	0.000101994
ENSMUSG00000037254	Itih2	0.828955064	7.61902E-11	ENSMUSG00000038518	Jarid2	1.103670581	1.71138E-24
ENSMUSG00000110040	Gm49369	0.828276981	0.001171236	ENSMUSG00000002308	Cd320	1.101403218	2.27022E-27
ENSMUSG00000071266	Zfp946	0.826890714	9.25188E-09	ENSMUSG00000035107	Dcbld2	1.099269888	9.5135E-189
ENSMUSG00000030980	Knop1	0.826759857	1.78389E-95	ENSMUSG00000079677	Fdx2	1.097668953	0.022050999
ENSMUSG00000035769	Xylb	0.82501119	1.2147E-06	ENSMUSG00000090083	Rnf8	1.097485506	4.06569E-13
ENSMUSG00000024778	Fas	0.824881444	9.9215E-53	ENSMUSG00000033066	Gas7	1.094502826	2.1882E-135
ENSMUSG00000108228	6430584L05Rik	0.823826549	0.000874338	ENSMUSG00000034993	Vat1	1.094156455	0
ENSMUSG00000020092	Pald1	0.823264335	1.25071E-28	ENSMUSG00000106874	Gm20186	1.093929972	7.60461E-27
ENSMUSG00000031548	Sfrp1	0.823117077	9.7865E-201	ENSMUSG00000027674	Pex5l	1.093121362	0.019124479
ENSMUSG00000117628	Gm50012	0.822485583	4.47684E-05	ENSMUSG00000025478	Dpysl4	1.093028388	2.69802E-05
ENSMUSG00000035595	Fam174c	0.821647681	2.03348E-08	ENSMUSG00000045917	Tmem268	1.092614752	1.44467E-20
ENSMUSG00000020152	Actr2	0.818936993	2.4468E-176	ENSMUSG00000002475	Abhd3	1.092502428	0.000154224
ENSMUSG00000028179	Cth	0.818363517	0.000496374	ENSMUSG00000029153	Ociad2	1.092383013	4.64034E-17
ENSMUSG00000036412	Arsi	0.817939319	1.97525E-07	ENSMUSG00000031343	Gabra3	1.091870654	8.57564E-17
ENSMUSG00000022323	Rida	0.817883082	1.13418E-12	ENSMUSG00000026822	Lcn2	1.090183044	1.38394E-34
ENSMUSG00000023018	Smarcd1	0.816121074	4.837E-26	ENSMUSG00000079293	Clec7a	1.090022285	8.79474E-10
ENSMUSG00000040280	Ndufa4l2	0.81595093	8.50191E-06	ENSMUSG00000027792	Bche	1.086769827	9.55939E-12
ENSMUSG00000045045	Lrfn4	0.815701654	9.84557E-43	ENSMUSG00000093989	Rnasek	1.086678489	1.10919E-14
ENSMUSG00000052566	Hook2	0.815117529	5.06946E-07	ENSMUSG00000030259	Rassf8	1.085098695	1.5752E-208
ENSMUSG00000082475	Gm7206	0.814264547	0.029273212	ENSMUSG00000020078	Vps26a	1.083255245	9.7674E-181
ENSMUSG00000116673	A630089N07Rik	0.813769153	0.000514992	ENSMUSG00000018199	Ro60	1.08215193	2.2158E-142
ENSMUSG00000026042	Col5a2	0.813696301	0	ENSMUSG00000027371	Fahd2a	1.081903911	1.45243E-08
ENSMUSG00000020901	Pik3r5	0.81197247	1.04403E-12	ENSMUSG00000117959	D330050I16Rik	1.081301378	0.016907432
ENSMUSG00000062382	Ftl1-ps1	0.810446029	3.0773E-30	ENSMUSG00000091243	Vgll3	1.080808833	8.5481E-196
ENSMUSG00000022634	Yaf2	0.810063879	1.99348E-50	ENSMUSG00000022621	Rabl2	1.078872761	2.92712E-14
ENSMUSG00000087484	2900089D17Rik	0.809406987	0.000241563	ENSMUSG00000031604	Msmo1	1.076494489	2.0205E-140
ENSMUSG00000020950	Foxg1	0.807026111	5.69698E-08	ENSMUSG00000028341	Nr4a3	1.07500563	8.49687E-35
ENSMUSG00000066637	Ttc32	0.80695418	0.000290281	ENSMUSG00000004837	Grap	1.074037968	2.88871E-18
ENSMUSG00000039395	Mreg	0.806007548	0.016018728	ENSMUSG00000029377	Ereg	1.073357524	0
ENSMUSG00000028402	Mpdz	0.804983352	1.24802E-73	ENSMUSG00000028691	Prdx1	1.072725712	0
ENSMUSG00000019832	Rab32	0.804656078	1.26081E-31	ENSMUSG00000050737	Ptges	1.072028442	1.9658E-12
ENSMUSG00000049420	Tmem200a	0.804291683	2.67241E-17	ENSMUSG00000039062	Anpep	1.071049314	1.0924E-99
ENSMUSG00000020256	Aldh1l2	0.803892077	2.20879E-28	ENSMUSG00000054203	Ifi205	1.069856132	1.00863E-05
ENSMUSG00000003863	Ppfia3	0.803871825	0.037971793	ENSMUSG00000027797	Dclk1	1.066545063	3.2856E-234
ENSMUSG00000026028	Trak2	0.803203491	2.4169E-91	ENSMUSG00000071654	Uqcc3	1.066422604	9.51894E-10
ENSMUSG00000026939	Tmem141	0.802861652	2.73123E-06	ENSMUSG00000028124	Gclm	1.062946841	3.1168E-169
ENSMUSG00000046441	Cmtr2	0.801987168	3.10632E-12	ENSMUSG00000042717	Ppp1r3a	1.062602562	8.99436E-06
ENSMUSG00000066838	Zfp772	0.800402046	0.000332274	ENSMUSG00000021213	Akr1c13	1.062539279	1.05167E-15
ENSMUSG00000003458	Ncstn	0.800250862	1.38E-118	ENSMUSG00000019528	Gyg	1.060164213	1.6064E-164
ENSMUSG00000085457	1110046J04Rik	0.800012463	3.59051E-05	ENSMUSG00000027611	Procr	1.058979103	3.59537E-55
ENSMUSG00000046138	9930021J03Rik	0.799181523	9.95081E-39	ENSMUSG00000060147	Serpinb6a	1.058741911	6.4397E-255
ENSMUSG00000019850	Tnfaip3	0.798873036	1.53806E-37	ENSMUSG00000023013	Aqp2	1.056128863	3.93971E-05

ENSMUSG00000029223	Uchl1	0.798272088	1.99899E-48	ENSMUSG00000034300	Fam53c	1.056001684	1.3966E-117
ENSMUSG00000051736	Fam229b	0.797317348	0.011752064	ENSMUSG00000021614	Vcan	1.053537909	4.7657E-245
ENSMUSG00000029699	Ssc4d	0.796884772	0.000661841	ENSMUSG00000038679	Trps1	1.05305229	1.89E-87
ENSMUSG00000054893	Zfp667	0.796335978	0.000246913	ENSMUSG00000067365	Tmem128	1.052642842	2.87702E-87
ENSMUSG00000029426	Scarb2	0.795992848	1.111E-218	ENSMUSG00000039652	Cpeb3	1.052267028	8.75264E-24
ENSMUSG00000021614	Vcan	0.795680804	2.9914E-116	ENSMUSG00000027859	Ngf	1.052058854	0
ENSMUSG00000036019	Tmtc2	0.795166826	6.23051E-13	ENSMUSG00000028958	Tmub1	1.051477518	1.53311E-05
ENSMUSG00000003355	Fkbp11	0.794128785	1.03899E-25	ENSMUSG00000026289	Atg16l1	1.050561719	8.05223E-69
ENSMUSG00000032024	Clmp	0.793467678	8.27825E-23	ENSMUSG00000102691	Gm37780	1.04991149	0.018090663
ENSMUSG00000027859	Ngf	0.792652947	8.7349E-167	ENSMUSG00000036769	Wdr44	1.049798264	2.35711E-84
ENSMUSG00000047415	Gpr68	0.792466113	4.92916E-06	ENSMUSG00000027668	Mfn1	1.048848388	8.70243E-74
ENSMUSG00000016552	Foxred2	0.792353637	0.001702012	ENSMUSG00000028599	Tnfrsf1b	1.048445391	0
ENSMUSG00000070605	Zfp992	0.792129401	6.35701E-23	ENSMUSG00000087672	Gm15122	1.047825944	0.006972677
ENSMUSG00000028145	Them4	0.791191688	9.70338E-08	ENSMUSG00000048826	Dact2	1.046734687	0.000112909
ENSMUSG00000053626	Tll1	0.791144594	1.77647E-24	ENSMUSG00000041423	Paqr6	1.042063278	0.010251396
ENSMUSG00000035000	Dpp4	0.790118177	3.74744E-07	ENSMUSG00000038932	Tcfl5	1.040336472	0.004541542
ENSMUSG00000078630	Tomt	0.789525411	2.66722E-05	ENSMUSG00000097057	Gm17638	1.037837194	0.005120598
ENSMUSG00000039457	Ppl	0.787978775	7.12686E-23	ENSMUSG00000031380	Vegfd	1.037793168	3.6328E-103
ENSMUSG00000058388	Phtf1	0.787848852	3.81335E-36	ENSMUSG00000065336	Snora34	1.037114891	0.020861731
ENSMUSG00000002602	Axl	0.786962059	2.3204E-207	ENSMUSG00000040466	Blvrb	1.03704023	5.09203E-65
ENSMUSG00000041731	Pgm5	0.786304866	0.000530153	ENSMUSG00000032489	Kif9	1.03431672	0.000756292
ENSMUSG00000044231	Nhlrc1	0.785315727	0.007168117	ENSMUSG00000050010	Shisa3	1.033418738	0.003551428
ENSMUSG00000014776	Nol3	0.784295069	2.92052E-11	ENSMUSG00000073147	5031425E22Rik	1.033261041	1.56479E-35
ENSMUSG00000026558	Uck2	0.782851323	1.66921E-56	ENSMUSG00000040606	Kazn	1.032542988	1.43022E-39
ENSMUSG00000031963	Bmper	0.782740837	1.12904E-77	ENSMUSG00000042997	Nhlrc3	1.031371476	3.1864E-49
ENSMUSG00000041161	Otud3	0.78183172	2.15724E-14	ENSMUSG00000057173	Rfx8	1.031096366	0.045126484
ENSMUSG00000024593	Megf10	0.781249989	7.88423E-26	ENSMUSG00000001998	Ap4e1	1.030981405	2.09769E-57
ENSMUSG00000028164	Manba	0.781011453	9.102E-47	ENSMUSG00000021950	Anxa8	1.029415878	5.01039E-17
ENSMUSG00000102780	Gm38253	0.780071007	0.001383004	ENSMUSG00000041958	Pigs	1.027828286	1.70604E-70
ENSMUSG00000115248	Gm49037	0.779539431	5.56951E-11	ENSMUSG00000028351	Brinp1	1.027155396	8.70224E-06
ENSMUSG00000084862	Gm16278	0.7792787	0.001962472	ENSMUSG00000038324	Trpc4ap	1.0263326	5.8546E-182
ENSMUSG00000029161	Cgref1	0.778435558	1.4846E-06	ENSMUSG00000022144	Gdnf	1.025245844	3.48197E-17
ENSMUSG00000071656	Lrrn4cl	0.776992348	1.27002E-07	ENSMUSG00000044122	Proca1	1.023956245	0.011461867
ENSMUSG00000031608	Galnt7	0.775609177	7.71043E-31	ENSMUSG00000025934	Gsta3	1.023232642	1.6754E-53
ENSMUSG00000001627	Ifrd1	0.774459566	5.97709E-98	ENSMUSG00000031574	Star	1.02316594	1.58619E-18
ENSMUSG00000035235	Trim13	0.77269122	1.43943E-07	ENSMUSG00000096991	Gm26789	1.021116473	0.032731305
ENSMUSG00000080115	Eef1akmt3	0.772480598	0.000532786	ENSMUSG00000061436	Hipk2	1.01944456	5.73556E-09
ENSMUSG00000039497	Dse	0.772085237	1.95946E-96	ENSMUSG00000013593	Ndufs2	1.018952037	3.214E-204
ENSMUSG00000113836	Gm3325	0.771316353	0.001806137	ENSMUSG00000038725	Pkhd1l1	1.01393298	0.00016734
ENSMUSG00000102758	Naaladl2	0.768674717	1.22786E-06	ENSMUSG00000104917	Gm43289	1.013794031	0.015286335
ENSMUSG00000069727	Zfp975	0.767557209	0.000403743	ENSMUSG00000015837	Sqstm1	1.01368476	0
ENSMUSG00000023393	Slc17a9	0.766612117	2.67303E-07	ENSMUSG00000022999	Lmbr1l	1.0133834	1.55712E-33
ENSMUSG00000005225	Plekha8	0.765974515	2.24345E-20	ENSMUSG00000002489	Tiam1	1.011562365	3.27253E-49
ENSMUSG00000052151	Plpp2	0.765432628	8.06961E-82	ENSMUSG00000030861	Acadsb	1.010901851	2.2371E-205
ENSMUSG00000037169	Mycn	0.764731612	2.81624E-17	ENSMUSG00000048720	Tbc1d12	1.009564001	3.47606E-42
ENSMUSG00000026170	Cyp27a1	0.764327987	0.000472509	ENSMUSG00000034674	Tdg	1.009554633	2.9255E-129
ENSMUSG00000057286	St6galnac2	0.764326424	0.001211737	ENSMUSG00000031176	Dynlt3	1.008613617	1.9924E-196
ENSMUSG00000036676	Tmtc3	0.763831771	1.62549E-90	ENSMUSG00000020262	Adarb1	1.008413287	4.22227E-66
ENSMUSG00000039585	Myo9a	0.763798392	4.59321E-38	ENSMUSG00000028961	Pgd	1.007888612	3.459E-254
ENSMUSG00000091243	Vgll3	0.763033564	1.207E-130	ENSMUSG00000087119	Atg4a-ps	1.005353251	0.003348322
ENSMUSG00000030545	Pex11a	0.76112968	1.38855E-10	ENSMUSG00000011256	Adam19	0.999649013	1.6226E-264
ENSMUSG00000020407	Upp1	0.758562392	0.000130064	ENSMUSG00000063275	Hacd1	0.99954875	5.85472E-44
ENSMUSG00000031519	Asb5	0.758422126	3.8865E-120	ENSMUSG00000020077	Srgn	0.997129834	9.40834E-05
ENSMUSG00000074521	Gm14327	0.758372564	0.000370146	ENSMUSG00000032561	Acpp	0.993789738	0.010033534
ENSMUSG00000003665	Has1	0.758351346	1.25363E-06	ENSMUSG00000071253	Slc25a16	0.993004313	5.72438E-50
ENSMUSG00000005672	Kit	0.758309478	1.16551E-05	ENSMUSG00000031647	Mfap3l	0.989450844	1.636E-34
ENSMUSG00000027678	Ncoa3	0.75812492	1.59247E-28	ENSMUSG00000109379	Gm44550	0.986307077	0.004585462
ENSMUSG00000095193	Gm20939	0.757634155	0.017954063	ENSMUSG00000027303	Ptpra	0.986111486	4.0781E-138
ENSMUSG00000009731	Kcnd1	0.757458344	1.8671E-07	ENSMUSG00000070336	Fbxo47	0.985588425	0.019291211
ENSMUSG00000050697	Prkaa1	0.757271426	1.09797E-52	ENSMUSG00000110588	Gm45774	0.985347838	1.11759E-07
ENSMUSG00000043004	Gng2	0.756756342	4.18045E-16	ENSMUSG00000022707	Gbe1	0.985161304	7.63639E-76
ENSMUSG00000037411	Serpine1	0.756524704	2.1325E-205	ENSMUSG00000038375	Trp53inp2	0.983912445	0
ENSMUSG00000031875	Cmtm3	0.756521674	1.59552E-95	ENSMUSG00000008734	Gprc5b	0.983198694	5.3314E-264
ENSMUSG00000046719	Nxph3	0.756325528	0.010145938	ENSMUSG00000094651	Gal3st2	0.982460124	2.17331E-62
ENSMUSG00000081534	Slc48a1	0.75608308	5.7783E-68	ENSMUSG00000054065	Pkp3	0.980177761	0.00974111
ENSMUSG00000078894	2210418O10Rik	0.755810194	3.02881E-05	ENSMUSG00000047810	Ccdc88b	0.979927194	8.51584E-05
ENSMUSG00000038521	C1s1	0.755494179	6.60671E-24	ENSMUSG00000021460	Auh	0.979427697	5.87895E-38
ENSMUSG00000087260	Lamtor5	0.7553317	9.45794E-27	ENSMUSG00000020592	Sdc1	0.979049179	2.2768E-165
ENSMUSG00000031879	Ciao2b	0.755215812	1.96882E-14	ENSMUSG00000023046	Igfbp6	0.978817835	6.35014E-21
ENSMUSG00000089940	Gm4117	0.754818779	0.045936882	ENSMUSG00000084990	Gm14549	0.978773127	0.005433259
ENSMUSG00000038527	C1rl	0.75132155	1.92286E-19	ENSMUSG00000038028	Tigar	0.978354445	2.39654E-28
ENSMUSG00000024589	Nedd4l	0.750860799	6.52875E-89	ENSMUSG00000005054	Cstb	0.977948518	4.7164E-133
ENSMUSG00000082062	Ftl2-ps	0.750362352	4.77288E-06	ENSMUSG00000023143	Nagpa	0.977203072	5.8816E-64
ENSMUSG00000044026	Slc35g1	0.750190755	2.32204E-07	ENSMUSG00000061535	C1qtnf7	0.977158793	1.40203E-22
ENSMUSG00000028970	Abcb1b	0.74917565	2.05169E-59	ENSMUSG00000073234	Gm8773	0.977153652	1.32385E-07
ENSMUSG00000074867	Zfp808	0.748994932	0.010538395	ENSMUSG00000087249	Gm16062	0.976527056	0.000452294
ENSMUSG00000038028	Tigar	0.747982325	1.43014E-12	ENSMUSG00000114210	A330084C13Rik	0.975424221	8.83474E-05

ENSMUSG00000050533	Gm9845	0.747745707	0.045932973
ENSMUSG00000007655	Cav1	0.747473412	1.6549E-152
ENSMUSG00000071253	Slc25a16	0.746646142	1.03485E-26
ENSMUSG00000050708	Ftl1	0.746061588	7.2403E-105
ENSMUSG00000038503	Mesd	0.743977802	2.1774E-111
ENSMUSG00000027254	Map1a	0.743926781	4.43712E-38
ENSMUSG00000035021	Baz1a	0.743319933	4.4188E-47
ENSMUSG00000035900	Gramd4	0.742779417	1.48353E-55
ENSMUSG00000033405	Nudt15	0.742584871	4.27031E-05
ENSMUSG00000050855	Zfp940	0.742580418	0.015804189
ENSMUSG00000051391	Ywhag	0.740721032	4.4612E-140
ENSMUSG00000027668	Mfn1	0.739901054	3.43482E-36
ENSMUSG00000068699	Flnc	0.738933276	4.66225E-59
ENSMUSG00000029553	Tfec	0.738624058	5.67389E-38
ENSMUSG00000038508	Gdf15	0.738169128	1.43708E-10
ENSMUSG00000046610	Oacyl	0.737398035	2.32165E-05
ENSMUSG00000023046	Igfbp6	0.735948944	9.60437E-11
ENSMUSG00000096718	Zfp781	0.735435318	2.7212E-06
ENSMUSG00000020277	Pfkl	0.734386789	4.32538E-62
ENSMUSG00000023284	Zfp605	0.733914392	0.00183235
ENSMUSG00000114196	Gm47547	0.733760122	7.7884E-09
ENSMUSG00000029370	Rassf6	0.73318131	2.20641E-12
ENSMUSG00000054555	Adam12	0.733044413	1.09115E-58
ENSMUSG00000039158	Akna	0.732854533	4.77638E-14
ENSMUSG00000046230	Vps13a	0.7325712	3.07144E-22
ENSMUSG00000070509	Rgma	0.732409537	3.8221E-17
ENSMUSG00000040697	Dnajc16	0.731478858	1.01227E-28
ENSMUSG00000037820	Tgm2	0.728666221	6.54965E-46
ENSMUSG00000038342	Mlxip	0.728276155	2.88311E-64
ENSMUSG00000050737	Ptges	0.72795409	1.82621E-06
ENSMUSG00000038324	Trpc4ap	0.72716088	6.53458E-81
ENSMUSG00000031298	Adgrg2	0.726597284	6.91607E-33
ENSMUSG00000020262	Adarb1	0.72631352	3.05044E-28
ENSMUSG00000031824	6430548M08Rik	0.725127704	3.64762E-06
ENSMUSG00000042581	Thsd7b	0.724216579	0.000499959
ENSMUSG00000029552	Tes	0.724173409	9.88028E-46
ENSMUSG00000042784	Muc1	0.724049553	0.03742286
ENSMUSG00000079092	Prl2c2	0.723441519	1.33157E-27
ENSMUSG00000042759	Apobr	0.722656436	3.36238E-15
ENSMUSG00000062991	Nrg1	0.721950284	5.95933E-29
ENSMUSG00000036052	Dnajb5	0.721200989	6.08302E-11
ENSMUSG00000110630	K230015D01Rik	0.7208459	0.036561169
ENSMUSG00000028980	H6pd	0.719715541	3.6445E-141
ENSMUSG00000026012	Cd28	0.718807953	0.007558811
ENSMUSG00000023473	Celsr3	0.718576363	0.006011845
ENSMUSG00000085433	Gm16001	0.717240286	0.048991187
ENSMUSG00000051029	Serpinb1b	0.714238071	0.007331257
ENSMUSG00000060336	Zfp937	0.713095035	0.001677072
ENSMUSG00000039908	Slc26a11	0.709513537	3.04923E-13
ENSMUSG00000053604	Rpia	0.708736691	5.69446E-14
ENSMUSG00000024505	Dtwd2	0.707850463	0.008577235
ENSMUSG00000032419	Tbx18	0.707481279	1.70392E-39
ENSMUSG00000016382	Pls3	0.707090643	3.2583E-159
ENSMUSG00000015335	Zdhhc12	0.706982973	8.27388E-09
ENSMUSG00000103009	Gm4430	0.7063902	7.89241E-06
ENSMUSG00000039047	Pigk	0.704824374	2.1104E-68
ENSMUSG00000066798	Zbtb6	0.704750138	1.42315E-14
ENSMUSG00000016256	Ctsz	0.704087633	5.7748E-99
ENSMUSG00000072066	6720489N17Rik	0.70392254	0.000628424
ENSMUSG00000020250	Txnrd1	0.703865476	9.0744E-153
ENSMUSG00000027806	Tsc22d2	0.703865181	1.9403E-91
ENSMUSG00000105950	Gm43679	0.703822162	0.011492607
ENSMUSG00000034997	Htr2a	0.703799721	6.34193E-48
ENSMUSG00000028517	Plpp3	0.7031443	9.0366E-161
ENSMUSG00000078502	Gm13212	0.702788679	1.61027E-13
ENSMUSG00000031176	Dynlt3	0.702525487	2.40958E-83
ENSMUSG00000025921	Rdh10	0.701631713	9.11719E-16
ENSMUSG00000053641	Dennd4a	0.701355396	1.16252E-28
ENSMUSG00000031565	Fgfr1	0.701056527	6.9729E-153
ENSMUSG00000022148	Fyb	0.700719611	0.039507458
ENSMUSG00000115338	Pnp	0.699800489	5.25473E-48
ENSMUSG00000116812	Gm2792	0.699760663	3.17295E-10
ENSMUSG00000020176	Grb10	0.699649435	1.2396E-45
ENSMUSG00000039067	Psmd7	0.699552318	8.03056E-67
ENSMUSG00000038708	Golga4	0.698988886	6.80131E-45
ENSMUSG00000024381	Bin1	0.698638516	2.61025E-56
ENSMUSG00000025579	Gaa	0.697886242	7.31651E-79

ENSMUSG00000031927	1700012B09Rik	0.973366978	0.000907044
ENSMUSG00000025353	Ormdl2	0.972689548	5.04422E-24
ENSMUSG00000031520	Vegfc	0.970955441	1.3669E-57
ENSMUSG00000044367	Slc16a13	0.97018165	4.66087E-09
ENSMUSG00000038342	Mlxip	0.96770657	4.1411E-122
ENSMUSG00000044165	Bcl2l15	0.96767592	0.044444078
ENSMUSG00000005413	Hmox1	0.967036879	3.3524E-164
ENSMUSG00000005125	Ndrg1	0.965228309	3.80809E-31
ENSMUSG00000103292	Gm35048	0.964038759	0.028196907
ENSMUSG00000060961	Slc4a4	0.963571327	9.23415E-25
ENSMUSG00000078889	Gm14288	0.962385842	2.06454E-06
ENSMUSG00000028850	Gpatch3	0.962232729	1.77773E-17
ENSMUSG00000024646	Cyb5a	0.96069097	1.3966E-207
ENSMUSG00000052102	Gnpda1	0.960353779	3.85947E-93
ENSMUSG00000039428	Tmem135	0.959584214	1.11674E-74
ENSMUSG00000110393	Gm36445	0.959039864	0.041208693
ENSMUSG00000035762	Tmem161b	0.956199405	3.09245E-21
ENSMUSG00000027605	Acss2	0.955775922	2.07974E-38
ENSMUSG00000097888	Gm26682	0.955604511	0.004875415
ENSMUSG00000053897	Slc39a8	0.955588728	4.84032E-20
ENSMUSG00000107944	Gm44280	0.954247934	0.010425796
ENSMUSG00000091905	Dnajb6-ps	0.953834996	0.041065866
ENSMUSG00000019929	Dcn	0.952110062	0
ENSMUSG00000024593	Megf10	0.949122551	8.24218E-36
ENSMUSG00000026154	Sdhaf4	0.949011017	6.44636E-10
ENSMUSG00000040663	Clcf1	0.948836566	1.3463E-61
ENSMUSG00000060149	BC002059	0.948546207	6.32055E-07
ENSMUSG00000013539	Tango2	0.948358306	2.98531E-30
ENSMUSG00000029482	Aacs	0.94831654	1.5356E-117
ENSMUSG00000022450	Ndufa6	0.947650809	4.51035E-46
ENSMUSG00000113338	A530046M15Rik	0.947286135	0.002940597
ENSMUSG00000048264	Dip2c	0.946628552	1.82366E-84
ENSMUSG00000003559	As3mt	0.945642925	3.17658E-09
ENSMUSG00000109284	B230311B06Rik	0.945633071	0.031876586
ENSMUSG00000020044	Timp3	0.944475187	6.9507E-216
ENSMUSG00000106825	2510016D11Rik	0.944400744	0.001385776
ENSMUSG00000024104	Washc2	0.944094147	1.5148E-119
ENSMUSG00000019066	Rab3d	0.943784549	1.15915E-31
ENSMUSG00000046230	Vps13a	0.940945584	8.38046E-34
ENSMUSG00000043421	Hilpda	0.940292787	5.87053E-16
ENSMUSG00000027610	Gss	0.940272953	1.11054E-74
ENSMUSG00000053604	Rpia	0.939923357	1.20259E-26
ENSMUSG00000035227	Spcs2	0.938758254	1.0562E-161
ENSMUSG00000023931	Efhb	0.938317505	0.029218112
ENSMUSG00000055302	Mrfap1	0.935391731	2.9838E-201
ENSMUSG00000091780	Sco2	0.933416939	1.49824E-08
ENSMUSG00000110949	Nudt8	0.932972667	1.30229E-05
ENSMUSG00000026166	Ccl20	0.930437815	8.42078E-18
ENSMUSG00000079215	Zfp664	0.930004185	1.36421E-83
ENSMUSG00000083355	Gm11581	0.929923584	0.046866102
ENSMUSG00000061104	Sap18b	0.928645262	1.19727E-32
ENSMUSG00000048076	Arf1	0.92744084	3.1192E-181
ENSMUSG00000044952	Kctd21	0.92665416	8.32975E-17
ENSMUSG00000039782	Cpeb2	0.92608121	1.52667E-63
ENSMUSG00000037638	Zbtb42	0.925519688	7.37035E-07
ENSMUSG00000049580	Tsku	0.924708436	4.6313E-182
ENSMUSG00000027804	Ppid	0.924444901	3.04371E-95
ENSMUSG00000084968	Gm12743	0.923240983	0.015620779
ENSMUSG00000029209	Gnpda2	0.922066488	1.56865E-26
ENSMUSG00000026670	Uap1	0.922044116	1.1632E-106
ENSMUSG00000022094	Slc39a14	0.921734592	7.0719E-174
ENSMUSG00000031709	Tbc1d9	0.920766759	1.55538E-39
ENSMUSG00000041161	Otud3	0.920699075	5.46889E-21
ENSMUSG00000029361	Nos1	0.920287428	0.011444472
ENSMUSG00000021112	Mpp5	0.918536675	3.0358E-111
ENSMUSG00000025980	Hspd1	0.918400393	2.434E-126
ENSMUSG00000029456	Acad10	0.917849897	2.47976E-07
ENSMUSG00000047250	Ptgs1	0.916011528	1.50367E-60
ENSMUSG00000041229	Phf8	0.914186577	3.25998E-38
ENSMUSG00000025815	Dhtkd1	0.914162772	2.09503E-14
ENSMUSG00000039982	Dtx4	0.914060713	2.47375E-92
ENSMUSG00000039607	Rbms3	0.913737341	3.45531E-78
ENSMUSG00000042684	Npl	0.913692687	0.004991787
ENSMUSG00000030842	Lamtor1	0.911502213	5.5609E-100
ENSMUSG00000033491	Prss35	0.91050054	0.0321142
ENSMUSG00000052392	Acot4	0.910241739	0.013403407
ENSMUSG00000097842	9330104G04Rik	0.910067257	0.037477779

ENSMUSG00000040843	Tiprl	0.697160476	2.47232E-35	ENSMUSG00000034341	Wbp2	0.906687367	2.6575E-109
ENSMUSG00000042766	Trim46	0.696549945	3.82446E-06	ENSMUSG00000020607	Lratd1	0.903456074	0.003388419
ENSMUSG00000033488	Cryzl2	0.696539656	6.12343E-05	ENSMUSG00000092232	Gm20521	0.902212084	2.79062E-05
ENSMUSG00000056121	Fez2	0.695838235	3.86075E-52	ENSMUSG00000020826	Nos2	0.902138099	3.69673E-31
ENSMUSG00000116305	Lncppara	0.695084671	0.003509683	ENSMUSG00000037553	Zdhhc18	0.900648113	1.87091E-68
ENSMUSG00000061353	Cxcl12	0.694504682	9.54567E-95	ENSMUSG00000026558	Uck2	0.899143026	3.79932E-87
ENSMUSG00000039463	Slc9a8	0.693910911	2.18867E-34	ENSMUSG00000046169	Adamts6	0.899044776	5.83318E-16
ENSMUSG00000074519	Zfp971	0.69384366	6.21076E-06	ENSMUSG00000115423	AL731706.1	0.896663211	0.00023544
ENSMUSG00000025140	Pycr1	0.693225832	2.23946E-09	ENSMUSG00000058809	Hspd1-ps3	0.896501297	2.91711E-05
ENSMUSG00000078889	Gm14288	0.692510729	0.002403895	ENSMUSG00000022754	Tmem45a	0.895005632	1.5936E-235
ENSMUSG00000028670	Lypla2	0.692240511	2.0556E-31	ENSMUSG00000007038	Neu1	0.894173365	9.4843E-93
ENSMUSG00000045348	Nyap1	0.69181843	3.98028E-06	ENSMUSG00000068417	Pnp2	0.893164116	1.48535E-25
ENSMUSG00000040151	Hs2st1	0.691339774	4.11112E-43	ENSMUSG00000056185	Snx32	0.892623557	2.26946E-09
ENSMUSG00000041774	Ydjc	0.69097942	0.012794294	ENSMUSG00000024644	Cndp2	0.892467085	2.0383E-164
ENSMUSG00000044641	Pard6b	0.690959162	7.2969E-10	ENSMUSG00000053199	Arhgap20	0.892463311	1.1285E-10
ENSMUSG00000022197	Pdzd2	0.690705147	2.38784E-30	ENSMUSG00000027254	Map1a	0.892015786	3.76422E-78
ENSMUSG00000057605	Gm6807	0.690135411	0.007104133	ENSMUSG00000041235	Chd7	0.891903863	3.29715E-25
ENSMUSG00000011960	Ccnt1	0.69008846	9.91459E-51	ENSMUSG00000000340	Dbt	0.89174515	2.75878E-24
ENSMUSG00000042298	Ttc19	0.688293157	1.80034E-30	ENSMUSG00000029335	Bmp3	0.890970808	0.032509854
ENSMUSG00000042622	Maff	0.688097357	6.87421E-63	ENSMUSG00000028645	Slc2a1	0.890619596	1.2412E-141
ENSMUSG00000035561	Aldh1b1	0.687243567	0.014183404	ENSMUSG00000028780	Sema3c	0.889223823	8.14498E-31
ENSMUSG00000040701	Ap1g2	0.686514372	6.26339E-09	ENSMUSG00000020042	Btbd11	0.887993383	6.64514E-07
ENSMUSG00000028850	Gpatch3	0.684198512	2.49467E-08	ENSMUSG00000030284	Creld1	0.887639064	2.78889E-72
ENSMUSG00000104667	Gm4961	0.684163077	0.00702431	ENSMUSG00000015305	Sash1	0.887378999	1.9925E-246
ENSMUSG00000020105	Lrig3	0.683942456	3.57226E-07	ENSMUSG00000031441	Atp11a	0.887218893	1.4817E-148
ENSMUSG00000030268	Bcat1	0.683689634	2.5408E-21	ENSMUSG00000029596	Sdsl	0.885648036	5.20765E-13
ENSMUSG00000018659	Pnpo	0.683433354	4.34749E-06	ENSMUSG00000031558	Slit2	0.885519325	2.8096E-66
ENSMUSG00000028487	Bnc2	0.683168131	2.79463E-08	ENSMUSG00000021420	Fars2	0.885355152	8.56235E-12
ENSMUSG00000029009	Mthfr	0.683145508	2.02314E-58	ENSMUSG00000039067	Psmd7	0.884609531	1.4994E-137
ENSMUSG00000063687	Pcdhb5	0.683008179	0.013540622	ENSMUSG00000006818	Sod2	0.883859836	4.417E-130
ENSMUSG00000078862	Gm14326	0.681654626	3.1649E-06	ENSMUSG00000063273	Naa15	0.882572832	1.07861E-98
ENSMUSG00000023013	Aqp2	0.681580967	0.029218712	ENSMUSG00000097101	1810034E14Rik	0.882491962	0.000399208
ENSMUSG00000110105	Gm45844	0.681505909	0.049822841	ENSMUSG00000093805	Gal3st2b	0.882465661	3.15135E-25
ENSMUSG00000058093	Zfp729b	0.681148901	3.20093E-09	ENSMUSG00000002107	Celf2	0.88216494	5.27536E-97
ENSMUSG00000029174	Tbc1d1	0.680502878	6.91057E-51	ENSMUSG00000062554	Gm12751	0.881047772	7.30548E-05
ENSMUSG00000073678	Pgap1	0.679779585	1.52361E-07	ENSMUSG00000040446	Rprd1a	0.880145573	1.35157E-53
ENSMUSG00000005686	Ampd3	0.679137702	5.0135E-56	ENSMUSG00000058665	En1	0.878244563	0.003389607
ENSMUSG00000031150	Ccdc120	0.678993986	0.000899431	ENSMUSG00000029086	Prom1	0.878223707	0.006461032
ENSMUSG00000002228	Ppm1j	0.678372454	0.00892133	ENSMUSG00000021054	Sgpp1	0.878052066	2.1888E-108
ENSMUSG00000026796	Fam129b	0.678362965	1.4692E-154	ENSMUSG00000047497	Adamts12	0.877107382	1.32275E-24
ENSMUSG00000010663	Fads1	0.677405205	5.30066E-72	ENSMUSG00000014956	Ppp1cb	0.877080049	5.6492E-195
ENSMUSG00000021701	Plk2	0.677023999	1.02007E-92	ENSMUSG00000024042	Sik1	0.876878031	1.01177E-39
ENSMUSG00000048039	Isg20l2	0.676978274	3.32596E-35	ENSMUSG00000098188	Sowahc	0.876470437	1.10909E-18
ENSMUSG00000097412	1810014B01Rik	0.676020444	0.010188871	ENSMUSG00000020614	Fam20a	0.876022035	1.05193E-38
ENSMUSG00000110344	Gm45716	0.675693972	0.047614933	ENSMUSG00000026170	Cyp27a1	0.874575353	2.11038E-05
ENSMUSG00000024887	Asah2	0.675440188	1.78353E-21	ENSMUSG00000067121	Gm7027	0.874111619	0.045199898
ENSMUSG00000074634	Tmem267	0.67501657	1.25961E-08	ENSMUSG00000025888	Casp1	0.873354898	0.038114579
ENSMUSG00000024827	Gldc	0.673794099	5.30996E-10	ENSMUSG00000032024	Clmp	0.873029288	5.6922E-36
ENSMUSG00000035227	Spcs2	0.673155678	4.21509E-72	ENSMUSG00000024327	Slc39a7	0.872507067	1.23477E-05
ENSMUSG00000001555	Fkbp10	0.67241291	1.4852E-140	ENSMUSG00000022747	St3gal6	0.871588364	2.81987E-34
ENSMUSG00000032641	Gpr19	0.671828723	0.00052664	ENSMUSG00000030397	Mark4	0.86932172	7.91387E-60
ENSMUSG00000006221	Hspb7	0.671666915	8.0361E-36	ENSMUSG00000115151	Gm19276	0.869212121	0.038657215
ENSMUSG00000066880	Zfp617	0.670984767	8.661E-13	ENSMUSG00000046463	5930403N24Rik	0.868845078	0.000806733
ENSMUSG00000074283	Zfp109	0.670264058	0.001797727	ENSMUSG00000041135	Ripk2	0.868296864	2.2408E-168
ENSMUSG00000095648	Gm2004	0.670165268	0.030660769	ENSMUSG00000030037	Mrpl53	0.868161992	4.41985E-21
ENSMUSG00000041577	Prelp	0.669196176	1.77863E-75	ENSMUSG00000026271	Gpr35	0.86803523	5.59689E-36
ENSMUSG00000059493	Nhs	0.668999781	2.81958E-15	ENSMUSG00000015568	Lpl	0.867506478	1.2624E-103
ENSMUSG00000062794	Zfp599	0.668771624	0.001858417	ENSMUSG00000038967	Pdk2	0.867001032	7.87657E-09
ENSMUSG00000028776	Tinagl1	0.668456361	4.80087E-97	ENSMUSG00000030170	Wnt5b	0.866404034	0.036002215
ENSMUSG00000031613	Hpgd	0.667678635	0.004306155	ENSMUSG00000073608	Gal3st2c	0.865893546	0.001236353
ENSMUSG00000033855	Ston1	0.66599572	2.32704E-61	ENSMUSG00000020152	Actr2	0.865550257	6.7286E-204
ENSMUSG00000028096	Gpr89	0.665077381	7.63242E-20	ENSMUSG00000098158	Gm4804	0.865456693	7.25186E-10
ENSMUSG00000000794	Kcnn3	0.664610169	0.032916797	ENSMUSG00000095199	Zfp967	0.86507327	0.001090939
ENSMUSG00000063273	Naa15	0.664193668	1.21826E-56	ENSMUSG00000025736	Jmjd8	0.864368885	1.32247E-26
ENSMUSG00000032217	Rnf111	0.664084448	7.93746E-41	ENSMUSG00000018239	Zcchc10	0.862497211	9.03798E-15
ENSMUSG00000069135	Fgfr1op	0.664035371	7.64185E-34	ENSMUSG00000035671	Zswim4	0.861214077	5.87412E-78
ENSMUSG00000074657	Kif5a	0.663937278	0.003461307	ENSMUSG00000018916	Csf2	0.861033378	1.34012E-06
ENSMUSG00000033356	Pus7l	0.662916734	2.86685E-05	ENSMUSG00000025138	Sirt7	0.859441453	1.94551E-25
ENSMUSG00000023088	Abcc1	0.662594695	1.448E-103	ENSMUSG00000032217	Rnf111	0.857534414	1.19221E-72
ENSMUSG00000028700	Pomgnt1	0.662255762	1.01043E-51	ENSMUSG00000026718	Stam	0.857187572	2.7668E-72
ENSMUSG00000018604	Tbx3	0.661534847	8.23518E-06	ENSMUSG00000025812	Pard3	0.856457271	4.7494E-35
ENSMUSG00000058542	Gm15590	0.6613034	0.006251849	ENSMUSG00000043969	Emx2	0.85639036	0.020229614
ENSMUSG00000024621	Csf1r	0.660949443	3.81913E-11	ENSMUSG00000020056	Washc3	0.85594099	3.01065E-29
ENSMUSG00000116908	Gm49599	0.660734758	0.023521017	ENSMUSG00000014245	Pigl	0.855454349	1.50436E-11
ENSMUSG00000027248	Pdia3	0.658541004	9.0469E-187	ENSMUSG00000102573	Gm7265	0.854533783	0.001654742
ENSMUSG00000032092	Mpzl2	0.65769929	0.000278733	ENSMUSG00000074794	Arrdc3	0.854222683	1.07017E-38
ENSMUSG00000037251	Pomk	0.657411015	3.57077E-15	ENSMUSG00000048100	Taf13	0.85361839	4.80202E-44

ENSMUSG00000104394	Gm37254	0.656761073	0.014200567
ENSMUSG00000047731	Wbp1l	0.656661506	6.5417E-71
ENSMUSG00000042688	Mapk6	0.656631586	4.4172E-105
ENSMUSG00000053841	Txlna	0.656410731	2.17454E-53
ENSMUSG00000030560	Ctsc	0.656373856	2.08722E-11
ENSMUSG00000078864	Gm14322	0.655843783	0.027939706
ENSMUSG00000021959	Lats2	0.6555125	1.28479E-85
ENSMUSG00000079260	Tmppe	0.655045593	4.96829E-13
ENSMUSG00000023988	Bysl	0.654053567	4.41264E-31
ENSMUSG00000032334	Loxl1	0.653490406	4.01197E-93
ENSMUSG00000052760	A630001G21Rik	0.652948882	0.005025631
ENSMUSG00000041741	Pde3a	0.652616053	5.21724E-63
ENSMUSG00000021127	Zfp36l1	0.652505802	5.47984E-59
ENSMUSG00000039431	Mtmr7	0.651527928	0.00051316
ENSMUSG00000047554	Tmem41b	0.650412742	3.28561E-60
ENSMUSG00000046623	Gjb4	0.649601013	1.48195E-06
ENSMUSG00000029387	Gtf2h3	0.648472668	3.0752E-13
ENSMUSG00000028992	Nmnat1	0.648038376	1.36511E-06
ENSMUSG00000022371	Col14a1	0.647188079	3.20618E-82
ENSMUSG00000090952	Gm17251	0.646453806	0.015466175
ENSMUSG00000035863	Palm	0.6446701	1.06876E-28
ENSMUSG00000022769	Sdf2l1	0.644330042	1.85018E-14
ENSMUSG00000089704	Galnt2	0.643370448	1.7212E-102
ENSMUSG00000029267	Mtf2	0.643290022	9.47124E-26
ENSMUSG00000024346	Pfdn1	0.642315218	2.84078E-19
ENSMUSG00000025812	Pard3	0.641562784	9.4961E-22
ENSMUSG00000027006	Dnajc10	0.641276349	8.82649E-88
ENSMUSG00000026798	Coq4	0.641185912	8.58449E-06
ENSMUSG00000027808	Serp1	0.640418035	2.812E-131
ENSMUSG00000048600	Gm5763	0.639731308	0.003205948
ENSMUSG00000028803	Nipal3	0.639731139	0.004096689
ENSMUSG00000036136	Fam110c	0.639437858	1.28097E-08
ENSMUSG00000078517	Emc1	0.639417439	7.51932E-38
ENSMUSG00000028364	Tnc	0.639389547	9.9189E-115
ENSMUSG00000071984	Fndc1	0.638941777	1.0057E-37
ENSMUSG00000054611	Kdm2a	0.637335795	1.12051E-59
ENSMUSG00000024074	Crim1	0.636832542	3.31132E-79
ENSMUSG00000026289	Atg16l1	0.636323905	1.74992E-23
ENSMUSG00000062661	Ncs1	0.635399768	2.67004E-59
ENSMUSG00000097113	Gm19705	0.634527996	0.03362298
ENSMUSG00000078899	Gm4631	0.633470828	0.026227175
ENSMUSG00000078566	Bnip3	0.632779284	6.74383E-25
ENSMUSG00000037465	Klf10	0.632664074	3.60768E-20
ENSMUSG00000034800	Zfp661	0.632563848	0.016849121
ENSMUSG00000038725	Pkhd1l1	0.631160242	0.042220953
ENSMUSG00000074220	Zfp382	0.630982705	0.000215352
ENSMUSG00000005803	Sqor	0.630907419	1.29531E-22
ENSMUSG00000097472	Gm26586	0.629339812	0.043755434
ENSMUSG00000056692	Ilrun	0.628965305	2.39036E-70
ENSMUSG00000051279	Gdf6	0.628902322	4.46425E-95
ENSMUSG00000029761	Cald1	0.628815305	4.8662E-137
ENSMUSG00000033213	AA467197	0.628737733	0.003857452
ENSMUSG00000045136	Tubb2b	0.6287161	3.67624E-42
ENSMUSG00000055653	Gpc3	0.628605181	0.003662427
ENSMUSG00000024620	Pdgfrb	0.62832479	5.7348E-156
ENSMUSG00000039089	L3mbtl3	0.628306492	2.28426E-24
ENSMUSG00000022621	Rabl2	0.628220214	4.92114E-05
ENSMUSG00000022052	Ppp2r2a	0.627625705	1.2641E-59
ENSMUSG00000055435	Maf	0.627051629	0.002187067
ENSMUSG00000093979	Gm2237	0.626652432	0.0264971
ENSMUSG00000003363	Pld3	0.625849303	1.93393E-62
ENSMUSG00000025934	Gsta3	0.625809358	1.56055E-20
ENSMUSG00000026135	Zfp142	0.625580462	9.8675E-23
ENSMUSG00000040713	Creg1	0.625195115	4.2415E-40
ENSMUSG00000025911	Adhfe1	0.624835589	7.98048E-05
ENSMUSG00000020571	Pdia6	0.623853817	3.4561E-122
ENSMUSG00000096971	4930556M19Rik	0.623302095	0.004427888
ENSMUSG00000001348	Acp5	0.623090669	0.002502712
ENSMUSG00000025591	Tma16	0.622654176	6.12527E-16
ENSMUSG00000086290	Snhg12	0.622603669	2.76116E-09
ENSMUSG00000000823	Zfp512b	0.620694092	1.46819E-19
ENSMUSG00000026864	Hspa5	0.620505858	1.1831E-133
ENSMUSG00000025402	Nab2	0.620366066	1.27816E-48
ENSMUSG00000037470	Uggt1	0.620274287	1.0443E-98
ENSMUSG00000061731	Ext1	0.619739068	6.3875E-135
ENSMUSG00000039055	Eme1	0.619582099	0.001658295
ENSMUSG00000045374	Wdr81	0.619397497	3.50318E-35
ENSMUSG00000021190	Lgmn	0.853418639	1.6498E-104
ENSMUSG00000047554	Tmem41b	0.853334495	1.17564E-77
ENSMUSG00000089940	Gm4117	0.853180498	0.009125769
ENSMUSG00000030088	Aldh1l1	0.852462009	2.24346E-51
ENSMUSG00000074221	Zfp568	0.852126243	2.70641E-90
ENSMUSG00000089756	Zfp966	0.851222426	0.007028002
ENSMUSG00000021779	Thrb	0.851173032	1.63545E-06
ENSMUSG00000087260	Lamtor5	0.850845686	8.21852E-33
ENSMUSG00000026701	Prdx6	0.850278764	1.2969E-110
ENSMUSG00000023088	Abcc1	0.850132649	1.3479E-214
ENSMUSG00000025409	Mbd6	0.847590142	4.88419E-26
ENSMUSG00000051367	Six1	0.847006855	5.4873E-06
ENSMUSG00000021699	Pde4d	0.846264479	8.89207E-12
ENSMUSG00000039701	Usp53	0.845873322	5.04373E-56
ENSMUSG00000050549	Fam241a	0.845367559	4.74728E-08
ENSMUSG00000037913	Tmem156	0.8452095	0.033162801
ENSMUSG00000056220	Pla2g4a	0.844602985	2.203E-116
ENSMUSG00000079260	Tmppe	0.843887203	1.7178E-26
ENSMUSG00000029287	Tgfbr3	0.843521397	2.60884E-54
ENSMUSG00000059498	Fcgr3	0.842757968	0.001462929
ENSMUSG00000020794	Ube2g1	0.84188636	1.43821E-43
ENSMUSG00000056692	Ilrun	0.841565659	7.6677E-141
ENSMUSG00000016256	Ctsz	0.841212892	2.0776E-152
ENSMUSG00000032754	Slc8b1	0.84120811	2.01833E-41
ENSMUSG00000053475	Tnfaip6	0.840855696	9.96314E-36
ENSMUSG00000071540	3425401B19Rik	0.84005743	2.20022E-11
ENSMUSG00000038860	Garnl3	0.838231973	2.87549E-29
ENSMUSG00000049999	Ppp1r3d	0.838216775	0.00479908
ENSMUSG00000048277	Syngr2	0.838216159	1.08069E-69
ENSMUSG00000020101	Vsir	0.837837179	6.79447E-05
ENSMUSG00000033149	Phldb2	0.836421346	5.4016E-201
ENSMUSG00000028126	Pip5k1a	0.835353956	1.8482E-138
ENSMUSG00000103593	Gm37352	0.834514993	0.001954546
ENSMUSG00000024642	Tle4	0.833740233	7.09766E-22
ENSMUSG00000003863	Ppfia3	0.833665929	0.017986085
ENSMUSG00000020434	4921536K21Rik	0.833415237	0.039191977
ENSMUSG00000074934	Grem1	0.833351006	3.72195E-13
ENSMUSG00000067148	Polr1c	0.832107841	1.256E-57
ENSMUSG00000020629	Adi1	0.832027678	4.34848E-30
ENSMUSG00000025804	Ccr1	0.829716969	0.00537001
ENSMUSG00000002897	Il17ra	0.829337621	1.6695E-101
ENSMUSG00000104913	Gm6560	0.827070429	1.08099E-09
ENSMUSG00000075602	Ly6a	0.826667763	6.71286E-31
ENSMUSG00000042377	Fam83g	0.826562015	0.009952258
ENSMUSG00000047205	Dusp18	0.826205981	9.06237E-36
ENSMUSG00000028883	Sema3a	0.825746601	1.93357E-06
ENSMUSG00000035086	Becn1	0.824719528	4.00194E-98
ENSMUSG00000027618	Nfs1	0.823864722	2.38258E-32
ENSMUSG00000038267	Slc22a23	0.823488745	5.15448E-08
ENSMUSG00000028247	Coq3	0.823484308	5.27825E-27
ENSMUSG00000028744	Pqlc2	0.823436503	5.50552E-28
ENSMUSG00000025538	Sumf2	0.822695919	3.95986E-28
ENSMUSG00000019878	Hsf2	0.821321172	1.65897E-27
ENSMUSG00000062382	Ftl1-ps1	0.820680707	7.78435E-23
ENSMUSG00000031596	Slc7a2	0.820529209	9.2237E-125
ENSMUSG00000047786	Lix1	0.820178351	0.00524565
ENSMUSG00000020399	Havcr2	0.820111363	0.007702479
ENSMUSG00000010517	Faf1	0.819274705	2.02065E-62
ENSMUSG00000023074	Mospd1	0.819002972	1.54661E-47
ENSMUSG00000020467	Efemp1	0.818568137	2.758E-148
ENSMUSG00000043153	Crppa	0.818357791	3.39974E-07
ENSMUSG00000051335	Gfod1	0.816521356	1.32524E-63
ENSMUSG00000037827	Gm5884	0.816047272	0.010614822
ENSMUSG00000011960	Ccnt1	0.815967006	3.6818E-80
ENSMUSG00000032475	Nck1	0.815341038	7.62798E-66
ENSMUSG00000048807	Slc35e4	0.814681624	1.09634E-39
ENSMUSG00000028383	Hsdl2	0.814330415	1.71679E-21
ENSMUSG00000021139	Gm20498	0.814094251	3.08407E-05
ENSMUSG00000038683	Pak1ip1	0.813230287	9.2647E-104
ENSMUSG00000029413	Naaa	0.812892359	5.56345E-11
ENSMUSG00000027709	Mccc1	0.812156333	7.66083E-57
ENSMUSG00000037217	Syn1	0.811963569	0.000103667
ENSMUSG00000103144	Pcdhga1	0.811833034	4.74755E-05
ENSMUSG00000019927	Ube2d1	0.811699854	3.03996E-31
ENSMUSG00000028975	Pex14	0.811533198	5.88759E-49
ENSMUSG00000059316	Slc27a4	0.811317104	2.1276E-76
ENSMUSG00000024480	Ap3s1	0.810388219	8.03183E-87

ENSMUSG00000027560	Dok5	0.618913035	1.3556E-07
ENSMUSG00000035305	Ror1	0.618848039	1.58071E-06
ENSMUSG00000022367	Has2	0.618753722	4.10938E-07
ENSMUSG00000002108	Nr1h3	0.618735395	7.37855E-05
ENSMUSG00000028962	Slc4a2	0.618428274	6.0737E-126
ENSMUSG00000002580	Mien1	0.618325489	3.64972E-15
ENSMUSG00000032840	2410131K14Rik	0.618172313	1.36713E-12
ENSMUSG00000039004	Bmp6	0.618149907	1.83749E-43
ENSMUSG00000036782	Klhl13	0.618135447	2.89221E-51
ENSMUSG00000025212	Sfxn3	0.616891233	1.33757E-34
ENSMUSG00000067219	Nipal1	0.616252867	1.03504E-12
ENSMUSG00000087635	Gm13414	0.615985685	0.032059364
ENSMUSG00000022793	B4galt4	0.615704832	1.19639E-07
ENSMUSG00000002897	Il17ra	0.615321162	1.69137E-39
ENSMUSG00000000555	Itga5	0.614394823	1.8828E-132
ENSMUSG00000038375	Trp53inp2	0.614249897	6.175E-118
ENSMUSG00000050619	Zscan29	0.614236323	2.30498E-14
ENSMUSG00000032816	Igdcc4	0.614048057	1.10606E-11
ENSMUSG00000057069	Ero1lb	0.6140343	2.34601E-07
ENSMUSG00000060594	Layn	0.613541346	4.53762E-61
ENSMUSG00000078870	Gm14410	0.612285613	9.25599E-07
ENSMUSG00000021939	Ctsb	0.609637696	1.1168E-146
ENSMUSG00000052698	Tln2	0.608972245	2.95396E-28
ENSMUSG00000031729	Ist1	0.608885365	3.21527E-43
ENSMUSG00000036959	Bcorl1	0.608726082	5.48986E-12
ENSMUSG00000025355	Mmp19	0.608367329	1.96886E-24
ENSMUSG00000020696	Rffl	0.60794213	5.61633E-38
ENSMUSG00000001131	Timp1	0.60766003	2.9869E-119
ENSMUSG00000059423	Zfp933	0.607529352	3.96259E-07
ENSMUSG00000093661	Eif4e3	0.606252391	5.53439E-07
ENSMUSG00000059588	Calcrl	0.606051199	1.18837E-30
ENSMUSG00000028691	Prdx1	0.605894523	6.5408E-120
ENSMUSG00000062116	Zfp954	0.605775278	5.55053E-06
ENSMUSG00000001827	Folr1	0.605300937	7.6415E-06
ENSMUSG00000025969	Nrp2	0.604559839	5.86019E-78
ENSMUSG00000025130	P4hb	0.604273901	6.4447E-161
ENSMUSG00000025724	Sec11a	0.604204713	6.77473E-38
ENSMUSG00000032601	Prkar2a	0.603833703	7.54204E-39
ENSMUSG00000039115	Itga9	0.603707311	1.63558E-50
ENSMUSG00000025069	Gsto2	0.603370453	0.00678073
ENSMUSG00000021996	Esd	0.602442082	1.5772E-118
ENSMUSG00000051817	Sox12	0.601082472	0.000199749
ENSMUSG00000055421	Pcdh9	0.600476725	4.63124E-21
ENSMUSG00000036067	Slc2a6	0.600033002	0.039784645
ENSMUSG00000095362	Gm14325	0.599870169	8.95691E-06
ENSMUSG00000041355	Ssr2	0.599537438	2.67179E-87
ENSMUSG00000030753	Thap12	0.598609735	1.32971E-31
ENSMUSG00000039307	Hexdc	0.598476094	0.019445067
ENSMUSG00000040219	Ttc12	0.597085977	3.09418E-05
ENSMUSG00000030116	Mfap5	0.595415941	5.73576E-30
ENSMUSG00000041438	Utp4	0.595190278	1.80447E-32
ENSMUSG00000025809	Itgb1	0.594086056	1.7869E-124
ENSMUSG00000003814	Calr	0.593752341	7.0817E-159
ENSMUSG00000078768	Zfp566	0.593611525	0.03272624
ENSMUSG00000102555	6430511E19Rik	0.593302449	0.009216401
ENSMUSG00000037370	Enpp1	0.592780063	2.13591E-54
ENSMUSG00000031617	Tmem184c	0.592763485	1.09578E-21
ENSMUSG00000029233	Srd5a3	0.592448476	1.67635E-20
ENSMUSG00000024379	Tslp	0.590933683	0.000596062
ENSMUSG00000024952	Rps6ka4	0.590810498	5.29826E-33
ENSMUSG00000033542	Arhgef5	0.589731962	7.99892E-26
ENSMUSG00000031835	Mbtps1	0.588865801	6.2477E-58
ENSMUSG00000021807	Rtraf	0.588836885	9.88057E-47
ENSMUSG00000023186	Vwa5a	0.588668378	1.81917E-31
ENSMUSG00000029998	Pcyox1	0.588028586	3.33558E-59
ENSMUSG00000037443	Cep85	0.587904411	7.20835E-12
ENSMUSG00000032786	Alas1	0.587642135	1.22974E-47
ENSMUSG00000037029	Zfp146	0.58710335	5.30407E-21
ENSMUSG00000030203	Dusp16	0.586771281	1.92791E-22
ENSMUSG00000038023	Atp6v0a2	0.586418809	2.02892E-55
ENSMUSG00000036046	5031439G07Rik	0.586127346	2.85835E-56
ENSMUSG00000034501	Pcnx4	0.585905466	1.31521E-12
ENSMUSG00000026566	Mpzl1	0.58531969	1.14679E-40
ENSMUSG00000039474	Wfs1	0.585238436	1.92017E-19
ENSMUSG00000090626	Tex9	-0.585268497	0.001924668
ENSMUSG00000040270	Bach2	-0.585535488	0.035695083
ENSMUSG00000050627	Gpd1l	-0.58584961	2.76667E-22
ENSMUSG00000029198	Grpel1	0.810120515	9.1585E-49
ENSMUSG00000030225	Dera	0.809665745	2.05498E-30
ENSMUSG00000050619	Zscan29	0.80891011	1.3639E-26
ENSMUSG00000068270	Shroom4	0.806075384	7.00576E-24
ENSMUSG00000035329	Fbxo33	0.805915061	3.80013E-50
ENSMUSG00000059714	Flot1	0.8058235	4.84802E-78
ENSMUSG00000093798	Gm8355	0.805588648	1.28378E-14
ENSMUSG00000078901	Gm14440	0.80520571	5.92407E-05
ENSMUSG00000025731	Mettl26	0.80478292	0.000260999
ENSMUSG00000031298	Adgrg2	0.804627623	2.08662E-45
ENSMUSG00000031847	1700030J22Rik	0.804580927	0.046563352
ENSMUSG00000021257	Angel1	0.804277597	1.27737E-05
ENSMUSG00000004040	Stat3	0.802906468	1.9409E-189
ENSMUSG00000036959	Bcorl1	0.802880888	4.41478E-22
ENSMUSG00000040181	Fmo1	0.802878873	0.000463666
ENSMUSG00000027560	Dok5	0.802613391	1.91685E-14
ENSMUSG00000097530	Kansl2-ps	0.802358249	2.16001E-05
ENSMUSG00000039934	Gsap	0.802250972	9.99995E-07
ENSMUSG00000036067	Slc2a6	0.80223473	0.00246247
ENSMUSG00000022099	Dmtn	0.801977935	0.000459781
ENSMUSG00000052459	Atp6v1a	0.801906231	1.1569E-193
ENSMUSG00000023018	Smarcd1	0.801569191	5.77416E-25
ENSMUSG00000055128	Cgrrf1	0.801075443	7.80846E-22
ENSMUSG00000023048	Prr13	0.800445784	1.79669E-66
ENSMUSG00000029387	Gtf2h3	0.800378107	1.06666E-19
ENSMUSG00000018209	Stk4	0.800277993	5.57488E-74
ENSMUSG00000031169	Porcn	0.800233219	0.007411392
ENSMUSG00000032507	Fbxl2	0.799647649	5.06493E-15
ENSMUSG00000078898	Zfp968	0.799085861	0.004984256
ENSMUSG00000020598	Nrcam	0.79789245	3.02112E-11
ENSMUSG00000022856	Tmem41a	0.797585992	3.38617E-16
ENSMUSG00000022479	Vdr	0.797569197	1.94787E-08
ENSMUSG00000053411	Cbx7	0.796712956	0.000238467
ENSMUSG00000030790	Adm	0.796526929	1.17025E-77
ENSMUSG00000035725	Prkx	0.795523283	9.17137E-93
ENSMUSG00000033538	Casp4	0.793910001	5.49205E-49
ENSMUSG00000037820	Tgm2	0.793177467	9.30387E-50
ENSMUSG00000018740	Slc25a35	0.792834409	8.10218E-05
ENSMUSG00000090093	Gm14399	0.789917808	0.00071962
ENSMUSG00000028631	Kcnq4	0.789686305	0.028476311
ENSMUSG00000031458	Coprs	0.789649612	2.58077E-18
ENSMUSG00000024187	Fam234a	0.789389585	1.56364E-67
ENSMUSG00000115317	Gm32618	0.789267884	0.000422125
ENSMUSG00000018395	Kif3a	0.788950303	2.62811E-49
ENSMUSG00000060594	Layn	0.788346115	1.6237E-102
ENSMUSG00000098022	Zfp82	0.788299748	0.00645935
ENSMUSG00000029072	Tas1r3	0.788233548	0.014306128
ENSMUSG00000026479	Lamc2	0.787295991	3.44439E-45
ENSMUSG00000044231	Nhlrc1	0.784714806	0.016609356
ENSMUSG00000110040	Gm49369	0.784325171	0.03121363
ENSMUSG00000059040	Eno1b	0.783704709	1.20463E-06
ENSMUSG00000028793	Rnf19b	0.782551171	1.22347E-45
ENSMUSG00000045980	Tmem104	0.78235777	7.03463E-27
ENSMUSG00000020198	Ap3d1	0.781826372	9.5921E-113
ENSMUSG00000049811	Fam161a	0.781737768	0.000207682
ENSMUSG00000093908	Gm5784	0.781309685	0.023010869
ENSMUSG00000024867	Pip5k1b	0.78089109	0.004434295
ENSMUSG00000062753	AI413582	0.780520666	8.2893E-13
ENSMUSG00000038065	Mturn	0.779518622	3.55073E-24
ENSMUSG00000052428	Tmco1	0.778618622	1.20643E-62
ENSMUSG00000021963	Sap18	0.778240721	1.1559E-133
ENSMUSG00000022126	Acod1	0.777482571	0.045131625
ENSMUSG00000029386	Tctn2	0.777419718	2.10772E-15
ENSMUSG00000034761	Map4k5	0.776867185	8.54259E-58
ENSMUSG00000047945	Marcksl1	0.776550819	3.05136E-56
ENSMUSG00000013415	Igf2bp1	0.776319581	8.58401E-05
ENSMUSG00000025586	Cpeb1	0.775543986	1.51935E-56
ENSMUSG00000026819	Slc25a25	0.775428788	1.26282E-59
ENSMUSG00000108228	6430584L05Rik	0.77517775	0.000645771
ENSMUSG00000018068	Ints2	0.774994366	2.71599E-35
ENSMUSG00000036242	Armh4	0.774631945	0.002460246
ENSMUSG00000032252	Glce	0.773297842	8.53826E-45
ENSMUSG00000028048	Gba	0.772731085	5.2341E-119
ENSMUSG00000032652	Crebl2	0.772525763	5.93442E-17
ENSMUSG00000020153	Ndufs7	0.772439725	2.56498E-10
ENSMUSG00000047632	Fgfbp3	0.771999645	0.00022419
ENSMUSG00000072969	Armcx5	0.771317423	1.14727E-18

ENSMUSG00000086513	9130208D14Rik	-0.586470509	0.003279451	ENSMUSG00000045092	S1pr1	0.771023819	8.49834E-36
ENSMUSG00000022561	Gpaa1	-0.586862595	6.01628E-24	ENSMUSG00000085148	Mir22hg	0.76856104	8.29344E-58
ENSMUSG00000029463	Fam216a	-0.587093375	4.89044E-08	ENSMUSG00000027937	Jtb	0.768026232	8.94717E-27
ENSMUSG00000071001	Hrct1	-0.587128486	0.002880214	ENSMUSG00000073725	Lmbrd1	0.767936186	1.21912E-69
ENSMUSG00000034898	Filip1	-0.587456459	0.000685141	ENSMUSG00000027300	Ubox5	0.7672778	5.51998E-10
ENSMUSG00000041268	Dmxl2	-0.587726559	2.47834E-08	ENSMUSG00000031153	Gripap1	0.767019427	3.29321E-25
ENSMUSG00000029101	Rgs12	-0.587969683	6.33234E-33	ENSMUSG00000042298	Ttc19	0.766333471	7.24655E-31
ENSMUSG00000037375	Hhat	-0.588528736	1.19668E-06	ENSMUSG00000030681	Mvp	0.766112483	4.5201E-158
ENSMUSG00000038517	Tbkbp1	-0.58856586	1.69876E-06	ENSMUSG00000050335	Lgals3	0.765646923	2.98105E-45
ENSMUSG00000033880	Lgals3bp	-0.589472724	0.000735237	ENSMUSG00000060376	Bckdha	0.764232939	1.32029E-16
ENSMUSG00000042029	Ncapg2	-0.589621759	1.02248E-29	ENSMUSG00000034612	Chst11	0.76373137	1.8137E-127
ENSMUSG00000027469	Tpx2	-0.590320769	1.76155E-70	ENSMUSG00000029190	D5Ertd579e	0.763711983	1.68344E-69
ENSMUSG00000021936	Mapk8	-0.591285299	2.56689E-21	ENSMUSG00000030494	Rhpn2	0.763609323	0.000143226
ENSMUSG00000054863	Tafa5	-0.591304089	0.023509807	ENSMUSG00000046532	Ar	0.762744411	5.09101E-26
ENSMUSG00000005078	Jkamp	-0.592027722	2.16876E-15	ENSMUSG00000081534	Slc48a1	0.762177119	2.33482E-74
ENSMUSG00000020272	Stk10	-0.592990807	5.26095E-16	ENSMUSG00000022089	Bin3	0.76159388	5.78111E-36
ENSMUSG00000024817	Uhrf2	-0.593295775	1.28999E-41	ENSMUSG00000053799	Exoc6	0.761375423	2.71529E-07
ENSMUSG00000026123	Plekhb2	-0.593444802	3.17619E-48	ENSMUSG00000018446	C1qbp	0.760018811	1.77791E-98
ENSMUSG00000024079	Eif2ak2	-0.593635917	9.85385E-26	ENSMUSG00000031974	Abcb10	0.759925912	1.80501E-30
ENSMUSG00000031517	Gpm6a	-0.594253196	7.63854E-08	ENSMUSG00000024150	Mcfd2	0.759502907	9.6573E-141
ENSMUSG00000029153	Ociad2	-0.594577761	0.001235503	ENSMUSG00000079316	Rab9	0.759452792	1.5262E-30
ENSMUSG00000074170	Plekhf1	-0.595374099	2.40362E-11	ENSMUSG00000026383	Epb41l5	0.759169394	2.29302E-06
ENSMUSG00000015749	Anp32e	-0.595386632	1.29513E-41	ENSMUSG00000014905	Dnajb9	0.758681874	1.12596E-57
ENSMUSG00000025958	Creb1	-0.596598092	1.40139E-25	ENSMUSG00000028057	Rit1	0.758578758	8.18302E-36
ENSMUSG00000031453	Rasa3	-0.59668663	4.91889E-58	ENSMUSG00000070822	Zscan18	0.758362625	0.012199188
ENSMUSG00000078153	Psme2b	-0.596702658	1.732E-05	ENSMUSG00000087143	A830082K12Rik	0.757461445	0.020216776
ENSMUSG00000044966	Fbxo48	-0.597741786	0.013177962	ENSMUSG00000039763	Dnajc28	0.757110311	3.17924E-05
ENSMUSG00000062248	Cks2	-0.597980059	2.15751E-16	ENSMUSG00000032712	Resf1	0.756826534	5.73421E-42
ENSMUSG00000016559	H3f3b	-0.598251202	1.162E-104	ENSMUSG00000066361	Serpina3c	0.756818056	6.30003E-05
ENSMUSG00000020415	Pttg1	-0.59856549	1.34904E-06	ENSMUSG00000040697	Dnajc16	0.755474965	3.5082E-26
ENSMUSG00000106464	C130083M11Rik	-0.59864403	0.000566643	ENSMUSG00000033526	Ppip5k1	0.755170225	6.9944E-23
ENSMUSG00000027641	Rbl1	-0.599041546	7.28493E-18	ENSMUSG00000058581	Gm5801	0.755076271	0.041859243
ENSMUSG00000051855	Mest	-0.599554302	0.002121808	ENSMUSG00000058672	Tubb2a	0.754758176	8.2455E-115
ENSMUSG00000002222	Rmnd5a	-0.600113436	1.34361E-32	ENSMUSG00000047216	Cdh19	0.754627431	1.11738E-11
ENSMUSG00000042331	Specc1	-0.600253861	5.49835E-37	ENSMUSG00000089951	Zfp968-ps	0.753307537	0.002899841
ENSMUSG00000038145	Snrk	-0.600416795	6.73029E-11	ENSMUSG00000014776	Nol3	0.753284892	1.09372E-09
ENSMUSG00000022105	Rb1	-0.60046803	3.74319E-17	ENSMUSG00000074657	Kif5a	0.752945775	0.000452829
ENSMUSG00000059901	Adamts14	-0.60078391	5.24767E-18	ENSMUSG00000042770	Hebp1	0.752187006	4.12598E-15
ENSMUSG00000039648	Kyat1	-0.600846481	0.042776845	ENSMUSG00000032952	Ap4b1	0.752017968	1.58237E-17
ENSMUSG00000048988	Elfn1	-0.602177342	0.004278095	ENSMUSG00000039770	Ypel5	0.751783215	6.95924E-46
ENSMUSG00000000776	Polr3d	-0.602280873	2.19572E-29	ENSMUSG00000070323	Mmp27	0.7516388	5.97185E-07
ENSMUSG00000066595	Flvcr1	-0.60260971	2.43375E-16	ENSMUSG00000036136	Fam110c	0.751148156	1.2573E-12
ENSMUSG00000016128	Stard13	-0.602694475	3.21021E-20	ENSMUSG00000036402	Gng12	0.750521818	1.3878E-154
ENSMUSG00000003038	Hmgn2	-0.60275438	1.21883E-53	ENSMUSG00000024290	Rock1	0.748676005	2.00899E-82
ENSMUSG00000005370	Msh6	-0.602960465	2.9399E-26	ENSMUSG00000030304	Ergic2	0.748581254	5.00022E-61
ENSMUSG00000044551	9930012K11Rik	-0.603000793	0.00044964	ENSMUSG00000042759	Apobr	0.748388765	3.95706E-18
ENSMUSG00000053279	Aldh1a1	-0.603029913	2.7124E-122	ENSMUSG00000041975	Mettl8	0.748319746	1.93134E-09
ENSMUSG00000039577	Nphp4	-0.603307734	0.018296059	ENSMUSG00000039457	Ppl	0.748018156	7.09477E-20
ENSMUSG00000032462	Pik3cb	-0.603313772	3.42958E-20	ENSMUSG00000006386	Tek	0.746874198	3.85637E-21
ENSMUSG00000038718	Pbx3	-0.603684456	2.06138E-14	ENSMUSG00000033327	Tnxb	0.746694236	2.95279E-66
ENSMUSG00000004768	Rab23	-0.603727973	9.74385E-24	ENSMUSG00000029131	Dnajb6	0.74643022	4.0542E-133
ENSMUSG00000031934	Panx1	-0.604228519	1.66529E-16	ENSMUSG00000032463	Faim	0.746217029	8.91372E-21
ENSMUSG00000018809	Smyd4	-0.604478661	1.20495E-05	ENSMUSG00000022246	Rai14	0.745605577	9.5924E-156
ENSMUSG00000032192	Gnb5	-0.60514036	2.66186E-11	ENSMUSG00000030587	2200002D01Rik	0.745022798	2.2986E-06
ENSMUSG00000038507	Parp12	-0.605200172	0.005722044	ENSMUSG00000059743	Fdps	0.74373862	7.5831E-60
ENSMUSG00000014932	Yes1	-0.60610045	1.57145E-25	ENSMUSG00000003849	Nqo1	0.74362565	3.71952E-55
ENSMUSG00000026994	Galnt3	-0.606331399	0.005766681	ENSMUSG00000022453	Naga	0.742174799	2.81906E-53
ENSMUSG00000036875	Dna2	-0.607372572	0.002544233	ENSMUSG00000071650	Ganab	0.741874746	6.50599E-92
ENSMUSG00000033392	Clasp2	-0.607441702	1.35277E-40	ENSMUSG00000022797	Tfrc	0.741471392	2.7785E-81
ENSMUSG00000047003	Zfp41	-0.607632985	1.09694E-05	ENSMUSG00000040147	Maob	0.739980736	2.1193E-38
ENSMUSG00000045751	Mms22l	-0.608317924	2.96624E-10	ENSMUSG00000004667	Polr2e	0.739711594	2.90029E-34
ENSMUSG00000035275	Raver2	-0.608329365	0.02463843	ENSMUSG00000027871	Hsd3b1	0.739492218	0.004416368
ENSMUSG00000036887	C1qa	-0.608468291	0.021082506	ENSMUSG00000027346	Gpcpd1	0.738940261	5.90576E-61
ENSMUSG00000039601	Rcan2	-0.608835874	8.2298E-23	ENSMUSG00000100801	Gm15459	0.738847554	3.5387E-117
ENSMUSG00000010476	Ebf3	-0.608957108	1.78276E-12	ENSMUSG00000028300	C9orf72	0.738781498	2.07569E-19
ENSMUSG00000032586	Traip	-0.609623883	3.44947E-07	ENSMUSG00000045664	Cdc42ep2	0.73876091	3.768E-24
ENSMUSG00000029094	Afap1	-0.609836192	3.90871E-38	ENSMUSG00000022771	Ppil2	0.738758093	3.66405E-34
ENSMUSG00000005968	Tuft1	-0.610121829	4.17489E-09	ENSMUSG00000009907	Vps4b	0.738231414	3.8584E-122
ENSMUSG00000035572	Dcaf10	-0.610749539	4.07655E-31	ENSMUSG00000022802	Lmln	0.73799715	0.003479943
ENSMUSG00000026134	Prim2	-0.610975279	2.22385E-14	ENSMUSG00000028133	Rwdd3	0.737362279	0.025805514
ENSMUSG00000051220	Ercc6l	-0.611406878	6.57077E-14	ENSMUSG00000017837	Nkiras2	0.737028337	4.48902E-42
ENSMUSG00000027977	Ndst3	-0.611807076	5.25928E-05	ENSMUSG00000024818	Slc25a45	0.736726831	2.00707E-10
ENSMUSG00000032249	Anp32a	-0.612437802	1.26721E-23	ENSMUSG00000039740	Alg2	0.736147274	7.75637E-35
ENSMUSG00000050295	Foxc1	-0.612453326	4.94787E-12	ENSMUSG00000015656	Hspa8	0.735953783	3.0517E-212
ENSMUSG00000046561	Arsj	-0.612939012	1.17292E-07	ENSMUSG00000000794	Kcnn3	0.735781303	0.011385792
ENSMUSG00000024597	Slc12a2	-0.612962415	1.18025E-59	ENSMUSG00000015839	Nfe2l2	0.735141903	4.9939E-175
ENSMUSG00000106987	Gm42575	-0.612978936	0.045439811	ENSMUSG00000027102	Hoxd8	0.734248965	0.001133952

ENSMUSG00000044197	Gpr146	-0.614079804	7.64595E-14	ENSMUSG00000020427	Igfbp3	0.734154869	1.89382E-31
ENSMUSG00000091387	Gcnt4	-0.614729981	1.19928E-06	ENSMUSG00000015711	Prune1	0.733706408	4.59924E-34
ENSMUSG00000028893	Sesn2	-0.614919029	5.88689E-21	ENSMUSG00000021037	Ahsa1	0.7331844	7.08967E-66
ENSMUSG00000020681	Ace	-0.615100489	1.54173E-43	ENSMUSG00000046138	9930021J03Rik	0.733182808	5.37321E-34
ENSMUSG00000020513	Tubd1	-0.615466878	0.000115686	ENSMUSG00000032216	Nedd4	0.732934735	6.4361E-159
ENSMUSG00000018263	Tbx5	-0.615627553	0.016396323	ENSMUSG00000028145	Them4	0.73074044	2.74321E-07
ENSMUSG00000029725	Ppp1r35	-0.615646958	9.02694E-05	ENSMUSG00000019990	Pde7b	0.72996027	1.69015E-18
ENSMUSG00000021714	Cenpk	-0.615847129	5.81221E-05	ENSMUSG00000022634	Yaf2	0.729698316	7.75637E-35
ENSMUSG00000033157	Abhd10	-0.616846317	5.03715E-07	ENSMUSG00000030739	Myh14	0.729610216	0.017256842
ENSMUSG00000053560	Ier2	-0.617166708	7.1821E-17	ENSMUSG00000025337	Sbds	0.729002461	6.87124E-47
ENSMUSG00000074151	Nlrc5	-0.617319246	0.00104114	ENSMUSG00000034616	Ssh3	0.728593689	3.37408E-30
ENSMUSG00000078794	Dact3	-0.61815888	7.11139E-13	ENSMUSG00000021669	Col4a3bp	0.726654217	8.11998E-64
ENSMUSG00000025427	Rnf165	-0.618337979	0.00133338	ENSMUSG00000041355	Ssr2	0.725346257	9.3075E-142
ENSMUSG00000036555	Iqce	-0.618869036	1.39934E-12	ENSMUSG00000032298	Neil1	0.724158675	0.000188116
ENSMUSG00000061143	Maml3	-0.618874952	0.000623556	ENSMUSG00000030741	Spns1	0.724035217	1.00449E-38
ENSMUSG00000038241	Cep250	-0.619015717	3.4624E-43	ENSMUSG00000006169	Clint1	0.723973044	4.8013E-114
ENSMUSG00000036678	Aaas	-0.619251614	5.93377E-19	ENSMUSG00000029699	Ssc4d	0.723726782	0.003040966
ENSMUSG00000025060	Slk	-0.61951831	8.95581E-66	ENSMUSG00000025509	Pnpla2	0.723405666	1.34772E-29
ENSMUSG00000024352	Spata24	-0.619629026	0.004802869	ENSMUSG00000033658	Ddx19b	0.723096209	4.05676E-22
ENSMUSG00000046794	Ppp1r3b	-0.621726394	0.000176908	ENSMUSG00000024561	Mbd1	0.723007579	1.38937E-88
ENSMUSG00000018509	Cenpv	-0.62262125	0.003405116	ENSMUSG00000032309	Fbxo22	0.72242343	8.22852E-28
ENSMUSG00000050379	Septin6	-0.62267413	5.05142E-12	ENSMUSG00000000826	Dnajc5	0.721457188	6.79183E-86
ENSMUSG00000049858	Suox	-0.62308551	5.55601E-06	ENSMUSG00000073856	Iqck	0.721325847	0.011868307
ENSMUSG00000032113	Chek1	-0.62315405	2.42691E-10	ENSMUSG00000022793	B4galt4	0.721306672	4.70706E-09
ENSMUSG00000075033	Nxpe3	-0.623268777	5.17645E-05	ENSMUSG00000091512	Lamtor3	0.721261652	1.01611E-83
ENSMUSG00000003452	Bicd1	-0.623968645	0.001755385	ENSMUSG00000075600	Zc3h3	0.720951684	1.85446E-21
ENSMUSG00000064090	Vrk2	-0.625012265	1.78156E-07	ENSMUSG00000020482	Ccdc117	0.717993438	5.49517E-15
ENSMUSG00000030551	Nr2f2	-0.625103189	3.5728E-13	ENSMUSG00000018861	Fdxr	0.717605608	7.82005E-09
ENSMUSG00000021775	Nr1d2	-0.625451613	1.1516E-28	ENSMUSG00000037406	Htra4	0.717383503	6.4642E-07
ENSMUSG00000028551	Cdkn2c	-0.625962774	1.84623E-22	ENSMUSG00000036764	Dnajc12	0.717273424	0.000629404
ENSMUSG00000024049	Myom1	-0.626041153	0.030411465	ENSMUSG00000029203	Ube2k	0.716959235	3.92796E-65
ENSMUSG00000046324	Ermp1	-0.626101371	1.35778E-16	ENSMUSG00000060923	Acyp2	0.716869378	8.98521E-13
ENSMUSG00000031628	Casp3	-0.626626482	7.87249E-36	ENSMUSG00000033730	Egr3	0.716830388	1.36761E-07
ENSMUSG00000022673	Mcm4	-0.626819817	3.45982E-49	ENSMUSG00000027913	Crct1	0.716598584	1.51892E-31
ENSMUSG00000033685	Ucp2	-0.627120311	0.0052571	ENSMUSG00000025532	Crcp	0.716165761	8.82155E-22
ENSMUSG00000022043	Trim35	-0.627261245	6.02293E-66	ENSMUSG00000001482	Def8	0.716073962	3.63016E-26
ENSMUSG00000059834	Sclt1	-0.627365432	1.29112E-06	ENSMUSG00000097472	Gm26586	0.714531458	0.01377811
ENSMUSG00000116138	C030006K11Rik	-0.62790502	5.25887E-05	ENSMUSG00000087635	Gm13414	0.714420208	0.009272376
ENSMUSG00000028678	Kif2c	-0.628472126	6.31798E-29	ENSMUSG00000039942	Ptger4	0.713747782	8.38584E-29
ENSMUSG00000028413	B4galt1	-0.628703605	9.4439E-109	ENSMUSG00000032905	Atg12	0.713651054	2.72214E-35
ENSMUSG00000040328	Olfr56	-0.629009094	0.00066265	ENSMUSG00000020122	Egfr	0.713648089	7.86067E-92
ENSMUSG00000027654	Fam83d	-0.629114847	5.38196E-13	ENSMUSG00000024456	Diaph1	0.712883041	1.51667E-92
ENSMUSG00000046532	Ar	-0.629453971	1.25879E-09	ENSMUSG00000085436	Zfp335os	0.712787878	0.009377281
ENSMUSG00000036188	Ankmy2	-0.629609812	1.36129E-10	ENSMUSG00000044471	Lncpint	0.712107399	0.036702757
ENSMUSG00000003623	Crot	-0.630332129	4.18797E-21	ENSMUSG00000066880	Zfp617	0.711616244	6.83954E-18
ENSMUSG00000017776	Crk	-0.630352119	8.9933E-123	ENSMUSG00000039405	Prss23	0.711255489	2.31952E-98
ENSMUSG00000039458	Mtmr12	-0.630579149	1.33359E-19	ENSMUSG00000055452	Gm7353	0.710836401	0.002250578
ENSMUSG00000028312	Smc2	-0.630611618	1.74719E-65	ENSMUSG00000069135	Fgfr1op	0.71018439	6.13426E-39
ENSMUSG00000026088	Mitd1	-0.631073793	4.57388E-09	ENSMUSG00000006494	Pdk1	0.709994633	6.93388E-16
ENSMUSG00000030275	Etnk1	-0.631141266	1.08251E-47	ENSMUSG00000058388	Phtf1	0.709955715	1.4466E-33
ENSMUSG00000028581	Laptm5	-0.632248084	0.019773337	ENSMUSG00000041939	Mvk	0.709568483	2.77536E-22
ENSMUSG00000062190	Lancl2	-0.632257047	1.0709E-10	ENSMUSG00000032487	Ptgs2	0.709351228	1.0212E-77
ENSMUSG00000036941	Elac1	-0.63242146	3.18978E-07	ENSMUSG00000031273	Col4a6	0.708064325	1.76775E-46
ENSMUSG00000015478	Rnf5	-0.632919933	1.08328E-13	ENSMUSG00000056457	Prl2c3	0.70774786	2.54421E-10
ENSMUSG00000030309	Caprin2	-0.63330967	0.000384822	ENSMUSG00000043311	D17H6S53E	0.706972278	4.79231E-17
ENSMUSG00000037725	Ckap2	-0.633453768	1.01886E-43	ENSMUSG00000006717	Acot13	0.706913835	7.07005E-20
ENSMUSG00000047238	Mageh1	-0.633826013	3.24061E-12	ENSMUSG00000078681	Tm2d3	0.70666689	3.05684E-15
ENSMUSG00000017499	Cdc6	-0.633966831	7.06852E-09	ENSMUSG00000020715	Ern1	0.706340996	5.50385E-77
ENSMUSG00000060600	Eno3	-0.634067482	0.001801171	ENSMUSG00000003955	Fam162a	0.706024945	5.80483E-40
ENSMUSG00000029722	Agfg2	-0.634397424	1.96833E-10	ENSMUSG00000005087	Cd44	0.705786274	4.7496E-160
ENSMUSG00000026826	Nr4a2	-0.634520728	6.30087E-08	ENSMUSG00000038893	Fam117a	0.705637051	5.71817E-08
ENSMUSG00000020649	Rrm2	-0.635442625	7.85353E-49	ENSMUSG00000031749	St3gal2	0.705369216	5.98633E-32
ENSMUSG00000024048	Myl12a	-0.636264042	1.7604E-149	ENSMUSG00000050708	Ftl1	0.704679828	4.357E-107
ENSMUSG00000047420	Fam180a	-0.636523204	0.000499959	ENSMUSG00000063558	Aox1	0.70430919	3.80161E-20
ENSMUSG00000050103	Agmo	-0.636928873	2.68039E-06	ENSMUSG00000055723	Rras2	0.704119952	3.20101E-84
ENSMUSG00000041936	Agrn	-0.637016624	3.27572E-12	ENSMUSG00000095990	Zfp97	0.703935984	1.54055E-05
ENSMUSG00000015981	Stk32c	-0.63743449	0.022726732	ENSMUSG00000118346	Tmem179b	0.703835258	1.3749E-07
ENSMUSG00000029669	Tspan12	-0.639020969	4.92135E-21	ENSMUSG00000117869	Snhg4	0.703531842	1.42248E-13
ENSMUSG00000022021	Diaph3	-0.639137722	2.77011E-48	ENSMUSG00000039497	Dse	0.703354968	1.83595E-65
ENSMUSG00000020657	Dnajc27	-0.639336982	5.13753E-10	ENSMUSG00000028879	Stx12	0.703165361	6.09568E-70
ENSMUSG00000020882	Cacnb1	-0.640405257	0.03248374	ENSMUSG00000040843	Tiprl	0.702740167	1.19505E-38
ENSMUSG00000033970	Rfc3	-0.640672221	7.89203E-09	ENSMUSG00000024505	Dtwd2	0.701946241	0.000821944
ENSMUSG00000032238	Rora	-0.640687886	1.30981E-32	ENSMUSG00000078894	2210418O10Rik	0.701095977	0.000855318
ENSMUSG00000055493	Epm2a	-0.640918888	0.03783609	ENSMUSG00000097068	Gm26760	0.700368664	0.032184937
ENSMUSG00000031642	Sh3rf1	-0.641242652	9.08131E-30	ENSMUSG00000004393	Ddx56	0.700086573	7.54483E-28
ENSMUSG00000017718	Afmid	-0.64142167	0.019889945	ENSMUSG00000027076	Timm10	0.698706137	4.76111E-08
ENSMUSG00000060261	Gtf2i	-0.64199459	1.61322E-51	ENSMUSG00000024924	Vldlr	0.698668534	4.22746E-10

ENSMUSG00000090210	Itga10	-0.642155632	0.006355015	ENSMUSG00000002289	Angptl4	0.698086945	2.7407E-126
ENSMUSG00000028032	Papss1	-0.642244213	1.04571E-31	ENSMUSG00000109903	Gm28710	0.697965806	0.037067111
ENSMUSG00000026179	Pnkd	-0.643522054	1.05828E-13	ENSMUSG00000054942	Miga1	0.696605227	5.02266E-13
ENSMUSG00000034854	Mfsd12	-0.643904545	2.01058E-06	ENSMUSG00000021102	Glrx5	0.696088375	2.07864E-40
ENSMUSG00000035967	Ints6l	-0.644007585	1.22703E-06	ENSMUSG00000027208	Fgf7	0.695902102	9.6472E-54
ENSMUSG00000041354	Rgl2	-0.644380427	1.11622E-22	ENSMUSG00000062210	Tnfaip8	0.695147687	6.331E-44
ENSMUSG00000026576	Atp1b1	-0.64439352	5.81754E-42	ENSMUSG00000029146	Snx17	0.694831869	1.57973E-79
ENSMUSG00000090213	Tmem189	-0.64456065	9.06917E-36	ENSMUSG00000035757	Selenoo	0.694582942	3.8661E-19
ENSMUSG00000047583	Tyw3	-0.644869221	0.006613755	ENSMUSG00000042870	Tom1	0.694372622	1.10421E-40
ENSMUSG00000041126	H2az2	-0.645017926	1.43883E-15	ENSMUSG00000021891	Mettl6	0.69433768	1.64982E-45
ENSMUSG00000061665	Cd2ap	-0.645085962	1.19653E-83	ENSMUSG00000033102	Cdc14b	0.693053876	1.99615E-27
ENSMUSG00000009569	Mrtfb	-0.645591817	9.61359E-39	ENSMUSG00000074829	2010315B03Rik	0.692882015	4.04079E-07
ENSMUSG00000045205	Dpy19l4	-0.645851454	3.76103E-36	ENSMUSG00000021876	Rnase4	0.692876365	6.09833E-85
ENSMUSG00000038481	Cdk19	-0.646079349	4.32168E-18	ENSMUSG00000031150	Ccdc120	0.692674601	0.000341587
ENSMUSG00000030276	Ttll3	-0.646212179	5.8665E-05	ENSMUSG00000035064	Eef2k	0.692474051	2.79732E-14
ENSMUSG00000021572	Cep72	-0.646854264	0.000115357	ENSMUSG00000000326	Comt	0.691908998	4.81053E-60
ENSMUSG00000056531	Ccdc18	-0.64755948	0.037044469	ENSMUSG00000025277	Abhd6	0.691521568	1.08778E-06
ENSMUSG00000026683	Nuf2	-0.647953355	3.12871E-26	ENSMUSG00000046982	Tshz1	0.690359034	4.41426E-26
ENSMUSG00000034573	Ptpn13	-0.648220126	8.45196E-14	ENSMUSG00000056121	Fez2	0.690257884	9.78495E-45
ENSMUSG00000005465	Il27ra	-0.648589238	1.68851E-05	ENSMUSG00000020376	Rnf130	0.689349319	1.34604E-43
ENSMUSG00000043079	Synpo	-0.648609767	5.15638E-91	ENSMUSG00000116995	Gm21926	0.689132142	8.37879E-08
ENSMUSG00000027811	4930579G24Rik	-0.64922268	5.04773E-10	ENSMUSG00000036452	Arhgap26	0.688698147	0.00018503
ENSMUSG00000009246	Trpm5	-0.6492666	0.014289527	ENSMUSG00000024862	Klc2	0.687767943	8.28985E-27
ENSMUSG00000078515	Ddi2	-0.649279571	2.3219E-63	ENSMUSG00000035235	Trim13	0.68737679	3.4344E-07
ENSMUSG00000043336	Filip1l	-0.649999641	4.92166E-65	ENSMUSG00000042349	Ikbke	0.687011806	4.72674E-45
ENSMUSG00000044433	Camsap3	-0.650280646	0.004732659	ENSMUSG00000033793	Atp6v1h	0.686795502	8.8434E-99
ENSMUSG00000039968	Rsbn1l	-0.650659113	2.83501E-23	ENSMUSG00000016386	Mpped2	0.686355743	0.000314024
ENSMUSG00000002031	Ift46	-0.650664608	2.05697E-24	ENSMUSG00000032294	Pkm	0.685406345	1.3952E-247
ENSMUSG00000050107	Haspin	-0.65121733	1.11473E-06	ENSMUSG00000090958	Lrrc32	0.685255285	2.282E-191
ENSMUSG00000005357	Slc1a6	-0.651279177	3.16827E-06	ENSMUSG00000019846	Lama4	0.684451403	1.8023E-120
ENSMUSG00000035164	Zc3h12c	-0.651279837	3.71564E-09	ENSMUSG00000062480	Acat3	0.684353304	8.62465E-06
ENSMUSG00000066191	Anks6	-0.652259669	1.68725E-07	ENSMUSG00000029621	Arpc1a	0.684301104	2.56643E-53
ENSMUSG00000050777	Tmem37	-0.652815935	4.44251E-13	ENSMUSG00000024132	Eci1	0.68404976	1.1365E-17
ENSMUSG00000029516	Cit	-0.653994764	4.99244E-14	ENSMUSG00000005506	Celf1	0.683926895	2.17821E-95
ENSMUSG00000029687	Ezh2	-0.655432467	2.01168E-24	ENSMUSG00000002658	Gtf2f1	0.683916096	1.24523E-74
ENSMUSG00000031669	Gins3	-0.655782789	0.00018704	ENSMUSG00000030816	Rnf40	0.683439506	3.49357E-47
ENSMUSG00000024054	Smchd1	-0.656074954	3.31891E-28	ENSMUSG00000025026	Add3	0.683348136	4.68032E-40
ENSMUSG00000027803	Wwtr1	-0.656591051	7.09496E-98	ENSMUSG00000033124	Atg9a	0.683141456	2.56094E-31
ENSMUSG00000027330	Cdc25b	-0.656988271	1.02114E-23	ENSMUSG00000022629	Kif21a	0.682848416	3.17754E-18
ENSMUSG00000034731	Dgkh	-0.657885464	4.4702E-22	ENSMUSG00000022052	Ppp2r2a	0.682377795	3.74967E-72
ENSMUSG00000055866	Per2	-0.658057561	5.15202E-05	ENSMUSG00000026820	Ptges2	0.682375431	9.00244E-22
ENSMUSG00000055633	Zfp580	-0.65810961	0.007358433	ENSMUSG00000014243	Zswim7	0.68205307	4.06149E-05
ENSMUSG00000034349	Smc4	-0.65884971	1.10228E-59	ENSMUSG00000055963	Triqk	0.682012321	2.57099E-07
ENSMUSG00000047090	Tmem198b	-0.659000221	1.14794E-11	ENSMUSG00000006390	Elovl1	0.681981386	8.18197E-74
ENSMUSG00000067889	Sptbn2	-0.659291678	1.52487E-05	ENSMUSG00000020160	Meis1	0.681361575	5.52105E-24
ENSMUSG00000020102	Slc16a7	-0.661082188	0.037499198	ENSMUSG00000082062	Ftl2-ps	0.679666966	2.9963E-05
ENSMUSG00000054967	Zfp647	-0.661174842	0.015515762	ENSMUSG00000020448	Rnf185	0.678971789	8.21303E-47
ENSMUSG00000006678	Pola1	-0.661202324	2.8825E-24	ENSMUSG00000035776	Cd99l2	0.678930628	9.83015E-50
ENSMUSG00000002325	Irf9	-0.661373512	1.47047E-12	ENSMUSG00000034343	Ube2f	0.678358595	2.28014E-56
ENSMUSG00000038379	Ttk	-0.661418681	6.13673E-13	ENSMUSG00000020064	Herc4	0.677311506	2.51448E-44
ENSMUSG00000087060	Eldr	-0.662137392	0.031739522	ENSMUSG00000020250	Txnrd1	0.677197485	1.9E-118
ENSMUSG00000043015	Nemp2	-0.662445002	0.000745185	ENSMUSG00000031782	Coq9	0.677197391	3.73661E-14
ENSMUSG00000031216	Stard8	-0.662649825	2.76784E-22	ENSMUSG00000023456	Tpi1	0.677191022	1.06054E-76
ENSMUSG00000010175	Prox1	-0.663185736	0.0013244	ENSMUSG00000029761	Cald1	0.677165256	1.5906E-126
ENSMUSG00000047735	Samd9l	-0.663622147	6.53715E-06	ENSMUSG00000050668	Gpatch11	0.677086899	5.72578E-11
ENSMUSG00000049044	Rapgef4	-0.663760642	3.05298E-05	ENSMUSG00000020604	Arsg	0.676637107	3.4958E-09
ENSMUSG00000021707	Dhfr	-0.664463596	7.51945E-17	ENSMUSG00000057614	Gnai1	0.676377843	2.00774E-30
ENSMUSG00000001403	Ube2c	-0.664983907	1.16476E-31	ENSMUSG00000030272	Camk1	0.675668484	1.18142E-76
ENSMUSG00000032038	St3gal4	-0.665320112	1.24222E-33	ENSMUSG00000023947	Nfkbie	0.67537985	1.72566E-08
ENSMUSG00000024067	Dpy30	-0.665423414	2.97832E-11	ENSMUSG00000018507	Trpv2	0.675141634	6.05566E-09
ENSMUSG00000060441	Trim5	-0.665564505	0.001323401	ENSMUSG00000064372	mt-Tp	0.674684846	2.43682E-05
ENSMUSG00000027358	Bmp2	-0.666634814	0.001108203	ENSMUSG00000051989	Smim11	0.674006085	2.20133E-08
ENSMUSG00000042606	Hirip3	-0.666677129	3.88009E-08	ENSMUSG00000107002	0610012G03Rik	0.673902471	6.74674E-15
ENSMUSG00000032387	Rbpms2	-0.667267334	4.80171E-14	ENSMUSG00000060733	Ipmk	0.672945326	1.70721E-26
ENSMUSG00000014361	Mertk	-0.667961765	1.69577E-36	ENSMUSG00000029553	Tfec	0.672725751	3.74212E-33
ENSMUSG00000038022	Mindy4	-0.668419613	1.47318E-05	ENSMUSG00000059423	Zfp933	0.672317001	3.78545E-09
ENSMUSG00000034165	Ccnd3	-0.668625021	7.97314E-50	ENSMUSG00000055116	Arntl	0.672118112	2.08666E-38
ENSMUSG00000051483	Cbr1	-0.66889774	4.38166E-11	ENSMUSG00000079499	6530402F18Rik	0.671589289	1.8775E-13
ENSMUSG00000030554	Synm	-0.669063635	0.001673138	ENSMUSG00000093577	Gm20632	0.671492736	1.04268E-08
ENSMUSG00000025903	Lypla1	-0.669226052	2.3692E-56	ENSMUSG00000038546	Ranbp9	0.670917931	1.99575E-47
ENSMUSG00000024501	Dpysl3	-0.669234127	7.47662E-67	ENSMUSG00000042367	Gjb3	0.67047455	4.08322E-14
ENSMUSG00000002346	Slc25a42	-0.669301492	1.84148E-05	ENSMUSG00000029762	Akr1b8	0.670473438	1.01793E-53
ENSMUSG00000039043	Arpin	-0.669528319	1.41525E-22	ENSMUSG00000022992	Kansl2	0.669953607	4.50241E-70
ENSMUSG00000024228	Nudt12	-0.670603302	0.002530499	ENSMUSG00000032127	Vps11	0.669829569	5.62695E-47
ENSMUSG00000038451	Spsb2	-0.671535222	4.58817E-07	ENSMUSG00000020701	Tmem132e	0.669645629	0.015055878
ENSMUSG00000039903	Eva1c	-0.67176718	0.000293531	ENSMUSG00000032872	Cyb5r4	0.669594393	1.2578E-40
ENSMUSG00000048450	Msx1	-0.671793734	5.19688E-05	ENSMUSG00000056501	Cebpb	0.668620899	3.12836E-52

ENSMUSG00000022297	Fzd6	-0.67183759	1.00176E-25	ENSMUSG00000098041	Gm26981	0.667550006	0.010811851
ENSMUSG00000033540	Idua	-0.672060954	7.0555E-14	ENSMUSG00000029201	Ugdh	0.667037734	8.2337E-129
ENSMUSG00000047534	Mis18bp1	-0.672801655	5.47961E-24	ENSMUSG00000057147	Dph6	0.666782074	8.58481E-17
ENSMUSG00000046341	Gm11223	-0.673427358	0.044841297	ENSMUSG00000071655	Ubxn1	0.666513588	1.34016E-43
ENSMUSG00000003545	Fosb	-0.673583947	0.000249688	ENSMUSG00000039004	Bmp6	0.666339535	2.22915E-47
ENSMUSG00000063193	Cd300lb	-0.674066923	8.62391E-07	ENSMUSG00000054611	Kdm2a	0.666198255	4.16024E-53
ENSMUSG00000026678	Rgs5	-0.674144518	0.041068687	ENSMUSG00000061740	Cyp2d22	0.665540385	3.34142E-11
ENSMUSG00000027351	Spred1	-0.674175111	8.4959E-154	ENSMUSG00000021360	Gcnt2	0.665488566	2.8803E-27
ENSMUSG00000071379	Hpcal1	-0.674728185	1.67426E-27	ENSMUSG00000039047	Pigk	0.665474949	2.63755E-55
ENSMUSG00000025268	Maged2	-0.67487291	3.28979E-92	ENSMUSG00000058655	Eif4b	0.665239652	4.76289E-92
ENSMUSG00000019779	Frk	-0.674958964	6.84326E-15	ENSMUSG00000047604	Frat2	0.664498079	0.011276771
ENSMUSG00000045005	Fzd5	-0.67498069	5.06665E-11	ENSMUSG00000021939	Ctsb	0.664482058	2.9474E-171
ENSMUSG00000020897	Aurkb	-0.675633613	4.92483E-15	ENSMUSG00000042814	Mcts2	0.664002128	9.63159E-07
ENSMUSG00000005813	Metap1	-0.676355628	1.73401E-40	ENSMUSG00000038058	Nod1	0.663012026	8.78661E-84
ENSMUSG00000030255	Sspn	-0.676792031	1.15153E-12	ENSMUSG00000021550	2210016F16Rik	0.662990487	2.52289E-28
ENSMUSG00000001227	Sema6b	-0.676825541	9.07796E-06	ENSMUSG00000042660	Wdr55	0.662983146	4.86563E-19
ENSMUSG00000087179	Gm14230	-0.677736994	0.002721812	ENSMUSG00000017428	Psmd11	0.66245199	2.84519E-93
ENSMUSG00000046410	Kcnk6	-0.678376877	1.14016E-08	ENSMUSG00000004668	Abca13	0.661833546	0.004441144
ENSMUSG00000075289	Carns1	-0.679174601	0.005389999	ENSMUSG00000109536	9330162G02Rik	0.660352186	0.031605232
ENSMUSG00000006342	Susd2	-0.679216715	1.33948E-05	ENSMUSG00000043953	Ccrl2	0.660340481	6.39774E-05
ENSMUSG00000042099	Kank3	-0.679425819	0.003978759	ENSMUSG00000035239	Neu3	0.660040643	0.000402527
ENSMUSG00000000708	Kat2b	-0.679971848	5.17687E-45	ENSMUSG00000002602	Axl	0.659811051	5.5488E-182
ENSMUSG00000028414	Fktn	-0.680000576	6.58679E-23	ENSMUSG00000003234	Abcf3	0.65952557	2.51414E-36
ENSMUSG00000024747	Aldh1a7	-0.680393492	0.004965381	ENSMUSG00000009621	Vav2	0.659499954	3.6802E-43
ENSMUSG00000058163	Gm5431	-0.681256867	0.003473099	ENSMUSG00000079092	Prl2c2	0.659158355	4.81289E-23
ENSMUSG00000004698	Hdac9	-0.681439076	0.002332147	ENSMUSG00000031337	Mtm1	0.659058996	2.6471E-17
ENSMUSG00000027242	Wdr76	-0.681524018	2.83548E-06	ENSMUSG00000027834	Serpini1	0.658679298	2.12347E-15
ENSMUSG00000001098	Kctd10	-0.681889241	4.4684E-124	ENSMUSG00000022403	St13	0.657258443	1.63633E-66
ENSMUSG00000027792	Bche	-0.68192339	0.009410646	ENSMUSG00000023286	Ube2j2	0.656506361	2.11777E-44
ENSMUSG00000059022	Kcp	-0.682032877	0.032778942	ENSMUSG00000038708	Golga4	0.656489417	3.57348E-38
ENSMUSG00000001517	Foxm1	-0.682897386	1.90681E-43	ENSMUSG00000078896	Zfp965	0.656399012	0.002887877
ENSMUSG00000038943	Prc1	-0.683112401	6.34139E-70	ENSMUSG00000032068	Plet1	0.655573379	0.038656849
ENSMUSG00000033147	Slc22a15	-0.683913094	2.29391E-12	ENSMUSG00000029804	Herc3	0.655355929	2.58221E-13
ENSMUSG00000042190	Cmklr1	-0.685527369	1.12607E-10	ENSMUSG00000010751	Tnfrsf22	0.655279323	5.09873E-21
ENSMUSG00000030243	Recql	-0.685979698	1.67895E-15	ENSMUSG00000063531	Sema3e	0.655194491	2.03495E-30
ENSMUSG00000036862	Dchs1	-0.686519975	1.96214E-09	ENSMUSG00000064080	Fbln2	0.655042762	4.3739E-164
ENSMUSG00000074922	Fam122a	-0.686919327	2.14427E-05	ENSMUSG00000061981	Flot2	0.654791644	5.98198E-72
ENSMUSG00000037348	Paqr7	-0.686976615	2.57371E-16	ENSMUSG00000109901	Chmp1b	0.654673641	1.3032E-57
ENSMUSG00000042216	Sgsm1	-0.687554426	0.002069722	ENSMUSG00000003865	Gys1	0.654539154	4.96893E-35
ENSMUSG00000037363	Letm2	-0.687636789	0.028942979	ENSMUSG00000001627	Ifrd1	0.653991416	1.21074E-63
ENSMUSG00000039994	Timeless	-0.687691492	3.79306E-09	ENSMUSG00000026623	Lpgat1	0.653645074	2.69881E-62
ENSMUSG00000106951	5930430L01Rik	-0.687701506	0.004155925	ENSMUSG00000033499	Larp4b	0.653557602	8.05527E-78
ENSMUSG00000035929	H2-Q4	-0.687786623	0.011536792	ENSMUSG00000022971	Ifnar2	0.6534585	2.16139E-58
ENSMUSG00000030246	Ldhb	-0.688102541	1.38464E-09	ENSMUSG00000028700	Pomgnt1	0.653112294	2.29071E-50
ENSMUSG00000029910	Mad2l1	-0.688149578	2.91843E-36	ENSMUSG00000032422	Snx14	0.652393744	3.68407E-34
ENSMUSG00000040594	Ranbp17	-0.688566901	7.73981E-06	ENSMUSG00000037225	Fgf2	0.651554928	2.03755E-44
ENSMUSG00000056313	Tcim	-0.688596982	3.21228E-06	ENSMUSG00000069622	Gm10273	0.651349363	0.011555147
ENSMUSG00000021065	Fut8	-0.689225043	1.56771E-28	ENSMUSG00000055991	Zkscan5	0.651263963	1.51224E-09
ENSMUSG00000033350	Chst2	-0.689284496	2.89044E-08	ENSMUSG00000074649	BC029722	0.651193245	1.58351E-34
ENSMUSG00000028476	Reck	-0.689718335	1.09333E-67	ENSMUSG00000021930	Spryd7	0.651066222	1.33618E-13
ENSMUSG00000021066	Atl1	-0.69004524	2.02384E-11	ENSMUSG00000037376	Trmt6	0.650802945	5.93677E-38
ENSMUSG00000036944	Tmem71	-0.691215817	0.01639609	ENSMUSG00000020413	Hus1	0.650794012	2.77709E-10
ENSMUSG00000021536	Adcy2	-0.691348273	0.003431124	ENSMUSG00000028878	Fam76a	0.650682536	1.52231E-36
ENSMUSG00000074476	Spc24	-0.692357865	6.00601E-11	ENSMUSG00000021196	Pfkp	0.650279017	5.29832E-43
ENSMUSG00000025372	Baiap2	-0.69238385	9.31411E-16	ENSMUSG00000078622	Ccdc47	0.65020345	2.90392E-44
ENSMUSG00000041624	Gucy1a2	-0.692592307	0.007473788	ENSMUSG00000004843	Chmp2b	0.649547623	1.09881E-30
ENSMUSG00000021185	Dglucy	-0.692677046	9.99582E-33	ENSMUSG00000032175	Tyk2	0.64907604	7.56367E-27
ENSMUSG00000060639	H4c9	-0.692694096	5.41341E-05	ENSMUSG00000028347	Tmeff1	0.648870247	1.71084E-22
ENSMUSG00000032020	Ubash3b	-0.69277934	1.4375E-59	ENSMUSG00000068742	Cry2	0.648664946	1.8819E-18
ENSMUSG00000072980	Oip5	-0.692873062	3.66708E-06	ENSMUSG00000062352	Itgb1bp1	0.648446349	4.09995E-39
ENSMUSG00000067276	Capn6	-0.693364411	1.81727E-15	ENSMUSG00000050199	Lgr4	0.647975952	6.65977E-16
ENSMUSG00000018583	G3bp1	-0.693569412	3.56361E-95	ENSMUSG00000026864	Hspa5	0.647751852	8.4321E-151
ENSMUSG00000027306	Nusap1	-0.694186295	1.47161E-29	ENSMUSG00000041438	Utp4	0.647188934	5.36298E-29
ENSMUSG00000006800	Sulf2	-0.694284623	8.2467E-81	ENSMUSG00000032826	Ank2	0.647101108	5.14453E-29
ENSMUSG00000068015	Lrch1	-0.694332569	5.52906E-08	ENSMUSG00000038467	Chmp4b	0.647013948	6.00222E-58
ENSMUSG00000000093	Tbx2	-0.69459447	1.50477E-07	ENSMUSG00000022667	Cd200r1	0.646550602	0.005748404
ENSMUSG00000056394	Lig1	-0.694953005	3.026E-41	ENSMUSG00000032044	Rpusd4	0.64652417	2.40536E-14
ENSMUSG00000068101	Cenpm	-0.695094146	0.000772361	ENSMUSG00000047963	Stbd1	0.646158932	4.17959E-23
ENSMUSG00000029521	Chek2	-0.695273274	2.24339E-14	ENSMUSG00000016526	Dyrk3	0.64596535	0.009746825
ENSMUSG00000034023	Fancd2	-0.695841361	3.244E-09	ENSMUSG00000083327	Vcp-rs	0.645823267	0.00011747
ENSMUSG00000106847	Peg13	-0.69587583	3.14563E-12	ENSMUSG00000072774	Zfp951	0.645234668	0.002407514
ENSMUSG00000027115	Kif18a	-0.696011777	1.55462E-10	ENSMUSG00000022529	Zfp263	0.645021045	1.27449E-18
ENSMUSG00000066877	Nck2	-0.696222176	4.27411E-13	ENSMUSG00000006299	Aamp	0.644923829	1.86854E-74
ENSMUSG00000022385	Gtse1	-0.697459152	7.63435E-36	ENSMUSG00000059554	Ccdc28a	0.644835026	0.000341256
ENSMUSG00000062510	Nsl1	-0.698033034	6.47521E-09	ENSMUSG00000042607	Asb4	0.644676888	4.14232E-06
ENSMUSG00000041406	BC055324	-0.698483262	3.19969E-06	ENSMUSG00000078879	Zfp973	0.644599681	0.002627345
ENSMUSG00000032112	Trappc4	-0.699288236	3.66184E-26	ENSMUSG00000019362	D8Ertd738e	0.644441014	8.85604E-28

ENSMUSG00000011148	Adssl1	-0.699429117	0.046313942
ENSMUSG00000025103	Btbd1	-0.699523099	4.75303E-60
ENSMUSG00000056216	Cebpg	-0.699642281	1.24997E-38
ENSMUSG00000035365	Parpbp	-0.700002979	4.82246E-12
ENSMUSG00000027679	Dnajc19	-0.700207912	3.2467E-13
ENSMUSG00000037447	Arid5a	-0.700597828	6.19236E-18
ENSMUSG00000028132	Tlcd4	-0.700655108	1.2581E-05
ENSMUSG00000024989	Cep55	-0.700753361	3.21606E-40
ENSMUSG00000018849	Wwc1	-0.701091657	6.26276E-16
ENSMUSG00000026211	Obsl1	-0.701508297	3.20539E-41
ENSMUSG00000068744	Psrc1	-0.702071003	5.56356E-30
ENSMUSG00000022422	Dscc1	-0.702161104	0.012284858
ENSMUSG00000042284	Itga1	-0.702423491	8.97539E-44
ENSMUSG00000019942	Cdk1	-0.702890879	6.47596E-64
ENSMUSG00000041431	Ccnb1	-0.703090507	6.91676E-82
ENSMUSG00000030924	Rexo5	-0.703117297	1.32152E-05
ENSMUSG00000014426	Map3k4	-0.703399389	3.61796E-20
ENSMUSG00000030761	Myo7a	-0.703899861	2.07903E-39
ENSMUSG00000060519	Tor3a	-0.703967118	1.25085E-05
ENSMUSG00000025213	Kazald1	-0.704187969	0.01018512
ENSMUSG00000017716	Birc5	-0.704238653	6.5812E-30
ENSMUSG00000002227	Mov10	-0.704562374	2.55347E-16
ENSMUSG00000032220	Myo1e	-0.705457388	1.56908E-36
ENSMUSG00000041926	Rnpep	-0.706226593	2.49974E-41
ENSMUSG00000030528	Blm	-0.706403218	9.18698E-11
ENSMUSG00000028245	Nsmaf	-0.707113725	9.51358E-37
ENSMUSG00000025001	Hells	-0.707675341	2.04864E-27
ENSMUSG00000054252	Fgfr3	-0.707933043	0.007132872
ENSMUSG00000106928	Gm43860	-0.708177159	0.004721167
ENSMUSG00000061111	Mcrip1	-0.708585789	1.98755E-12
ENSMUSG00000034601	2700049A03Rik	-0.709669015	2.02947E-08
ENSMUSG00000033768	Nrxn2	-0.709740833	0.002489052
ENSMUSG00000034842	Art3	-0.710172325	7.88556E-06
ENSMUSG00000004105	Angptl2	-0.711008165	2.80358E-71
ENSMUSG00000074064	Mlycd	-0.711491594	9.26647E-11
ENSMUSG00000031885	Cbfb	-0.711568169	1.07566E-89
ENSMUSG00000046572	Zfp518b	-0.711649584	6.30557E-28
ENSMUSG00000040455	Usp45	-0.711967751	1.07753E-36
ENSMUSG00000032400	Zwilch	-0.712377302	3.00998E-23
ENSMUSG00000089715	Cbx6	-0.712498678	1.06877E-30
ENSMUSG00000031304	Il2rg	-0.712557882	0.004791832
ENSMUSG00000079014	Serpina3i	-0.712996113	1.76408E-10
ENSMUSG00000020108	Ddit4	-0.713691774	8.49611E-40
ENSMUSG00000054115	Skp2	-0.713914145	3.54297E-18
ENSMUSG00000098098	Bvht	-0.714094704	0.000544876
ENSMUSG00000024511	Rab27b	-0.71461759	1.38983E-07
ENSMUSG00000098934	Gm18853	-0.71497123	0.002845497
ENSMUSG00000036777	Anln	-0.715194933	9.34657E-68
ENSMUSG00000039813	Tbc1d2	-0.715701697	4.15327E-05
ENSMUSG00000036181	H1f2	-0.71603576	5.31477E-34
ENSMUSG00000010051	Hyal1	-0.716170478	5.48456E-05
ENSMUSG00000032766	Gng11	-0.716305423	1.96177E-15
ENSMUSG00000038252	Ncapd2	-0.717053926	1.14416E-48
ENSMUSG00000042793	Lgr6	-0.71800289	7.49754E-14
ENSMUSG00000024056	Ndc80	-0.718580573	7.88784E-24
ENSMUSG00000046806	Cyren	-0.71892119	3.0473E-06
ENSMUSG00000042607	Asb4	-0.719158718	4.00052E-05
ENSMUSG00000047793	Sned1	-0.719505775	6.76705E-06
ENSMUSG00000063889	Crem	-0.719631073	3.27187E-14
ENSMUSG00000038349	Plcl1	-0.71971945	6.51727E-05
ENSMUSG00000042129	Rassf4	-0.719957014	0.034100211
ENSMUSG00000020160	Meis1	-0.720656324	2.45523E-18
ENSMUSG00000051451	Crebzf	-0.720829323	1.38878E-44
ENSMUSG00000042599	Kdm7a	-0.721057278	1.00604E-20
ENSMUSG00000112980	D430020J02Rik	-0.72127957	5.97103E-09
ENSMUSG00000061013	Mkx	-0.721551817	0.005889284
ENSMUSG00000052364	B630019K06Rik	-0.721937481	0.000166124
ENSMUSG00000072082	Ccnf	-0.722138159	2.5237E-33
ENSMUSG00000046916	Myct1	-0.723549021	1.03842E-06
ENSMUSG00000037887	Dusp8	-0.723577091	1.26277E-20
ENSMUSG00000039396	Neil3	-0.72399531	2.80546E-09
ENSMUSG00000041219	Arhgap11a	-0.724177954	1.8475E-45
ENSMUSG00000035268	Pkig	-0.724556902	2.0765E-27
ENSMUSG00000075528	Aarsd1	-0.724667499	1.79095E-12
ENSMUSG00000041907	Gpr45	-0.724982302	0.021669618
ENSMUSG00000055044	Pdlim1	-0.725368079	2.64485E-54
ENSMUSG00000020453	Patz1	-0.725762953	6.73403E-14

ENSMUSG00000029428	Stx2	0.64403044	4.32004E-68
ENSMUSG00000037242	Clic4	0.643967984	1.3123E-120
ENSMUSG00000035105	Egln3	0.642839773	1.9355E-19
ENSMUSG00000072115	Ang	0.642588549	1.60901E-08
ENSMUSG00000040006	Ginm1	0.642431552	3.32968E-40
ENSMUSG00000105741	C79130	0.642229013	0.020988884
ENSMUSG00000028093	Acp6	0.641843672	3.1415E-06
ENSMUSG00000050930	Map10	0.641575326	0.006156547
ENSMUSG00000016308	Ube2a	0.641199211	3.01072E-24
ENSMUSG00000031767	Nudt7	0.640861565	1.58765E-05
ENSMUSG00000051169	Rpusd3	0.640622729	0.007621441
ENSMUSG00000017491	Rarb	0.640587169	0.000373766
ENSMUSG00000035713	Usp35	0.640262144	3.78782E-06
ENSMUSG00000040563	Plppr2	0.639697054	3.09717E-07
ENSMUSG00000078517	Emc1	0.639132833	1.99157E-37
ENSMUSG00000022895	Ets2	0.639005017	4.93575E-29
ENSMUSG00000087590	Epb41l4aos	0.638922364	0.004716656
ENSMUSG00000030314	Atg7	0.638831845	1.571E-44
ENSMUSG00000003458	Ncstn	0.638666727	9.40458E-98
ENSMUSG00000051391	Ywhag	0.638542206	1.652E-130
ENSMUSG00000016382	Pls3	0.638478973	1.1276E-100
ENSMUSG00000024169	Ift140	0.637855259	6.7765E-14
ENSMUSG00000023055	Calcoco1	0.637478876	6.32626E-36
ENSMUSG00000023988	Bysl	0.637154373	3.24526E-32
ENSMUSG00000017132	Cyth1	0.636824238	4.03999E-21
ENSMUSG00000029458	Brap	0.636448838	3.49863E-36
ENSMUSG00000056458	Mok	0.635685535	0.019320434
ENSMUSG00000029007	Agtrap	0.634945955	1.56799E-32
ENSMUSG00000021890	Eaf1	0.634300955	3.65E-109
ENSMUSG00000029642	Polr1d	0.634228264	7.37393E-48
ENSMUSG00000032548	Slco2a1	0.633283365	2.9337E-129
ENSMUSG00000042675	Ypel3	0.63282856	9.53652E-08
ENSMUSG00000033629	Hacd3	0.632631668	6.95353E-21
ENSMUSG00000038605	Samd10	0.632515644	0.011515857
ENSMUSG00000038485	Socs7	0.632061516	2.02859E-21
ENSMUSG00000037613	Tnfrsf23	0.631578587	4.63024E-70
ENSMUSG00000042015	Wdr41	0.630406544	1.42181E-25
ENSMUSG00000039242	B3galnt2	0.629792834	1.17222E-28
ENSMUSG00000069844	Sco1	0.629603039	8.28362E-13
ENSMUSG00000025776	Crispld1	0.629596645	0.027382871
ENSMUSG00000029207	Apbb2	0.628599623	1.34961E-35
ENSMUSG00000020495	Smg8	0.628062826	4.85278E-24
ENSMUSG00000029465	Arpc3	0.627170286	5.48028E-33
ENSMUSG00000007812	Zfp655	0.626704483	2.5892E-32
ENSMUSG00000024826	Dpf2	0.62643384	8.10706E-35
ENSMUSG00000074634	Tmem267	0.626334502	7.70831E-07
ENSMUSG00000093752	Gm20716	0.625813223	0.009839796
ENSMUSG00000031519	Asb5	0.625798915	5.10125E-60
ENSMUSG00000049680	Urgcp	0.62553075	3.3798E-23
ENSMUSG00000029291	Rufy3	0.625154946	8.57883E-14
ENSMUSG00000085741	5430405H02Rik	0.624680074	0.033316778
ENSMUSG00000036390	Gadd45a	0.624061748	1.1662E-16
ENSMUSG00000042165	Gm9774	0.624040592	0.011604276
ENSMUSG00000027423	Snx5	0.622961505	5.93748E-70
ENSMUSG00000069670	Nkain2	0.622565256	0.002261763
ENSMUSG00000046058	Eid2	0.622367558	3.45563E-09
ENSMUSG00000057156	Homez	0.6217813	2.08851E-05
ENSMUSG00000042677	Zc3h12a	0.62044065	8.53133E-62
ENSMUSG00000019433	Gipc1	0.620324353	8.05413E-47
ENSMUSG00000047030	Spata2	0.620291934	7.82983E-22
ENSMUSG00000002345	Borcs8	0.62023291	7.03066E-13
ENSMUSG00000024901	Peli3	0.620201444	0.011208631
ENSMUSG00000073676	Hspe1	0.620015336	3.30663E-58
ENSMUSG00000032892	Rangrf	0.618706934	0.000690711
ENSMUSG00000024130	Abca3	0.618594474	1.68636E-31
ENSMUSG00000079834	Tmlhe	0.618446152	5.36708E-21
ENSMUSG00000063659	Zbtb18	0.61827942	4.74099E-22
ENSMUSG00000026796	Fam129b	0.618113497	3.1988E-146
ENSMUSG00000026426	Arl8a	0.61701586	2.36519E-48
ENSMUSG00000038240	Pdss2	0.616926354	8.21424E-07
ENSMUSG00000040859	Bsdc1	0.616441743	4.65738E-36
ENSMUSG00000029552	Tes	0.616439384	1.47066E-22
ENSMUSG00000074158	Zfp976	0.616338474	8.36844E-06
ENSMUSG00000063410	Stk24	0.615850905	1.19397E-28
ENSMUSG00000053470	Kdm3a	0.615635416	2.10306E-36
ENSMUSG00000029223	Uchl1	0.615471885	1.3324E-32
ENSMUSG00000004455	Ppp1cc	0.615334571	2.84785E-70

ENSMUSG00000020914	Top2a	-0.72631657	3.27186E-87	ENSMUSG00000057531	Dtnbp1	0.615229609	5.34503E-41
ENSMUSG00000029228	Lnx1	-0.726490322	0.001479772	ENSMUSG00000029009	Mthfr	0.613753083	3.31926E-43
ENSMUSG00000022508	Bcl6	-0.72660398	3.35241E-12	ENSMUSG00000022111	Uchl3	0.613692622	4.10736E-16
ENSMUSG00000033762	Recql4	-0.727252711	0.00097988	ENSMUSG00000031429	Psmd10	0.613623121	2.09145E-24
ENSMUSG00000115149	9330188P03Rik	-0.727254631	1.20691E-10	ENSMUSG00000003546	Klc4	0.613529028	1.46386E-08
ENSMUSG00000038143	Stox2	-0.728736992	2.48765E-27	ENSMUSG00000034501	Pcnx4	0.613260991	4.35427E-14
ENSMUSG00000002307	Daxx	-0.728755036	1.04911E-05	ENSMUSG00000057738	Sptan1	0.613233888	1.06538E-89
ENSMUSG00000035329	Fbxo33	-0.729302129	6.69564E-20	ENSMUSG00000027257	Pacsin3	0.612975651	6.43756E-11
ENSMUSG00000038319	Kcnh2	-0.729628588	7.88591E-05	ENSMUSG00000085795	Zfp703	0.612843657	6.50839E-24
ENSMUSG00000043391	2510009E07Rik	-0.729806431	1.6978E-28	ENSMUSG00000033721	Vav3	0.611818482	6.49902E-06
ENSMUSG00000024486	Hbegf	-0.731207394	1.02973E-74	ENSMUSG00000066900	Suds3	0.611726654	4.38252E-40
ENSMUSG00000030844	Rgs10	-0.731728267	1.99788E-10	ENSMUSG00000025161	Slc16a3	0.611249364	1.44064E-14
ENSMUSG00000020523	Fam114a2	-0.732436013	4.45986E-32	ENSMUSG00000029560	Snx8	0.611165418	1.64229E-36
ENSMUSG00000047368	Abhd17b	-0.732490026	2.46776E-27	ENSMUSG00000034928	Rnf44	0.610238644	3.28595E-68
ENSMUSG00000034780	B3galt1	-0.732540825	1.55331E-07	ENSMUSG00000031776	Arl2bp	0.610071334	6.26006E-54
ENSMUSG00000007080	Pole	-0.733294542	2.62497E-23	ENSMUSG00000050234	Gja4	0.610035065	2.2202E-07
ENSMUSG00000043518	Rai2	-0.733346321	0.039855298	ENSMUSG00000022519	Srl	0.609991842	0.044867486
ENSMUSG00000025931	Paqr8	-0.733658507	8.37137E-10	ENSMUSG00000026872	Zeb2	0.609765311	1.00368E-72
ENSMUSG00000070643	Sox13	-0.735246678	1.69166E-13	ENSMUSG00000011752	Pgam1	0.60963531	9.2685E-112
ENSMUSG00000027580	Helz2	-0.735455428	1.24714E-06	ENSMUSG00000029640	Usp12	0.609504341	6.04089E-75
ENSMUSG00000024521	Pmaip1	-0.73550661	1.2488E-15	ENSMUSG00000058317	Ube2e2	0.609393767	4.86504E-22
ENSMUSG00000006418	Rnf114	-0.735864294	2.35408E-37	ENSMUSG00000032657	Fam189b	0.609187425	2.46863E-13
ENSMUSG00000029177	Cenpa	-0.736888438	1.14657E-34	ENSMUSG00000021635	Rad17	0.60903994	5.63179E-14
ENSMUSG00000026355	Mcm6	-0.737752403	1.08406E-63	ENSMUSG00000002763	Pex6	0.608992523	2.56173E-10
ENSMUSG00000031840	Rab3a	-0.738014167	2.55365E-07	ENSMUSG00000005069	Pex5	0.608503827	1.26009E-43
ENSMUSG00000027555	Car13	-0.738515446	6.18982E-45	ENSMUSG00000024228	Nudt12	0.60844513	0.000341116
ENSMUSG00000004319	Clcn3	-0.738608607	2.57569E-31	ENSMUSG00000031255	Sytl4	0.608206647	8.8982E-08
ENSMUSG00000004099	Dnmt1	-0.739273412	4.9348E-49	ENSMUSG00000026678	Rgs5	0.607902657	0.024538841
ENSMUSG00000009739	Pou6f1	-0.740011344	1.78863E-11	ENSMUSG00000021575	Ahrr	0.607557929	0.010689273
ENSMUSG00000039542	Ncam1	-0.740179821	4.59213E-24	ENSMUSG00000042632	Pla2g6	0.607469793	2.69069E-23
ENSMUSG00000026708	Cenpl	-0.740339063	4.73326E-10	ENSMUSG00000019256	Ahr	0.606791019	5.7352E-26
ENSMUSG00000026768	Itga8	-0.740823209	1.02591E-06	ENSMUSG00000021028	Mbip	0.606706141	2.00773E-08
ENSMUSG00000096210	H1f0	-0.741216861	4.15859E-96	ENSMUSG00000024376	Epb41l4a	0.606247485	0.002044138
ENSMUSG00000047888	Tnrc6b	-0.742046478	2.62173E-23	ENSMUSG00000042035	Igsf3	0.606080359	1.79294E-30
ENSMUSG00000028698	Pik3r3	-0.743235243	5.80974E-27	ENSMUSG00000082016	Pgam1-ps2	0.605797902	2.5809E-15
ENSMUSG00000069769	Msi2	-0.743905803	1.59259E-94	ENSMUSG00000038712	Mindy1	0.605486803	2.30849E-40
ENSMUSG00000018427	Ypel2	-0.743907203	8.71811E-12	ENSMUSG00000070730	Rmdn3	0.605016815	8.38433E-15
ENSMUSG00000028341	Nr4a3	-0.744173961	1.54007E-10	ENSMUSG00000019818	Cd164	0.60426692	5.63271E-91
ENSMUSG00000012443	Kif11	-0.744326729	1.37528E-38	ENSMUSG00000078867	Gm14418	0.603949056	0.006482421
ENSMUSG00000015880	Ncapg	-0.744567677	9.72078E-23	ENSMUSG00000034266	Batf	0.603788462	0.00981217
ENSMUSG00000022803	Popdc2	-0.744705758	5.22546E-08	ENSMUSG00000003437	Paf1	0.603721307	2.30966E-38
ENSMUSG00000066357	Wdr6	-0.744945531	2.24055E-76	ENSMUSG00000072946	Ptgr2	0.60337844	1.27125E-22
ENSMUSG00000015340	Cybb	-0.745582466	0.020606728	ENSMUSG00000024978	Gpam	0.603311783	6.38307E-21
ENSMUSG00000036764	Dnajc12	-0.7456685	0.002529599	ENSMUSG00000024085	Man2a1	0.603269983	1.7842E-107
ENSMUSG00000066258	Trim12a	-0.74625245	2.63481E-12	ENSMUSG00000031732	Phlpp2	0.602892369	4.39897E-16
ENSMUSG00000097325	Gm16897	-0.746435576	0.007247829	ENSMUSG00000027004	Frzb	0.602733574	6.944E-106
ENSMUSG00000040913	Fbxw4	-0.746436919	1.06929E-09	ENSMUSG00000031617	Tmem184c	0.602481103	3.25598E-24
ENSMUSG00000025742	Prps2	-0.746762662	1.17635E-45	ENSMUSG00000062081	Gm6055	0.602464184	0.011352327
ENSMUSG00000022421	Nptxr	-0.747267808	5.88E-05	ENSMUSG00000051510	Mafg	0.602331725	1.63412E-48
ENSMUSG00000029098	Acox3	-0.747343976	1.48523E-15	ENSMUSG00000026944	Abca2	0.602218104	2.57638E-11
ENSMUSG00000053870	Fpgt	-0.747374901	7.26287E-18	ENSMUSG00000022800	Fyttd1	0.600813276	1.75851E-42
ENSMUSG00000061411	Nol4l	-0.747609002	1.58911E-07	ENSMUSG00000048796	Cyb561d1	0.600371858	4.12797E-07
ENSMUSG00000020802	Ube2o	-0.747957895	4.51947E-39	ENSMUSG00000014668	Chfr	0.600161709	1.85493E-50
ENSMUSG00000029771	Irf5	-0.748118567	8.40894E-05	ENSMUSG00000032312	Csk	0.599790472	7.24229E-44
ENSMUSG00000022150	Dab2	-0.748161293	1.67778E-94	ENSMUSG00000090564	A430057M04Rik	0.599642103	0.007895362
ENSMUSG00000036585	Fgf1	-0.748388887	0.001739809	ENSMUSG00000027942	4933434E20Rik	0.599340983	2.21372E-15
ENSMUSG00000026048	Ercc5	-0.74894366	2.49841E-39	ENSMUSG00000097287	D130017N08Rik	0.599188032	0.026218604
ENSMUSG00000050821	Fam131a	-0.748955548	2.06846E-05	ENSMUSG00000004931	Apba3	0.598935317	2.17282E-22
ENSMUSG00000044350	Lacc1	-0.749676414	2.65204E-18	ENSMUSG00000027829	Ccnl1	0.598824156	1.88282E-53
ENSMUSG00000070031	Sp140	-0.749978129	8.69647E-06	ENSMUSG00000020544	Cox11	0.598625582	1.07526E-08
ENSMUSG00000031327	Chic1	-0.750573965	1.44918E-08	ENSMUSG00000007039	Ddah2	0.598322765	3.17812E-13
ENSMUSG00000039428	Tmem135	-0.750818783	3.64987E-28	ENSMUSG00000039463	Slc9a8	0.597743849	7.27404E-28
ENSMUSG00000051331	Cacna1c	-0.751263191	2.3863E-15	ENSMUSG00000030718	Ppme1	0.59673958	1.69653E-44
ENSMUSG00000023025	Larp4	-0.751306775	2.20682E-88	ENSMUSG00000092569	Gm20544	0.596105688	4.27549E-05
ENSMUSG00000074794	Arrdc3	-0.751846595	1.32913E-24	ENSMUSG00000020315	Sptbn1	0.596102677	4.69266E-96
ENSMUSG00000007989	Fzd3	-0.75194156	1.05921E-06	ENSMUSG00000006412	Pfdn2	0.595694942	9.28582E-25
ENSMUSG00000026278	Bok	-0.753102116	5.91175E-55	ENSMUSG00000015733	Capza2	0.595676691	1.61146E-74
ENSMUSG00000043398	Gpr135	-0.754214883	0.014029742	ENSMUSG00000003617	Cp	0.595607991	2.63094E-80
ENSMUSG00000045328	Cenpe	-0.754371026	7.27712E-43	ENSMUSG00000024912	Fosl1	0.595472577	7.88562E-14
ENSMUSG00000074994	Qser1	-0.75515791	2.22433E-43	ENSMUSG00000056749	Nfil3	0.594912164	1.69724E-19
ENSMUSG00000049303	Syt12	-0.755409729	5.50528E-13	ENSMUSG00000022540	Rogdi	0.594856769	0.000100493
ENSMUSG00000034974	Dapk3	-0.755982485	9.84807E-19	ENSMUSG00000056429	Tgoln1	0.594726339	5.932E-102
ENSMUSG00000031918	Mtmr2	-0.756000958	1.9487E-85	ENSMUSG00000031825	Crispld2	0.594533977	3.23331E-08
ENSMUSG00000019891	Dcbld1	-0.756966291	2.35233E-12	ENSMUSG00000022139	Mbnl2	0.594410213	9.92796E-86
ENSMUSG00000051517	Arhgef39	-0.757057451	5.10874E-05	ENSMUSG00000027099	Mtx2	0.593907672	1.09953E-27
ENSMUSG00000061603	Akap6	-0.757085218	2.41936E-09	ENSMUSG00000001036	Epn2	0.593856141	3.3281E-51
ENSMUSG00000021965	Ska3	-0.75709809	2.05648E-11	ENSMUSG00000048874	Phf3	0.593828721	3.87992E-52

ENSMUSG00000033209	Ttc28	-0.757114307	2.90494E-37	ENSMUSG00000038181	Chpf2	0.593811823	1.37087E-59
ENSMUSG00000002870	Mcm2	-0.757583491	6.13858E-45	ENSMUSG00000028164	Manba	0.59376109	6.12192E-26
ENSMUSG00000030093	Wnt7a	-0.757748642	2.99861E-11	ENSMUSG00000051344	Plekhm3	0.593327886	8.52927E-24
ENSMUSG00000044033	Ccdc141	-0.757844495	2.91724E-05	ENSMUSG00000027244	Atg13	0.593180941	6.41808E-37
ENSMUSG00000010609	Psen2	-0.758461219	7.52961E-51	ENSMUSG00000041426	Hibch	0.592355301	6.10388E-11
ENSMUSG00000037440	Vnn1	-0.759454118	7.85211E-06	ENSMUSG00000027222	Pex16	0.592296129	1.55928E-06
ENSMUSG00000038168	P3h2	-0.760279337	1.68167E-15	ENSMUSG00000024782	Ak3	0.592086028	1.98675E-35
ENSMUSG00000054404	Slfn5	-0.761552895	6.87863E-24	ENSMUSG00000025993	Slc40a1	0.591865982	1.52018E-12
ENSMUSG00000028438	Kif24	-0.762147657	5.09613E-05	ENSMUSG00000027649	Ctnnbl1	0.591654086	7.37055E-14
ENSMUSG00000021540	Smad5	-0.762714211	6.21773E-46	ENSMUSG00000005625	Psmd4	0.591281557	1.83942E-39
ENSMUSG00000028262	Clca3a2	-0.763326346	1.61517E-18	ENSMUSG00000010607	Pigyl	0.591163743	3.66215E-06
ENSMUSG00000030557	Mef2a	-0.763536616	2.8331E-142	ENSMUSG00000039745	Htatip2	0.590750876	4.16714E-20
ENSMUSG00000033610	Pank1	-0.763558246	7.82511E-08	ENSMUSG00000037415	Ranbp10	0.590624682	1.22935E-38
ENSMUSG00000037868	Egr2	-0.763623079	2.01895E-13	ENSMUSG00000030291	Med21	0.59061499	2.87075E-12
ENSMUSG00000023909	Paqr4	-0.764416632	6.05927E-14	ENSMUSG00000040139	9430038I01Rik	0.590567718	0.010874042
ENSMUSG00000036768	Kif15	-0.76571274	1.45318E-22	ENSMUSG00000027502	Rtf2	0.590261998	1.53592E-35
ENSMUSG00000027323	Rad51	-0.766194951	1.59454E-24	ENSMUSG00000026107	Nabp1	0.589597727	1.51133E-35
ENSMUSG00000031431	Tsc22d3	-0.766464941	1.02555E-07	ENSMUSG00000031731	Ap1g1	0.589193324	6.02748E-62
ENSMUSG00000045854	Lyrm2	-0.766642864	2.40686E-11	ENSMUSG00000049723	Mmp12	0.588642252	0.000766934
ENSMUSG00000024791	Cdca5	-0.767456722	1.55115E-12	ENSMUSG00000024773	Atg2a	0.588618109	1.48776E-17
ENSMUSG00000020674	Pxdn	-0.767704884	8.79E-145	ENSMUSG00000044308	Ubr3	0.588538147	1.63604E-56
ENSMUSG00000022092	Ppp3cc	-0.77013847	1.13124E-15	ENSMUSG00000056999	Ide	0.588285775	5.73498E-31
ENSMUSG00000049086	Bmyc	-0.77032238	0.000870702	ENSMUSG00000007987	Ift22	0.587934136	1.90592E-11
ENSMUSG00000009470	Tnpo1	-0.770505909	9.08977E-97	ENSMUSG00000018796	Acsl1	0.587327999	2.13964E-30
ENSMUSG00000030688	Stard10	-0.771013501	0.000235259	ENSMUSG00000053070	Cfap300	0.586609634	2.61131E-06
ENSMUSG00000052565	H1f3	-0.771508564	0.017677619	ENSMUSG00000010057	Nprl2	0.586320328	2.65269E-05
ENSMUSG00000096472	Cdkn2d	-0.772907018	2.29119E-13	ENSMUSG00000037519	Ppfia1	0.586142361	2.7636E-50
ENSMUSG00000020974	Pole2	-0.772959574	3.05964E-07	ENSMUSG00000020921	Tmem101	0.586049426	3.75843E-11
ENSMUSG00000038552	Fndc4	-0.773116847	0.001084131	ENSMUSG00000030839	Sergef	0.585831442	0.000263742
ENSMUSG00000005470	Asf1b	-0.773466237	2.15211E-24	ENSMUSG00000040584	Abcb1a	0.585794974	9.08985E-08
ENSMUSG00000024222	Fkbp5	-0.77346789	4.06167E-43	ENSMUSG00000047843	Bri3	0.585416335	6.70123E-13
ENSMUSG00000042251	Pm20d1	-0.774086727	0.003109583	ENSMUSG00000021759	Plpp1	0.585362366	8.50501E-43
ENSMUSG00000048960	Prex2	-0.774532509	2.4555E-62	ENSMUSG00000036002	Fam214b	0.585341629	4.02885E-38
ENSMUSG00000027639	Samhd1	-0.774754928	8.66881E-32	ENSMUSG00000047227	Gm527	0.585244378	0.001311052
ENSMUSG00000002257	Def6	-0.775045214	1.06628E-13	ENSMUSG00000024853	Sf3b2	0.585203778	2.51014E-90
ENSMUSG00000030956	Fam53b	-0.777301001	2.04809E-26	ENSMUSG00000001105	Ift20	0.585052978	6.45815E-24
ENSMUSG00000026860	Sh3glb2	-0.777901783	1.42063E-34	ENSMUSG00000006731	B4galnt1	0.585050652	1.35598E-05
ENSMUSG00000046179	E2f8	-0.777970971	3.93118E-13	ENSMUSG00000002688	Prkd1	0.585049651	7.38627E-11
ENSMUSG00000024921	Smarca2	-0.779018242	2.37174E-39	ENSMUSG00000079197	Psme2	-0.585228979	1.04535E-20
ENSMUSG00000026605	Cenpf	-0.779121666	4.96599E-53	ENSMUSG00000027692	Tnik	-0.585913715	7.57976E-06
ENSMUSG00000031790	Mmp15	-0.779464735	0.009410646	ENSMUSG00000031433	Rbm41	-0.585959621	2.42607E-10
ENSMUSG00000079020	Slc45a4	-0.780206608	3.98394E-37	ENSMUSG00000031431	Tsc22d3	-0.585968314	5.18841E-05
ENSMUSG00000019920	Lims1	-0.780310516	8.8049E-155	ENSMUSG00000021277	Traf3	-0.586080903	1.03768E-34
ENSMUSG00000060487	Samd5	-0.780952667	5.51906E-21	ENSMUSG00000024974	Smc3	-0.586196822	1.03996E-40
ENSMUSG00000026546	Cfap45	-0.781373402	1.11883E-07	ENSMUSG00000005566	Trim28	-0.586458175	6.39381E-70
ENSMUSG00000025026	Add3	-0.7823665	2.22026E-46	ENSMUSG00000082809	Gm14150	-0.587024284	8.4582E-18
ENSMUSG00000038122	Tbc1d32	-0.782391201	1.58332E-08	ENSMUSG00000032363	Adamts7	-0.587282662	2.84602E-19
ENSMUSG00000046668	Cxxc5	-0.782813425	1.42222E-18	ENSMUSG00000045636	Mtus1	-0.587461643	0.000213871
ENSMUSG00000040084	Bub1b	-0.783009554	7.0645E-51	ENSMUSG00000037286	Stag1	-0.587530565	1.24831E-26
ENSMUSG00000074277	Phldb3	-0.783126955	0.048770464	ENSMUSG00000038351	Sgsm2	-0.587586923	2.38487E-08
ENSMUSG00000019961	Tmpo	-0.783835377	3.26612E-86	ENSMUSG00000024319	Vps52	-0.587980829	2.01833E-19
ENSMUSG00000039206	Daglb	-0.784454217	1.12579E-15	ENSMUSG00000041354	Rgl2	-0.588001178	2.08235E-16
ENSMUSG00000050370	Ch25h	-0.78445804	7.2484E-46	ENSMUSG00000066952	Myo1h	-0.588379331	0.027127688
ENSMUSG00000031803	B3gnt3	-0.784807267	2.0705E-13	ENSMUSG00000036622	Atp13a2	-0.588538977	1.36677E-10
ENSMUSG00000001249	Hpn	-0.786157409	0.043714304	ENSMUSG00000028073	Pear1	-0.588611802	9.35814E-31
ENSMUSG00000035697	Arhgap45	-0.78735227	0.027058486	ENSMUSG00000082585	Gm15387	-0.589029266	0.048990117
ENSMUSG00000051378	Kif18b	-0.787908066	8.46282E-30	ENSMUSG00000022574	Naprt	-0.589179268	0.000228817
ENSMUSG00000024727	Trpm6	-0.788356335	0.000186554	ENSMUSG00000033166	Dis3	-0.589366736	2.41914E-25
ENSMUSG00000035151	Elmod2	-0.788539278	1.6516E-21	ENSMUSG00000002416	Ndufb2	-0.589443765	1.00733E-09
ENSMUSG00000041828	Abca8a	-0.788569204	2.95693E-45	ENSMUSG00000022906	Parp9	-0.589889851	0.01324029
ENSMUSG00000047867	Gimap6	-0.788702895	0.000235421	ENSMUSG00000020536	Llgl1	-0.590156524	1.63332E-18
ENSMUSG00000040811	Eml2	-0.78876002	1.50409E-16	ENSMUSG00000046311	Zfp62	-0.590169566	1.3093E-13
ENSMUSG00000021892	Sh3bp5	-0.789124686	1.6349E-110	ENSMUSG00000038241	Cep250	-0.590268875	8.90055E-47
ENSMUSG00000029373	Pf4	-0.789914984	4.6545E-11	ENSMUSG00000113328	Gm47260	-0.590351705	0.001348863
ENSMUSG00000055761	Nkain3	-0.790432207	0.000713801	ENSMUSG00000035310	Lin54	-0.590595708	1.87686E-16
ENSMUSG00000019718	L3hypdh	-0.79045408	2.31795E-09	ENSMUSG00000087502	Gm16091	-0.590862482	0.001221507
ENSMUSG00000001663	Gstt1	-0.790750886	2.05112E-07	ENSMUSG00000097750	Gm4673	-0.591796274	0.038294173
ENSMUSG00000023072	Cep89	-0.791889558	3.97428E-13	ENSMUSG00000036905	C1qb	-0.591874471	0.030850739
ENSMUSG00000052632	Asap2	-0.792086452	1.13882E-43	ENSMUSG00000025049	Taf5	-0.592119908	0.000568077
ENSMUSG00000057388	Mrpl18	-0.792541322	5.98869E-30	ENSMUSG00000032643	Fhl3	-0.592355922	3.9505E-12
ENSMUSG00000047557	Lxn	-0.79318539	7.7261E-28	ENSMUSG00000027342	Pcna	-0.592359838	1.18832E-57
ENSMUSG00000025915	Sgk3	-0.793824019	5.69058E-24	ENSMUSG00000074358	Ccdc61	-0.59250283	1.46248E-06
ENSMUSG00000055805	Fmnl1	-0.794006927	0.001944987	ENSMUSG00000002109	Ddb2	-0.593061227	0.036002215
ENSMUSG00000055538	Zcchc24	-0.794331386	1.06109E-37	ENSMUSG00000056758	Hmga2	-0.593243932	5.63708E-55
ENSMUSG00000020709	Adap2	-0.794723239	0.003630089	ENSMUSG00000067629	Syngap1	-0.593315197	0.000213755
ENSMUSG00000093726	Gm20667	-0.794832624	0.009457269	ENSMUSG00000054871	Tmem158	-0.593811466	0.024807535
ENSMUSG00000032839	Trpc1	-0.795275188	6.7777E-14	ENSMUSG00000032038	St3gal4	-0.594572802	1.11314E-28

ENSMUSG00000022033	Pbk	-0.796357273	2.51715E-31	ENSMUSG00000039801	Cplane1	-0.594742194	2.25955E-16
ENSMUSG00000031965	Tbx20	-0.797243425	6.5021E-222	ENSMUSG00000027996	Sfrp2	-0.595023256	1.0169E-08
ENSMUSG00000037649	H2-DMa	-0.797378685	0.000379296	ENSMUSG00000043556	Fbxl7	-0.595230848	0.000123834
ENSMUSG00000085399	Foxd2os	-0.798113156	4.4367E-05	ENSMUSG00000040034	Nup43	-0.595489311	8.38995E-08
ENSMUSG00000027327	1700037H04Rik	-0.798998422	1.83721E-16	ENSMUSG00000031730	Dhodh	-0.595500149	3.53711E-05
ENSMUSG00000002731	Prkra	-0.799884384	3.16775E-16	ENSMUSG00000028080	Lrba	-0.595509333	1.05354E-10
ENSMUSG00000118559	A930007A09Rik	-0.80041067	2.33111E-08	ENSMUSG00000043346	Gm6741	-0.595576236	0.004444903
ENSMUSG00000030671	Pde3b	-0.800542038	3.76451E-17	ENSMUSG00000003308	Keap1	-0.595943818	3.11826E-31
ENSMUSG00000022322	Shcbp1	-0.801091282	6.55823E-26	ENSMUSG00000022231	Sema5a	-0.596574539	1.79259E-29
ENSMUSG00000040296	Ddx58	-0.801647811	1.11473E-06	ENSMUSG00000028556	Dock7	-0.596992887	9.66627E-47
ENSMUSG00000036834	Plch1	-0.804993902	0.016615376	ENSMUSG00000090112	Shprh	-0.597281173	3.25017E-16
ENSMUSG00000038793	Lefty1	-0.80589623	2.18985E-05	ENSMUSG00000027800	Tm4sf1	-0.597574968	1.0762E-162
ENSMUSG00000063714	Sp3os	-0.805961424	0.040076471	ENSMUSG00000028059	Arhgef2	-0.597661624	5.40471E-41
ENSMUSG00000021411	Pxdc1	-0.806612662	2.74983E-44	ENSMUSG00000024985	Tcf7l2	-0.597666835	9.15744E-10
ENSMUSG00000073489	Ifi204	-0.807118017	0.002432143	ENSMUSG00000054517	Trim65	-0.59817293	0.000136452
ENSMUSG00000050192	Eif5a2	-0.807142366	7.54855E-25	ENSMUSG00000005687	Bcas2	-0.598204795	4.63182E-20
ENSMUSG00000006398	Cdc20	-0.807322427	4.26635E-39	ENSMUSG00000033147	Slc22a15	-0.598309181	7.20411E-11
ENSMUSG00000022177	Haus4	-0.807352744	3.35241E-12	ENSMUSG00000028607	Cpt2	-0.598311767	6.26781E-14
ENSMUSG00000078776	9530053A07Rik	-0.807868525	0.012821191	ENSMUSG00000021947	Cryl1	-0.598398969	0.000101652
ENSMUSG00000043614	Vps37d	-0.808357521	0.001339955	ENSMUSG00000021809	Nudt13	-0.598677065	0.00013423
ENSMUSG00000063568	Jazf1	-0.808823478	1.71776E-08	ENSMUSG00000005802	Slc30a4	-0.598728816	6.76968E-34
ENSMUSG00000027353	Mcm8	-0.809259009	3.0307E-07	ENSMUSG00000032855	Pkd1	-0.598790739	1.68301E-44
ENSMUSG00000027304	Rtf1	-0.809579402	5.44746E-44	ENSMUSG00000020646	Mboat2	-0.598946768	3.83647E-06
ENSMUSG00000020646	Mboat2	-0.810150121	2.96263E-09	ENSMUSG00000040957	Cables1	-0.599632157	0.01458271
ENSMUSG00000074480	Mex3a	-0.810515554	4.03441E-10	ENSMUSG00000040282	BC052040	-0.599755791	0.000635855
ENSMUSG00000114019	Gm47155	-0.810829754	0.036165736	ENSMUSG00000032860	P2ry2	-0.599933846	8.00686E-24
ENSMUSG00000039770	Ypel5	-0.811476209	4.90924E-34	ENSMUSG00000025213	Kazald1	-0.600084482	0.032088693
ENSMUSG00000041147	Brca2	-0.811890541	9.90648E-12	ENSMUSG00000004562	Arhgef40	-0.600209552	1.76833E-49
ENSMUSG00000020661	Dnmt3a	-0.812955833	1.02166E-66	ENSMUSG00000022814	Umps	-0.600647614	4.42305E-25
ENSMUSG00000063445	Nmral1	-0.813912199	2.02821E-06	ENSMUSG00000025218	Poll	-0.600660216	0.001745526
ENSMUSG00000020003	Pex7	-0.815581473	4.87749E-22	ENSMUSG00000047281	Sfn	-0.601285745	0.00127794
ENSMUSG00000028795	Ccdc28b	-0.816106872	4.3816E-05	ENSMUSG00000006445	Epha2	-0.601612932	6.89322E-59
ENSMUSG00000023089	Ndufa5	-0.816848242	1.62527E-19	ENSMUSG00000051890	Klhdc1	-0.601708435	0.049660166
ENSMUSG00000040350	Trim7	-0.816873929	6.5271E-07	ENSMUSG00000032243	Itga11	-0.601876003	1.66644E-10
ENSMUSG00000026955	Sapcd2	-0.817470409	1.6998E-08	ENSMUSG00000097828	6430562O15Rik	-0.602219325	0.023406951
ENSMUSG00000037126	Psd	-0.817534485	3.84084E-08	ENSMUSG00000003929	Zfp81	-0.602472438	2.39556E-06
ENSMUSG00000020525	Ppm1d	-0.817608729	4.63664E-23	ENSMUSG00000028567	Txndc12	-0.603350384	5.75337E-35
ENSMUSG00000040723	Rcsd1	-0.817907462	7.64986E-49	ENSMUSG00000058756	Thra	-0.603389204	6.94806E-14
ENSMUSG00000026039	Sgo2a	-0.818807877	2.00948E-36	ENSMUSG00000039328	Rnf122	-0.603629801	7.42018E-06
ENSMUSG00000034656	Cacna1a	-0.819460619	3.9267E-10	ENSMUSG00000007655	Cav1	-0.604217488	4.72884E-71
ENSMUSG00000034275	Igsf9b	-0.820148603	5.03256E-08	ENSMUSG00000051910	Sox6	-0.604740437	8.24503E-06
ENSMUSG00000030074	Gxylt2	-0.820650189	9.3635E-106	ENSMUSG00000067336	Bmpr2	-0.604921216	4.18511E-62
ENSMUSG00000042842	Serpinb6b	-0.821221161	7.75212E-50	ENSMUSG00000024736	Tmem132a	-0.604974019	2.60206E-28
ENSMUSG00000030031	Kbtbd8	-0.821623897	0.000627099	ENSMUSG00000046191	Pcdhb20	-0.605405571	0.040166865
ENSMUSG00000059013	Sh2d3c	-0.821735717	0.001724464	ENSMUSG00000043336	Filip1l	-0.605521198	5.93279E-32
ENSMUSG00000034906	Ncaph	-0.822444341	2.2362E-31	ENSMUSG00000026047	Poglut2	-0.605618347	1.94194E-22
ENSMUSG00000032667	Pon2	-0.823059903	6.87267E-64	ENSMUSG00000035704	Alg8	-0.605944157	1.83499E-12
ENSMUSG00000084883	Ccdc85c	-0.823350064	3.33902E-05	ENSMUSG00000024790	Sac3d1	-0.606053105	1.01925E-08
ENSMUSG00000060572	Mfap2	-0.825086827	0.042082073	ENSMUSG00000070604	Vsig10l	-0.606084448	0.018335982
ENSMUSG00000005233	Spc25	-0.825956303	6.60837E-31	ENSMUSG00000044528	Tram1l1	-0.606152138	3.21574E-05
ENSMUSG00000027346	Gpcpd1	-0.826701939	9.97978E-51	ENSMUSG00000116656	Gm49708	-0.606537595	0.017342081
ENSMUSG00000017861	Mybl2	-0.826717741	3.94633E-15	ENSMUSG00000057234	Mettl15	-0.606722549	0.005374833
ENSMUSG00000051832	E230016K23Rik	-0.827045558	0.029179967	ENSMUSG00000068130	Zfp442	-0.606771561	0.044988735
ENSMUSG00000024134	Six2	-0.827090077	1.79364E-06	ENSMUSG00000057886	Cbx3-ps6	-0.606778663	0.005672773
ENSMUSG00000047246	H2bc6	-0.828256047	0.004080018	ENSMUSG00000075327	Zbtb2	-0.60688055	9.88788E-15
ENSMUSG00000099241	Gm18852	-0.830136562	0.00012505	ENSMUSG00000057530	Ece1	-0.60699642	1.04375E-97
ENSMUSG00000029787	Avl9	-0.831654004	1.04047E-49	ENSMUSG00000035268	Pkig	-0.607542773	1.94555E-14
ENSMUSG00000041642	Kif21b	-0.831824119	4.6376E-10	ENSMUSG00000052906	Ubxn8	-0.607799414	2.66984E-16
ENSMUSG00000021057	Akap5	-0.833039089	1.06565E-09	ENSMUSG00000061273	Mmgt1	-0.60811733	7.49116E-36
ENSMUSG00000020623	Map2k6	-0.833051628	8.43744E-06	ENSMUSG00000059851	Kmt5c	-0.608415768	2.79738E-06
ENSMUSG00000031004	Mki67	-0.833209494	1.9189E-79	ENSMUSG00000027508	Pag1	-0.608615588	7.56954E-08
ENSMUSG00000049036	Tmem121	-0.834201136	1.73237E-05	ENSMUSG00000035401	Emsy	-0.60863701	8.221E-22
ENSMUSG00000073434	Wdr90	-0.834283491	1.9933E-14	ENSMUSG00000028957	Per3	-0.608666835	1.53482E-05
ENSMUSG00000038070	Cntln	-0.834318744	1.46392E-20	ENSMUSG00000075289	Carns1	-0.608845977	0.015321664
ENSMUSG00000024339	Tap2	-0.834529038	1.92758E-22	ENSMUSG00000110631	Gm42047	-0.609382108	2.71334E-48
ENSMUSG00000070802	Pnmal2	-0.834542967	0.011429633	ENSMUSG00000025092	Hspa12a	-0.609469456	7.64307E-11
ENSMUSG00000048327	Ckap2l	-0.834758059	7.64685E-52	ENSMUSG00000009681	Bcr	-0.609487108	3.91251E-25
ENSMUSG00000028737	Aldh4a1	-0.835458703	1.99741E-26	ENSMUSG00000044250	Pced1b	-0.609663876	0.032955196
ENSMUSG00000022419	Deptor	-0.835885553	5.31265E-08	ENSMUSG00000026663	Atf6	-0.609704177	1.54627E-53
ENSMUSG00000046807	Lrrc75b	-0.835970572	8.10449E-05	ENSMUSG00000053137	Mapk11	-0.60976533	2.88884E-08
ENSMUSG00000033060	Lmo7	-0.83701093	2.61604E-07	ENSMUSG00000054387	Mdm4	-0.609796065	1.50641E-36
ENSMUSG00000016494	Cd34	-0.837508298	1.6233E-250	ENSMUSG00000037447	Arid5a	-0.610158802	5.98935E-13
ENSMUSG00000020326	Ccng1	-0.837645919	1.3386E-237	ENSMUSG00000020647	Ncoa1	-0.610251626	2.36198E-15
ENSMUSG00000029442	Wdr66	-0.837881997	3.04027E-06	ENSMUSG00000023089	Ndufa5	-0.610494924	1.15589E-13
ENSMUSG00000029238	Clock	-0.83800895	7.46493E-69	ENSMUSG00000018427	Ypel2	-0.61050619	3.39472E-11
ENSMUSG00000025104	Hdgfl3	-0.838435922	1.12586E-24	ENSMUSG00000059049	Frem1	-0.610839335	9.53995E-07
ENSMUSG00000037313	Tacc3	-0.839236563	3.49755E-68	ENSMUSG00000034636	Zyg11b	-0.611073426	1.01965E-28

ENSMUSG00000023940	Sgo1	-0.839490903	3.36173E-21
ENSMUSG00000049281	Scn3b	-0.839632693	0.028819979
ENSMUSG00000049791	Fzd4	-0.839997367	5.19063E-36
ENSMUSG00000037110	Ralgapa2	-0.84026317	8.40037E-34
ENSMUSG00000074785	Plxnc1	-0.841766144	1.49762E-05
ENSMUSG00000075284	Wipf1	-0.842964707	1.5985E-64
ENSMUSG00000021697	Depdc1b	-0.843357114	0.000160696
ENSMUSG00000072964	Bhlhb9	-0.843681913	1.03903E-45
ENSMUSG00000026102	Inpp1	-0.843723789	4.20373E-43
ENSMUSG00000095457	Gm8989	-0.844794193	0.00915022
ENSMUSG00000024679	Ms4a6d	-0.844802528	0.003485651
ENSMUSG00000043953	Ccrl2	-0.845060632	7.10993E-05
ENSMUSG00000021457	Syk	-0.846370657	3.01662E-53
ENSMUSG00000048200	Cracr2b	-0.846427956	1.05774E-06
ENSMUSG00000062421	Arf2	-0.846838019	1.05796E-76
ENSMUSG00000025650	Col7a1	-0.847610799	6.65521E-15
ENSMUSG00000072244	Trim6	-0.847744233	1.97665E-05
ENSMUSG00000060675	Plaat3	-0.848101676	6.55823E-26
ENSMUSG00000029869	Ephb6	-0.848239187	7.83949E-05
ENSMUSG00000072620	Slfn2	-0.848584138	5.80561E-08
ENSMUSG00000019845	Tube1	-0.849266409	2.02424E-05
ENSMUSG00000029765	Plxna4	-0.849366166	2.06868E-14
ENSMUSG00000036672	Cenpt	-0.850511721	3.14395E-32
ENSMUSG00000068882	Ssb	-0.850756257	7.22519E-95
ENSMUSG00000112941	Gm48623	-0.8511042	0.009506984
ENSMUSG00000048368	Omd	-0.851120667	0.01579655
ENSMUSG00000031937	Vstm5	-0.851433226	5.19955E-10
ENSMUSG00000022906	Parp9	-0.851634833	0.000762162
ENSMUSG00000030677	Kif22	-0.852123754	7.59905E-51
ENSMUSG00000021087	Rtn1	-0.852920916	2.67081E-07
ENSMUSG00000037997	Parp11	-0.853335509	1.61924E-11
ENSMUSG00000020439	Smtn	-0.854923968	4.7912E-105
ENSMUSG00000039629	Strip2	-0.856901583	0.00542328
ENSMUSG00000029730	Mcm7	-0.857085003	1.00858E-52
ENSMUSG00000005950	P2rx5	-0.857132998	4.30682E-13
ENSMUSG00000108358	Gm44509	-0.860107005	0.005667095
ENSMUSG00000115718	Gm49179	-0.860297951	0.042731667
ENSMUSG00000032011	Thy1	-0.860341676	2.9567E-163
ENSMUSG00000033022	Cdo1	-0.860854706	3.83017E-09
ENSMUSG00000066842	Hmcn1	-0.861905157	0.003830462
ENSMUSG00000038037	Socs1	-0.861963548	0.000170773
ENSMUSG00000026622	Nek2	-0.862167628	1.6472E-51
ENSMUSG00000039704	Lmbrd2	-0.864051102	8.94873E-55
ENSMUSG00000028789	Azin2	-0.866327406	6.97022E-25
ENSMUSG00000027379	Bub1	-0.867809366	2.9515E-42
ENSMUSG00000013973	Dedd	-0.86814517	1.74937E-23
ENSMUSG00000042978	Sbk1	-0.868189441	5.00557E-07
ENSMUSG00000044678	Ly6k	-0.869006297	0.000549285
ENSMUSG00000023010	Tmbim6	-0.870256873	5.218E-221
ENSMUSG00000027022	Xirp2	-0.870530541	0.002909874
ENSMUSG00000006575	Rundc3a	-0.871341996	0.012807075
ENSMUSG00000044345	Marveld1	-0.872142543	1.2636E-112
ENSMUSG00000021253	Tgfb3	-0.872659203	1.19159E-98
ENSMUSG00000015745	Plekho1	-0.874697649	1.29486E-18
ENSMUSG00000021624	Cd180	-0.875900281	6.1909E-12
ENSMUSG00000027253	Lrp4	-0.876272172	8.09872E-09
ENSMUSG00000046879	Irgm1	-0.877590059	2.269E-10
ENSMUSG00000020303	Stc2	-0.879062219	7.94918E-19
ENSMUSG00000042726	Trafd1	-0.879223466	4.97572E-29
ENSMUSG00000028931	Kcnab2	-0.88005046	0.0016422
ENSMUSG00000051177	Plcb1	-0.881079994	4.24697E-11
ENSMUSG00000016262	Sertad4	-0.881091695	9.35657E-05
ENSMUSG00000022236	Ropn1l	-0.881188926	0.000909879
ENSMUSG00000105509	C130013H08Rik	-0.881814421	3.9681E-05
ENSMUSG00000026196	Bard1	-0.881892768	7.7867E-13
ENSMUSG00000025574	Tk1	-0.883289096	1.70392E-39
ENSMUSG00000038351	Sgsm2	-0.883506501	8.77475E-12
ENSMUSG00000030217	Art4	-0.883791204	1.98979E-07
ENSMUSG00000072647	Adam1a	-0.884107696	0.008251879
ENSMUSG00000091549	Gm6548	-0.885339021	3.48463E-18
ENSMUSG00000028035	Dnajb4	-0.886660175	6.65504E-56
ENSMUSG00000042460	C1galt1	-0.88802897	2.41068E-63
ENSMUSG00000022235	Cmbl	-0.888894165	6.40016E-08
ENSMUSG00000026737	Pip4k2a	-0.889034519	2.25854E-57
ENSMUSG00000070287	Slc35g2	-0.891807316	0.001165009
ENSMUSG00000024269	Tpgs2	-0.891875799	2.2225E-23
ENSMUSG00000031543	Ank1	-0.892726544	1.58767E-15
ENSMUSG00000029122	Evc	-0.611173185	9.0631E-20
ENSMUSG00000038872	Zfhx3	-0.611540305	4.78885E-11
ENSMUSG00000028894	Inpp5b	-0.611689136	3.57905E-19
ENSMUSG00000108381	Gm9299	-0.611694059	2.60859E-11
ENSMUSG00000042404	Dennd4b	-0.611958278	8.23588E-06
ENSMUSG00000029924	Slc37a3	-0.61238295	5.86457E-44
ENSMUSG00000032253	Phip	-0.613443497	5.26071E-28
ENSMUSG00000044982	Sft2d3	-0.613595285	9.9526E-06
ENSMUSG00000053436	Mapk14	-0.613639622	9.0054E-61
ENSMUSG00000020889	Nr1d1	-0.614065885	1.2854E-16
ENSMUSG00000022636	Alcam	-0.614571826	1.32121E-24
ENSMUSG00000027550	Lrrcc1	-0.614653544	1.30403E-13
ENSMUSG00000034731	Dgkh	-0.614678093	1.40152E-20
ENSMUSG00000049307	Fut4	-0.614678617	2.87277E-05
ENSMUSG00000044934	Zfp367	-0.614755737	1.49476E-22
ENSMUSG00000024769	Cdc42bpg	-0.615087027	1.92914E-16
ENSMUSG00000066800	Rnasel	-0.615230695	2.03592E-09
ENSMUSG00000078486	Perm1	-0.615312551	0.002274927
ENSMUSG00000015053	Gata2	-0.615465709	0.000668463
ENSMUSG00000037295	Ldlrap1	-0.615562632	6.2135E-35
ENSMUSG00000035521	Gnptg	-0.615631944	8.25999E-12
ENSMUSG00000041313	Slc7a1	-0.615770382	1.44756E-75
ENSMUSG00000055435	Maf	-0.616187927	0.010707035
ENSMUSG00000031823	Zdhhc7	-0.616647152	8.65536E-23
ENSMUSG00000041718	Alg13	-0.616790289	5.00404E-11
ENSMUSG00000021959	Lats2	-0.617039744	2.46049E-85
ENSMUSG00000004500	Zfp324	-0.617235701	1.88339E-08
ENSMUSG00000040699	Limd2	-0.617692858	1.0245E-20
ENSMUSG00000037621	Atoh8	-0.618321019	7.14136E-22
ENSMUSG00000117113	Gm49883	-0.618399722	0.024427796
ENSMUSG00000037816	Fbxw17	-0.618518577	5.03896E-10
ENSMUSG00000049516	Spty2d1	-0.61878619	1.42235E-34
ENSMUSG00000028467	Gba2	-0.619401271	1.11833E-10
ENSMUSG00000040681	Hmgn1	-0.619976397	3.84015E-70
ENSMUSG00000003549	Ercc1	-0.620352831	2.17041E-19
ENSMUSG00000026121	Sema4c	-0.620725779	2.66612E-19
ENSMUSG00000036097	Slf2	-0.620844928	6.85945E-41
ENSMUSG00000020189	Osbpl8	-0.62129423	6.68481E-30
ENSMUSG00000022106	Rcbtb2	-0.621883435	5.20195E-15
ENSMUSG00000029836	Cbx3	-0.622016782	6.8668E-56
ENSMUSG00000022822	Abcc5	-0.622034011	2.63207E-42
ENSMUSG00000039715	Wdr34	-0.622056127	4.50551E-07
ENSMUSG00000038507	Parp12	-0.622453119	0.001031443
ENSMUSG00000038250	Usp38	-0.622622311	6.50545E-21
ENSMUSG00000037110	Ralgapa2	-0.622718485	2.2643E-19
ENSMUSG00000029228	Lnx1	-0.623461211	0.00547461
ENSMUSG00000074165	Zfp788	-0.623527664	0.000394288
ENSMUSG00000019761	Krt10	-0.623634408	0.045609626
ENSMUSG00000045680	Tcf21	-0.623954649	1.91938E-54
ENSMUSG00000051238	Swsap1	-0.624133486	8.0882E-05
ENSMUSG00000030279	C2cd5	-0.624265715	3.92746E-09
ENSMUSG00000038545	Cul7	-0.624350362	3.43866E-42
ENSMUSG00000096054	Syne1	-0.624514603	9.84846E-23
ENSMUSG00000073176	Zfp449	-0.62464744	1.99042E-08
ENSMUSG00000025474	Tubgcp2	-0.625060462	1.0345E-22
ENSMUSG00000111375	Btbd8	-0.625429471	8.12883E-06
ENSMUSG00000025856	Pdgfa	-0.625709316	2.49102E-20
ENSMUSG00000074886	Grk6	-0.625856552	9.89073E-50
ENSMUSG00000072620	Slfn2	-0.62675881	8.10913E-06
ENSMUSG00000038732	Mboat1	-0.627186876	1.82866E-05
ENSMUSG00000070462	Tlnrd1	-0.627604705	3.84885E-20
ENSMUSG00000066878	Gm10184	-0.628620167	0.000996122
ENSMUSG00000005672	Kit	-0.628795157	0.004228026
ENSMUSG00000031530	Dusp4	-0.628906088	3.11491E-85
ENSMUSG00000027739	Rab33b	-0.628924084	1.74365E-25
ENSMUSG00000030688	Stard10	-0.629016557	0.001578456
ENSMUSG00000058325	Dock1	-0.629351048	1.7691E-37
ENSMUSG00000041992	Rapgef5	-0.629726147	7.43259E-22
ENSMUSG00000095432	Zfp748	-0.629975818	1.25464E-07
ENSMUSG00000030612	Mrpl46	-0.630625973	4.61941E-09
ENSMUSG00000052539	Magi3	-0.630773507	1.18803E-19
ENSMUSG00000026102	Inpp1	-0.630902325	2.24242E-31
ENSMUSG00000111080	Gm20300	-0.631059605	0.001150924
ENSMUSG00000031253	Srpx2	-0.631111048	6.98354E-46
ENSMUSG00000029826	Zc3hav1	-0.63121684	6.13062E-38
ENSMUSG00000031295	Phka2	-0.631306765	6.12736E-14
ENSMUSG00000040711	Sh3pxd2b	-0.631609234	1.0136E-117

ENSMUSG00000032492	Pth1r	-0.893568775	0.003081523
ENSMUSG00000027459	Fam110a	-0.893726437	2.68012E-16
ENSMUSG00000022856	Tmem41a	-0.894175053	3.40295E-10
ENSMUSG00000005951	Shpk	-0.894902667	0.009793069
ENSMUSG00000037010	Apln	-0.895303268	4.23394E-20
ENSMUSG00000025789	St8sia2	-0.895663594	0.005100383
ENSMUSG00000105176	Gm43668	-0.896140632	0.034250756
ENSMUSG00000052783	Grk4	-0.896398601	0.014160849
ENSMUSG00000032500	Dclk3	-0.896986742	0.006280938
ENSMUSG00000035403	Crb2	-0.897716564	0.034834244
ENSMUSG00000033952	Aspm	-0.897927753	5.70827E-46
ENSMUSG00000051041	Olfml1	-0.90127637	1.07236E-10
ENSMUSG00000056486	Chn1	-0.901661597	3.66873E-45
ENSMUSG00000023919	Cenpq	-0.902172591	1.44829E-21
ENSMUSG00000108350	Gm44950	-0.902487537	1.37786E-05
ENSMUSG00000059824	Dbp	-0.904561247	0.000661726
ENSMUSG00000002055	Spag5	-0.905731393	6.74293E-30
ENSMUSG00000023505	Cdca3	-0.905742964	2.79104E-48
ENSMUSG00000006715	Gmnn	-0.906624731	2.35178E-15
ENSMUSG00000005774	Rfx5	-0.907514432	1.79539E-16
ENSMUSG00000020182	Ddc	-0.907544973	0.015037284
ENSMUSG00000020150	Gamt	-0.90779782	2.26597E-11
ENSMUSG00000028328	Tmod1	-0.909634816	0.00527069
ENSMUSG00000097440	Gm6277	-0.90996605	0.022676141
ENSMUSG00000043252	Tmem64	-0.910037068	2.20836E-90
ENSMUSG00000020707	Rnf135	-0.910452965	2.3241E-07
ENSMUSG00000109157	Gm44829	-0.912922457	0.04585849
ENSMUSG00000001270	Ckb	-0.913306954	1.4082E-122
ENSMUSG00000079499	6530402F18Rik	-0.914167267	8.49676E-13
ENSMUSG00000003779	Kif20a	-0.914522074	2.5651E-117
ENSMUSG00000040167	Ikzf5	-0.915091114	0.000282187
ENSMUSG00000024524	Gnal	-0.915137128	0.002582434
ENSMUSG00000085030	2810455O05Rik	-0.915708117	0.026274621
ENSMUSG00000021908	Ncoa4-ps	-0.916739549	2.22065E-11
ENSMUSG00000062075	Lmnb2	-0.918257844	1.81383E-25
ENSMUSG00000072844	G530011O06Rik	-0.918592035	0.006968867
ENSMUSG00000026222	Sp100	-0.918775294	2.46044E-06
ENSMUSG00000036023	Parp2	-0.919415274	1.28992E-19
ENSMUSG00000048965	Mrgpre	-0.919476268	1.36468E-05
ENSMUSG00000040424	Hipk4	-0.91951926	0.042138278
ENSMUSG00000054717	Hmgb2	-0.920068329	1.83658E-72
ENSMUSG00000025265	Fgd1	-0.921619719	1.23753E-30
ENSMUSG00000019866	Crybg1	-0.923190346	0.006932953
ENSMUSG00000053113	Socs3	-0.923578938	3.30572E-33
ENSMUSG00000041540	Sox5	-0.923662871	4.46606E-20
ENSMUSG00000027349	Fam98b	-0.924168734	2.88988E-40
ENSMUSG00000026781	Acbd5	-0.92450977	2.30736E-97
ENSMUSG00000111681	Gm47640	-0.924778863	0.000554606
ENSMUSG00000078954	Arhgap8	-0.925975253	0.040725634
ENSMUSG00000103649	Gm37768	-0.92693238	0.005151451
ENSMUSG00000047759	Hs3st3a1	-0.927250373	0.00031456
ENSMUSG00000029636	Wasf3	-0.927515732	3.14234E-05
ENSMUSG00000028525	Pde4b	-0.92815603	1.81584E-61
ENSMUSG00000056234	Ncoa4	-0.928266889	8.9522E-124
ENSMUSG00000037712	Fermt2	-0.928997983	3.739E-227
ENSMUSG00000048852	Gm12185	-0.929825461	0.003831017
ENSMUSG00000022546	Gpt	-0.929844015	0.0001558
ENSMUSG00000042182	Bend6	-0.931023797	7.45906E-06
ENSMUSG00000044770	Scml4	-0.932146861	8.32983E-12
ENSMUSG00000020544	Cox11	-0.932929794	2.07628E-12
ENSMUSG00000026946	Nmi	-0.933160393	1.26455E-15
ENSMUSG00000053646	Plxnb1	-0.933409509	0.000432971
ENSMUSG00000024590	Lmnb1	-0.934527646	1.8292E-108
ENSMUSG00000027331	Knstrn	-0.934921863	7.2744E-59
ENSMUSG00000031639	Tlr3	-0.934942908	4.53122E-05
ENSMUSG00000050345	4930486L24Rik	-0.935050266	0.001537361
ENSMUSG00000051339	2900026A02Rik	-0.936116913	2.44245E-69
ENSMUSG00000010358	Ifi35	-0.936518531	2.66975E-05
ENSMUSG00000111080	Gm20300	-0.936577269	1.75032E-05
ENSMUSG00000105940	Gm42635	-0.938220001	0.003387238
ENSMUSG00000039697	Ncoa7	-0.938595863	2.73102E-30
ENSMUSG00000032698	Lmo2	-0.942422563	9.2699E-25
ENSMUSG00000026779	Mastl	-0.942726538	2.22244E-33
ENSMUSG00000027199	Gatm	-0.942989878	0.000137638
ENSMUSG00000041633	Kctd12b	-0.943959113	1.13221E-18
ENSMUSG00000114419	5430414B19Rik	-0.945451309	0.048802801
ENSMUSG00000032254	Kif23	-0.945607492	3.61785E-85
ENSMUSG00000062694	Cav3	-0.631683249	0.000824255
ENSMUSG00000113047	Gm47469	-0.631862405	0.021764075
ENSMUSG00000031995	St14	-0.632271235	0.008951636
ENSMUSG00000079555	Haus3	-0.632339813	8.88021E-25
ENSMUSG00000066551	Hmgb1	-0.632883873	5.2218E-104
ENSMUSG00000034601	2700049A03Rik	-0.63299886	6.85286E-07
ENSMUSG00000074793	Hspa12b	-0.633024292	3.79181E-05
ENSMUSG00000096221	1500002C15Rik	-0.633864233	0.049398265
ENSMUSG00000020620	Abca8b	-0.634122625	2.31547E-15
ENSMUSG00000097910	5033428I22Rik	-0.634392884	3.77311E-20
ENSMUSG00000020973	Dnaaf2	-0.634503112	3.22664E-08
ENSMUSG00000030671	Pde3b	-0.634760375	1.6874E-11
ENSMUSG00000024030	Abcg1	-0.635055889	7.40803E-06
ENSMUSG00000042726	Trafd1	-0.635211284	1.90436E-15
ENSMUSG00000022843	Clcn2	-0.635365991	0.000672825
ENSMUSG00000001128	Cfp	-0.635408319	0.016051183
ENSMUSG00000015214	Mtmr1	-0.635846458	4.75564E-18
ENSMUSG00000075588	Hoxb2	-0.636117136	0.019230562
ENSMUSG00000060288	Ppih	-0.636442558	1.51448E-08
ENSMUSG00000062190	Lancl2	-0.63667538	2.67444E-14
ENSMUSG00000045827	Serpinb9	-0.637356312	1.17169E-58
ENSMUSG00000034021	Pds5b	-0.637450499	3.5577E-27
ENSMUSG00000028689	Ccdc163	-0.637636681	0.000392809
ENSMUSG00000032293	Ireb2	-0.637939351	3.8834E-69
ENSMUSG00000026946	Nmi	-0.638273954	2.57607E-09
ENSMUSG00000031024	Denn2b	-0.638905172	1.11098E-43
ENSMUSG00000061371	Zfp873	-0.639337227	0.01251011
ENSMUSG00000042812	Foxf1	-0.639379646	0.008581817
ENSMUSG00000031642	Sh3rf1	-0.639427364	1.61607E-24
ENSMUSG00000026082	Rev1	-0.639449105	1.02843E-17
ENSMUSG00000006360	Crip1	-0.640281777	4.66543E-15
ENSMUSG00000022048	Dpysl2	-0.641184813	2.00173E-28
ENSMUSG00000037692	Ahdc1	-0.641835505	6.96573E-16
ENSMUSG00000028480	Glipr2	-0.642896139	1.28662E-45
ENSMUSG00000030498	Gas2	-0.643204924	5.35907E-05
ENSMUSG00000038349	Plcl1	-0.643369372	5.74196E-05
ENSMUSG00000028799	Zfp362	-0.643497922	1.60921E-13
ENSMUSG00000027201	Myef2	-0.645032007	4.62658E-64
ENSMUSG00000018387	Shroom1	-0.64506338	0.002439341
ENSMUSG00000002718	Cse1l	-0.645100221	3.08312E-51
ENSMUSG00000056313	Tcim	-0.645719886	2.85465E-06
ENSMUSG00000022075	Rhobtb2	-0.645925398	1.55747E-07
ENSMUSG00000118012	Gm46620	-0.646097473	3.44773E-07
ENSMUSG00000043439	Epop	-0.646833274	0.004199723
ENSMUSG00000001333	Sync	-0.646873318	7.82471E-09
ENSMUSG00000040998	Npnt	-0.647261363	3.74212E-33
ENSMUSG00000042502	Cd2bp2	-0.647309265	3.85133E-53
ENSMUSG00000027324	Rpusd2	-0.647485602	0.001084549
ENSMUSG00000033436	Armcx2	-0.64759848	4.77135E-63
ENSMUSG00000003283	Hck	-0.648183081	0.002925583
ENSMUSG00000018362	Kpna2	-0.64826889	2.2681E-105
ENSMUSG00000010154	Spire2	-0.648456933	0.008147483
ENSMUSG00000047656	Trpt1	-0.648501431	0.016663805
ENSMUSG00000027959	Sass6	-0.649093274	1.92774E-09
ENSMUSG00000103009	Gm4430	-0.649117395	0.000354566
ENSMUSG00000026726	Cubn	-0.649359535	1.11606E-05
ENSMUSG00000003423	Pih1d1	-0.650103756	5.65018E-20
ENSMUSG00000036504	Phpt1	-0.650132871	1.33814E-06
ENSMUSG00000047617	Paxx	-0.650674365	0.000242787
ENSMUSG00000009614	Sardh	-0.650692549	4.38617E-17
ENSMUSG00000059555	Tor4a	-0.651142583	1.0264E-30
ENSMUSG00000013584	Aldh1a2	-0.651629467	1.11434E-51
ENSMUSG00000087132	A930001C03Rik	-0.651654343	0.03979481
ENSMUSG00000040913	Fbxw4	-0.651828861	5.62987E-10
ENSMUSG00000038843	Gcnt1	-0.652389583	0.000960183
ENSMUSG00000043154	Ppp2r3a	-0.653817233	4.51402E-18
ENSMUSG00000027281	Slx4ip	-0.654113304	4.70257E-08
ENSMUSG00000066357	Wdr6	-0.65454263	2.028E-59
ENSMUSG00000079038	D130040H23Rik	-0.654606178	0.038113527
ENSMUSG00000026743	Mllt10	-0.65518119	4.79889E-25
ENSMUSG00000033885	Pxk	-0.655321038	3.78733E-32
ENSMUSG00000032932	Hspa13	-0.655993981	2.64337E-42
ENSMUSG00000021629	Slc30a5	-0.656012806	4.14309E-28
ENSMUSG00000035246	Pcyt1b	-0.656254499	4.59272E-09
ENSMUSG00000029094	Afap1	-0.656601376	7.00978E-56
ENSMUSG00000022364	Tbc1d31	-0.657238687	2.41344E-09
ENSMUSG00000106734	Gm20559	-0.657506506	0.005503803

ENSMUSG00000066643	Wdr35	-0.945776441	4.1542E-46	ENSMUSG00000058761	Rnf169	-0.658066306	2.44731E-50
ENSMUSG00000037060	Cavin3	-0.947864359	7.38149E-37	ENSMUSG00000033209	Ttc28	-0.658490283	9.3739E-36
ENSMUSG00000024301	Kifc5b	-0.949572012	1.28355E-20	ENSMUSG00000018401	Mtmr4	-0.658607059	1.42638E-16
ENSMUSG00000030787	Lyve1	-0.949693349	7.11649E-06	ENSMUSG00000033880	Lgals3bp	-0.659065215	4.82693E-43
ENSMUSG00000037206	Islr	-0.949716911	4.66755E-22	ENSMUSG00000025036	Sfxn2	-0.659206049	9.18254E-13
ENSMUSG00000061882	Ccdc62	-0.950168911	0.027484383	ENSMUSG00000028693	Nasp	-0.659248719	1.1209E-33
ENSMUSG00000040957	Cables1	-0.950559547	1.84132E-05	ENSMUSG00000041187	Prkd2	-0.659301427	8.10672E-17
ENSMUSG00000109904	Gm45819	-0.950562222	0.006822165	ENSMUSG00000112478	Gm47761	-0.659406643	0.021845928
ENSMUSG00000021367	Edn1	-0.951611381	1.12898E-29	ENSMUSG00000034659	Tmem109	-0.659885574	4.93398E-35
ENSMUSG00000021485	Mxd3	-0.951668197	6.90525E-08	ENSMUSG00000018800	Abca5	-0.660100548	2.65079E-12
ENSMUSG00000052485	Tmem171	-0.951978121	0.000156007	ENSMUSG00000020627	Klhl29	-0.660244311	1.34237E-11
ENSMUSG00000002006	Pdzd4	-0.952491378	2.91349E-07	ENSMUSG00000037509	Arhgef4	-0.660304337	0.001431174
ENSMUSG00000037544	Dlgap5	-0.952997918	3.89758E-61	ENSMUSG00000024640	Psat1	-0.661067101	1.55776E-60
ENSMUSG00000020785	Camkk1	-0.953224517	1.56085E-10	ENSMUSG00000048058	Ldlrad3	-0.66171728	1.21151E-08
ENSMUSG00000027132	Katnbl1	-0.953871277	2.44538E-77	ENSMUSG00000002814	Top3a	-0.661898818	1.39078E-09
ENSMUSG00000053475	Tnfaip6	-0.953888332	1.71172E-31	ENSMUSG00000054893	Zfp667	-0.661930864	0.036456824
ENSMUSG00000038042	Ptpdc1	-0.954467877	2.46007E-22	ENSMUSG00000034724	Cnot6l	-0.661966183	9.769E-46
ENSMUSG00000022708	Zbtb20	-0.955614168	5.10107E-93	ENSMUSG00000018672	Copz2	-0.66214444	8.61388E-29
ENSMUSG00000040430	Pitpnc1	-0.959616811	1.8832E-08	ENSMUSG00000036617	Etl4	-0.662755927	4.22767E-09
ENSMUSG00000040260	Daam2	-0.960450428	6.77257E-11	ENSMUSG00000022296	Baalc	-0.662836246	0.002201229
ENSMUSG00000027381	Bcl2l11	-0.961609361	4.06157E-14	ENSMUSG00000059834	Sclt1	-0.663269299	4.06845E-07
ENSMUSG00000099583	H3c4	-0.962462758	0.022397807	ENSMUSG00000022090	Pdlim2	-0.663341781	3.20553E-55
ENSMUSG00000079017	Ifi27l2a	-0.96401739	5.96174E-18	ENSMUSG00000079003	Samd1	-0.664475909	2.39605E-09
ENSMUSG00000063268	Parp10	-0.96436794	1.33389E-19	ENSMUSG00000102602	A930004J17Rik	-0.664564678	0.000224455
ENSMUSG00000015217	Hmgb3	-0.96439993	3.89295E-16	ENSMUSG00000001518	Itfg2	-0.665054659	3.98515E-09
ENSMUSG00000064141	Zfp69	-0.965075204	0.033074269	ENSMUSG00000057706	Mex3b	-0.665525671	7.92173E-09
ENSMUSG00000028497	Hacd4	-0.965489484	9.52579E-70	ENSMUSG00000072568	Lratd2	-0.66592599	5.70141E-25
ENSMUSG00000045636	Mtus1	-0.965612505	4.14357E-09	ENSMUSG00000024486	Hbegf	-0.666119508	4.61209E-71
ENSMUSG00000027293	Ehd4	-0.965872117	2.2262E-105	ENSMUSG00000028960	Ube4b	-0.666133785	5.20577E-67
ENSMUSG00000050558	Prokr2	-0.966376372	0.001409187	ENSMUSG00000034485	Uaca	-0.666343644	1.69423E-60
ENSMUSG00000032221	Mns1	-0.967795805	1.55351E-17	ENSMUSG00000019278	Dpep1	-0.666413647	6.65127E-21
ENSMUSG00000041064	Pif1	-0.969319836	1.90523E-33	ENSMUSG00000081738	Hmgb1-ps2	-0.666502786	0.030868309
ENSMUSG00000049307	Fut4	-0.970087425	4.77479E-10	ENSMUSG00000021318	Gli3	-0.666876669	4.39943E-27
ENSMUSG00000067297	Ifit1bl2	-0.970593575	0.018418549	ENSMUSG00000026565	Pou2f1	-0.667485905	1.36331E-12
ENSMUSG00000057706	Mex3b	-0.97087525	1.90025E-19	ENSMUSG00000040488	Ltbp4	-0.667860006	4.20829E-22
ENSMUSG00000030867	Plk1	-0.97127937	5.4282E-103	ENSMUSG00000043333	Rhbdl2	-0.667956758	0.004075733
ENSMUSG00000021453	Gadd45g	-0.972609339	3.37315E-30	ENSMUSG00000022533	Atp13a3	-0.668298701	1.3145E-106
ENSMUSG00000025478	Dpysl4	-0.972689225	0.035133258	ENSMUSG00000047344	Lancl3	-0.668451387	1.80233E-09
ENSMUSG00000032224	Fam81a	-0.972693751	0.040323481	ENSMUSG00000050212	Eva1b	-0.668767429	5.93162E-25
ENSMUSG00000075502	Kbtbd6	-0.973584416	0.000612574	ENSMUSG00000021185	Dglucy	-0.669358951	3.15038E-37
ENSMUSG00000026443	Lrrn2	-0.974480031	7.2428E-05	ENSMUSG00000104063	Pcdhgb7	-0.670492178	0.002593285
ENSMUSG00000093445	Lrch4	-0.974527608	0.015352713	ENSMUSG00000034998	Foxn2	-0.671580354	3.69486E-36
ENSMUSG00000037892	Pcdh18	-0.976202415	9.39521E-31	ENSMUSG00000021175	Cdca7l	-0.671765688	1.99014E-05
ENSMUSG00000036040	Adamtsl2	-0.977075496	7.28003E-05	ENSMUSG00000057835	Zfp119a	-0.671784834	0.002466915
ENSMUSG00000048458	Inka2	-0.977355516	1.22955E-34	ENSMUSG00000050240	Hic2	-0.672237667	0.001137106
ENSMUSG00000000275	Trim25	-0.977814039	2.49375E-13	ENSMUSG00000038126	Mphosph9	-0.672344283	7.10847E-13
ENSMUSG00000006205	Htra1	-0.978446237	1.5548E-119	ENSMUSG00000020290	Xpo1	-0.672597442	5.78991E-82
ENSMUSG00000030465	Psd3	-0.978574898	2.179E-68	ENSMUSG00000031216	Stard8	-0.672680836	2.09054E-24
ENSMUSG00000021719	Rgs7bp	-0.979174587	0.023834709	ENSMUSG00000007646	Rad51c	-0.673228373	0.022555783
ENSMUSG00000091144	Phf11c	-0.979190746	8.88749E-05	ENSMUSG00000020739	Nup85	-0.673359131	2.23032E-24
ENSMUSG00000053414	Hunk	-0.979367532	0.02959908	ENSMUSG00000115222	Gm49747	-0.673413027	0.001746882
ENSMUSG00000031378	Abcd1	-0.980688237	1.43025E-24	ENSMUSG00000038205	Prkab2	-0.673999866	4.03916E-34
ENSMUSG00000019577	Pdk4	-0.980858554	7.59899E-29	ENSMUSG00000025358	Cdk2	-0.674755475	2.29545E-25
ENSMUSG00000036617	Etl4	-0.980923363	1.32054E-15	ENSMUSG00000006342	Susd2	-0.674776849	2.81621E-06
ENSMUSG00000022353	Mtss1	-0.982488207	2.87334E-18	ENSMUSG00000024098	Twsg1	-0.675255801	1.06407E-88
ENSMUSG00000020086	Macroh2a2	-0.98279426	8.12144E-19	ENSMUSG00000038150	Ormdl3	-0.675968126	8.49918E-35
ENSMUSG00000028339	Col15a1	-0.983267443	2.60969E-36	ENSMUSG00000027752	Exosc8	-0.67609737	2.27417E-16
ENSMUSG00000029438	Bcl7a	-0.984028823	5.79314E-17	ENSMUSG00000054263	Lifr	-0.677343686	8.08709E-14
ENSMUSG00000097467	Gm26737	-0.985293485	9.01454E-06	ENSMUSG00000044328	Trp53i13	-0.678321681	1.8646E-06
ENSMUSG00000036223	Ska1	-0.986077928	7.14039E-08	ENSMUSG00000086370	Ftx	-0.678546394	0.001310291
ENSMUSG00000001604	Tcea3	-0.98845805	2.05798E-13	ENSMUSG00000007589	Tinf2	-0.678706885	1.76257E-15
ENSMUSG00000056144	Trim34a	-0.989145578	9.76496E-12	ENSMUSG00000030770	Parva	-0.678798034	4.2739E-119
ENSMUSG00000053477	Tcf4	-0.989730357	8.7073E-148	ENSMUSG00000054057	A930004D18Rik	-0.678948087	1.27053E-06
ENSMUSG00000046818	Ddit4l	-0.991127389	1.47134E-31	ENSMUSG00000002222	Rmnd5a	-0.678972134	3.17458E-52
ENSMUSG00000039989	Cbx4	-0.991296384	2.00135E-26	ENSMUSG00000023953	Polh	-0.679247638	5.52491E-31
ENSMUSG00000021379	Id4	-0.991611568	1.9059E-09	ENSMUSG00000041483	Zfp281	-0.67952162	6.45297E-37
ENSMUSG00000110185	Igip	-0.992271495	2.78038E-24	ENSMUSG00000035121	Neil2	-0.680198069	0.034213986
ENSMUSG00000026896	Ifih1	-0.992319551	5.42285E-07	ENSMUSG00000051146	Camk2n2	-0.680311358	0.000891325
ENSMUSG00000044362	Ccdc89	-0.993217545	0.007981711	ENSMUSG00000091549	Gm6548	-0.68150567	1.25321E-10
ENSMUSG00000031137	Fgf13	-0.993463653	0.001978819	ENSMUSG00000020831	0610010K14Rik	-0.681514702	0.014177552
ENSMUSG00000009654	Oit3	-0.993558202	2.3236E-05	ENSMUSG00000020156	Pwwp3a	-0.681855044	6.7038E-24
ENSMUSG00000036264	Fstl4	-0.99401009	0.011867678	ENSMUSG00000019214	Chtf18	-0.682107533	3.66105E-08
ENSMUSG00000066800	Rnasel	-0.994033542	1.67635E-20	ENSMUSG00000009731	Kcnd1	-0.682332006	0.002197084
ENSMUSG00000028799	Zfp362	-0.994233763	2.12844E-21	ENSMUSG00000050945	Zfp438	-0.683002044	0.004723727
ENSMUSG00000054408	Spcs3	-0.995575343	0	ENSMUSG00000021998	Lcp1	-0.683695435	6.89689E-08
ENSMUSG00000028681	Ptch2	-0.996761211	0.002310726	ENSMUSG00000029863	Casp2	-0.683850487	4.39232E-13
ENSMUSG00000020475	Pgam2	-0.997718323	4.18022E-05	ENSMUSG00000032042	Srpr	-0.684046712	4.0975E-166

ENSMUSG00000057329	Bcl2	-0.999151067	4.17034E-34
ENSMUSG00000027684	Mecom	-0.999209075	1.36702E-46
ENSMUSG00000026589	Sec16b	-1.000197926	2.33075E-22
ENSMUSG00000054342	Kcnn4	-1.0003606	0.047983192
ENSMUSG00000031756	Cenpn	-1.001997657	4.67239E-26
ENSMUSG00000037108	Zcwpw1	-1.002645356	0.039300321
ENSMUSG00000035683	Melk	-1.002671852	1.11126E-39
ENSMUSG00000041992	Rapgef5	-1.003037802	9.3935E-32
ENSMUSG00000087006	Gm13889	-1.004588744	2.84447E-05
ENSMUSG00000021464	Ror2	-1.005865526	6.99774E-19
ENSMUSG00000093930	Hmgcs1	-1.007049132	1.53694E-85
ENSMUSG00000082163	Gm14276	-1.008011092	0.010688462
ENSMUSG00000020357	Flt4	-1.008086247	4.05789E-67
ENSMUSG00000069520	Tmem19	-1.009656214	2.69932E-56
ENSMUSG00000045672	Col27a1	-1.010162553	2.84257E-12
ENSMUSG00000079481	Nhsl2	-1.010194746	3.74744E-07
ENSMUSG00000097194	9330175E14Rik	-1.010593084	0.036296673
ENSMUSG00000024065	Ehd3	-1.010872621	5.26033E-33
ENSMUSG00000014778	Fhod1	-1.01189698	1.12217E-29
ENSMUSG00000097684	Gm26645	-1.012078791	0.038175137
ENSMUSG00000028182	Lrriq3	-1.014739813	0.04051293
ENSMUSG00000035829	Ppp1r26	-1.015537292	0.027736843
ENSMUSG00000004151	Etv1	-1.016037293	1.46824E-26
ENSMUSG00000034957	Cebpa	-1.01638285	1.51593E-06
ENSMUSG00000034422	Parp14	-1.016489954	2.36113E-08
ENSMUSG00000052776	Oas1a	-1.020188966	0.009255338
ENSMUSG00000027763	Mbnl1	-1.021812938	1.091E-258
ENSMUSG00000050541	Adra1b	-1.023225264	0.000302706
ENSMUSG00000021388	Aspn	-1.023692427	1.51256E-19
ENSMUSG00000017830	Dhx58	-1.024699971	5.0154E-10
ENSMUSG00000021811	Dnajc9	-1.026144731	6.55143E-65
ENSMUSG00000097910	5033428I22Rik	-1.026372283	2.78917E-41
ENSMUSG00000023927	Satb1	-1.028690408	1.87032E-06
ENSMUSG00000046449	Nexmif	-1.028807123	2.51503E-08
ENSMUSG00000030541	Idh2	-1.028818345	7.4091E-92
ENSMUSG00000020948	Klhl28	-1.029895102	8.61234E-60
ENSMUSG00000070425	Xntrpc	-1.031628256	0.033258428
ENSMUSG00000039377	Hlx	-1.032037757	2.28976E-08
ENSMUSG00000024534	Sncaip	-1.032506536	0.000331704
ENSMUSG00000042807	Hecw2	-1.032512518	1.57092E-32
ENSMUSG00000028873	Cdca8	-1.033066426	3.52449E-51
ENSMUSG00000029217	Tec	-1.033485979	1.5597E-10
ENSMUSG00000056602	Fry	-1.033856755	2.80802E-09
ENSMUSG00000079553	Kifc1	-1.034329981	4.81966E-73
ENSMUSG00000064043	Trerf1	-1.035495414	2.1064E-18
ENSMUSG00000027695	Pld1	-1.0359916	2.23056E-39
ENSMUSG00000023206	Il15ra	-1.036221611	3.96094E-09
ENSMUSG00000029096	Htra3	-1.037224448	1.54942E-14
ENSMUSG00000030064	Frmd4b	-1.040517042	1.31213E-18
ENSMUSG00000021676	Iqgap2	-1.040619223	5.08001E-09
ENSMUSG00000047180	Neurl3	-1.040864104	7.83116E-14
ENSMUSG00000057596	Trim30d	-1.041175266	0.000643813
ENSMUSG00000034858	Fam214a	-1.042173848	1.37817E-46
ENSMUSG00000043157	Arl11	-1.043189608	0.004938561
ENSMUSG00000021373	Cap2	-1.046358924	1.99676E-14
ENSMUSG00000085586	Gm11613	-1.047720297	0.000514956
ENSMUSG00000070720	Tmem200b	-1.049722614	7.26556E-09
ENSMUSG00000043008	Klhl6	-1.050288119	2.2355E-108
ENSMUSG00000017314	Mpp2	-1.050346584	2.00264E-17
ENSMUSG00000040624	Plekhg1	-1.051327973	4.17086E-17
ENSMUSG00000035711	Dok3	-1.051404423	0.008168981
ENSMUSG00000097006	9530082P21Rik	-1.053085086	0.000167314
ENSMUSG00000017692	Rhbdl3	-1.054233148	0.005127341
ENSMUSG00000043881	Kbtbd7	-1.055495265	4.96239E-19
ENSMUSG00000019851	Perp	-1.055868479	9.50042E-20
ENSMUSG00000031530	Dusp4	-1.056658026	2.4585E-228
ENSMUSG00000097789	Gm2115	-1.057168831	2.11443E-05
ENSMUSG00000001021	S100a3	-1.061077677	0.000124319
ENSMUSG00000029536	Gatc	-1.061549937	7.46136E-29
ENSMUSG00000005410	Mcm5	-1.062657035	3.45091E-93
ENSMUSG00000022885	St6gal1	-1.062695291	8.94318E-08
ENSMUSG00000062960	Kdr	-1.063058976	1.15524E-10
ENSMUSG00000046997	Spsb4	-1.063715777	0.000180294
ENSMUSG00000039716	Dock3	-1.064759547	2.34792E-06
ENSMUSG00000036109	Mbnl3	-1.065363067	5.1599E-14
ENSMUSG00000052584	Serp2	-1.066093026	0.010826621
ENSMUSG00000034485	Uaca	-1.067423459	1.7349E-123
ENSMUSG00000078496	Zfp982	-0.684136409	0.015663174
ENSMUSG00000090626	Tex9	-0.684186884	0.000442063
ENSMUSG00000064326	Siva1	-0.684640657	8.21765E-12
ENSMUSG00000054237	Fra10ac1	-0.685114308	2.954E-09
ENSMUSG00000021763	BC067074	-0.685840352	8.29507E-08
ENSMUSG00000039470	Zdhhc2	-0.685957005	1.8174E-29
ENSMUSG00000041261	Car8	-0.686053182	1.44327E-27
ENSMUSG00000006715	Gmnn	-0.686970742	1.09811E-10
ENSMUSG00000022428	Cby1	-0.686979141	5.34008E-10
ENSMUSG00000031697	Orc6	-0.687467078	9.46894E-24
ENSMUSG00000041797	Abca9	-0.687546516	9.5706E-15
ENSMUSG00000021253	Tgfb3	-0.687685555	1.98031E-57
ENSMUSG00000020594	Pum2	-0.688375531	3.7177E-65
ENSMUSG00000019817	Plagl1	-0.688918938	2.38633E-09
ENSMUSG00000031626	Sorbs2	-0.689315637	1.33724E-58
ENSMUSG00000022236	Ropn1l	-0.689772748	0.00836703
ENSMUSG00000029472	Anapc5	-0.690538304	3.685E-109
ENSMUSG00000024151	Msh2	-0.690567232	5.53165E-11
ENSMUSG00000033157	Abhd10	-0.690984916	2.39841E-08
ENSMUSG00000022391	Rangap1	-0.691388841	2.40972E-87
ENSMUSG00000028207	Asph	-0.69139559	4.62978E-74
ENSMUSG00000060121	Gemin2	-0.692673825	8.55561E-07
ENSMUSG00000072621	Slfn10-ps	-0.692675115	0.001919499
ENSMUSG00000041215	Yeats2	-0.693142096	2.16789E-32
ENSMUSG00000035914	Cd276	-0.693400696	4.64345E-22
ENSMUSG00000057133	Chd6	-0.693544614	1.38004E-21
ENSMUSG00000028795	Ccdc28b	-0.693848727	0.000234889
ENSMUSG00000053080	2700081O15Rik	-0.694317033	1.09569E-15
ENSMUSG00000027397	Slc20a1	-0.694924209	1.20173E-75
ENSMUSG00000085334	Gm12940	-0.695150455	9.45892E-06
ENSMUSG00000030123	Plxnd1	-0.695448564	9.83491E-65
ENSMUSG00000064061	Dzip3	-0.695557333	2.19555E-20
ENSMUSG00000023885	Thbs2	-0.696692671	3.9534E-148
ENSMUSG00000007827	Ankrd26	-0.697278077	5.03587E-10
ENSMUSG00000030613	Ccdc90b	-0.697322792	4.87112E-15
ENSMUSG00000097415	AU020206	-0.698211409	4.55115E-07
ENSMUSG00000034825	Nrip3	-0.698403997	0.017374007
ENSMUSG00000043015	Nemp2	-0.698728621	0.000369322
ENSMUSG00000028251	Tstd3	-0.698852985	3.84833E-12
ENSMUSG00000015749	Anp32e	-0.699757343	6.97221E-40
ENSMUSG00000027242	Wdr76	-0.70053626	7.08549E-07
ENSMUSG00000002228	Ppm1j	-0.70054469	0.028127687
ENSMUSG00000031683	Lsm6	-0.70061042	3.03082E-12
ENSMUSG00000058420	Syt17	-0.700829556	7.57645E-34
ENSMUSG00000034610	Tut4	-0.700851005	6.24338E-28
ENSMUSG00000078452	Raet1d	-0.701396573	4.4565E-17
ENSMUSG00000037216	Lipt1	-0.701549782	0.00100843
ENSMUSG00000031482	Slc25a15	-0.701560961	4.25981E-19
ENSMUSG00000034684	Sema3f	-0.701816522	1.90344E-14
ENSMUSG00000053198	Prx	-0.702055542	1.54931E-05
ENSMUSG00000039055	Eme1	-0.702075822	0.015729233
ENSMUSG00000052727	Map1b	-0.702523012	1.53097E-19
ENSMUSG00000029569	Tmem168	-0.702844865	6.28768E-25
ENSMUSG00000040618	Pck2	-0.703334603	4.76995E-27
ENSMUSG00000030787	Lyve1	-0.703651798	0.001311005
ENSMUSG00000042439	Zfp532	-0.703915057	1.00085E-23
ENSMUSG00000029084	Cd38	-0.704581983	1.92938E-07
ENSMUSG00000056665	Them6	-0.704922178	0.000803018
ENSMUSG00000044703	Phf11a	-0.706360255	0.041325209
ENSMUSG00000029380	Cxcl1	-0.706430957	2.31612E-69
ENSMUSG00000031652	N4bp1	-0.706481416	1.62854E-51
ENSMUSG00000030093	Wnt7a	-0.706763504	8.32647E-11
ENSMUSG00000040785	Ttc3	-0.706892483	1.25496E-49
ENSMUSG00000047443	Erfe	-0.707310912	3.54262E-05
ENSMUSG00000035916	Ptprq	-0.708048379	1.372E-09
ENSMUSG00000025384	Faap100	-0.708217326	8.56082E-09
ENSMUSG00000019975	Ikbip	-0.708240931	8.3197E-108
ENSMUSG00000091811	Inafm1	-0.708286049	1.05549E-08
ENSMUSG00000050910	Cdr2l	-0.708834012	3.34371E-48
ENSMUSG00000040690	Col16a1	-0.709246133	3.54878E-87
ENSMUSG00000028270	Gbp2	-0.709293346	0.001308764
ENSMUSG00000052917	Senp7	-0.709972252	5.58561E-10
ENSMUSG00000037533	Rapgef6	-0.7100032	1.41943E-35
ENSMUSG00000021754	Map3k1	-0.710743895	6.84723E-10
ENSMUSG00000103793	Pcdhga6	-0.710980406	0.001099367
ENSMUSG00000031728	Zfp821	-0.711665197	2.51474E-06
ENSMUSG00000023805	Synj2	-0.71184375	3.38643E-35

ENSMUSG00000040918	Slc19a2	-1.067988507	4.8505E-111
ENSMUSG00000030966	Trim21	-1.069908048	4.07865E-21
ENSMUSG00000105112	Gm42778	-1.072416451	0.015357804
ENSMUSG00000071359	Tbpl1	-1.073110892	1.21679E-42
ENSMUSG00000024039	Cbs	-1.073707535	0.029553796
ENSMUSG00000033781	Asb13	-1.076233042	5.75183E-15
ENSMUSG00000037685	Atp8a1	-1.07757559	7.95447E-08
ENSMUSG00000021047	Nova1	-1.081375528	3.22611E-05
ENSMUSG00000043051	Disc1	-1.081481978	0.028791437
ENSMUSG00000020493	Prr11	-1.085433882	6.1289E-104
ENSMUSG00000030313	Dennd5b	-1.085462987	1.75084E-30
ENSMUSG00000007591	Tssk4	-1.085946242	0.033117485
ENSMUSG00000063286	Gm8995	-1.086309015	7.17269E-08
ENSMUSG00000099032	Tcf24	-1.087176566	6.91059E-07
ENSMUSG00000106025	Gm42940	-1.087413802	0.012799921
ENSMUSG00000040483	Xaf1	-1.087533236	4.45005E-06
ENSMUSG00000030319	Cand2	-1.089264618	4.45495E-46
ENSMUSG00000023015	Racgap1	-1.090294558	5.5272E-160
ENSMUSG00000027333	Smox	-1.092374599	1.55655E-32
ENSMUSG00000023972	Ptk7	-1.093550529	1.8109E-130
ENSMUSG00000034457	Eda2r	-1.093622987	1.218E-173
ENSMUSG00000025938	Slco5a1	-1.094520795	0.006464181
ENSMUSG00000000253	Gmpr	-1.094707081	2.83284E-11
ENSMUSG00000034203	Chchd4	-1.096162029	1.61903E-20
ENSMUSG00000017417	Plxdc1	-1.098183973	0.016576577
ENSMUSG00000075304	Sp5	-1.098659963	0.028206409
ENSMUSG00000028243	Ubxn2b	-1.098762378	9.63178E-21
ENSMUSG00000030616	Sytl2	-1.098879886	1.60605E-25
ENSMUSG00000020808	Pimreg	-1.099089616	1.92796E-39
ENSMUSG00000022428	Cby1	-1.099850871	4.22045E-15
ENSMUSG00000036986	Pml	-1.10087534	5.62116E-59
ENSMUSG00000054013	Tmem179	-1.101049415	0.044485033
ENSMUSG00000022220	Adcy4	-1.101677999	9.60372E-34
ENSMUSG00000000782	Tcf7	-1.10175527	0.00152535
ENSMUSG00000045658	Pid1	-1.102368971	3.26885E-15
ENSMUSG00000044201	Cdc25c	-1.105546767	5.41866E-21
ENSMUSG00000039450	Dcxr	-1.108582523	6.93398E-36
ENSMUSG00000004371	Il11	-1.108893617	2.7246E-06
ENSMUSG00000049493	Pls1	-1.11027014	0.001522921
ENSMUSG00000048388	Fam171b	-1.110379087	3.57419E-92
ENSMUSG00000078920	Ifi47	-1.111121976	1.14872E-44
ENSMUSG00000046808	Atp10d	-1.111188986	2.06898E-12
ENSMUSG00000036553	Sh3tc1	-1.111789246	1.28601E-09
ENSMUSG00000037709	Fam13a	-1.112869095	0.000621574
ENSMUSG00000090215	Trim34b	-1.113283224	0.032780689
ENSMUSG00000026414	Tnnt2	-1.11333706	2.67712E-80
ENSMUSG00000052415	Tchh	-1.114819369	7.34718E-06
ENSMUSG00000031780	Ccl17	-1.114912383	0.000159762
ENSMUSG00000040852	Plekhh2	-1.115486091	2.0222E-99
ENSMUSG00000022836	Mylk	-1.116155878	2.96324E-14
ENSMUSG00000025551	Fgf14	-1.117334834	0.000204501
ENSMUSG00000021319	Sfrp4	-1.118812577	3.48647E-09
ENSMUSG00000021803	Cdhr1	-1.119805328	6.09879E-05
ENSMUSG00000037855	Zfp365	-1.120130056	6.621E-116
ENSMUSG00000003134	Tbc1d8	-1.121771055	0.0001297
ENSMUSG00000033083	Tbc1d4	-1.121849469	1.21023E-28
ENSMUSG00000062309	Rpp25	-1.123099527	0.000132983
ENSMUSG00000073293	Nudt10	-1.12337886	0.008996447
ENSMUSG00000097245	Gm5421	-1.123536435	0.010688462
ENSMUSG00000038173	Enpp6	-1.124133907	0.000397368
ENSMUSG00000016087	Fli1	-1.125683643	3.02249E-36
ENSMUSG00000022602	Arc	-1.12588146	1.64148E-10
ENSMUSG00000028524	Sgip1	-1.126120318	8.96662E-07
ENSMUSG00000047881	Rell1	-1.126160445	5.01325E-41
ENSMUSG00000109783	Gm45338	-1.127507313	0.011197185
ENSMUSG00000025867	Cplx2	-1.127831873	1.09986E-55
ENSMUSG00000032010	Usp2	-1.128312778	8.60686E-23
ENSMUSG00000079018	Ly6c1	-1.129064089	5.72111E-05
ENSMUSG00000025240	Sacm1l	-1.130176879	1.4451E-163
ENSMUSG00000069793	Slfn9	-1.133853471	6.84847E-66
ENSMUSG00000031538	Plat	-1.133929983	2.0002E-150
ENSMUSG00000042684	Npl	-1.134360273	0.017963056
ENSMUSG00000025498	Irf7	-1.135444597	0.00035387
ENSMUSG00000027715	Ccna2	-1.135472349	2.5525E-128
ENSMUSG00000022240	Ctnnd2	-1.138518438	1.06907E-09
ENSMUSG00000043165	Lor	-1.139494445	2.66114E-05
ENSMUSG00000045102	Poln	-1.140882255	0.00853692

ENSMUSG00000029246	Ppat	-0.712142093	8.02456E-17
ENSMUSG00000001576	Ergic1	-0.712398115	9.85184E-85
ENSMUSG00000062980	Cped1	-0.713157185	8.99676E-69
ENSMUSG00000041559	Fmod	-0.713298449	1.24071E-29
ENSMUSG00000043065	Spice1	-0.714410304	0.000889456
ENSMUSG00000025198	Erlin1	-0.714644417	1.6204E-37
ENSMUSG00000040102	Klhl42	-0.714840661	6.25941E-21
ENSMUSG00000022070	Bora	-0.716362058	3.1134E-12
ENSMUSG00000000317	Bcl6b	-0.71682863	0.00046184
ENSMUSG00000032766	Gng11	-0.717736849	2.95958E-21
ENSMUSG00000012483	Rpa3	-0.717863376	1.67221E-09
ENSMUSG00000029283	Cdc7	-0.718249036	6.77207E-09
ENSMUSG00000021319	Sfrp4	-0.718437924	0.000102449
ENSMUSG00000049047	Armcx3	-0.718748397	7.69872E-53
ENSMUSG00000021703	Serinc5	-0.719065605	3.99777E-29
ENSMUSG00000079049	Serpinb1c	-0.719076921	0.013566333
ENSMUSG00000031885	Cbfb	-0.719560458	5.2539E-112
ENSMUSG00000042842	Serpinb6b	-0.719833486	2.88942E-40
ENSMUSG00000054823	Nsd3	-0.720003948	6.00982E-74
ENSMUSG00000027939	Nup210l	-0.720380836	0.037696822
ENSMUSG00000037490	Slc2a12	-0.721090366	0.008906925
ENSMUSG00000118559	A930007A09Rik	-0.721912415	3.75334E-07
ENSMUSG00000026604	Ptpn14	-0.722832256	5.09813E-61
ENSMUSG00000039753	Fbxl5	-0.722857247	3.30659E-40
ENSMUSG00000041801	Phlda3	-0.723439803	1.67361E-63
ENSMUSG00000087141	Plcxd2	-0.724075011	9.72289E-19
ENSMUSG00000062960	Kdr	-0.727658995	1.17311E-05
ENSMUSG00000071064	Zfp827	-0.728137661	3.23165E-09
ENSMUSG00000025316	Banp	-0.728243095	1.12844E-11
ENSMUSG00000060477	Irak2	-0.728308807	1.43612E-28
ENSMUSG00000021536	Adcy2	-0.729397749	0.001736644
ENSMUSG00000038056	Kmt2c	-0.729671396	1.89742E-16
ENSMUSG00000018819	Lsp1	-0.730407599	1.35482E-24
ENSMUSG00000035275	Raver2	-0.73068354	0.000562387
ENSMUSG00000098557	Kctd12	-0.731659486	2.3002E-08
ENSMUSG00000032372	Plscr2	-0.733428838	1.64039E-22
ENSMUSG00000032997	Chpf	-0.733977307	8.35744E-69
ENSMUSG00000030513	Pcsk6	-0.734632819	4.83789E-68
ENSMUSG00000012422	Tmem167	-0.735404616	1.43816E-78
ENSMUSG00000027883	Gpsm2	-0.735512856	3.60406E-20
ENSMUSG00000030327	Necap1	-0.735598648	9.8684E-50
ENSMUSG00000024965	Fermt3	-0.735742798	0.000587149
ENSMUSG00000052776	Oas1a	-0.736115294	2.48743E-05
ENSMUSG00000031129	Slc9a9	-0.736352215	8.18722E-12
ENSMUSG00000027695	Pld1	-0.738153017	4.1572E-24
ENSMUSG00000031828	Klhl36	-0.738297425	0.005231152
ENSMUSG00000040265	Dnm3	-0.738434548	0.032564485
ENSMUSG00000005951	Shpk	-0.73847812	0.003336729
ENSMUSG00000097141	Gm10524	-0.738756256	2.83566E-05
ENSMUSG00000085133	B930095G15Rik	-0.738916581	3.73543E-09
ENSMUSG00000014846	Tppp3	-0.738980666	0.038215476
ENSMUSG00000032396	Dis3l	-0.739275231	2.90049E-25
ENSMUSG00000032034	Kcnj5	-0.739700623	0.019334707
ENSMUSG00000025077	Dclre1a	-0.740153809	1.84938E-14
ENSMUSG00000051177	Plcb1	-0.74033262	5.41084E-09
ENSMUSG00000029463	Fam216a	-0.740607952	4.08261E-14
ENSMUSG00000108350	Gm44950	-0.741747278	0.000123366
ENSMUSG00000063605	Ccdc102a	-0.74183562	2.53057E-16
ENSMUSG00000038780	Smurf1	-0.742470754	1.1326E-122
ENSMUSG00000037795	N4bp2	-0.742546098	3.52481E-17
ENSMUSG00000073791	Efcab7	-0.742755144	0.013891952
ENSMUSG00000037643	Prkci	-0.743037792	2.80602E-57
ENSMUSG00000040037	Negr1	-0.743161446	0.006390234
ENSMUSG00000026594	Ralgps2	-0.743393315	9.80722E-12
ENSMUSG00000020362	Cnot6	-0.743557024	1.1241E-128
ENSMUSG00000058163	Gm5431	-0.743958448	0.001175068
ENSMUSG00000024590	Lmnb1	-0.744129522	7.04071E-44
ENSMUSG00000053916	Nanp	-0.744148448	7.06876E-10
ENSMUSG00000068394	Cep152	-0.744652266	1.58962E-08
ENSMUSG00000028944	Prkag2	-0.744959246	3.78658E-20
ENSMUSG00000037316	Bag4	-0.745112888	1.35059E-10
ENSMUSG00000050295	Foxc1	-0.745120186	5.50269E-18
ENSMUSG00000036639	Nudt1	-0.745202713	0.000432255
ENSMUSG00000019960	Dusp6	-0.745710864	3.06081E-63
ENSMUSG00000029370	Rassf6	-0.746130624	3.13173E-08
ENSMUSG00000020154	Ptprb	-0.746480419	1.05763E-63
ENSMUSG00000017724	Etv4	-0.747070752	3.68554E-13

ENSMUSG00000037419	Endod1	-1.143123102	3.80867E-97
ENSMUSG00000004891	Nes	-1.145281337	3.6071E-197
ENSMUSG00000027454	Gins1	-1.149616187	1.03189E-10
ENSMUSG00000040086	Tnni3k	-1.150917903	5.21374E-07
ENSMUSG00000052331	Ankrd44	-1.153375924	3.5621E-24
ENSMUSG00000010651	Acaa1b	-1.154067577	0.001518225
ENSMUSG00000003585	Sec14l2	-1.155290584	5.16767E-14
ENSMUSG00000026640	Plxna2	-1.155864491	4.14592E-69
ENSMUSG00000021990	Spata13	-1.157038425	4.96911E-19
ENSMUSG00000027508	Pag1	-1.158678244	3.43776E-25
ENSMUSG00000030921	Trim30a	-1.161448026	0.000410352
ENSMUSG00000066363	Serpina3f	-1.161913484	5.36722E-34
ENSMUSG00000038305	Spats2l	-1.165877277	2.49649E-15
ENSMUSG00000027765	P2ry1	-1.16660469	1.39392E-10
ENSMUSG00000032596	Uba7	-1.166719104	2.8471E-08
ENSMUSG00000028884	Rpa2	-1.167187307	8.74809E-40
ENSMUSG00000013033	Adgrl1	-1.168715065	8.7424E-74
ENSMUSG00000043333	Rhbdl2	-1.168915416	4.05047E-07
ENSMUSG00000079049	Serpinb1c	-1.169812558	3.02951E-05
ENSMUSG00000027107	Chrna1	-1.17366722	8.2333E-09
ENSMUSG00000099974	Bcl2a1d	-1.173910376	0.018273519
ENSMUSG00000037826	Ppm1k	-1.178425413	8.16218E-16
ENSMUSG00000032218	Ccnb2	-1.18005824	9.6889E-130
ENSMUSG00000028005	Gucy1b1	-1.180342456	6.9425E-194
ENSMUSG00000037977	6430571L13Rik	-1.180658266	0.04649849
ENSMUSG00000039114	Nrn1	-1.181343496	1.60389E-25
ENSMUSG00000038855	Itpkb	-1.181540735	4.41641E-83
ENSMUSG00000020330	Hmmr	-1.184042978	2.63603E-97
ENSMUSG00000052516	Robo2	-1.188498658	2.26975E-11
ENSMUSG00000048799	Cep120	-1.188602756	1.114E-105
ENSMUSG00000052889	Prkcb	-1.192130376	0.008495038
ENSMUSG00000087691	Cd55os	-1.193627579	4.44626E-08
ENSMUSG00000038816	Ctnnal1	-1.194208803	2.0164E-53
ENSMUSG00000021176	Efcab11	-1.195533368	1.86747E-07
ENSMUSG00000028568	Btf3l4	-1.196877458	2.8111E-105
ENSMUSG00000028965	Tnfrsf9	-1.198216897	5.46887E-05
ENSMUSG00000037628	Cdkn3	-1.199176058	2.91756E-21
ENSMUSG00000063060	Sox7	-1.199586771	1.57372E-16
ENSMUSG00000013089	Etv5	-1.205435983	1.03526E-60
ENSMUSG00000025076	Casp7	-1.20546956	7.03187E-43
ENSMUSG00000039556	Ppp1r3f	-1.206275543	0.000146806
ENSMUSG00000039853	Trim14	-1.209473193	0.008143144
ENSMUSG00000024640	Psat1	-1.209905033	5.4264E-120
ENSMUSG00000037321	Tap1	-1.212571392	1.06501E-26
ENSMUSG00000103473	Gm37696	-1.21310097	0.016520561
ENSMUSG00000026548	Slamf9	-1.213686679	5.67795E-08
ENSMUSG00000082676	Gm11843	-1.215166152	0.030722666
ENSMUSG00000030772	Dkk3	-1.215851978	1.23858E-87
ENSMUSG00000032122	Slc37a2	-1.218118649	6.88562E-14
ENSMUSG00000022758	P2rx6	-1.219295163	0.013653057
ENSMUSG00000021250	Fos	-1.219529518	6.89008E-17
ENSMUSG00000049357	Brd8dc	-1.220979662	0.011560574
ENSMUSG00000061887	Ssbp3	-1.221086766	1.57938E-64
ENSMUSG00000011263	Exoc3l2	-1.221293674	0.00672567
ENSMUSG00000079317	Trappc2	-1.222389541	1.41742E-41
ENSMUSG00000040037	Negr1	-1.222578437	2.84867E-05
ENSMUSG00000032135	Mcam	-1.22295265	1.01064E-32
ENSMUSG00000071862	Lrrtm2	-1.223065104	0.000709777
ENSMUSG00000044313	Mab21l3	-1.223628505	1.42547E-07
ENSMUSG00000025950	Idh1	-1.224559884	1.6298E-169
ENSMUSG00000029822	Osbpl3	-1.224801215	3.58767E-40
ENSMUSG00000015083	C8g	-1.227782702	0.015292237
ENSMUSG00000087370	Tmem170b	-1.229507489	1.16276E-28
ENSMUSG00000063232	Serpina11	-1.231219347	0.015252897
ENSMUSG00000050762	Prss27	-1.232060474	0.016014081
ENSMUSG00000026873	Phf19	-1.23477565	8.67313E-32
ENSMUSG00000020486	Septin4	-1.235860945	1.54534E-11
ENSMUSG00000025507	Pidd1	-1.238131152	1.74172E-37
ENSMUSG00000037253	Mex3c	-1.238245462	5.4707E-195
ENSMUSG00000024190	Dusp1	-1.241472076	1.6531E-105
ENSMUSG00000027574	Nkain4	-1.241891093	2.78933E-15
ENSMUSG00000087651	1500009L16Rik	-1.242573248	4.17235E-21
ENSMUSG00000049191	Rtl5	-1.24280779	1.9599E-47
ENSMUSG00000031506	Ptpn7	-1.24396636	0.00128723
ENSMUSG00000044469	Tnfaip8l1	-1.244347369	3.17269E-06
ENSMUSG00000062826	Ces2f	-1.251559837	0.007522127
ENSMUSG00000023000	Dhh	-1.252038703	0.034941675
ENSMUSG00000025903	Lypla1	-0.747421912	1.68974E-69
ENSMUSG00000035828	Pim3	-0.748760204	1.16454E-16
ENSMUSG00000002020	Ltbp2	-0.749612664	1.21665E-22
ENSMUSG00000033578	Tmem35a	-0.749812943	0.000353041
ENSMUSG00000075266	Cenpw	-0.749828079	2.49215E-06
ENSMUSG00000021668	Polk	-0.750687637	9.60575E-38
ENSMUSG00000036995	Asap3	-0.751194065	1.0027E-08
ENSMUSG00000056091	St3gal5	-0.751195439	1.23623E-30
ENSMUSG00000048756	Foxo3	-0.751847879	1.73589E-44
ENSMUSG00000024352	Spata24	-0.752090917	0.001419379
ENSMUSG00000067158	Col4a4	-0.752370268	2.36054E-13
ENSMUSG00000002980	Bcam	-0.752407504	2.24712E-21
ENSMUSG00000029617	Ccz1	-0.752823124	7.54594E-49
ENSMUSG00000030761	Myo7a	-0.753020423	2.23082E-34
ENSMUSG00000028184	Adgrl2	-0.753280046	1.06283E-37
ENSMUSG00000059142	Zfp945	-0.75408107	1.0171E-11
ENSMUSG00000097493	9930014A18Rik	-0.754137971	6.61106E-05
ENSMUSG00000027778	Ift80	-0.754252917	4.61327E-25
ENSMUSG00000060301	2610008E11Rik	-0.75444483	4.73021E-13
ENSMUSG00000021377	Dek	-0.755159439	1.89971E-64
ENSMUSG00000031636	Pdlim3	-0.755498027	2.70049E-19
ENSMUSG00000042333	Tnfrsf14	-0.755675372	0.006211988
ENSMUSG00000026688	Mgst3	-0.755874334	0.000206897
ENSMUSG00000097145	9230114K14Rik	-0.756829915	1.56546E-06
ENSMUSG00000036887	C1qa	-0.756954831	0.002212298
ENSMUSG00000042500	Ago4	-0.757199966	2.55255E-09
ENSMUSG00000026430	Rassf5	-0.757697897	1.22286E-14
ENSMUSG00000031872	Bean1	-0.757862853	7.95124E-08
ENSMUSG00000090115	Usp49	-0.758089116	1.57458E-05
ENSMUSG00000044452	Zfp507	-0.758333684	3.82537E-33
ENSMUSG00000054967	Zfp647	-0.758544467	0.004733143
ENSMUSG00000022960	Donson	-0.758677649	9.58797E-14
ENSMUSG00000068923	Syt11	-0.758956147	2.04048E-08
ENSMUSG00000001911	Nfix	-0.75908127	5.5029E-110
ENSMUSG00000040502	Marchf9	-0.760189499	0.009518305
ENSMUSG00000040423	Rc3h1	-0.760350499	3.06606E-59
ENSMUSG00000022742	Cpox	-0.761141609	2.36342E-48
ENSMUSG00000026222	Sp100	-0.761142835	5.33412E-06
ENSMUSG00000049521	Cdc42ep1	-0.761169285	3.47845E-57
ENSMUSG00000060260	Pwwp2b	-0.761742064	4.18641E-16
ENSMUSG00000031075	Ano1	-0.762777385	2.2912E-13
ENSMUSG00000028551	Cdkn2c	-0.763005578	1.0209E-32
ENSMUSG00000078994	Zfp429	-0.763490544	0.001022496
ENSMUSG00000012428	Steap4	-0.764934796	4.77397E-26
ENSMUSG00000029330	Cds1	-0.765213509	1.08668E-05
ENSMUSG00000038648	Creb3l2	-0.76562227	6.459E-167
ENSMUSG00000078153	Psme2b	-0.765891197	1.32323E-09
ENSMUSG00000043415	Otud1	-0.766126575	3.4056E-22
ENSMUSG00000029810	Tmem176b	-0.766184266	1.79304E-67
ENSMUSG00000075271	Ttc30a1	-0.766585779	0.000218098
ENSMUSG00000024135	Srbd1	-0.766736936	3.94364E-15
ENSMUSG00000085208	Brip1os	-0.766911923	8.15917E-78
ENSMUSG00000031595	Pdgfrl	-0.767657554	5.0309E-08
ENSMUSG00000022887	Masp1	-0.76767977	4.12127E-44
ENSMUSG00000067377	Tspan6	-0.767908375	6.26974E-72
ENSMUSG00000030643	Rab30	-0.768008859	1.59778E-13
ENSMUSG00000078651	Aoc2	-0.768295025	0.00012036
ENSMUSG00000029581	Fscn1	-0.768380561	9.1047E-111
ENSMUSG00000024065	Ehd3	-0.768409858	8.55515E-22
ENSMUSG00000090386	Mir99ahg	-0.768593154	2.09794E-08
ENSMUSG00000062248	Cks2	-0.769022315	1.28326E-36
ENSMUSG00000051041	Olfml1	-0.769325011	1.05965E-08
ENSMUSG00000068206	Pick1	-0.769362343	1.04766E-07
ENSMUSG00000015222	Map2	-0.770364918	1.10303E-29
ENSMUSG00000022369	Mtbp	-0.771456573	1.67276E-16
ENSMUSG00000034780	B3galt1	-0.771585506	8.3651E-09
ENSMUSG00000019851	Perp	-0.772064421	6.83336E-11
ENSMUSG00000046269	Usp27x	-0.772120333	1.56498E-12
ENSMUSG00000043557	Mdga1	-0.772294438	2.02811E-09
ENSMUSG00000046157	Tmem229b	-0.772684303	0.00130499
ENSMUSG00000031997	Trpc6	-0.772944424	1.76188E-08
ENSMUSG00000026238	Ptma	-0.773122851	5.8806E-223
ENSMUSG00000031538	Plat	-0.773427873	2.91555E-80
ENSMUSG00000047181	Samd14	-0.773641089	7.2975E-06
ENSMUSG00000015112	Slc25a13	-0.774002792	2.50463E-15
ENSMUSG00000036568	Bicral	-0.774264916	3.56299E-24
ENSMUSG00000040552	C3ar1	-0.774306292	0.00017653

ENSMUSG00000004633	Chn2	-1.252905061	7.77396E-11	ENSMUSG00000028497	Hacd4	-0.774385709	2.92257E-40
ENSMUSG00000027335	Adra1d	-1.253514672	0.020493378	ENSMUSG00000112324	Gm47939	-0.774609571	0.023993942
ENSMUSG00000055013	Agap1	-1.253988375	5.99614E-36	ENSMUSG00000107215	Gm43197	-0.77499099	3.03169E-05
ENSMUSG00000050556	Kcnb1	-1.254042331	0.000674156	ENSMUSG00000027188	Pamr1	-0.775170086	1.47137E-29
ENSMUSG00000022537	Tmem44	-1.254661687	0.000586067	ENSMUSG00000024501	Dpysl3	-0.775190519	3.94379E-97
ENSMUSG00000035208	Slfn8	-1.255393704	8.98111E-05	ENSMUSG00000105263	Gm42427	-0.775634431	0.001579945
ENSMUSG00000034522	Zfp395	-1.255801281	3.81416E-09	ENSMUSG00000021047	Nova1	-0.775761452	0.000376569
ENSMUSG00000066861	Oas1g	-1.256047783	0.000811955	ENSMUSG00000038685	Rtel1	-0.776550307	2.11235E-23
ENSMUSG00000000244	Tspan32	-1.256257913	0.012245545	ENSMUSG00000024206	Rfx2	-0.777365207	0.038878524
ENSMUSG00000045827	Serpinb9	-1.256523626	1.4136E-121	ENSMUSG00000018648	Dusp14	-0.777942196	0.000290162
ENSMUSG00000067818	Myl9	-1.25694508	8.87827E-81	ENSMUSG00000026994	Galnt3	-0.778244165	7.82821E-05
ENSMUSG00000037490	Slc2a12	-1.261941517	1.25017E-05	ENSMUSG00000043881	Kbtbd7	-0.778457916	3.26689E-10
ENSMUSG00000070034	Sp110	-1.263519714	1.07795E-20	ENSMUSG00000029376	Mthfd2l	-0.779167177	1.56156E-13
ENSMUSG00000055027	Smyd1	-1.263848028	0.001635159	ENSMUSG00000111514	E230014E18Rik	-0.779739329	0.025813277
ENSMUSG00000038007	Acer2	-1.266025859	9.71144E-30	ENSMUSG00000038886	Man2a2	-0.7803631	6.2512E-134
ENSMUSG00000038418	Egr1	-1.266730628	4.17235E-21	ENSMUSG00000021177	Tdp1	-0.781308673	1.77871E-11
ENSMUSG00000032769	Trpa1	-1.269719605	0.026329845	ENSMUSG00000003585	Sec14l2	-0.781666987	1.50504E-06
ENSMUSG00000026582	Sele	-1.269728276	6.553E-190	ENSMUSG00000020471	Pold2	-0.781735216	6.35092E-17
ENSMUSG00000005611	Mrvi1	-1.272411943	4.79756E-19	ENSMUSG00000028560	Usp1	-0.781792913	3.83721E-60
ENSMUSG00000019990	Pde7b	-1.273198117	6.38812E-37	ENSMUSG00000117050	1700023B13Rik	-0.782029692	0.010372884
ENSMUSG00000028037	Ifi44	-1.273490988	0.004248159	ENSMUSG00000057596	Trim30d	-0.782443221	0.011007491
ENSMUSG00000004880	Lbr	-1.274467222	1.1711E-116	ENSMUSG00000079109	Pms2	-0.782618545	1.05296E-11
ENSMUSG00000078349	AW011738	-1.275319199	5.79667E-07	ENSMUSG00000035783	Acta2	-0.782995654	2.8365E-270
ENSMUSG00000079429	Mroh2a	-1.275468114	4.47411E-11	ENSMUSG00000006344	Ggt5	-0.783242535	1.51017E-10
ENSMUSG00000051537	Gm5124	-1.275914772	0.007824791	ENSMUSG00000041762	Gpr155	-0.783355154	0.009085848
ENSMUSG00000026308	Klhl30	-1.278926533	2.43327E-14	ENSMUSG00000060671	Atp8b2	-0.783625681	1.4081E-130
ENSMUSG00000056476	Med12l	-1.279691337	0.01606776	ENSMUSG00000016984	Etaa1	-0.783802994	1.42248E-13
ENSMUSG00000047242	Taf9b	-1.280622373	5.23204E-21	ENSMUSG00000034487	Poglut3	-0.784562207	1.26771E-38
ENSMUSG00000006344	Ggt5	-1.280969715	2.56427E-19	ENSMUSG00000090641	Zfp712	-0.785051693	0.015761767
ENSMUSG00000031776	Arl2bp	-1.285875287	7.5118E-180	ENSMUSG00000003623	Crot	-0.785083507	2.38096E-33
ENSMUSG00000032511	Scn5a	-1.286909091	0.019297695	ENSMUSG00000073590	3222401L13Rik	-0.785870863	1.62406E-05
ENSMUSG00000027762	Sucnr1	-1.287417977	0.00345	ENSMUSG00000025135	Anapc11	-0.785916473	4.67995E-31
ENSMUSG00000029659	Medag	-1.287797346	2.7427E-238	ENSMUSG00000039976	Tbc1d16	-0.786049545	0.001492299
ENSMUSG00000069874	Irgm2	-1.290233	7.78809E-11	ENSMUSG00000024339	Tap2	-0.786059058	1.37348E-22
ENSMUSG00000047496	Rnf152	-1.293158463	0.002124966	ENSMUSG00000002835	Chaf1a	-0.786629375	3.08684E-39
ENSMUSG00000028943	Espn	-1.293301302	7.39487E-08	ENSMUSG00000038005	Hpf1	-0.786757455	8.221E-22
ENSMUSG00000074738	Fndc10	-1.293333541	1.06561E-08	ENSMUSG00000058835	Abi1	-0.786825005	3.6164E-122
ENSMUSG00000068762	Gstm6	-1.293499247	0.001579183	ENSMUSG00000035354	Uvrag	-0.786884145	3.48673E-59
ENSMUSG00000086075	Gm15728	-1.298112992	0.012778989	ENSMUSG00000053469	Tg	-0.78698731	0.012343599
ENSMUSG00000071112	Spx	-1.29831366	0.006663985	ENSMUSG00000036712	Cyld	-0.787602427	1.19256E-39
ENSMUSG00000042155	Klhl23	-1.299706591	1.32488E-20	ENSMUSG00000067878	Map7d3	-0.787724376	2.75258E-07
ENSMUSG00000009281	Rarres2	-1.30010515	4.23041E-06	ENSMUSG00000002257	Def6	-0.787787668	7.38036E-13
ENSMUSG00000053007	Creb5	-1.300563284	2.02952E-36	ENSMUSG00000056014	A430033K04Rik	-0.788075839	6.41479E-05
ENSMUSG00000051726	Kcnf1	-1.303313959	0.03477921	ENSMUSG00000023050	Map3k12	-0.788197744	3.83683E-18
ENSMUSG00000040033	Stat2	-1.304607202	4.74505E-12	ENSMUSG00000030704	Rab6a	-0.788235233	1.4606E-131
ENSMUSG00000029605	Oas1b	-1.306790101	5.88047E-11	ENSMUSG00000029061	Mmp23	-0.788452475	6.04801E-33
ENSMUSG00000038146	Notch3	-1.307005763	3.2505E-143	ENSMUSG00000029675	Eln	-0.788582003	2.82622E-82
ENSMUSG00000032374	Plod2	-1.307184436	0	ENSMUSG00000043993	2900052L18Rik	-0.789032495	0.006571077
ENSMUSG00000027221	Chst1	-1.30888975	1.24307E-17	ENSMUSG00000021072	Tmx1	-0.789837289	1.5753E-95
ENSMUSG00000022899	Slc15a2	-1.310153331	0.020352957	ENSMUSG00000030208	Emp1	-0.790373177	3.0265E-214
ENSMUSG00000035615	Frmpd1	-1.310216066	0.047372146	ENSMUSG00000045180	Shroom2	-0.790438123	1.447E-09
ENSMUSG00000036411	9530077C05Rik	-1.311381924	0.000401141	ENSMUSG00000040717	Il17rd	-0.79071419	1.44067E-16
ENSMUSG00000000142	Axin2	-1.311812861	3.42364E-05	ENSMUSG00000016493	Cd46	-0.790756112	0.010267157
ENSMUSG00000033730	Egr3	-1.314473511	3.00735E-13	ENSMUSG00000062397	Zfp706	-0.790831313	3.57379E-78
ENSMUSG00000036912	Piwil4	-1.316072069	0.00091103	ENSMUSG00000028496	Mllt3	-0.791278283	7.23817E-47
ENSMUSG00000056665	Them6	-1.318892877	1.75303E-10	ENSMUSG00000028044	Cks1b	-0.79149716	1.27182E-35
ENSMUSG00000037921	Ddx60	-1.320316243	1.57212E-11	ENSMUSG00000118239	Gm50107	-0.791814285	0.0350825
ENSMUSG00000070407	Hs3st3b1	-1.32151464	3.69277E-07	ENSMUSG00000020955	Ap4s1	-0.792176184	8.17641E-09
ENSMUSG00000036022	Fam122b	-1.322901565	1.22362E-21	ENSMUSG00000023206	Il15ra	-0.792187818	5.67476E-06
ENSMUSG00000039485	Tspyl4	-1.324806225	3.45531E-19	ENSMUSG00000034765	Dusp5	-0.792233881	1.2516E-28
ENSMUSG00000026610	Esrrg	-1.326894456	0.00048193	ENSMUSG00000001053	N4bp3	-0.79234647	0.013484699
ENSMUSG00000104342	Gm36401	-1.326964989	3.64926E-05	ENSMUSG00000030393	Zik1	-0.792854993	3.59001E-06
ENSMUSG00000021822	Plau	-1.32719871	6.9541E-194	ENSMUSG00000036206	Sh3bp4	-0.793017636	1.39334E-19
ENSMUSG00000021391	Cenpp	-1.327792763	6.31036E-08	ENSMUSG00000059013	Sh2d3c	-0.793267627	0.000752229
ENSMUSG00000032202	Rab27a	-1.32833755	0.03108364	ENSMUSG00000025969	Nrp2	-0.793824352	1.8015E-132
ENSMUSG00000051043	Gprc5c	-1.33158268	0.012799921	ENSMUSG00000034656	Cacna1a	-0.79426665	1.41444E-14
ENSMUSG00000031637	Lrp2bp	-1.332215454	0.010931529	ENSMUSG00000021589	Rhobtb3	-0.794295391	6.06591E-76
ENSMUSG00000039997	Ifi203	-1.337064506	1.24592E-07	ENSMUSG00000037855	Zfp365	-0.794304814	2.07214E-80
ENSMUSG00000029279	Brdt	-1.337322887	0.000174692	ENSMUSG00000028412	Slc44a1	-0.794341122	3.5587E-120
ENSMUSG00000040605	Bace2	-1.340632833	2.26911E-50	ENSMUSG00000043467	Zbtb37	-0.794343864	8.08424E-12
ENSMUSG00000108049	Gm44168	-1.340897219	0.023948216	ENSMUSG00000026600	Soat1	-0.79474681	1.0601E-101
ENSMUSG00000027932	Slc27a3	-1.341027556	2.93878E-20	ENSMUSG00000022489	Pde1b	-0.795056606	1.88401E-05
ENSMUSG00000078956	Gm14221	-1.343725296	0.00167592	ENSMUSG00000102813	Gm37795	-0.795151404	7.24264E-11
ENSMUSG00000050675	Gp1ba	-1.346876336	0.000472053	ENSMUSG00000027200	Sema6d	-0.795234684	2.09087E-32
ENSMUSG00000070469	Adamtsl3	-1.347606545	9.2858E-212	ENSMUSG00000061887	Ssbp3	-0.795365935	3.15422E-33
ENSMUSG00000072720	Myo18b	-1.348827956	1.1657E-16	ENSMUSG00000001870	Ltbp1	-0.795635973	4.18332E-71
ENSMUSG00000026764	Kif5c	-1.350815695	9.02165E-15	ENSMUSG00000029003	Mad2l2	-0.797330563	6.91163E-10

ENSMUSG00000105695	Gm43327	-1.352010001	0.007875318	ENSMUSG00000027966	Col11a1	-0.797402787	1.8841E-74
ENSMUSG00000029798	Herc6	-1.355490782	1.30778E-09	ENSMUSG00000016494	Cd34	-0.797540236	3.6968E-224
ENSMUSG00000044703	Phf11a	-1.357703865	0.00083641	ENSMUSG00000099760	Gm28800	-0.797833918	0.043429368
ENSMUSG00000005107	Slc2a9	-1.359870811	6.82586E-22	ENSMUSG00000021180	Rps6ka5	-0.797911951	0.000810843
ENSMUSG00000032009	Sesn3	-1.360100755	5.21285E-82	ENSMUSG00000032409	Atr	-0.798225053	1.00208E-27
ENSMUSG00000031066	Usp11	-1.363908365	7.98925E-10	ENSMUSG00000050965	Prkca	-0.798811118	1.17963E-78
ENSMUSG00000034614	Pik3ip1	-1.364967219	1.75111E-10	ENSMUSG00000020186	Csrp2	-0.798931594	2.29506E-19
ENSMUSG00000021208	Ifi27l2b	-1.366984069	0.000170258	ENSMUSG00000034218	Atm	-0.799779055	1.13225E-26
ENSMUSG00000033847	Pla2g4c	-1.369351626	0.041726842	ENSMUSG00000045799	Gm9800	-0.800449684	1.52985E-08
ENSMUSG00000026104	Stat1	-1.369875721	3.39633E-12	ENSMUSG00000035390	Brsk1	-0.800530325	1.22681E-08
ENSMUSG00000022464	Slc38a4	-1.371429073	1.4349E-23	ENSMUSG00000036745	Ttll7	-0.800781398	5.55797E-29
ENSMUSG00000074272	Ceacam1	-1.37268848	2.48999E-05	ENSMUSG00000029591	Ung	-0.801416454	0.00216106
ENSMUSG00000038984	Tspyl5	-1.372911443	1.00559E-05	ENSMUSG00000016128	Stard13	-0.802522461	9.54559E-41
ENSMUSG00000068877	Selenbp2	-1.379479048	0.005673244	ENSMUSG00000050332	Amer1	-0.802883388	1.35289E-14
ENSMUSG00000037235	Mxd4	-1.380645525	8.9218E-179	ENSMUSG00000074863	Platr25	-0.803410874	8.70082E-09
ENSMUSG00000018927	Ccl6	-1.382688509	3.02918E-11	ENSMUSG00000039450	Dcxr	-0.803716344	6.86404E-23
ENSMUSG00000114133	Gm20075	-1.387946626	1.30609E-12	ENSMUSG00000024063	Lbh	-0.804012829	9.2991E-174
ENSMUSG00000054675	Tmem119	-1.394592394	1.24499E-67	ENSMUSG00000032498	Mlh1	-0.804457928	3.00698E-11
ENSMUSG00000042109	Csdc2	-1.395289198	4.43093E-11	ENSMUSG00000044949	Ubtd2	-0.805084406	3.08397E-22
ENSMUSG00000015396	Cd83	-1.395726604	0.017763267	ENSMUSG00000015202	Cnksr3	-0.805113205	3.26658E-15
ENSMUSG00000025154	Arhgap19	-1.396293475	4.77114E-30	ENSMUSG00000027109	Sp3	-0.8051361	2.1276E-95
ENSMUSG00000096039	D830030K20Rik	-1.396353077	0.033048787	ENSMUSG00000046873	Mbtps2	-0.805324911	1.82374E-35
ENSMUSG00000045333	Zfp423	-1.396353754	3.00156E-08	ENSMUSG00000029265	Dr1	-0.805424739	3.45245E-51
ENSMUSG00000042444	Mindy2	-1.398666666	1.6617E-201	ENSMUSG00000025017	Pik3ap1	-0.805605624	0.003557739
ENSMUSG00000048126	Col6a3	-1.399478828	1.879E-125	ENSMUSG00000090877	Hspa1b	-0.80561175	6.83245E-22
ENSMUSG00000018983	E2f2	-1.400479935	9.36149E-08	ENSMUSG00000036661	Dennd3	-0.805713751	0.000903329
ENSMUSG00000049502	Dtx3l	-1.402786544	1.10846E-33	ENSMUSG00000006651	Aplp1	-0.806124443	3.45285E-28
ENSMUSG00000079671	2610203C22Rik	-1.403977924	3.80422E-05	ENSMUSG00000000275	Trim25	-0.806155497	1.04107E-28
ENSMUSG00000029561	Oasl2	-1.407936369	6.72786E-05	ENSMUSG00000054720	Lrrc8c	-0.806654069	3.92055E-96
ENSMUSG00000052143	Gm9869	-1.411876394	3.52334E-09	ENSMUSG00000027221	Chst1	-0.80686641	2.86473E-08
ENSMUSG00000020251	Glt8d2	-1.412792108	5.26833E-83	ENSMUSG00000078794	Dact3	-0.807458632	4.38902E-22
ENSMUSG00000086763	Plxna4os1	-1.412979992	0.036548314	ENSMUSG00000030008	Pradc1	-0.808092904	4.95572E-05
ENSMUSG00000074968	Ano3	-1.41409384	2.76509E-77	ENSMUSG00000033618	Map3k13	-0.809259007	2.99212E-06
ENSMUSG00000068245	Phf11d	-1.414595625	1.14148E-10	ENSMUSG00000001229	Dpp9	-0.809283174	1.5493E-75
ENSMUSG00000021340	Gpld1	-1.414979633	1.76779E-06	ENSMUSG00000033557	Fam20b	-0.80938477	7.39352E-70
ENSMUSG00000028399	Ptprd	-1.415135978	4.6605E-139	ENSMUSG00000028664	Ephb2	-0.810157431	3.29271E-16
ENSMUSG00000032380	Dapk2	-1.415610182	6.26547E-05	ENSMUSG00000032380	Dapk2	-0.810222035	0.014444724
ENSMUSG00000086233	Gm11816	-1.416648202	0.049816463	ENSMUSG00000021747	4930452B06Rik	-0.810241603	0.000249013
ENSMUSG00000007613	Tgfbr1	-1.418752974	7.7745E-234	ENSMUSG00000048582	Gja3	-0.810452213	4.16637E-07
ENSMUSG00000018648	Dusp14	-1.418803831	2.88225E-10	ENSMUSG00000032527	Pccb	-0.810531121	3.16963E-23
ENSMUSG00000106139	Gm30648	-1.419282704	0.018859322	ENSMUSG00000069520	Tmem19	-0.811115749	7.33485E-36
ENSMUSG00000031292	Cdkl5	-1.422230292	8.05475E-20	ENSMUSG00000028527	Ak4	-0.811481952	5.06504E-22
ENSMUSG00000078607	1810010H24Rik	-1.425152121	0.032846801	ENSMUSG00000031562	Dctd	-0.811566978	1.50754E-16
ENSMUSG00000022309	Angpt1	-1.43135532	1.04349E-06	ENSMUSG00000042284	Itga1	-0.812532883	3.7798E-59
ENSMUSG00000024338	Psmb8	-1.432511526	3.46564E-20	ENSMUSG00000032782	Cntrob	-0.812645516	1.10689E-08
ENSMUSG00000038663	Fsd2	-1.433258247	0.026688578	ENSMUSG00000046447	Camk2n1	-0.812788383	5.71577E-16
ENSMUSG00000042115	Klhdc8a	-1.43339446	4.71744E-54	ENSMUSG00000040841	Six5	-0.813068184	3.14329E-09
ENSMUSG00000031886	Ces2e	-1.436599241	1.06144E-24	ENSMUSG00000027132	Katnbl1	-0.813818663	1.08086E-54
ENSMUSG00000056145	AI504432	-1.444371998	0.014655508	ENSMUSG00000044033	Ccdc141	-0.81401933	5.35539E-06
ENSMUSG00000037962	Rflna	-1.445445503	0.016276874	ENSMUSG00000086119	Gm2415	-0.814573842	0.016108269
ENSMUSG00000025930	Msc	-1.448485254	0.000126815	ENSMUSG00000029576	Radil	-0.814721943	0.049523976
ENSMUSG00000049092	Gpr137c	-1.44944028	0.004388451	ENSMUSG00000089817	Gm7162	-0.816777788	0.031361628
ENSMUSG00000062563	Cys1	-1.453217852	6.95249E-06	ENSMUSG00000018678	Sp2	-0.818231611	4.23127E-14
ENSMUSG00000104713	Gbp6	-1.456060221	2.84903E-17	ENSMUSG00000050973	Gdpgp1	-0.81855986	7.62525E-07
ENSMUSG00000017446	C1qtnf1	-1.456418095	3.62711E-46	ENSMUSG00000031486	Adgra2	-0.818696146	2.7728E-101
ENSMUSG00000093629	Prox2os	-1.456818578	0.047739161	ENSMUSG00000085957	Syna	-0.818698167	0.019090181
ENSMUSG00000034295	Fhod3	-1.46175386	9.18929E-14	ENSMUSG00000027333	Smox	-0.819305535	3.32199E-22
ENSMUSG00000015968	Cacna1d	-1.463725196	0.026980318	ENSMUSG00000055866	Per2	-0.819370715	1.19761E-07
ENSMUSG00000048402	Gli2	-1.467529795	0.026242358	ENSMUSG00000035877	Zhx3	-0.820248904	1.75058E-34
ENSMUSG00000029862	Clcn1	-1.470870896	0.014807092	ENSMUSG00000097039	Pvt1	-0.820339115	1.89685E-12
ENSMUSG00000057337	Chst3	-1.471600278	0.007487028	ENSMUSG00000034723	Tmx4	-0.820727034	5.46622E-48
ENSMUSG00000027329	Spef1	-1.475497274	1.23846E-33	ENSMUSG00000000142	Axin2	-0.820844434	0.010242487
ENSMUSG00000041329	Atp1b2	-1.475628449	0.000462264	ENSMUSG00000074064	Mlycd	-0.821003172	1.74046E-14
ENSMUSG00000116165	Pdxp	-1.476290753	0.000547349	ENSMUSG00000026980	Ly75	-0.821071804	2.53622E-09
ENSMUSG00000043496	Tril	-1.479441761	2.35808E-10	ENSMUSG00000030064	Frmd4b	-0.821481208	9.84492E-14
ENSMUSG00000051098	Mblac2	-1.484213455	1.87031E-06	ENSMUSG00000009647	Mcu	-0.822980477	5.12762E-36
ENSMUSG00000045087	S1pr5	-1.484271415	0.027349703	ENSMUSG00000033589	Reep4	-0.823410146	4.15765E-27
ENSMUSG00000022831	Hcls1	-1.48744332	6.18176E-19	ENSMUSG00000037568	Vash2	-0.823557253	4.2952E-11
ENSMUSG00000022032	Scara5	-1.490764303	4.70473E-08	ENSMUSG00000027293	Ehd4	-0.82444811	1.22845E-70
ENSMUSG00000004748	Mtfp1	-1.492159897	0.001090764	ENSMUSG00000057329	Bcl2	-0.824549304	5.35494E-24
ENSMUSG00000033207	Mamdc2	-1.493214192	8.20515E-40	ENSMUSG00000015942	Gtf2ird2	-0.824893445	4.51033E-06
ENSMUSG00000029597	Sds	-1.494045627	0.043972809	ENSMUSG00000022587	Ly6e	-0.825471912	4.56978E-06
ENSMUSG00000029797	Sspo	-1.495137314	0.028844827	ENSMUSG00000021285	Ppp1r13b	-0.825590663	6.45657E-31
ENSMUSG00000074896	Ifit3	-1.495608152	1.05693E-06	ENSMUSG00000040848	Sft2d2	-0.826097881	2.01336E-60
ENSMUSG00000058656	Samd12	-1.497392563	1.13643E-08	ENSMUSG00000016995	Matn4	-0.82616938	0.003652096
ENSMUSG00000034738	Nostrin	-1.498640116	1.64303E-35	ENSMUSG00000021870	Slmap	-0.826335937	2.22554E-84
ENSMUSG00000037940	Inpp4b	-1.500926545	0.000238199	ENSMUSG00000024896	Minpp1	-0.826609918	1.55135E-36

ENSMUSG00000024043	Arhgap28	-1.502637759	6.39937E-43	ENSMUSG00000066842	Hmcn1	-0.827440294	0.002194379
ENSMUSG00000086825	Gm15675	-1.504629298	9.40011E-22	ENSMUSG00000037493	Cib2	-0.828440908	0.000444381
ENSMUSG00000079362	Gm43302	-1.504652696	3.04062E-10	ENSMUSG00000031907	Zfp90	-0.828888658	9.80257E-12
ENSMUSG00000029309	Sparcl1	-1.505667633	3.8617E-102	ENSMUSG00000044345	Marveld1	-0.828941173	1.29959E-87
ENSMUSG00000035493	Tgfbi	-1.506714476	3.4967E-117	ENSMUSG00000027698	Nceh1	-0.828946966	1.1179E-27
ENSMUSG00000094483	Purb	-1.507827892	0	ENSMUSG00000047238	Mageh1	-0.829188566	1.57864E-20
ENSMUSG00000032661	Oas3	-1.512938226	0.000335006	ENSMUSG00000026017	Carf	-0.829695767	1.10223E-05
ENSMUSG00000037705	Tecta	-1.51415146	0.000505741	ENSMUSG00000020728	Cep112	-0.829832114	0.001637776
ENSMUSG00000031880	Rrad	-1.516408604	7.7957E-09	ENSMUSG00000035547	Capn5	-0.830277966	3.01988E-14
ENSMUSG00000031805	Jak3	-1.51883373	7.67058E-31	ENSMUSG00000028024	Enpep	-0.830555807	4.91837E-08
ENSMUSG00000004655	Aqp1	-1.52489949	2.1131E-192	ENSMUSG00000022708	Zbtb20	-0.83086836	2.4334E-71
ENSMUSG00000030793	Pycard	-1.53255259	4.36408E-16	ENSMUSG00000006205	Htra1	-0.830985842	1.60811E-96
ENSMUSG00000035692	Isg15	-1.533874241	0.000160846	ENSMUSG00000109005	Gm45221	-0.83143723	0.008840442
ENSMUSG00000072437	Nanos1	-1.536957095	6.77308E-34	ENSMUSG00000038264	Sema7a	-0.831786442	4.28071E-28
ENSMUSG00000027171	Prrg4	-1.545417536	2.2792E-43	ENSMUSG00000069920	B3gnt9	-0.832222736	4.75105E-34
ENSMUSG00000034171	Faah	-1.548339816	0.009903929	ENSMUSG00000061751	Kalrn	-0.832383057	4.01769E-22
ENSMUSG00000029861	Fam131b	-1.548441792	9.57848E-06	ENSMUSG00000003526	Prodh	-0.832408047	0.000298426
ENSMUSG00000034459	Ifit1	-1.551281368	3.11373E-07	ENSMUSG00000028078	Dclk2	-0.832627278	5.81856E-09
ENSMUSG00000054072	Iigp1	-1.551445943	8.05814E-06	ENSMUSG00000029427	Zcchc8	-0.833002109	3.95349E-29
ENSMUSG00000091649	Phf11b	-1.555245037	0.024536191	ENSMUSG00000045658	Pid1	-0.833008891	9.81904E-10
ENSMUSG00000037379	Spon2	-1.559218875	1.0519E-189	ENSMUSG00000008206	Cers4	-0.833090252	1.75368E-24
ENSMUSG00000049866	Arl4c	-1.560575016	2.80649E-81	ENSMUSG00000058729	Lin9	-0.833248937	4.08558E-12
ENSMUSG00000086228	Ubap1l	-1.562935364	0.016010012	ENSMUSG00000020601	Trib2	-0.833548602	6.5319E-115
ENSMUSG00000050505	Pcdh20	-1.563255557	0.004716313	ENSMUSG00000031434	Morc4	-0.834399178	7.63116E-13
ENSMUSG00000042834	Nrep	-1.566854506	3.4137E-181	ENSMUSG00000039813	Tbc1d2	-0.835303831	8.30971E-09
ENSMUSG00000042874	D930007J09Rik	-1.573324217	0.030624619	ENSMUSG00000050777	Tmem37	-0.836178358	2.69369E-21
ENSMUSG00000015733	Capza2	-1.573844703	0	ENSMUSG00000020770	Unk	-0.836310655	9.86247E-24
ENSMUSG00000025612	Bach1	-1.581155963	8.8442E-203	ENSMUSG00000049044	Rapgef4	-0.836698421	1.17835E-07
ENSMUSG00000056031	9330154J02Rik	-1.582475734	0.013510099	ENSMUSG00000040121	Rep15	-0.837815981	0.040899547
ENSMUSG00000034282	Evpl	-1.584452179	3.54464E-05	ENSMUSG00000030978	Rrm1	-0.837896013	6.6331E-125
ENSMUSG00000027200	Sema6d	-1.585656492	4.8266E-173	ENSMUSG00000022604	Cep97	-0.838118479	2.84319E-06
ENSMUSG00000032744	Heyl	-1.591567662	8.49741E-17	ENSMUSG00000025555	Farp1	-0.838433834	1.2063E-116
ENSMUSG00000020340	Cyfip2	-1.59198206	9.43998E-05	ENSMUSG00000020453	Patz1	-0.838449036	1.27786E-15
ENSMUSG00000084842	Pabpc1l2b-ps	-1.596469077	0.003154458	ENSMUSG00000000171	Sdhd	-0.838520621	3.92301E-49
ENSMUSG00000024440	Pcdh12	-1.598049116	7.04737E-12	ENSMUSG00000095677	Dynlt1f	-0.839475102	2.88645E-14
ENSMUSG00000062257	Opcml	-1.603253587	3.35335E-05	ENSMUSG00000039234	Sec24d	-0.839476708	1.3227E-152
ENSMUSG00000078853	Igtp	-1.60334786	1.14833E-08	ENSMUSG00000109378	Gm49396	-0.839496785	1.84911E-06
ENSMUSG00000027489	Necab3	-1.604216021	0.037315456	ENSMUSG00000040296	Ddx58	-0.839535825	1.35082E-08
ENSMUSG00000024168	Tmem204	-1.60866249	8.20108E-40	ENSMUSG00000040428	Plekha4	-0.839943592	0.000111516
ENSMUSG00000062151	Unc13c	-1.610870037	8.09145E-11	ENSMUSG00000020649	Rrm2	-0.840294746	5.94976E-72
ENSMUSG00000039814	Xkr5	-1.612222432	3.92179E-06	ENSMUSG00000051444	Bbs12	-0.841119842	7.53665E-09
ENSMUSG00000039954	Stk32a	-1.614118693	0.033875637	ENSMUSG00000031107	Rbmx2	-0.841593972	2.99673E-09
ENSMUSG00000040447	Spns2	-1.621125924	6.61961E-23	ENSMUSG00000050931	Sgms2	-0.841798224	5.54904E-80
ENSMUSG00000040855	Reps2	-1.634283754	5.94599E-05	ENSMUSG00000021730	Hcn1	-0.842138932	2.13356E-08
ENSMUSG00000046157	Tmem229b	-1.64129496	3.48193E-10	ENSMUSG00000097439	Gm16754	-0.842147928	0.029619889
ENSMUSG00000032034	Kcnj5	-1.643027018	4.25435E-06	ENSMUSG00000030322	Mbd4	-0.842331692	1.59583E-06
ENSMUSG00000023034	Nr4a1	-1.646091461	7.42418E-87	ENSMUSG00000037339	Fam53a	-0.842794356	7.04463E-27
ENSMUSG00000025610	Map3k7cl	-1.655834471	7.93434E-25	ENSMUSG00000053846	Lipg	-0.842973053	7.2606E-08
ENSMUSG00000037166	Ppp1r14a	-1.659985773	2.47686E-12	ENSMUSG00000000031	H19	-0.843191787	2.01439E-05
ENSMUSG00000030711	Sult1a1	-1.661814502	0.0203269	ENSMUSG00000038508	Gdf15	-0.84333197	2.57217E-07
ENSMUSG00000073600	Prob1	-1.667017212	0.000602045	ENSMUSG00000033970	Rfc3	-0.843674871	2.87775E-15
ENSMUSG00000026051	Ecrg4	-1.674599238	1.02407E-83	ENSMUSG00000025809	Itgb1	-0.844265392	4.7618E-239
ENSMUSG00000029298	Gbp9	-1.678854081	7.84183E-08	ENSMUSG00000071537	Klrg2	-0.844947854	0.000404414
ENSMUSG00000049173	Myoz3	-1.679971539	0.015593006	ENSMUSG00000091971	Hspa1a	-0.844997015	4.82643E-11
ENSMUSG00000002324	Rec8	-1.682499481	0.03477921	ENSMUSG00000024620	Pdgfrb	-0.845787052	1.0483E-133
ENSMUSG00000104965	Gm43437	-1.683053549	0.000208273	ENSMUSG00000030309	Caprin2	-0.846307469	3.43976E-06
ENSMUSG00000037846	Rtkn2	-1.683691337	2.6747E-13	ENSMUSG00000047767	Atg16l2	-0.846569441	1.00135E-12
ENSMUSG00000062393	Dgkk	-1.683746239	0.020145469	ENSMUSG00000034295	Fhod3	-0.846723185	5.61109E-05
ENSMUSG00000022296	Baalc	-1.686380306	5.95387E-13	ENSMUSG00000032322	Pstpip1	-0.847155608	7.1825E-05
ENSMUSG00000052302	Tbc1d30	-1.688142285	0.003381134	ENSMUSG00000063445	Nmral1	-0.84770757	1.97321E-06
ENSMUSG00000052942	Glis3	-1.690030245	2.47506E-61	ENSMUSG00000027840	Wnt2b	-0.847775472	0.022712077
ENSMUSG00000021798	Ldb3	-1.692336026	2.21325E-07	ENSMUSG00000031827	Cotl1	-0.848013962	2.69638E-50
ENSMUSG00000118138	Gm50322	-1.693342702	4.27137E-11	ENSMUSG00000038963	Slco4a1	-0.848518742	2.26892E-14
ENSMUSG00000107017	Gm43196	-1.696595074	0.002052478	ENSMUSG00000019813	Cep57l1	-0.848960135	1.59319E-06
ENSMUSG00000009394	Syn2	-1.697308454	2.05589E-07	ENSMUSG00000051855	Mest	-0.849296694	3.5165E-06
ENSMUSG00000041515	Irf8	-1.702060312	4.36327E-25	ENSMUSG00000033671	Cep350	-0.849312626	1.47437E-36
ENSMUSG00000111685	Gm10686	-1.712721749	0.03746694	ENSMUSG00000056144	Trim34a	-0.849511728	1.30673E-09
ENSMUSG00000042359	Osbpl6	-1.717001369	3.60722E-18	ENSMUSG00000039145	Camk1d	-0.849763371	1.12036E-16
ENSMUSG00000031165	Was	-1.717011724	0.036072761	ENSMUSG00000021264	Yy1	-0.850415241	1.8174E-76
ENSMUSG00000015476	Prrt1	-1.717040714	0.017472352	ENSMUSG00000043252	Tmem64	-0.850441273	3.42925E-77
ENSMUSG00000046314	Stxbp6	-1.72894074	4.30264E-15	ENSMUSG00000025925	Terf1	-0.850730518	5.87863E-18
ENSMUSG00000105504	Gbp5	-1.73007837	7.81465E-13	ENSMUSG00000056486	Chn1	-0.8511671	2.95563E-40
ENSMUSG00000031698	Mylk3	-1.734046996	0.001955197	ENSMUSG00000024190	Dusp1	-0.852048701	5.12283E-41
ENSMUSG00000116597	Gm536	-1.74121062	0.001875349	ENSMUSG00000029544	Cabp1	-0.852129467	0.004384577
ENSMUSG00000030107	Usp18	-1.74180376	8.315E-08	ENSMUSG00000022462	Slc38a2	-0.853156361	2.5357E-204
ENSMUSG00000090698	Apold1	-1.753342211	5.7617E-31	ENSMUSG00000018899	Irf1	-0.853778137	1.5932E-17
ENSMUSG00000028782	Adgrb2	-1.755424544	1.21222E-06	ENSMUSG00000066643	Wdr35	-0.854623816	9.14833E-61

ENSMUSG00000035095	Fam167a	-1.757763009	7.81354E-11	ENSMUSG00000026004	Kansl1l	-0.854827516	1.64544E-20
ENSMUSG00000086524	Pabpc1l2a-ps	-1.769343928	0.002416962	ENSMUSG00000041879	Ipo9	-0.855379121	3.4365E-137
ENSMUSG00000004791	Pgf	-1.769468531	1.5726E-101	ENSMUSG00000030557	Mef2a	-0.855539595	4.8437E-180
ENSMUSG00000071658	Gng3	-1.773052229	0.009968534	ENSMUSG00000059824	Dbp	-0.855912752	0.00036051
ENSMUSG00000057948	Unc13d	-1.773170438	0.007839861	ENSMUSG00000085395	Gm13056	-0.85596036	0.005167632
ENSMUSG00000105096	Gbp10	-1.780521195	3.48521E-05	ENSMUSG00000047180	Neurl3	-0.85623532	6.32326E-11
ENSMUSG00000114926	Gm2379	-1.782377904	0.03393069	ENSMUSG00000038037	Socs1	-0.857054402	9.64426E-05
ENSMUSG00000039699	Batf2	-1.783762902	0.040640862	ENSMUSG00000024691	Fam111a	-0.857151536	8.15006E-66
ENSMUSG00000037337	Map4k1	-1.791172394	0.000663035	ENSMUSG00000017718	Afmid	-0.857516334	2.37937E-06
ENSMUSG00000090272	Mndal	-1.800622931	2.66911E-10	ENSMUSG00000066829	Zfp810	-0.859328303	3.95766E-08
ENSMUSG00000024525	Impa2	-1.804090253	7.79091E-54	ENSMUSG00000042156	Dzip1	-0.859733099	1.42163E-45
ENSMUSG00000022102	Dok2	-1.806460333	0.020092762	ENSMUSG00000027820	Mme	-0.859899779	2.56307E-10
ENSMUSG00000073295	Nudt11	-1.813439107	5.21888E-08	ENSMUSG00000042109	Csdc2	-0.860222509	1.79109E-05
ENSMUSG00000032690	Oas2	-1.831088976	7.71704E-21	ENSMUSG00000040749	Siah1b	-0.860236378	7.46122E-10
ENSMUSG00000073555	Gm4951	-1.831158233	2.77563E-21	ENSMUSG00000046062	Ppp1r15b	-0.860288511	7.8903E-108
ENSMUSG00000050666	Vstm4	-1.836000026	0.000843272	ENSMUSG00000035845	Alg12	-0.860314393	4.03505E-08
ENSMUSG00000104748	Gm43592	-1.836198495	0.044661844	ENSMUSG00000020169	Best3	-0.860980868	0.000697553
ENSMUSG00000011171	Vipr2	-1.83647286	0.019665795	ENSMUSG00000010048	Ifrd2	-0.86120596	1.12871E-15
ENSMUSG00000046634	Pkd1l1	-1.843292289	9.74569E-06	ENSMUSG00000024548	Setbp1	-0.861716943	7.32723E-17
ENSMUSG00000041827	Oasl1	-1.85516131	5.2209E-15	ENSMUSG00000003228	Grk5	-0.861832037	1.87419E-88
ENSMUSG00000048186	Bend7	-1.855420552	1.18328E-07	ENSMUSG00000022142	Nup155	-0.862315902	1.9847E-57
ENSMUSG00000027223	Mapk8ip1	-1.859350665	4.85734E-53	ENSMUSG00000002325	Irf9	-0.862369609	2.81382E-21
ENSMUSG00000023341	Mx2	-1.86645861	3.06032E-06	ENSMUSG00000037169	Mycn	-0.862417891	8.54046E-14
ENSMUSG00000030310	Slc6a1	-1.869502607	0.023676205	ENSMUSG00000026576	Atp1b1	-0.862878711	3.24402E-77
ENSMUSG00000014030	Pax5	-1.877887656	0.003070248	ENSMUSG00000021608	Lpcat1	-0.863052571	1.84453E-38
ENSMUSG00000024598	Fbn2	-1.881922906	1.31527E-13	ENSMUSG00000037646	Vps13b	-0.863081224	4.48227E-45
ENSMUSG00000108218	Olfr1372-ps1	-1.882952582	3.53563E-20	ENSMUSG00000033904	Ccp110	-0.86335394	3.79536E-36
ENSMUSG00000014164	Klhl3	-1.885947857	2.45717E-09	ENSMUSG00000020183	Cpm	-0.863825993	0.01014997
ENSMUSG00000112932	Gm48308	-1.895058075	0.009459576	ENSMUSG00000106928	Gm43860	-0.864370384	0.002227276
ENSMUSG00000090942	F830016B08Rik	-1.898294956	4.76126E-16	ENSMUSG00000040658	Dnph1	-0.864538089	4.5494E-09
ENSMUSG00000049799	Lrrc19	-1.907143499	0.009410646	ENSMUSG00000040339	Fam102b	-0.866940724	7.4059E-67
ENSMUSG00000041757	Plekha6	-1.910083508	1.04206E-60	ENSMUSG00000073787	Gm10575	-0.867197893	1.28813E-06
ENSMUSG00000033082	Clec1a	-1.913946834	0.00561161	ENSMUSG00000042082	Arsb	-0.867206657	5.1886E-62
ENSMUSG00000074973	Gm11382	-1.915792477	0.010905306	ENSMUSG00000069114	Zbtb10	-0.867623326	1.04706E-41
ENSMUSG00000052572	Dlg2	-1.920271369	2.78627E-23	ENSMUSG00000029436	Mmp17	-0.868776923	0.000753094
ENSMUSG00000033355	Rtp4	-1.929980605	1.34862E-13	ENSMUSG00000099481	Xndc1	-0.869664469	3.55345E-09
ENSMUSG00000070348	Ccnd1	-1.944939588	0	ENSMUSG00000021115	Vrk1	-0.870151573	5.44844E-27
ENSMUSG00000042078	Svop	-1.955045373	6.95623E-07	ENSMUSG00000006575	Rundc3a	-0.870347606	0.011470993
ENSMUSG00000026090	2010300C02Rik	-1.965908574	0.019774337	ENSMUSG00000110185	Igip	-0.870399104	4.24211E-23
ENSMUSG00000022015	Tnfsf11	-1.972557832	1.65235E-12	ENSMUSG00000043300	B3galnt1	-0.870702014	4.29058E-16
ENSMUSG00000038224	Serpinf2	-1.976007134	0.026139146	ENSMUSG00000028755	Cda	-0.87082316	9.18391E-05
ENSMUSG00000035407	Kank4	-1.979274241	0.02180262	ENSMUSG00000020658	Efr3b	-0.870844967	7.14777E-37
ENSMUSG00000062488	Ifit3b	-1.986138079	7.81114E-10	ENSMUSG00000026208	Des	-0.871587135	0.000625048
ENSMUSG00000028211	Trp53inp1	-1.987532133	0	ENSMUSG00000027896	Slc16a4	-0.872260184	6.89385E-06
ENSMUSG00000100798	Gm19589	-1.988897811	3.74307E-05	ENSMUSG00000042256	Ptchd4	-0.872947395	4.77025E-07
ENSMUSG00000025475	Adgra1	-1.98963962	3.86456E-06	ENSMUSG00000055053	Nfic	-0.874120374	1.82933E-83
ENSMUSG00000034855	Cxcl10	-1.990121155	4.47183E-05	ENSMUSG00000046111	Cep295	-0.874297796	2.51219E-37
ENSMUSG00000031712	Il15	-1.992228039	2.46641E-14	ENSMUSG00000082127	Gm13577	-0.875156195	0.034627208
ENSMUSG00000046005	D830044D21Rik	-2.011313211	0.008428486	ENSMUSG00000023104	Rfc2	-0.875215924	1.29296E-27
ENSMUSG00000040653	Ppp1r14c	-2.013287024	0.000120555	ENSMUSG00000075528	Aarsd1	-0.875367296	3.79896E-21
ENSMUSG00000040253	Gbp7	-2.033083542	2.04338E-17	ENSMUSG00000022508	Bcl6	-0.877599267	5.65843E-15
ENSMUSG00000051506	Wdfy4	-2.037455631	0.037575778	ENSMUSG00000036022	Fam122b	-0.878092057	3.47503E-11
ENSMUSG00000113909	Gm36377	-2.039878154	0.00052698	ENSMUSG00000044433	Camsap3	-0.879045167	4.97928E-05
ENSMUSG00000075070	4932412D23Rik	-2.040437988	0.020848694	ENSMUSG00000024747	Aldh1a7	-0.879458203	0.000241401
ENSMUSG00000070644	Etnk2	-2.05362149	1.21196E-05	ENSMUSG00000055884	Fancm	-0.880154348	5.21855E-24
ENSMUSG00000080268	Brms1	-2.057376818	0.000149333	ENSMUSG00000021234	Fam161b	-0.880404069	0.017525401
ENSMUSG00000048485	Zbtb8b	-2.057457035	1.00293E-06	ENSMUSG00000006221	Hspb7	-0.880711941	3.83914E-28
ENSMUSG00000001029	Icam2	-2.064574775	1.09507E-14	ENSMUSG00000039989	Cbx4	-0.880834442	1.94456E-19
ENSMUSG00000107045	Gm43636	-2.077207407	0.042138278	ENSMUSG00000031478	Nek3	-0.882723372	6.20837E-08
ENSMUSG00000000049	Apoh	-2.079585122	2.96164E-05	ENSMUSG00000049791	Fzd4	-0.882736081	4.15076E-52
ENSMUSG00000109459	Gm39526	-2.079715204	0.024212158	ENSMUSG00000089762	Ier5l	-0.882954469	7.31124E-28
ENSMUSG00000026126	Ptpn18	-2.088932685	0.001502999	ENSMUSG00000046841	Ckap4	-0.884008415	7.057E-210
ENSMUSG00000057346	Apol9a	-2.100989503	2.47613E-17	ENSMUSG00000054733	Msra	-0.88498645	6.55146E-07
ENSMUSG00000033578	Tmem35a	-2.107555132	1.6126E-17	ENSMUSG00000083907	Plk-ps1	-0.885047194	0.013808003
ENSMUSG00000046480	Scn4b	-2.107952287	0.003985433	ENSMUSG00000050222	Il17d	-0.885222333	0.002619084
ENSMUSG00000036295	Lrrn3	-2.12005762	3.6184E-10	ENSMUSG00000006154	Eps8l1	-0.885678649	0.012623301
ENSMUSG00000032648	Pygm	-2.120273037	4.97752E-21	ENSMUSG00000024521	Pmaip1	-0.886243264	8.12195E-22
ENSMUSG00000029923	Rab19	-2.135677696	0.023811138	ENSMUSG00000117183	Gm20008	-0.88676429	1.63669E-06
ENSMUSG00000044927	H1f10	-2.135776683	1.19159E-48	ENSMUSG00000026080	Chst10	-0.887193648	0.000366236
ENSMUSG00000006235	Epor	-2.138422629	9.48665E-06	ENSMUSG00000028675	Pnrc2	-0.887522654	9.08439E-89
ENSMUSG00000036655	Colec11	-2.151968896	7.45073E-15	ENSMUSG00000034422	Parp14	-0.888832317	4.51245E-06
ENSMUSG00000096727	Psmb9	-2.155098296	2.34304E-11	ENSMUSG00000042379	Esm1	-0.889333071	2.25618E-87
ENSMUSG00000057880	Abat	-2.156167515	9.9232E-140	ENSMUSG00000039599	Fam149b	-0.889368815	5.47457E-32
ENSMUSG00000020638	Cmpk2	-2.156534977	3.73455E-07	ENSMUSG00000103088	Pcdhgb6	-0.889418018	0.039189563
ENSMUSG00000005677	Nr1i3	-2.156839254	0.023191951	ENSMUSG00000026825	Dnm1	-0.891354278	2.95821E-16
ENSMUSG00000006930	Hap1	-2.175090497	2.93956E-22	ENSMUSG00000073599	Ecscr	-0.891422081	1.74267E-31
ENSMUSG00000028555	Ttc39a	-2.190770785	4.63563E-18	ENSMUSG00000075318	Scn2a	-0.891621702	0.000465853

ENSMUSG00000051457	Spn	-2.192228895	1.99778E-20
ENSMUSG00000029838	Ptn	-2.197019129	5.0668E-233
ENSMUSG00000037849	Ifi206	-2.199779881	0.002363503
ENSMUSG00000047842	Diras2	-2.200376114	2.28097E-20
ENSMUSG00000108176	Gm43965	-2.201264334	0.005180288
ENSMUSG00000035456	Prdm8	-2.209495708	0.010236437
ENSMUSG00000025701	Alox5	-2.226164095	9.14415E-11
ENSMUSG00000079505	Gm11131	-2.236003433	0.002307847
ENSMUSG00000063388	BC023105	-2.243403624	7.26518E-16
ENSMUSG00000047638	Nr1h4	-2.272104594	3.34397E-43
ENSMUSG00000061068	Mcpt4	-2.276031767	0.03260912
ENSMUSG00000048764	Tmprss11f	-2.286961687	0.03336546
ENSMUSG00000035189	Ano4	-2.29071017	3.17634E-05
ENSMUSG00000058589	Anks1b	-2.296002625	2.86106E-22
ENSMUSG00000039899	Fgl2	-2.29811284	2.01951E-94
ENSMUSG00000079652	Fam71f2	-2.318047583	0.010455032
ENSMUSG00000107314	Gm20488	-2.324967481	2.10498E-08
ENSMUSG00000039217	Il18	-2.329507724	7.86726E-05
ENSMUSG00000109134	Gm45076	-2.331179433	0.015078536
ENSMUSG00000090222	Ifi203-ps	-2.34934547	1.58298E-05
ENSMUSG00000021703	Serinc5	-2.352802061	2.6049E-190
ENSMUSG00000068246	Apol9b	-2.358871314	2.02967E-57
ENSMUSG00000078202	Nrarp	-2.371603904	0.000173218
ENSMUSG00000078234	Klhdc7a	-2.380483449	9.10534E-11
ENSMUSG00000103469	Gm9910	-2.381107978	0.022565414
ENSMUSG00000029120	Ppp2r2c	-2.383773857	0.000178025
ENSMUSG00000026564	Dusp27	-2.389517354	1.47993E-22
ENSMUSG00000021448	Shc3	-2.394035665	2.41206E-08
ENSMUSG00000045534	Kcna5	-2.396878774	6.05531E-10
ENSMUSG00000051359	Ncald	-2.397050595	4.71416E-73
ENSMUSG00000020151	Ptprr	-2.41346163	0.001256591
ENSMUSG00000090257	Gm4524	-2.41886044	0.000308812
ENSMUSG00000022821	Hgd	-2.421396093	0.019913746
ENSMUSG00000089739	Gm20431	-2.424737986	0.024234058
ENSMUSG00000002100	Mybpc3	-2.439785072	0.033010994
ENSMUSG00000047146	Tet1	-2.440528859	3.68843E-15
ENSMUSG00000020641	Rsad2	-2.469614998	1.26456E-13
ENSMUSG00000057137	Tmem140	-2.479890928	4.2964E-98
ENSMUSG00000034990	Otoa	-2.486676258	4.0442E-06
ENSMUSG00000079363	Gbp4	-2.49128285	2.72007E-12
ENSMUSG00000031778	Cx3cl1	-2.492835871	0
ENSMUSG00000059645	Gm7361	-2.502879915	0.032784149
ENSMUSG00000109056	A630009H07Rik	-2.506978992	0.00752443
ENSMUSG00000038540	Tmc3	-2.533803294	2.49065E-07
ENSMUSG00000030054	Gp9	-2.538101353	0.009474252
ENSMUSG00000030674	Qprt	-2.564494101	0.046373237
ENSMUSG00000111857	1190001M18Rik	-2.579355589	3.90885E-06
ENSMUSG00000020798	Spns3	-2.585467021	0.012755716
ENSMUSG00000047844	Bex4	-2.618451961	0.005642844
ENSMUSG00000025955	Akr1cl	-2.622822983	0.014624939
ENSMUSG00000019987	Arg1	-2.628522968	0.002589016
ENSMUSG00000045287	Rtn4rl1	-2.640150788	8.92933E-19
ENSMUSG00000057606	Colq	-2.693807546	1.18224E-07
ENSMUSG00000078922	Tgtp1	-2.699980105	1.47983E-27
ENSMUSG00000057074	Ces1g	-2.703214067	0.033281659
ENSMUSG00000039419	Cntnap2	-2.710967669	0.00783345
ENSMUSG00000082292	Gm12250	-2.738785177	1.64662E-09
ENSMUSG00000000386	Mx1	-2.742966872	2.37901E-16
ENSMUSG00000034818	Celf5	-2.77141698	8.48549E-21
ENSMUSG00000053310	Nrgn	-2.774335428	0.004489479
ENSMUSG00000025020	Slit1	-2.792140008	0.002138028
ENSMUSG00000078921	Tgtp2	-2.796118348	1.26011E-18
ENSMUSG00000064294	Aox3	-2.819640569	0.019938463
ENSMUSG00000058396	Gpr182	-2.856770879	3.54951E-46
ENSMUSG00000090394	4930523C07Rik	-2.856918649	6.3956E-118
ENSMUSG00000055489	Ano5	-2.879008589	0.009616712
ENSMUSG00000055882	Abhd16b	-2.886671219	0.013539243
ENSMUSG00000090125	Pou3f1	-2.88815253	0.000431658
ENSMUSG00000000792	Slc5a5	-2.972873869	0.009875637
ENSMUSG00000020787	P2rx1	-2.97627338	5.4455E-05
ENSMUSG00000068859	Sp9	-2.977475821	2.49065E-07
ENSMUSG00000040310	Alx4	-2.99191072	2.24304E-10
ENSMUSG00000036596	Cpz	-3.023336143	1.53281E-10
ENSMUSG00000074517	Gm10710	-3.052964061	0.040725634
ENSMUSG00000028011	Tdo2	-3.084776941	0.001422116
ENSMUSG00000024155	Meiob	-3.084802935	0.000116345
ENSMUSG00000114515	Aldoa	-3.103627068	0.033684621
ENSMUSG00000021302	Ggps1	-0.891912718	2.44563E-78
ENSMUSG00000001507	Itga3	-0.892948307	3.16204E-38
ENSMUSG00000097233	Gm17552	-0.893543729	0.04070592
ENSMUSG00000075122	Cd80	-0.89379653	6.14712E-42
ENSMUSG00000037712	Fermt2	-0.894294512	6.2711E-162
ENSMUSG00000020340	Cyfip2	-0.894319262	0.013566333
ENSMUSG00000022003	Slc25a30	-0.894344415	2.17353E-39
ENSMUSG00000050538	B230217C12Rik	-0.894430386	0.011674414
ENSMUSG00000073792	Alg6	-0.894434538	4.63479E-12
ENSMUSG00000029468	P2rx7	-0.894588314	5.61257E-28
ENSMUSG00000026932	Nacc2	-0.895548203	2.83147E-57
ENSMUSG00000035847	Ids	-0.895558274	2.76316E-65
ENSMUSG00000043259	Fam13c	-0.895757064	4.02797E-19
ENSMUSG00000055013	Agap1	-0.895998551	1.52784E-20
ENSMUSG00000035051	Dhx57	-0.89614682	1.7537E-27
ENSMUSG00000028479	Gne	-0.897462633	1.77453E-61
ENSMUSG00000064128	Cenpj	-0.897585261	2.46392E-20
ENSMUSG00000027210	Meis2	-0.897695658	3.28234E-42
ENSMUSG00000015468	Notch4	-0.898907347	2.21841E-12
ENSMUSG00000097325	Gm16897	-0.899322249	0.00064209
ENSMUSG00000040274	Cdk6	-0.899880465	8.0814E-131
ENSMUSG00000027313	Chac1	-0.899882093	7.53943E-10
ENSMUSG00000030222	Rerg	-0.900526854	2.08811E-45
ENSMUSG00000003062	Stard3nl	-0.900716927	1.47082E-41
ENSMUSG00000038816	Ctnnal1	-0.901103524	1.03728E-26
ENSMUSG00000044551	9930012K11Rik	-0.901180408	3.90332E-08
ENSMUSG00000008136	Fhl2	-0.902037089	1.5022E-108
ENSMUSG00000055917	Zfp277	-0.902647257	4.26533E-17
ENSMUSG00000040653	Ppp1r14c	-0.902835652	0.038949039
ENSMUSG00000035640	Cbarp	-0.903034916	5.73762E-11
ENSMUSG00000021597	Slf1	-0.90312677	3.32687E-19
ENSMUSG00000027669	Gnb4	-0.90314976	4.46219E-56
ENSMUSG00000029019	Nppb	-0.903292132	0.001110744
ENSMUSG00000022483	Col2a1	-0.904041945	0.002287552
ENSMUSG00000039601	Rcan2	-0.904100986	6.83713E-41
ENSMUSG00000113585	Gm19605	-0.904157419	0.031605232
ENSMUSG00000048779	P2ry6	-0.904327483	0.002017769
ENSMUSG00000052446	Zfp961	-0.90446458	1.25677E-05
ENSMUSG00000043631	Ecm2	-0.904803964	3.83525E-08
ENSMUSG00000114540	Gm6421	-0.905103886	0.047337085
ENSMUSG00000021775	Nr1d2	-0.905416252	4.67914E-86
ENSMUSG00000051615	Rap2a	-0.905878032	6.5679E-115
ENSMUSG00000041552	Ptchd1	-0.906059038	0.000219873
ENSMUSG00000051375	Pcdh1	-0.906237852	3.45083E-49
ENSMUSG00000034842	Art3	-0.906293367	1.45227E-07
ENSMUSG00000027827	Kcnab1	-0.90719509	6.56628E-58
ENSMUSG00000034898	Filip1	-0.907615933	2.84981E-06
ENSMUSG00000021432	Slc35b3	-0.907802236	2.16501E-36
ENSMUSG00000026048	Ercc5	-0.908162901	2.21169E-56
ENSMUSG00000040620	Dhx33	-0.9087951	1.7979E-107
ENSMUSG00000027750	Postn	-0.90936201	1.6393E-272
ENSMUSG00000069910	Spdl1	-0.910239842	1.28127E-25
ENSMUSG00000021806	Nid2	-0.910619267	8.616E-124
ENSMUSG00000036943	Rab8b	-0.910774346	5.45828E-70
ENSMUSG00000024077	Strn	-0.910783438	1.86554E-78
ENSMUSG00000030788	Rnf141	-0.911595641	1.64135E-81
ENSMUSG00000020258	Glyctk	-0.91235961	0.000562136
ENSMUSG00000010803	Gabra1	-0.912805964	0.000874559
ENSMUSG00000047749	Zc3hav1l	-0.912997806	3.05635E-15
ENSMUSG00000051950	B3glct	-0.91323238	4.82695E-56
ENSMUSG00000058900	Rsl1	-0.913865167	1.68489E-08
ENSMUSG00000067889	Sptbn2	-0.913949919	3.27305E-09
ENSMUSG00000021810	Ecd	-0.915324897	5.26431E-32
ENSMUSG00000005682	Pan2	-0.915753766	1.59195E-29
ENSMUSG00000047888	Tnrc6b	-0.916262881	9.74653E-46
ENSMUSG00000037752	Xkr8	-0.916524401	3.00381E-20
ENSMUSG00000045752	Tssc4	-0.916650406	2.54982E-58
ENSMUSG00000036570	Fxyd1	-0.916687341	2.65999E-05
ENSMUSG00000038618	Rassf7	-0.916725554	0.018490482
ENSMUSG00000030380	Mzf1	-0.916738633	0.016972035
ENSMUSG00000112397	Gm10824	-0.917447209	0.006946664
ENSMUSG00000039043	Arpin	-0.917642459	1.33275E-40
ENSMUSG00000089857	Zfp882	-0.917762314	0.00040298
ENSMUSG00000025997	Ikzf2	-0.917880735	1.46333E-06
ENSMUSG00000025937	Lactb2	-0.918572315	6.23361E-61
ENSMUSG00000021388	Aspn	-0.919160686	2.88386E-17
ENSMUSG00000037035	Inhbb	-0.919352591	2.42596E-36

ENSMUSG00000026831	1700007K13Rik	-3.118547566	6.26656E-32
ENSMUSG00000086918	4930429F24Rik	-3.135981374	0.002822144
ENSMUSG00000020884	Asgr1	-3.146285171	0.003359551
ENSMUSG00000050014	Apol10b	-3.178354492	5.50581E-05
ENSMUSG00000075010	AW112010	-3.250363569	0.002623568
ENSMUSG00000027168	Pax6	-3.267270258	0.01369375
ENSMUSG00000068606	Gm4841	-3.300973217	1.1338E-18
ENSMUSG00000026468	Lhx4	-3.320334527	0.030046348
ENSMUSG00000024036	Slc37a1	-3.330732248	3.75212E-05
ENSMUSG00000022868	Ahsg	-3.331980302	0.003644299
ENSMUSG00000113780	Gm33195	-3.369182611	0.01680421
ENSMUSG00000047040	Prr15l	-3.374612715	0.025198867
ENSMUSG00000079355	Ackr4	-3.392309361	6.2198E-146
ENSMUSG00000116993	Gm32461	-3.42072251	0.031916552
ENSMUSG00000039438	Ttc36	-3.42586963	0.015461328
ENSMUSG00000055333	Fat2	-3.438217032	0.002463371
ENSMUSG00000093634	Gm10860	-3.439517998	0.011836546
ENSMUSG00000039304	Tnfsf10	-3.446153612	1.20119E-18
ENSMUSG00000030344	Akap3	-3.450255886	0.020547013
ENSMUSG00000117029	Gm36201	-3.46025229	0.017339923
ENSMUSG00000097365	C030034L19Rik	-3.465622547	0.018910799
ENSMUSG00000064225	Paqr9	-3.594419906	0.010687799
ENSMUSG00000037798	Mat1a	-3.636351641	0.001300695
ENSMUSG00000050440	Hamp	-3.70582386	0.03272624
ENSMUSG00000074628	Tldc2	-3.772270007	0.003988607
ENSMUSG00000109931	Gm39929	-3.809467424	0.007660752
ENSMUSG00000027793	Ccna1	-3.88234024	0.034801887
ENSMUSG00000000673	Haao	-3.906779734	9.46503E-05
ENSMUSG00000046999	1110032F04Rik	-3.950328835	3.97651E-12
ENSMUSG00000024990	Rbp4	-3.996118589	0.002438196
ENSMUSG00000073430	Gm10505	-4.038791436	0.001684863
ENSMUSG00000051716	Apon	-4.105466259	7.55487E-05
ENSMUSG00000117966	1700061A03Rik	-4.126941323	0.013264252
ENSMUSG00000034872	Gipc3	-4.134644487	0.000889271
ENSMUSG00000030895	Hpx	-4.149132857	0.00096947
ENSMUSG00000026715	Serpinc1	-4.186993251	0.033313385
ENSMUSG00000035283	Adrb1	-4.246475843	0.048362995
ENSMUSG00000018459	Slc13a3	-4.417077513	8.15881E-05
ENSMUSG00000006522	Itih3	-4.621330857	1.54938E-07
ENSMUSG00000028528	Dnajc6	-4.841222787	0.001179586
ENSMUSG00000010064	Slc38a3	-4.933279336	3.35759E-08
ENSMUSG00000005681	Apoa2	-4.945634219	0.000522013
ENSMUSG00000086534	Gm16758	-5.197537786	0.049259546
ENSMUSG00000074651	Mcidas	-5.261786538	0.042117859
ENSMUSG00000087149	Itih5l-ps	-5.262614809	0.042064633
ENSMUSG00000040525	Cblc	-5.322080875	0.037590995
ENSMUSG00000047394	Odf3b	-5.322613728	0.03666777
ENSMUSG00000050201	Otop2	-5.324165036	0.036700415
ENSMUSG00000027875	Hmgcs2	-5.354587853	6.15888E-05
ENSMUSG00000106089	Gm9419	-5.378138427	0.032082832
ENSMUSG00000085918	Gm13032	-5.379704611	0.031503179
ENSMUSG00000092216	Gm19345	-5.380361527	0.030438378
ENSMUSG00000105834	4930592C13Rik	-5.382469713	0.031997988
ENSMUSG00000022269	Marchf11	-5.382541082	0.030614084
ENSMUSG00000082776	Gm7061	-5.438785842	0.026509132
ENSMUSG00000108686	Gm45080	-5.442354179	0.029457929
ENSMUSG00000114603	Gm17938	-5.489180846	0.023494026
ENSMUSG00000112481	Gm29684	-5.491980194	0.021998085
ENSMUSG00000026686	Lmx1a	-5.495410214	0.022976772
ENSMUSG00000057455	Rit2	-5.496795521	0.02519354
ENSMUSG00000073940	Hbb-bt	-5.541486972	0.045768002
ENSMUSG00000036110	Slc17a2	-5.542875752	0.045632048
ENSMUSG00000038583	Pln	-5.547009004	0.048515874
ENSMUSG00000022156	Gzme	-5.551682984	0.048064452
ENSMUSG00000043832	Clec4a3	-5.591800771	0.04089266
ENSMUSG00000095956	1700036A12Rik	-5.595295723	0.021569485
ENSMUSG00000030972	Acsm5	-5.599169306	0.044452088
ENSMUSG00000040367	Lrrd1	-5.638781257	0.037092645
ENSMUSG00000090063	Dlx6os1	-5.639400981	0.036819528
ENSMUSG00000035836	Ugt2b1	-5.643660251	0.036500634
ENSMUSG00000032739	Pram1	-5.68606468	5.70769E-05
ENSMUSG00000042474	Fcmr	-5.687325013	0.013557235
ENSMUSG00000056648	Hoxb8	-5.692119718	0.011736039
ENSMUSG00000054757	Akr1c20	-5.731778137	0.02993258
ENSMUSG00000001943	Vsig2	-5.735854155	0.010370661
ENSMUSG00000030617	Ccdc83	-5.739668202	0.013027342
ENSMUSG00000030834	Abcc6	-5.740117389	0.012256525
ENSMUSG00000046404	Yod1	-0.919696967	4.26409E-14
ENSMUSG00000024054	Smchd1	-0.920098967	7.68084E-63
ENSMUSG00000029363	Rfc5	-0.920624537	2.10167E-24
ENSMUSG00000025930	Msc	-0.921310717	0.047114985
ENSMUSG00000034795	Ccdc122	-0.921990873	1.62288E-10
ENSMUSG00000052584	Serp2	-0.922006984	0.034697009
ENSMUSG00000066894	Vsig10	-0.922494752	3.5744E-12
ENSMUSG00000026896	Ifih1	-0.923511108	1.81401E-06
ENSMUSG00000026839	Upp2	-0.923518889	0.020423218
ENSMUSG00000043795	Prr33	-0.924353466	0.003371888
ENSMUSG00000027217	Tspan18	-0.924454155	1.40469E-06
ENSMUSG00000023367	Tmem176a	-0.924796078	4.29249E-73
ENSMUSG00000051339	2900026A02Rik	-0.924815809	3.54848E-88
ENSMUSG00000078349	AW011738	-0.924850133	6.26049E-05
ENSMUSG00000041859	Mcm3	-0.924867097	1.09006E-79
ENSMUSG00000104453	Gm37829	-0.925138599	0.014974999
ENSMUSG00000033083	Tbc1d4	-0.925826697	1.64749E-28
ENSMUSG00000032534	Cep63	-0.926147835	2.12298E-32
ENSMUSG00000070867	Trabd2b	-0.926606617	1.3854E-13
ENSMUSG00000026874	Hc	-0.927395541	0.013687045
ENSMUSG00000001018	Snapin	-0.927536249	6.91001E-79
ENSMUSG00000037138	Aff3	-0.927938984	9.79314E-17
ENSMUSG00000035572	Dcaf10	-0.928067665	8.70455E-88
ENSMUSG00000038718	Pbx3	-0.928792879	4.93973E-38
ENSMUSG00000022761	Lztr1	-0.928907496	2.2246E-109
ENSMUSG00000074738	Fndc10	-0.928995428	2.34172E-05
ENSMUSG00000087370	Tmem170b	-0.929003152	4.13109E-27
ENSMUSG00000030844	Rgs10	-0.929020616	6.43914E-16
ENSMUSG00000036109	Mbnl3	-0.929482382	3.28437E-11
ENSMUSG00000032555	Topbp1	-0.929648766	3.13217E-84
ENSMUSG00000038022	Mindy4	-0.929783005	2.60101E-11
ENSMUSG00000046714	Foxc2	-0.930021682	1.02731E-56
ENSMUSG00000109539	Gm44667	-0.930157139	0.00011281
ENSMUSG00000051977	Prdm9	-0.930828634	0.016891281
ENSMUSG00000032661	Oas3	-0.930972836	0.019682625
ENSMUSG00000020070	Rufy2	-0.933599269	2.2249E-33
ENSMUSG00000024594	Prrc1	-0.934241559	1.5017E-122
ENSMUSG00000024727	Trpm6	-0.934515799	5.55008E-07
ENSMUSG00000050379	Septin6	-0.936108731	2.89836E-22
ENSMUSG00000026380	Tfcp2l1	-0.936415959	3.45434E-05
ENSMUSG00000097048	1600020E01Rik	-0.936444196	3.07076E-06
ENSMUSG00000029651	Mtus2	-0.937381444	1.09927E-30
ENSMUSG00000093904	Tomm20	-0.937590968	6.9686E-158
ENSMUSG00000039789	Zfp597	-0.938446183	2.44354E-24
ENSMUSG00000037946	Fgd3	-0.939335574	1.60915E-72
ENSMUSG00000027361	Gabpb1	-0.939666216	7.6367E-56
ENSMUSG00000035891	Cerk	-0.940131451	9.30441E-54
ENSMUSG00000047867	Gimap6	-0.940590865	9.09016E-06
ENSMUSG00000069808	Fam57a	-0.941161004	7.08489E-35
ENSMUSG00000033031	Cip2a	-0.941584011	1.36741E-70
ENSMUSG00000051048	P4ha3	-0.942200216	4.0183E-20
ENSMUSG00000031785	Adgrg1	-0.942532934	0.006319199
ENSMUSG00000034738	Nostrin	-0.94257858	6.64207E-19
ENSMUSG00000059791	Nrm	-0.943551073	5.5292E-10
ENSMUSG00000031292	Cdkl5	-0.944720112	3.18958E-08
ENSMUSG00000021340	Gpld1	-0.945843202	0.00056006
ENSMUSG00000037674	Rfx7	-0.946752965	7.69024E-76
ENSMUSG00000055148	Klf2	-0.946789654	5.79351E-15
ENSMUSG00000023391	Dlx2	-0.946861281	0.001289476
ENSMUSG00000039137	Whrn	-0.946994927	0.00184639
ENSMUSG00000036975	Tmem177	-0.947565655	4.94823E-09
ENSMUSG00000061411	Nol4l	-0.94794935	5.37415E-11
ENSMUSG00000094595	Fsbp	-0.948383595	9.43707E-05
ENSMUSG00000031381	Piga	-0.949502543	1.75391E-19
ENSMUSG00000033900	Map9	-0.949738767	2.44219E-23
ENSMUSG00000036875	Dna2	-0.949802994	6.45158E-05
ENSMUSG00000090053	Pakap	-0.950920809	2.29897E-17
ENSMUSG00000039298	Cdk5rap2	-0.951765448	1.397E-46
ENSMUSG00000042895	Abra	-0.952954863	0.002775788
ENSMUSG00000018809	Smyd4	-0.953243504	1.41514E-12
ENSMUSG00000002274	Metrn	-0.953481299	1.10418E-33
ENSMUSG00000032340	Neo1	-0.953612616	1.3965E-136
ENSMUSG00000097006	9530082P21Rik	-0.954094978	0.000399948
ENSMUSG00000040651	Tasor	-0.954395737	3.23518E-53
ENSMUSG00000057778	Cyb5d2	-0.954805184	2.88522E-15
ENSMUSG00000025747	Tyms	-0.956039151	1.75829E-63
ENSMUSG00000052241	A930035D04Rik	-0.956282496	0.00984352

ENSMUSG00000029260	Ugt2b34	-5.74150699	0.032702004	ENSMUSG00000026274	Pask	-0.956592691	1.30519E-12
ENSMUSG00000079563	Pglyrp2	-5.78245982	0.026523595	ENSMUSG00000024869	Gm49405	-0.95710982	0.000332604
ENSMUSG00000042118	Bhmt2	-5.821140555	0.023444328	ENSMUSG00000073164	2410018L13Rik	-0.957319572	2.46287E-07
ENSMUSG00000114656	2810403G07Rik	-5.821140555	0.023444328	ENSMUSG00000027961	Lrrc39	-0.957411892	9.99795E-12
ENSMUSG00000025396	Hsd17b6	-5.825147771	0.023636442	ENSMUSG00000003070	Efna2	-0.958558674	0.047312954
ENSMUSG00000086495	Gm13778	-5.82636373	0.008141235	ENSMUSG00000018547	Pip4k2b	-0.958578172	1.17997E-71
ENSMUSG00000031285	Dcx	-5.829504513	0.010531368	ENSMUSG00000025867	Cplx2	-0.95865211	7.49635E-38
ENSMUSG00000117608	AC103362.1	-5.831482095	0.022906619	ENSMUSG00000022673	Mcm4	-0.959102347	9.8912E-124
ENSMUSG00000024863	Mbl2	-5.85710869	0.025052864	ENSMUSG00000071722	Spin4	-0.960237812	1.00474E-07
ENSMUSG00000054910	4931415C17Rik	-5.860979463	0.008481513	ENSMUSG00000032349	Elovl5	-0.961234318	3.40013E-78
ENSMUSG00000022583	Ly6f	-5.867898647	0.006344591	ENSMUSG00000033405	Nudt15	-0.961594881	4.64161E-05
ENSMUSG00000021336	Slc17a4	-5.870105609	0.006953416	ENSMUSG00000021203	Otub2	-0.962967653	5.52957E-07
ENSMUSG00000031323	Dmrtc1a	-5.904655084	0.006241318	ENSMUSG00000089945	Pakap	-0.963744448	3.17767E-31
ENSMUSG00000033007	Asic4	-5.905319165	0.01882086	ENSMUSG00000094526	Gm21451	-0.964135821	4.4409E-186
ENSMUSG00000042707	Dnali1	-5.907991856	0.019205459	ENSMUSG00000118394	Gm50475	-0.964333148	0.047693698
ENSMUSG00000026542	Apcs	-5.907991856	0.019205459	ENSMUSG00000037455	Slc18b1	-0.964879655	9.71112E-25
ENSMUSG00000093587	Gm20554	-5.91015294	0.020316814	ENSMUSG00000084883	Ccdc85c	-0.966345737	2.32541E-07
ENSMUSG00000037335	Hand1	-5.911034066	0.007302674	ENSMUSG00000067276	Capn6	-0.969093379	3.89276E-27
ENSMUSG00000036892	Prodh2	-5.943485516	0.016949071	ENSMUSG00000055761	Nkain3	-0.969705532	0.000186273
ENSMUSG00000102925	Gm37150	-5.950472903	0.005294287	ENSMUSG00000005124	Ccn4	-0.970130596	5.4094E-149
ENSMUSG00000024694	Keg1	-5.984800027	0.015299889	ENSMUSG00000041324	Inhba	-0.97047234	1.4554E-181
ENSMUSG00000085147	Gm12609	-6.018987608	0.007580798	ENSMUSG00000045273	Cenph	-0.970825053	8.89202E-13
ENSMUSG00000062624	Cyp2c67	-6.0209736	0.01367363	ENSMUSG00000019647	Sema6a	-0.97101516	2.80421E-09
ENSMUSG00000020932	Gfap	-6.021259834	0.003551885	ENSMUSG00000032344	Cgas	-0.971068845	2.04852E-11
ENSMUSG00000067225	Cyp2c54	-6.021473165	0.013695187	ENSMUSG00000020652	Cenpo	-0.971233714	6.22638E-17
ENSMUSG00000115936	4833415N18Rik	-6.024079506	0.003469151	ENSMUSG00000018334	Ksr1	-0.971277224	1.65154E-28
ENSMUSG00000085427	6430710C18Rik	-6.024660785	0.003534139	ENSMUSG00000027009	Itga4	-0.971644385	6.0778E-112
ENSMUSG00000089678	Agxt2	-6.049883635	0.015094313	ENSMUSG00000052684	Jun	-0.972605756	4.97926E-72
ENSMUSG00000052520	Cyp2j5	-6.058698179	0.01244778	ENSMUSG00000022978	Mis18a	-0.973536041	3.56193E-18
ENSMUSG00000026272	Agxt	-6.090705929	0.01130979	ENSMUSG00000042167	Tent2	-0.974696889	1.15175E-80
ENSMUSG00000056569	Mpz	-6.092471607	0.00282284	ENSMUSG00000034858	Fam214a	-0.975203295	3.79336E-39
ENSMUSG00000014542	Clec4f	-6.096411983	0.011684603	ENSMUSG00000038677	Scube3	-0.976615569	0.007784843
ENSMUSG00000027296	Itpka	-6.099402391	0.003457791	ENSMUSG00000028339	Col15a1	-0.976661259	1.61676E-15
ENSMUSG00000058755	Osm	-6.101430019	0.011715219	ENSMUSG00000103649	Gm37768	-0.977140766	0.000758308
ENSMUSG00000106461	Gm20755	-6.101854613	0.01170322	ENSMUSG00000047759	Hs3st3a1	-0.978222336	0.001271224
ENSMUSG00000104149	Gm37138	-6.163704051	0.001915783	ENSMUSG00000047793	Sned1	-0.979374202	4.62573E-10
ENSMUSG00000064147	Rab44	-6.190228705	0.002832385	ENSMUSG00000027220	Syt13	-0.979748538	0.014876472
ENSMUSG00000049593	Lce1h	-6.221669013	0.002930711	ENSMUSG00000025498	Irf7	-0.980288222	0.00127161
ENSMUSG00000097476	Gm26583	-6.225787661	0.001922041	ENSMUSG00000024998	Plce1	-0.980334972	5.0662E-33
ENSMUSG00000110447	Gm31518	-6.229683231	0.001458611	ENSMUSG00000022853	Ehhadh	-0.980527385	1.28796E-09
ENSMUSG00000062074	Ccn6	-6.265681972	0.001895403	ENSMUSG00000064141	Zfp69	-0.980656695	0.028091044
ENSMUSG00000030108	Slc6a13	-6.291367097	0.001134114	ENSMUSG00000039217	Il18	-0.980882164	0.04176048
ENSMUSG00000108617	Gm31749	-6.347599853	0.001148259	ENSMUSG00000055612	Cdca7	-0.980916952	1.22378E-17
ENSMUSG00000036353	P2ry12	-6.356542504	0.001192105	ENSMUSG00000060743	H3f3a	-0.982125086	3.0689E-158
ENSMUSG00000025991	Cps1	-6.382087087	0.002379235	ENSMUSG00000117775	Gm50462	-0.982214854	0.007403596
ENSMUSG00000078161	Erich3	-6.382512766	0.000787395	ENSMUSG00000025140	Pycr1	-0.982596517	1.06408E-11
ENSMUSG00000053889	Kirrel3os	-6.406891555	0.000730216	ENSMUSG00000021611	Tert	-0.982764856	6.0585E-07
ENSMUSG00000073491	Ifi213	-6.409715432	0.000711018	ENSMUSG00000034520	Gjc1	-0.98338341	1.42283E-39
ENSMUSG00000039179	Tekt5	-6.432131116	0.000976518	ENSMUSG00000034275	Igsf9b	-0.984032519	2.0093E-16
ENSMUSG00000103383	Gm37754	-6.488143081	0.000567649	ENSMUSG00000026546	Cfap45	-0.98455864	1.45342E-10
ENSMUSG00000034528	Hsd17b13	-6.493517615	0.000548769	ENSMUSG00000022265	Ank	-0.984595898	2.001E-108
ENSMUSG00000027345	4921508D12Rik	-6.519517202	0.000376591	ENSMUSG00000028414	Fktn	-0.984950933	4.81281E-44
ENSMUSG00000074595	Wfdc6a	-6.543709109	0.000331859	ENSMUSG00000097915	A330009N23Rik	-0.98537291	0.01245556
ENSMUSG00000022490	Ppp1r1a	-6.572879422	0.000323154	ENSMUSG00000057137	Tmem140	-0.985629675	4.76274E-19
ENSMUSG00000025422	Agap2	-6.715628854	0.000130722	ENSMUSG00000015217	Hmgb3	-0.986887086	5.19656E-20
ENSMUSG00000116009	Gm49503	-6.75988392	0.000147807	ENSMUSG00000026434	Nucks1	-0.987187426	6.7293E-113
ENSMUSG00000035948	Acss3	-6.822443257	8.93417E-05	ENSMUSG00000040260	Daam2	-0.988374762	2.84851E-11
ENSMUSG00000020017	Hal	-6.887654561	0.000121403	ENSMUSG00000059022	Kcp	-0.989682565	0.002511324
ENSMUSG00000114291	Gm47471	-6.904531634	5.96894E-05	ENSMUSG00000039153	Runx2	-0.990230581	3.94432E-26
ENSMUSG00000054966	Lmntd1	-6.92970587	4.53231E-05	ENSMUSG00000000686	Abhd15	-0.991334603	2.7262E-05
ENSMUSG00000062296	Trank1	-6.965658242	3.48896E-05	ENSMUSG00000017314	Mpp2	-0.99135119	1.47577E-15
ENSMUSG00000039653	Baat	-6.983976488	6.41665E-05	ENSMUSG00000045672	Col27a1	-0.992077872	1.74493E-13
ENSMUSG00000022149	C9	-7.077392352	5.81524E-05	ENSMUSG00000029238	Clock	-0.992190758	5.5262E-102
ENSMUSG00000028715	Cyp4a14	-7.517741491	5.75712E-06	ENSMUSG00000025817	Nudt5	-0.993641794	7.04735E-65
ENSMUSG00000029155	Spata18	-7.677971699	4.27987E-07	ENSMUSG00000029664	Tfpi2	-0.995068013	2.35123E-82
ENSMUSG00000060371	Caln1	-7.94489182	6.50942E-08	ENSMUSG00000037126	Psd	-0.99533406	3.4067E-08
ENSMUSG00000021492	F12	-8.003859149	5.18022E-07	ENSMUSG00000046668	Cxxc5	-0.995925435	3.36682E-26
ENSMUSG00000047631	Apof	-8.067078744	1.40191E-07	ENSMUSG00000031661	Nkd1	-0.996312746	2.0505E-05
ENSMUSG00000071178	Serpina1b	-8.081921047	0.003216322	ENSMUSG00000004748	Mtfp1	-0.996540441	0.01630066
ENSMUSG00000015854	Cd5l	-8.090784747	3.29191E-07	ENSMUSG00000037894	H2az1	-0.996751591	1.9716E-183
ENSMUSG00000029685	Asb15	-8.361466096	3.03901E-09	ENSMUSG00000026988	Wdsub1	-0.997387244	3.75884E-19
ENSMUSG00000069805	Fbp1	-8.381273028	3.72251E-08	ENSMUSG00000043157	Arl11	-0.997828168	0.008001586
ENSMUSG00000068086	Cyp2d9	-9.185944388	0.000160335	ENSMUSG00000027848	Olfml3	-0.998178795	1.28904E-90
ENSMUSG00000069922	Ces3a	-9.345820504	0.04979801	ENSMUSG00000017692	Rhbdl3	-0.998464322	0.006703467
ENSMUSG00000032081	Apoc3	-9.395931641	0.000253014	ENSMUSG00000043372	Hexim2	-0.998523297	0.01592668
ENSMUSG00000078674	Mup18	-9.649828517	0.041164282	ENSMUSG00000028475	Spaar	-0.998553802	9.8092E-10
ENSMUSG00000059908	Mug1	-9.741706667	0.038807083	ENSMUSG00000051652	Lrrc3	-0.999052624	0.020400391

ENSMUSG00000078673	Mup19	-9.818522612	0.036951507
ENSMUSG00000110439	Mup22	-9.966119021	0.033611399
ENSMUSG00000029445	Hpd	-9.973076498	0.000129747
ENSMUSG00000059481	Plg	-9.995094756	0.032993619
ENSMUSG00000078687	Mup8	-10.01544754	0.032588277
ENSMUSG00000096688	Mup17	-10.02443635	0.032398507
ENSMUSG00000078683	Mup1	-10.03426772	0.032193926
ENSMUSG00000030359	Pzp	-10.04221686	6.2166E-05
ENSMUSG00000078688	Mup2	-10.06367669	0.031563798
ENSMUSG00000028356	Ambp	-10.13090973	0.030198852
ENSMUSG00000073830	Mup14	-10.16905443	0.029457929
ENSMUSG00000028307	Aldob	-10.19279688	0.029013598
ENSMUSG00000078686	Mup9	-10.22443176	0.028394959
ENSMUSG00000078672	Mup20	-10.28154276	0.027341859
ENSMUSG00000028001	Fga	-10.52799681	0.023102784
ENSMUSG00000071177	Serpina1d	-10.53520757	0.023003647
ENSMUSG00000017344	Vtn	-10.55272142	7.80294E-05
ENSMUSG00000073834	Mup11	-10.64664717	0.021309859
ENSMUSG00000072849	Serpina1e	-10.698681	0.020534028
ENSMUSG00000066154	Mup3	-10.81354723	0.018965945
ENSMUSG00000035540	Gc	-10.8627102	0.018295175
ENSMUSG00000061808	Ttr	-10.9043646	0.017781182
ENSMUSG00000078680	Mup10	-11.11700212	0.015261834
ENSMUSG00000073842	Mup7	-11.16140065	0.014781454
ENSMUSG00000066366	Serpina1a	-11.24905557	0.000198927
ENSMUSG00000116024	Gm49527	-11.26897561	0.013665328
ENSMUSG00000025479	Cyp2e1	-11.80675514	0.00915022
ENSMUSG00000079015	Serpina1c	-11.85765112	0.000135146
ENSMUSG00000032083	Apoa1	-12.00583193	5.9963E-05
ENSMUSG00000058207	Serpina3k	-12.30522705	0.006172077
ENSMUSG00000029368	Alb	-14.10409017	5.60748E-07

ENSMUSG00000089862	Umad1	-1.000267591	2.82253E-19
ENSMUSG00000022831	Hcls1	-1.001225375	2.19941E-07
ENSMUSG00000017652	Cd40	-1.0015227	4.53985E-41
ENSMUSG00000074968	Ano3	-1.002373001	1.48677E-35
ENSMUSG00000035829	Ppp1r26	-1.003342594	0.009289058
ENSMUSG00000052889	Prkcb	-1.00338353	0.012295232
ENSMUSG00000025265	Fgd1	-1.004657858	4.78344E-37
ENSMUSG00000046818	Ddit4l	-1.004768725	2.9335E-40
ENSMUSG00000020486	Septin4	-1.005978999	3.33356E-10
ENSMUSG00000007613	Tgfbr1	-1.006038622	8.0573E-141
ENSMUSG00000037826	Ppm1k	-1.006904314	1.06349E-10
ENSMUSG00000038884	Shfl	-1.007038916	5.81827E-20
ENSMUSG00000002365	Snx9	-1.007503948	1.5147E-206
ENSMUSG00000018849	Wwc1	-1.007658456	7.03396E-34
ENSMUSG00000030657	Xylt1	-1.008485517	7.54E-25
ENSMUSG00000022893	Adamts1	-1.0095755	3.5877E-164
ENSMUSG00000017417	Plxdc1	-1.010671305	0.041568823
ENSMUSG00000015745	Plekho1	-1.011590189	2.06958E-27
ENSMUSG00000090231	Cfb	-1.012928211	1.28336E-09
ENSMUSG00000005370	Msh6	-1.013244873	1.68523E-69
ENSMUSG00000038894	Irs2	-1.013952673	1.09026E-28
ENSMUSG00000028121	Bcar3	-1.015152817	0.002224103
ENSMUSG00000037685	Atp8a1	-1.015452273	1.18973E-08
ENSMUSG00000027580	Helz2	-1.015855726	2.93032E-12
ENSMUSG00000036960	Clca2	-1.016988456	0.000764181
ENSMUSG00000038025	Phf2	-1.017775964	2.30041E-69
ENSMUSG00000028268	Gbp3	-1.018938092	0.000363437
ENSMUSG00000002997	Prkar2b	-1.01943982	1.3278E-197
ENSMUSG00000036565	Ttyh3	-1.020201121	1.79655E-42
ENSMUSG00000062075	Lmnb2	-1.020679446	5.57756E-33
ENSMUSG00000097239	Gm27029	-1.023370735	0.005559451
ENSMUSG00000070526	Peg12	-1.024310192	3.2974E-13
ENSMUSG00000021835	Bmp4	-1.024629923	2.31489E-25
ENSMUSG00000040093	Bmf	-1.025044299	5.31683E-05
ENSMUSG00000025912	Mybl1	-1.025309206	9.17036E-29
ENSMUSG00000041889	Shisa4	-1.02542753	1.77558E-37
ENSMUSG00000039943	Plcb4	-1.025901071	1.23111E-52
ENSMUSG00000046985	Tapt1	-1.02599013	2.462E-51
ENSMUSG00000031026	Trim66	-1.026083117	0.017115675
ENSMUSG00000031840	Rab3a	-1.0260907	3.02328E-12
ENSMUSG00000087264	Gad1os	-1.026114962	0.038863419
ENSMUSG00000047496	Rnf152	-1.026300877	0.016355007
ENSMUSG00000066258	Trim12a	-1.026747537	1.40051E-22
ENSMUSG00000097440	Gm6277	-1.026904892	0.004814669
ENSMUSG00000041245	Wnk3	-1.027393341	1.14017E-07
ENSMUSG00000022803	Popdc2	-1.028106764	1.27321E-12
ENSMUSG00000017830	Dhx58	-1.028422167	8.85137E-10
ENSMUSG00000031906	Smpd3	-1.029049059	2.42827E-26
ENSMUSG00000027784	Ppm1l	-1.029105028	9.64883E-07
ENSMUSG00000042302	Ehbp1	-1.029539308	6.59842E-35
ENSMUSG00000040669	Phc1	-1.030259904	6.75267E-38
ENSMUSG00000027203	Dut	-1.030327327	3.53301E-22
ENSMUSG00000086544	Chn1os3	-1.03132427	0.00137847
ENSMUSG00000070473	Cldn3	-1.031510032	1.08084E-19
ENSMUSG00000074916	Chst14	-1.031602782	3.23358E-32
ENSMUSG00000047420	Fam180a	-1.032748263	4.33852E-09
ENSMUSG00000051413	Plagl2	-1.033727149	3.97363E-38
ENSMUSG00000000093	Tbx2	-1.03424733	5.89065E-17
ENSMUSG00000027459	Fam110a	-1.034720252	1.41515E-17
ENSMUSG00000098098	Bvht	-1.034908779	3.08084E-09
ENSMUSG00000020534	Shmt1	-1.035075897	1.31141E-13
ENSMUSG00000052632	Asap2	-1.035503075	4.18255E-69
ENSMUSG00000027460	Angpt4	-1.035684034	0.000708614
ENSMUSG00000096056	Gm21986	-1.035816698	0.020505041
ENSMUSG00000036678	Aaas	-1.036605387	6.86864E-53
ENSMUSG00000091553	Serpina3e-ps	-1.036693365	0.004600772
ENSMUSG00000018339	Gpx3	-1.037918756	8.2859E-102
ENSMUSG00000013878	Rnf170	-1.039266413	6.01041E-53
ENSMUSG00000079491	H2-T10	-1.03954182	2.22267E-06
ENSMUSG00000020123	Avpr1a	-1.040078413	2.69483E-09
ENSMUSG00000035126	Wdr78	-1.040438134	4.59197E-05
ENSMUSG00000032702	Kank1	-1.040492556	8.3584E-115
ENSMUSG00000091191	Gm17334	-1.041650467	0.009718931
ENSMUSG00000021906	Oxnad1	-1.041711717	6.73426E-16
ENSMUSG00000052143	Gm9869	-1.042057237	4.06856E-06
ENSMUSG00000114255	Gm10734	-1.042570833	0.045826858
ENSMUSG00000006800	Sulf2	-1.042728239	1.0431E-130

ENSMUSG00000020423	Btg2	-1.042809504	6.3953E-107
ENSMUSG00000025156	Gps1	-1.044143093	3.7522E-130
ENSMUSG00000026207	Speg	-1.044308677	4.47869E-29
ENSMUSG00000068551	Zfp467	-1.045591086	1.9463E-05
ENSMUSG00000073409	H2-Q6	-1.045661509	0.016017286
ENSMUSG00000033967	Rnf225	-1.046365292	0.000431343
ENSMUSG00000000627	Sema4f	-1.0487758	1.0051E-07
ENSMUSG00000049657	Zbtb5	-1.0493413	4.72004E-18
ENSMUSG00000037321	Tap1	-1.049714512	7.42379E-20
ENSMUSG00000074818	Pdzd7	-1.050168331	0.001010937
ENSMUSG00000070427	Il18bp	-1.050369431	1.91479E-09
ENSMUSG00000022881	Rfc4	-1.050394664	1.20482E-13
ENSMUSG00000046618	Olfml2a	-1.051506083	0.000273291
ENSMUSG00000027796	Smad9	-1.052390395	4.32738E-12
ENSMUSG00000046491	C1qtnf2	-1.052480598	6.78889E-29
ENSMUSG00000036672	Cenpt	-1.053258866	1.75993E-51
ENSMUSG00000030772	Dkk3	-1.054270218	3.24582E-73
ENSMUSG00000031506	Ptpn7	-1.054706974	0.007394797
ENSMUSG00000063919	Srrm4	-1.055733835	0.003711607
ENSMUSG00000020877	Scrn2	-1.056010354	1.21232E-07
ENSMUSG00000111275	Gm29824	-1.056340727	0.016941882
ENSMUSG00000036777	Anln	-1.056608005	1.381E-157
ENSMUSG00000031669	Gins3	-1.057470109	3.58986E-12
ENSMUSG00000026659	Dusp12	-1.057680258	2.43671E-21
ENSMUSG00000047193	Dync2h1	-1.057728026	3.42398E-30
ENSMUSG00000000037	Scml2	-1.05803885	5.29155E-07
ENSMUSG00000025154	Arhgap19	-1.058310663	1.30496E-18
ENSMUSG00000021592	Arsk	-1.059020869	2.27682E-23
ENSMUSG00000004151	Etv1	-1.059176142	6.70954E-23
ENSMUSG00000066861	Oas1g	-1.060102947	0.001088623
ENSMUSG00000029636	Wasf3	-1.060186846	4.55896E-06
ENSMUSG00000106464	C130083M11Rik	-1.060324806	4.17627E-10
ENSMUSG00000007097	Atp1a2	-1.06068725	0.004755742
ENSMUSG00000037235	Mxd4	-1.060740929	1.1172E-119
ENSMUSG00000102543	Pcdhgc5	-1.060956677	1.43988E-07
ENSMUSG00000024524	Gnal	-1.061038822	0.000426372
ENSMUSG00000097960	A330074K22Rik	-1.061680778	0.019562066
ENSMUSG00000061607	Mdc1	-1.061927538	2.38892E-59
ENSMUSG00000041936	Agrn	-1.062382455	1.82034E-36
ENSMUSG00000032011	Thy1	-1.062690394	5.7814E-269
ENSMUSG00000027932	Slc27a3	-1.063779465	9.09564E-17
ENSMUSG00000021365	Nedd9	-1.064286735	3.2634E-121
ENSMUSG00000037020	Wdr62	-1.064668007	4.76995E-27
ENSMUSG00000029309	Sparcl1	-1.064885563	3.67248E-37
ENSMUSG00000082676	Gm11843	-1.065312964	0.038180586
ENSMUSG00000096210	H1f0	-1.065677922	2.3478E-142
ENSMUSG00000031821	Gins2	-1.066165836	3.93172E-35
ENSMUSG00000079654	Prrt4	-1.067189125	2.39513E-06
ENSMUSG00000076431	Sox4	-1.068819171	2.9697E-174
ENSMUSG00000028884	Rpa2	-1.068925597	2.84524E-28
ENSMUSG00000070802	Pnmal2	-1.06900637	0.000817018
ENSMUSG00000019866	Crybg1	-1.070489895	5.38964E-05
ENSMUSG00000003452	Bicd1	-1.071556501	1.35247E-08
ENSMUSG00000038600	Atp6v0a4	-1.071798899	0.021525172
ENSMUSG00000001655	Hoxc13	-1.071912726	0.033490346
ENSMUSG00000001151	Pcnt	-1.072314908	3.746E-39
ENSMUSG00000053626	Tll1	-1.072490818	4.90979E-23
ENSMUSG00000018931	Natd1	-1.072635909	7.68969E-67
ENSMUSG00000024347	Psd2	-1.073050143	0.043983193
ENSMUSG00000099689	Zfp383	-1.0741764	3.9098E-16
ENSMUSG00000060716	Plekhh1	-1.074466212	3.51844E-11
ENSMUSG00000033763	Mtss2	-1.074685962	4.57764E-34
ENSMUSG00000046694	Tent5b	-1.074915061	0.000588965
ENSMUSG00000027381	Bcl2l11	-1.075465928	6.08792E-15
ENSMUSG00000034544	Rsrc1	-1.076756387	1.99663E-57
ENSMUSG00000029687	Ezh2	-1.077186456	6.67222E-51
ENSMUSG00000037440	Vnn1	-1.077238554	9.32355E-08
ENSMUSG00000023243	Kcnk5	-1.078287486	1.31071E-06
ENSMUSG00000002346	Slc25a42	-1.078529859	2.81454E-10
ENSMUSG00000027078	Ube2l6	-1.078698503	2.55667E-63
ENSMUSG00000001542	Ell2	-1.078972156	8.1495E-115
ENSMUSG00000031886	Ces2e	-1.079042309	1.49717E-13
ENSMUSG00000075302	Erich2	-1.079379786	0.020406194
ENSMUSG00000037353	Letmd1	-1.079698485	3.01047E-45
ENSMUSG00000040612	Ildr2	-1.079926532	3.27269E-65
ENSMUSG00000003134	Tbc1d8	-1.07994367	4.227E-06
ENSMUSG00000090077	Lime1	-1.080278822	0.005214964

ENSMUSG00000097589	Dleu2	-1.08048252	5.9636E-21
ENSMUSG00000022103	Gfra2	-1.080532717	2.81342E-05
ENSMUSG00000021411	Pxdc1	-1.081369384	2.41016E-59
ENSMUSG00000029442	Wdr66	-1.081441208	2.72215E-09
ENSMUSG00000030035	Wbp1	-1.08256084	7.84503E-27
ENSMUSG00000044881	Coa4	-1.082678055	3.09846E-08
ENSMUSG00000013236	Ptprs	-1.084134748	2.9841E-162
ENSMUSG00000051098	Mblac2	-1.085580813	3.31433E-06
ENSMUSG00000001604	Tcea3	-1.086458998	5.38407E-17
ENSMUSG00000105112	Gm42778	-1.087425791	0.016374165
ENSMUSG00000035799	Twist1	-1.088408679	1.2265E-37
ENSMUSG00000026466	Tor1aip1	-1.089132033	2.70907E-22
ENSMUSG00000047115	Fam221a	-1.089916333	0.000774565
ENSMUSG00000022833	Ccdc14	-1.090018735	3.91925E-12
ENSMUSG00000034881	Tbxa2r	-1.090776444	0.017916929
ENSMUSG00000048200	Cracr2b	-1.091048167	8.91966E-12
ENSMUSG00000110350	Gm10252	-1.091395686	0.00460698
ENSMUSG00000026764	Kif5c	-1.092203185	4.27364E-11
ENSMUSG00000049191	Rtl5	-1.092368502	2.23316E-41
ENSMUSG00000042210	Abhd14a	-1.092475363	9.46894E-24
ENSMUSG00000054404	Slfn5	-1.092701364	5.99205E-43
ENSMUSG00000028018	Gstcd	-1.093495215	1.08383E-26
ENSMUSG00000017386	Traf4	-1.093728391	9.87232E-69
ENSMUSG00000039377	Hlx	-1.094408903	7.44512E-12
ENSMUSG00000046879	Irgm1	-1.09507711	3.70889E-21
ENSMUSG00000020525	Ppm1d	-1.095792449	1.44235E-36
ENSMUSG00000016262	Sertad4	-1.09627385	1.0977E-06
ENSMUSG00000031618	Nr3c2	-1.096593716	0.019838971
ENSMUSG00000039231	Suv39h1	-1.096660884	9.29176E-53
ENSMUSG00000098488	Pla2g4b	-1.097015927	0.00216396
ENSMUSG00000040350	Trim7	-1.097048526	1.9384E-11
ENSMUSG00000001986	Gria3	-1.097634488	5.1537E-100
ENSMUSG00000020902	Ntn1	-1.097786925	8.5872E-88
ENSMUSG00000021936	Mapk8	-1.098041907	1.37136E-63
ENSMUSG00000087179	Gm14230	-1.098550393	8.86159E-07
ENSMUSG00000024236	Svil	-1.098996608	3.90385E-98
ENSMUSG00000044576	Garem2	-1.100441225	1.18463E-71
ENSMUSG00000032122	Slc37a2	-1.100906446	9.98873E-14
ENSMUSG00000025268	Maged2	-1.101032713	2.2338E-190
ENSMUSG00000078247	Airn	-1.10175824	2.67497E-08
ENSMUSG00000055760	Gemin6	-1.102871961	1.01534E-20
ENSMUSG00000027833	Shox2	-1.103144982	2.89101E-10
ENSMUSG00000044005	Gls2	-1.103228296	0.022649486
ENSMUSG00000070469	Adamtsl3	-1.104332965	7.6966E-148
ENSMUSG00000028464	Tpm2	-1.104346304	2.8772E-107
ENSMUSG00000021262	Evl	-1.104379314	1.13567E-38
ENSMUSG00000037997	Parp11	-1.105353753	3.85974E-18
ENSMUSG00000046605	B3gntl1	-1.105546862	1.28735E-07
ENSMUSG00000013033	Adgrl1	-1.106438608	9.47582E-64
ENSMUSG00000046916	Myct1	-1.10668833	9.02921E-12
ENSMUSG00000058444	Map2k5	-1.107283587	8.74801E-24
ENSMUSG00000042116	Vwa1	-1.108360429	0.003943854
ENSMUSG00000050556	Kcnb1	-1.109120689	0.000213161
ENSMUSG00000061048	Cdh3	-1.110965023	0.020600712
ENSMUSG00000018012	Rac3	-1.11129913	0.0006679
ENSMUSG00000019122	Ccl9	-1.111492215	1.6989E-104
ENSMUSG00000036553	Sh3tc1	-1.113202959	3.50363E-10
ENSMUSG00000038855	Itpkb	-1.113792743	3.26618E-81
ENSMUSG00000082226	Gm715	-1.114364942	0.041523292
ENSMUSG00000030703	Gdpd3	-1.11488578	0.02407487
ENSMUSG00000074925	Ptar1	-1.115274254	4.30699E-93
ENSMUSG00000044224	Dnajc21	-1.115514339	2.43995E-54
ENSMUSG00000042099	Kank3	-1.115951016	1.47779E-06
ENSMUSG00000074480	Mex3a	-1.115994875	2.34181E-18
ENSMUSG00000027978	Prss12	-1.117182792	2.18864E-08
ENSMUSG00000059939	9430015G10Rik	-1.117223739	2.72442E-14
ENSMUSG00000034303	Ccdc15	-1.117367935	9.83939E-07
ENSMUSG00000008730	Hipk1	-1.117943522	2.7693E-197
ENSMUSG00000026281	Dtymk	-1.119485566	7.64749E-52
ENSMUSG00000048388	Fam171b	-1.120356952	4.04293E-54
ENSMUSG00000022468	Endou	-1.120382241	0.020526709
ENSMUSG00000056025	Clca3a1	-1.120595404	0
ENSMUSG00000047003	Zfp41	-1.120913597	4.39029E-13
ENSMUSG00000044807	Zfp354c	-1.121109566	1.86812E-26
ENSMUSG00000079465	Col4a3	-1.121838638	1.53797E-34
ENSMUSG00000111521	Gm48529	-1.122820348	4.76691E-23
ENSMUSG00000021952	Xpo4	-1.122861499	4.18243E-80

ENSMUSG00000023345	Poc1a	-1.122903621	3.01988E-14
ENSMUSG00000032177	Pde4a	-1.124359951	1.3419E-59
ENSMUSG00000020238	Ncln	-1.125202229	1.038E-111
ENSMUSG00000076437	Selenoh	-1.126645444	1.98291E-23
ENSMUSG00000009585	Apobec3	-1.126773158	1.38608E-17
ENSMUSG00000030254	Rad18	-1.127446477	3.09898E-63
ENSMUSG00000090215	Trim34b	-1.128008345	0.0230846
ENSMUSG00000020707	Rnf135	-1.128010651	1.4658E-13
ENSMUSG00000041117	Ccdc8	-1.128253864	2.27734E-29
ENSMUSG00000026147	Col9a1	-1.128458935	0.03046116
ENSMUSG00000021978	Extl3	-1.128875019	2.2547E-172
ENSMUSG00000021820	Camk2g	-1.129879121	5.39541E-64
ENSMUSG00000002006	Pdzd4	-1.130878599	3.10045E-08
ENSMUSG00000051278	Zgrf1	-1.132349514	4.63206E-22
ENSMUSG00000021811	Dnajc9	-1.132936569	7.63185E-87
ENSMUSG00000022945	Chaf1b	-1.13323634	3.1145E-23
ENSMUSG00000028807	Zbtb8a	-1.133927301	8.13049E-11
ENSMUSG00000068854	H2bc21	-1.134433531	0.000126555
ENSMUSG00000032417	Rwdd2a	-1.134935827	0.003241192
ENSMUSG00000029765	Plxna4	-1.135590121	1.52885E-34
ENSMUSG00000031529	Tnks	-1.136383953	7.217E-152
ENSMUSG00000020623	Map2k6	-1.136756007	2.68636E-10
ENSMUSG00000070576	Mn1	-1.138508779	6.58144E-23
ENSMUSG00000038736	Nudcd1	-1.139248842	3.35769E-53
ENSMUSG00000041828	Abca8a	-1.139345664	4.84318E-97
ENSMUSG00000028152	Tspan5	-1.139562587	1.6817E-44
ENSMUSG00000055980	Irs1	-1.13957651	3.35767E-51
ENSMUSG00000042662	Dusp15	-1.139822278	1.40506E-06
ENSMUSG00000005465	Il27ra	-1.140069699	3.1057E-16
ENSMUSG00000020990	Cdkl1	-1.140884557	0.010417268
ENSMUSG00000032289	Thsd4	-1.141049666	0.018090663
ENSMUSG00000031877	Ces2g	-1.141086726	4.39056E-66
ENSMUSG00000026640	Plxna2	-1.141550575	1.0806E-70
ENSMUSG00000026104	Stat1	-1.14291802	3.94288E-10
ENSMUSG00000026832	Cytip	-1.143023665	0.00053146
ENSMUSG00000022092	Ppp3cc	-1.143207593	6.18011E-36
ENSMUSG00000063568	Jazf1	-1.14327926	2.9469E-17
ENSMUSG00000087651	1500009L16Rik	-1.143761993	7.83063E-21
ENSMUSG00000073705	Cenps	-1.144297096	1.24312E-08
ENSMUSG00000041642	Kif21b	-1.145242944	7.84463E-15
ENSMUSG00000004319	Clcn3	-1.145436685	1.16755E-71
ENSMUSG00000028037	Ifi44	-1.147134566	0.002043066
ENSMUSG00000050675	Gp1ba	-1.147333964	0.005196023
ENSMUSG00000095159	Tubb4b-ps1	-1.14857201	1.53716E-14
ENSMUSG00000079410	Gm2897	-1.149157346	0.002578276
ENSMUSG00000070327	Rnf213	-1.149157415	5.80388E-15
ENSMUSG00000090210	Itga10	-1.149979263	1.9271E-07
ENSMUSG00000028245	Nsmaf	-1.151109259	9.19416E-50
ENSMUSG00000032410	Xrn1	-1.152016333	6.12474E-76
ENSMUSG00000038332	Sesn1	-1.152972219	2.5923E-15
ENSMUSG00000086681	Gm16178	-1.153498918	0.030388528
ENSMUSG00000038042	Ptpdc1	-1.153755421	3.85467E-28
ENSMUSG00000070720	Tmem200b	-1.15439565	9.9382E-09
ENSMUSG00000031924	Cyb5b	-1.154686018	2.1439E-177
ENSMUSG00000049001	Ndnf	-1.156424447	7.07675E-31
ENSMUSG00000105402	Gm3716	-1.156718041	0.014156645
ENSMUSG00000023972	Ptk7	-1.156992489	7.0096E-147
ENSMUSG00000001988	Npas1	-1.157686556	0.008477333
ENSMUSG00000016087	Fli1	-1.158128489	8.22163E-59
ENSMUSG00000025650	Col7a1	-1.15886322	7.46071E-18
ENSMUSG00000019874	Fabp7	-1.15930697	0.007286724
ENSMUSG00000038244	Mical2	-1.159343916	0
ENSMUSG00000031748	Gnao1	-1.161082095	1.48723E-19
ENSMUSG00000072244	Trim6	-1.162048718	3.55057E-08
ENSMUSG00000021707	Dhfr	-1.162125025	5.13589E-46
ENSMUSG00000020439	Smtn	-1.162708219	1.5158E-179
ENSMUSG00000022385	Gtse1	-1.162932669	3.5122E-86
ENSMUSG00000108046	Gm43924	-1.163292902	0.003783122
ENSMUSG00000041809	Efhc1	-1.163372164	0.002396516
ENSMUSG00000090257	Gm4524	-1.163736687	0.006972677
ENSMUSG00000032815	Fanca	-1.164465546	3.1217E-39
ENSMUSG00000023066	Rttn	-1.164566421	5.69156E-16
ENSMUSG00000036752	Tubb4b	-1.165952188	0
ENSMUSG00000085783	Gm9816	-1.166304969	0.000237533
ENSMUSG00000030411	Nova2	-1.166334265	0.027951153
ENSMUSG00000026785	Pkn3	-1.167024694	9.30068E-12
ENSMUSG00000114660	Gm47881	-1.167271374	0.026090568

ENSMUSG00000111977	Gm47163	-1.167340137	0.016952821
ENSMUSG00000036086	Zranb3	-1.167694212	1.00798E-07
ENSMUSG00000029510	Gpc2	-1.168577885	0.017107199
ENSMUSG00000009246	Trpm5	-1.168604751	9.98173E-06
ENSMUSG00000026843	Fubp3	-1.170197848	2.4644E-162
ENSMUSG00000035024	Ncapd3	-1.172491757	4.34159E-43
ENSMUSG00000026708	Cenpl	-1.172718854	7.87787E-22
ENSMUSG00000051279	Gdf6	-1.173075654	2.3829E-292
ENSMUSG00000032402	Smad3	-1.173439008	1.059E-148
ENSMUSG00000056888	Glipr1	-1.17411861	1.79169E-37
ENSMUSG00000022309	Angpt1	-1.175164386	1.39592E-05
ENSMUSG00000037253	Mex3c	-1.177002307	1.2146E-194
ENSMUSG00000041444	Arhgap32	-1.178793044	4.02145E-82
ENSMUSG00000008658	Rbfox1	-1.179896773	2.05479E-14
ENSMUSG00000039236	Isg20	-1.180571697	1.64332E-07
ENSMUSG00000020102	Slc16a7	-1.180581975	0.000679081
ENSMUSG00000079481	Nhsl2	-1.180775752	1.73074E-11
ENSMUSG00000044864	Ankrd50	-1.184742262	1.0457E-291
ENSMUSG00000020609	Apob	-1.185212314	0.026647014
ENSMUSG00000085184	4933439K11Rik	-1.185489012	0.026128856
ENSMUSG00000037541	Shank2	-1.185515484	4.62711E-05
ENSMUSG00000025665	Rps6ka6	-1.185653671	0.016307104
ENSMUSG00000025271	Pfkfb1	-1.185799678	1.27576E-06
ENSMUSG00000024043	Arhgap28	-1.185804198	5.60216E-27
ENSMUSG00000031284	Pak3	-1.1862552	1.48632E-88
ENSMUSG00000022240	Ctnnd2	-1.188440769	5.36705E-09
ENSMUSG00000024222	Fkbp5	-1.189735773	7.7377E-101
ENSMUSG00000026646	Suv39h2	-1.190137796	3.84804E-11
ENSMUSG00000032607	Amt	-1.190394859	4.92907E-05
ENSMUSG00000025207	Sema4g	-1.19081507	0.002068727
ENSMUSG00000089755	0610012D04Rik	-1.190885496	0.024900124
ENSMUSG00000110537	Gm4316	-1.190890629	5.93559E-05
ENSMUSG00000030929	Eri2	-1.191035988	2.36522E-42
ENSMUSG00000028066	Pmf1	-1.192838025	1.96694E-40
ENSMUSG00000035842	Ddx11	-1.19350624	3.63172E-23
ENSMUSG00000026429	Ube2t	-1.193654377	1.03851E-35
ENSMUSG00000068762	Gstm6	-1.193657741	0.000583954
ENSMUSG00000041827	Oasl1	-1.193696223	0.004826814
ENSMUSG00000052676	Zmat1	-1.193807861	2.42024E-10
ENSMUSG00000043635	Adamts3	-1.194942494	0.009315232
ENSMUSG00000027811	4930579G24Rik	-1.195171323	7.4679E-33
ENSMUSG00000048096	Lmod1	-1.195998979	5.05213E-05
ENSMUSG00000116165	Pdxp	-1.196445083	0.030172453
ENSMUSG00000027570	Col9a3	-1.197128343	0.008335604
ENSMUSG00000028524	Sgip1	-1.197738139	1.86226E-06
ENSMUSG00000072964	Bhlhb9	-1.198081338	1.89548E-84
ENSMUSG00000033446	Lpar6	-1.198714322	9.78121E-37
ENSMUSG00000021390	Ogn	-1.199108492	1.8067E-228
ENSMUSG00000015647	Lama5	-1.199685002	7.1003E-126
ENSMUSG00000089901	Gm8113	-1.200198537	0.001142896
ENSMUSG00000022199	Slc22a17	-1.200635432	1.94508E-10
ENSMUSG00000037010	Apln	-1.200936963	1.6324E-60
ENSMUSG00000043398	Gpr135	-1.201516476	6.80793E-07
ENSMUSG00000042078	Svop	-1.201988619	0.001284875
ENSMUSG00000022015	Tnfsf11	-1.20226241	2.90001E-06
ENSMUSG00000044783	Hjurp	-1.203250081	5.7989E-241
ENSMUSG00000023886	Smoc2	-1.203382447	1.2509E-204
ENSMUSG00000033715	Akr1c14	-1.204494771	2.20363E-14
ENSMUSG00000038011	Dnah10	-1.20482767	0.006929348
ENSMUSG00000026828	Galnt5	-1.20483219	3.40513E-05
ENSMUSG00000069237	Fam8a1	-1.205538184	7.3626E-271
ENSMUSG00000029167	Ppargc1a	-1.205951059	0.011844312
ENSMUSG00000061533	Cep128	-1.206115382	7.35621E-17
ENSMUSG00000040033	Stat2	-1.206131145	5.07442E-10
ENSMUSG00000049823	Zbtb12	-1.207425927	1.67883E-08
ENSMUSG00000044469	Tnfaip8l1	-1.207488257	9.90737E-07
ENSMUSG00000034205	Loxl2	-1.208576883	0
ENSMUSG00000063268	Parp10	-1.209369182	1.43178E-34
ENSMUSG00000032717	Mdfi	-1.209390549	1.64073E-17
ENSMUSG00000002870	Mcm2	-1.209462281	2.4549E-129
ENSMUSG00000034271	Jdp2	-1.210512427	6.09026E-68
ENSMUSG00000050345	4930486L24Rik	-1.211135779	1.28449E-07
ENSMUSG00000055745	Rtl6	-1.211155074	3.56144E-12
ENSMUSG00000036863	Syde2	-1.211445553	3.9966E-13
ENSMUSG00000036036	Zfp57	-1.21148884	4.64824E-52
ENSMUSG00000051359	Ncald	-1.213630675	1.2769E-40
ENSMUSG00000045751	Mms22l	-1.214283103	9.70905E-36

ENSMUSG00000020331	Hcn2	-1.215838652	9.90233E-06
ENSMUSG00000053477	Tcf4	-1.215925537	3.219E-209
ENSMUSG00000025758	Plk4	-1.216254388	3.58401E-64
ENSMUSG00000005107	Slc2a9	-1.216537665	1.89058E-21
ENSMUSG00000032744	Heyl	-1.216559895	1.76208E-09
ENSMUSG00000007591	Tssk4	-1.216893902	0.004875415
ENSMUSG00000069874	Irgm2	-1.216973746	9.09192E-09
ENSMUSG00000045903	Npas4	-1.21846179	3.75884E-06
ENSMUSG00000049502	Dtx3l	-1.218819765	2.22976E-09
ENSMUSG00000037940	Inpp4b	-1.218856389	0.00045104
ENSMUSG00000028264	Spaca1	-1.219303089	0.031330628
ENSMUSG00000036655	Colec11	-1.219806049	2.42011E-06
ENSMUSG00000097820	E530011L22Rik	-1.219961633	0.000814638
ENSMUSG00000039997	Ifi203	-1.220531997	6.08738E-07
ENSMUSG00000085030	2810455O05Rik	-1.220717723	0.006132203
ENSMUSG00000042606	Hirip3	-1.221790476	2.60386E-18
ENSMUSG00000106025	Gm42940	-1.222063282	0.00139014
ENSMUSG00000031146	Plp2	-1.222287231	8.6186E-167
ENSMUSG00000031861	Lpar2	-1.222351445	1.67894E-26
ENSMUSG00000019877	Serinc1	-1.222581802	0
ENSMUSG00000017146	Brca1	-1.224283831	8.07261E-31
ENSMUSG00000032690	Oas2	-1.224422738	2.34482E-11
ENSMUSG00000050394	Armcx6	-1.224589968	7.03799E-12
ENSMUSG00000105449	Gm43379	-1.225368131	0.000391878
ENSMUSG00000074500	Zfp558	-1.225961168	0.000424544
ENSMUSG00000043987	Cep164	-1.226236663	8.03109E-64
ENSMUSG00000015714	Cers2	-1.227568911	1.8564E-262
ENSMUSG00000050410	Tcf19	-1.227954397	9.9308E-27
ENSMUSG00000025006	Sorbs1	-1.228035994	4.79872E-42
ENSMUSG00000087589	D430040D24Rik	-1.22939836	0.02969437
ENSMUSG00000029156	Sgcb	-1.230043935	7.1552E-113
ENSMUSG00000026096	Osgepl1	-1.230082103	4.43716E-12
ENSMUSG00000105504	Gbp5	-1.231829176	2.26785E-07
ENSMUSG00000031639	Tlr3	-1.232413801	7.71739E-24
ENSMUSG00000003779	Kif20a	-1.233287756	1.4106E-204
ENSMUSG00000019992	Mtfr2	-1.234654455	2.75053E-19
ENSMUSG00000060510	Zfp266	-1.235597504	3.24266E-98
ENSMUSG00000057762	Gm6169	-1.236073524	1.40977E-08
ENSMUSG00000032511	Scn5a	-1.237947125	0.030351309
ENSMUSG00000028614	Ndc1	-1.239584349	1.0218E-120
ENSMUSG00000041362	Shtn1	-1.241406214	5.21621E-08
ENSMUSG00000030654	Arl6ip1	-1.241729614	4.7797E-300
ENSMUSG00000010651	Acaa1b	-1.241752785	0.000826357
ENSMUSG00000032085	Tagln	-1.241779904	1.1325E-251
ENSMUSG00000042978	Sbk1	-1.24240531	3.49596E-12
ENSMUSG00000035208	Slfn8	-1.242535137	7.52661E-05
ENSMUSG00000083097	Gm14494	-1.242755314	0.000217442
ENSMUSG00000044702	Palb2	-1.243622972	1.47762E-08
ENSMUSG00000042873	Lhfpl4	-1.24626797	0.007732268
ENSMUSG00000035692	Isg15	-1.247302215	0.000640983
ENSMUSG00000026669	Mcm10	-1.247366711	4.36997E-30
ENSMUSG00000038764	Ptpn3	-1.248109773	4.25982E-24
ENSMUSG00000029096	Htra3	-1.249802083	3.93069E-19
ENSMUSG00000071984	Fndc1	-1.250291762	1.1292E-106
ENSMUSG00000035293	G2e3	-1.250352541	3.2596E-110
ENSMUSG00000019779	Frk	-1.250808591	2.05833E-42
ENSMUSG00000098318	Lockd	-1.25086346	2.2417E-05
ENSMUSG00000027454	Gins1	-1.250938956	1.69253E-13
ENSMUSG00000078762	Haus5	-1.251331183	1.67716E-17
ENSMUSG00000021714	Cenpk	-1.252004949	2.67717E-14
ENSMUSG00000020974	Pole2	-1.252221874	7.97436E-19
ENSMUSG00000012017	Scarf2	-1.252571594	1.87091E-94
ENSMUSG00000005583	Mef2c	-1.253130823	2.8598E-16
ENSMUSG00000052396	Mogat2	-1.253376033	3.2234E-06
ENSMUSG00000038173	Enpp6	-1.253744675	0.000324445
ENSMUSG00000038811	Gngt2	-1.254263374	0.042488455
ENSMUSG00000029366	Dck	-1.254495291	3.40985E-40
ENSMUSG00000023942	Slc29a1	-1.255949073	3.9857E-142
ENSMUSG00000021613	Hapln1	-1.25813062	8.0031E-05
ENSMUSG00000074896	Ifit3	-1.258471511	8.13134E-05
ENSMUSG00000108358	Gm44509	-1.261201354	7.69713E-05
ENSMUSG00000087368	BC065397	-1.261579553	0.00360911
ENSMUSG00000078956	Gm14221	-1.261641825	0.005414791
ENSMUSG00000039114	Nrn1	-1.261827632	1.64523E-24
ENSMUSG00000025150	Cbr2	-1.263151231	5.13467E-05
ENSMUSG00000034317	Trim59	-1.264271177	7.1268E-171
ENSMUSG00000022686	B3gnt5	-1.264844273	8.94515E-05

ENSMUSG00000029730	Mcm7	-1.26732203	3.3384E-100
ENSMUSG00000015083	C8g	-1.267668797	0.015606383
ENSMUSG00000028438	Kif24	-1.268712457	3.2727E-11
ENSMUSG00000021259	Cyp46a1	-1.268925094	0.012710953
ENSMUSG00000103865	Gm37416	-1.270103674	0.012477061
ENSMUSG00000030795	Fus	-1.270675105	0
ENSMUSG00000049130	C5ar1	-1.271065144	0.021930527
ENSMUSG00000032517	Mobp	-1.271246399	0.048618264
ENSMUSG00000055210	Foxd2	-1.271361631	0.036547178
ENSMUSG00000054196	Cthrc1	-1.27139419	1.16802E-33
ENSMUSG00000028698	Pik3r3	-1.27171379	6.64758E-77
ENSMUSG00000043885	Slc36a4	-1.27234497	8.0341E-141
ENSMUSG00000029149	Krtcap3	-1.272396746	0.005447094
ENSMUSG00000029561	Oasl2	-1.274057675	1.14889E-05
ENSMUSG00000038663	Fsd2	-1.274426571	0.030210054
ENSMUSG00000041126	H2az2	-1.277400374	5.07207E-50
ENSMUSG00000025001	Hells	-1.277468866	1.78259E-93
ENSMUSG00000026193	Fn1	-1.277498105	0
ENSMUSG00000036864	Proser3	-1.27753058	1.89961E-08
ENSMUSG00000100980	Gm29100	-1.277614386	0.015193425
ENSMUSG00000071847	Apcdd1	-1.278257172	8.82625E-33
ENSMUSG00000023084	Lrrc71	-1.278699861	0.046862604
ENSMUSG00000026228	Htr2b	-1.278849254	0.008457267
ENSMUSG00000035151	Elmod2	-1.278958198	1.67602E-50
ENSMUSG00000035929	H2-Q4	-1.278983435	3.8782E-33
ENSMUSG00000034522	Zfp395	-1.279351807	9.27167E-10
ENSMUSG00000030966	Trim21	-1.279809444	5.30369E-28
ENSMUSG00000031290	Lrch2	-1.280281065	7.29818E-35
ENSMUSG00000051037	Zfp455	-1.281946634	0.00030029
ENSMUSG00000045932	Ifit2	-1.285192244	4.7122E-12
ENSMUSG00000109408	A930037H05Rik	-1.286465328	0.000561353
ENSMUSG00000022021	Diaph3	-1.286908356	4.2018E-122
ENSMUSG00000035237	Lcat	-1.28691696	2.19546E-05
ENSMUSG00000043587	Pxylp1	-1.286974896	5.71653E-72
ENSMUSG00000062901	Klhl24	-1.287717354	1.817E-100
ENSMUSG00000030122	Ptms	-1.288356758	2.1625E-176
ENSMUSG00000075585	6330403L08Rik	-1.28993138	1.45448E-30
ENSMUSG00000048368	Omd	-1.290073678	0.000396954
ENSMUSG00000027246	Ell3	-1.290768795	0.028472064
ENSMUSG00000032221	Mns1	-1.290812953	4.17921E-33
ENSMUSG00000031951	Tmem231	-1.291683756	5.52823E-12
ENSMUSG00000040483	Xaf1	-1.293472979	2.22598E-25
ENSMUSG00000006585	Cdt1	-1.294402467	6.87746E-22
ENSMUSG00000021872	Rnase10	-1.298320391	0.007254123
ENSMUSG00000025240	Sacm1l	-1.298765943	3.1037E-225
ENSMUSG00000004952	Rasa4	-1.299376051	1.05691E-76
ENSMUSG00000090222	Ifi203-ps	-1.299641703	0.005644764
ENSMUSG00000030616	Sytl2	-1.300014575	2.7218E-26
ENSMUSG00000004127	Trmt10a	-1.300624753	8.17267E-74
ENSMUSG00000026355	Mcm6	-1.300716463	1.9589E-223
ENSMUSG00000026413	Pkp1	-1.303413103	2.03115E-78
ENSMUSG00000115149	9330188P03Rik	-1.303899333	8.22989E-28
ENSMUSG00000028933	Xrcc2	-1.305355003	1.16051E-18
ENSMUSG00000055296	Tmem245	-1.305548383	3.84865E-72
ENSMUSG00000049470	Aff4	-1.305988245	9.0781E-269
ENSMUSG00000074785	Plxnc1	-1.306506066	2.08444E-12
ENSMUSG00000019699	Akt3	-1.306631088	1.6974E-185
ENSMUSG00000027639	Samhd1	-1.30685718	8.69358E-82
ENSMUSG00000030921	Trim30a	-1.30805071	1.9906E-06
ENSMUSG00000072844	G530011O06Rik	-1.309039021	0.00039476
ENSMUSG00000043243	Fam129c	-1.309501518	0.000803018
ENSMUSG00000018761	Mpdu1	-1.309772	2.58144E-60
ENSMUSG00000005774	Rfx5	-1.310799701	5.86172E-35
ENSMUSG00000005045	Chd5	-1.31102932	0.047362525
ENSMUSG00000025915	Sgk3	-1.311599934	2.27075E-34
ENSMUSG00000025931	Paqr8	-1.313766784	9.89117E-29
ENSMUSG00000050107	Haspin	-1.313772371	9.73731E-25
ENSMUSG00000052565	H1f3	-1.3138338	5.20648E-06
ENSMUSG00000023908	Pkmyt1	-1.314641583	1.07001E-27
ENSMUSG00000038390	Gpr162	-1.317462685	5.62321E-08
ENSMUSG00000038917	3930402G23Rik	-1.317547311	0.015055878
ENSMUSG00000031398	Plxna3	-1.318177409	9.19935E-17
ENSMUSG00000096544	Gm4617	-1.31817944	7.76071E-05
ENSMUSG00000043079	Synpo	-1.318438356	8.0569E-166
ENSMUSG00000035042	Ccl5	-1.318750626	0.020070768
ENSMUSG00000074802	Gas2l3	-1.319857287	3.89601E-55
ENSMUSG00000042190	Cmklr1	-1.32068012	7.05137E-37

ENSMUSG00000089687	Rab42	-1.321279852	0.045322071
ENSMUSG00000054999	Naaladl1	-1.321291611	0.013830558
ENSMUSG00000024451	Arap3	-1.321981126	3.80331E-13
ENSMUSG00000068744	Psrc1	-1.323585615	2.9446E-115
ENSMUSG00000023909	Paqr4	-1.324890433	1.66054E-32
ENSMUSG00000061410	Zcchc14	-1.325572756	4.3695E-179
ENSMUSG00000025272	Tro	-1.325992992	8.13193E-19
ENSMUSG00000099032	Tcf24	-1.326555886	2.43507E-07
ENSMUSG00000085604	Dhx58os	-1.326650427	1.1071E-09
ENSMUSG00000097084	Foxl1	-1.327552945	0.000336183
ENSMUSG00000021815	Mss51	-1.328070434	0.000942244
ENSMUSG00000034906	Ncaph	-1.328478302	1.94354E-56
ENSMUSG00000023031	Cela1	-1.328650864	3.7055E-05
ENSMUSG00000056116	H2-T22	-1.329502363	4.56099E-74
ENSMUSG00000022053	Ebf2	-1.329882725	2.04325E-06
ENSMUSG00000007379	Dennd2c	-1.330674135	8.27522E-17
ENSMUSG00000037892	Pcdh18	-1.331149073	2.26962E-78
ENSMUSG00000037921	Ddx60	-1.331279843	3.86637E-11
ENSMUSG00000020681	Ace	-1.332269564	3.0565E-119
ENSMUSG00000039542	Ncam1	-1.333703676	1.61049E-66
ENSMUSG00000007656	Arpp19	-1.335071523	1.0247E-140
ENSMUSG00000051246	Msantd1	-1.335405901	0.005137923
ENSMUSG00000001493	Meox1	-1.335706608	2.56834E-48
ENSMUSG00000062609	Kcnj15	-1.337404662	1.6314E-75
ENSMUSG00000036867	Smad6	-1.338212762	2.7822E-253
ENSMUSG00000021803	Cdhr1	-1.339328849	2.92978E-07
ENSMUSG00000030711	Sult1a1	-1.341222316	0.038251614
ENSMUSG00000040195	Nemp1	-1.341782097	5.05007E-43
ENSMUSG00000017550	Atad5	-1.341993905	4.55493E-27
ENSMUSG00000027022	Xirp2	-1.343262876	2.54481E-07
ENSMUSG00000068245	Phf11d	-1.343515212	3.98181E-10
ENSMUSG00000001270	Ckb	-1.344760003	1.0833E-255
ENSMUSG00000019303	Psmc3ip	-1.345117491	5.75489E-11
ENSMUSG00000085389	1700003M07Rik	-1.345147384	0.022503884
ENSMUSG00000079429	Mroh2a	-1.345776236	1.57592E-12
ENSMUSG00000020827	Mink1	-1.345898616	4.4782E-80
ENSMUSG00000038168	P3h2	-1.345899897	5.88639E-41
ENSMUSG00000028399	Ptprd	-1.346187419	5.1814E-115
ENSMUSG00000021676	Iqgap2	-1.348450041	1.53004E-13
ENSMUSG00000034459	Ifit1	-1.349561198	8.47378E-06
ENSMUSG00000042988	Notum	-1.350838943	0.006054999
ENSMUSG00000078920	Ifi47	-1.352504878	1.54304E-86
ENSMUSG00000037474	Dtl	-1.35261457	6.85936E-32
ENSMUSG00000025395	Prim1	-1.354060141	4.36406E-44
ENSMUSG00000021097	Clmn	-1.355158725	0.000194751
ENSMUSG00000002265	Peg3	-1.358978851	6.48801E-32
ENSMUSG00000027496	Aurka	-1.361026438	8.99251E-64
ENSMUSG00000029334	Prkg2	-1.362059243	1.636E-34
ENSMUSG00000017861	Mybl2	-1.36390385	4.9147E-34
ENSMUSG00000097391	Mirg	-1.364224643	4.45481E-07
ENSMUSG00000056394	Lig1	-1.364566773	4.5545E-119
ENSMUSG00000038047	Haus6	-1.365099456	7.211E-99
ENSMUSG00000051832	E230016K23Rik	-1.365220361	0.00102773
ENSMUSG00000073422	H2-Ke6	-1.365433002	0.019239161
ENSMUSG00000048218	Amigo2	-1.368336277	9.74119E-09
ENSMUSG00000017639	Rab11fip4	-1.369553212	0.032792426
ENSMUSG00000045539	Sprr3	-1.369914259	0.002342362
ENSMUSG00000036986	Pml	-1.370873479	1.87682E-86
ENSMUSG00000019726	Lyst	-1.371062038	2.6875E-128
ENSMUSG00000022469	Rapgef3	-1.371939276	2.28E-77
ENSMUSG00000038070	Cntln	-1.372759367	4.95388E-42
ENSMUSG00000115718	Gm49179	-1.373068346	0.001060839
ENSMUSG00000070034	Sp110	-1.374895416	9.63452E-25
ENSMUSG00000035403	Crb2	-1.37498607	0.000241271
ENSMUSG00000062826	Ces2f	-1.375381174	0.001600847
ENSMUSG00000115232	Gm49378	-1.376560746	0.009055513
ENSMUSG00000026064	Ptp4a1	-1.376858124	5.65106E-58
ENSMUSG00000108957	Gm45235	-1.378152418	0.006171758
ENSMUSG00000040918	Slc19a2	-1.378768708	5.2076E-224
ENSMUSG00000005950	P2rx5	-1.380484653	2.97163E-26
ENSMUSG00000001227	Sema6b	-1.380746349	5.91331E-16
ENSMUSG00000041449	Serpina3h	-1.381057068	1.84765E-41
ENSMUSG00000042043	Tbca	-1.38237562	1.724E-100
ENSMUSG00000096472	Cdkn2d	-1.386230645	2.23385E-31
ENSMUSG00000038418	Egr1	-1.386428165	1.1152E-114
ENSMUSG00000039748	Exo1	-1.388736424	4.70494E-26
ENSMUSG00000036882	Arhgap33	-1.38979788	9.6251E-06

ENSMUSG00000055240	Zfp101	-1.391108792	1.85069E-25
ENSMUSG00000020357	Flt4	-1.391450023	2.0631E-128
ENSMUSG00000020330	Hmmr	-1.392340681	1.5451E-141
ENSMUSG00000004473	Clec11a	-1.393632635	2.46531E-16
ENSMUSG00000042213	Zfand4	-1.393667729	0.000338779
ENSMUSG00000029648	Flt1	-1.394306294	8.2612E-114
ENSMUSG00000028873	Cdca8	-1.394478574	1.65225E-80
ENSMUSG00000062309	Rpp25	-1.395011445	4.11273E-06
ENSMUSG00000073733	Cplane2	-1.395569789	0.002298434
ENSMUSG00000049281	Scn3b	-1.398247592	7.9262E-05
ENSMUSG00000020086	Macroh2a2	-1.398570893	1.52241E-38
ENSMUSG00000040372	Gpr63	-1.400091728	6.87835E-11
ENSMUSG00000033411	Ctdspl2	-1.400320352	5.03226E-72
ENSMUSG00000059674	Cdh24	-1.400365529	8.41762E-06
ENSMUSG00000027329	Spef1	-1.400567129	1.56586E-35
ENSMUSG00000032113	Chek1	-1.400735297	2.65122E-39
ENSMUSG00000004633	Chn2	-1.400869947	1.98792E-15
ENSMUSG00000004085	Map3k20	-1.401210491	1.5764E-195
ENSMUSG00000021464	Ror2	-1.401444824	4.53193E-32
ENSMUSG00000109783	Gm45338	-1.401973357	0.001514186
ENSMUSG00000005958	Ephb3	-1.402337314	2.62665E-16
ENSMUSG00000026605	Cenpf	-1.403601717	1.671E-176
ENSMUSG00000056579	Tug1	-1.404223122	0
ENSMUSG00000022150	Dab2	-1.404649757	0
ENSMUSG00000036395	Glb1l2	-1.404700575	0.012408343
ENSMUSG00000021965	Ska3	-1.406070114	1.23453E-50
ENSMUSG00000037991	Rmi2	-1.406132204	0.019303105
ENSMUSG00000021990	Spata13	-1.407198946	1.72648E-33
ENSMUSG00000081769	Gm12216	-1.409458586	0.024947637
ENSMUSG00000105135	Gm43667	-1.410591727	0.00084657
ENSMUSG00000022220	Adcy4	-1.410812506	3.36417E-62
ENSMUSG00000025789	St8sia2	-1.410939019	1.59662E-05
ENSMUSG00000097467	Gm26737	-1.411405119	7.37288E-15
ENSMUSG00000038319	Kcnh2	-1.412048691	8.53632E-14
ENSMUSG00000037313	Tacc3	-1.412106153	2.3728E-181
ENSMUSG00000027018	Hat1	-1.413270603	6.9695E-162
ENSMUSG00000022766	Serpind1	-1.413510555	6.72174E-24
ENSMUSG00000007080	Pole	-1.413917877	7.50978E-88
ENSMUSG00000021130	Galnt16	-1.414248526	1.4203E-05
ENSMUSG00000042489	Clspn	-1.415622436	1.19044E-61
ENSMUSG00000014361	Mertk	-1.416041453	4.0667E-195
ENSMUSG00000020926	Adam11	-1.416154528	0.023174222
ENSMUSG00000043051	Disc1	-1.417328542	0.000518143
ENSMUSG00000104713	Gbp6	-1.417585905	1.9374E-16
ENSMUSG00000070047	Fat1	-1.417711834	0
ENSMUSG00000031016	Wee1	-1.418268475	4.95805E-70
ENSMUSG00000016496	Cd274	-1.418298351	8.61496E-12
ENSMUSG00000037466	Tedc1	-1.419720968	8.80703E-37
ENSMUSG00000028175	Depdc1a	-1.420266364	4.02031E-85
ENSMUSG00000062257	Opcml	-1.421366769	0.005803058
ENSMUSG00000030255	Sspn	-1.421905816	1.40204E-39
ENSMUSG00000024660	Incenp	-1.422034425	1.2536E-138
ENSMUSG00000051113	Fam71e1	-1.423858508	0.036706023
ENSMUSG00000049758	Olfr1318	-1.425018002	0.001808719
ENSMUSG00000040447	Spns2	-1.427430205	1.06494E-21
ENSMUSG00000005677	Nr1i3	-1.42895525	0.038492366
ENSMUSG00000097789	Gm2115	-1.429026222	2.73986E-11
ENSMUSG00000031262	Cenpi	-1.429069774	8.58213E-52
ENSMUSG00000005470	Asf1b	-1.431269656	5.2865E-58
ENSMUSG00000020381	Mrnip	-1.431507097	0.011060781
ENSMUSG00000045767	B230219D22Rik	-1.431667883	0
ENSMUSG00000093661	Eif4e3	-1.431731393	1.84862E-18
ENSMUSG00000004267	Eno2	-1.432798752	4.93358E-25
ENSMUSG00000001029	Icam2	-1.432906191	1.08763E-09
ENSMUSG00000024039	Cbs	-1.434058528	0.0012617
ENSMUSG00000102577	Gm37969	-1.434385007	0.000384922
ENSMUSG00000035095	Fam167a	-1.434524945	2.65389E-07
ENSMUSG00000033762	Recql4	-1.434623678	4.64817E-10
ENSMUSG00000028555	Ttc39a	-1.435907106	8.44315E-08
ENSMUSG00000107017	Gm43196	-1.436554305	0.020144687
ENSMUSG00000025574	Tk1	-1.436658146	1.35688E-98
ENSMUSG00000006930	Hap1	-1.43689432	3.88136E-12
ENSMUSG00000020182	Ddc	-1.43708102	4.02529E-05
ENSMUSG00000112941	Gm48623	-1.437140955	3.40074E-07
ENSMUSG00000025104	Hdgfl3	-1.437286602	1.71963E-93
ENSMUSG00000057337	Chst3	-1.437473719	0.01072481
ENSMUSG00000028832	Stmn1	-1.43798878	4.0884E-142

ENSMUSG00000032841	Prr5l	-1.438133886	1.23437E-50
ENSMUSG00000049357	Brd8dc	-1.438464884	0.000866716
ENSMUSG00000097908	4933404O12Rik	-1.43853974	6.09784E-08
ENSMUSG00000009575	Cbx5	-1.438917361	4.3385E-293
ENSMUSG00000029822	Osbpl3	-1.43917242	3.51133E-64
ENSMUSG00000050671	Ism2	-1.439318015	0.002870135
ENSMUSG00000034329	Brip1	-1.439557848	8.80876E-31
ENSMUSG00000027598	Itch	-1.440592754	1.2323E-196
ENSMUSG00000040328	Olfr56	-1.443071704	3.936E-16
ENSMUSG00000044927	H1f10	-1.443508159	4.2638E-27
ENSMUSG00000056531	Ccdc18	-1.443652248	0.000152454
ENSMUSG00000036040	Adamtsl2	-1.443720201	5.52225E-11
ENSMUSG00000046179	E2f8	-1.44378939	1.85703E-36
ENSMUSG00000084842	Pabpc1l2b-ps	-1.444749841	0.004989264
ENSMUSG00000106864	Gtf3c2	-1.445304127	2.3788E-193
ENSMUSG00000038644	Pold1	-1.446046161	1.74621E-61
ENSMUSG00000020032	Nuak1	-1.44707888	7.9049E-182
ENSMUSG00000017716	Birc5	-1.44738171	2.46596E-87
ENSMUSG00000029638	Glcci1	-1.447708584	3.08631E-28
ENSMUSG00000054206	Gzmm	-1.447725973	0.013569593
ENSMUSG00000028312	Smc2	-1.448988922	1.1628E-302
ENSMUSG00000019326	Aoc3	-1.449396347	2.8388E-06
ENSMUSG00000043008	Klhl6	-1.44965846	6.9862E-177
ENSMUSG00000040204	Pclaf	-1.450819868	1.31207E-68
ENSMUSG00000111681	Gm47640	-1.450850618	3.45423E-10
ENSMUSG00000029798	Herc6	-1.451642025	1.44473E-35
ENSMUSG00000051537	Gm5124	-1.452616549	0.005870899
ENSMUSG00000021485	Mxd3	-1.45271071	3.23185E-21
ENSMUSG00000005410	Mcm5	-1.45454149	3.4003E-153
ENSMUSG00000022546	Gpt	-1.456725601	2.96145E-07
ENSMUSG00000086524	Pabpc1l2a-ps	-1.457617236	0.008952628
ENSMUSG00000079017	Ifi27l2a	-1.459666918	9.86144E-21
ENSMUSG00000026039	Sgo2a	-1.459680442	9.61434E-87
ENSMUSG00000072980	Oip5	-1.461353688	3.83683E-18
ENSMUSG00000044362	Ccdc89	-1.461671185	0.000316115
ENSMUSG00000027663	Zmat3	-1.462019429	0
ENSMUSG00000074994	Qser1	-1.462212879	1.4571E-194
ENSMUSG00000117285	Gm49863	-1.462374468	0.025340553
ENSMUSG00000074476	Spc24	-1.462718925	3.32062E-39
ENSMUSG00000096937	Gm10825	-1.465225132	0.045127716
ENSMUSG00000051323	Pcdh19	-1.465515083	1.5814E-110
ENSMUSG00000039697	Ncoa7	-1.465947041	5.61838E-38
ENSMUSG00000054150	Syne3	-1.46623668	4.27298E-06
ENSMUSG00000030528	Blm	-1.466797894	2.56756E-37
ENSMUSG00000028919	Arhgef19	-1.467201552	2.50586E-22
ENSMUSG00000001349	Cnn1	-1.467751747	0.000651689
ENSMUSG00000034773	Hrob	-1.467860583	3.24461E-21
ENSMUSG00000010601	Apol7a	-1.468289462	0.000734959
ENSMUSG00000032667	Pon2	-1.468523166	4.5736E-227
ENSMUSG00000039735	Fnbp1l	-1.468540648	8.29168E-42
ENSMUSG00000003934	Efnb3	-1.469039292	0.041057757
ENSMUSG00000030346	Rad51ap1	-1.470294014	7.54251E-33
ENSMUSG00000027323	Rad51	-1.470422498	3.09949E-74
ENSMUSG00000034349	Smc4	-1.470588522	4.427E-293
ENSMUSG00000029843	Slc13a4	-1.472155028	0.025881647
ENSMUSG00000042807	Hecw2	-1.472282764	3.85024E-72
ENSMUSG00000019942	Cdk1	-1.472519467	1.1471E-260
ENSMUSG00000048960	Prex2	-1.472751663	0
ENSMUSG00000020099	Unc5b	-1.473072785	0
ENSMUSG00000044201	Cdc25c	-1.473642472	7.19925E-33
ENSMUSG00000038252	Ncapd2	-1.474329146	5.2647E-185
ENSMUSG00000071317	Bves	-1.475852604	0.029225006
ENSMUSG00000041431	Ccnb1	-1.477433382	3.4555E-247
ENSMUSG00000022033	Pbk	-1.478221342	3.07026E-86
ENSMUSG00000085873	Ttc39aos1	-1.479337069	0.045101686
ENSMUSG00000067215	Usp51	-1.481797219	0.020449012
ENSMUSG00000035493	Tgfbi	-1.481882357	5.5658E-123
ENSMUSG00000032400	Zwilch	-1.482226991	9.94315E-94
ENSMUSG00000022422	Dscc1	-1.483250837	3.96226E-05
ENSMUSG00000035778	Ggta1	-1.483316195	1.0663E-212
ENSMUSG00000031849	Comp	-1.483417784	6.94453E-05
ENSMUSG00000000028	Cdc45	-1.48401573	5.01322E-40
ENSMUSG00000040543	Pitpnm3	-1.484184888	0.048269767
ENSMUSG00000089769	Gm16574	-1.484691247	0.001964572
ENSMUSG00000030600	Lrfn1	-1.485146608	0.001607283
ENSMUSG00000015243	Abca1	-1.485352164	0
ENSMUSG00000004099	Dnmt1	-1.486054492	2.122E-199

ENSMUSG00000046561	Arsj	-1.486265778	9.42932E-43
ENSMUSG00000001249	Hpn	-1.486930312	0.000306406
ENSMUSG00000031385	Plxnb3	-1.48732486	0.028579745
ENSMUSG00000051235	Gen1	-1.48834485	5.15472E-42
ENSMUSG00000023919	Cenpq	-1.489833857	1.90197E-57
ENSMUSG00000042106	Inka1	-1.490791546	2.4023E-21
ENSMUSG00000107928	Gm45140	-1.492348051	0.000726699
ENSMUSG00000022034	Esco2	-1.49291864	3.4439E-31
ENSMUSG00000056174	Col8a2	-1.495679914	0.043838618
ENSMUSG00000039109	F13a1	-1.497299893	0.000212874
ENSMUSG00000053004	Hrh1	-1.498415749	0.001159877
ENSMUSG00000005233	Spc25	-1.498470993	6.1219E-104
ENSMUSG00000111229	Gm39323	-1.499407302	0.015715064
ENSMUSG00000052949	Rnf157	-1.499512958	3.74148E-32
ENSMUSG00000021286	Zfyve21	-1.49955401	5.42815E-38
ENSMUSG00000030677	Kif22	-1.500873956	6.9355E-109
ENSMUSG00000027635	Dsn1	-1.501768868	1.07482E-26
ENSMUSG00000007107	Atp1a4	-1.501942578	0.006991344
ENSMUSG00000032224	Fam81a	-1.502450768	0.002000085
ENSMUSG00000038180	Spag4	-1.502528453	5.62698E-10
ENSMUSG00000004540	Psg17	-1.502996475	0.003476546
ENSMUSG00000100975	Gm28875	-1.503032465	5.83801E-11
ENSMUSG00000091568	Gm8206	-1.503534656	0.010165833
ENSMUSG00000027075	Slc43a1	-1.503891783	2.59232E-07
ENSMUSG00000021265	Slc25a29	-1.50389694	6.51627E-06
ENSMUSG00000054717	Hmgb2	-1.504219474	3.5418E-191
ENSMUSG00000031543	Ank1	-1.504519254	1.79878E-38
ENSMUSG00000034040	Galnt17	-1.50483411	2.03654E-50
ENSMUSG00000097755	2010110K18Rik	-1.507414157	0.02920028
ENSMUSG00000108528	Gm8319	-1.508017909	0.041539698
ENSMUSG00000024472	Dcp2	-1.508066655	1.707E-142
ENSMUSG00000037628	Cdkn3	-1.508111596	3.52102E-45
ENSMUSG00000006678	Pola1	-1.512860506	2.0824E-105
ENSMUSG00000086421	Gm14091	-1.513505756	0.002682079
ENSMUSG00000028228	Cpne3	-1.513795801	3.6576E-249
ENSMUSG00000028687	Mutyh	-1.513811475	0.000575295
ENSMUSG00000037375	Hhat	-1.514755618	3.1102E-24
ENSMUSG00000035045	Zc3h12b	-1.515055863	5.90083E-21
ENSMUSG00000004105	Angptl2	-1.515275557	0
ENSMUSG00000020053	Igf1	-1.516800598	6.1392E-129
ENSMUSG00000055805	Fmnl1	-1.517078572	9.88159E-10
ENSMUSG00000024136	Dnase1l2	-1.517770045	0.004546252
ENSMUSG00000027641	Rbl1	-1.518688787	1.51481E-88
ENSMUSG00000053693	Mast1	-1.51885599	0.042295225
ENSMUSG00000111312	Gm47205	-1.519385321	0.002564421
ENSMUSG00000028068	Iqgap3	-1.519414679	1.6094E-123
ENSMUSG00000087060	Eldr	-1.519500915	6.02333E-08
ENSMUSG00000028444	Cntfr	-1.519554531	8.46974E-08
ENSMUSG00000026831	1700007K13Rik	-1.520888917	3.37783E-11
ENSMUSG00000027160	Ccdc34	-1.521584045	2.19482E-42
ENSMUSG00000024056	Ndc80	-1.523085696	2.18586E-85
ENSMUSG00000029910	Mad2l1	-1.523130853	1.2365E-146
ENSMUSG00000073434	Wdr90	-1.523850571	2.7448E-41
ENSMUSG00000079283	2310009B15Rik	-1.524235499	3.24546E-17
ENSMUSG00000024168	Tmem204	-1.524325518	9.97485E-39
ENSMUSG00000017499	Cdc6	-1.527155965	5.84624E-42
ENSMUSG00000046807	Lrrc75b	-1.528774891	5.67264E-13
ENSMUSG00000047746	Fbxo40	-1.529354078	0.000460842
ENSMUSG00000020151	Ptprr	-1.529513948	0.015369343
ENSMUSG00000024989	Cep55	-1.530208085	7.6933E-131
ENSMUSG00000022836	Mylk	-1.532681259	1.36839E-25
ENSMUSG00000037166	Ppp1r14a	-1.534871249	2.01276E-13
ENSMUSG00000021594	Srd5a1	-1.535268159	7.2616E-07
ENSMUSG00000035142	Nubpl	-1.53539222	2.17163E-16
ENSMUSG00000024134	Six2	-1.535796976	1.51887E-17
ENSMUSG00000037572	Wdhd1	-1.536279386	4.5551E-38
ENSMUSG00000095115	Itpripl2	-1.536883518	0
ENSMUSG00000023940	Sgo1	-1.537779166	3.78925E-57
ENSMUSG00000039994	Timeless	-1.538138881	3.23712E-37
ENSMUSG00000026484	Rnf2	-1.538366699	4.028E-120
ENSMUSG00000032291	Crabp1	-1.539266704	0.000156516
ENSMUSG00000001228	Uhrf1	-1.540230981	3.12573E-84
ENSMUSG00000023505	Cdca3	-1.540326997	1.48426E-97
ENSMUSG00000033597	Caskin1	-1.541263912	0.003393663
ENSMUSG00000037725	Ckap2	-1.541965146	8.2859E-192
ENSMUSG00000079362	Gm43302	-1.544587923	6.64234E-11
ENSMUSG00000051331	Cacna1c	-1.544886574	8.09335E-68

ENSMUSG00000071862	Lrrtm2	-1.545864521	7.66011E-05
ENSMUSG00000038552	Fndc4	-1.546036207	5.00584E-12
ENSMUSG00000109086	Gm5596	-1.547539964	0.024221308
ENSMUSG00000024811	Tnks2	-1.549202501	0
ENSMUSG00000046480	Scn4b	-1.552764849	0.010468496
ENSMUSG00000031965	Tbx20	-1.552939684	0
ENSMUSG00000027306	Nusap1	-1.554894128	1.4452E-128
ENSMUSG00000021572	Cep72	-1.556837867	1.73182E-17
ENSMUSG00000033632	AW554918	-1.557229882	7.60404E-11
ENSMUSG00000024210	Ip6k3	-1.558607023	0.003372309
ENSMUSG00000030830	Itgal	-1.560373943	0.009638103
ENSMUSG00000021373	Cap2	-1.560379979	6.18036E-24
ENSMUSG00000020836	Coro6	-1.56079676	4.17883E-05
ENSMUSG00000048922	Cdca2	-1.561839561	2.31583E-79
ENSMUSG00000053414	Hunk	-1.562859404	0.000367437
ENSMUSG00000038608	Dock10	-1.563114466	1.7394E-68
ENSMUSG00000058290	Espl1	-1.563187981	9.2886E-101
ENSMUSG00000030042	Pole4	-1.563662619	7.8279E-106
ENSMUSG00000039396	Neil3	-1.563763856	5.55696E-33
ENSMUSG00000047692	4930533K18Rik	-1.563965483	0.014838541
ENSMUSG00000097961	Gm27000	-1.564772765	5.42415E-08
ENSMUSG00000027379	Bub1	-1.565654086	5.448E-155
ENSMUSG00000036030	Prtg	-1.566164505	0.016394474
ENSMUSG00000035683	Melk	-1.567680579	1.84895E-77
ENSMUSG00000021903	Galnt15	-1.568889788	0.027540723
ENSMUSG00000072082	Ccnf	-1.571078247	7.2988E-118
ENSMUSG00000042029	Ncapg2	-1.571261838	6.7863E-163
ENSMUSG00000029101	Rgs12	-1.57237736	2.4805E-210
ENSMUSG00000015452	Ager	-1.575988248	0.002472288
ENSMUSG00000112980	D430020J02Rik	-1.576261698	8.15381E-26
ENSMUSG00000058743	Kcnj14	-1.576403758	0.019084232
ENSMUSG00000046449	Nexmif	-1.578423258	7.80966E-19
ENSMUSG00000082163	Gm14276	-1.578586726	3.53846E-05
ENSMUSG00000000049	Apoh	-1.579819422	0.000930636
ENSMUSG00000085399	Foxd2os	-1.580787555	3.91542E-17
ENSMUSG00000075592	Nynrin	-1.581280309	1.94498E-86
ENSMUSG00000027309	4930402H24Rik	-1.581444758	1.0174E-271
ENSMUSG00000029605	Oas1b	-1.58526996	7.34527E-15
ENSMUSG00000034413	Neurl1b	-1.586283491	7.32054E-17
ENSMUSG00000058589	Anks1b	-1.58858098	4.8337E-14
ENSMUSG00000033610	Pank1	-1.588679125	6.15711E-28
ENSMUSG00000073144	4930599N23Rik	-1.589873164	0.011750681
ENSMUSG00000006362	Cbfa2t3	-1.590585226	0.035975851
ENSMUSG00000049493	Pls1	-1.592384075	1.35734E-05
ENSMUSG00000097451	Rian	-1.592496094	2.972E-184
ENSMUSG00000026960	Arl6ip6	-1.592937564	6.92497E-67
ENSMUSG00000086213	A330040F15Rik	-1.593922727	0.023204742
ENSMUSG00000043110	Lrrn4	-1.594383327	6.23133E-90
ENSMUSG00000004655	Aqp1	-1.594410146	4.4368E-223
ENSMUSG00000081052	Gm6305	-1.5949786	0.029438539
ENSMUSG00000029121	Crmp1	-1.595067671	1.43102E-09
ENSMUSG00000014164	Klhl3	-1.595247661	6.78435E-07
ENSMUSG00000051220	Ercc6l	-1.599751241	4.76147E-53
ENSMUSG00000078853	Igtp	-1.600149286	1.85011E-07
ENSMUSG00000106062	Gm43820	-1.600517791	0.022053303
ENSMUSG00000028702	Rad54l	-1.600852337	6.03531E-35
ENSMUSG00000003038	Hmgn2	-1.6015621	0
ENSMUSG00000030107	Usp18	-1.601772019	8.99191E-07
ENSMUSG00000032500	Dclk3	-1.60348674	1.36164E-06
ENSMUSG00000013663	Pten	-1.603508642	0
ENSMUSG00000090942	F830016B08Rik	-1.604283119	5.78553E-13
ENSMUSG00000048458	Inka2	-1.60715901	3.00925E-79
ENSMUSG00000022421	Nptxr	-1.60738589	1.39193E-16
ENSMUSG00000021540	Smad5	-1.608358238	7.9976E-199
ENSMUSG00000021782	Dlg5	-1.608493936	3.4516E-265
ENSMUSG00000024791	Cdca5	-1.60884455	1.43234E-44
ENSMUSG00000024330	Col11a2	-1.610287396	0.001293976
ENSMUSG00000005611	Mrvi1	-1.611061516	8.30531E-15
ENSMUSG00000071658	Gng3	-1.613026872	0.019640976
ENSMUSG00000019773	Fbxo5	-1.613750523	9.38523E-47
ENSMUSG00000042251	Pm20d1	-1.615138985	1.40073E-10
ENSMUSG00000041670	Rims1	-1.616173173	0.018438308
ENSMUSG00000032586	Traip	-1.617346388	1.41819E-31
ENSMUSG00000022322	Shcbp1	-1.617860042	4.17032E-85
ENSMUSG00000050315	Synpo2	-1.617992311	1.99826E-82
ENSMUSG00000033350	Chst2	-1.618756747	1.40405E-31
ENSMUSG00000053310	Nrgn	-1.618778751	0.03283629

ENSMUSG00000023019	Gpd1	-1.620284263	0.000783463
ENSMUSG00000042793	Lgr6	-1.620477167	1.16793E-54
ENSMUSG00000043789	Vwce	-1.621682325	0.033215903
ENSMUSG00000057897	Camk2b	-1.623200074	4.30545E-08
ENSMUSG00000027330	Cdc25b	-1.62323062	2.27878E-98
ENSMUSG00000025701	Alox5	-1.625845092	6.73709E-07
ENSMUSG00000026414	Tnnt2	-1.628295352	4.3482E-162
ENSMUSG00000038486	Sv2a	-1.629067587	3.71059E-10
ENSMUSG00000087291	Gm11946	-1.629959647	0.000190725
ENSMUSG00000004891	Nes	-1.631481265	0
ENSMUSG00000042428	Mgat3	-1.631957284	9.52021E-14
ENSMUSG00000001517	Foxm1	-1.635709742	8.7155E-225
ENSMUSG00000040424	Hipk4	-1.637454137	0.000514938
ENSMUSG00000040852	Plekhh2	-1.638664866	2.2884E-194
ENSMUSG00000029570	Lfng	-1.638964447	1.11948E-09
ENSMUSG00000031327	Chic1	-1.639715158	4.17513E-34
ENSMUSG00000030216	Wbp11	-1.640243622	4.5406E-253
ENSMUSG00000022537	Tmem44	-1.641278936	7.37244E-08
ENSMUSG00000028132	Tlcd4	-1.646085042	2.05739E-24
ENSMUSG00000035455	Fignl1	-1.647080115	9.5514E-63
ENSMUSG00000034024	Cct2	-1.648292353	0
ENSMUSG00000013367	Iglon5	-1.649147571	0.000688209
ENSMUSG00000072919	Noxred1	-1.652474873	0.012282268
ENSMUSG00000086942	Gm15489	-1.652602188	0.043211652
ENSMUSG00000034883	Lrr1	-1.653017167	4.784E-09
ENSMUSG00000034311	Kif4	-1.653212909	2.7968E-145
ENSMUSG00000030031	Kbtbd8	-1.653462636	4.9192E-12
ENSMUSG00000081972	Gm16397	-1.653805729	0.009505793
ENSMUSG00000027861	Casq2	-1.653999201	0.005374759
ENSMUSG00000028445	Enho	-1.655011976	1.6708E-07
ENSMUSG00000000247	Lhx2	-1.655865571	0.029172882
ENSMUSG00000029923	Rab19	-1.657781202	0.013274526
ENSMUSG00000046314	Stxbp6	-1.659801775	4.32385E-09
ENSMUSG00000035189	Ano4	-1.660636205	0.000174548
ENSMUSG00000022676	Snai2	-1.661352111	1.3265E-56
ENSMUSG00000049866	Arl4c	-1.661990336	5.4112E-101
ENSMUSG00000008496	Pou2f2	-1.663118518	2.01696E-07
ENSMUSG00000085042	Abhd11os	-1.663144661	0.007629926
ENSMUSG00000063060	Sox7	-1.663171902	2.81989E-28
ENSMUSG00000020900	Myh10	-1.664103702	0
ENSMUSG00000020057	Dram1	-1.664744115	1.5856E-135
ENSMUSG00000010476	Ebf3	-1.66625877	4.63323E-58
ENSMUSG00000029177	Cenpa	-1.668056204	1.0283E-173
ENSMUSG00000070425	Xntrpc	-1.668293653	0.000209581
ENSMUSG00000012443	Kif11	-1.668379253	2.6282E-247
ENSMUSG00000027115	Kif18a	-1.670598254	7.02234E-62
ENSMUSG00000027699	Ect2	-1.670733682	4.0354E-149
ENSMUSG00000030641	Ddias	-1.674130726	3.4704E-139
ENSMUSG00000049036	Tmem121	-1.675003798	2.31743E-15
ENSMUSG00000036902	Neto2	-1.679106298	1.79689E-14
ENSMUSG00000032596	Uba7	-1.679677941	3.65187E-16
ENSMUSG00000054115	Skp2	-1.681827071	2.09196E-75
ENSMUSG00000018983	E2f2	-1.68367311	2.85118E-14
ENSMUSG00000021379	Id4	-1.685633164	4.5546E-22
ENSMUSG00000040605	Bace2	-1.686841394	1.31579E-62
ENSMUSG00000009376	Met	-1.688154794	1.60034E-77
ENSMUSG00000075304	Sp5	-1.688961809	0.000291284
ENSMUSG00000026779	Mastl	-1.689738127	5.2676E-102
ENSMUSG00000071648	Rom1	-1.690035914	7.60148E-28
ENSMUSG00000038602	Slc35f1	-1.690429365	4.16205E-20
ENSMUSG00000090272	Mndal	-1.690648431	7.75286E-24
ENSMUSG00000041219	Arhgap11a	-1.691734126	4.8893E-211
ENSMUSG00000067818	Myl9	-1.691781774	2.2509E-122
ENSMUSG00000059323	Tonsl	-1.693954246	5.51101E-30
ENSMUSG00000020914	Top2a	-1.694833782	0
ENSMUSG00000029093	Sorcs2	-1.694833912	4.12045E-08
ENSMUSG00000031712	Il15	-1.694855503	3.32764E-11
ENSMUSG00000050505	Pcdh20	-1.695091181	0.000326507
ENSMUSG00000027253	Lrp4	-1.696443728	3.91558E-24
ENSMUSG00000027353	Mcm8	-1.696811775	6.10697E-29
ENSMUSG00000026582	Sele	-1.698157989	2.4419E-270
ENSMUSG00000091144	Phf11c	-1.699551793	1.82525E-10
ENSMUSG00000027469	Tpx2	-1.700765905	1.47E-232
ENSMUSG00000019961	Tmpo	-1.70386514	0
ENSMUSG00000070407	Hs3st3b1	-1.70462943	3.2088E-16
ENSMUSG00000038569	Rad9b	-1.704897655	0.008052347
ENSMUSG00000086477	Gm15506	-1.705051272	0.003546452

ENSMUSG00000105695	Gm43327	-1.707587364	0.000333966
ENSMUSG00000046634	Pkd1l1	-1.709056589	1.3074E-05
ENSMUSG00000020251	Glt8d2	-1.70993982	5.06837E-98
ENSMUSG00000036768	Kif15	-1.711245871	9.08163E-82
ENSMUSG00000108049	Gm44168	-1.7130498	0.003040966
ENSMUSG00000062393	Dgkk	-1.713271192	0.01294359
ENSMUSG00000052516	Robo2	-1.714770485	1.08726E-13
ENSMUSG00000024049	Myom1	-1.71492379	1.28166E-08
ENSMUSG00000026622	Nek2	-1.714955185	1.2193E-211
ENSMUSG00000020785	Camkk1	-1.716524845	8.78361E-26
ENSMUSG00000048534	Jaml	-1.716828261	2.3764E-08
ENSMUSG00000021798	Ldb3	-1.71702114	1.03505E-09
ENSMUSG00000033355	Rtp4	-1.717092283	2.40639E-11
ENSMUSG00000027715	Ccna2	-1.717212939	0
ENSMUSG00000041406	BC055324	-1.717262431	3.97059E-41
ENSMUSG00000041515	Irf8	-1.717518648	1.32598E-25
ENSMUSG00000044313	Mab21l3	-1.717768041	4.83537E-13
ENSMUSG00000027977	Ndst3	-1.718755511	1.12344E-17
ENSMUSG00000024534	Sncaip	-1.718902709	3.33823E-07
ENSMUSG00000027654	Fam83d	-1.724199498	1.1219E-104
ENSMUSG00000045287	Rtn4rl1	-1.728040308	5.56566E-10
ENSMUSG00000030541	Idh2	-1.730339804	1.7435E-201
ENSMUSG00000041757	Plekha6	-1.732374228	1.48242E-51
ENSMUSG00000034023	Fancd2	-1.732980829	1.01835E-32
ENSMUSG00000001403	Ube2c	-1.73458453	3.1098E-159
ENSMUSG00000109367	Gm18782	-1.737387116	0.044468193
ENSMUSG00000021822	Plau	-1.737676731	0
ENSMUSG00000109452	Gm44530	-1.738528939	0.033666003
ENSMUSG00000098090	2700099C18Rik	-1.739568426	7.47695E-23
ENSMUSG00000063193	Cd300lb	-1.74177432	1.43369E-25
ENSMUSG00000036223	Ska1	-1.744202591	4.40095E-19
ENSMUSG00000031137	Fgf13	-1.74457288	1.28623E-14
ENSMUSG00000091649	Phf11b	-1.74506101	7.40315E-10
ENSMUSG00000067780	Pi15	-1.747477736	0
ENSMUSG00000063354	Slc39a4	-1.747487918	2.5249E-11
ENSMUSG00000028011	Tdo2	-1.749134621	0.035451212
ENSMUSG00000032946	Rasgrp2	-1.753713182	4.58565E-10
ENSMUSG00000071042	Rasgrp3	-1.756233636	6.62435E-49
ENSMUSG00000000223	Drp2	-1.75724028	2.34316E-16
ENSMUSG00000031099	Smarca1	-1.757584009	1.91856E-16
ENSMUSG00000020897	Aurkb	-1.758569187	5.9063E-100
ENSMUSG00000046295	Ankle1	-1.758652759	2.63329E-22
ENSMUSG00000015880	Ncapg	-1.759056318	1.4493E-115
ENSMUSG00000036264	Fstl4	-1.760109795	1.90552E-06
ENSMUSG00000097519	4930558J18Rik	-1.763564082	0.010707035
ENSMUSG00000019845	Tube1	-1.765578552	3.69135E-13
ENSMUSG00000050541	Adra1b	-1.769993243	1.21989E-09
ENSMUSG00000046341	Gm11223	-1.771891226	1.09707E-07
ENSMUSG00000028678	Kif2c	-1.772898367	2.3091E-108
ENSMUSG00000067321	Gm7931	-1.773932112	0.011996686
ENSMUSG00000047844	Bex4	-1.773972637	0.007658369
ENSMUSG00000030867	Plk1	-1.778431869	1.5356E-248
ENSMUSG00000032218	Ccnb2	-1.779318682	7.4942E-258
ENSMUSG00000021268	Meg3	-1.779389266	4.6203E-109
ENSMUSG00000025742	Prps2	-1.781306404	7.0035E-217
ENSMUSG00000031629	Cenpu	-1.783796071	1.24521E-20
ENSMUSG00000006398	Cdc20	-1.784159934	0
ENSMUSG00000066363	Serpina3f	-1.784790748	1.1196E-191
ENSMUSG00000024795	Kif20b	-1.791142701	1.7325E-110
ENSMUSG00000005268	Prlr	-1.7912623	0.003185429
ENSMUSG00000048327	Ckap2l	-1.793718364	8.9167E-151
ENSMUSG00000040723	Rcsd1	-1.794295781	5.0167E-191
ENSMUSG00000026955	Sapcd2	-1.794445276	2.23046E-29
ENSMUSG00000062563	Cys1	-1.794876285	1.32425E-14
ENSMUSG00000103726	Gm30074	-1.796200731	0.000135092
ENSMUSG00000068876	Cgn	-1.796816824	0.001347115
ENSMUSG00000031880	Rrad	-1.798590098	1.11168E-10
ENSMUSG00000041498	Kif14	-1.79955132	3.37062E-82
ENSMUSG00000070713	Gm10282	-1.800894165	6.46024E-05
ENSMUSG00000105096	Gbp10	-1.800962801	2.79261E-05
ENSMUSG00000046324	Ermp1	-1.801220967	1.19506E-76
ENSMUSG00000095865	Gm13237	-1.801271438	0.005585096
ENSMUSG00000020641	Rsad2	-1.802131628	7.7683E-05
ENSMUSG00000023341	Mx2	-1.804108317	1.6774E-05
ENSMUSG00000060795	Gm13363	-1.805116591	0.008358374
ENSMUSG00000090698	Apold1	-1.808438939	2.03376E-31
ENSMUSG00000083811	Gm13071	-1.810618551	0.003615834

ENSMUSG00000072720	Myo18b	-1.810954635	8.95807E-24
ENSMUSG00000081833	Gm13669	-1.812436833	0.002871906
ENSMUSG00000027171	Prrg4	-1.821052162	4.95006E-53
ENSMUSG00000052485	Tmem171	-1.821694745	8.80681E-11
ENSMUSG00000026211	Obsl1	-1.821950644	2.3901E-220
ENSMUSG00000022177	Haus4	-1.822167468	3.32682E-48
ENSMUSG00000096592	Gm15801	-1.823619467	1.69051E-13
ENSMUSG00000034799	Unc13a	-1.827391716	9.14595E-13
ENSMUSG00000094388	Gm8783	-1.828366944	1.89822E-19
ENSMUSG00000047534	Mis18bp1	-1.829090379	4.1042E-129
ENSMUSG00000022360	Atad2	-1.829539354	3.4322E-214
ENSMUSG00000002055	Spag5	-1.830193436	5.5479E-139
ENSMUSG00000039556	Ppp1r3f	-1.830828385	4.04243E-09
ENSMUSG00000023336	Wfdc1	-1.832394672	0.007489818
ENSMUSG00000034059	Ypel4	-1.844313861	3.38888E-05
ENSMUSG00000032254	Kif23	-1.844333127	2.4736E-215
ENSMUSG00000070044	Fam149a	-1.846627029	1.4982E-155
ENSMUSG00000041923	Nol4	-1.847428008	4.16002E-06
ENSMUSG00000034990	Otoa	-1.848623838	0.000138496
ENSMUSG00000045333	Zfp423	-1.849189442	3.60712E-09
ENSMUSG00000028211	Trp53inp1	-1.849636029	0
ENSMUSG00000051243	Islr2	-1.852723198	0.038739679
ENSMUSG00000020492	Ska2	-1.85425502	1.0734E-119
ENSMUSG00000040084	Bub1b	-1.855416267	6.1154E-213
ENSMUSG00000026768	Itga8	-1.8554656	2.51237E-33
ENSMUSG00000032135	Mcam	-1.856367517	7.34396E-83
ENSMUSG00000007122	Casq1	-1.86347948	3.43259E-07
ENSMUSG00000051043	Gprc5c	-1.864630034	0.001605583
ENSMUSG00000030055	Rab43	-1.864889286	0
ENSMUSG00000063388	BC023105	-1.868029929	2.50423E-13
ENSMUSG00000073555	Gm4951	-1.870045596	4.95877E-22
ENSMUSG00000047182	Irs3	-1.87042648	0.011518089
ENSMUSG00000046591	Ticrr	-1.870656588	4.329E-38
ENSMUSG00000018654	Ikzf1	-1.872837551	0.014195869
ENSMUSG00000032202	Rab27a	-1.872910556	0.007126616
ENSMUSG00000020674	Pxdn	-1.874042619	0
ENSMUSG00000026589	Sec16b	-1.880689477	2.13344E-65
ENSMUSG00000027326	Knl1	-1.881164166	1.7414E-122
ENSMUSG00000032783	Troap	-1.883097894	4.34876E-58
ENSMUSG00000037419	Endod1	-1.885224175	1.2862E-276
ENSMUSG00000068748	Ptprz1	-1.890201458	1.41369E-06
ENSMUSG00000048621	Gm6377	-1.89116555	0.003465607
ENSMUSG00000056145	AI504432	-1.897923879	0.004156819
ENSMUSG00000045328	Cenpe	-1.898094411	8.8703E-221
ENSMUSG00000041147	Brca2	-1.898435833	7.28419E-48
ENSMUSG00000039187	Fanci	-1.907416907	2.34045E-45
ENSMUSG00000024026	Glo1	-1.908046491	7.3358E-161
ENSMUSG00000026023	Cdk15	-1.909913518	3.63774E-05
ENSMUSG00000027331	Knstrn	-1.910081367	6.0773E-198
ENSMUSG00000042155	Klhl23	-1.910250336	8.03128E-31
ENSMUSG00000044139	Prss53	-1.911995951	0.00027022
ENSMUSG00000035365	Parpbp	-1.912895124	4.30961E-61
ENSMUSG00000038943	Prc1	-1.913315574	0
ENSMUSG00000030054	Gp9	-1.915636019	0.006821117
ENSMUSG00000078521	Aunip	-1.920338008	1.86532E-13
ENSMUSG00000003545	Fosb	-1.920631392	1.32218E-20
ENSMUSG00000026051	Ecrg4	-1.924181649	3.7479E-107
ENSMUSG00000020023	Tmcc3	-1.925471269	4.70841E-98
ENSMUSG00000057606	Colq	-1.925556055	0.002228403
ENSMUSG00000029438	Bcl7a	-1.928150078	2.81892E-70
ENSMUSG00000092021	Gbp11	-1.934146409	0.012142081
ENSMUSG00000022602	Arc	-1.934250426	7.02672E-28
ENSMUSG00000113909	Gm36377	-1.938382582	0.001423623
ENSMUSG00000051378	Kif18b	-1.941017534	1.11403E-90
ENSMUSG00000109904	Gm45819	-1.943906843	1.47952E-06
ENSMUSG00000050762	Prss27	-1.944871971	7.1077E-05
ENSMUSG00000079067	Hmgn2-ps1	-1.945060163	9.29111E-07
ENSMUSG00000021057	Akap5	-1.945261016	4.07209E-30
ENSMUSG00000113831	Gm49602	-1.945769387	1.9463E-05
ENSMUSG00000038379	Ttk	-1.946881027	1.06898E-56
ENSMUSG00000096727	Psmb9	-1.947747615	8.31801E-12
ENSMUSG00000037206	Islr	-1.948151191	2.2681E-105
ENSMUSG00000028782	Adgrb2	-1.948372784	2.33229E-07
ENSMUSG00000073600	Prob1	-1.950491576	3.74112E-05
ENSMUSG00000029516	Cit	-1.952469998	9.0477E-96
ENSMUSG00000037108	Zcwpw1	-1.953639859	9.42595E-05
ENSMUSG00000031196	F8	-1.957327501	3.85264E-26

ENSMUSG00000024598	Fbn2	-1.969510153	1.16424E-11
ENSMUSG00000047757	Fancb	-1.970206362	3.42742E-26
ENSMUSG00000041633	Kctd12b	-1.971711587	3.03854E-66
ENSMUSG00000033590	Myo5c	-1.973685171	0.000288369
ENSMUSG00000014773	Dll1	-1.973897514	0.000358658
ENSMUSG00000037624	Kcnk2	-1.974215821	6.2907E-120
ENSMUSG00000040310	Alx4	-1.976869018	3.39026E-05
ENSMUSG00000059901	Adamts14	-1.976904132	5.6922E-138
ENSMUSG00000029414	Kntc1	-1.977238958	1.5511E-125
ENSMUSG00000031805	Jak3	-1.981268791	5.50883E-56
ENSMUSG00000034987	Hrh2	-1.981815293	0.048034951
ENSMUSG00000026196	Bard1	-1.983422465	2.42607E-34
ENSMUSG00000020638	Cmpk2	-1.983801503	2.59898E-06
ENSMUSG00000021922	Itih4	-1.984397517	0.038854723
ENSMUSG00000030074	Gxylt2	-1.98700989	2.4858E-267
ENSMUSG00000031780	Ccl17	-1.987855915	1.49799E-11
ENSMUSG00000031004	Mki67	-1.988122262	0
ENSMUSG00000025507	Pidd1	-1.992949603	2.41819E-90
ENSMUSG00000116919	Gm17809	-1.993752149	0.009048105
ENSMUSG00000026700	Tnfsf4	-1.999158373	0.002945475
ENSMUSG00000031756	Cenpn	-1.999990037	2.51613E-87
ENSMUSG00000021176	Efcab11	-2.00140341	9.25605E-17
ENSMUSG00000023032	Slc4a8	-2.00278202	2.63712E-05
ENSMUSG00000031158	Timm17b	-2.002991531	4.25378E-97
ENSMUSG00000048939	Atp13a5	-2.004752766	0.0048046
ENSMUSG00000020475	Pgam2	-2.00508997	1.10821E-11
ENSMUSG00000097463	4930448E22Rik	-2.007214099	0.008964099
ENSMUSG00000062488	Ifit3b	-2.011379034	8.54702E-11
ENSMUSG00000117310	Ptp4a1	-2.020150327	0
ENSMUSG00000118364	AC124502.1	-2.0207867	0.000931336
ENSMUSG00000020493	Prr11	-2.025880848	9.0052E-176
ENSMUSG00000011263	Exoc3l2	-2.026300609	1.57518E-05
ENSMUSG00000024525	Impa2	-2.029085403	2.45859E-66
ENSMUSG00000032172	Olfm2	-2.029772407	3.45161E-16
ENSMUSG00000116347	Gm49416	-2.029996077	0.035694226
ENSMUSG00000021391	Cenpp	-2.03192968	4.54408E-13
ENSMUSG00000014782	Plekhg4	-2.032025097	0.047031669
ENSMUSG00000051517	Arhgef39	-2.034192292	4.97313E-29
ENSMUSG00000114019	Gm47155	-2.034623098	5.88848E-06
ENSMUSG00000112026	Gm6653	-2.036150054	0.007042957
ENSMUSG00000024579	Pcyox1l	-2.036407125	1.73468E-39
ENSMUSG00000112794	Gm48878	-2.041934611	0.000100279
ENSMUSG00000066554	Gm10167	-2.047386897	0.006787352
ENSMUSG00000062510	Nsl1	-2.049545046	1.07091E-53
ENSMUSG00000034614	Pik3ip1	-2.051718878	5.93877E-22
ENSMUSG00000084844	Hoxb3os	-2.05616873	0.003671004
ENSMUSG00000093726	Gm20667	-2.059275788	8.30067E-11
ENSMUSG00000041688	Amot	-2.060895813	4.2843E-165
ENSMUSG00000075502	Kbtbd6	-2.061062697	1.623E-09
ENSMUSG00000051817	Sox12	-2.063564319	4.90694E-23
ENSMUSG00000040856	Dlk1	-2.064196401	0.003967696
ENSMUSG00000047658	Gal3st3	-2.064574698	6.45597E-05
ENSMUSG00000026675	Hsd17b7	-2.070395647	0
ENSMUSG00000020808	Pimreg	-2.070915921	1.7187E-144
ENSMUSG00000044966	Fbxo48	-2.072339845	4.7215E-14
ENSMUSG00000037544	Dlgap5	-2.077439467	3.9625E-275
ENSMUSG00000050666	Vstm4	-2.078279687	2.74372E-05
ENSMUSG00000020185	E2f7	-2.079332143	2.2078E-81
ENSMUSG00000086555	Gm13446	-2.084316685	0.000817336
ENSMUSG00000014077	Chp1	-2.084866118	0
ENSMUSG00000028287	1700009N14Rik	-2.089887736	0.002712804
ENSMUSG00000071112	Spx	-2.090503963	0.001018441
ENSMUSG00000056481	Cd248	-2.094119047	3.1383E-169
ENSMUSG00000070348	Ccnd1	-2.094259752	0
ENSMUSG00000024338	Psmb8	-2.09564616	1.2925E-40
ENSMUSG00000028718	Stil	-2.095903323	1.1752E-130
ENSMUSG00000019737	Syne4	-2.096159546	0.007939472
ENSMUSG00000116665	E130310I04Rik	-2.10093861	0.019150206
ENSMUSG00000040086	Tnni3k	-2.101042441	3.83689E-23
ENSMUSG00000047842	Diras2	-2.10161336	1.76175E-14
ENSMUSG00000019945	Cabcoco1	-2.102235196	1.17804E-07
ENSMUSG00000009394	Syn2	-2.103233629	3.1644E-08
ENSMUSG00000116597	Gm536	-2.103571702	2.72031E-05
ENSMUSG00000021453	Gadd45g	-2.113070069	1.338E-113
ENSMUSG00000033752	Mnd1	-2.114138196	7.07893E-12
ENSMUSG00000032009	Sesn3	-2.1163796	1.1569E-111
ENSMUSG00000021624	Cd180	-2.116380987	2.05159E-51

ENSMUSG00000117964	Gm36043	-2.117093992	0.000222394
ENSMUSG00000061762	Tac1	-2.13268122	0.041563574
ENSMUSG00000032769	Trpa1	-2.133794208	0.000271322
ENSMUSG00000071547	Nt5dc2	-2.138549004	2.1778E-127
ENSMUSG00000009654	Oit3	-2.139617251	1.90361E-13
ENSMUSG00000068101	Cenpm	-2.146350003	5.87172E-26
ENSMUSG00000010362	Rdm1	-2.156792918	7.41417E-05
ENSMUSG00000041482	Piezo2	-2.15688648	1.47151E-40
ENSMUSG00000071724	Smpd5	-2.157438489	0.007150164
ENSMUSG00000052353	Cemip	-2.159060514	0
ENSMUSG00000029735	Tpk1	-2.165482568	1.11593E-35
ENSMUSG00000039853	Trim14	-2.176520935	7.27913E-05
ENSMUSG00000111738	Gm18329	-2.177936409	0.000494033
ENSMUSG00000064043	Trerf1	-2.180141641	2.26519E-77
ENSMUSG00000050132	Sarm1	-2.181816608	0.019339297
ENSMUSG00000036862	Dchs1	-2.182239158	2.81075E-61
ENSMUSG00000024301	Kifc5b	-2.183170011	2.14807E-94
ENSMUSG00000104342	Gm36401	-2.184952602	1.40586E-06
ENSMUSG00000079553	Kifc1	-2.191422958	6.2331E-230
ENSMUSG00000032698	Lmo2	-2.196146729	4.58402E-90
ENSMUSG00000118138	Gm50322	-2.209854265	4.27419E-17
ENSMUSG00000043496	Tril	-2.210347717	4.32457E-17
ENSMUSG00000051457	Spn	-2.21460841	7.00585E-17
ENSMUSG00000033544	Angptl1	-2.215260258	8.55377E-06
ENSMUSG00000032648	Pygm	-2.215417324	8.07527E-25
ENSMUSG00000108176	Gm43965	-2.218535038	0.003396591
ENSMUSG00000021367	Edn1	-2.223806321	8.2368E-127
ENSMUSG00000102840	Gm38037	-2.234211192	0.005433237
ENSMUSG00000037337	Map4k1	-2.241797974	3.34684E-06
ENSMUSG00000029298	Gbp9	-2.245497631	8.30084E-44
ENSMUSG00000042115	Klhdc8a	-2.24635308	9.17887E-80
ENSMUSG00000026610	Esrrg	-2.251335016	2.25768E-08
ENSMUSG00000075010	AW112010	-2.253819575	0.010961412
ENSMUSG00000009281	Rarres2	-2.256766243	7.8268E-09
ENSMUSG00000035711	Dok3	-2.257090874	1.11767E-08
ENSMUSG00000034872	Gipc3	-2.265250371	0.04225707
ENSMUSG00000031351	Zfp185	-2.268107469	2.24344E-05
ENSMUSG00000109056	A630009H07Rik	-2.27568783	0.027342102
ENSMUSG00000041064	Pif1	-2.276041961	1.6506E-102
ENSMUSG00000108671	2700080J24Rik	-2.276321569	0.001458866
ENSMUSG00000078773	Rad54b	-2.285949262	6.44234E-27
ENSMUSG00000027335	Adra1d	-2.289990802	1.35525E-05
ENSMUSG00000053646	Plxnb1	-2.300128134	1.43189E-16
ENSMUSG00000104965	Gm43437	-2.305071691	3.23458E-07
ENSMUSG00000025666	Tmem47	-2.306322602	1.932E-118
ENSMUSG00000082292	Gm12250	-2.313466703	4.09798E-07
ENSMUSG00000048655	Ccdc169	-2.316985147	0.04975348
ENSMUSG00000026126	Ptpn18	-2.31711276	0.010688712
ENSMUSG00000022758	P2rx6	-2.322582854	4.33493E-06
ENSMUSG00000069793	Slfn9	-2.322988871	1.1331E-251
ENSMUSG00000048402	Gli2	-2.327811667	5.38164E-05
ENSMUSG00000026873	Phf19	-2.33293681	6.60491E-90
ENSMUSG00000034818	Celf5	-2.334963576	1.74759E-17
ENSMUSG00000110575	6330537M06Rik	-2.346569682	7.0553E-06
ENSMUSG00000024440	Pcdh12	-2.354402287	2.4003E-17
ENSMUSG00000033207	Mamdc2	-2.355558395	2.14202E-93
ENSMUSG00000021055	Esr2	-2.356138922	0.002013819
ENSMUSG00000021697	Depdc1b	-2.358945513	2.07279E-23
ENSMUSG00000049092	Gpr137c	-2.361866866	4.11084E-07
ENSMUSG00000018570	2810408A11Rik	-2.367347949	0.029052932
ENSMUSG00000057751	Megf6	-2.371321737	0.002538743
ENSMUSG00000029168	Dpysl5	-2.37147939	0.002572507
ENSMUSG00000030742	Lat	-2.375599225	0.031906348
ENSMUSG00000005357	Slc1a6	-2.383335807	9.77963E-55
ENSMUSG00000079652	Fam71f2	-2.383654918	0.03240711
ENSMUSG00000029716	Tfr2	-2.386610071	0.003527122
ENSMUSG00000004880	Lbr	-2.392855967	0
ENSMUSG00000045102	Poln	-2.394329423	1.34269E-07
ENSMUSG00000034457	Eda2r	-2.398728613	0
ENSMUSG00000053007	Creb5	-2.400056795	2.7259E-103
ENSMUSG00000038146	Notch3	-2.405083488	6.2209E-234
ENSMUSG00000105954	Gm42793	-2.417202544	0.010351016
ENSMUSG00000017446	C1qtnf1	-2.421894025	2.6771E-114
ENSMUSG00000036339	Tmem260	-2.423295632	5.70105E-78
ENSMUSG00000104507	A430027H14Rik	-2.443261201	0.015093416
ENSMUSG00000050640	Tmem150c	-2.446946891	0.000993174
ENSMUSG00000001247	Lsr	-2.44733328	0.000123745

ENSMUSG00000072437	Nanos1	-2.455823331	2.99224E-71
ENSMUSG00000030319	Cand2	-2.457234938	4.3363E-171
ENSMUSG00000053318	Slamf8	-2.46319864	0.011685281
ENSMUSG00000020787	P2rx1	-2.466915052	1.51924E-05
ENSMUSG00000022353	Mtss1	-2.472708959	4.90823E-53
ENSMUSG00000053964	Lgals4	-2.483784398	0.008089451
ENSMUSG00000054675	Tmem119	-2.497198302	1.91314E-73
ENSMUSG00000112932	Gm48308	-2.499684802	0.00066468
ENSMUSG00000026715	Serpinc1	-2.504192071	2.9008E-05
ENSMUSG00000073295	Nudt11	-2.50598813	1.33071E-08
ENSMUSG00000034041	Lyl1	-2.509957627	0.015093416
ENSMUSG00000043165	Lor	-2.511857668	5.05358E-18
ENSMUSG00000057948	Unc13d	-2.513941186	0.005978592
ENSMUSG00000100798	Gm19589	-2.515309017	9.97609E-07
ENSMUSG00000034855	Cxcl10	-2.522933542	1.44051E-07
ENSMUSG00000086528	Gm15731	-2.528092944	4.94566E-08
ENSMUSG00000082088	Gm15753	-2.528721326	0.031369264
ENSMUSG00000037995	Igsf9	-2.528783392	7.38203E-10
ENSMUSG00000039814	Xkr5	-2.532762742	6.13273E-12
ENSMUSG00000046186	Cd109	-2.549190732	0
ENSMUSG00000040270	Bach2	-2.549261509	1.55768E-17
ENSMUSG00000052544	St6galnac3	-2.557163415	0.005986741
ENSMUSG00000116311	4930478M13Rik	-2.560892868	0.023443647
ENSMUSG00000068606	Gm4841	-2.562253059	1.30286E-11
ENSMUSG00000068522	Aard	-2.570305098	0.001850215
ENSMUSG00000069227	Gprin1	-2.5854565	0.018556346
ENSMUSG00000103469	Gm9910	-2.58789157	0.000962127
ENSMUSG00000020805	Slc13a5	-2.596822902	0.018629444
ENSMUSG00000029862	Clcn1	-2.598084512	4.57813E-05
ENSMUSG00000028626	Col9a2	-2.605877748	0.049336621
ENSMUSG00000042834	Nrep	-2.610773236	0
ENSMUSG00000107432	Gm36189	-2.610854238	0.009186081
ENSMUSG00000051805	A530095I07Rik	-2.611066888	0.049817863
ENSMUSG00000036306	Lzts1	-2.616240886	2.83704E-08
ENSMUSG00000022032	Scara5	-2.625599479	2.64341E-12
ENSMUSG00000000244	Tspan32	-2.632238925	2.13007E-05
ENSMUSG00000060183	Cxcl11	-2.634965851	0.036178372
ENSMUSG00000114165	Gm47513	-2.635483503	0.000725598
ENSMUSG00000045062	Pcdhb7	-2.639743265	4.27315E-05
ENSMUSG00000031994	Adamts8	-2.640708325	7.76695E-14
ENSMUSG00000030699	Tbx6	-2.645616314	0.011461867
ENSMUSG00000034438	Gbp8	-2.647916531	0.016368788
ENSMUSG00000037705	Tecta	-2.650400812	1.86746E-08
ENSMUSG00000023015	Racgap1	-2.652320833	0
ENSMUSG00000025610	Map3k7cl	-2.661608938	4.24236E-58
ENSMUSG00000062151	Unc13c	-2.663043711	8.68253E-27
ENSMUSG00000000386	Mx1	-2.670460251	9.80253E-15
ENSMUSG00000068246	Apol9b	-2.675959936	1.56199E-61
ENSMUSG00000050822	Slc29a4	-2.679314747	0.003406599
ENSMUSG00000002324	Rec8	-2.680861973	0.006095952
ENSMUSG00000049173	Myoz3	-2.681631128	0.00278816
ENSMUSG00000044814	Olfr543	-2.683494642	0.020356318
ENSMUSG00000001155	Ftcd	-2.684853519	0.047963692
ENSMUSG00000026683	Nuf2	-2.688631312	1.5295E-252
ENSMUSG00000097709	2810429I04Rik	-2.698703735	0.011188205
ENSMUSG00000108218	Olfr1372-ps1	-2.706267025	1.36475E-33
ENSMUSG00000039155	Cdh26	-2.712700167	0.000853031
ENSMUSG00000074673	Ttll9	-2.714352665	0.008150825
ENSMUSG00000033952	Aspm	-2.731300136	1.2603E-301
ENSMUSG00000036295	Lrrn3	-2.74964956	3.79254E-11
ENSMUSG00000002007	Srpk3	-2.754246042	0.04725054
ENSMUSG00000022464	Slc38a4	-2.767868891	1.33788E-66
ENSMUSG00000027489	Necab3	-2.787888717	0.002409775
ENSMUSG00000079323	Gm20661	-2.798299949	0.014974999
ENSMUSG00000051920	Rspo2	-2.802255398	0.000167424
ENSMUSG00000103558	Gm38220	-2.809160512	0.008577317
ENSMUSG00000048485	Zbtb8b	-2.817783574	3.11449E-10
ENSMUSG00000004791	Pgf	-2.82691813	3.3771E-166
ENSMUSG00000047638	Nr1h4	-2.851958094	1.29844E-57
ENSMUSG00000057346	Apol9a	-2.864869124	2.64219E-35
ENSMUSG00000105613	Gm43684	-2.865745957	0.036766586
ENSMUSG00000048126	Col6a3	-2.866561577	0
ENSMUSG00000115902	AC113595.1	-2.86708654	0.011464566
ENSMUSG00000079588	Tmem182	-2.867647291	1.55319E-10
ENSMUSG00000025076	Casp7	-2.868191625	7.5386E-181
ENSMUSG00000037846	Rtkn2	-2.883229722	1.26102E-30
ENSMUSG00000078234	Klhdc7a	-2.914336149	3.08489E-21

ENSMUSG00000029861	Fam131b	-2.929831364	1.46945E-22
ENSMUSG00000026548	Slamf9	-2.942761575	2.83286E-22
ENSMUSG00000099465	Gm3830	-2.961309021	0.004656786
ENSMUSG00000029838	Ptn	-2.989723574	1.8576E-295
ENSMUSG00000024155	Meiob	-3.012750814	0.000981369
ENSMUSG00000047146	Tet1	-3.032862312	1.17667E-20
ENSMUSG00000025475	Adgra1	-3.054770289	4.11244E-09
ENSMUSG00000038540	Tmc3	-3.061643874	2.30444E-07
ENSMUSG00000079355	Ackr4	-3.069676724	2.9089E-100
ENSMUSG00000078922	Tgtp1	-3.072226746	1.1464E-39
ENSMUSG00000055027	Smyd1	-3.084804052	6.09576E-10
ENSMUSG00000041329	Atp1b2	-3.086327466	5.13232E-10
ENSMUSG00000070388	Fbxo39	-3.097285037	0.040603856
ENSMUSG00000045087	S1pr5	-3.107513097	0.000459781
ENSMUSG00000025422	Agap2	-3.114516007	0.039212719
ENSMUSG00000109134	Gm45076	-3.122984032	3.35997E-06
ENSMUSG00000021751	Acox2	-3.130046284	0.023590289
ENSMUSG00000015854	Cd5l	-3.130898848	0.005339532
ENSMUSG00000067235	H2-Q10	-3.138473256	6.82259E-06
ENSMUSG00000057880	Abat	-3.139507006	3.8078E-165
ENSMUSG00000075070	4932412D23Rik	-3.164704865	0.012343599
ENSMUSG00000029005	Draxin	-3.166077663	0.048359618
ENSMUSG00000081755	Mup-ps23	-3.17187741	0.047708637
ENSMUSG00000084050	Gm14439	-3.175232201	0.044334222
ENSMUSG00000002668	Dennd1c	-3.178499828	0.043342858
ENSMUSG00000052854	Nrk	-3.191260691	1.6738E-153
ENSMUSG00000031778	Cx3cl1	-3.208055752	0
ENSMUSG00000033849	B3galt2	-3.221175514	0.018861083
ENSMUSG00000111857	1190001M18Rik	-3.229836316	4.1857E-08
ENSMUSG00000022868	Ahsg	-3.240579124	0.002780709
ENSMUSG00000078240	Gm3550	-3.245511477	0.008936105
ENSMUSG00000006235	Epor	-3.258265687	1.28859E-06
ENSMUSG00000000982	Ccl3	-3.28276218	0.016066797
ENSMUSG00000035852	Misp	-3.28355802	0.015901776
ENSMUSG00000039899	Fgl2	-3.287924729	3.9135E-190
ENSMUSG00000022821	Hgd	-3.30689078	0.000515129
ENSMUSG00000030543	Mesp2	-3.324111308	0.041678171
ENSMUSG00000108010	Gm38708	-3.342545833	0.020653183
ENSMUSG00000074517	Gm10710	-3.378088968	0.0134882
ENSMUSG00000034687	Fras1	-3.400109809	0.001618521
ENSMUSG00000024990	Rbp4	-3.400198931	0.006193166
ENSMUSG00000090394	4930523C07Rik	-3.443123871	5.5525E-168
ENSMUSG00000026564	Dusp27	-3.458835374	9.79531E-53
ENSMUSG00000078921	Tgtp2	-3.470762904	2.76511E-89
ENSMUSG00000006542	Prkag3	-3.49120144	0.024683366
ENSMUSG00000025401	Myo1a	-3.494857231	0.003113176
ENSMUSG00000035407	Kank4	-3.501983971	0.000377912
ENSMUSG00000020017	Hal	-3.509662688	0.02258549
ENSMUSG00000117029	Gm36201	-3.532270119	0.012043437
ENSMUSG00000020155	Kcnmb1	-3.563462375	6.20838E-15
ENSMUSG00000040253	Gbp7	-3.571024193	1.92963E-51
ENSMUSG00000015947	Fcgr1	-3.582183791	8.28118E-05
ENSMUSG00000113619	Gm47118	-3.591607284	0.009536273
ENSMUSG00000050357	Carmil2	-3.622587952	0.030167855
ENSMUSG00000021448	Shc3	-3.625489582	7.0664E-12
ENSMUSG00000057074	Ces1g	-3.644883149	0.009202485
ENSMUSG00000000673	Haao	-3.652082503	0.000337635
ENSMUSG00000100944	Gm28900	-3.686481658	0.043276713
ENSMUSG00000032595	Cdhr4	-3.717897907	8.12747E-06
ENSMUSG00000055489	Ano5	-3.721405102	0.00305663
ENSMUSG00000086103	Gm11832	-3.725246109	0.018255693
ENSMUSG00000001663	Gstt1	-3.761494011	7.02902E-96
ENSMUSG00000083668	Gm5648	-3.768738799	0.003792948
ENSMUSG00000039304	Tnfsf10	-3.772381879	7.18649E-21
ENSMUSG00000030895	Hpx	-3.788121151	0.002776509
ENSMUSG00000068859	Sp9	-3.795398289	9.18982E-08
ENSMUSG00000053024	Cntn2	-3.859665828	3.31387E-19
ENSMUSG00000036596	Cpz	-3.910605055	5.15751E-10
ENSMUSG00000051726	Kcnf1	-3.953312316	7.77621E-07
ENSMUSG00000079363	Gbp4	-3.977263241	5.58337E-24
ENSMUSG00000027875	Hmgcs2	-4.044948281	0.013440431
ENSMUSG00000058396	Gpr182	-4.059818322	1.5354E-106
ENSMUSG00000050014	Apol10b	-4.06320661	3.6121E-09
ENSMUSG00000079564	Gm11149	-4.116164741	0.03879652
ENSMUSG00000103625	Gm37357	-4.150943967	0.037755374
ENSMUSG00000006522	Itih3	-4.182573871	1.04668E-07
ENSMUSG00000090125	Pou3f1	-4.205398432	7.60933E-09

ENSMUSG00000045534	Kcna5	-4.221417693	5.01096E-26
ENSMUSG00000039438	Ttc36	-4.225394821	0.001841109
ENSMUSG00000021200	Asb2	-4.228629069	0.00022983
ENSMUSG00000070644	Etnk2	-4.268752692	2.09112E-14
ENSMUSG00000068086	Cyp2d9	-4.308583788	0.026869946
ENSMUSG00000030124	Lag3	-4.313716095	0.000112866
ENSMUSG00000005681	Apoa2	-4.314991151	0.00076804
ENSMUSG00000030310	Slc6a1	-4.348081537	0.000126314
ENSMUSG00000029797	Sspo	-4.348423576	8.61405E-06
ENSMUSG00000020798	Spns3	-4.459556399	5.97434E-05
ENSMUSG00000051716	Apon	-4.532862843	5.77905E-05
ENSMUSG00000037798	Mat1a	-4.53373651	0.000382577
ENSMUSG00000047631	Apof	-4.689465739	5.20914E-05
ENSMUSG00000086083	Gm15829	-4.748408395	0.033460125
ENSMUSG00000019987	Arg1	-4.847847404	0.009236309
ENSMUSG00000107468	5730507A11Rik	-4.987944783	0.001850215
ENSMUSG00000018459	Slc13a3	-5.066849769	8.85171E-06
ENSMUSG00000032739	Pram1	-5.167545688	3.17924E-05
ENSMUSG00000086462	Gm13990	-5.195146791	0.043105822
ENSMUSG00000104306	Gm36972	-5.196003646	0.04323153
ENSMUSG00000029685	Asb15	-5.246233795	8.56978E-08
ENSMUSG00000067455	H4c11	-5.26320259	0.038629262
ENSMUSG00000117762	Gm30889	-5.263436806	0.037941187
ENSMUSG00000111504	Gm48284	-5.32705852	0.031605232
ENSMUSG00000087149	Itih5l-ps	-5.327249815	0.031065592
ENSMUSG00000092556	Olfr755-ps1	-5.328016447	0.03153691
ENSMUSG00000046999	1110032F04Rik	-5.356005482	2.77174E-23
ENSMUSG00000105935	Gm43628	-5.385723577	0.026586531
ENSMUSG00000051212	Gpr183	-5.388926353	0.026075617
ENSMUSG00000098210	Gm5224	-5.390981157	0.028196907
ENSMUSG00000085941	Gm11201	-5.393639997	0.03930244
ENSMUSG00000048489	Depp1	-5.44427739	0.022687399
ENSMUSG00000022900	Ildr1	-5.44517656	0.022299716
ENSMUSG00000049653	Spatc1	-5.501242644	0.018956113
ENSMUSG00000076439	Mog	-5.501950032	0.019332942
ENSMUSG00000060268	Armh1	-5.50311337	0.047289921
ENSMUSG00000022304	Dpys	-5.504525522	0.047804329
ENSMUSG00000089797	Gm16118	-5.505572429	0.019744426
ENSMUSG00000092201	A530058N18Rik	-5.553761417	0.042594411
ENSMUSG00000078127	Fam170b	-5.554667561	0.042046209
ENSMUSG00000108081	Gm44026	-5.555879929	0.01593985
ENSMUSG00000110787	Gm6667	-5.556488374	0.041797703
ENSMUSG00000112481	Gm29684	-5.557216004	0.015484605
ENSMUSG00000032327	Stra6	-5.557896818	0.015863593
ENSMUSG00000034829	Nxnl1	-5.558742284	0.017116015
ENSMUSG00000085398	4931406G06Rik	-5.55920957	0.042518654
ENSMUSG00000083885	Gm13213	-5.561076669	0.018354253
ENSMUSG00000027776	Il12a	-5.561338985	0.048974693
ENSMUSG00000036962	Cfap221	-5.603372913	0.039122784
ENSMUSG00000073940	Hbb-bt	-5.607958189	0.037272717
ENSMUSG00000044518	Foxe3	-5.608842991	0.038207807
ENSMUSG00000107989	Gm19692	-5.608995569	0.013679
ENSMUSG00000036110	Slc17a2	-5.609271981	0.037168875
ENSMUSG00000041550	Serpina5	-5.612226569	0.013568022
ENSMUSG00000061577	Adgrg5	-5.61320216	0.014753437
ENSMUSG00000070533	Wfdc8	-5.614267205	0.039728428
ENSMUSG00000022156	Gzme	-5.61683193	0.039515326
ENSMUSG00000111671	Gm6983	-5.617322938	0.043743115
ENSMUSG00000021492	F12	-5.626820677	2.64642E-05
ENSMUSG00000095956	1700036A12Rik	-5.659479751	0.015816566
ENSMUSG00000040140	Tdrd6	-5.659782423	0.011314494
ENSMUSG00000086480	Gm15287	-5.661659906	0.011930074
ENSMUSG00000030972	Acsm5	-5.665263073	0.036575816
ENSMUSG00000116777	Zfp520-ps	-5.705797148	0.011067404
ENSMUSG00000091055	Siglec15	-5.706385261	0.011518089
ENSMUSG00000027579	Srms	-5.708226607	0.009952258
ENSMUSG00000025279	Dnase1l3	-5.709377129	0.029393283
ENSMUSG00000035836	Ugt2b1	-5.709989982	0.029482854
ENSMUSG00000050700	Emilin3	-5.712273922	0.010131115
ENSMUSG00000093634	Gm10860	-5.724094916	0.000937235
ENSMUSG00000002992	Apoc2	-5.752183868	0.027126103
ENSMUSG00000042474	Fcmr	-5.753030944	0.009438593
ENSMUSG00000022510	Trp63	-5.753618254	0.009055295
ENSMUSG00000031981	Capn9	-5.757877809	0.009320755
ENSMUSG00000054757	Akr1c20	-5.798377861	0.023986977
ENSMUSG00000001943	Vsig2	-5.801323481	0.006991344
ENSMUSG00000086050	Gm16045	-5.802725892	0.007327045

ENSMUSG00000030834	Abcc6	-5.805687968	0.008569412
ENSMUSG00000029260	Ugt2b34	-5.807585461	0.026633683
ENSMUSG00000096883	Shisa8	-5.843186071	0.02082819
ENSMUSG00000062007	Hsh2d	-5.845932983	0.02070066
ENSMUSG00000102605	Gm37264	-5.846836596	0.006909645
ENSMUSG00000082272	Gm11675	-5.852297029	0.009232872
ENSMUSG00000091864	Gm17102	-5.852660989	0.020403537
ENSMUSG00000020140	Lgr5	-5.884412166	0.019176427
ENSMUSG00000042118	Bhmt2	-5.887638069	0.01866117
ENSMUSG00000114656	2810403G07Rik	-5.887638069	0.01866117
ENSMUSG00000066975	Cryba4	-5.890387525	0.004826814
ENSMUSG00000086495	Gm13778	-5.890669172	0.005506189
ENSMUSG00000026322	Htr4	-5.890766161	0.005804396
ENSMUSG00000025396	Hsd17b6	-5.891429888	0.018883968
ENSMUSG00000117608	AC103362.1	-5.895399582	0.018336042
ENSMUSG00000058806	Col13a1	-5.896591523	0.019098984
ENSMUSG00000024863	Mbl2	-5.923923867	0.020253267
ENSMUSG00000038916	Soga3	-5.9293212	0.004784144
ENSMUSG00000004031	Brinp2	-5.93047253	0.017096997
ENSMUSG00000066366	Serpina1a	-5.935426796	0.009488363
ENSMUSG00000015401	Cltrn	-5.968106298	0.015506748
ENSMUSG00000047619	Ddi1	-5.968620248	0.015329278
ENSMUSG00000102615	Gm37844	-5.973231381	0.015056397
ENSMUSG00000026542	Apcs	-5.974254271	0.015286335
ENSMUSG00000093587	Gm20554	-5.976300481	0.016301406
ENSMUSG00000038115	Ano2	-5.977381935	0.015176009
ENSMUSG00000036892	Prodh2	-6.009964716	0.013414002
ENSMUSG00000066071	Cyp4a12a	-6.015940819	0.014778612
ENSMUSG00000032083	Apoa1	-6.04532538	0.0095433
ENSMUSG00000024694	Keg1	-6.051126947	0.012068955
ENSMUSG00000111774	Gm38398	-6.051292632	0.002836497
ENSMUSG00000090191	9230105E05Rik	-6.051640326	0.002628277
ENSMUSG00000092261	Gm20519	-6.053208474	0.003552212
ENSMUSG00000010760	Phlda2	-6.053647324	0.003007068
ENSMUSG00000041644	Slc5a12	-6.053657334	0.00301515
ENSMUSG00000034427	Myo15b	-6.084579628	0.010820178
ENSMUSG00000062624	Cyp2c67	-6.087370584	0.010767945
ENSMUSG00000084864	1700027A07Rik	-6.087671319	0.002422154
ENSMUSG00000067225	Cyp2c54	-6.087843341	0.010788676
ENSMUSG00000089678	Agxt2	-6.116692739	0.012098706
ENSMUSG00000038403	Hjv	-6.124109298	0.009690385
ENSMUSG00000086313	Gm15940	-6.125200323	0.002407802
ENSMUSG00000029368	Alb	-6.14578326	0.020959117
ENSMUSG00000085162	Gm12295	-6.158876594	0.001701267
ENSMUSG00000115979	Gm49410	-6.160222836	0.001999544
ENSMUSG00000025082	Vwa2	-6.162199113	0.001833903
ENSMUSG00000014542	Clec4f	-6.162633827	0.009211389
ENSMUSG00000035383	Pmch	-6.162640175	0.001905906
ENSMUSG00000058755	Osm	-6.164724895	0.00931854
ENSMUSG00000110273	Gm41231	-6.165599826	0.008600355
ENSMUSG00000116635	Gm49699	-6.193321513	0.00146389
ENSMUSG00000043747	1520401A03Rik	-6.199782873	0.008080849
ENSMUSG00000041660	Bbox1	-6.224612884	0.001820508
ENSMUSG00000025497	Cdhr5	-6.226018727	0.007199298
ENSMUSG00000086015	4833417C18Rik	-6.234009693	0.007106767
ENSMUSG00000064147	Rab44	-6.256383243	0.001908792
ENSMUSG00000115456	Gm35248	-6.25973705	0.001351572
ENSMUSG00000049593	Lce1h	-6.287968358	0.002013819
ENSMUSG00000086236	5830418P13Rik	-6.296383462	0.001016137
ENSMUSG00000116033	Gm49442	-6.296884902	0.00110455
ENSMUSG00000017344	Vtn	-6.302569248	0.001024125
ENSMUSG00000072849	Serpina1e	-6.323140496	0.036002215
ENSMUSG00000085207	Gm11767	-6.355920132	0.00067877
ENSMUSG00000030108	Slc6a13	-6.356311528	0.000690482
ENSMUSG00000020566	Atp6v1c2	-6.357956679	0.000797451
ENSMUSG00000027793	Ccna1	-6.385923805	0.000906679
ENSMUSG00000040794	C1qtnf4	-6.387708094	0.000557674
ENSMUSG00000057058	Skap1	-6.418550795	0.00062459
ENSMUSG00000086150	Bach2os	-6.419088939	0.000528102
ENSMUSG00000040061	Plcb2	-6.446343026	0.000581712
ENSMUSG00000005763	Cd247	-6.474539406	0.000446697
ENSMUSG00000079042	Apela	-6.476434585	0.000475249
ENSMUSG00000063821	Dupd1	-6.478645272	0.000555798
ENSMUSG00000105699	Gm43703	-6.500287852	0.000507094
ENSMUSG00000035395	Dcaf8l	-6.503662719	0.000511999
ENSMUSG00000110071	Gm45512	-6.50777578	0.000637484
ENSMUSG00000021509	Slc25a48	-6.53124498	0.000557539

ENSMUSG00000103383	Gm37754	-6.55385475	0.000346891
ENSMUSG00000034528	Hsd17b13	-6.559132843	0.000334398
ENSMUSG00000027345	4921508D12Rik	-6.584260551	0.000218813
ENSMUSG00000029445	Hpd	-6.59958196	0.008222734
ENSMUSG00000103738	Gm37652	-6.638775596	0.000305716
ENSMUSG00000022055	Nefl	-6.756992008	8.3632E-05
ENSMUSG00000052861	Dnah6	-6.777453082	9.31523E-05
ENSMUSG00000026354	Lct	-6.778898555	7.58038E-05
ENSMUSG00000116009	Gm49503	-6.82434028	8.61179E-05
ENSMUSG00000030237	Slco1a4	-6.825283895	7.71605E-05
ENSMUSG00000108024	Gm43912	-6.970937391	2.81545E-05
ENSMUSG00000054966	Lmntd1	-6.994380287	2.45204E-05
ENSMUSG00000085272	Sbk3	-6.995512313	3.66977E-05
ENSMUSG00000071178	Serpina1b	-7.029939833	0.018203835
ENSMUSG00000074817	Papolb	-7.072006967	1.9549E-05
ENSMUSG00000022149	C9	-7.143452781	3.58269E-05
ENSMUSG00000074628	Tldc2	-7.278948667	3.56751E-06
ENSMUSG00000097617	Gm10687	-7.292872325	4.46415E-06
ENSMUSG00000024292	Cyp4f14	-7.400107085	2.67033E-06
ENSMUSG00000002100	Mybpc3	-7.533442644	1.11407E-06
ENSMUSG00000028715	Cyp4a14	-7.583894109	3.42893E-06
ENSMUSG00000060371	Caln1	-8.010384383	2.98128E-08
ENSMUSG00000020884	Asgr1	-8.242807102	7.18356E-08
ENSMUSG00000073830	Mup14	-8.3804769	0.023409641
ENSMUSG00000078686	Mup9	-8.435852177	0.022687652
ENSMUSG00000069805	Fbp1	-8.447394828	2.17095E-08
ENSMUSG00000030382	Slc27a5	-8.598073781	0.000214426
ENSMUSG00000035283	Adrb1	-8.635857386	2.06478E-10
ENSMUSG00000059900	Tmem40	-8.715911188	1.00074E-10
ENSMUSG00000027249	F2	-9.297617786	0.043890021
ENSMUSG00000054422	Fabp1	-9.394690567	0.041290174
ENSMUSG00000069922	Ces3a	-9.412093865	0.040857863
ENSMUSG00000032081	Apoc3	-9.462205419	0.000171542
ENSMUSG00000022875	Kng1	-10.02726836	0.027254605
ENSMUSG00000110439	Mup22	-10.03258685	0.027156648
ENSMUSG00000059481	Plg	-10.06153791	0.02663078
ENSMUSG00000096688	Mup17	-10.09080505	0.026090568
ENSMUSG00000028356	Ambp	-10.19729245	0.024248396
ENSMUSG00000078672	Mup20	-10.34791904	0.021845472
ENSMUSG00000071177	Serpina1d	-10.60161921	0.018304378
ENSMUSG00000035540	Gc	-10.92911704	0.014492021
ENSMUSG00000061808	Ttr	-10.97072059	0.01404725

Supplemental Table 6. Primer sequences for RT-qPCR

Species	genes	Primer sequence (5' - 3')
Mouse	IAMP	TGAGCTGGATCTGGACTGAA; TCTCTCAGCTTCACGTTTGG
Mouse	Pecam1	CGGTGTTCAGCGAGATCC; ACTCGACAGGATGGAAATCAC
Mouse	Postn	AAGCTGCGGCAAGACAAG; TCAAATCTGCAGCTTCAAGG
Mouse	Tnnt2	GGCTCACTTCGAGAACAGGA; TCATTGCGAATACGCTGCT
Mouse	Cebpb	CAACCTGGAGACGCAGCACAAG; GCTTGAACAAGTTCCGCAGGGT
Mouse	Cxcl5	CCGCTGGCATTTCTGTTGCTGT; CAGGGATCACCTCCAAATTAGCG
Mouse	Ccl7	CAGAAGGATCACCAGTAGTCGG; ATAGCCTCCTCGACCCACTTCT
Mouse	Cxcl3	TGAGACCATCCAGAGCTTGACG; CCTTGGGGGTTGAGGCAAACTT
Mouse	Gsta1	CTGCCTTGGCAAAAGATAGGACC; CTTCCAGTAGGTGGATGTCCAC
Mouse	Mgst1	TGCGACCGCATTCCAGAGGATA; TCCACCTTCTCGTCAGTGCGAA
Mouse	Ugt1a2	ATGCCAAGCGCATGGAAACTCG; ATAGGACGGTCCTTGTGAAGGC
Mouse	Ugt1a6a	GCTGAGGCTTTGGGCAGAATTC; GTGATGAATGCCCGAGTCTTTGG
Mouse	Ccna1	GCTACTGAGGATGGAGCATCTG; CAGCTTCCAGAAGGCTCAGTTC
Mouse	Mcm2	CCGTTCCAAGGATGCCATTCTC; TGGAAAGCCGTTGGCGGTGTTA
Mouse	Plk1	CCATCTTCTGGGTCAGCAAGTG; CCGTCATTGTAGAGAATCAGGCG
Mouse	Wee1	CCATTGGCTGGCTCTGTTGATG; CAGGCAGAGAAATAGCGAACGAC
Mouse	Gtse1	AGAGGATCACCAGCAAGCTCCA; GTTTCGTCCTCTGAATGCTGGC
Mouse	Sesn1	GCAGTTACAGGAATGCCGAGAG; CCTCAAAGTGCCGAGACACATC
Mouse	Pten	TGAGTTCCCTCAGCCATTGCCT; GAGGTTTCCTCTGGTCCTGGTA
Mouse	Il6	TACCACTTCACAAGTCGGAGGC; CTGCAAGTGCATCATCGTTGTTC
Mouse	S100a8	CAAGGAAATCACCATGCCCTCTA; ACCATCGCAAGGAACTCCTCGA
Mouse	Mmp3	CTCTGGAACCTGAGACATCACC; AGGAGTCCTGAGAGATTTGCGC
Mouse	Cxcl1	TCCAGAGCTTGAAGGTGTTGCC; AACCAAGGGAGCTTCAGGGTCA
Mouse	Il1b	AGTTGACGGACCCCAAAAG; AGCTGGATGCTCTCATCAGG
Mouse	Col1a1	CATGTTCAGCTTTGTGGACCT; GCAGCTGACTTCAGGGATGT
Mouse	Col3a1	TCCCCTGGAATCTGTGAATC; TGAGTCGAATTGGGGAGAAT
Mouse	Acta2	CCAGCACCATGAAGATCAAG; TCCACATCTGCTGGAAGGTA
Mouse	Nppa	CACAGATCTGATGGATTTCAAGA; CCTCATCTTCTACCGGCATC
Mouse	Nppb	GTCAGTCGTTTGGGCTGTAAC; AGACCCAGGCAGAGTCAGAA
Mouse	Acta1	GCCCATCTATGAGGGCTATG; AATCTCACGTTCAGCTGTGG
Mouse	Hif1a	CCTGCACTGAATCAAGAGGTTGC; CCATCAGAAGGACTTGCTGGCT
Mouse	Ctgf	TGACCTGAGGAAAACATTAAGA; AGCCCTGTATGTCTTCACACTG
Mouse	Nfkb1	GCTGCCAAAGAAGGACACGACA; GGCAGGCTATTGCTCATCACAG
Mouse	Nfkbia	GCCAGGAATTGCTGAGGCACTT; GTCTGCGTCAAGACTGCTACAC
Mouse	Chuk	TCGGAAACCAGCCTCTCAGTGT; CTTCTGGATGCAAATGGTCCTTC
Mouse	Ikbkb	GCAGACTGACATTGTGGACCTG; ATCTCCTGGCTGTCACCTTCTG
Mouse	Rela	TCCTGTTCGAGTCTCCATGCAG; GGTCTCATAGGTCCTTTTGCGC
Human	IAMP	TTTTGCTGCTGTGTGTGGA; TTTATTCCTCTTCCAGCTTTGG
Human	Il6	AGACAGCCACTCACCTCTTCAG; TTCTGCCAGTGCCTCTTTGCTG
Rat	IAMP	GAACAGATCCAGCGGTCCT; AAGCCACTGGGAGCACCT
Human	Gapdh	GTCTCCTCTGACTTCAACAGCG; ACCACCCTGTTGCTGTAGCCAA
Mouse	Gapdh	TTCCTCGTCCCGTAGACAAAATGGT; CATTTGATGTTAGTGGGGTCTCGCT
Mouse/Human/Rat	18s	TCCGACCATAAACGATGCCG; CAATCTGTCAATCCTGTCCGTGTC
