# Supplementary material for: A cardiac fibroblast-enriched micropeptide regulates inflammation in ischemia/reperfusion injury
Source: JCI Insight. 2025 Mar 20;10(9):e187848. doi: 10.1172/jci.insight.187848 (PMC12128956; doi:10.1172/jci.insight.187848)

Full unedited blot for Figure1-D

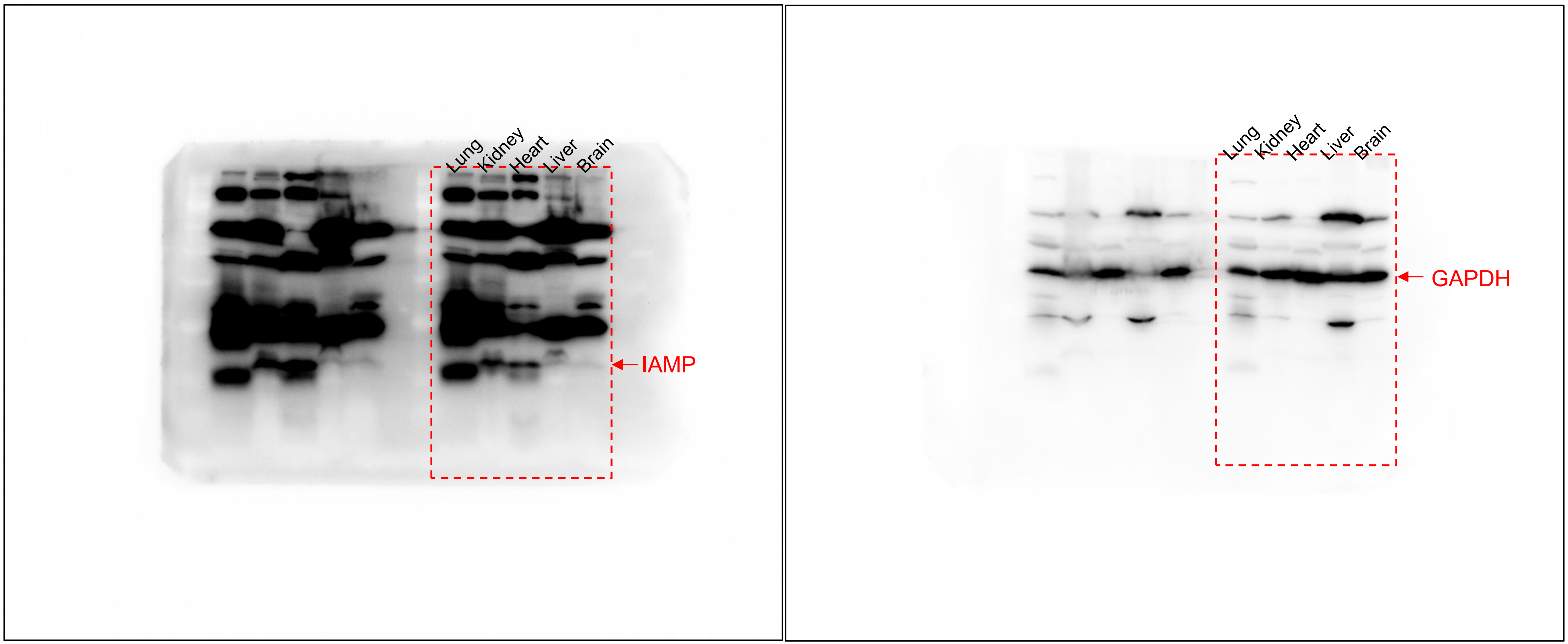

Full unedited blot for Figure1-I

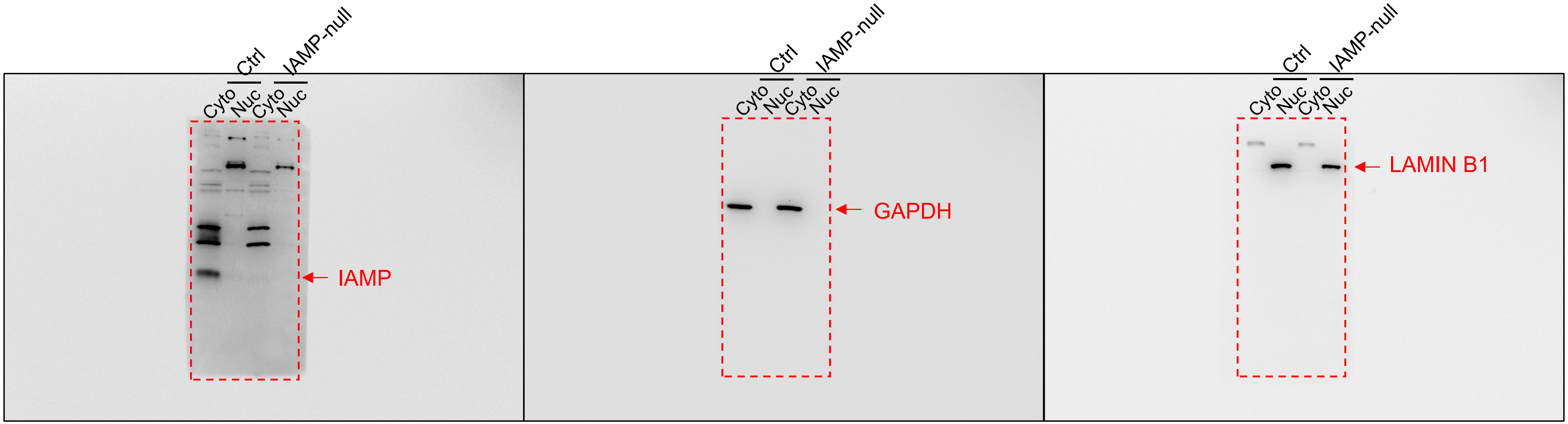

Full unedited blot for Figure2-B

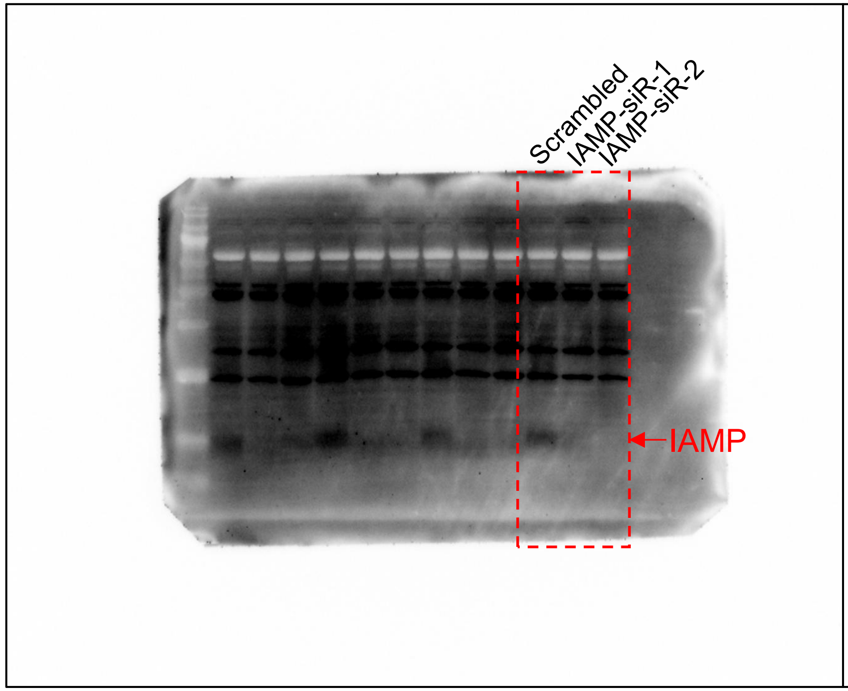

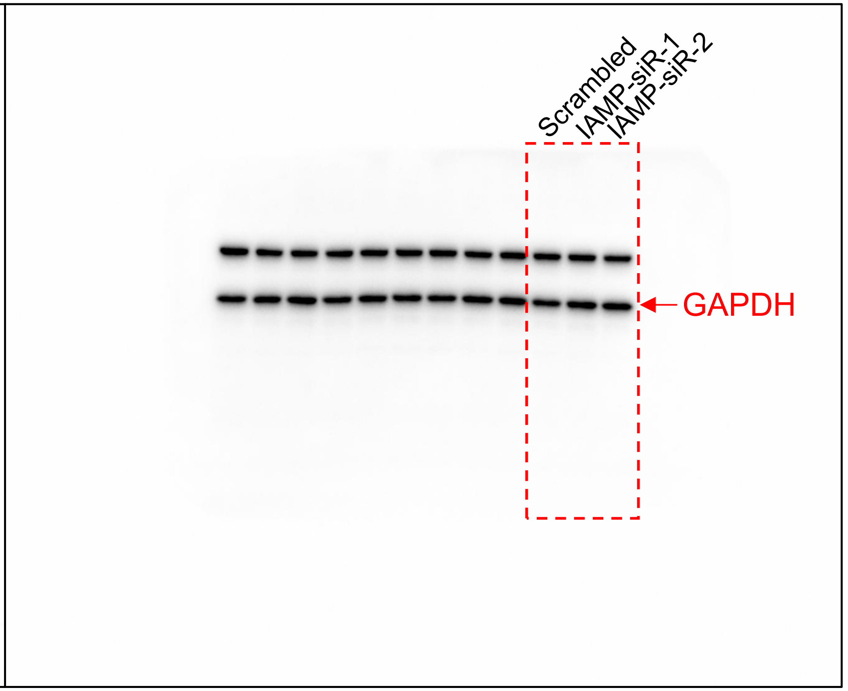

Full unedited blot for Figure3-I

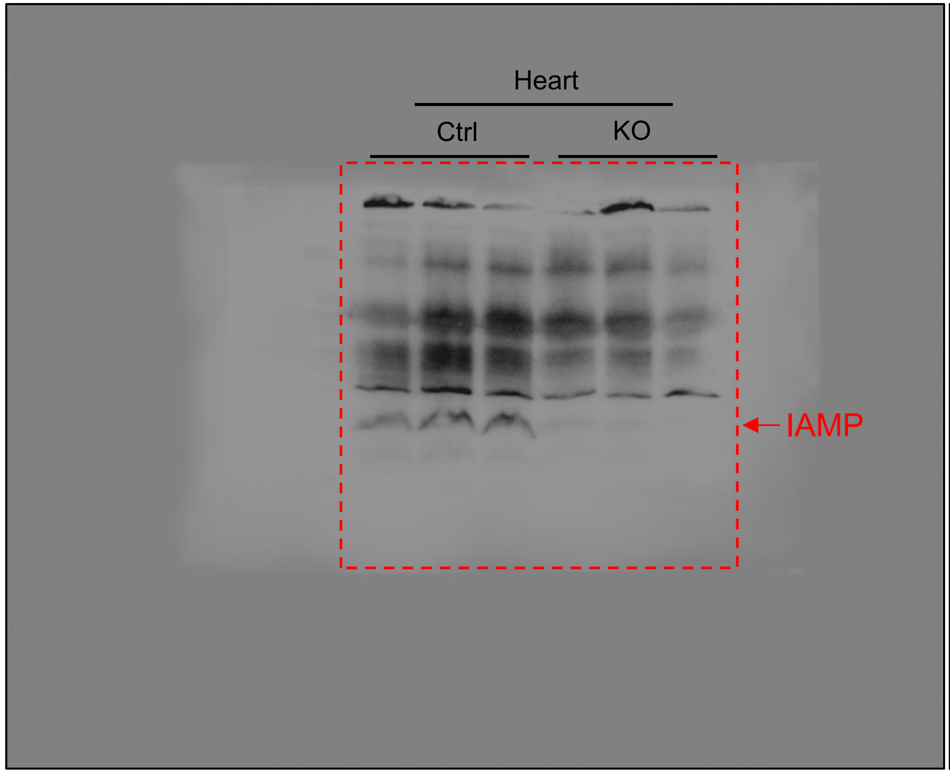

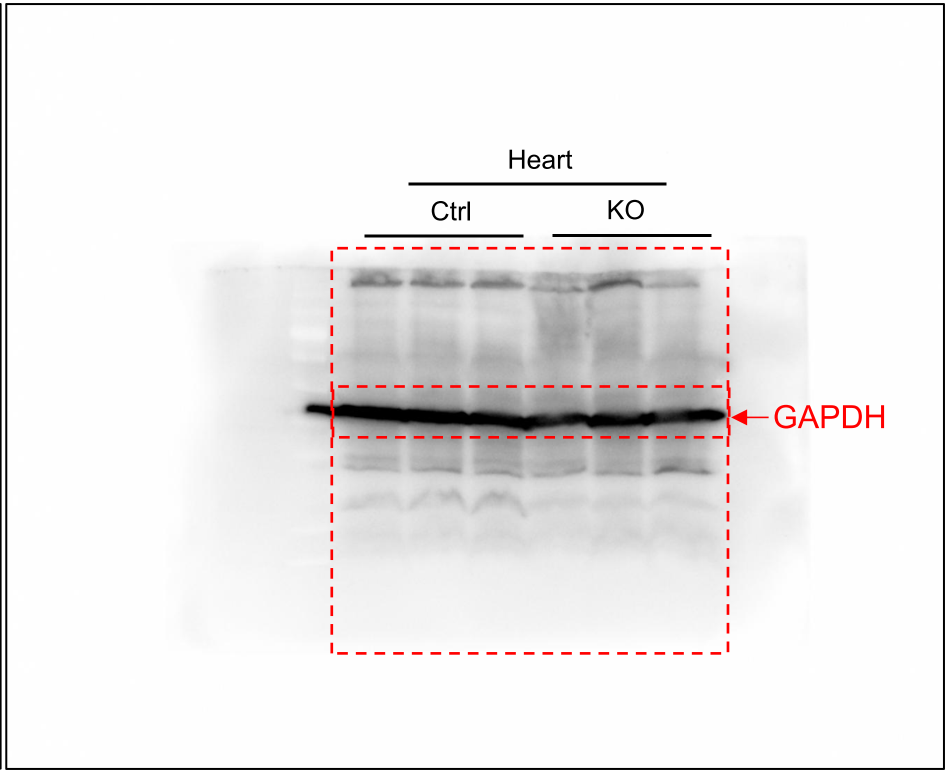

Full unedited blot for Figure3-J

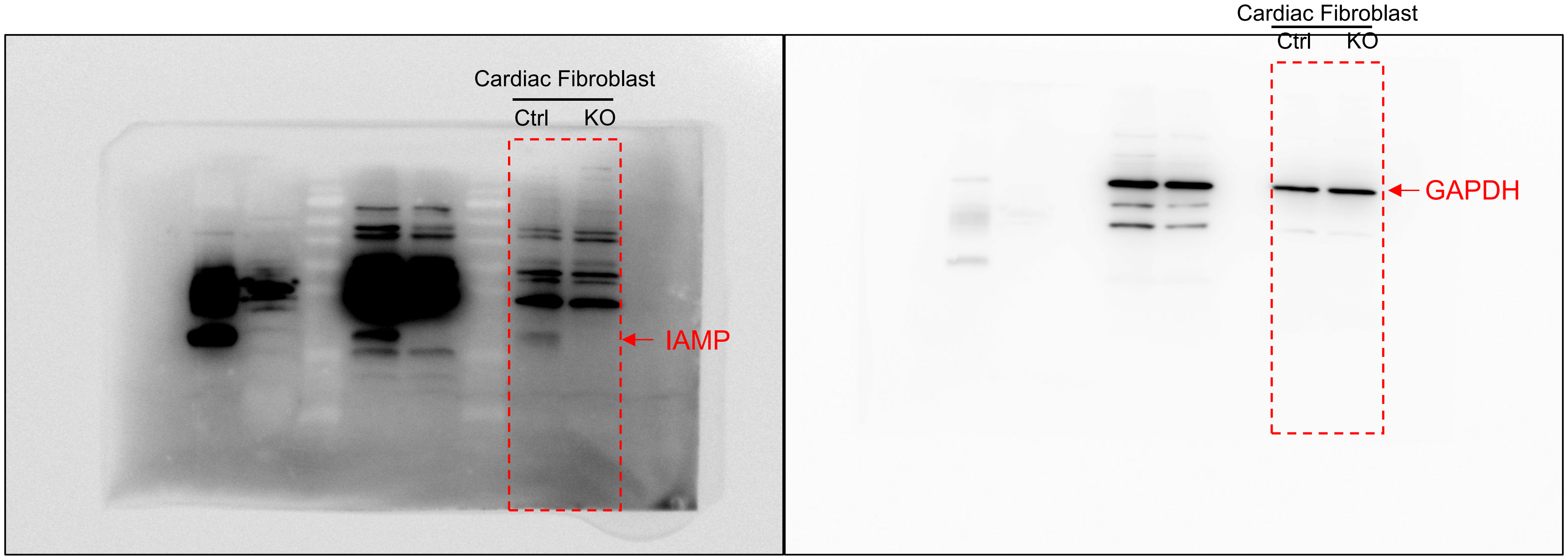

Full unedited blot for Figure5-E

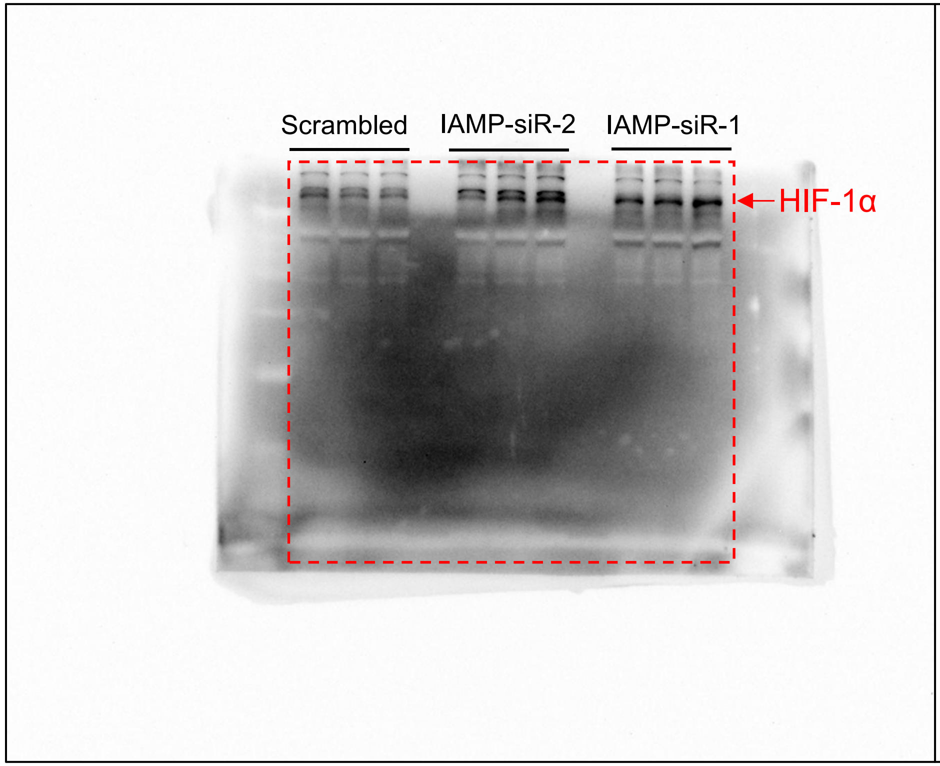

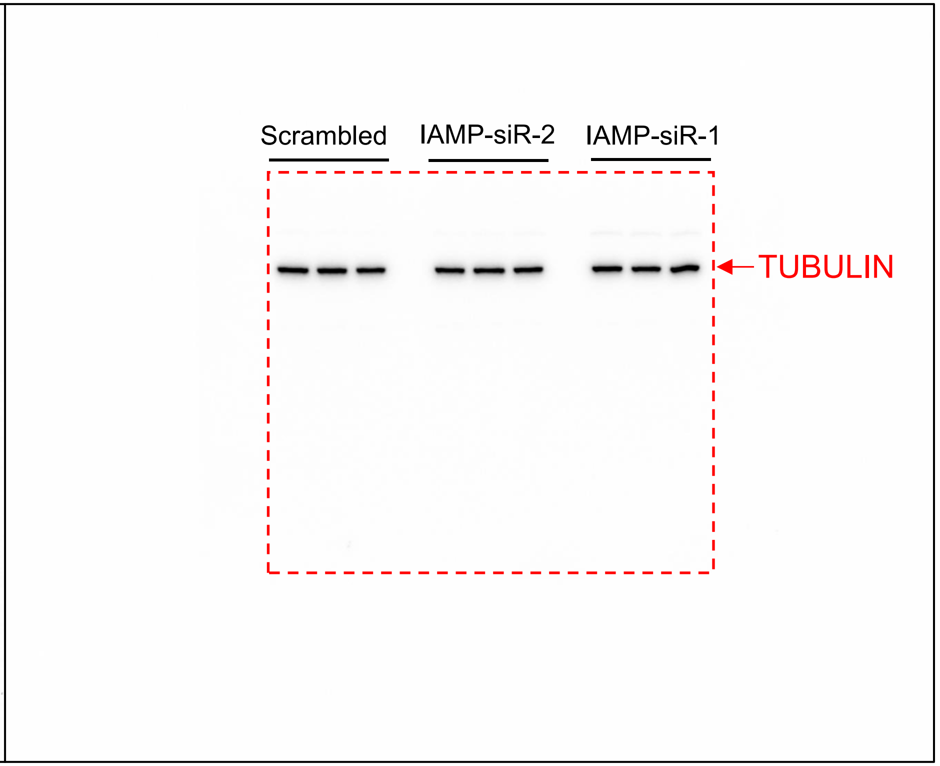

Full unedited blot for Figure5-F

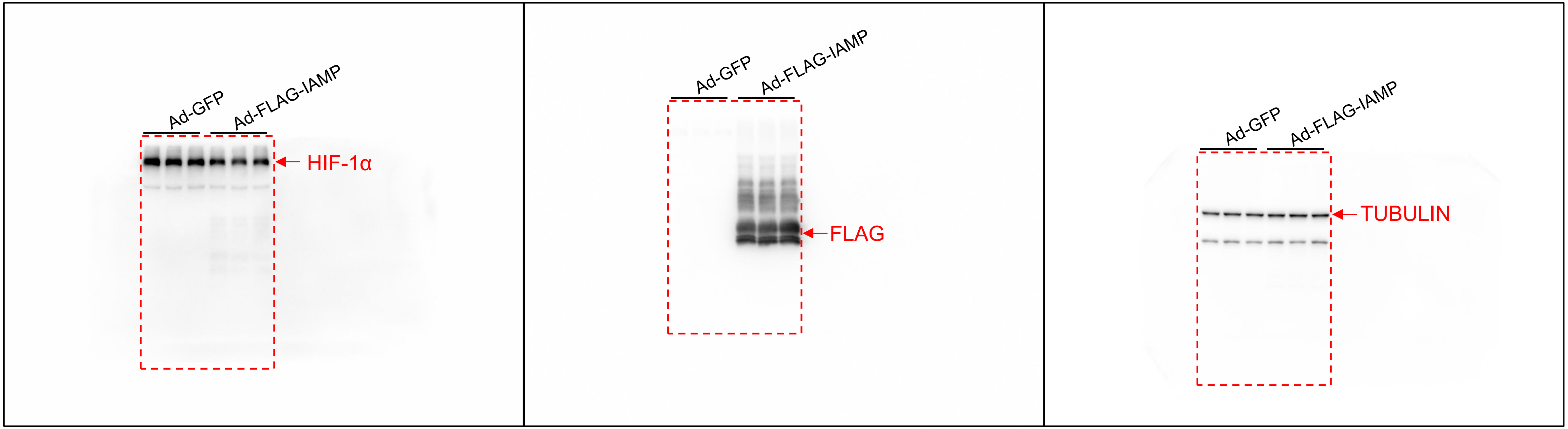

Full unedited blot for Figure5-G

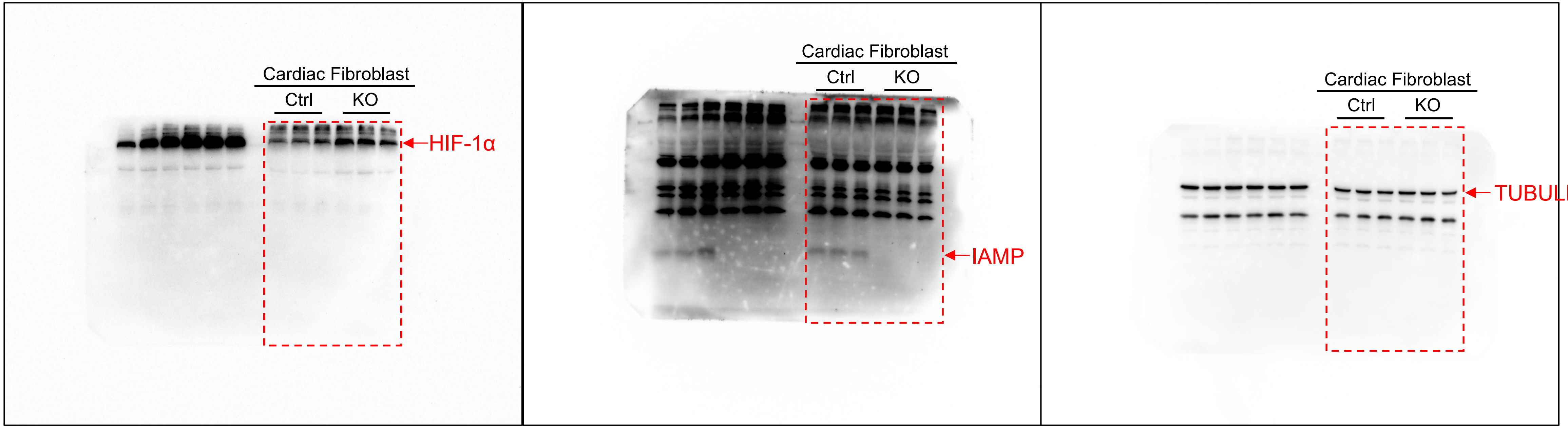

Full unedited blot for Figure6-A

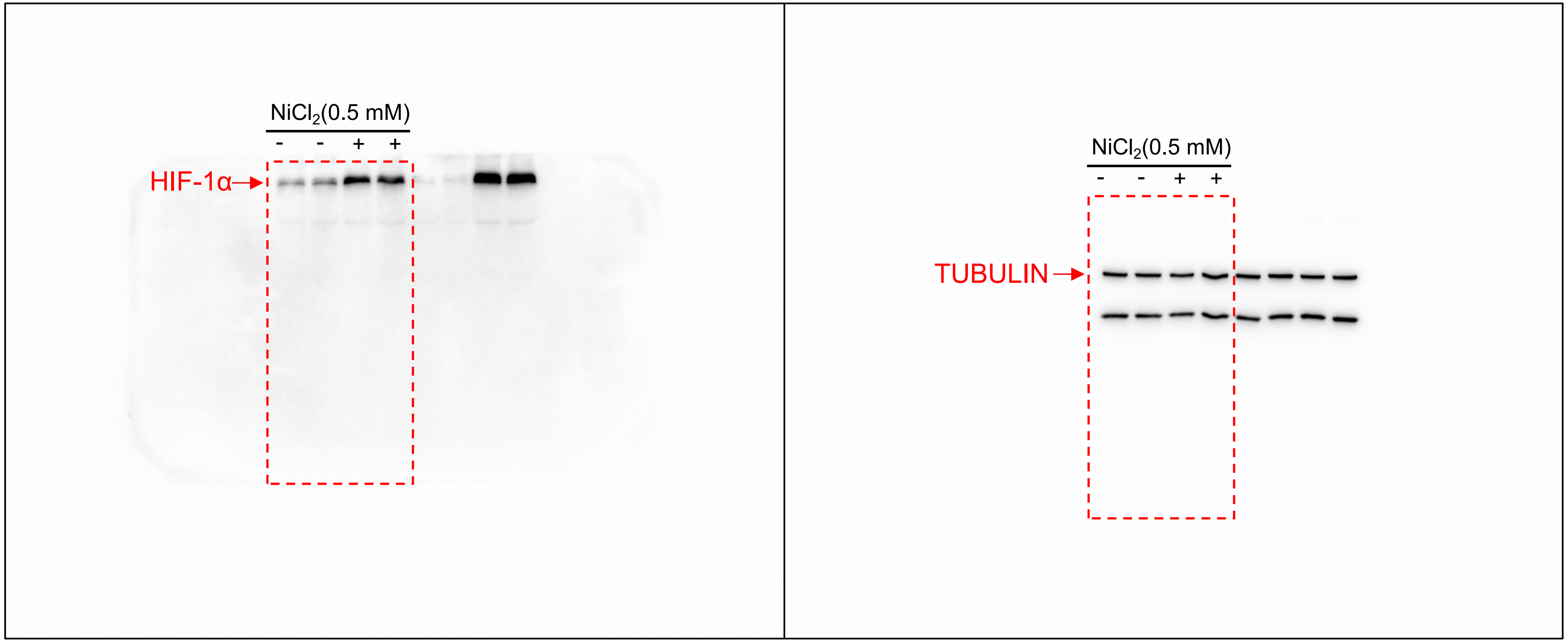

Full unedited blot for Figure6-C

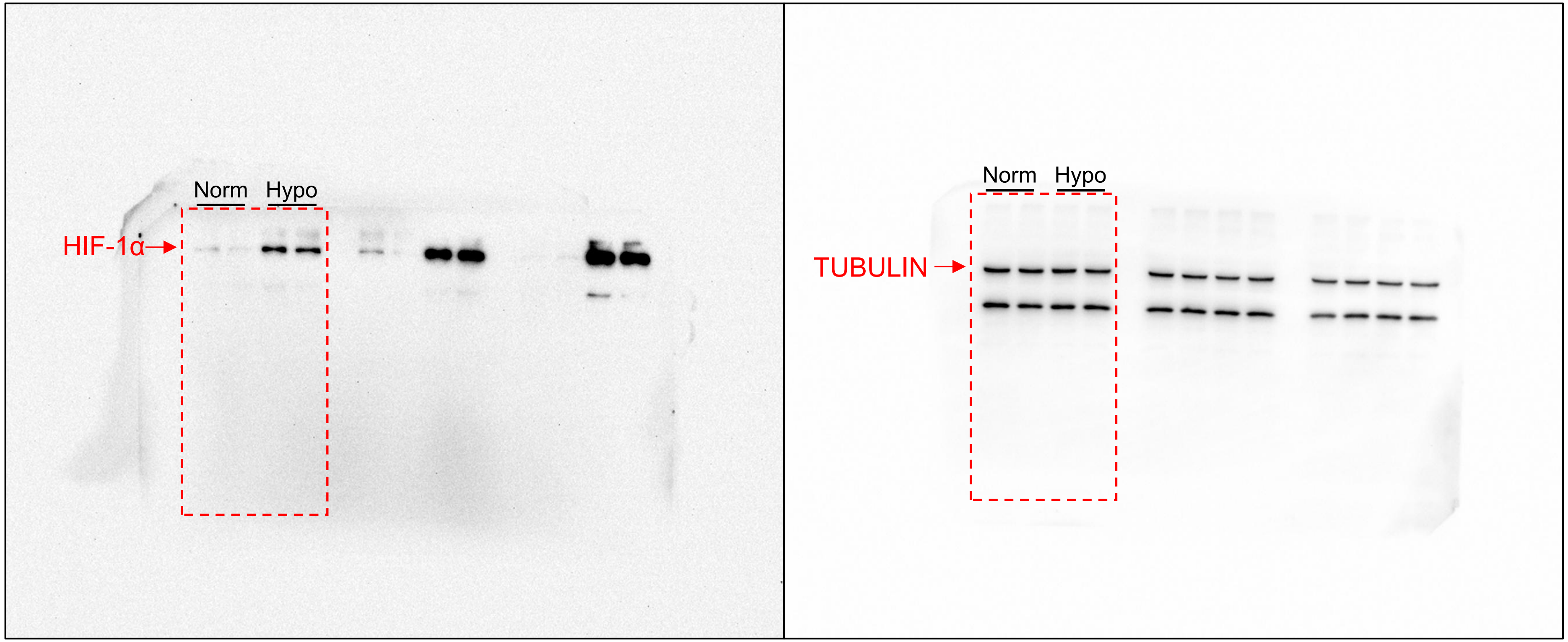

Full unedited blot for Figure7-A

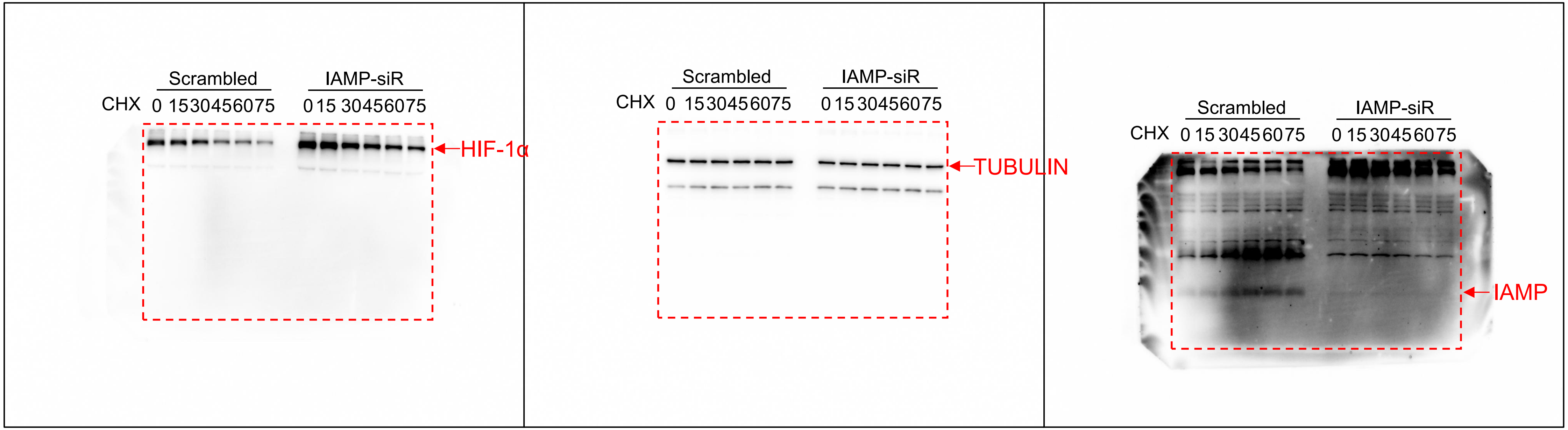

Full unedited blot for Figure7-B

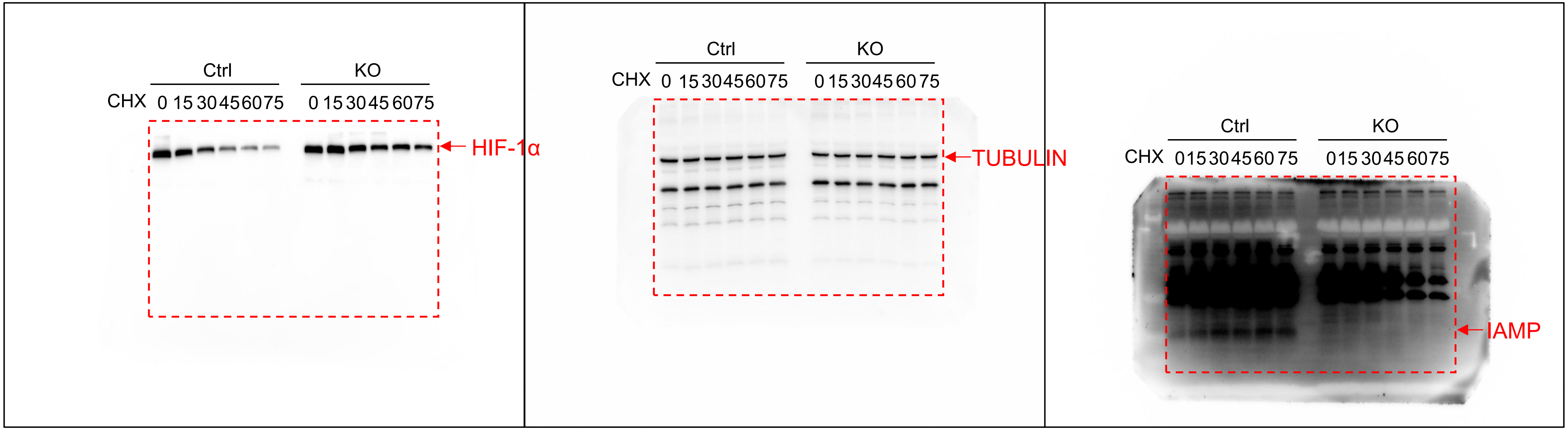

Full unedited blot for Figure7-C

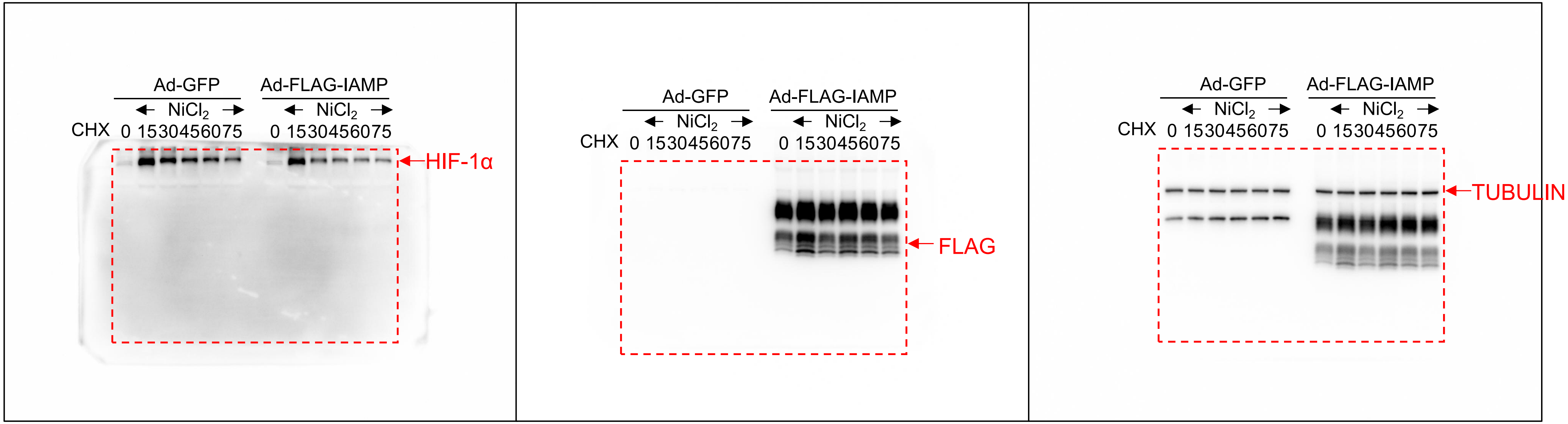

Full unedited blot for Figure7-D

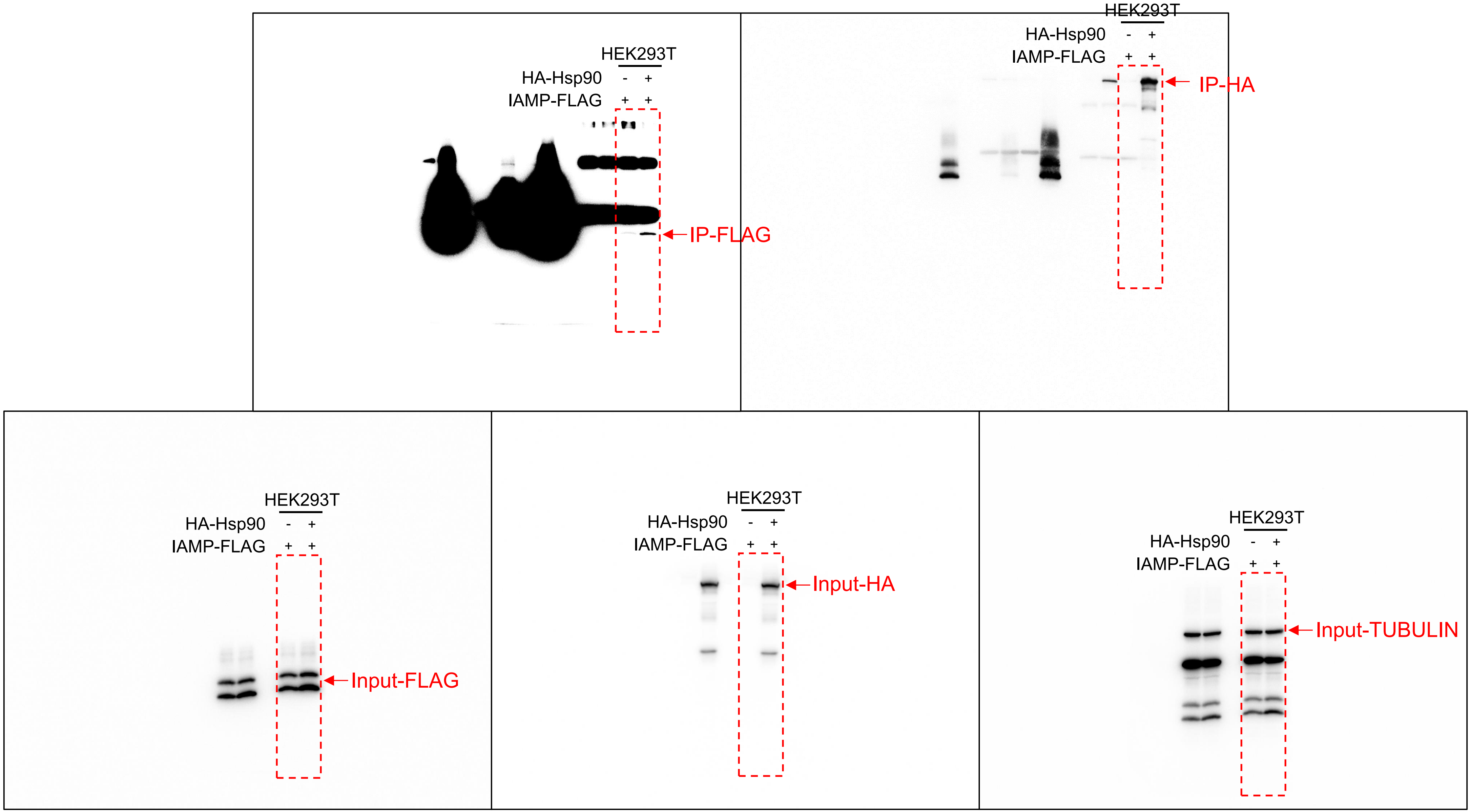

Full unedited blot for Figure7-E

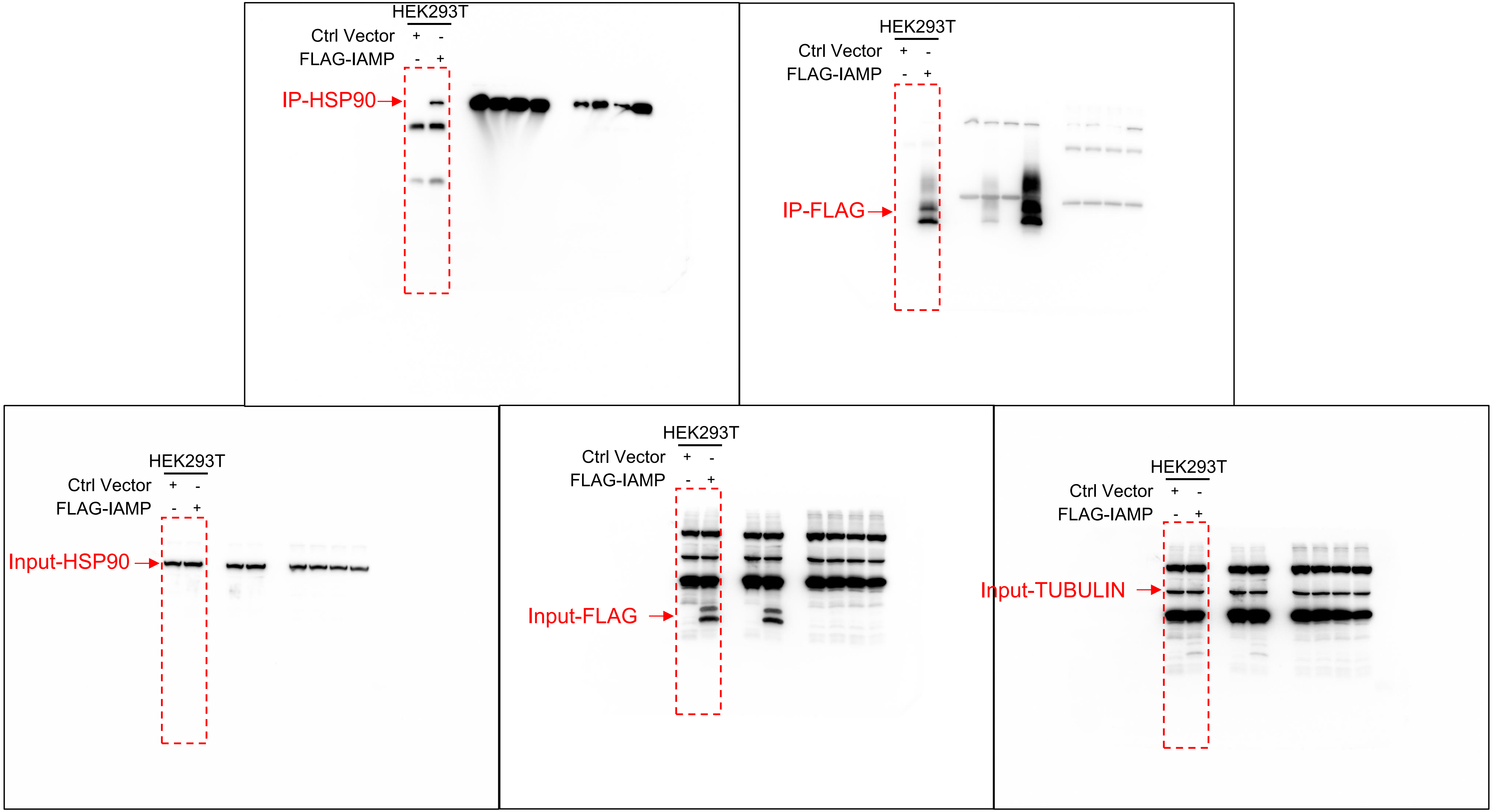

Full unedited blot for Figure7-F

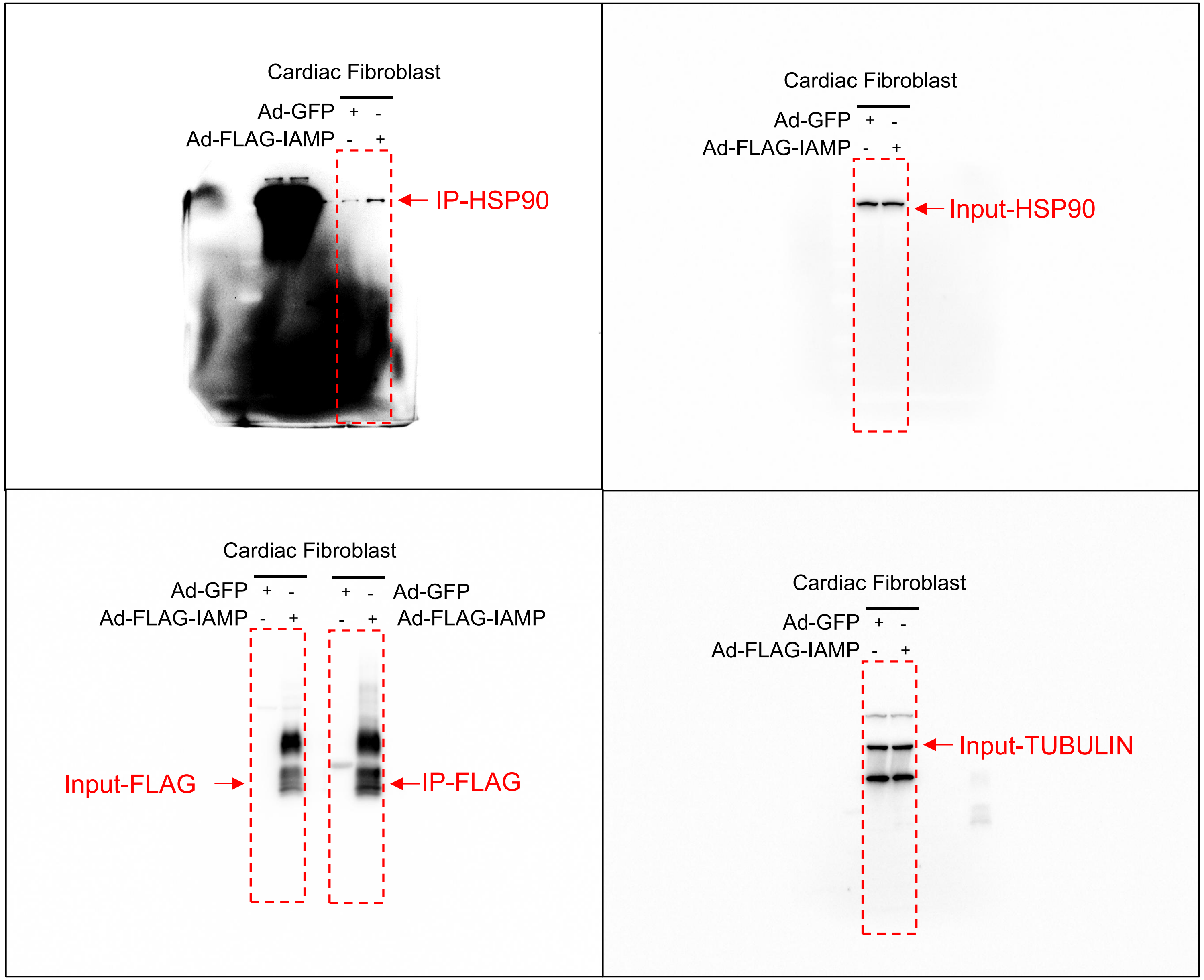

Full unedited blot for Figure7-G

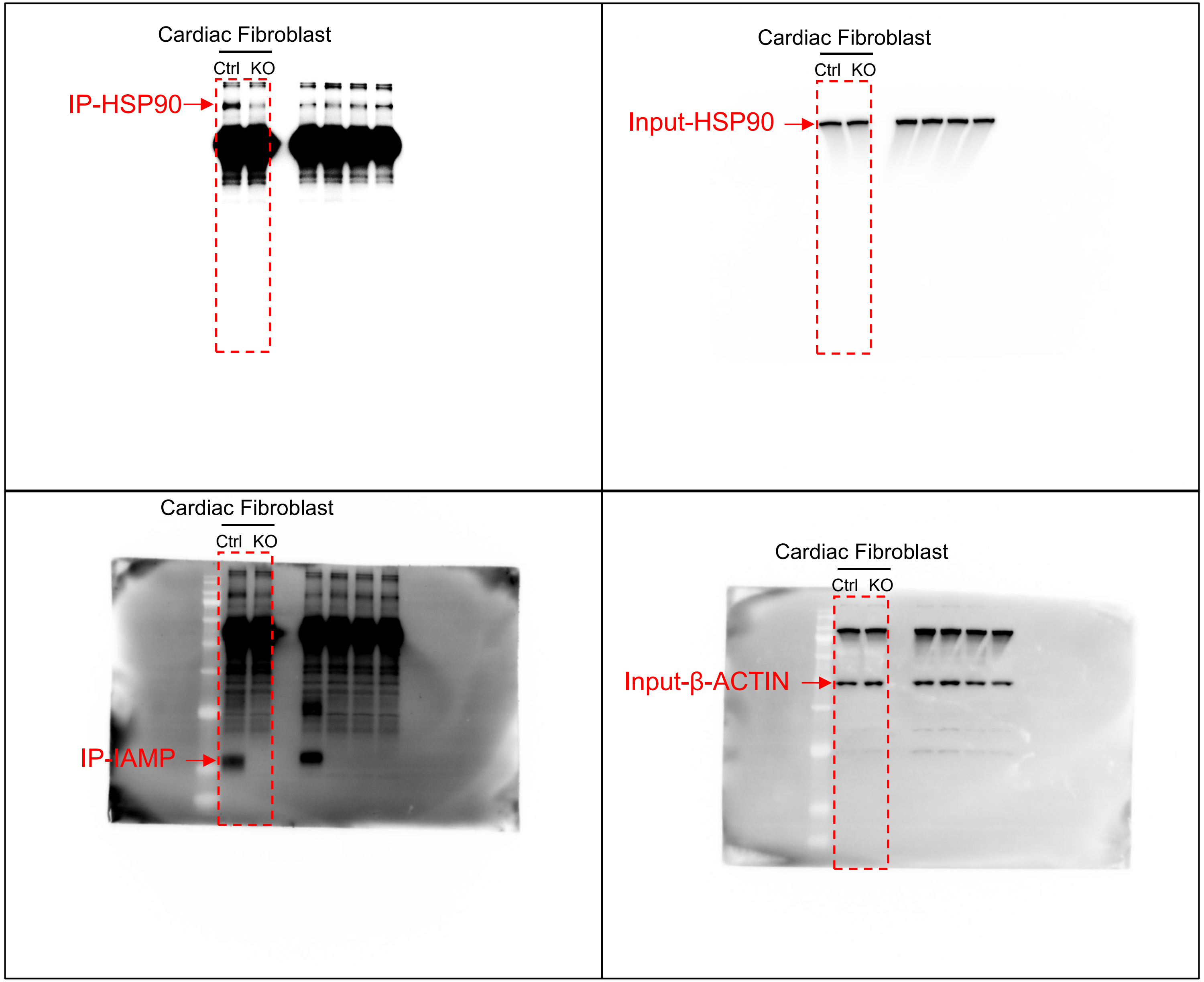

Full unedited blot for Figure7-H

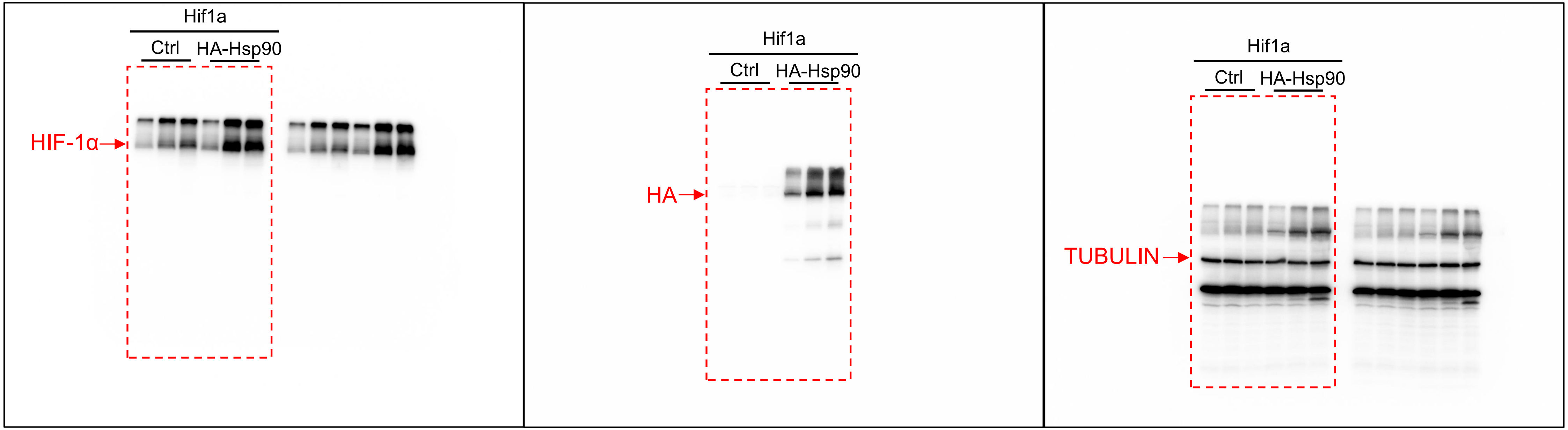

Full unedited blot for Figure7-I

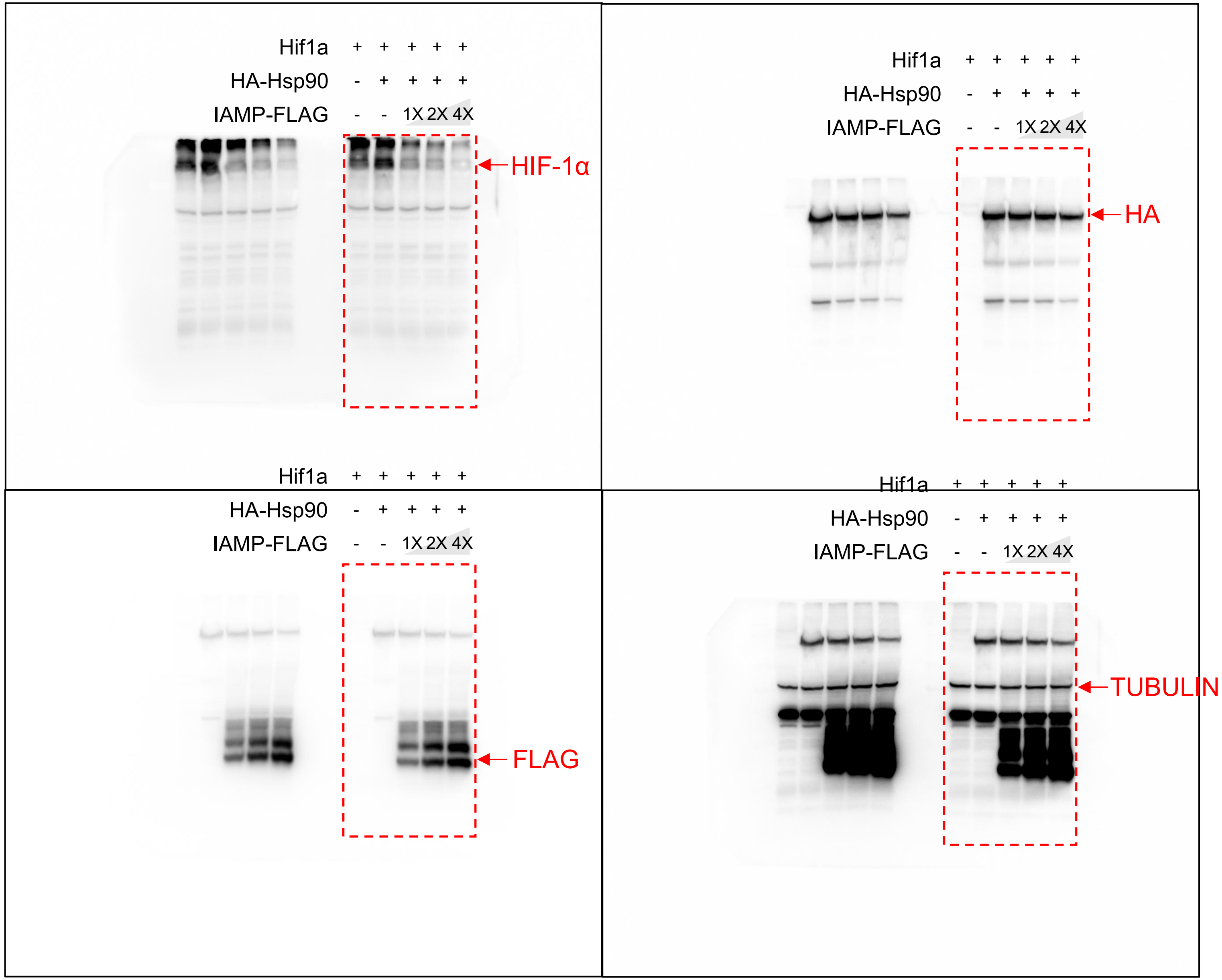

Full unedited blot for Figure7-J

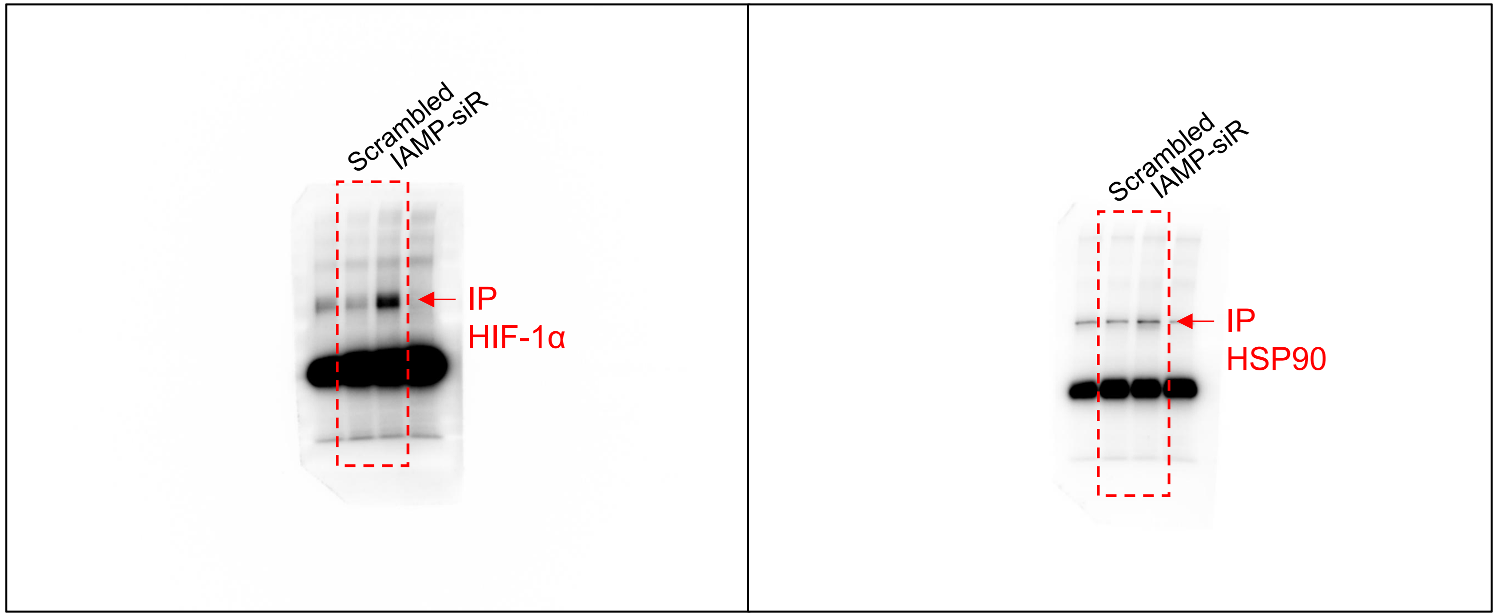

Full unedited blot for Figure7-K

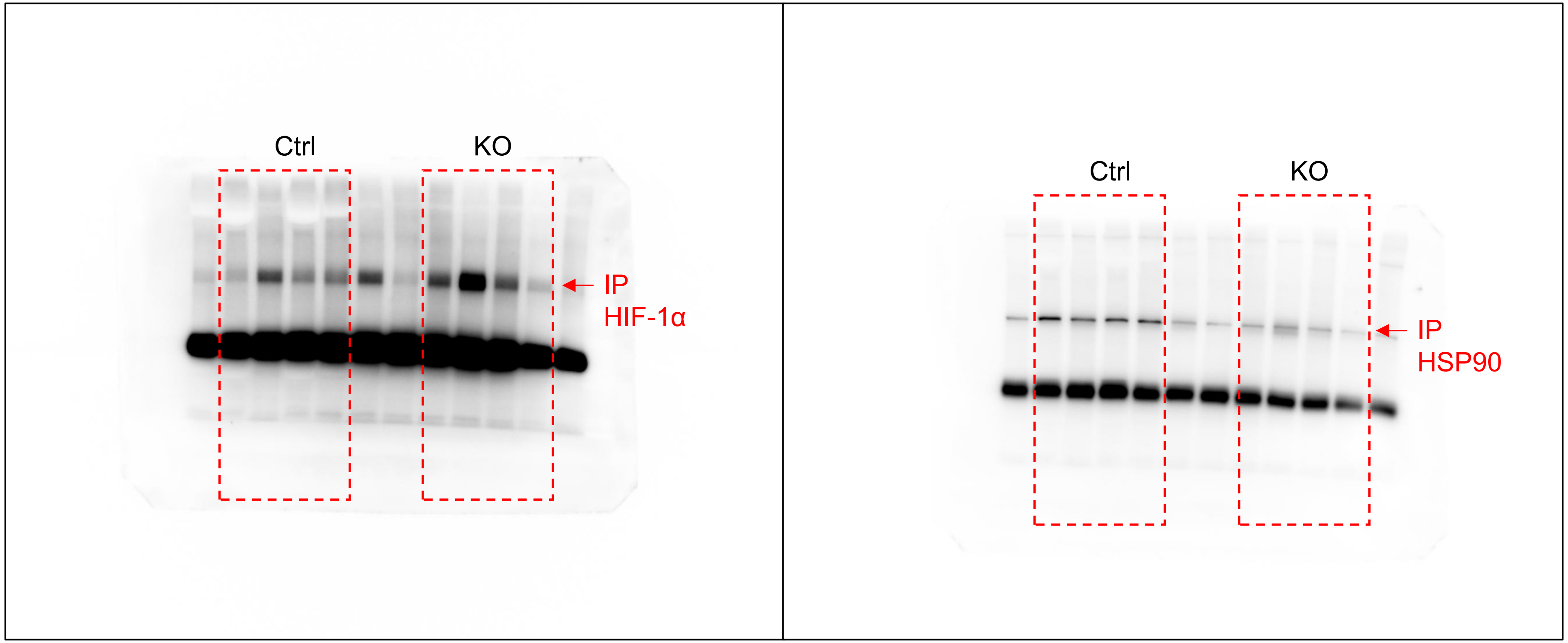

Full unedited blot for Supplemental Figure3-B

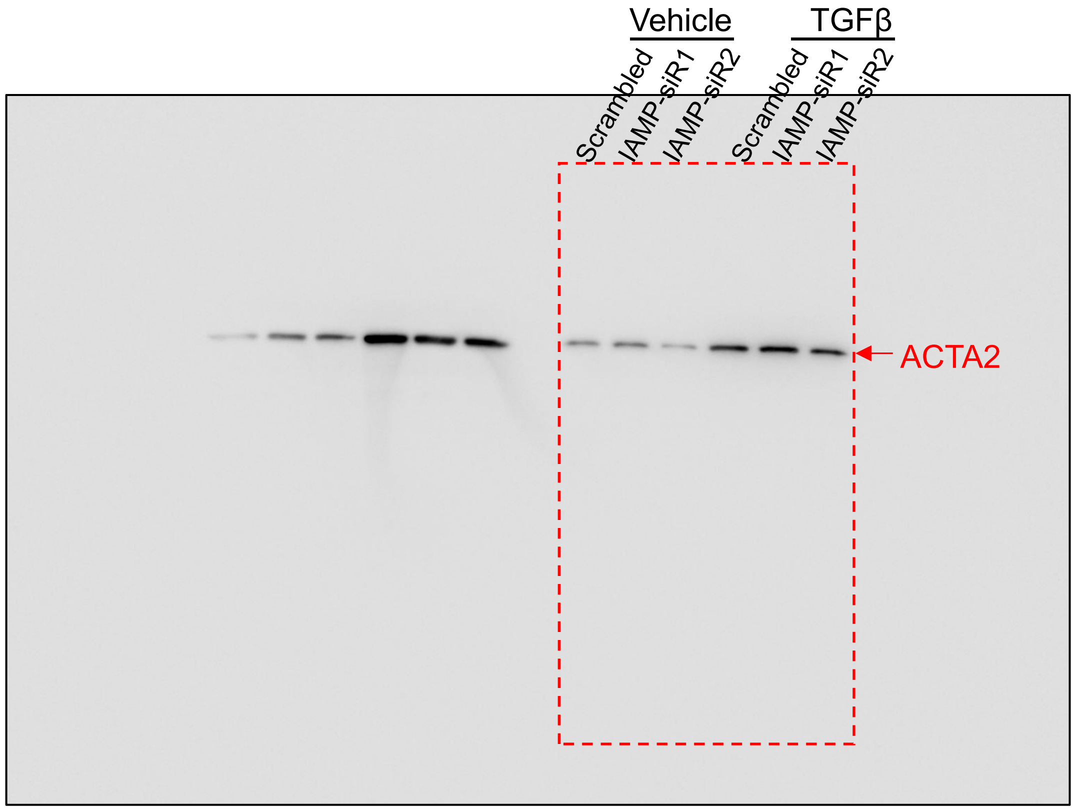

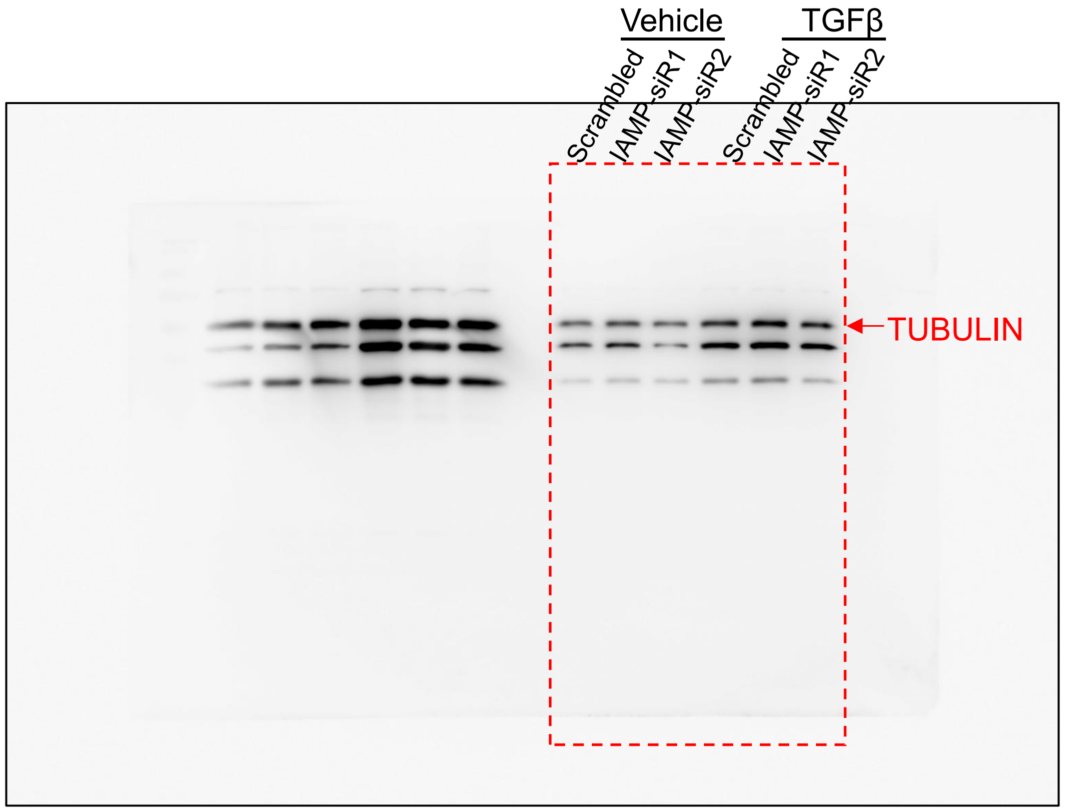

Full unedited blot for Supplemental Figure4-B

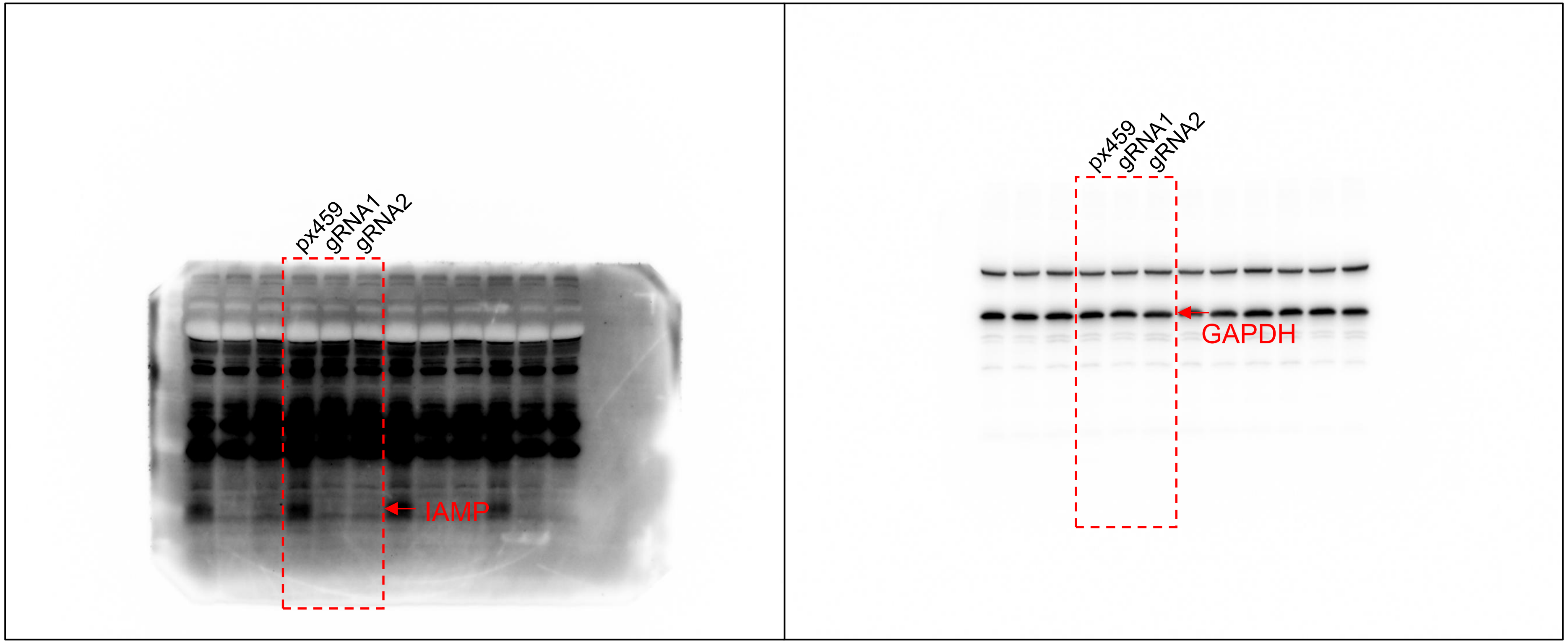

Full unedited blot for Supplemental Figure7-A

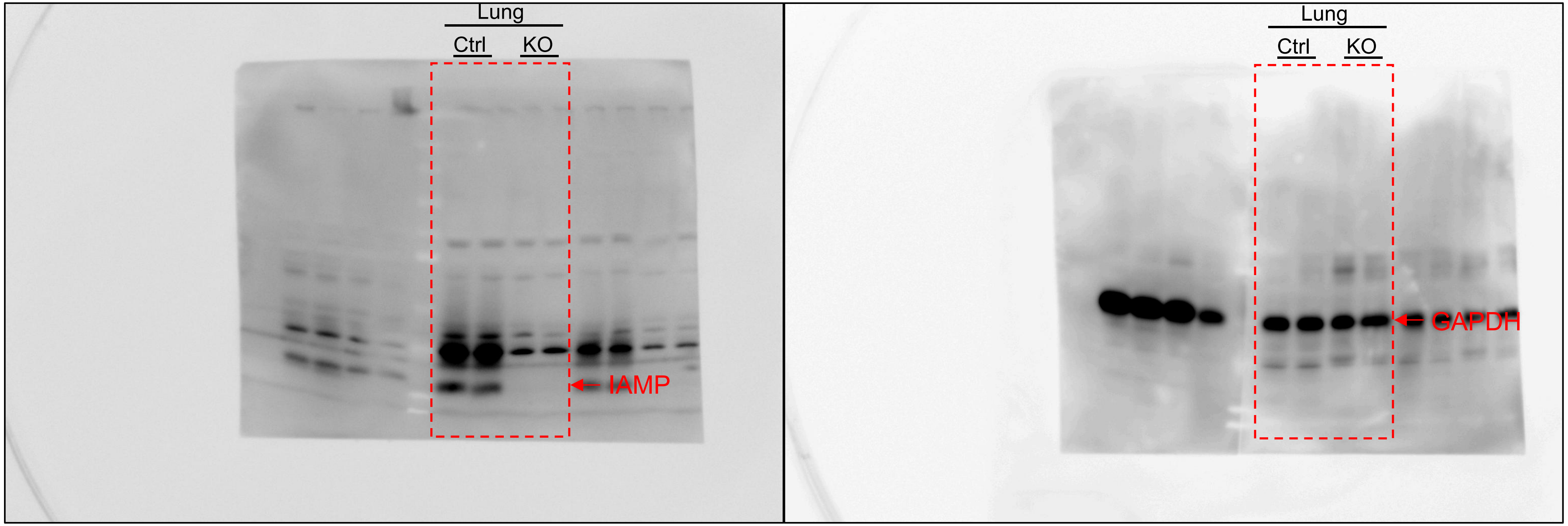

Full unedited blot for Supplemental Figure 9-A

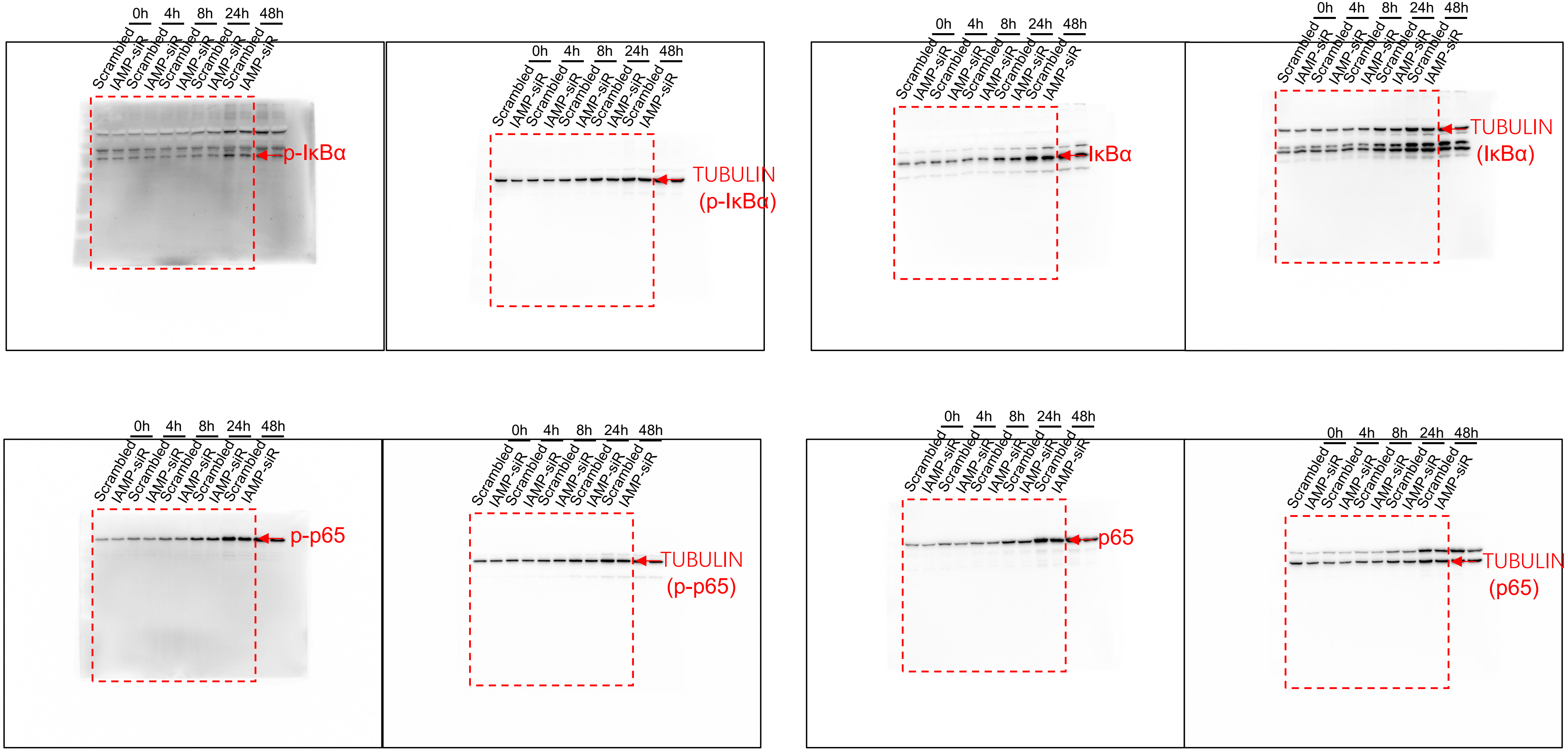

Full unedited blot for Supplemental Figure 9-B

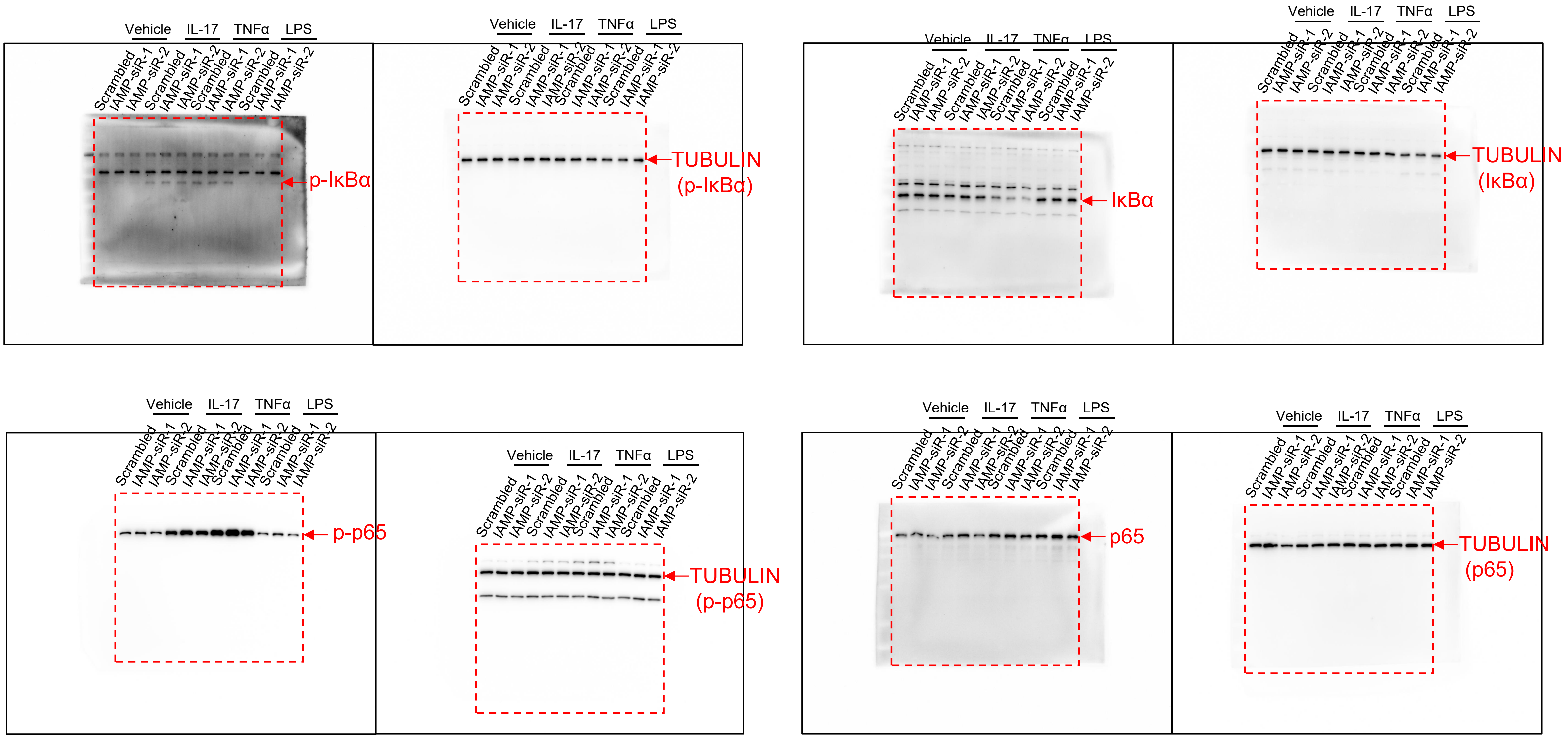

Full unedited blot for Supplemental Figure 10-A

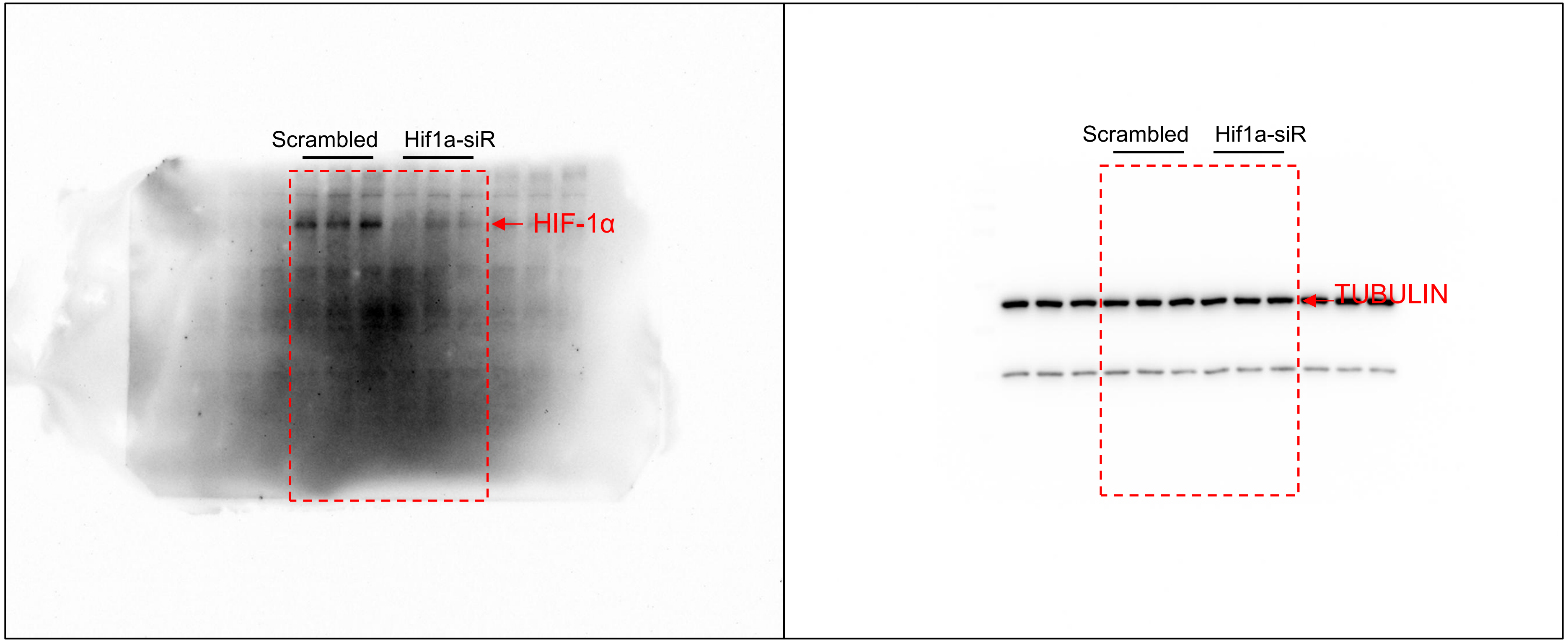

Supplement: Unedited blot and gel images [file jciinsight-10-187848-s238.pdf]
